# Variations in the non-coding transcriptome as a driver of inter-strain divergence and physiological adaptation in bacteria

**DOI:** 10.1038/srep09560

**Published:** 2015-04-22

**Authors:** Matthias Kopf, Stephan Klähn, Ingeborg Scholz, Wolfgang R. Hess, Björn Voß

**Affiliations:** 1 Genetics and Experimental Bioinformatics, Faculty of Biology, University of Freiburg, Schänzlestr. 1, 79104 Freiburg, Germany

## Abstract

In all studied organisms, a substantial portion of the transcriptome consists of non-coding RNAs that frequently execute regulatory functions. Here, we have compared the primary transcriptomes of the cyanobacteria *Synechocystis* sp. PCC 6714 and PCC 6803 under 10 different conditions. These strains share 2854 protein-coding genes and a 16S rRNA identity of 99.4%, indicating their close relatedness. Conserved major transcriptional start sites (TSSs) give rise to non-coding transcripts within the *sigB* gene, from the 5′UTRs of *cmpA* and *isiA*, and 168 loci in antisense orientation. Distinct differences include single nucleotide polymorphisms rendering promoters inactive in one of the strains, e.g., for *cmpR* and for the asRNA PsbA2R. Based on the genome-wide mapped location, regulation and classification of TSSs, non-coding transcripts were identified as the most dynamic component of the transcriptome. We identified a class of mRNAs that originate by read-through from an sRNA that accumulates as a discrete and abundant transcript while also serving as the 5′UTR. Such an sRNA/mRNA structure, which we name ‘actuaton’, represents another way for bacteria to remodel their transcriptional network. Our findings support the hypothesis that variations in the non-coding transcriptome constitute a major evolutionary element of inter-strain divergence and capability for physiological adaptation.

Organismic diversity as well as differences in metabolic, developmental and physiological capabilities cannot be related to divergent gene content and gene arrangement alone. Instead, differences in the regulation of gene expression and the composition of the transcriptome have been suggested as critical factors^1^. Accordingly, a substantial share of the transcriptome consists of non-coding and antisense RNAs, many of which have regulatory impact, e.g., in the form of miRNAs^2^, long non-coding RNAs^3^ or long natural antisense transcripts^4^. It is widely accepted that RNA complexity is at the heart of biological complexity^5^.

For prokaryotic organisms, it has long been thought that regulatory and transcriptomic divergence is less relevant because genomic differences, higher mutation rates and horizontal gene transfer provide sufficient means for rapid adaptation to various environments. Moreover, most bacterial genomes are relatively compact and have a large protein-coding fraction, leaving less room for non-coding transcripts. However, the discovery of large numbers of sRNAs, including asRNAs^6^,^7^,^8^,^9^,^10^,^11^,^12^,^13^, and of their versatile roles in regulatory processes, especially during stress adaptation, have clearly demonstrated the relevance of non-coding RNA in prokaryotes^14^,^15^,^16^.

Genomic comparisons between closely related bacteria have been pivotal in gaining insight into their metabolic potential, regulatory networks and genome evolution. In contrast, the number of inter-strain or inter-species transcriptomic comparisons has remained relatively scarce so far. Differential RNA-seq-type transcriptomic analyses (dRNA-seq^7^) are especially powerful, as this technique enables the identification of TSSs at a genome-wide scale at single-nucleotide resolution and can easily identify sRNAs as well as transcripts that originate within genes in either orientation. Thus, the detailed information on TSSs provided by dRNA-seq gives deep insight into the transcriptional landscape of an organism. Comparative transcriptomics has proven useful at inferring the dynamics of transcriptional regulation by analysing regulatory responses to different conditions. Such an analysis compared primary transcriptomes of the human pathogen *Helicobacter pylori* under the mid-logarithmic growth phase versus acid stress conditions, mimicking the host environment^7^. A comparative analysis of the primary transcriptome of the cyanobacterium *Anabaena* sp. PCC 7120 revealed more than 10,000 TSSs active during the differentiation of N_2_-fixing heterocysts, of which >900 TSSs exhibited minimum fold changes (FCs) of eight, suggesting a large number of unidentified regulators of cell differentiation and N_2_-fixation^9^.

There are very few double-comparative transcriptomic approaches in which the responses of two different but closely related organisms to multiple environmental conditions have been studied. The comparison of the primary transcriptomes of pathogenic *L. monocytogenes* and non-pathogenic *L. innocua* species under mid-log and stationary growth phases led to the discovery of 33 sRNAs and 53 asRNAs in *L. monocytogenes*^11^. Interestingly, some were not expressed in one of the species, although they were conserved at the DNA level, which indicates the importance of transcriptomic analyses and suggests a possible additional layer of divergence^11^. Comparative analysis of samples from mid-logarithmic growth stages of three isolates of the human pathogen and one isolate of the chicken pathogen *Campylobacter jejuni* revealed conserved as well as strain-specific TSSs and detected 15 conserved and 24 strain-specific sRNA candidates^17^. The comparison of transcriptome profiles of the model cyanobacteria *Synechococcus* sp. PCC 7942 and *Synechocystis* sp. PCC 6803 (from here: *Synechocystis* 6803) revealed substantial differences in the transcriptional response to environmental fluctuations^18^, which in fact may be linked to the relatively large taxonomic distance between the two species, indicated by the 10% divergence in their 16S ribosomal RNA sequences.

To address the extent to which bacterial transcriptome organisation and composition is conserved and functionally relevant, here we performed a multi-condition, double-comparative transcriptomic analysis of two closely related strains of the unicellular cyanobacterium *Synechocystis*. In *Synechocystis* 6803, substantial pervasive transcription was reported, with ~64% of all TSSs giving rise to antisense or sRNAs in a genome that is to 87% protein coding^8^. Recently, we elucidated the response of *Synechocystis* 6803 to specific environmental conditions and identified more than 4000 transcriptional units, about half of which represent non-coding RNAs^19^. Several of these non-coding RNAs are important regulators of photosynthetic gene expression, such as the *cis*-encoded antisense RNAs (asRNAs) IsrR, As1_flv4 and PsbA2R^20^,^21^,^22^ or the *trans*-encoded sRNA PsrR1^23^. There are several more non-coding RNAs with an expression that is tightly controlled by environmental conditions^19^, and therefore are likely to be of similar importance, but their characterisation is pending^24^. Moreover, information about the conservation and expression of these non-coding RNAs in other strains would be of high interest, but is largely lacking.

*Synechocystis* sp. strain PCC 6714 (from here: *Synechocystis* 6714) is closely related to *Synechocystis* 6803 and its genome has recently been sequenced^25^. Their genomes are similar in size, number of encoded genes, fraction of non-coding DNA and 16S rDNA (99.4% identity)^26^. Both strains share 2854 protein-coding genes, leaving 829 unique genes in *Synechocystis* 6803 and 916 in *Synechocystis* 6714, and have an average nucleotide identity (ANI) of 86.4% (see Table 1). Here, we present genome-wide maps of the TSSs active in *Synechocystis* 6714 under the same 10 conditions as in the previous analysis of *Synechocystis* 6803^19^, and identified 4292 transcriptional units (TUs). The genome-wide comparison of these transcripts and the TSS maps in both strains under the different conditions revealed substantial conservation but also specific differences in the respective transcriptional organisation. In the transcriptome comparison we find the presence and expression of non-coding RNAs to constitute the evolutionarily most flexible component and identified a new genetic element, which we propose calling actuaton.

## Results

### The primary transcriptome of *Synechocystis* 6714 and its comparison to the closely related model *Synechocystis* 6803

There is not a single genome-wide study of gene expression for *Synechocystis* 6714 thus far. To enable the double-comparative transcriptomics approach, we used existing information for strain 6803^19^ and generated a matching dataset for strain 6714 employing the same growth conditions, library preparation protocols and computational methods. Total RNA was isolated from cells cultured under multiple growth conditions (darkness, high light (HL), cold (15°C) and heat stress (42°C), depletion of iron (-Fe), phosphate (-P), nitrogen (-N) or inorganic carbon (-C), and exponential and stationary growth phases), analysed according to the dRNA-seq protocol^7^ and used to infer transcriptional units (TUs)^27^. Following the previously introduced terminology^19^, we termed a transcriptional unit gTU if it covers one or more annotated genes, aTU if it is antisense to another TU (overlap ≥20 nt) and nTU if it is free-standing. The classification of a TU can be ambiguous as is the case for excludons^28^, which cover annotated genes and regions antisense to another gene or TU. Therefore, we provided all possible notations when a TU belonged to several categories. Each TU has a corresponding TSS whose associated read count in the treated library defines the expression level of that TU. The full list of TUs is presented in Table S1 and a genome-wide visualisation is presented in Supporting files S1-4.

We defined 4,292 TUs in *Synechocystis* 6714 compared to 4,091 TUs in *Synechocystis* 6803^19^. Table 1 summarises the numbers in the different transcript categories for each strain. We found 2,373 (83%) of the 2,854 protein-coding orthologous genes^26^ transcribed from a gTSS in both strains under at least one of the examined conditions, yielding 2,012 and 1,924 gTUs for *Synechocystis* 6803 and *Synechocystis* 6714, respectively. From these, 850 gTUs (>42%) are perfectly conserved with regard to the covered genes and their arrangement. Strain-specific gTSSs for genes without any TSS in the other strain existed for 159 and 202 orthologs in *Synechocystis* 6803 or 6714, respectively.

For each of the 10 tested conditions, the TU associated with the highest read number, among all TUs that are maximally expressed under the respective condition, is given in Table S2. In three conditions, the top ranking TU gave rise to an abundant sRNA (Table S2). The sRNA Ncr0700 (TU3047), with 11,257,987 normalised reads, is the most abundant transcript in strain 6714 and is maximally expressed in darkness, as is its ortholog in strain 6803^19^. Altogether, in four conditions (-P, darkness, 42°C and -N) we found orthologous transcripts to be associated with the highest read numbers in both strains (Table S2). For two TUs (-C and cold), the ortholog in *Synechocystis* 6803 either showed highest read levels under a different growth condition or was outperformed by another TU with higher read numbers. For the TUs top ranking in stat. phase and HL, ortholog TUs in 6803 are simply lacking, while for the remaining (-Fe and exp. phase), interesting regulatory differences were detected (see below).

In *Synechocystis* 6714, the longest TU (TU2897, Table S1) encompasses 18 genes encoding ribosomal proteins (corresponding to the enterobacterial S10 and *spc* operons, except for the *rps10* gene). The longest TU in *Synechocystis* 6803 (22 genes) contains mainly genes without an ortholog in 6714 (or in any other cyanobacteria) and has features of a genomic island^19^,^26^. This genomic island exists in both strains. In both cases the genes encode glycosyltransferases and glycoside hydrolases possibly involved in the modification of cell surface properties. However, these genes are of entirely different phylogenetic origin in the two strains.

The median 5′ UTR length for *Synechocystis* 6714 is 54 nt, similar to the 52 nt in *Synechocystis* 6803 (Supplementary Figure S1), and the median 3′ UTR length is, with 128 nt, also only slightly longer than the 118 nt found in strain 6803. Our data provide evidence for 48 leaderless mRNAs in *Synechocystis* 6714. Interestingly, their expression was distributed over all conditions, and only 6 of them are conserved in *Synechocystis* 6803 (Table S3). Leaderless mRNAs play a role in stress adaptation in *E. coli*^29^ and are generally thought to be restricted to a particular condition, which we did not observe in our data.

**Figure 1:**
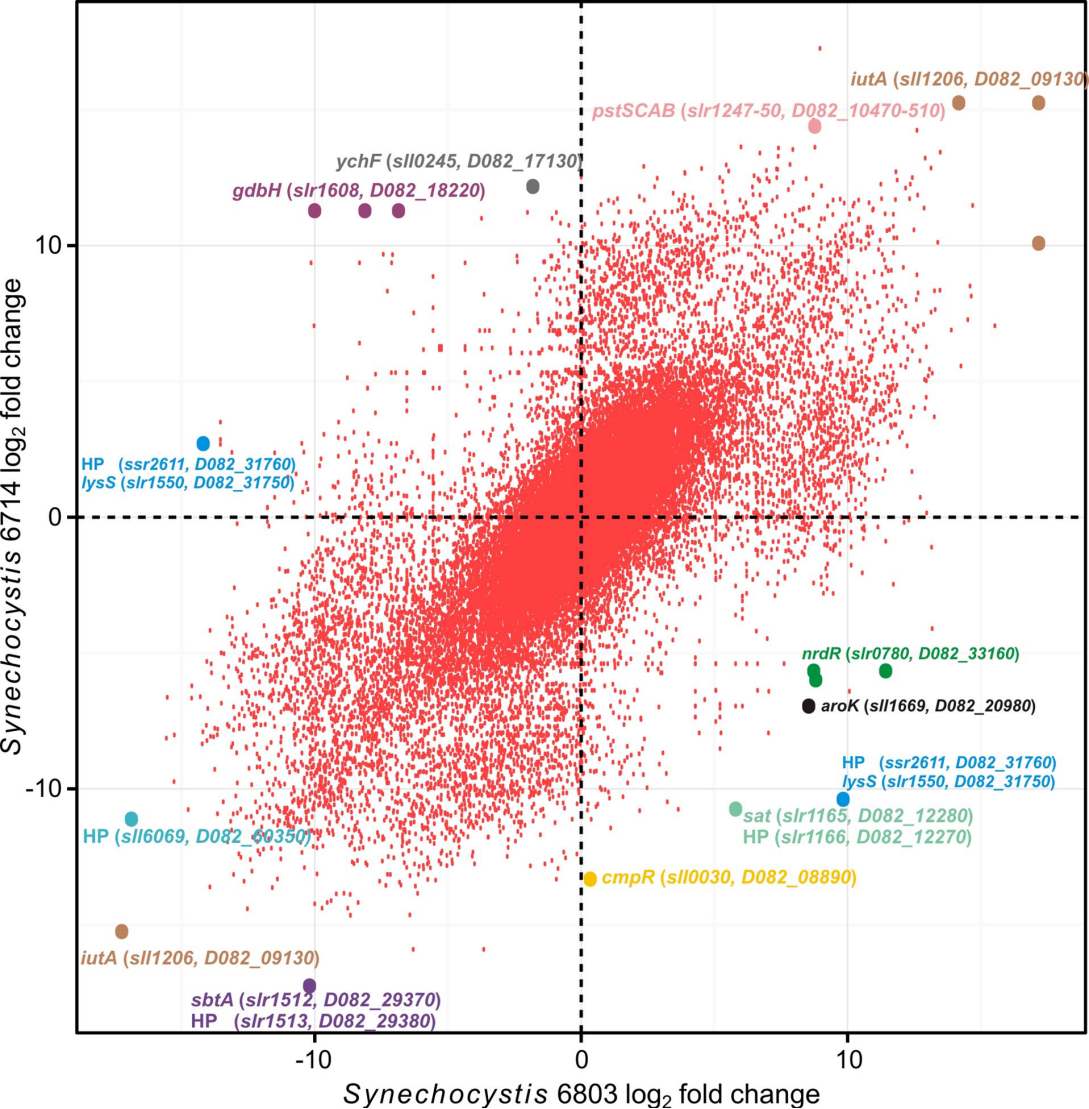
Regulation and conservation. Scatter plot of all pairwise log_2_ fold changes for the 859 gTUs orthologous between *Synechocystis* 6803 (x-axis) and 6714 (y-axis). The coordinates of each point resemble the pairwise log_2_ fold change between a pair of conditions in one strain compared with the corresponding pair of conditions in the other. Some extreme examples are annotated. For *iutA*, encoding the TonB-dependent ferric siderophore receptor the regulation is conserved with maximum expression in -Fe and no expression in any of the other conditions. For *gdbH*, the maximum differences in fold changes between *Synechocystis* 6803 and 6714 appeared for 15°C/42°C, 15°C/stat. phase and 15°C/-C. For *cmpR*, the maximum difference in fold changes was detected for -C/HL. See Figure 2 and Table S1 for details about the expression patterns and the annotation of the mentioned genes; HP, hypothetical protein.

**Figure 2:**
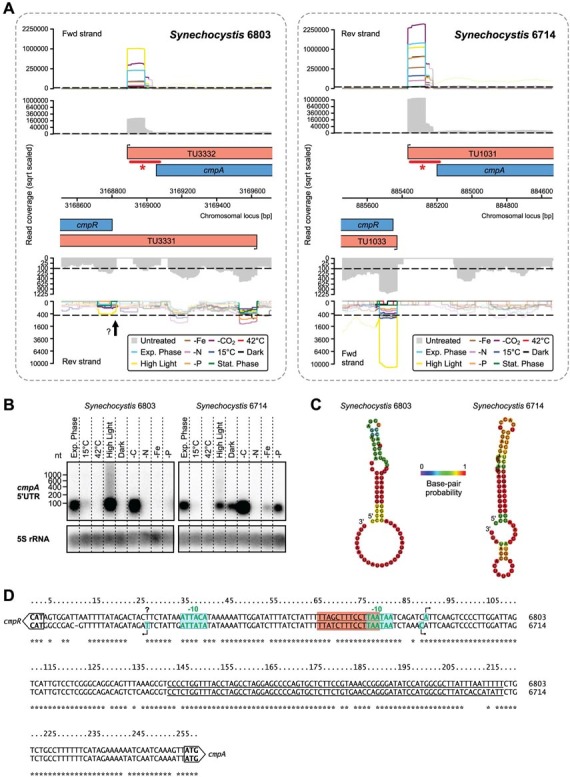
Differences in the expression of CmpR, a LysR family transcription factor involved in the control of carbon uptake and concentration. (A) Transcriptional organization in the intergenic region between *cmpA* and *cmpR*. The color-coded graphs represent the accumulation of primary reads in the dRNA-seq analysis for the ten compared conditions. From the TSS identification and secondary read coverage (grey), transcriptional units (TUs, red) were inferred. Protein-coding genes are displayed in blue. A gTSS upstream of *cmpR* in strain 6714, but below the detection limit in strain 6803, is indicated by an arrow. Positions of ^32^P-labelled probes for Northern verification in panel (B) are marked by asterisked bars. The dashed lines mark the thresholds for the required minimum sequence coverage. (B) Northern verification for *cmpA* that shows accumulation of a distinct, small transcript from the 5′UTR. The signal for the 5S rRNA was used as a loading control. (C) Potential RNA terminator hairpins upstream of *cmpA* between −39 to −114 (+1 = first nucleotide of the *cmpA* start codon). (D) The *cmpA/cmpR* intergenic region harbouring the promoters for both genes. The underlined sequence was used for the RNA structure prediction in panel (C). The red box indicates putative *cmpR* binding sites consistent with the consensus motif TTA-N_7/8_-TAA described for *Synechococcus* sp. PCC 7942^30^. The entire region is almost identical in both strains, except a small number of single nucleotide exchanges, one of which is in the −10 element (boxed + green letters) and probably linked to the observed weaker *cmpR* promoter activity in *Synechocystis* 6803.

To address the question of whether orthologous transcripts have globally similar expression patterns, we performed a combined clustering of all orthologous TUs based on their expression profiles. Interestingly, only about one third of the orthologs were grouped in the same cluster for a relatively low number of clusters (k-means; k = 10), and this value decreased with increasing k (Figure S2). This shows that the majority of the orthologous transcripts differ substantially in their expression pattern. While the expression may differ in one aspect, regulation as a whole may still be conserved to a large degree. Thus, in a second approach, we compared the FCs of ortholog TUs for all pairs of growth conditions. The resulting scatter plot indicates an overall medium correlation (R^2^ = 0.70) between the FCs of the two strains (Figure 1). Remarkable regulatory differences include the *cmpR* gTSS, which strongly responds to HL in strain 6714 but is barely active in strain 6803 in this condition (Figure 2). The *cmpR* gene encodes a LysR-type transcriptional activator for a part of the carbon concentrating mechanism^30^. Its divergent regulation may affect the expression of its main target, the *cmpABCD* operon encoding a transporter for bicarbonate uptake (Figure 2A, B), and can be linked to specific differences in its promoter organisation, in particular to a single nucleotide exchange in the −10 element (TATAAT → TGTAAT) that renders it less active in strain 6803 (Figure 2D). Another observation concerns the 5′UTR of *cmpA* that accumulates as a very abundant separate short transcript in both strains, most likely caused by premature termination at Rho-independent terminators (Figure 2B, C).

Differential expression is commonly regarded as evidence of the functional significance of a gene because it requires the existence of a regulator and a specific regulatory element. We assessed the four different TU types for their propensity for differential expression by computing for each TU the maximum FC in every pair of conditions. Figure S3A shows that the majority of TUs of all types differ by more than 10-fold in expression in at least one pair of conditions. It is noteworthy that gTUs more frequently show high FCs compared to aTUs and iTUs. Similarly, gTUs also show a tendency for higher expression levels (Figure S3B).

### Responses to environmental stimuli are largely conserved but also reveal specific regulatory differences

**Figure 3:**
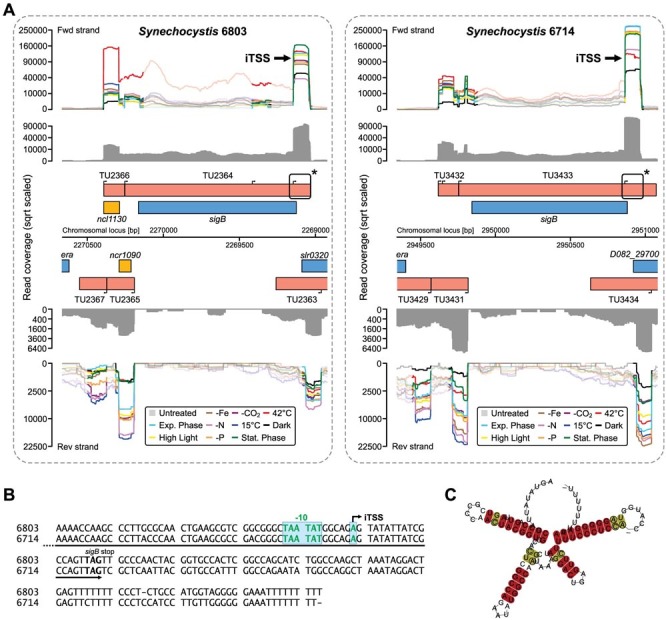
Conserved complex TSS arrangements. (A) The *sigB* gene and loci near to it illustrate the complexity and conservation of TSS arrangements. In accordance with previous findings, the *sigB* gene in *Synechocystis* 6803 is induced by a short treatment at 42°C^31^,^32^. This regulation is also found in strain 6714. In both strains, the *sigB* TSS is located antisense to a putative sRNA, qualifying the *sigB* mRNA as a gaTU. In addition there are two iTSS, one located within the *sigB* gaTU towards the end of the *sigB* reading frame and the other one located at the end of the downstream, tail-to-tail oriented genes *slr0320*/*D082_29700*, which encode an Fe-S oxidoreductase. Also these iTSSs appear to be environmentally regulated, suggesting functional relevance. For annotation details see Figure 2A. (B) Details for the location of the rear iTSS in *sigB*. (C) The consensus structure of the transcript originating at the *sigB* iTSS supports its classification as an sRNA based on length, lack of a reading frame and high support from the program RNAz^58^ with an SVM RNA class probability of 0.99.

The *sigB* (*sll0306*) gene, encoding the RNA polymerase σ factor SigB, illustrates the complexity and conservation of transcriptional signals. There are at least four TSSs associated with this gene (Figure 3). In *Synechocystis* 6803, *sigB* is induced by a short treatment at 42°C^31^,^32^ and possesses a central role in the survival of the cells during short heat stress conditions^32^,^33^. Consistent with these facts, we identified a gTSS that is strongly induced upon heat stress. This gTSS is conserved in *Synechocystis* 6714 and, similar to strain 6803, shows maximum expression under heat stress. Furthermore, in both strains, the *sigB* gTSS is located antisense to a putative sRNA (TU3431 in 6714), causing a 197 nt (200 nt in 6803) long overlap within the 5′UTR of *sigB* and qualifying the *sigB* mRNA as a gaTU. Interestingly, the TSS driving transcription of the anTU antisense to the *sigB* 5′UTR is under environmental control. In both strains, it is high in the cold and –N condition but relatively weak during heat stress (Figure 3A). The most bizarre findings are two iTSSs, one located towards the end of the *sigB* reading frame and the other located at the end of the downstream, tail-to-tail oriented gene. Additionally, these iTSSs appear to be environmentally regulated, suggesting functional relevance. The very strong iTSS at the end of the *sigB* reading frame is regulated different from the *sigB* host gene, supporting it further as a separate transcriptional entity. The transcript originating from this iTSS was identified in *Synechocystis* 6803 independently before, characterising it as a ~100 nt sRNA on the basis of Northern and tiling microarray analyses^34^. This observation is consistent with the prediction of a short sRNA originating from a conserved promoter and finishing with a pronounced Rho-independent terminator of transcription (Figure 3B,C). Consequently, it is very interesting to note that not only the complexity of this arrangement within and around the *sigB* gene has been conserved, but also the modes of regulation.

The expression of stress-related genes may be restricted to a single condition in which a TSS shows maximum activity. We analysed TSS activity globally and comparatively by dividing the top number of normalised reads for a gene in one condition by the number of reads from the condition with the second highest number of reads (UEF, unique expression factor^19^). A high UEF (>5.0) indicates strong induction of the respective TSS under one particular condition. Many of the top-induced TSSs are consistent with the respective stress conditions, e.g., for the phycobilisome degradation protein NblA during -N or for the aforementioned transcriptional regulator CmpR under HL (Table S1). In the previous analysis of *Synechocystis* 6803, the phosphate and iron stress regulons were studied more closely. The *Synechocystis* 6803 regulon responding to phosphate depletion encompassed 8 TUs^19^, which comprise the genes reported before^35^ as well as newly identified ones, e.g., the phosphate-stress-induced PsiR1 transcript. Our results (Table S4) reveal a high level of conservation for this regulon, with the exception of PsiR1, which is missing in *Synechocystis* 6714 and two TUs that are not affected by phosphate depletion in strain 6714. Interestingly, we identified with D082_04580 in addition to D082_05330 a second cAMP-binding regulator of the CAP family of transcription factors that belongs into the P-regulon. Upon closer inspection, it turned out that also the two homologs in strain 6803, Slr0607 and Sll0594, are up-regulated upon phosphate depletion and that the former had just so missed the threshold in the previous analysis. These two regulatory proteins are quite different from each other. Their example illustrates how only by the comparison of the two datasets novel regulatory factors can be identified. Both proteins are widely distributed throughout the cyanobacterial phylum but their involvement in the response to phosphorus depletion was not known so far.

**Figure 4:**
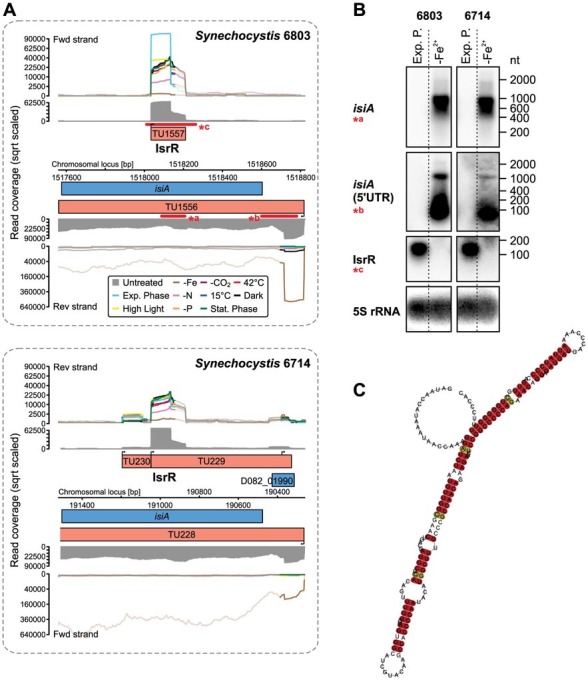
Two conserved sRNAs in the iron stress-response of *Synechocystis*. (A) Transcriptional organization and expression of the *isiA* locus in *Synechocystis* 6803 and 6714. For annotation details see Figure 2. In both strains, *isiA* is highly inducible in the -Fe condition (brown graph), while the asRNA IsrR is inversely regulated, suggesting a conserved function. (B) Verification experiment for the presence of IsrR and for the iron stress-dependent upregulation of the *isiA* mRNA and a separate sRNA from its 5′UTR. The 5S rRNA was used as a loading control. (C) Consensus structure of the IsrR homologs predicted by RNAz^58^.

Similar to the analysis of the phosphate stress regulon, we ranked the iron stress-specific TUs according to their maximum UEF values (Table S5). In *Synechocystis* 6803 18 TUs belong into the core iron stress regulon^19^, compared to 17 TUs in *Synechocystis* 6714. In both strains, these TUs include 32 protein-coding genes and one sRNA. The fact that the majority of genes responded in a similar fashion in both strains, including the well-characterised iron stress marker gene *isiA* (Figure 4), suggests a highly conserved iron stress response in the two strains. Differences include the two genes *ssr2333* and *slr1392*, which lack a homolog in *Synechocystis* 6714. These genes encode a FeoA/FeoB-type ferrous iron transporter that exists in only few cyanobacteria. On the other hand, there are two additional genes in *Synechocystis* 6714 that belong into the core iron stress regulon. These are D082_02330, without known function, and D082_21730 encoding a GT1_wbuB_like protein closely related to the GT1 family of glycosyltransferases that is a monocistron in strain 6714 (TU959), whereas the homologous gene in strain 6803, *slr1085*, is part of a multicistron not affected by -Fe.

In several bacteria, regulatory sRNAs have been characterised that play an important role in the iron stress response^36^,^37^,^38^. Indeed, we also detected the antisense RNA IsrR in *Synechocystis* 6714 (Figure 4A, B), which acts as a negative regulator of *isiA* encoding the iron stress inducible protein A in *Synechocystis* 6803^20^. In addition, the comparison of possible secondary structures shows 4 base transitions that are consistent with the structural model, suggesting selective force on the structure of IsrR. Moreover, we identified the sRNA IsaR1^19^ with an UEF of 130.9 to be the specifically and by far the most strongly up-regulated nTU under iron depletion (Table S5), supporting its functional relevance within the iron stress regulon.

### Diversity and conservation within the non-coding transcriptome

The number of orthologous genes that are associated with at least one antisense transcript (aTU) in both organisms is 995. However, the corresponding TSS was conserved for 168 aTUs only (Table S6). Many of them likely play an important regulatory role, because they are conserved in terms of sequence, position and sometimes even regulation. Indeed, we detected the antisense RNA IsrR in *Synechocystis* 6714 in this class (Figure 4). Other examples of conserved asRNAs were found for the ABC transporter subunit genes *sll0484/D082_06780* or the *ccmK* genes (*sll1028/D082_28020*). The latter encode the carbon dioxide-concentrating mechanism protein K homolog 2. Therefore, the maximum expression of these *ccmK*-aTUs under N-depletion points to a possible silencing function when nitrogen is limited (Supporting File S1).

**Figure 5:**
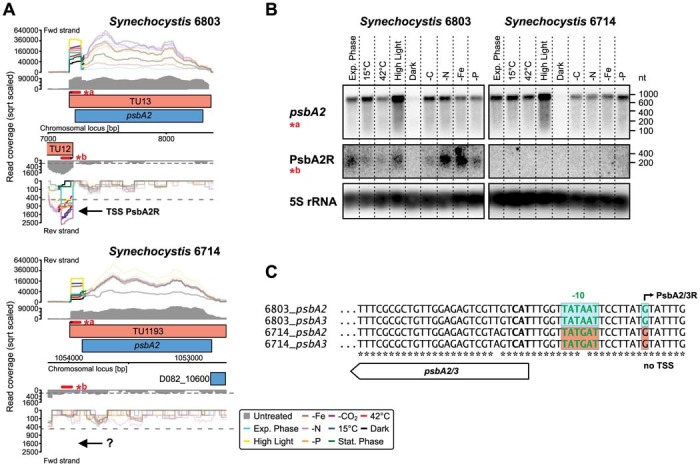
Example for a functional asRNA that is not conserved. (A) Transcriptional organization and expression of the *psbA2* locus in *Synechocystis* 6803 and 6714. The mRNAs of the very closely related genes *psbA2* and *3* are positively regulated by light in both strains, whereas the asRNAs PsbA2R and PsbA3R^22^ originating from a TSS 19 nt upstream of the start codon (TU12 and TU1885) are specific for *Synechocystis* 6803. (B) The absence of PsbA2R in *Synechocystis* 6714 was verified by Northern blots. The 5S rRNA was used as a loading control (the *psbA2* membrane is shown). (C) Alignment of the promoter sequences for PsbA2R and PsbA3R with the corresponding regions in *Synechocystis* 6714 indicate only two mismatches between the two strains. Of these, a single transition TATAAT → TATGAT within the −10 element (boxed) is likely involved in the activation/deactivation of the aTSS. For orientation, the *psbA* start codon on the reverse strand is shown in bold, and the direction of translation is indicated by the arrow bar.

In contrast, we found in *Synechocystis* 6714 no evidence for the conservation of asRNAs to the *psbA* genes, which sustain high levels of expression of *psbA2* and *psbA3* in *Synechocystis* 6803^22^. Despite the very high (97%) nucleotide sequence conservation between the *psbA* genes of both strains, corresponding asRNAs were not detectable in *Synechocystis* 6714 (Figure 5A, B). On a closer inspection, two nucleotide polymorphisms were identified, from which a G-to-A transition within the −10 element of the TSS of the asRNA PsbA2R is a likely gain-of-function mutation in *Synechocystis* 6803 (Figure 5C). Thus, a single mutation led to the activation of this antisense promoter in strain 6803, or alternatively, disrupted it in strain 6714. Our verification experiments show that in addition to the known co-induction of *psbA2* and *psbA2R* in *Synechocystis* 6803, the asRNA is expressed more highly under conditions that lead to oxidative stress (Figure 5B), possibly extending the known functional role of *psbA2R*^22^.

Trans-encoded sRNAs are by far the most promising class of non-protein regulators. Therefore, we checked conservation and regulation of these transcripts revealing 69 intergenic transcripts (nTUs) that are conserved and predicted as nTU in both strains. Additionally, 48 nTUs from *Synechocystis* 6714 and 91 nTUs from *Synechocystis* 6803 were conserved but classified differently in the other strain (Table S7). Furthermore, in both strains, 12 known sRNAs were part of gTUs or aTUs, e.g., caused by a downstream located and co-transcribed gene lacking a gTSS. When these known sRNAs are included, the number of conserved sRNAs and sRNA candidates increases to 221. We found both, the sequences (87.8% average identity) as well as the expression of many of these sRNAs conserved, pointing to their potential functional relevance. This is illustrated by the above mentioned sRNAs IsaR1 and Ncr0700, which are maximally expressed during iron depletion or in the dark, as well as PsrR1 (Photosynthesis regulatory RNA 1), which is up-regulated under high-light treatment or CO_2_ depletion^19^,^34^ and controls the expression of several genes encoding photosynthetic proteins^23^, and by the sRNA NsiR4, which is strongly induced upon nitrogen depletion (Table S1 and Figure S4).

The same type of analysis revealed conservation of 147 iTSSs in 141 out of 360 iTSS-containing ortholog pairs (Table S8). Some of these conserved iTSSs appear very strong and are in genes of known relevance. Among them are the iTUs originating within the *sigB* gene (Figure 3), in the gene *rre39* (*slr1588/D082_10840*, regulatory component of sensory transduction system Ssp2/Rre39), and in the *ntcA* gene, which encodes the major regulator of nitrogen metabolism.

### Actuatons: sRNA cassettes driving gene expression

We noticed that some known sRNAs were classified as gTUs because a downstream located gene in sense orientation lacks a specific gTSS and is apparently co-transcribed with the sRNA due to incomplete termination of transcription (Table 2). An example of this arrangement is constituted by the sRNAs Yfr2b and Yfr2c. These belong to an sRNA family of unknown function that is widely distributed among cyanobacteria, the genes of which occur in different genetic arrangements and in copy numbers from one to nine^39^. There are three members of the Yfr2 family in *Synechocystis* 6803, which accumulate as sRNAs of 80, 65 and 70 nt^40^. All three are conserved in strain 6714 and present in the same genomic context, with *yfr2a* as a free-standing gene, and *yfr2b/c* linked to the respective orthologous protein-coding genes in the two strains (Table 2).

We noticed at least 10 cases in which very abundant sRNAs became part of such a chimeric precursor transcript and therefore give rise to the respective mRNAs, which otherwise have no distinct TSS (Table 2). Such an sRNA/mRNA structure, which we named ‘actuaton’, constitutes a means of remodeling the transcriptional network. This concept is strongly supported by TSS/sRNA cassettes that are fused to different genes or are specific for one of the investigated strains and hence provide a different expression pattern. Examples include the Ncr0700 sRNA that originates from a free-standing TU in *Synechocystis* 6714, whereas it became part of a chimeric TU in strain 6803 due to rearrangement by transposition.

**Figure 6:**
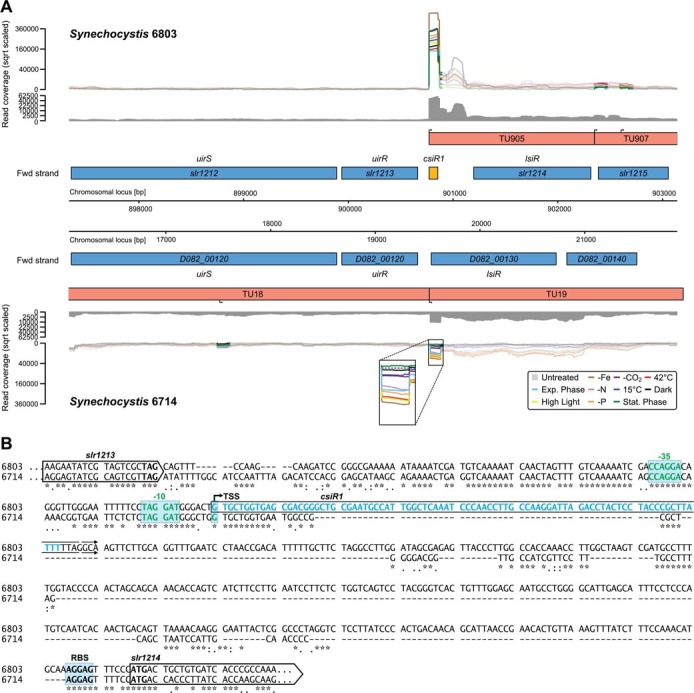
Actuatons in bacterial gene expression. The *uirS-lsiR* region encompassing a cyanobacteriochrome (*slr1212/uirS* or *pixA*) and two response regulator genes (*slr1213/uirR* or *nixB* and the PatA-type regulator *slr1214/lsiR* or *nixC*) encodes a UV-A-activated signaling system^41^,^42^. This genomic region is very similar in both strains with the exception of a proximal TSS upstream of *D082_00130* and a more distally located TSS upstream of its ortholog *slr1214*. The latter gives rise to the sRNA CsiR1, from which read-through occurs into *slr1214*. (B) Sequence alignment of the intergenic region between *slr1213*/D082_00120 and *slr1214*/D082_00130.

Evolutionary events such as gain or loss of an sRNA within or close to a promoter may directly affect other genes. The sRNA CsiR1^19^ illustrates this effect (Figure 6). CsiR1 originates from the *uirS-lsiR* region in *Synechocystis* 6803 but is not present in strain 6714. However, the *uirS-lsiR* region encompassing a cyanobacteriochrome (*slr1212/uirS* or *pixA*) and two response regulator genes (*slr1213/uirR* or *nixB* and the PatA-type regulator *slr1214/lsiR* or *nixC*), which encode a UV-A-activated signaling system that is required for negative phototaxis in *Synechocystis* 6803^41^,^42^, is, except *csiR1*, fully syntenic between the two strains (Figure 6A). As we show here, the previously reported expression and accumulation of CsiR1 in *Synechocystis* 6803^19^ results from integration of a sequence downstream of an otherwise conserved promoter element (Figure 6B). In *Synechocystis* 6714, the intergenic region between *uiR* and *lsiR* measures only 205 bp, while it spans 535 bp in *Synechocystis* 6803. Although the sequence was integrated downstream of the -10 element, it has a clear influence on the general expression level, which is lower in strain 6714. Moreover, whereas the expression of that TSS seems rather constitutive under these tested conditions in strain 6803, it appears very low or even absent in darkness and in stationary phase cells of strain 6714 (Figure 6A, Supporting File S1).

## Discussion

With ten different conditions analysed in two closely related cyanobacteria, this work presents a complex comparative study of microbial primary transcriptomes at single-nucleotide resolution. We consider the selected conditions to include some of the most relevant environmental factors for a photosynthetic organism. In particular, high light, darkness, the bioavailable iron and the supply of inorganic carbon are important determinants for the performance of oxygenic photosynthesis and the photosynthetic electron chain. The other chosen conditions, cold and heat stress, the depletion of phosphate or nitrogen and the effects of differential growth phases are also highly relevant but represent factors of general relevance. For these reasons, our data are useful to understand the lifestyle of these photosynthetic microbes and their behaviour in nature.

From the conserved protein-coding genes between *Synechocystis* 6803 and 6714, we found 83% to be expressed in both, but for only 45%, the transcriptional organisation was also conserved. In line with previous findings from Listeria^11^ and enterobacteria^43^, 53% of sRNAs and sRNA candidates, but only 12% of the iTSSs and 4% of the aTSSs were conserved between the two *Synechocystis* strains. The lower degree of conservation of asRNAs raises the question of whether they result from pervasive transcription, the functional relevance of which is the scope of a lively debate^43^,^44^. Lack of conservation is often seen as evidence for a lack of function, used e.g., for bacterial antisense transcripts in a comparison between two different enterobacteria^43^. If this argument is turned around, our identification of 168 highly conserved *cis*-asRNAs with regard to sequence, position and regulation, likely identified important regulatory players. Indeed, one of them is the functionally well-characterised asRNA IsrR^20^.

However, in addition to IsrR there are three more asRNAs with known regulatory function in *Synechocystis* 6803, As1_flv4^21^, PsbA2R and PsbA3R^22^, none of which were found in *Synechocystis* 6714. For As2_flv4, the respective operon is simply lacking^26^. However, *psbA* genes are present in both strains and here we identified two base mutations, one of which is within the −10 element as the likely molecular basis for the presence or absence of PsbA2R and PsbA3R asRNAs (Figure 5B, C). Hence, a single point mutation leading to the loss or gain of a functionally relevant asRNA may provide a small but significant physiological advantage that becomes rapidly fixed during only a few generations, as was experimentally demonstrated for the *psbA2*/PsbA2R sense-antisense pair in *Synechocystis* 6803^22^. This is even more interesting, as PsbA2R is present only in substoichiometric amounts, is co-regulated with its sense gene and has a very short half life^22^. The number of asRNAs with a characterised function is still very small compared to the total number of such transcripts. Nevertheless, the existing data indicate that it might be too simple to infer non-functionality of bacterial asRNAs from the lack of conservation.

Our data suggest that the majority of these TUs are subject to regulation because they show largely different expression levels under the conditions tested. These differences should mainly reflect changes in promoter activity and be less strongly affected by transcript stability or the presence of stable degradation products, as we focused here on primary transcripts. This finding is consistent with an earlier report in which, based on microarray datasets, *Synechocystis* 6803 cultures exposed to only three different conditions (high light, CO_2_ depletion, or darkness) were interrogated with respect to the percentage of transcripts with significant regulation, which was 46.4% for putative trans-acting sRNAs, similar as for mRNAs (43%)^8^.

An exciting feature of the transcriptomic adaption identified here are the genetic elements that we named actuatons. A hallmark of these elements is that they are followed by a protein-coding gene in sense direction that lacks its own gTSS. Nevertheless, the dominating RNA species is an sRNA that accumulates as an abundant and discrete transcript and therefore constitutes a clearly separate entity. Therefore, an actuaton gives rise to an sRNA and at the same time constitutes part of the 5′ region of a gene. We found evidence for at least ten such events and show that insertion of an actuaton modifies the expression of the downstream gene. Striking examples include the CsiR1 element (Figure 6), the Yfr2b and Yfr2c sRNA genes, which belong to an sRNA family that is widely distributed among cyanobacteria^39^, and the sRNA SyR9 forming a variable part of the mRNA encoding aldehyde deformylating oxygenase^45^, the key enzyme for cyanobacterial alkane biosynthesis. Even the long 5′ UTRs of TUs encompassing otherwise well-characterised genes, such as those encoding RNase E and RNase HII (Table 2), the CmpABCD bicarbonate transporter (Figure 2B) or IsiA (Figure 4B), belong to this class. The change in expression of the mRNA part of an actuaton may result only from the sRNA promoter replacing the original one. In more complex scenarios, it may also result from events causing differential termination, for instance from the attenuation of transcription, as can happen with some riboswitches. Actuatons may also be predecessors or derivatives of riboswitches in which the sRNA function is modified to serve as the metabolite-sensing entity regulating the expression of the protein-coding part. One particular case of an actuaton is represented by *ncr1265* (Table 2). This ncRNA exists in 11 copies in strain 6803, whereas there is only a single free-standing TU of this type in *Synechocystis* 6714, on plasmid pSYLA. Ncr1265 originates in all cases from an ISY523-type transposase gene on the reverse strand close to the start codon, covering it and the ribosome binding site as an asRNA. With these features, Ncr1265 appears as a silencing RNA for the transposase, analogous to the RNA-OUT transcripts in enteric bacteria^46^. In the arrangement with *lpxB* in strain 6803, the original promoter was disrupted by the insertion of the transposase-ncr1265 cassette and there is no other TSS associated with *lpxB*, hence the mRNA is generated by read-through from *ncr1265*.

These findings are in line with the increasing understanding that functional RNA elements possess a certain plasticity^14^. Other examples include riboswitches in *L. monocytogenes*, that also act as trans-acting sRNAs with a regulatory function^47^ or those that control the 5′UTR of one gene by transcriptional termination in one condition as well as terminating the upstream gene in another condition^12^. In addition to regulation, non-coding RNAs in bacteria fulfill more global functions. A process, discovered in *Staphylococcus aureus*, and possibly evolutionarily conserved in Gram-positive bacteria uses pervasive antisense transcription to process sense transcripts for up to 75% of all annotated genes by creating double-stranded substrates^13^. The latter mechanism was suggested to act as a posttranscriptional mechanism to adjust mRNA levels in a genome-wide fashion^48^. Thus, the transcription of non-coding RNA in bacteria is pervasive^6^,^7^,^8^,^9^,^10^,^11^,^12^,^13^, but not meaningless, because there are many examples that demonstrate that non-coding RNA in bacteria fulfils specific regulatory functions targeting individual genes^15^,^16^, or serves more global functions^48^. These facts resemble findings for the composition of the eukaryotic transcriptome and the prevalence and functionality of non-coding transcripts^49^,^50^. In eukaryotes, comparative analyses suggested the evolution of the non-coding share of the transcriptome at a more rapid pace than the protein-coding fraction and there are spectacular examples on how this has impacted organismic complexity, cellular differentiation and the capability for physiological adaptation^51^.

Based on the double-comparative analysis of the primary transcriptomes of two closely related cyanobacteria, we demonstrated not only commonalities but also large and distinct differences in their repertoires of non-coding RNAs and in the regulation of gene expression among them. Several of these differences are of known functional relevance. We conclude that rapid fluctuations in the composition of the non-coding share of the bacterial transcriptome play an underestimated role in bacterial evolution and that pervasive transcription serves specific purposes rather than constituting an accidental activity.

## Methods

### Biological material and growth conditions

*Synechocystis* 6714 was purchased from the Pasteur Culture Collection (PCC) in Paris, France. Liquid cultures were grown at 30°C in BG11 medium^52^ under continuous white light illumination of 50–80 μmol quanta m^−2^s^−1^ and a continuous stream of air to the desired growth phase (OD_750_ = 0.6–0.8). For the transcriptome analyses, cultures were initially grown under standard conditions and then transferred to ten different conditions: (1) cold stress, 15°C for 30 min; (2) heat stress, 42°C for 30 min; (3) C_i_ depletion, 150 mL of culture was washed 3 times with 100 mL of carbon-free BG11 and cultured for additional 20 h; (4) dark, no light for 12 h; (5) Fe^2+^ limitation, addition of iron-specific chelator desferrioxamine B (DFB) and cultivation for additional 24 h; (6) high light, 470 μmol q s^−1^ m^−2^ for 30 min; (7) N depletion, 150 mL of culture was washed 3 times with 100 mL of nitrogen-free BG11 and cultured for additional 12 h; (8) P depletion, cultures were washed 3 times with P-free BG11 and further grown for 12 h; (9) stationary phase, cells were grown until an OD_750_ of 4.1 was reached; (10) exponential phase, cells were harvested at an OD_750_ of 0.6.

### RNA extraction, cDNA synthesis and sequencing

*Synechocystis* 6714 cultures were harvested by rapid filtration (Pall Supor 800 Filter, 0.8 mm), the filter immediately immersed in 1 ml of PGTX solution^53^ and frozen in liquid nitrogen. Total RNA was extracted and analysed by gel electrophoresis and Northern blotting as described^54^. For each condition, total RNA from two independent cultures was pooled. For sequence analysis, cDNA libraries were constructed (vertis Biotechnologie AG, Germany) and sequenced on an Illumina HiSeq 2000 machine as previously described^9^. The dRNA-seq protocol^7^ distinguishes treated and untreated libraries. In both cases, total RNA was fragmented with ultrasound (four pulses of 30 s at 4°C). For the treated libraries, RNA with a 5′ monophosphate was degraded using Terminator™ 5′ phosphate-dependent exonuclease (TEX, Epicentre). The remaining RNA (mainly primary transcripts with 5′-PPP) was poly(A)-tailed using poly(A) polymerase. Then, 5′-PPP RNA was dephosphorylated using tobacco acid pyrophosphatase (TAP) and ligated to an RNA linker with reverse complementarity to the linker-specific primer Lsp1 (Table S9). First-strand cDNA synthesis was initiated using M-MLV reverse transcriptase and oligo(dT)-adapter primer OdT1 containing a library-specific barcode sequence (Table S9). The cDNA fragments were amplified by 11–12 PCR cycles using linker-specific primer Lsp1 (Table S9). The resulting cDNA samples were double-stranded with a length of ~150–700 bp. The cDNA was purified using the Agencourt AMPure XP kit (Beckman Coulter Genomics). For the untreated library, we pooled total RNA from samples representing all 10 growth conditions, depleted rRNA using the MICROBExpress kit (Ambion), fragmented the RNA and performed a treatment with T4 polynucleotide kinase. Otherwise, the libraries were processed as described for the treated libraries, except for the TEX treatment.

### Read mapping, data normalization and differential expression analysis

A total of 216,592,987 reads were obtained for *Synechocystis* 6714 from the ten different conditions and the untreated library. The data were deposited in the NCBI Short Read Archive under accession SRP032230. A total of 204,428,620 reads (29,555,678 non-ribosomal reads) were mapped to the genome with segemehl^55^ using the default parameters. Details about the mapping statistics of the individual libraries are provided in Table S10. The data were normalized in two steps. First, the treated libraries were scaled to library sizes of 100 million reads, and positions with ≥500 read starts and with a ratio >0.5 between read starts and coverage were defined as true primary positions. Second, library-specific correction factors based on the fraction of counts at true primary positions were applied. The counts for the untreated library were scaled to counts per 100 million. The fold change (FC) was computed as the ratio of the normalised reads. The FC between the condition with maximum number of reads and the condition with the second highest number of reads is defined as the unique expression factor (UEF)^19^. For statistical support of UEF values, a pairwise analysis of TSS raw counts from all combinations of conditions was performed with Analysis of Sequence Counts (ASC)^56^. A UEF was considered significant if |FC| ≥ 2 and p(|FC| ≥ 2) ≥ 0.95.

### Prediction of transcriptional units

Transcriptional units were detected by RNAseg^27^ as described^19^. We termed a transcriptional unit gTU when one or more annotated genes were covered, aTU when it was antisense to annotated genes or TUs (overlap ≥20 nt), iTU when the TU was located within an annotated gene and nTU when it was freestanding. In ambiguous cases we provided all possible classifications. Thus, a TU starting at an iTSS, covering an annotated gene and extending into an antisense region was termed gaiTU.

### Leaderless transcript detection

gTSSs that were mapped to start codons, either the A of AUG or the preceding 10 nucleotides, were subjected to a closer inspection. When N-terminally shorter homologs were found in other bacteria, the transcripts were re-classified as non-leaderless and the corresponding gene models corrected and submitted to Genbank (CP007542.1 - CP007545.1).

### Ortholog TSS prediction

Orthologs of protein-coding genes in *Synechocystis* 6803 were defined previously^26^. Evolutionarily conserved gTSSs needed to be present in both orthologs. For aTSSs and iTSSs, we required the locations within the orthologous genes to differ by at most 10 nt. For nTSSs, we could not make use of prior ortholog information, thus, we used BLASTN with an e-value cut-off of 1e-5 and query coverage of at least 50% to infer evolutionary conservation.

### Northern Blot analysis

Selected transcripts were verified by Northern hybridization using single-stranded radioactively labelled RNA probes. These probes were generated by *in vitro* transcription as described^57^, using templates amplified by PCR and oligonucleotides listed in Table S10. For the analysis, 3 μg of total RNA was separated on 1.5% agarose gels, transferred to Hybond-N nylon membranes by capillary blotting and cross-linked by UV-illumination. The membranes were hybridized with the labelled RNA probes as described^57^. The signals were visualized using a Personal Molecular Imager FX system with Quantity One software (Bio-Rad).

## Supplementary Material

Supplementary Information Supporting File 1

Supplementary Information Supporting File 2

Supplementary Information Supporting File 3

Supplementary Information Supporting File 4

Supplementary Information Supplementary Figures

Supplementary Information Supplementary Tables

## Figures and Tables

**Table 1:** Genome statistics and numbers of different types of TUs for *Synechocystis* 6803 and 6714. gTUs were assumed to be conserved when the complete TU arrangement including the covered CDSs was conserved. Alignment positions with regard to the encoded amino acid sequence were used for the determination of conserved aTUs and iTUs. Conserved nTUs were detected via BLASTN and known non-nTU sRNAs were included

	*Synechocystis* 6803^a^	*Synechocystis* 6714	Conservation
Genome [kb]	3957	3739	ANI: 86%; 16S: 99.4%
Plasmids	7	3	-
Protein genes	3683	3770	2854
Non-coding fraction	12.85%	12.47%	-

Transcriptome characteristics			
TUs total	4091	4292	1341
gTUs	2012	1924	850
aTUs	3040	3758	168
iTUs	483	424	147 (incl. iTSSs)
iTSSs	766	776		
nTUs	367	306	221 (incl. known sRNAs)	

^a^Numbers for *Synechocystis* 6803 are taken from the literature^19^. ANI = average nucleotide identity.

**Table 2:** Actuatons detected in *Synechocystis* 6803 and 6714

6803 TU	ncRNA	6803 First gene	6714 TU	6714 First gene	Accumulation of sRNA part shown in	Annotation and comments
TU1737	Ncl0820	*sll0815*	NA	NA	Kopf et al., 2014^19^	hypothetical protein; only flanking genes *Sll0814* and *Sll0816* but not *Ncl0820* and *Sll0815* are conserved.
TU3300	SyR5	*sll0737*	TU1772	*D082_15700*	Georg et al., 2009^34^	O-antigen polymerase and TPR domain; conserved arrangement
TU3575	Yfr2c	*sll1477*	TU341	*D082_02910*	Voß et al., 2009^40^	protease of the Abi (CAAX) family; conserved arrangement
TU2628-2629	SyR9	*sll0208*	TU610–611	*D082_05310*	Klähn et al., 2014^45^	aldehyde deformylating oxygenase; conserved arrangement in the two strains
TU2885	Yfr2b	*slr0199*	TU2652	*D082_23050*	Voß et al., 2009^40^	glutamine amidotransferase; conserved arrangement
TU643	Ncr0280	*slr1028*	NA	NA	Kopf et al., 2014^19^	integrin subunit alpha; *Ncr0280* and *Slr1028* unconserved in *Synechocystis* 6803
TU905	CsiR1	*slr1214*	TU19	*D082_00130*	Kopf et al., 2014^19^	two-component response regulator LsiR or NixC; flanking genes are conserved but not *csiR1*
TU3599	Ncr1680	*slr0753*	TU1242	*D082_10970*	Kopf et al., 2014^19^	probable transport protein; conserved arrangement
TU1715	Ncr0700	*ssr2227*	TU3047	none	Kopf et al., 2014^19^, this paper, Figure S4	transposase in *Synechocystis* 6803, free-standing gene in 6714; sequence of *Ncr0700* is 91% identical in a 195 nt long overlap
TU87	Ncr0020	*slr1129*	TU2357	*D082_20640*	Kopf et al., 2014^19^	ribonuclease E; conserved arrangement
TU3332	Ncr1575	*slr0040*	TU1031	*D082_08870*	Kopf et al., 2014^19^, this paper, Figure 2B	*cmpA*; conserved arrangement
TU2592	Ncr1265	*slr0015*	TU5109	NA	Kopf et al., 2014^19^	lipid A disaccharide synthase LpxB; *Ncr1265* exists in 11 copies in strain 6803, representing a likely RNA-OUT transcript from an ISY523-type transposase gene. It is a free standing TU on plasmid pSYLA in *Synechocystis* 6714.
TU1556	-	*sll0247*	TU228	*D082_02000*	Kopf et al., 2014^19^, Dühring et al., 2006^20^; this paper, Figure 4	* isiA* 5′UTR
