# Supplementary material for: Variations in the non-coding transcriptome as a driver of inter-strain divergence and physiological adaptation in bacteria
Source: Sci Rep. 2015 Apr 22;5:9560. doi: 10.1038/srep09560 (PMC5386190; doi:10.1038/srep09560)
Supplement: Supplementary Information — Supporting File 2 [file srep09560-s2.pdf]

Variations in the non-coding transcriptome as a driver of inter-strain divergence and physiological adaptation in bacteria

Matthias Kopf*, Stephan Klähn*, Ingeborg Scholz, Wolfgang R. Hess[§] and Björn Voß
Genetics and Experimental Bioinformatics, Faculty of Biology, University of Freiburg,
Schänzlestr. 1, 79104 Freiburg, Germany
[§]Corresponding author
*shared first authors

Supporting file S2 | Visualization of the dRNA-seq data for the plasmid pSYLA of *Synechocystis* sp. PCC 6714.

The colored graphs represent the coverage of reads from the treated libraries for ten sequenced conditions. The corresponding colors are given at the bottom of each page. The TSS positions are indicated by black arrows, e.g. at the beginning of each predicted transcriptional unit (TU, shown in red), and the read coverage graphs are highlighted for the following 100 bp. The coverage from the untreated library is shown grey. The y-axis of the graphs is square-root-scaled but the tick labels show the true normalized coverage values. Protein coding genes are shown in blue, tRNA in green and rRNA in dark grey. The data for the forward strand is shown above and data for the reverse strand below the x-axis. The x-axis shows the nucleotide position of the plasmid pSYLA in bp. This document is searchable by locus tags from *Synechocystis* sp. PCC 6714, gene names (if available), nucleotide position (every 2000 bp) and the *Synechocystis* sp. PCC 6803 locus tags of the 2854 orthologous protein-coding genes.

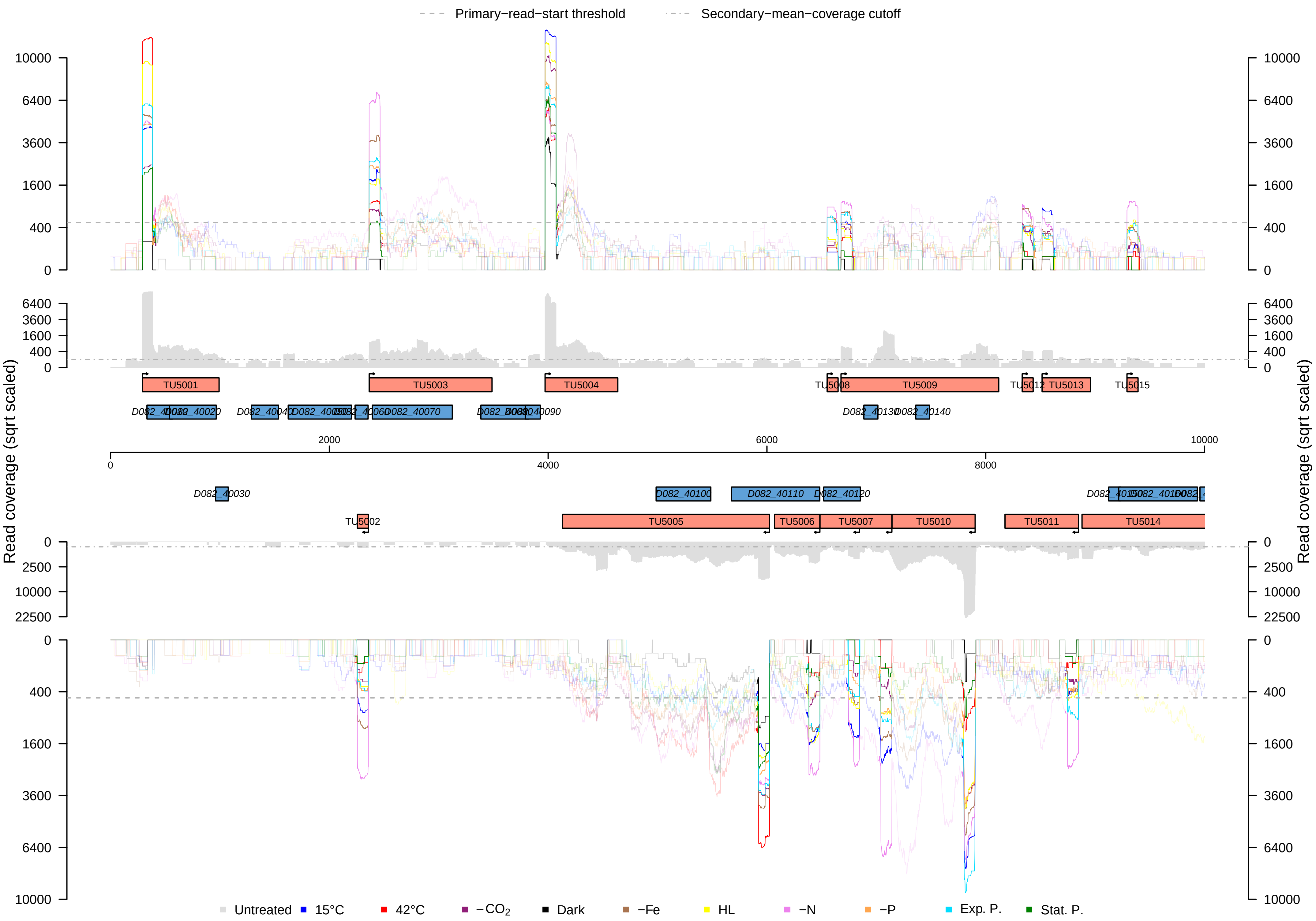

Primary−read−start threshold
Secondary−mean−coverage cutoff
Read coverage (sqrt scaled)
TU5001
TU5003
TU5004
TU5008
TU5009
TU5012
TU5013
TU5015
D082_40010
D082_40020
D082_40040
D082_40050
D082_40060
D082_40070
D082_40080
D082_40090
D082_40130
D082_40140
D082_40030
D082_40100
D082_40110
D082_40120
D082_40150
D082_40160
TU5002
TU5005
TU5006
TU5007
TU5010
TU5011
TU5014
Untreated
15°C
42°C
−CO2
Dark
−Fe
HL
−N
−P
Exp. P.
Stat. P.

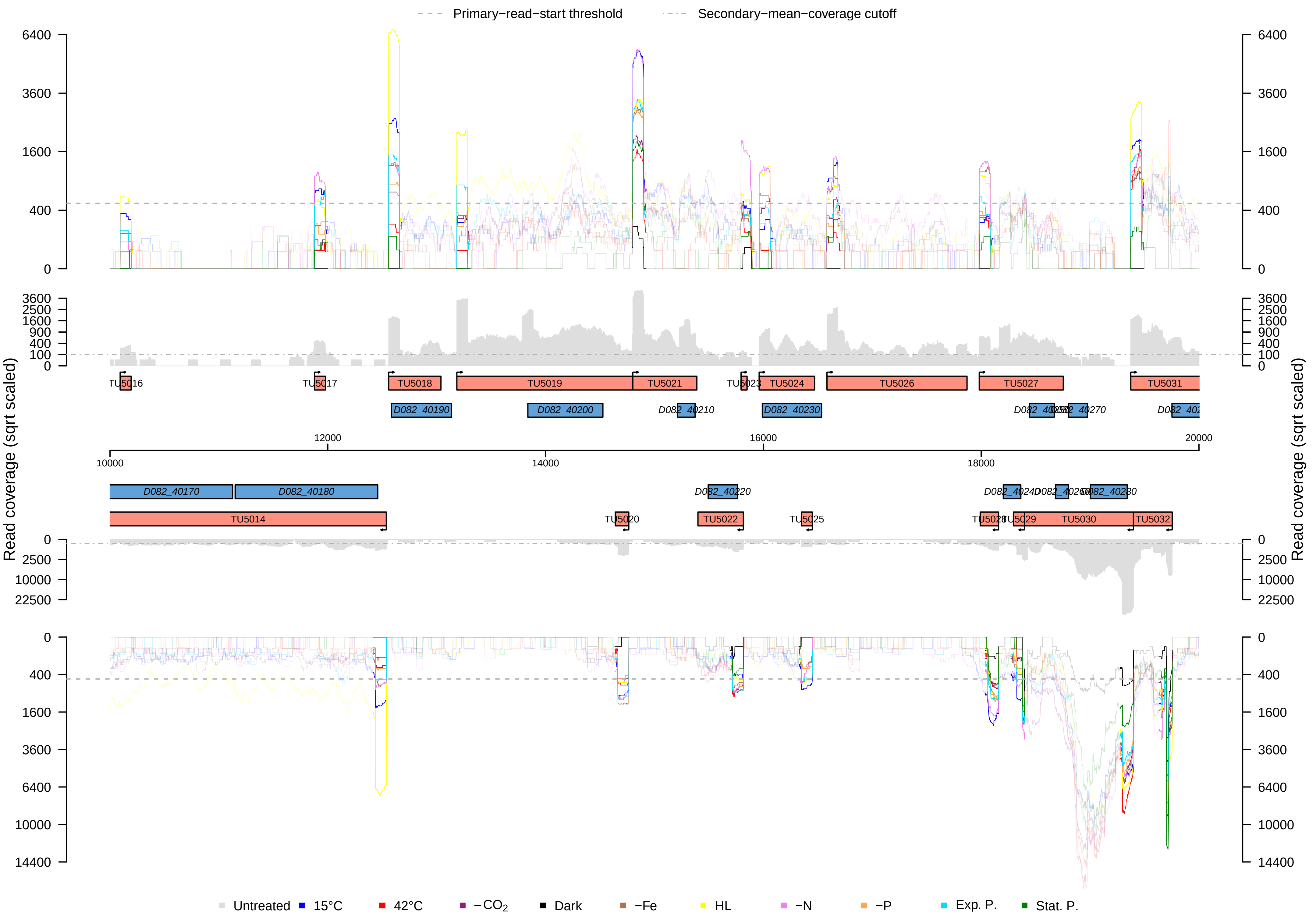

Primary−read−start threshold
Secondary−mean−coverage cutoff
Read coverage (sqrt scaled)
TU5016
TU5017
TU5018
TU5019
TU5021
TU5023
TU5024
TU5026
TU5027
TU5031
D082_40190
D082_40200
D082_40210
D082_40230
D082_40270
10000
12000
14000
16000
18000
20000
D082_40170
D082_40180
D082_40220
TU5014
TU5020
TU5022
TU5025
TU5029
TU5030
TU5032
Untreated
15°C
42°C
−CO2
Dark
−Fe
HL
−N
−P
Exp. P.
Stat. P.

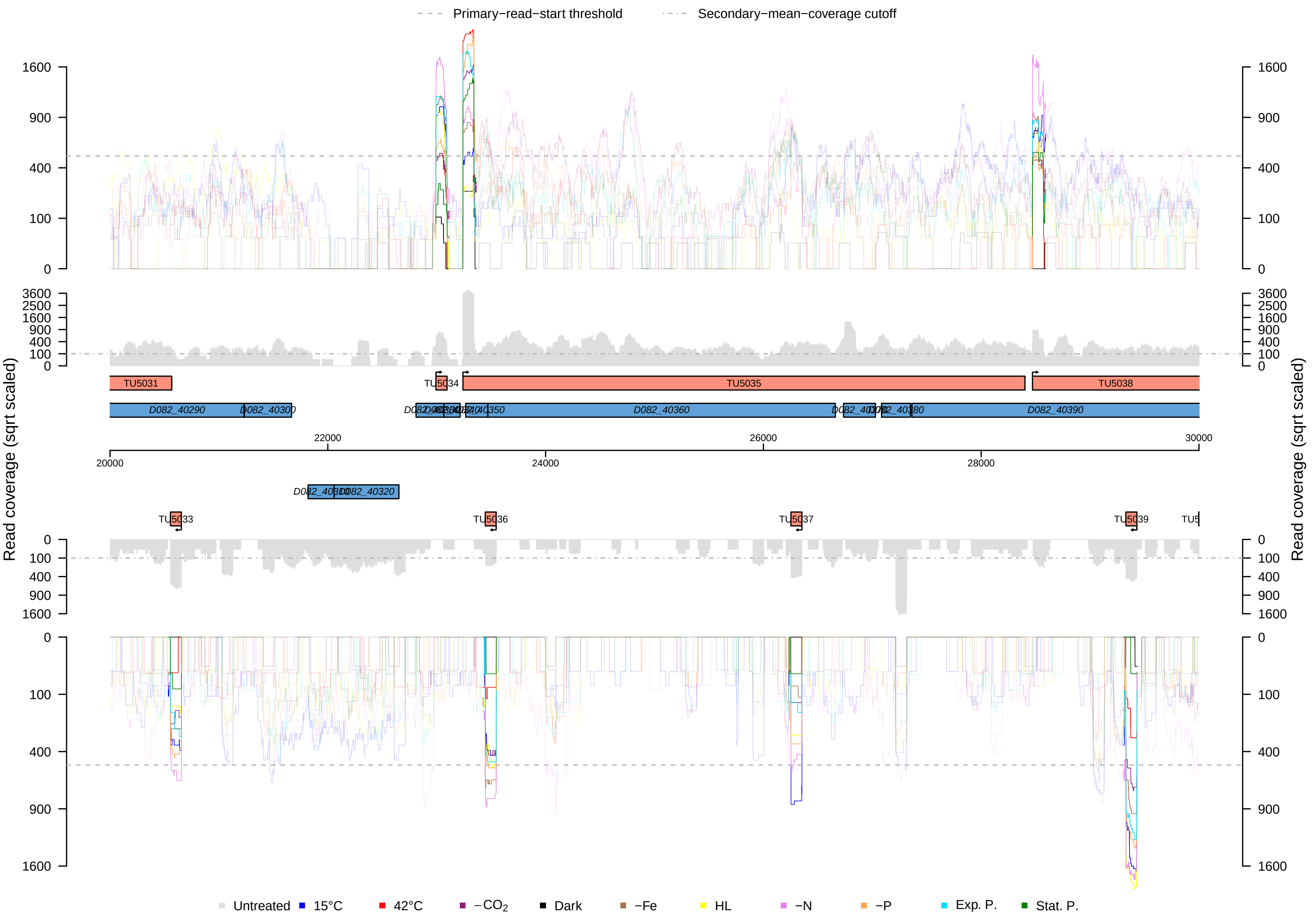

Primary−read−start threshold
Secondary−mean−coverage cutoff
Read coverage (sqrt scaled)
Read coverage (sqrt scaled)
1600
900
400
100
0
3600
2500
1600
900
400
100
0
TU5031
TU5034
TU5035
TU5038
D082_40290
D082_40300
D082_40350
D082_40360
D082_40380
D082_40390
D082_40310
D082_40320
20000
22000
24000
26000
28000
30000
TU5033
TU5036
TU5037
TU5039
TU5
Untreated
15°C
42°C
−CO2
Dark
−Fe
HL
−N
−P
Exp. P.
Stat. P.

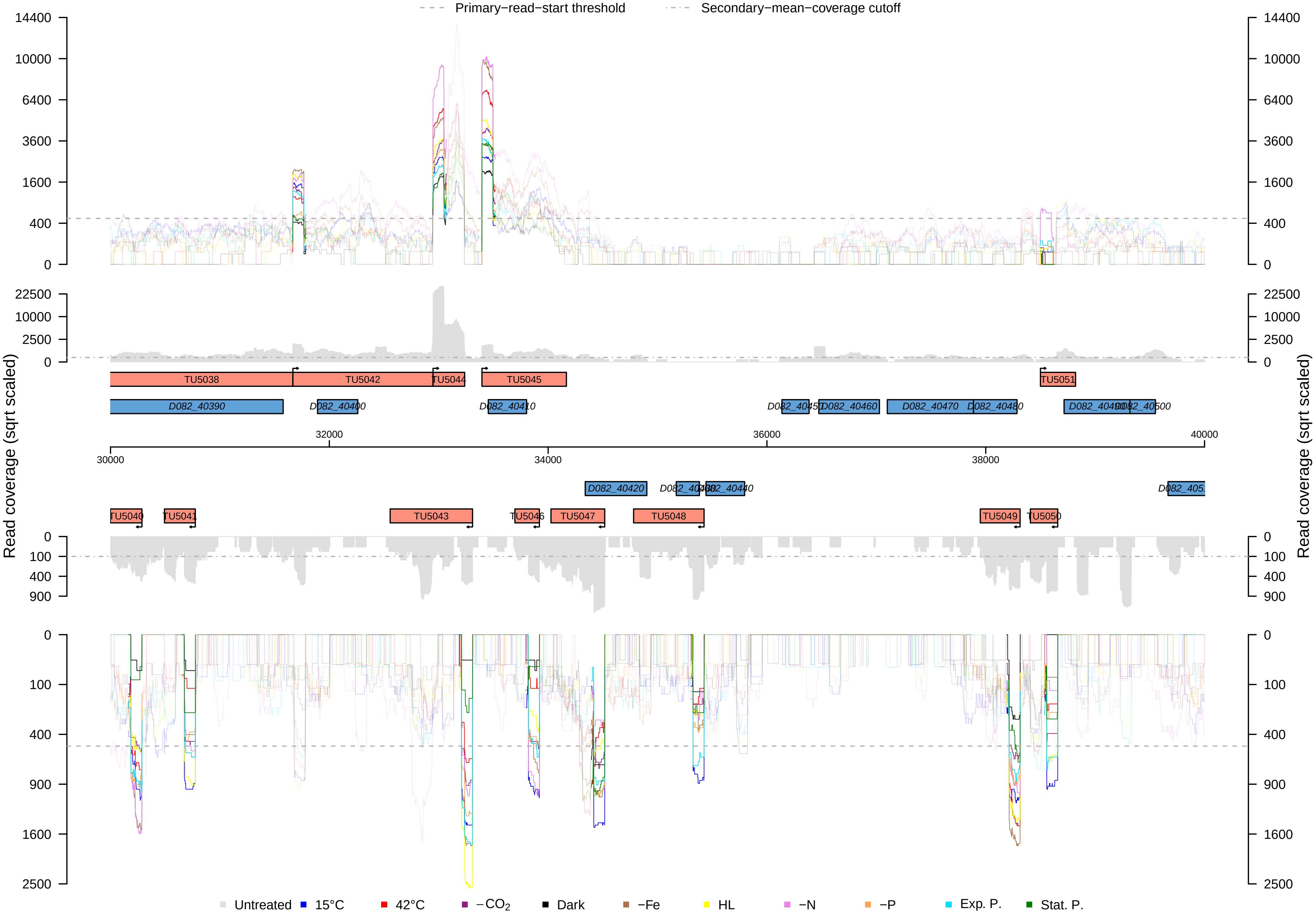

Primary−read−start threshold
Secondary−mean−coverage cutoff
Read coverage (sqrt scaled)
TU5038
TU5042
TU5044
TU5045
TU5051
D082_40390
D082_40400
D082_40410
D082_40450
D082_40460
D082_40470
D082_40480
D082_40490
D082_40500
D082_40420
D082_40430
D082_40440
TU5040
TU5041
TU5043
TU5046
TU5047
TU5048
TU5049
TU5050
30000
32000
34000
36000
38000
40000
Untreated
15°C
42°C
−CO2
Dark
−Fe
HL
−N
−P
Exp. P.
Stat. P.

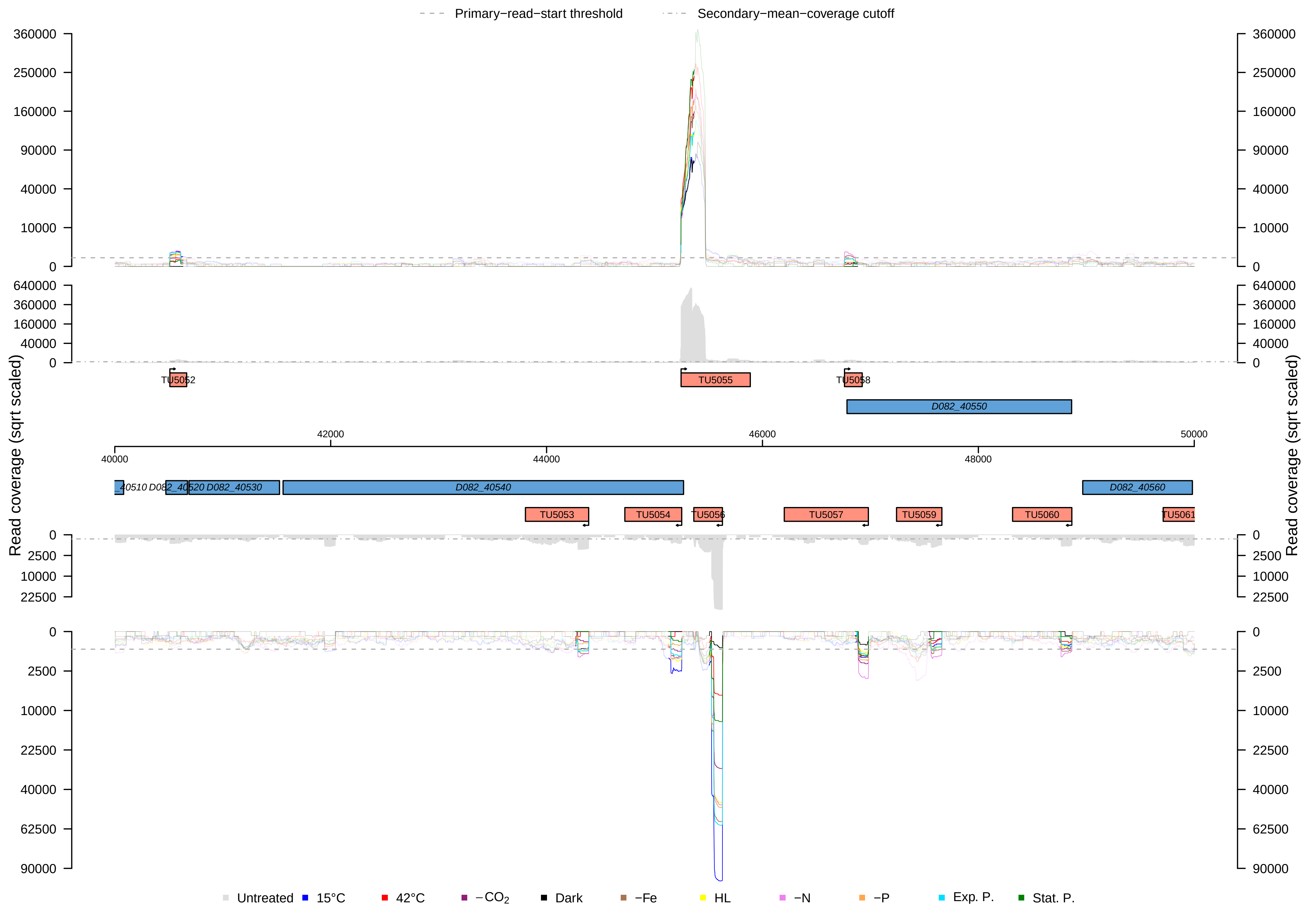

Primary−read−start threshold
Secondary−mean−coverage cutoff
Read coverage (sqrt scaled)
TU5052
TU5055
TU5058
D082_40550
D082_40510
D082_40520
D082_40530
D082_40540
D082_40560
TU5053
TU5054
TU5056
TU5057
TU5059
TU5060
TU5061
Untreated
15°C
42°C
−CO2
Dark
−Fe
HL
−N
−P
Exp. P.
Stat. P.

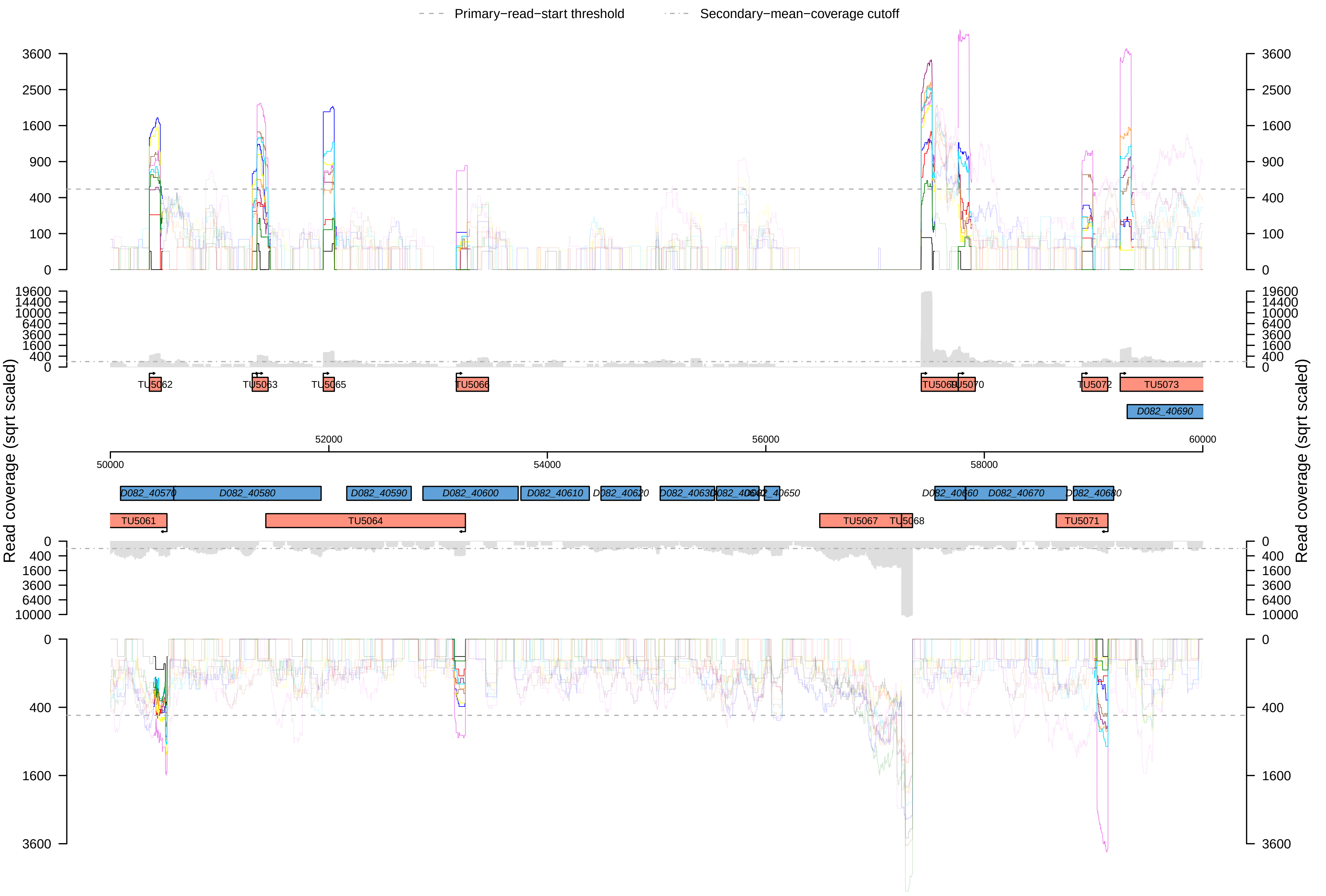

Primary−read−start threshold
Secondary−mean−coverage cutoff
Read coverage (sqrt scaled)
Read coverage (sqrt scaled)
TU5061
TU5062
TU5063
TU5064
TU5065
TU5066
TU5067
TU5068
TU5069
TU5070
TU5071
TU5072
TU5073
D082_40570
D082_40580
D082_40590
D082_40600
D082_40610
D082_40620
D082_40630
D082_40650
D082_40660
D082_40670
D082_40680
D082_40690
50000
52000
54000
56000
58000
60000
Untreated
15°C
42°C
−CO2
Dark
−Fe
HL
−N
−P
Exp. P.
Stat. P.

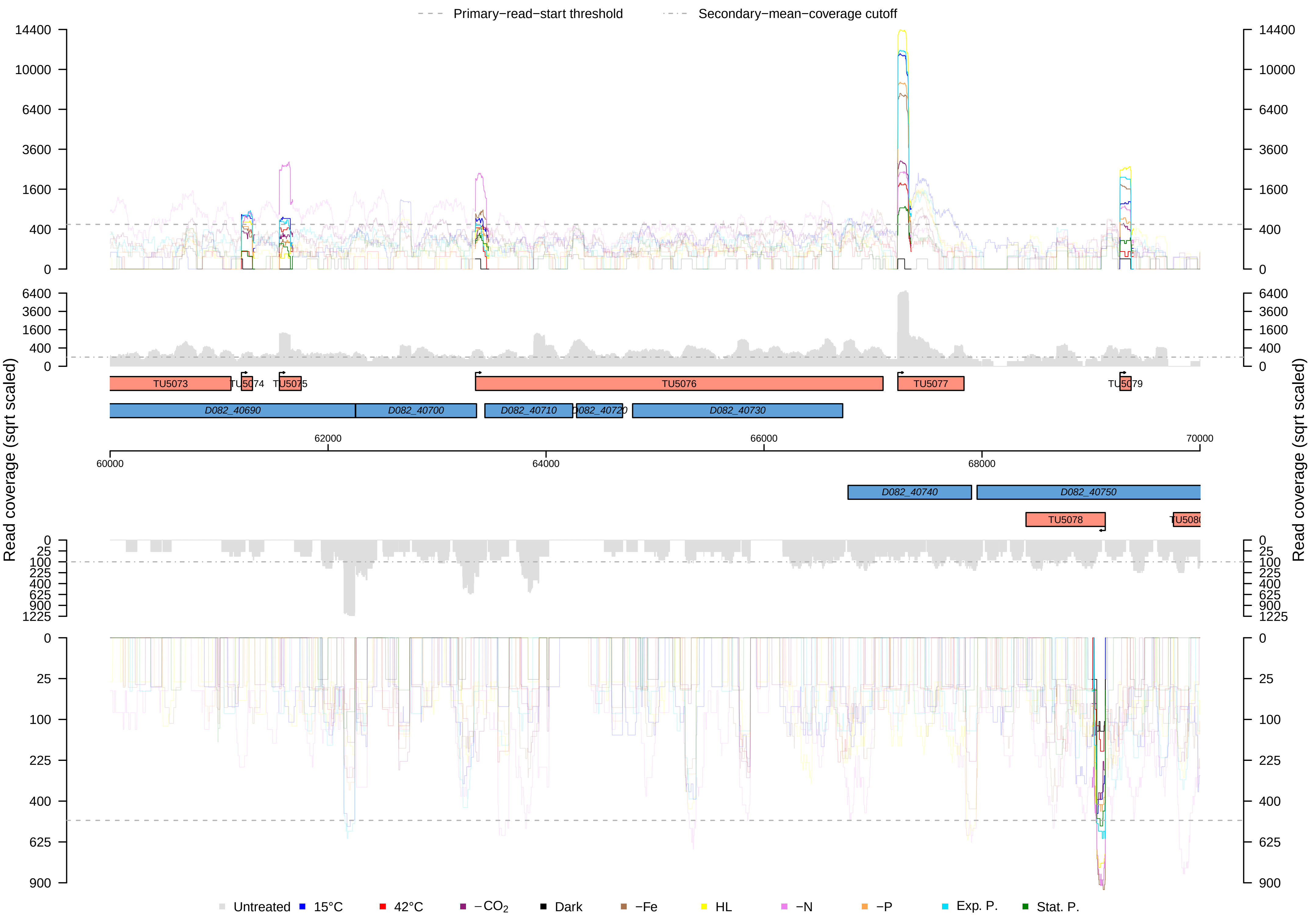

Primary−read−start threshold
Secondary−mean−coverage cutoff
Read coverage (sqrt scaled)
14400
10000
6400
3600
1600
400
0
TU5073
TU5074
TU5075
TU5076
TU5077
TU5079
TU5078
TU5080
D082_40690
D082_40700
D082_40710
D082_40720
D082_40730
D082_40740
D082_40750
60000
62000
64000
66000
68000
70000
0
25
100
225
400
625
900
1225
Untreated
15°C
42°C
−CO2
Dark
−Fe
HL
−N
−P
Exp. P.
Stat. P.

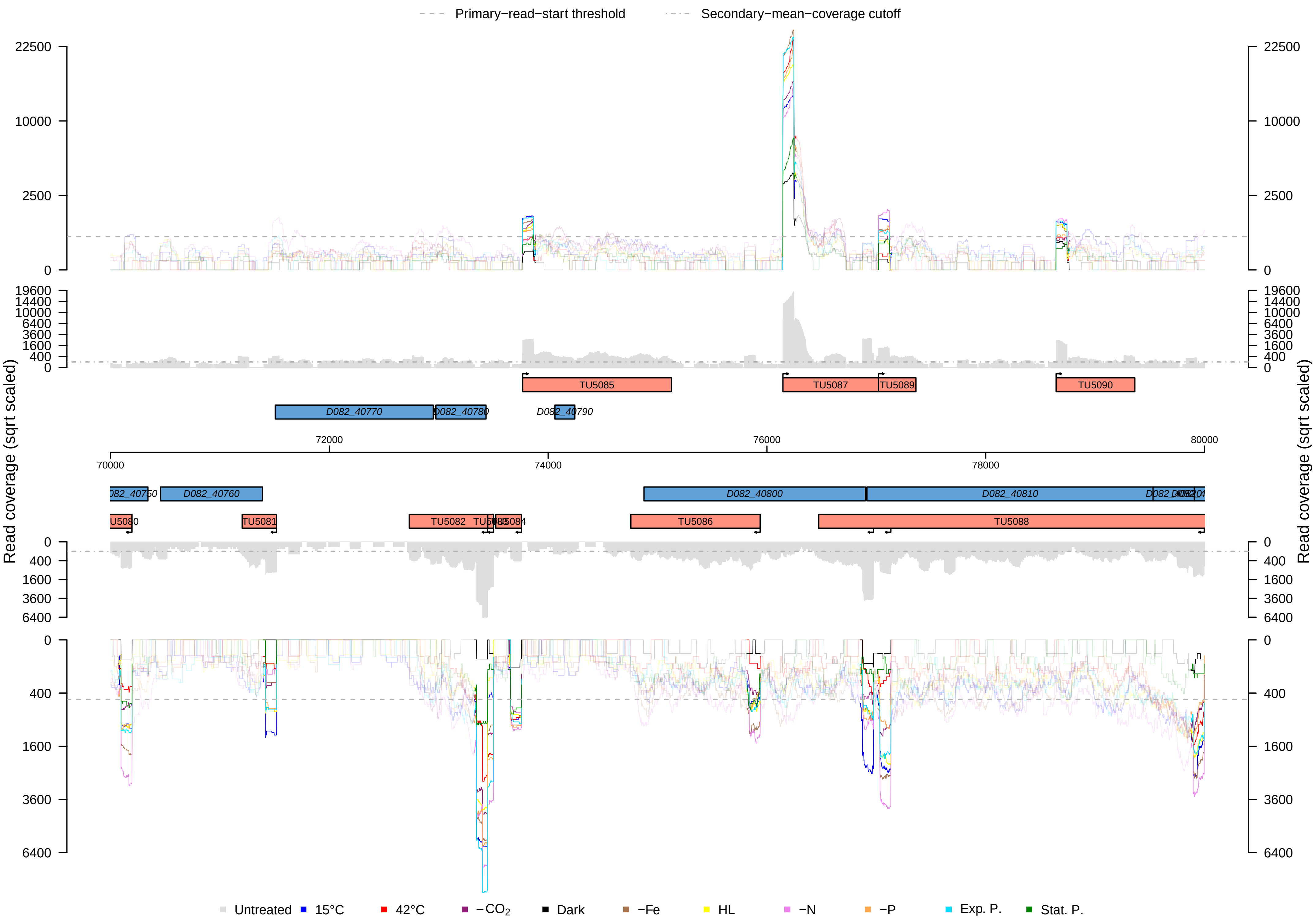

Primary−read−start threshold
Secondary−mean−coverage cutoff
22500
10000
2500
0
19600
14400
10000
6400
3600
1600
400
0
Read coverage (sqrt scaled)
TU5085
TU5087
TU5089
TU5090
D082_40770
D082_40780
D082_40790
72000
76000
80000
70000
74000
78000
D082_40760
D082_40800
D082_40810
TU5081
TU5082
TU5086
TU5088
0
400
1600
3600
6400
Untreated
15°C
42°C
−CO2
Dark
−Fe
HL
−N
−P
Exp. P.
Stat. P.

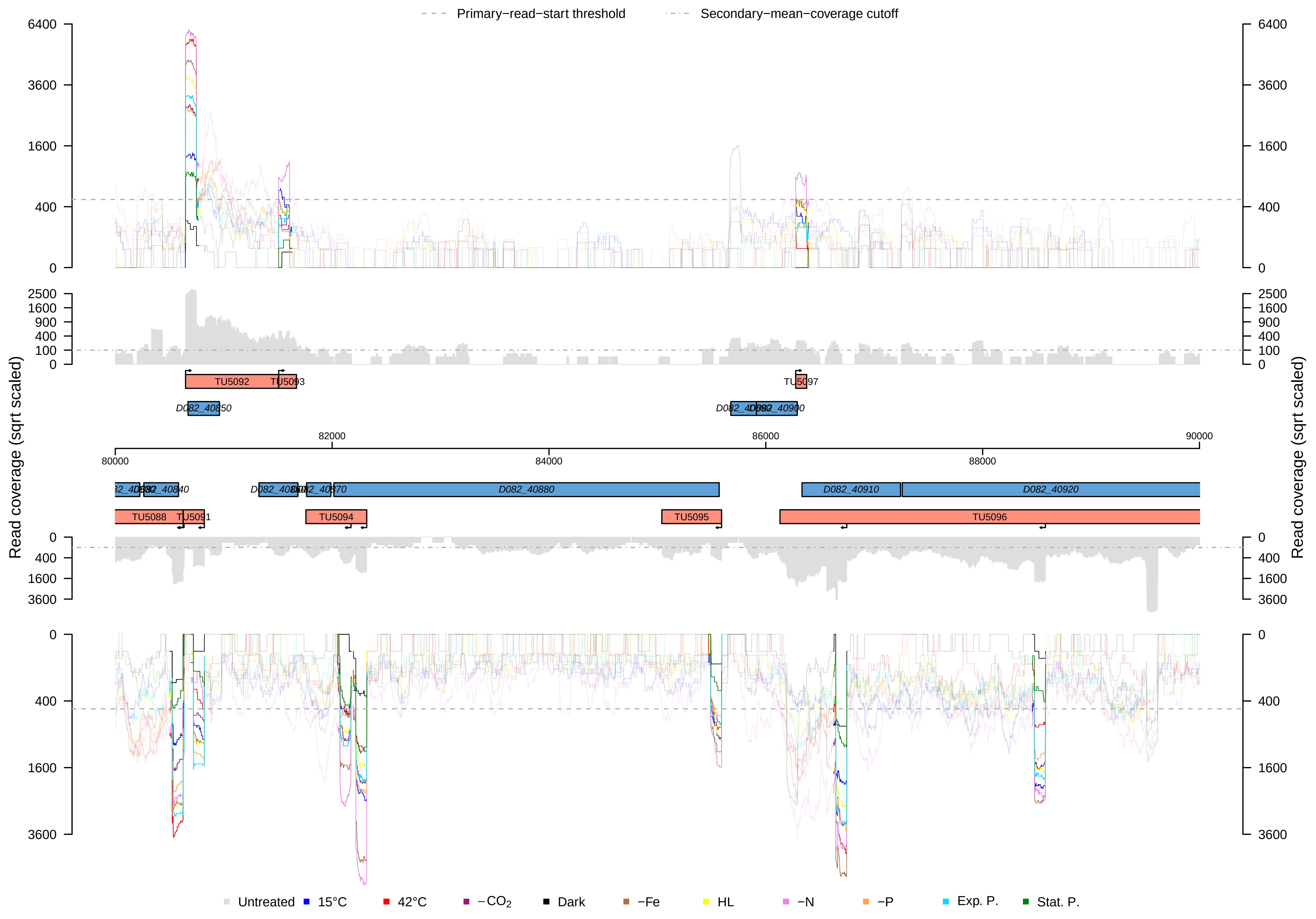

Primary−read−start threshold
Secondary−mean−coverage cutoff
Read coverage (sqrt scaled)
TU5092
TU5093
TU5097
D082_40850
D082_40900
D082_40840
D082_40870
D082_40880
D082_40910
D082_40920
TU5088
TU5091
TU5094
TU5095
TU5096
80000
82000
84000
86000
88000
90000
Untreated
15°C
42°C
−CO2
Dark
−Fe
HL
−N
−P
Exp. P.
Stat. P.

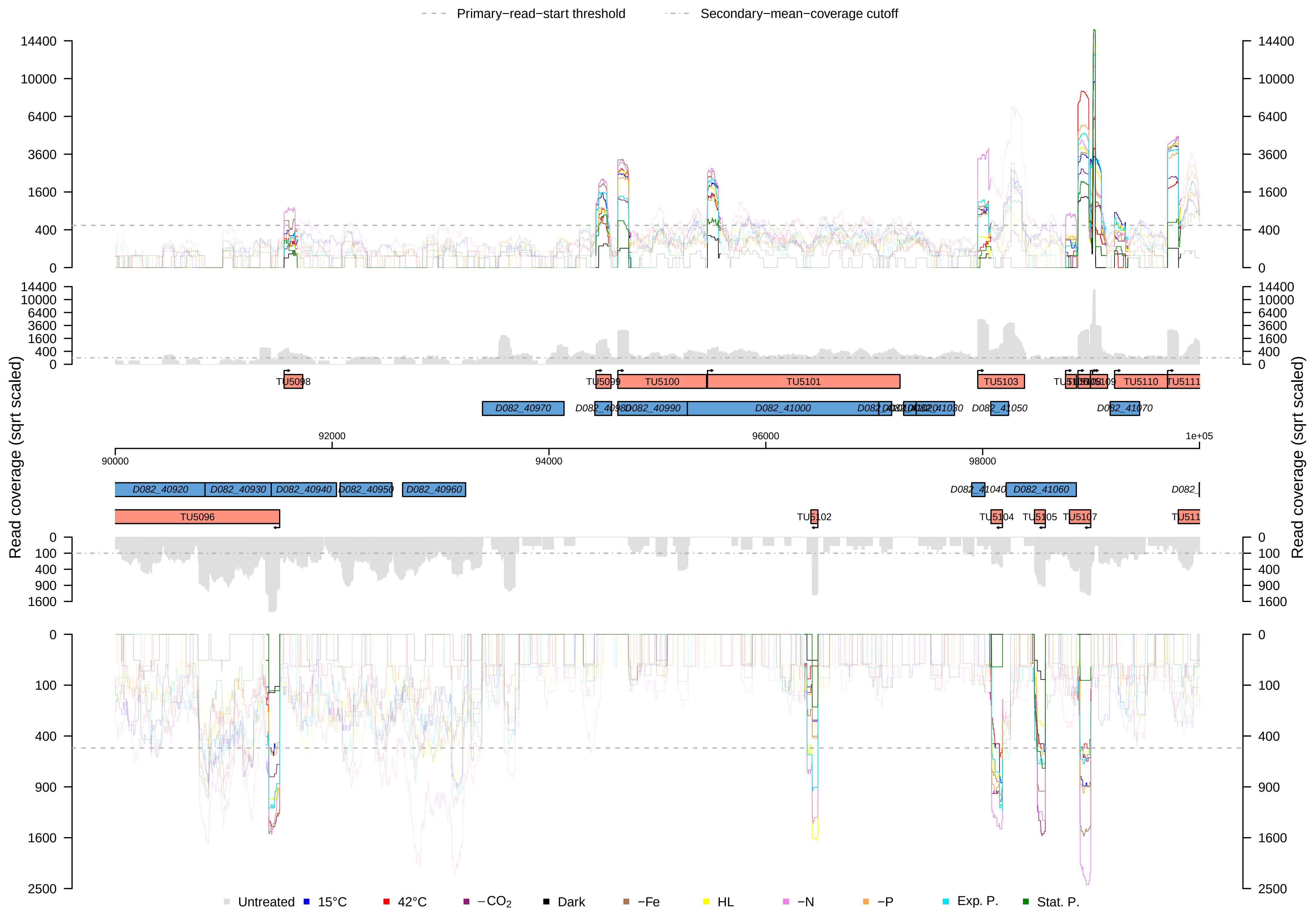

Primary−read−start threshold
Secondary−mean−coverage cutoff
Read coverage (sqrt scaled)
TU5096
TU5098
TU5099
TU5100
TU5101
TU5102
TU5103
TU5104
TU5105
TU5107
TU5109
TU5110
TU5111
D082_40920
D082_40930
D082_40940
D082_40950
D082_40960
D082_40970
D082_40990
D082_41000
D082_41030
D082_41040
D082_41050
D082_41060
D082_41070
90000
92000
94000
96000
98000
1e+05
Untreated
15°C
42°C
−CO2
Dark
−Fe
HL
−N
−P
Exp. P.
Stat. P.

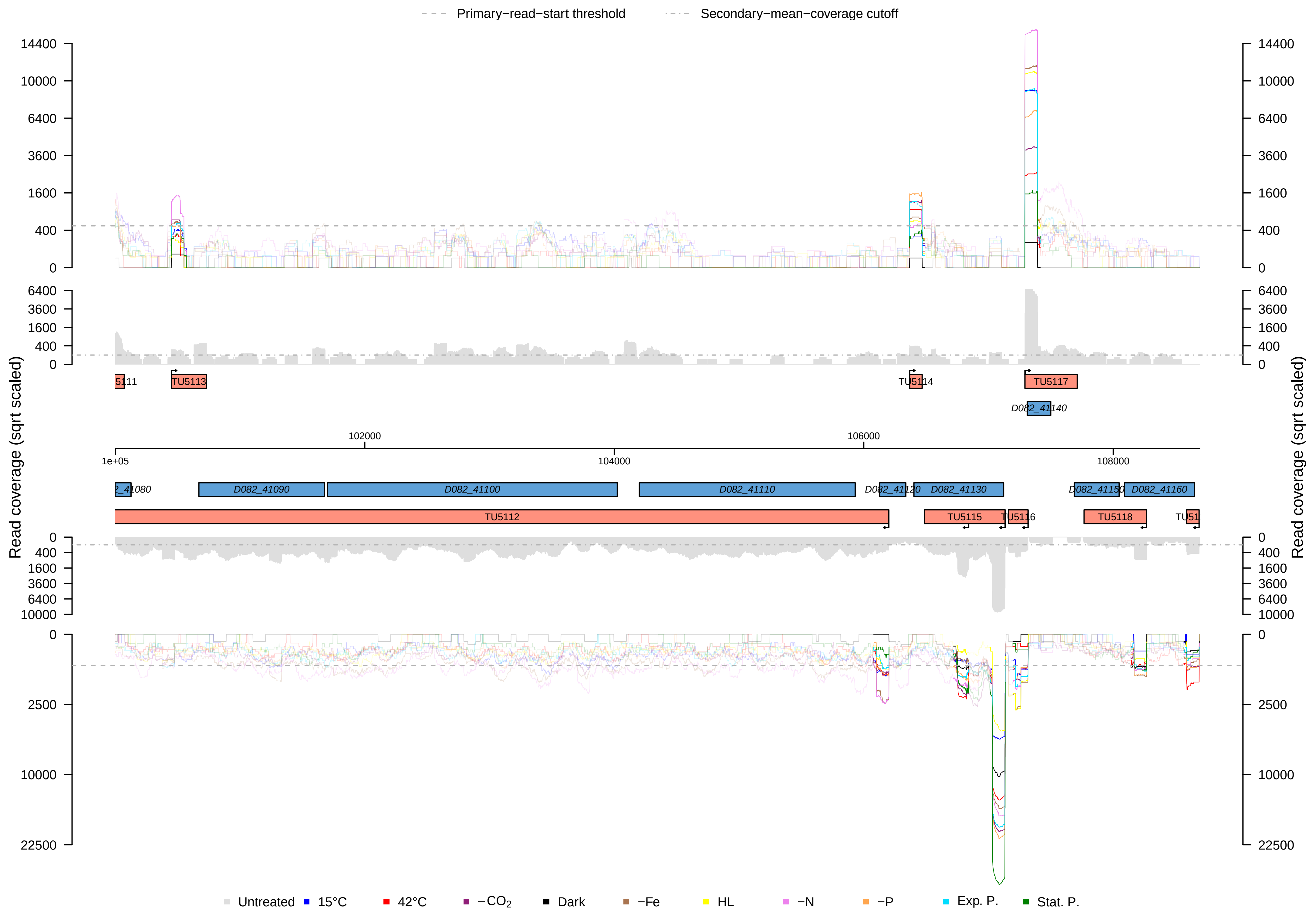

Primary−read−start threshold
Secondary−mean−coverage cutoff
Read coverage (sqrt scaled)
14400
10000
6400
3600
1600
400
0
TU5113
TU5114
TU5117
D082_41140
102000
106000
1e+05
104000
108000
D082_41090
D082_41100
D082_41110
D082_41130
D082_41160
TU5112
TU5115
TU5118
2500
22500
Untreated
15°C
42°C
−CO2
Dark
−Fe
HL
−N
−P
Exp. P.
Stat. P.
