# Supplementary material for: Variations in the non-coding transcriptome as a driver of inter-strain divergence and physiological adaptation in bacteria
Source: Sci Rep. 2015 Apr 22;5:9560. doi: 10.1038/srep09560 (PMC5386190; doi:10.1038/srep09560)
Supplement: Supplementary Information — Supporting File 3 [file srep09560-s3.pdf]

Variations in the non-coding transcriptome as a driver of inter-strain divergence and physiological adaptation in bacteria

Matthias Kopf*, Stephan Klähn*, Ingeborg Scholz, Wolfgang R. Hess[§] and Björn Voß
Genetics and Experimental Bioinformatics, Faculty of Biology, University of Freiburg,
Schänzlestr. 1, 79104 Freiburg, Germany
[§]Corresponding author
*shared first authors

Supporting file S3 | Visualization of the dRNA-seq data for the plasmid pSYLB of *Synechocystis* sp. PCC 6714.

The colored graphs represent the coverage of reads from the treated libraries for ten sequenced conditions. The corresponding colors are given at the bottom of each page. The TSS positions are indicated by black arrows, e.g. at the beginning of each predicted transcriptional unit (TU, shown in red), and the read coverage graphs are highlighted for the following 100 bp. The coverage from the untreated library is shown grey. The y-axis of the graphs is square-root-scaled but the tick labels show the true normalized coverage values. Protein coding genes are shown in blue, tRNA in green and rRNA in dark grey. The data for the forward strand is shown above and data for the reverse strand below the x-axis. The x-axis shows the nucleotide position of the plasmid pSYLB in bp. This document is searchable by locus tags from *Synechocystis* sp. PCC 6714, gene names (if available), nucleotide position (every 2000 bp) and the *Synechocystis* sp. PCC 6803 locus tags of the 2854 orthologous protein-coding genes.

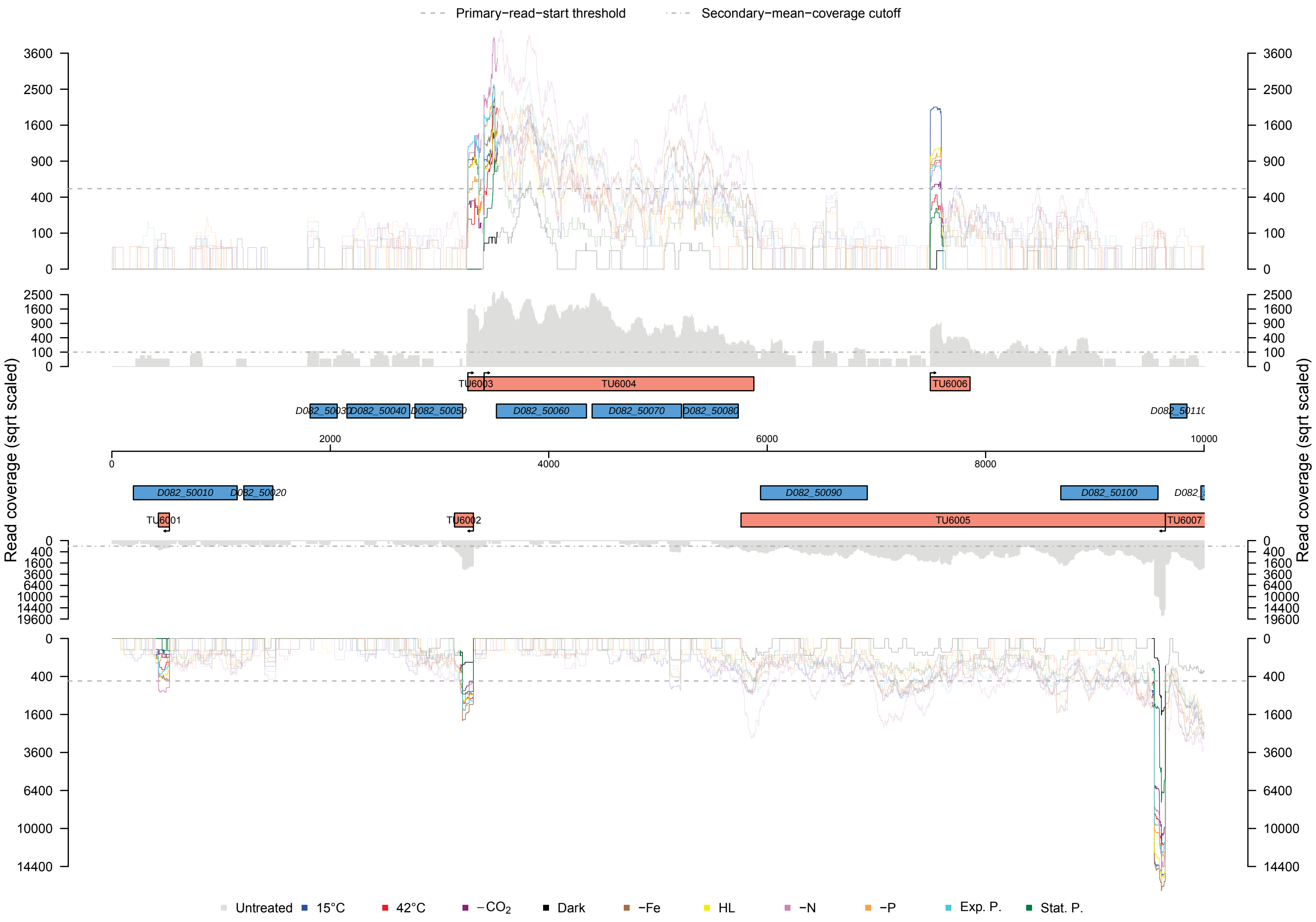

Primary−read−start threshold
Secondary−mean−coverage cutoff
Read coverage (sqrt scaled)
3600
2500
1600
900
400
100
0
TU6003
TU6004
TU6006
D082_50030
D082_50040
D082_50050
D082_50060
D082_50070
D082_50080
D082_50110
0
2000
4000
6000
8000
10000
D082_50010
D082_50020
D082_50090
D082_50100
TU6001
TU6002
TU6005
TU6007
19600
14400
10000
6400
Untreated
15°C
42°C
−CO2
Dark
−Fe
HL
−N
−P
Exp. P.
Stat. P.

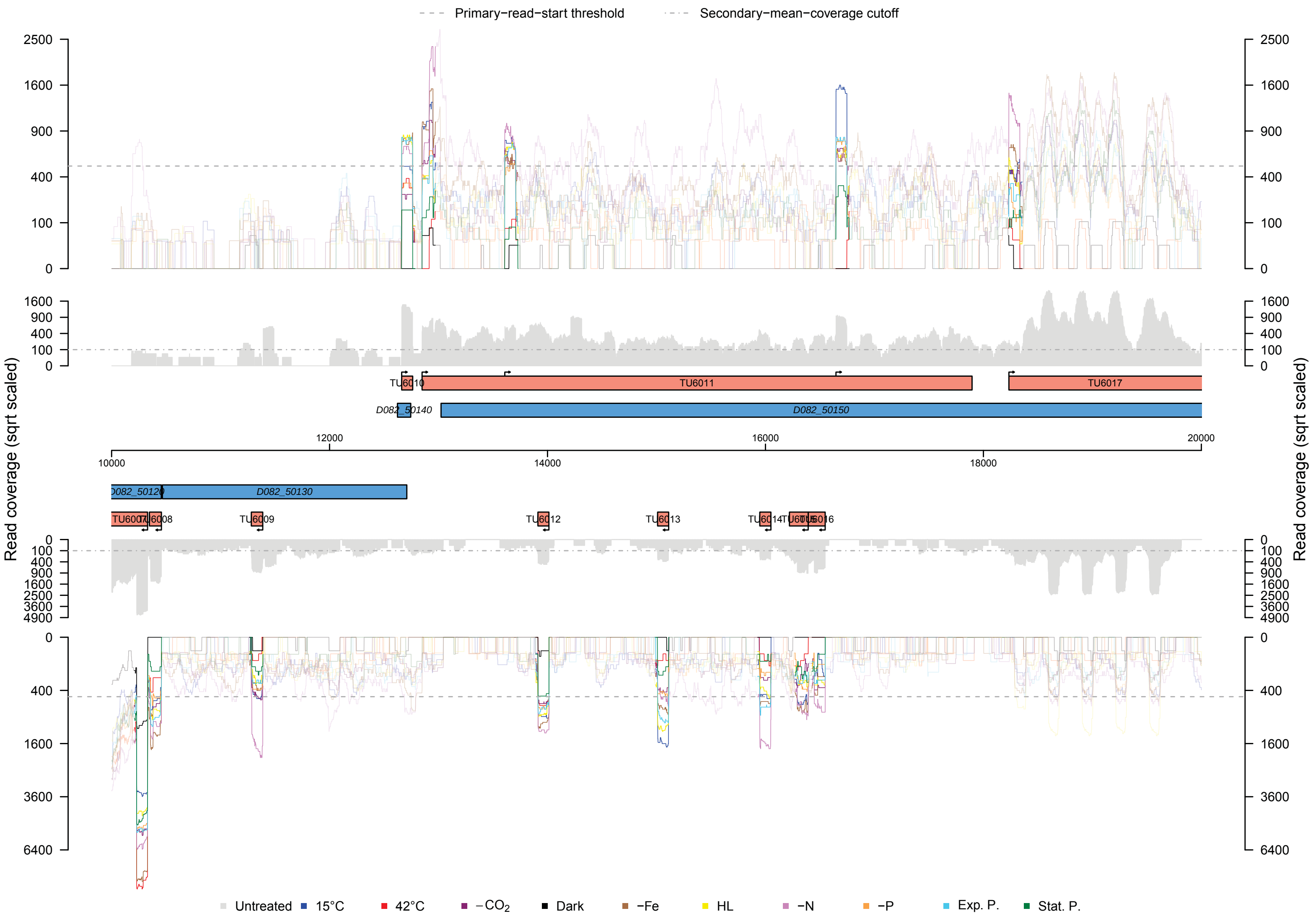

Primary−read−start threshold
Secondary−mean−coverage cutoff
Read coverage (sqrt scaled)
2500
1600
900
400
100
0
4900
3600
6400
TU6010
TU6011
TU6017
D082_50140
D082_50150
D082_50120
D082_50130
TU6007
TU6008
TU6009
TU6012
TU6013
TU6014
TU6015
TU6016
10000
12000
14000
16000
18000
20000
Untreated
15°C
42°C
−CO2
Dark
−Fe
HL
−N
−P
Exp. P.
Stat. P.

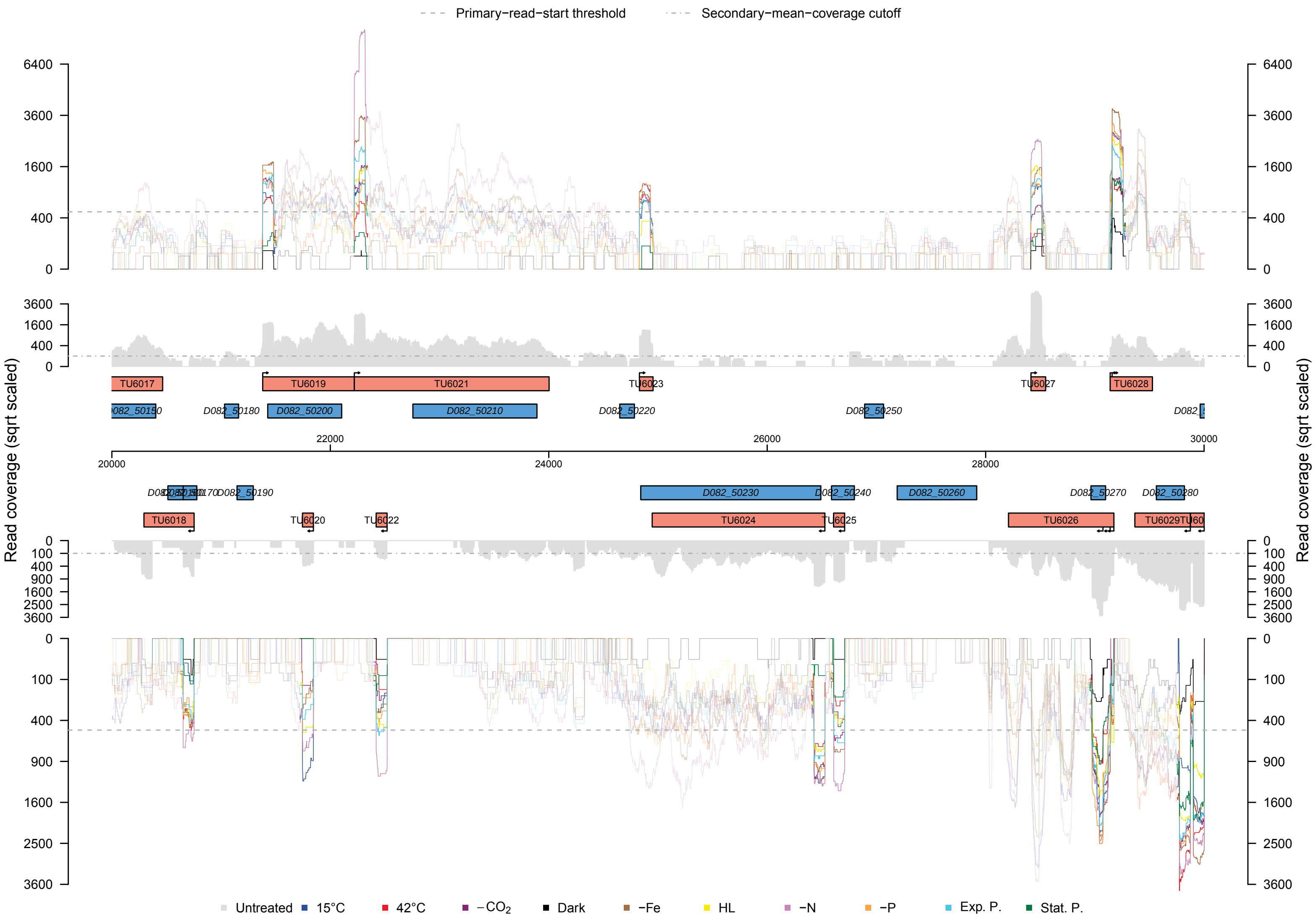

Primary−read−start threshold
Secondary−mean−coverage cutoff
Read coverage (sqrt scaled)
TU6017
TU6019
TU6021
TU6023
TU6027
TU6028
TU6018
TU6020
TU6022
TU6024
TU6025
TU6026
D082_50200
D082_50210
D082_50250
D082_50230
D082_50240
D082_50260
D082_50270
D082_50280
20000
22000
24000
26000
28000
30000
Untreated
15°C
42°C
−CO2
Dark
−Fe
HL
−N
−P
Exp. P.
Stat. P.

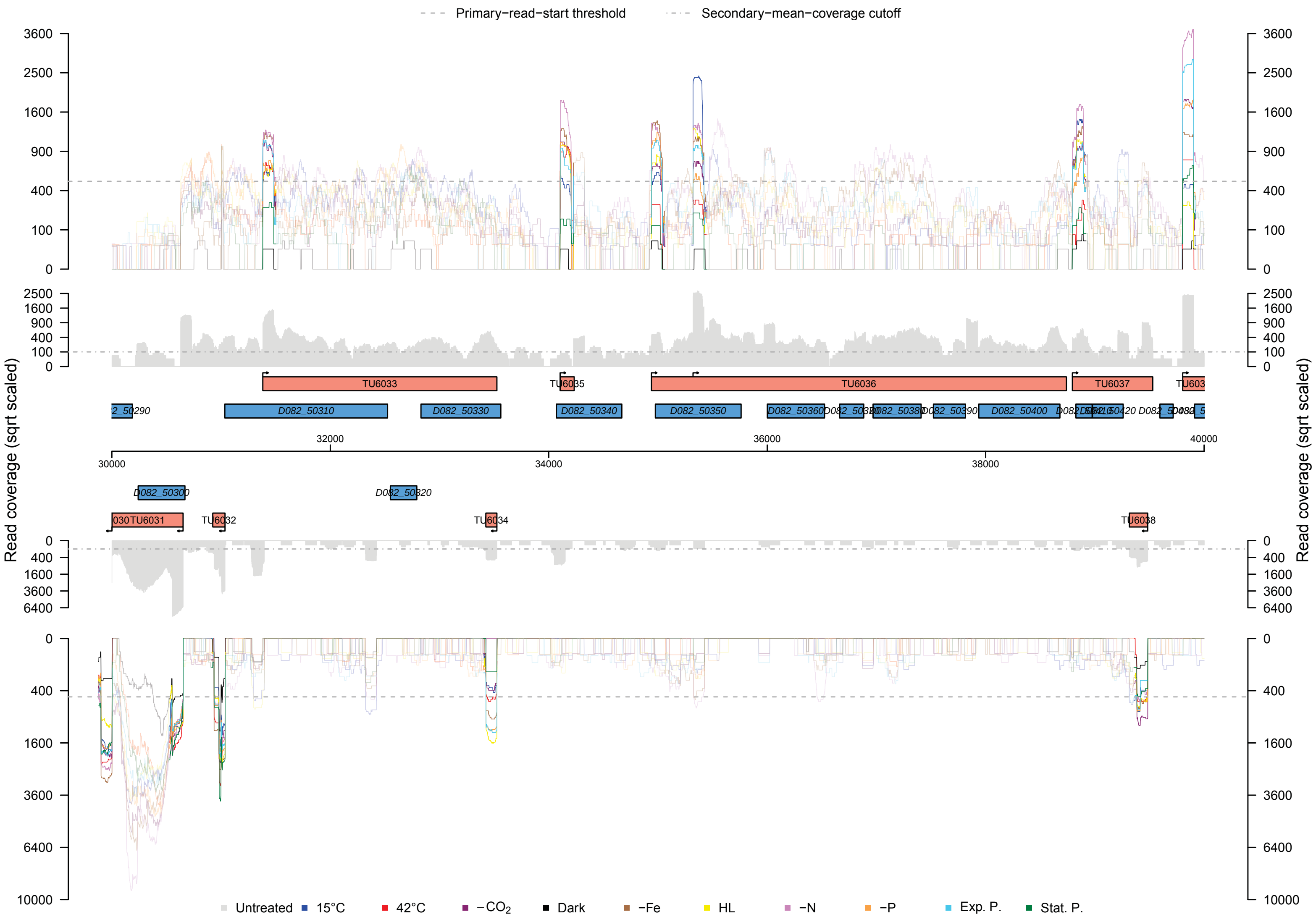

Primary−read−start threshold
Secondary−mean−coverage cutoff
Read coverage (sqrt scaled)
3600
2500
1600
900
400
100
0
TU6033
TU6035
TU6036
TU6037
D082_50310
D082_50330
D082_50340
D082_50350
D082_50400
30000
32000
34000
36000
38000
40000
D082_50300
TU6031
TU6032
TU6034
TU6038
6400
10000
Untreated
15°C
42°C
−CO2
Dark
−Fe
HL
−N
−P
Exp. P.
Stat. P.

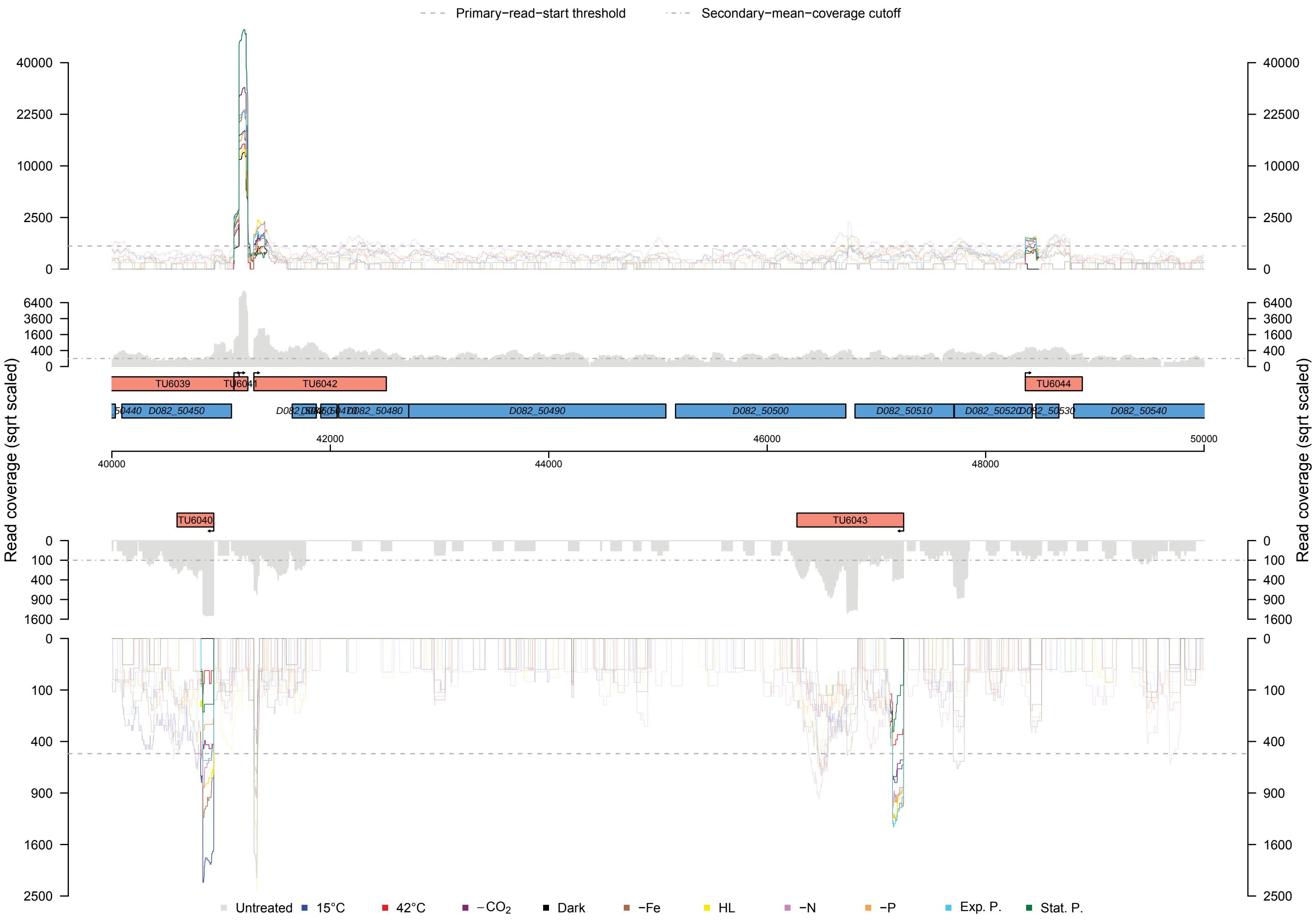
Primary−read−start threshold
Secondary−mean−coverage cutoff
Read coverage (sqrt scaled)
40000
22500
10000
2500
0
6400
3600
1600
400
TU6039
TU6041
TU6042
TU6044
D082_50450
D082_50480
D082_50490
D082_50500
D082_50510
D082_50520
D082_50540
40000
42000
44000
46000
48000
50000
TU6040
TU6043
100
900
2500
Untreated
15°C
42°C
−CO2
Dark
−Fe
HL
−N
−P
Exp. P.
Stat. P.

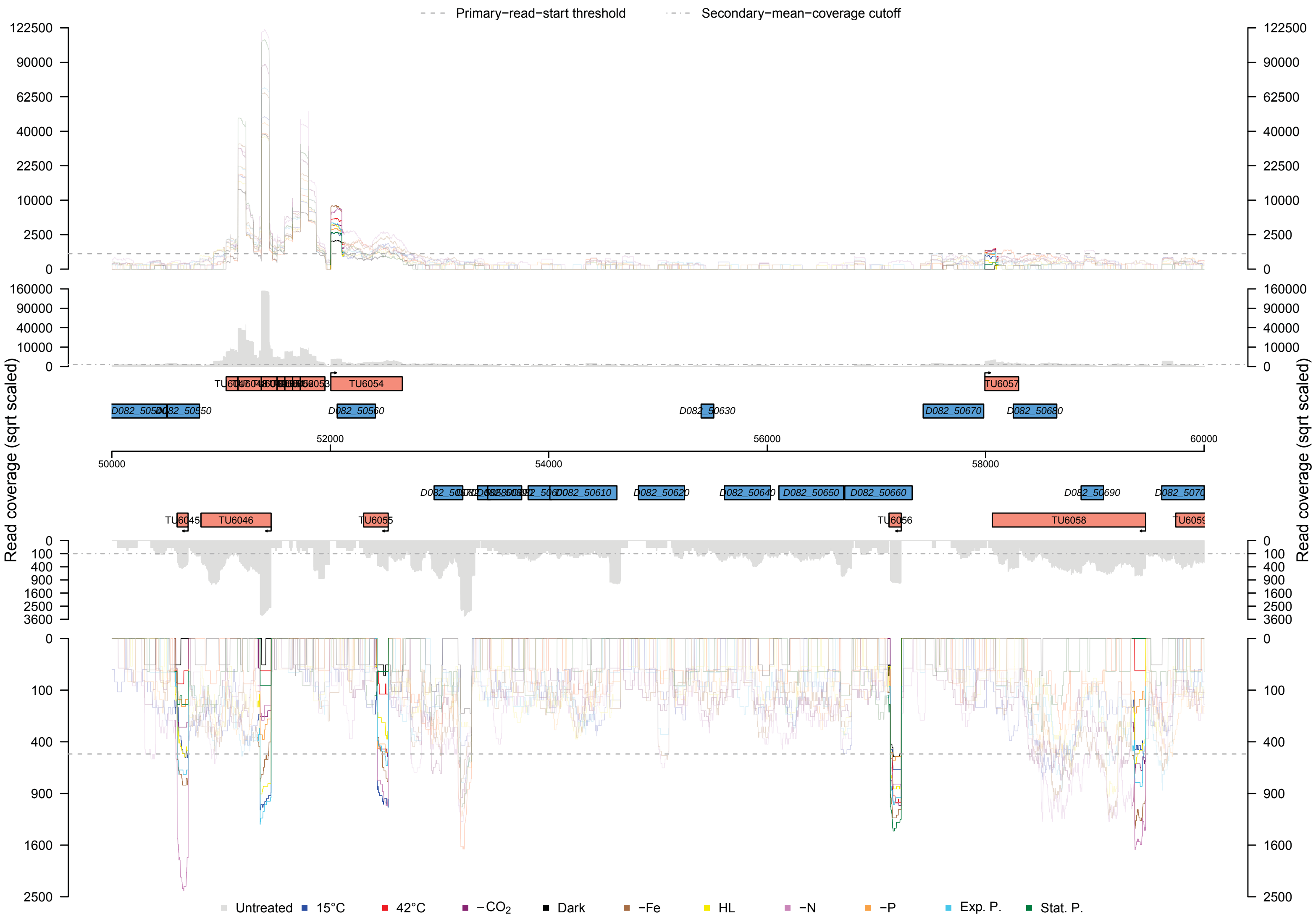

Primary−read−start threshold
Secondary−mean−coverage cutoff
Read coverage (sqrt scaled)
TU6054
TU6057
D082_50560
D082_50630
D082_50670
D082_50550
52000
56000
60000
50000
54000
58000
D082_50610
D082_50620
D082_50640
D082_50650
D082_50660
D082_50690
TU6046
TU6058
Untreated
15°C
42°C
−CO2
Dark
−Fe
HL
−N
−P
Exp. P.
Stat. P.

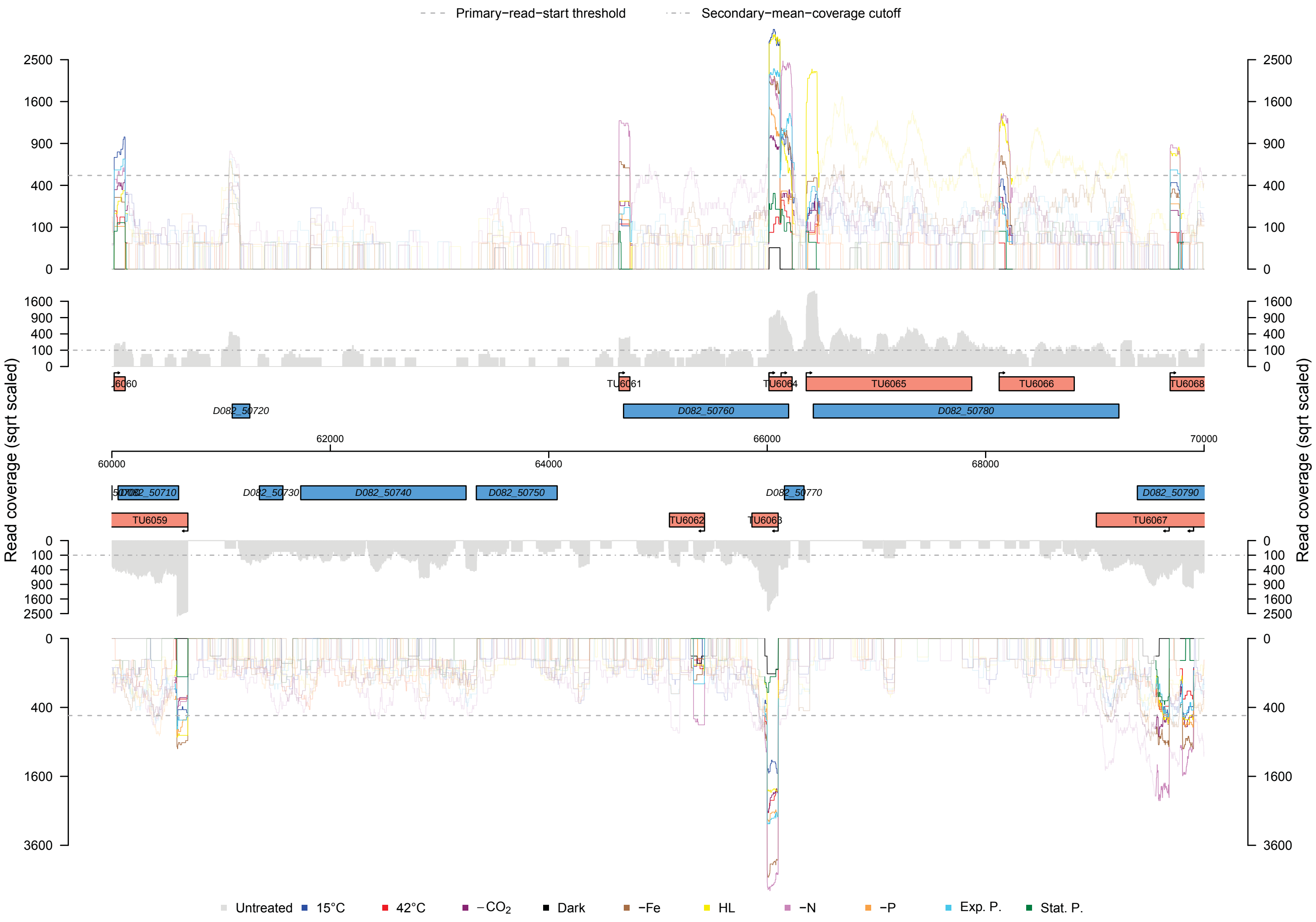

Primary−read−start threshold
Secondary−mean−coverage cutoff
Read coverage (sqrt scaled)
2500
1600
900
400
100
0
3600
TU6060
TU6061
TU6064
TU6065
TU6066
TU6068
D082_50720
D082_50760
D082_50780
60000
62000
64000
66000
68000
70000
D082_50710
D082_50730
D082_50740
D082_50750
D082_50770
D082_50790
TU6059
TU6062
TU6063
TU6067
Untreated
15°C
42°C
−CO2
Dark
−Fe
HL
−N
−P
Exp. P.
Stat. P.

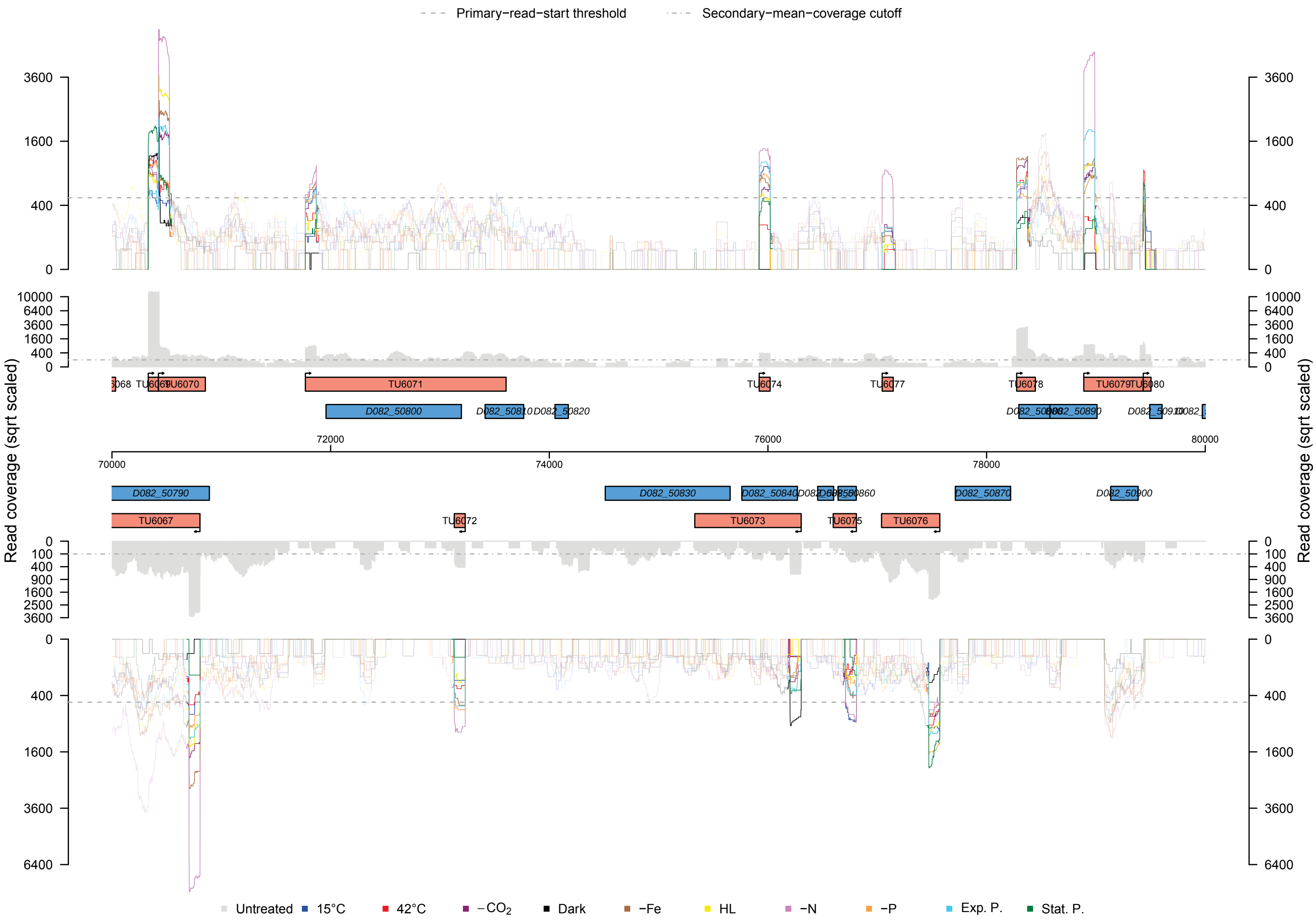

Primary−read−start threshold
Secondary−mean−coverage cutoff
Read coverage (sqrt scaled)
3600
1600
400
0
10000
6400
3600
1600
400
0
TU6067
TU6070
TU6071
TU6072
TU6073
TU6074
TU6075
TU6076
TU6077
TU6078
TU6079
D082_50790
D082_50800
D082_50830
D082_50840
D082_50870
70000
72000
74000
76000
78000
80000
100
900
2500
6400
Untreated
15°C
42°C
−CO_2
Dark
−Fe
HL
−N
−P
Exp. P.
Stat. P.

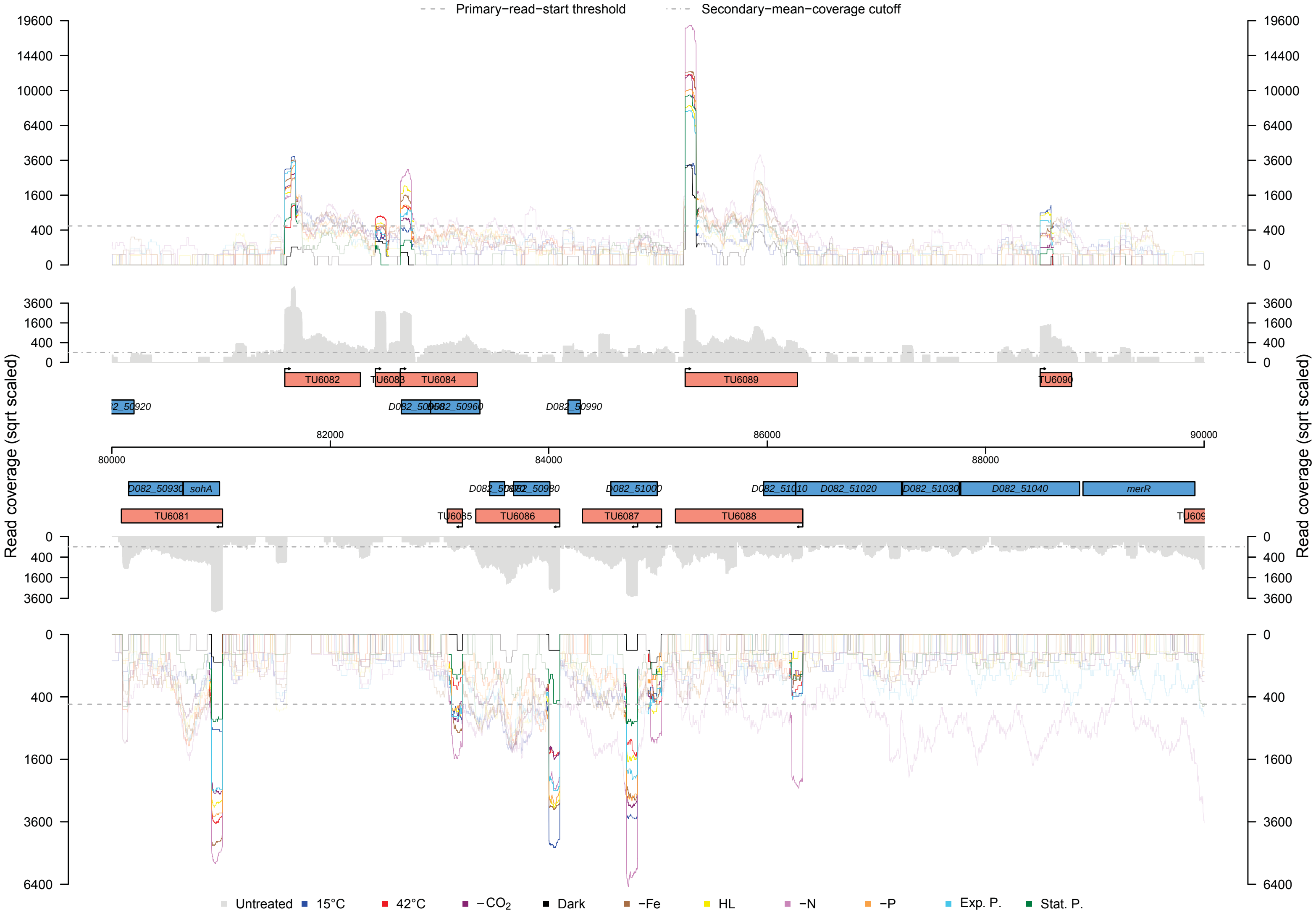

Primary−read−start threshold
Secondary−mean−coverage cutoff
19600
14400
10000
6400
3600
1600
400
0
Read coverage (sqrt scaled)
TU6082
TU6083
TU6084
TU6089
TU6090
D082_50920
D082_50950
D082_50960
D082_50990
80000
82000
84000
86000
88000
90000
D082_50930
sohA
D082_50970
D082_50980
D082_51000
D082_51010
D082_51020
D082_51030
D082_51040
merR
TU6081
TU6085
TU6086
TU6087
TU6088
Untreated
15°C
42°C
−CO2
Dark
−Fe
HL
−N
−P
Exp. P.
Stat. P.

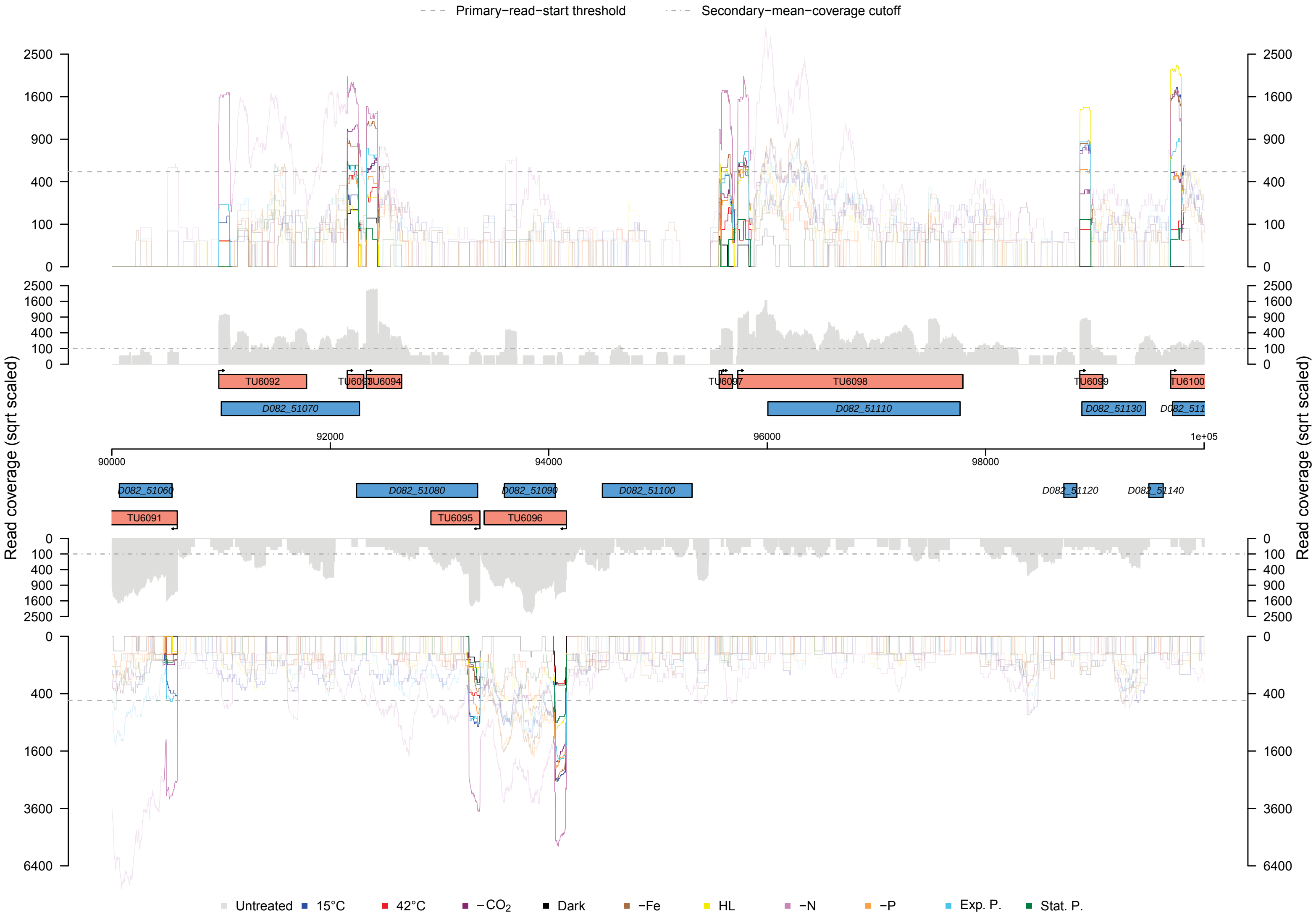

Primary−read−start threshold
Secondary−mean−coverage cutoff
Read coverage (sqrt scaled)
TU6091
TU6092
TU6093
TU6094
TU6095
TU6096
TU6097
TU6098
TU6099
TU6100
D082_51060
D082_51070
D082_51080
D082_51090
D082_51100
D082_51110
D082_51120
D082_51130
D082_51140
90000
92000
94000
96000
98000
1e+05
Untreated
15°C
42°C
−CO2
Dark
−Fe
HL
−N
−P
Exp. P.
Stat. P.

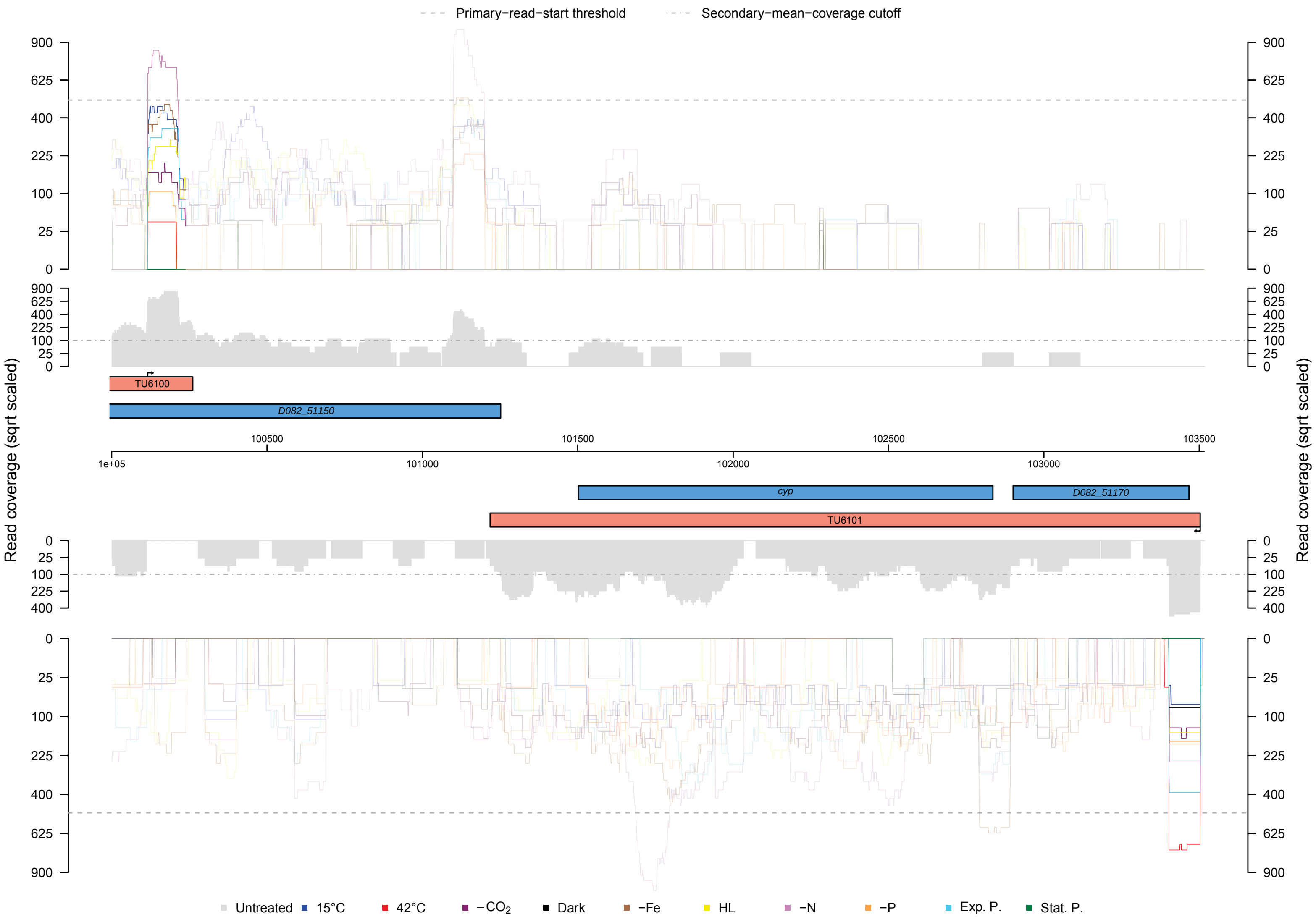

Primary−read−start threshold
Secondary−mean−coverage cutoff
Read coverage (sqrt scaled)
900
625
400
225
100
25
0
TU6100
D082_51150
100500
101500
102500
103500
1e+05
101000
102000
103000
cyp
D082_51170
TU6101
Untreated
15°C
42°C
−CO2
Dark
−Fe
HL
−N
−P
Exp. P.
Stat. P.
