# Supplementary material for: Variations in the non-coding transcriptome as a driver of inter-strain divergence and physiological adaptation in bacteria
Source: Sci Rep. 2015 Apr 22;5:9560. doi: 10.1038/srep09560 (PMC5386190; doi:10.1038/srep09560)
Supplement: Supplementary Information — Supporting File 1 [file srep09560-s1.pdf]

Variations in the non-coding transcriptome as a driver of inter-strain divergence and physiological adaptation in bacteria

Matthias Kopf*, Stephan Klähn*, Ingeborg Scholz, Wolfgang R. Hess[§] and Björn Voß
Genetics and Experimental Bioinformatics, Faculty of Biology, University of Freiburg,
Schänzlestr. 1, 79104 Freiburg, Germany
[§]Corresponding author
*shared first authors

Supporting file S1 | Visualization of the dRNA-seq data for the chromosome of *Synechocystis* sp. PCC 6714.

The colored graphs represent the coverage of reads from the treated libraries for ten sequenced conditions. The corresponding colors are given at the bottom of each page. The TSS positions are indicated by black arrows, e.g. at the beginning of each predicted transcriptional unit (TU, shown in red), and the read coverage graphs are highlighted for the following 100 bp. The coverage from the untreated library is shown grey. In order to reduce file size, only the highlighted graph areas are plotted in single nucleotide resolution while the transparent and the untreated coverage graphs were averaged over 41 nt and 11 nt long windows, respectively. The y-axis of the graphs is square-root-scaled but the tick labels show the true normalized coverage values. Protein coding genes are shown in blue, tRNA in green and rRNA in dark grey. The data for the forward strand is shown above and data for the reverse strand below the x-axis. The x-axis shows the chromosomal position in bp. This document is searchable by locus tags from *Synechocystis* sp. PCC 6714, gene names (if available), nucleotide position (every 2000 bp) and the *Synechocystis* sp. PCC 6803 locus tags of the 2854 orthologous protein-coding genes.

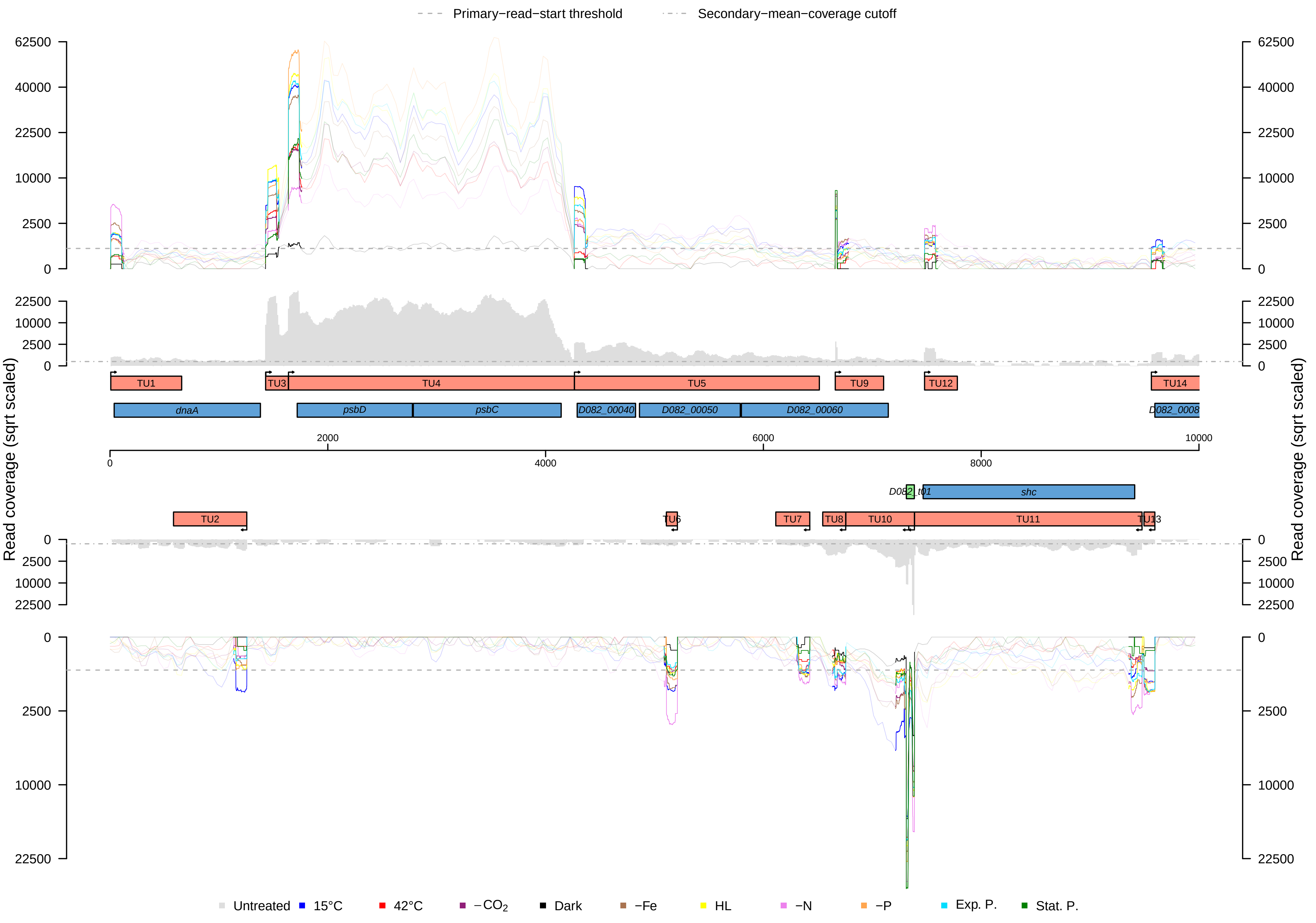

Primary−read−start threshold
Secondary−mean−coverage cutoff
Read coverage (sqrt scaled)
62500
40000
22500
10000
2500
0
TU1
TU3
TU4
TU5
TU9
TU12
TU14
dnaA
psbD
psbC
D082_00040
D082_00050
D082_00060
D082_0008
0
2000
4000
6000
8000
10000
D082_t01
shc
TU2
TU6
TU7
TU8
TU10
TU11
TU13
Untreated
15°C
42°C
−CO2
Dark
−Fe
HL
−N
−P
Exp. P.
Stat. P.

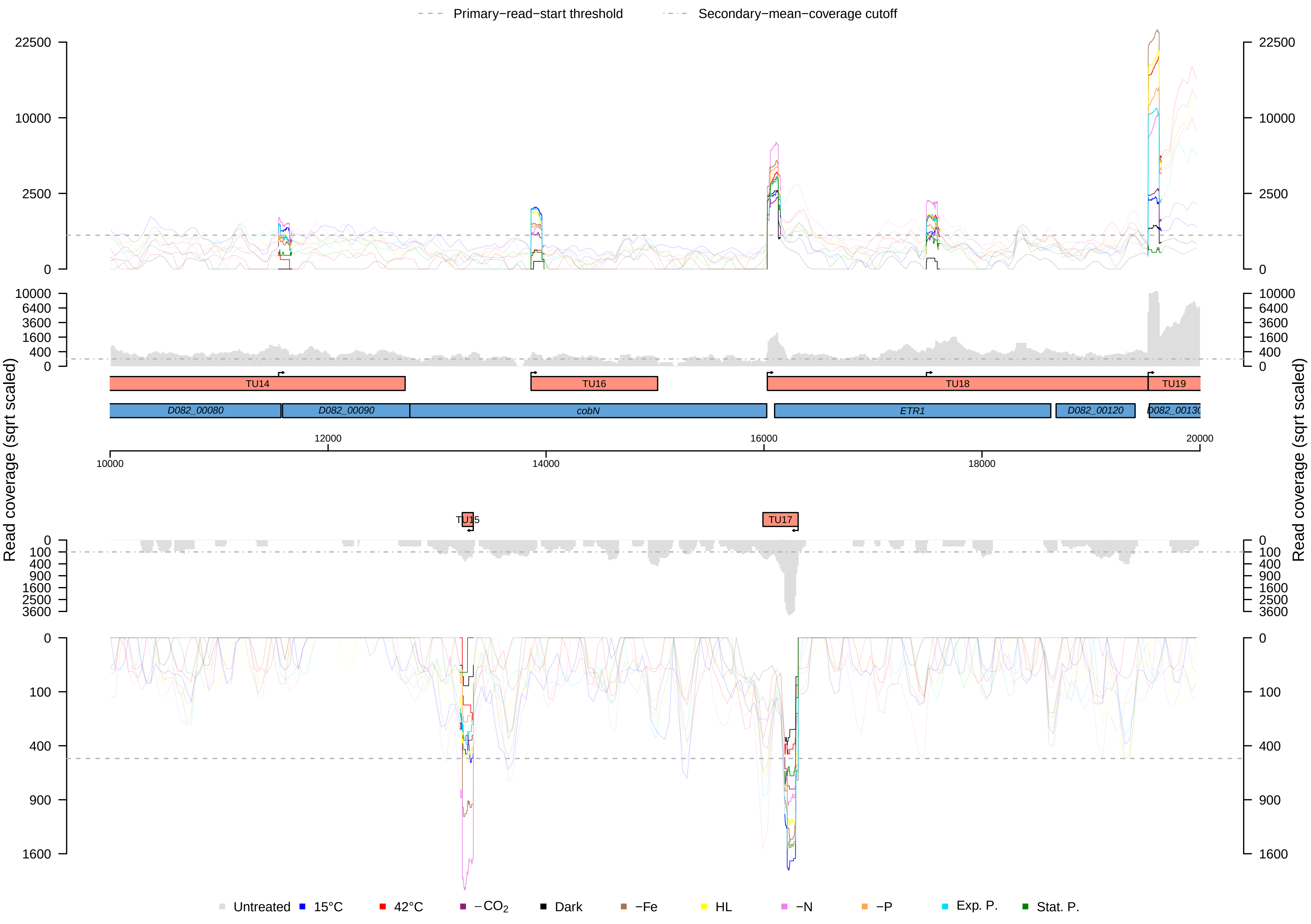

Primary−read−start threshold
Secondary−mean−coverage cutoff
Read coverage (sqrt scaled)
22500
10000
2500
0
6400
3600
1600
400
100
900
TU14
TU15
TU16
TU17
TU18
TU19
D082_00080
D082_00090
cobN
ETR1
D082_00120
10000
12000
14000
16000
18000
20000
Untreated
15°C
42°C
−CO2
Dark
−Fe
HL
−N
−P
Exp. P.
Stat. P.

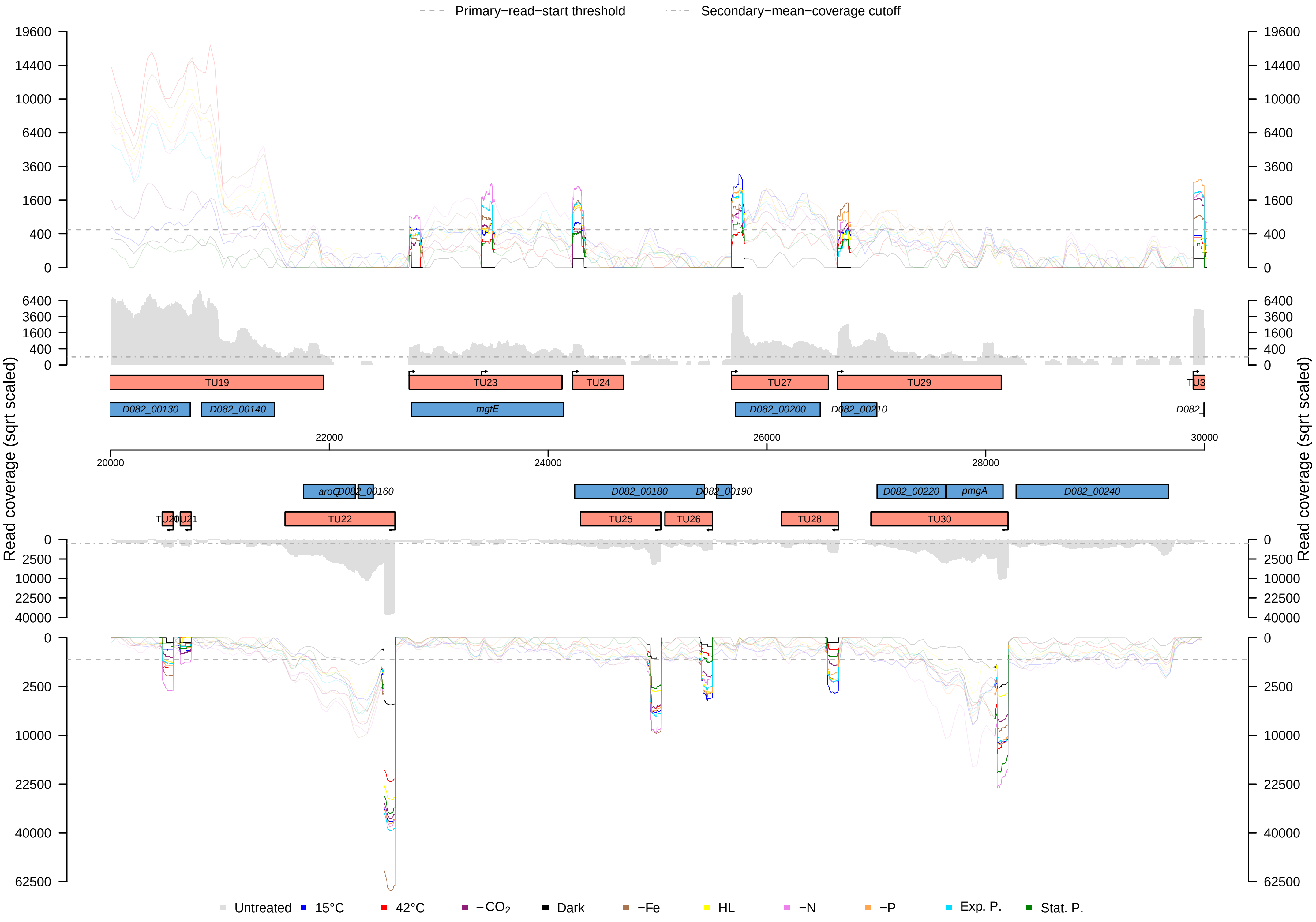

Primary−read−start threshold
Secondary−mean−coverage cutoff
Read coverage (sqrt scaled)
19600
14400
10000
6400
3600
1600
400
0
TU19
TU23
TU24
TU27
TU29
TU3
D082_00130
D082_00140
mgtE
D082_00200
D082_00210
D082_
20000
22000
24000
26000
28000
30000
aroQ
D082_00160
D082_00180
D082_00190
D082_00220
pmgA
D082_00240
TU20
TU21
TU22
TU25
TU26
TU28
TU30
2500
10000
22500
40000
62500
Untreated
15°C
42°C
−CO2
Dark
−Fe
HL
−N
−P
Exp. P.
Stat. P.

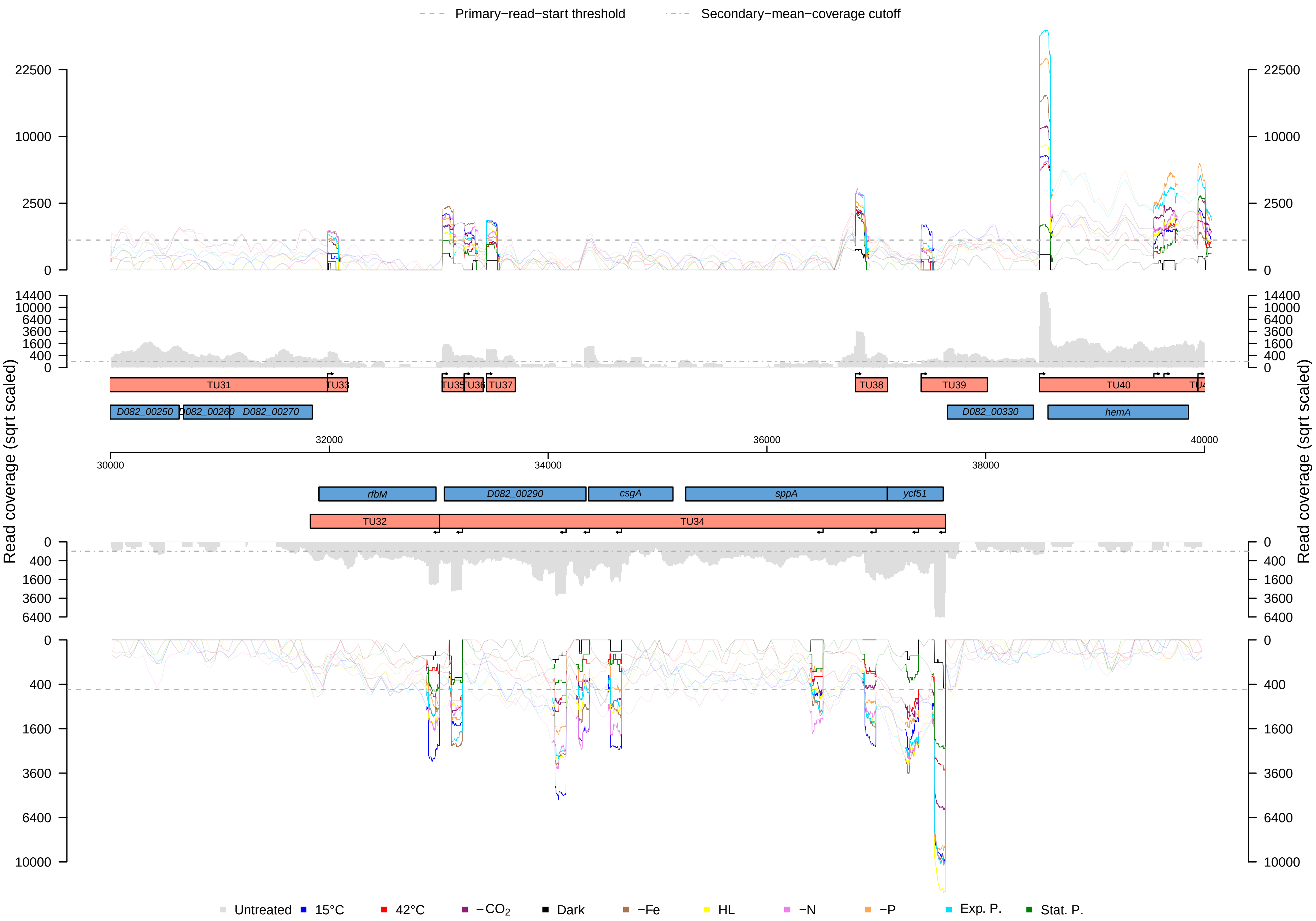

Primary−read−start threshold
Secondary−mean−coverage cutoff
Read coverage (sqrt scaled)
22500
10000
2500
0
14400
10000
6400
3600
1600
400
TU31
TU33
TU35
TU36
TU37
TU38
TU39
TU40
D082_00250
D082_00260
D082_00270
D082_00330
hemA
30000
32000
34000
36000
38000
40000
rfbM
D082_00290
csgA
sppA
ycf51
TU32
TU34
Untreated
15°C
42°C
−CO2
Dark
−Fe
HL
−N
−P
Exp. P.
Stat. P.

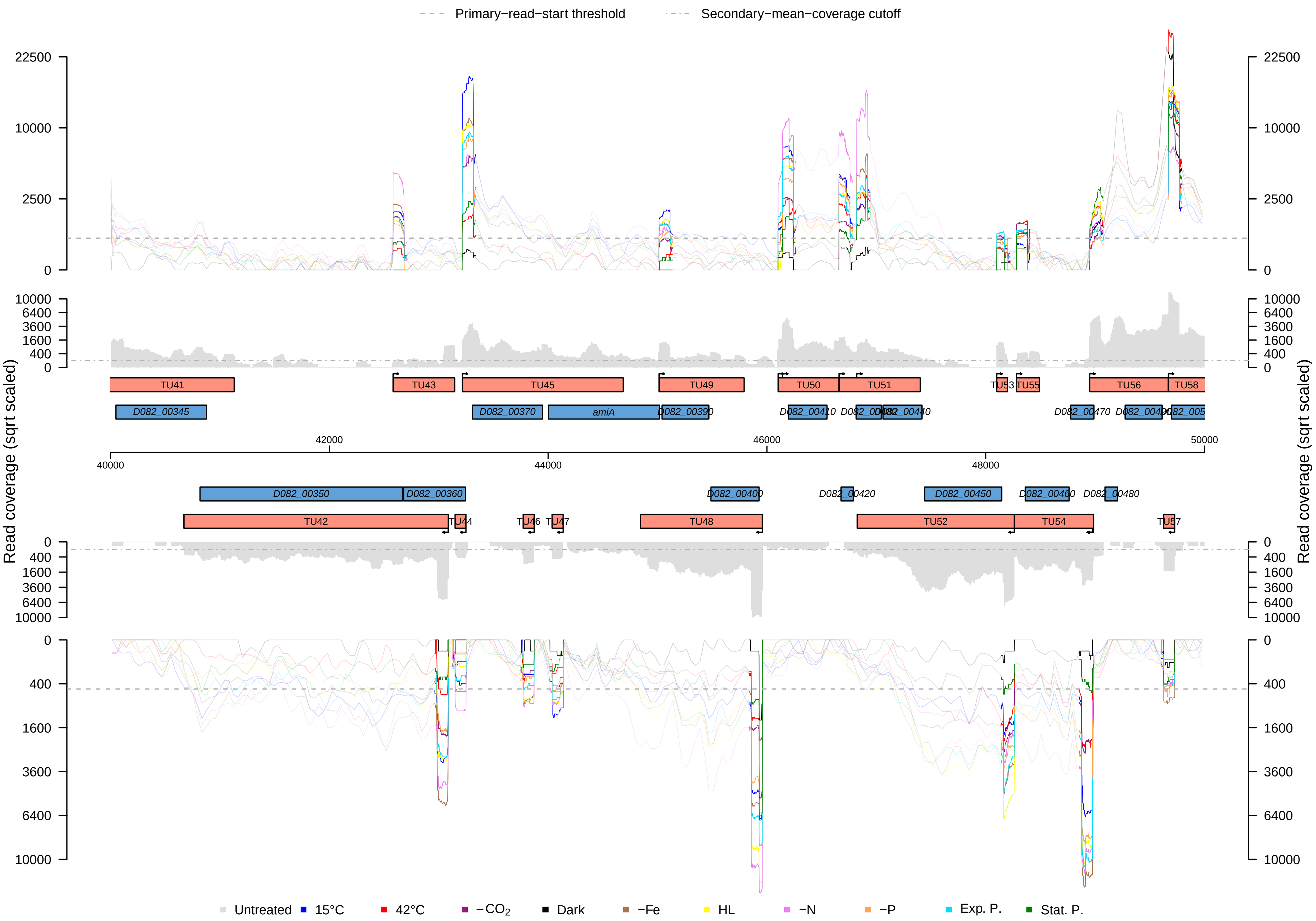

Primary−read−start threshold
Secondary−mean−coverage cutoff
Read coverage (sqrt scaled)
22500
10000
2500
0
6400
3600
1600
400
TU41
TU43
TU45
TU49
TU50
TU51
TU53
TU55
TU56
TU58
D082_00345
D082_00370
amiA
D082_00390
D082_00410
D082_00440
D082_00470
40000
42000
44000
46000
48000
50000
D082_00350
D082_00360
D082_00400
D082_00420
D082_00450
D082_00460
D082_00480
TU42
TU44
TU46
TU47
TU48
TU52
TU54
TU57
Untreated
15°C
42°C
$-CO_2$
Dark
−Fe
HL
−N
−P
Exp. P.
Stat. P.

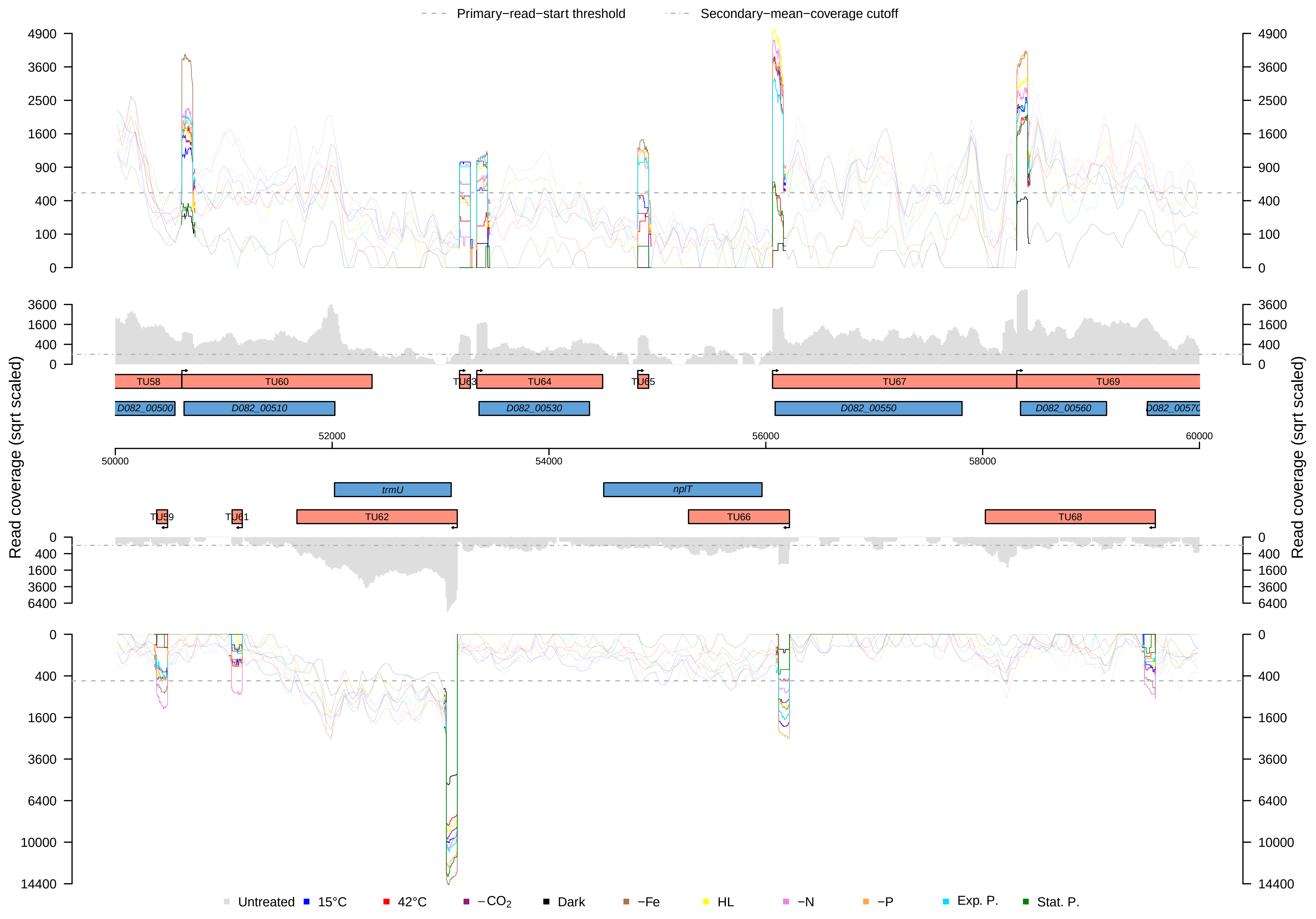

Primary−read−start threshold
Secondary−mean−coverage cutoff
Read coverage (sqrt scaled)
4900
3600
2500
1600
900
400
100
0
6400
10000
14400
TU58
TU59
TU60
TU61
TU62
TU63
TU64
TU65
TU66
TU67
TU68
TU69
D082_00500
D082_00510
D082_00530
D082_00550
D082_00560
D082_0057
trmU
nplT
50000
52000
54000
56000
58000
60000
Untreated
15°C
42°C
−CO2
Dark
−Fe
HL
−N
−P
Exp. P.
Stat. P.

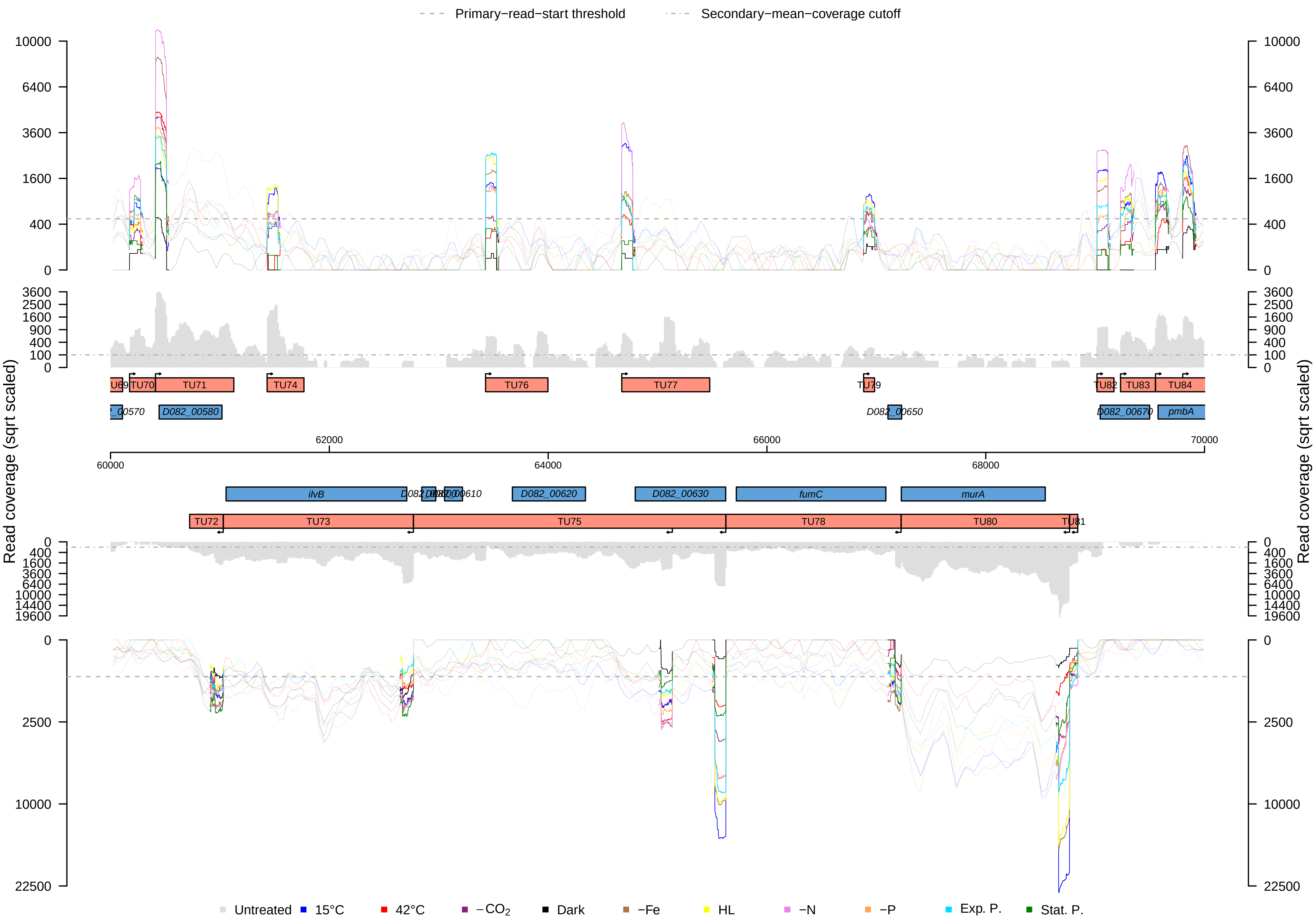

Primary−read−start threshold
Secondary−mean−coverage cutoff
Read coverage (sqrt scaled)
TU70
TU71
TU74
TU76
TU77
TU79
TU82
TU83
TU84
D082_00580
D082_00650
D082_00670
pmbA
ilvB
D082_00620
D082_00630
fumC
murA
TU72
TU73
TU75
TU78
TU80
60000
62000
64000
66000
68000
70000
Untreated
15°C
42°C
−CO2
Dark
−Fe
HL
−N
−P
Exp. P.
Stat. P.

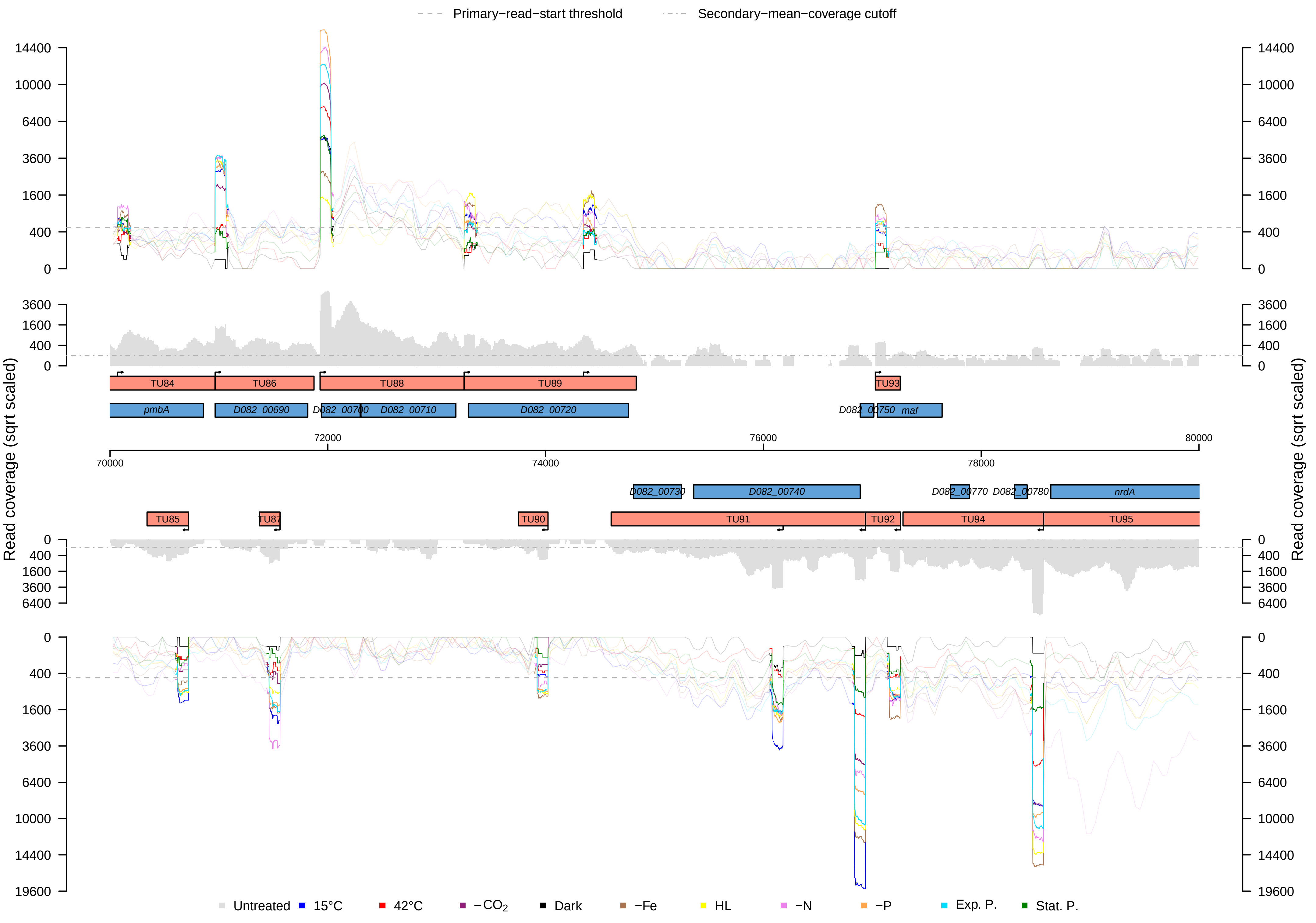

Primary−read−start threshold
Secondary−mean−coverage cutoff
Read coverage (sqrt scaled)
14400
10000
6400
3600
1600
400
0
TU84
TU86
TU88
TU89
TU93
pmbA
D082_00690
D082_00700
D082_00710
D082_00720
D082_00750
maf
70000
72000
74000
76000
78000
80000
D082_00730
D082_00740
D082_00770
D082_00780
nrdA
TU85
TU87
TU90
TU91
TU92
TU94
TU95
19600
Untreated
15°C
42°C
−CO2
Dark
−Fe
HL
−N
−P
Exp. P.
Stat. P.

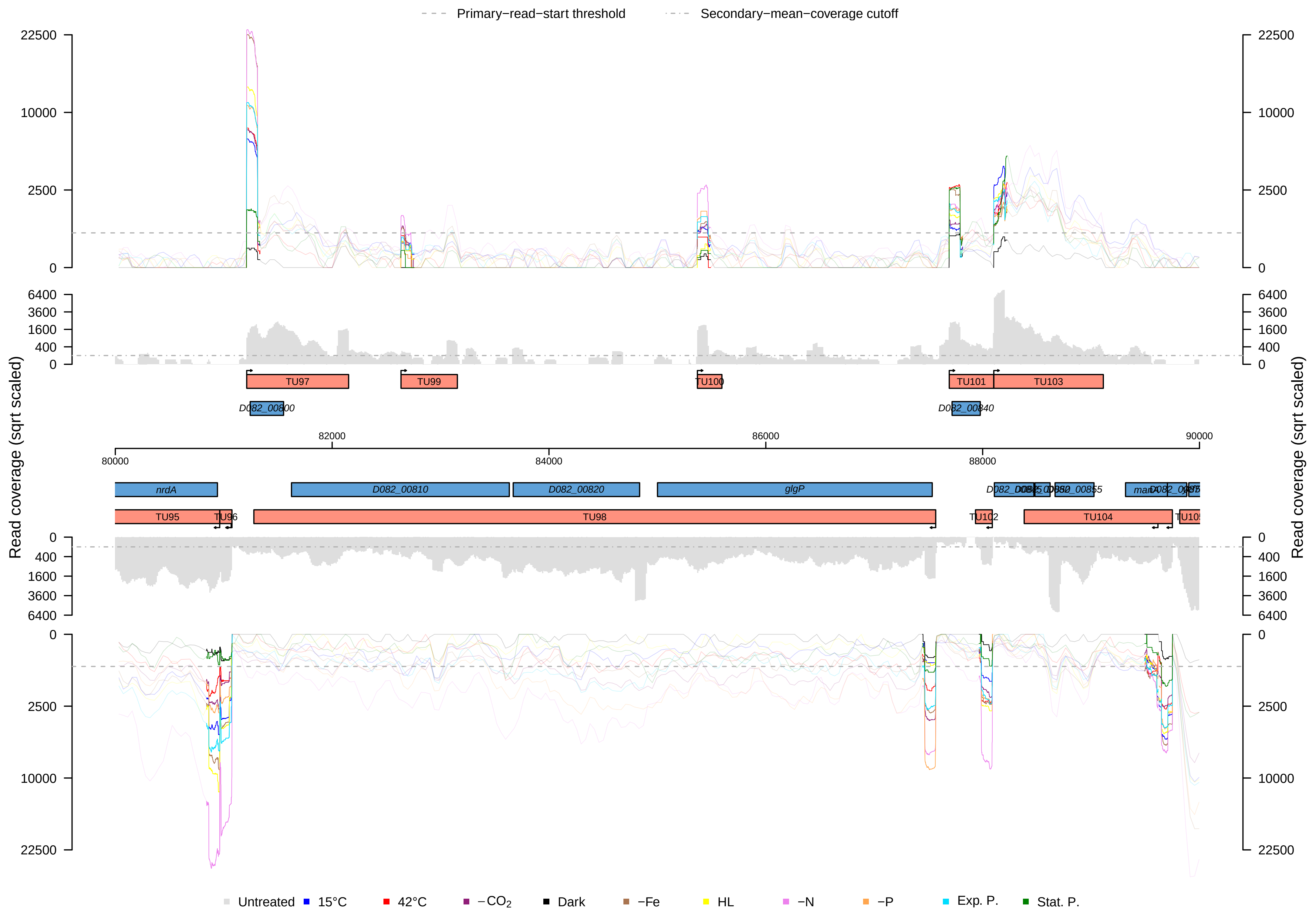

Primary−read−start threshold
Secondary−mean−coverage cutoff
Read coverage (sqrt scaled)
22500
10000
2500
0
6400
3600
1600
400
TU97
TU99
TU100
TU101
TU103
D082_00800
D082_00840
80000
82000
84000
86000
88000
90000
nrdA
D082_00810
D082_00820
glgP
TU95
TU96
TU98
TU102
TU104
Untreated
15°C
42°C
−CO_2
Dark
−Fe
HL
−N
−P
Exp. P.
Stat. P.

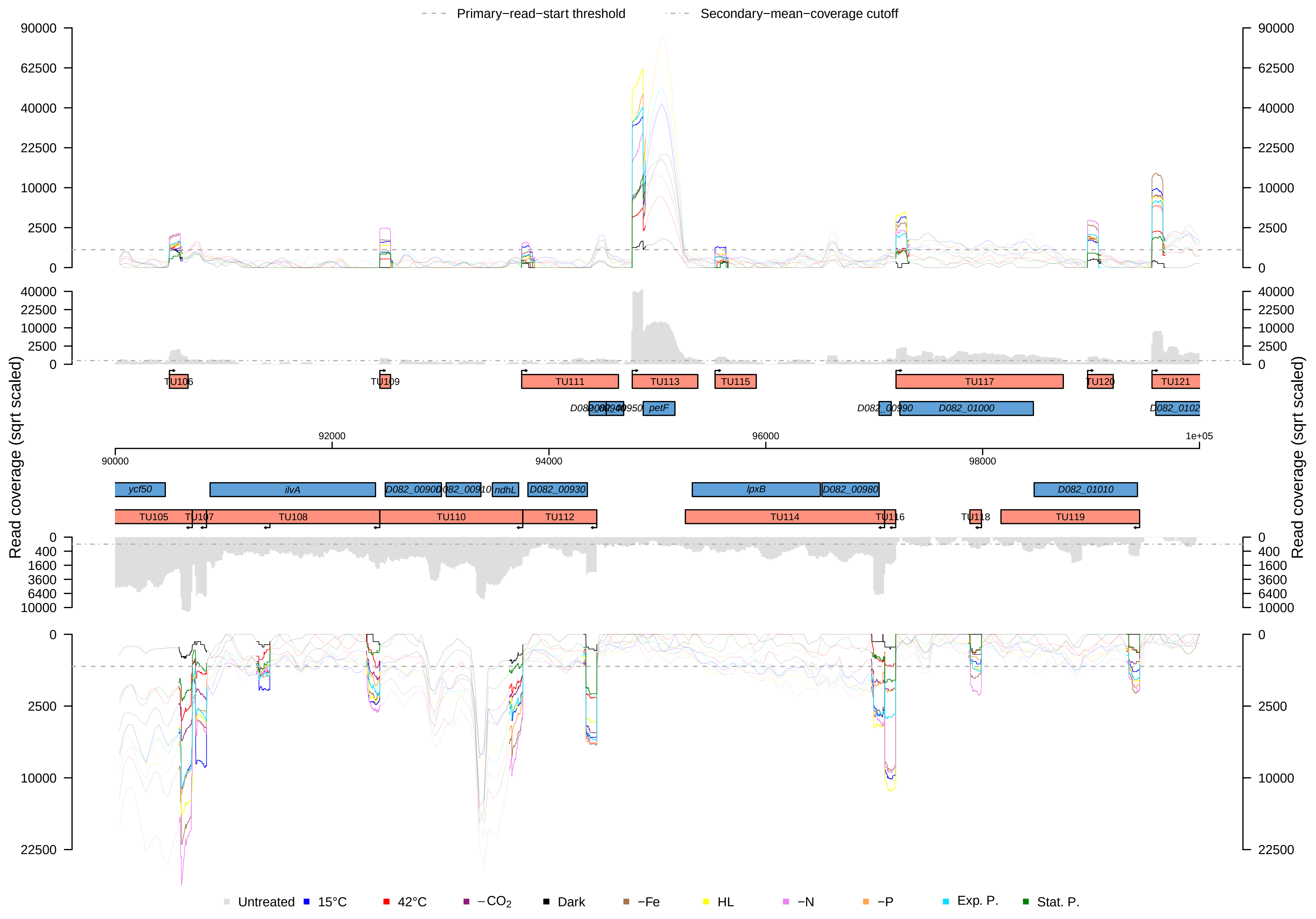

Primary−read−start threshold
Secondary−mean−coverage cutoff
Read coverage (sqrt scaled)
90000
62500
40000
22500
10000
2500
0
400
1600
3600
6400
TU106
TU109
TU111
TU113
TU115
TU117
TU120
TU121
petF
D082_01000
D082_00990
92000
94000
96000
98000
1e+05
90000
ycf50
ilvA
ndhL
D082_00930
lpxB
D082_00980
D082_01010
TU105
TU107
TU108
TU110
TU112
TU114
TU116
TU118
TU119
Untreated
15°C
42°C
−CO2
Dark
−Fe
HL
−N
−P
Exp. P.
Stat. P.

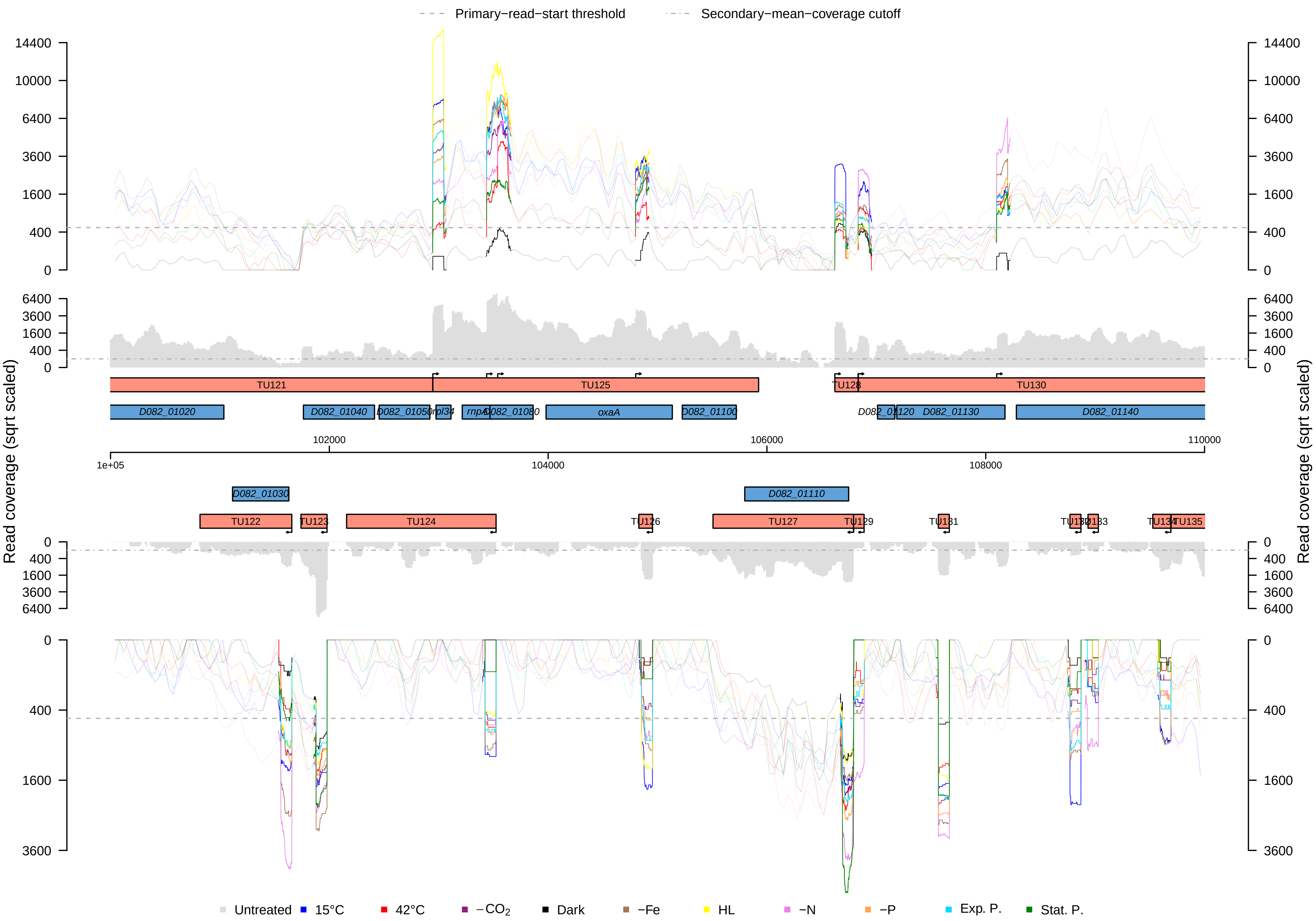

Primary−read−start threshold
Secondary−mean−coverage cutoff
Read coverage (sqrt scaled)
TU121
TU125
TU128
TU130
D082_01020
D082_01040
D082_01050
rpl34
rnpA
D082_01080
oxaA
D082_01100
D082_01120
D082_01130
D082_01140
1e+05
102000
104000
106000
108000
110000
D082_01030
D082_01110
TU122
TU123
TU124
TU126
TU127
TU129
TU131
TU133
TU134
TU135
Untreated
15°C
42°C
−CO_2
Dark
−Fe
HL
−N
−P
Exp. P.
Stat. P.

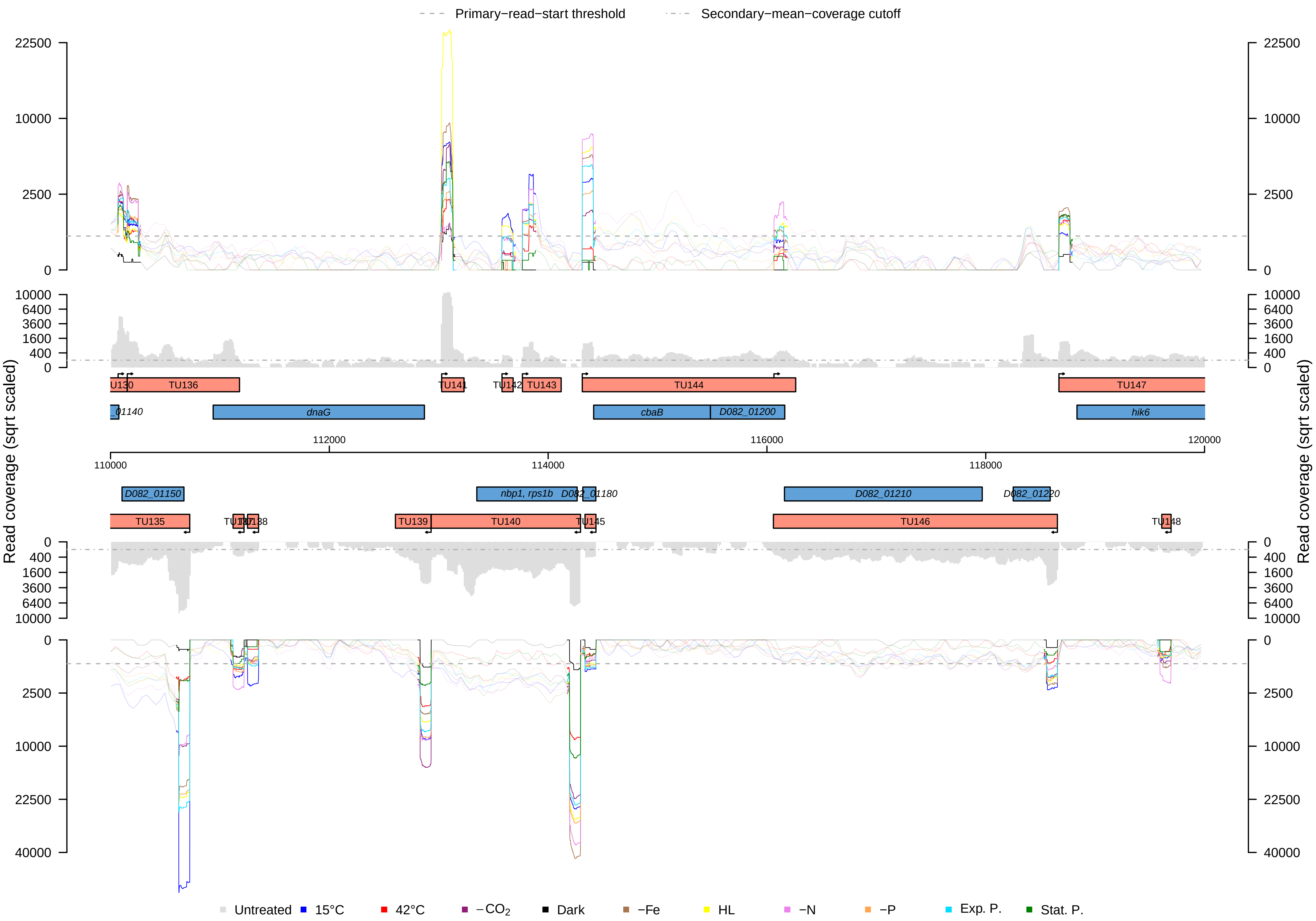

Primary−read−start threshold
Secondary−mean−coverage cutoff
22500
10000
2500
0
10000
6400
3600
1600
400
0
TU130
TU136
TU141
TU142
TU143
TU144
TU147
_01140
dnaG
cbaB
D082_01200
hik6
112000
116000
120000
110000
114000
118000
D082_01150
nbp1, rps1b
D082_01180
D082_01210
D082_01220
TU135
TU139
TU140
TU145
TU146
TU148
40000
Read coverage (sqrt scaled)
Untreated
15°C
42°C
−CO2
Dark
−Fe
HL
−N
−P
Exp. P.
Stat. P.

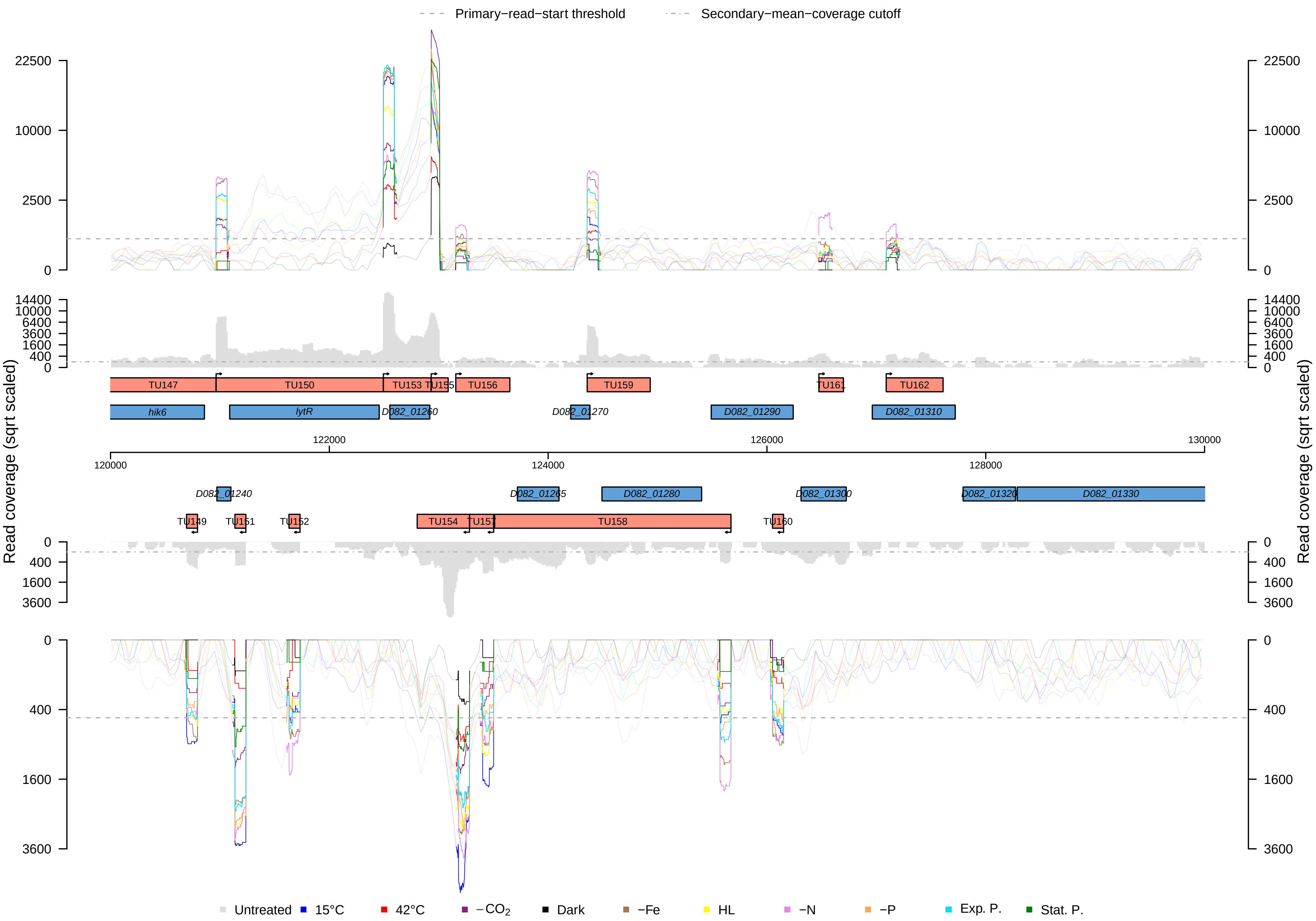

Primary−read−start threshold
Secondary−mean−coverage cutoff
22500
10000
2500
0
14400
10000
6400
3600
1600
400
Read coverage (sqrt scaled)
TU147
TU150
TU153
TU155
TU156
TU159
TU161
TU162
hik6
lytR
D082_01260
D082_01270
D082_01290
D082_01310
120000
122000
124000
126000
128000
130000
D082_01240
D082_01265
D082_01280
D082_01300
D082_01320
D082_01330
TU149
TU151
TU152
TU154
TU157
TU158
TU160
3600
Untreated
15°C
42°C
−CO2
Dark
−Fe
HL
−N
−P
Exp. P.
Stat. P.

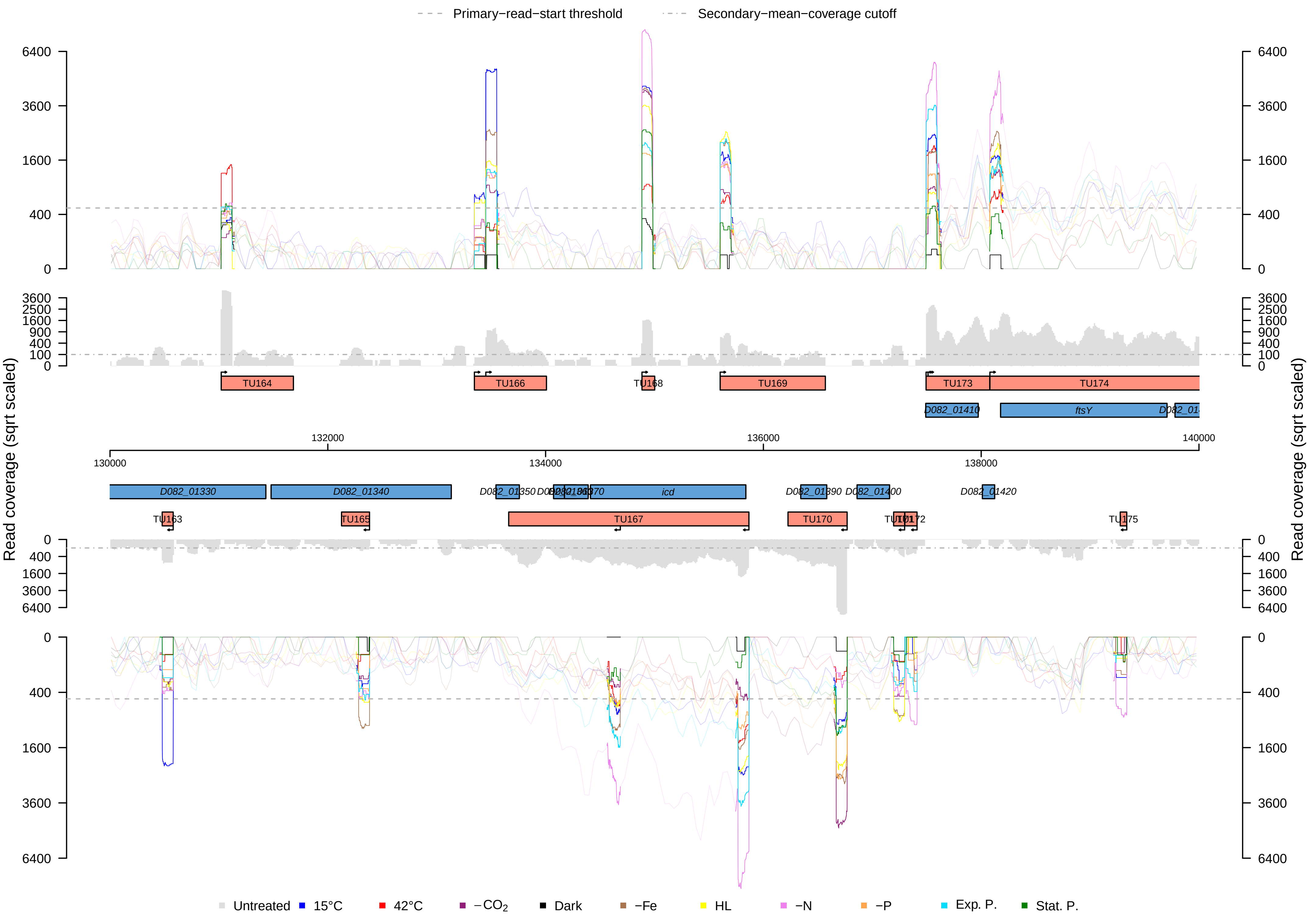

Primary−read−start threshold
Secondary−mean−coverage cutoff
Read coverage (sqrt scaled)
TU164
TU166
TU168
TU169
TU173
TU174
D082_01410
ftsY
D082_01330
D082_01340
D082_01350
icd
D082_01390
D082_01400
D082_01420
TU163
TU165
TU167
TU170
TU175
Untreated
15°C
42°C
−CO2
Dark
−Fe
HL
−N
−P
Exp. P.
Stat. P.

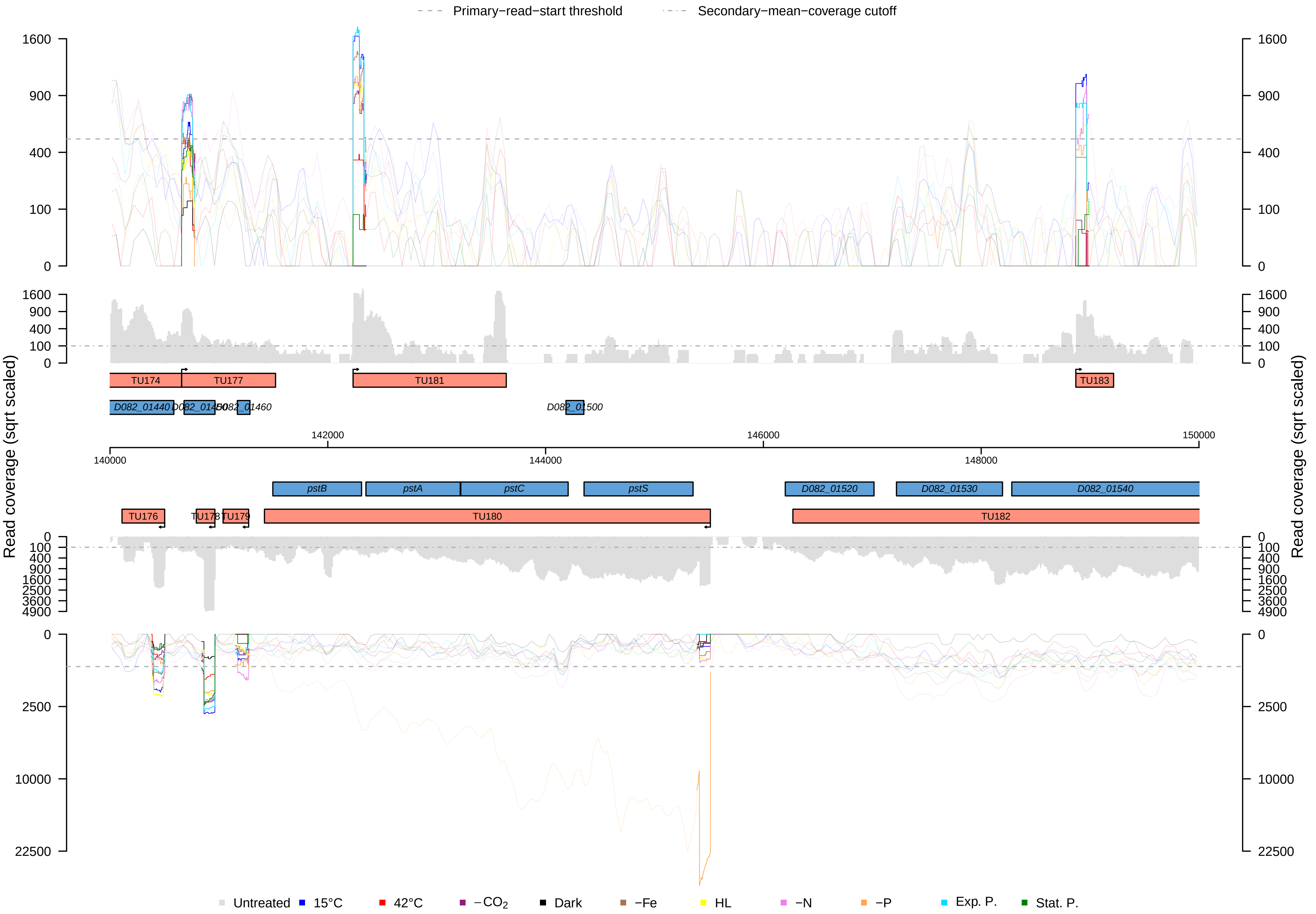

Primary−read−start threshold
Secondary−mean−coverage cutoff
Read coverage (sqrt scaled)
Read coverage (sqrt scaled)
1600
900
400
100
0
2500
3600
4900
10000
22500
TU174
TU177
TU181
TU183
D082_01440
D082_01450
D082_01460
D082_01500
140000
142000
144000
146000
148000
150000
pstB
pstA
pstC
pstS
D082_01520
D082_01530
D082_01540
TU176
TU178
TU179
TU180
TU182
Untreated
15°C
42°C
−CO_2
Dark
−Fe
HL
−N
−P
Exp. P.
Stat. P.

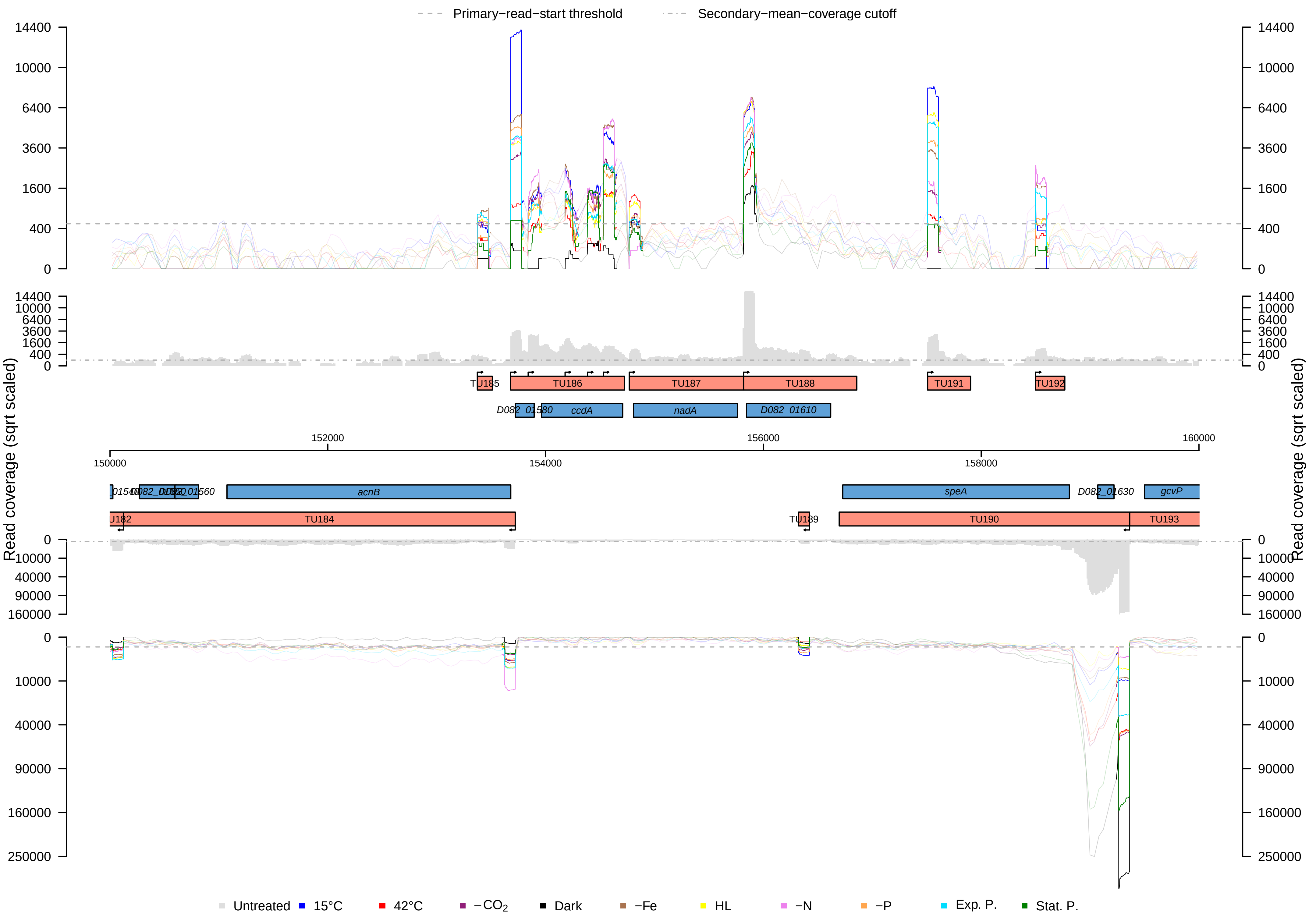

Primary−read−start threshold
Secondary−mean−coverage cutoff
Read coverage (sqrt scaled)
14400
10000
6400
3600
1600
400
0
TU185
TU186
TU187
TU188
TU191
TU192
D082_01580
ccdA
nadA
D082_01610
150000
152000
154000
156000
158000
160000
acnB
speA
D082_01630
gcvP
TU184
TU189
TU190
TU193
10000
40000
90000
160000
250000
Untreated
15°C
42°C
−CO2
Dark
−Fe
HL
−N
−P
Exp. P.
Stat. P.

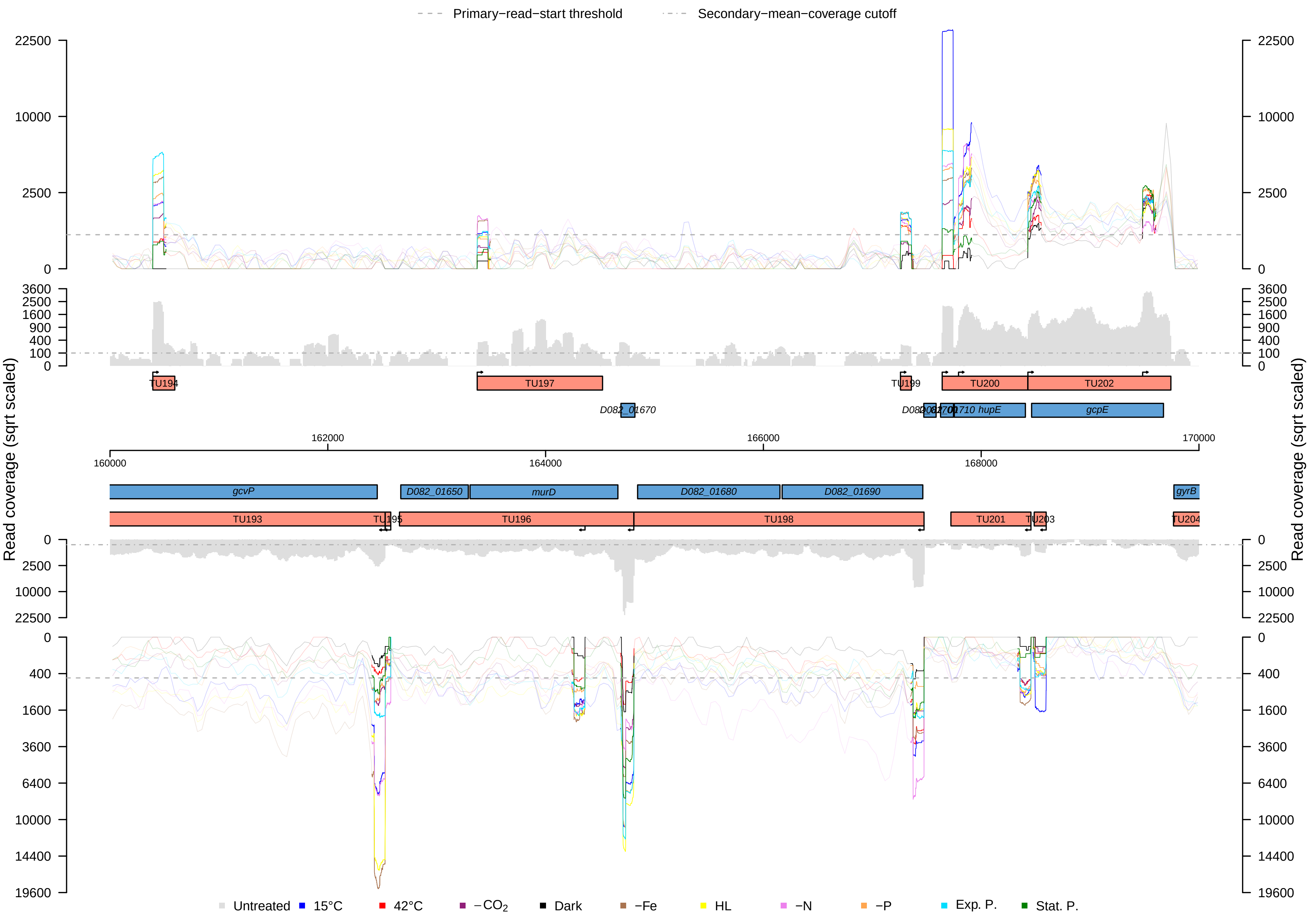

Primary−read−start threshold
Secondary−mean−coverage cutoff
Read coverage (sqrt scaled)
Read coverage (sqrt scaled)
TU194
TU197
TU199
TU200
TU202
D082_01670
gcpE
160000
162000
164000
166000
168000
170000
gcvP
D082_01650
murD
D082_01680
D082_01690
gyrB
TU193
TU195
TU196
TU198
TU201
TU203
TU204
Untreated
15°C
42°C
−CO2
Dark
−Fe
HL
−N
−P
Exp. P.
Stat. P.

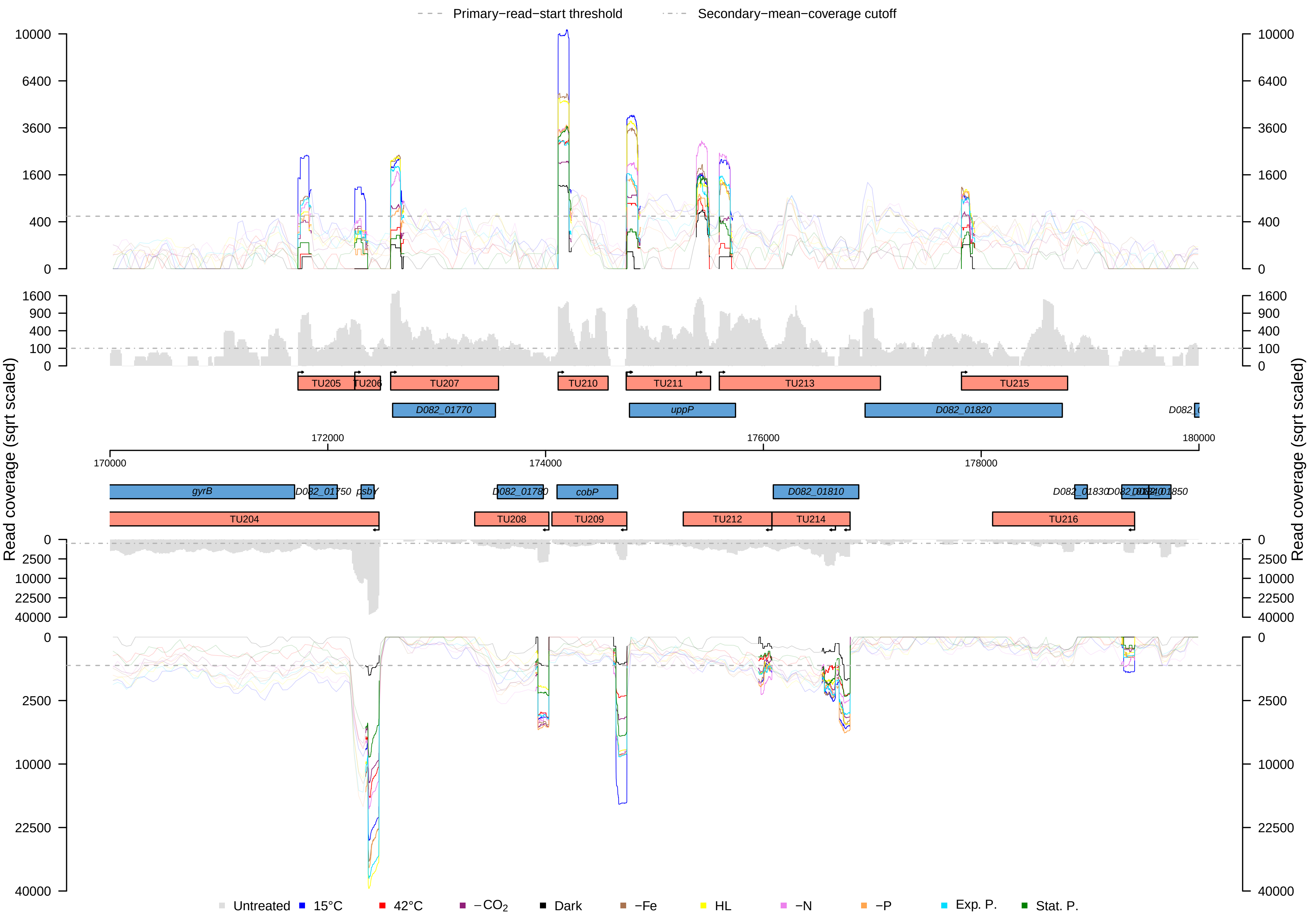

Primary−read−start threshold
Secondary−mean−coverage cutoff
Read coverage (sqrt scaled)
10000
6400
3600
1600
400
0
900
100
2500
22500
40000
TU204
TU205
TU206
TU207
TU208
TU209
TU210
TU211
TU212
TU213
TU214
TU215
TU216
gyrB
D082_01750
psbY
D082_01770
D082_01780
cobP
uppP
D082_01810
D082_01820
D082_01830
D082_01850
170000
172000
174000
176000
178000
180000
Untreated
15°C
42°C
−CO2
Dark
−Fe
HL
−N
−P
Exp. P.
Stat. P.

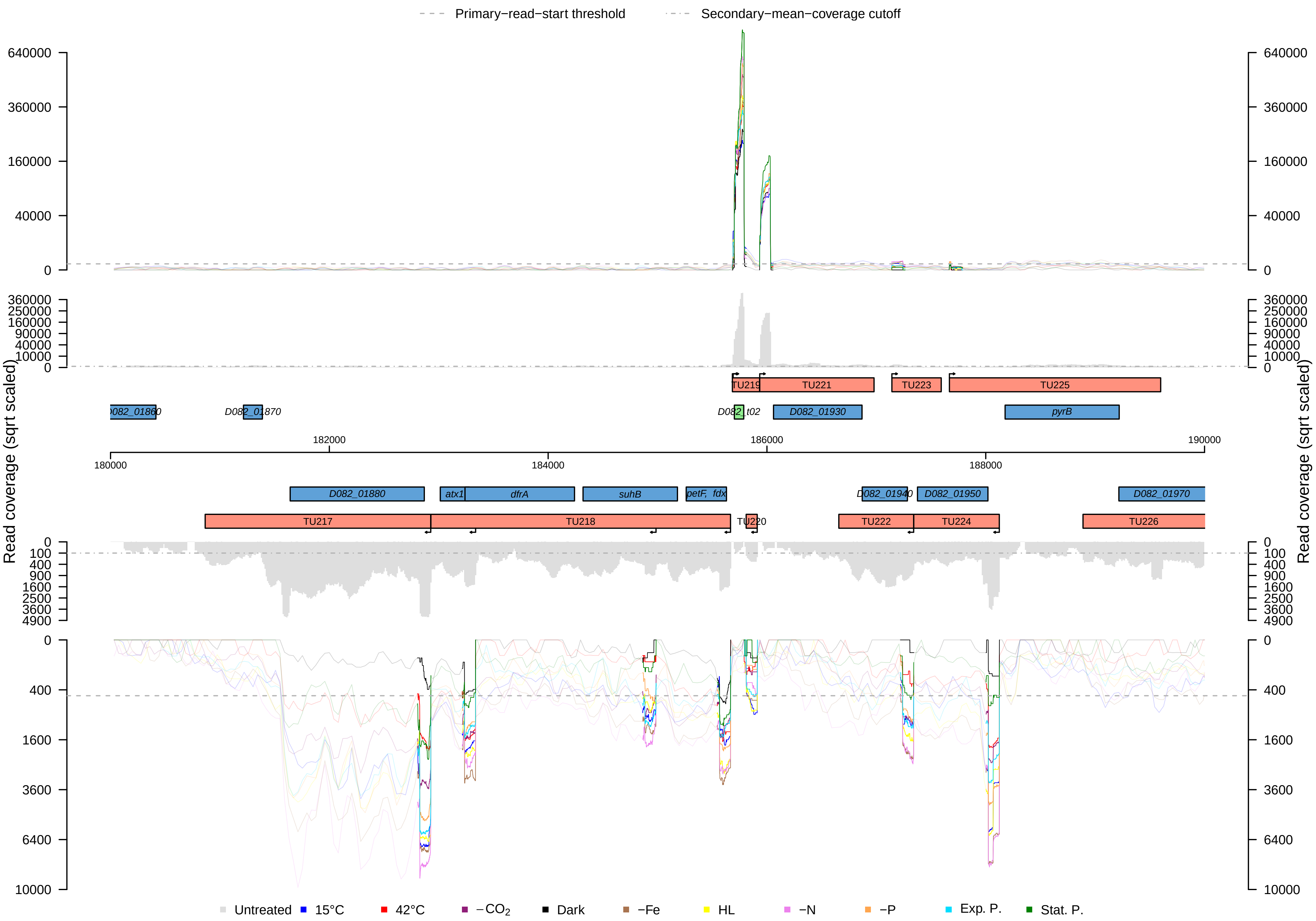

Primary−read−start threshold
Secondary−mean−coverage cutoff
Read coverage (sqrt scaled)
TU219
TU221
TU223
TU225
D082_01870
D082_t02
D082_01930
pyrB
D082_01880
atx1
dfrA
suhB
petF, fdx
D082_01950
D082_01970
TU217
TU218
TU222
TU224
TU226
Untreated
15°C
42°C
−CO2
Dark
−Fe
HL
−N
−P
Exp. P.
Stat. P.

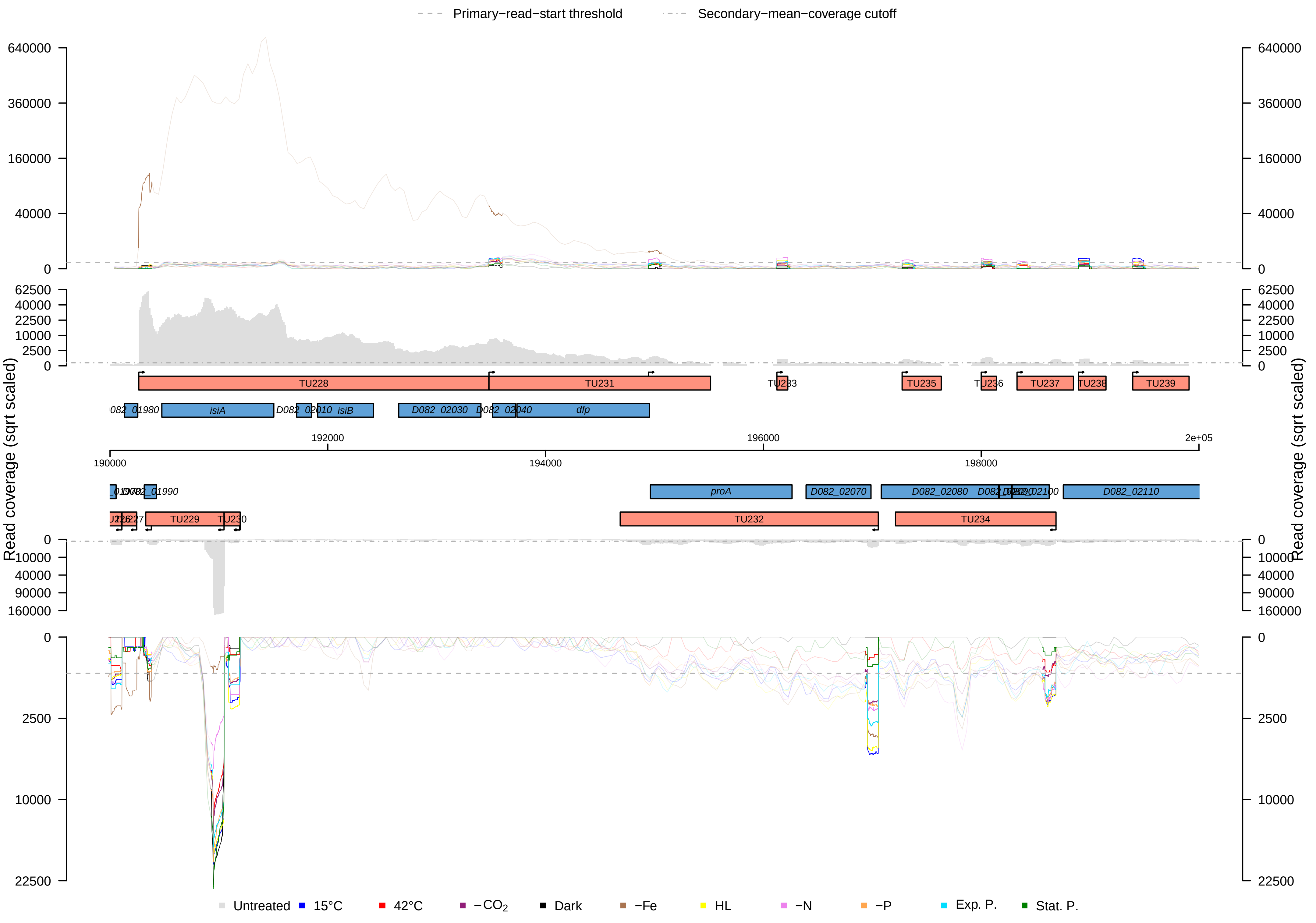

Primary−read−start threshold
Secondary−mean−coverage cutoff
Read coverage (sqrt scaled)
640000
360000
160000
40000
0
62500
40000
22500
10000
2500
0
TU228
TU231
TU233
TU235
TU236
TU237
TU238
TU239
isiA
isiB
D082_02030
dfp
192000
196000
2e+05
190000
194000
198000
proA
D082_02070
D082_02080
D082_02110
TU229
TU230
TU232
TU234
10000
90000
160000
2500
22500
Untreated
15°C
42°C
−CO2
Dark
−Fe
HL
−N
−P
Exp. P.
Stat. P.

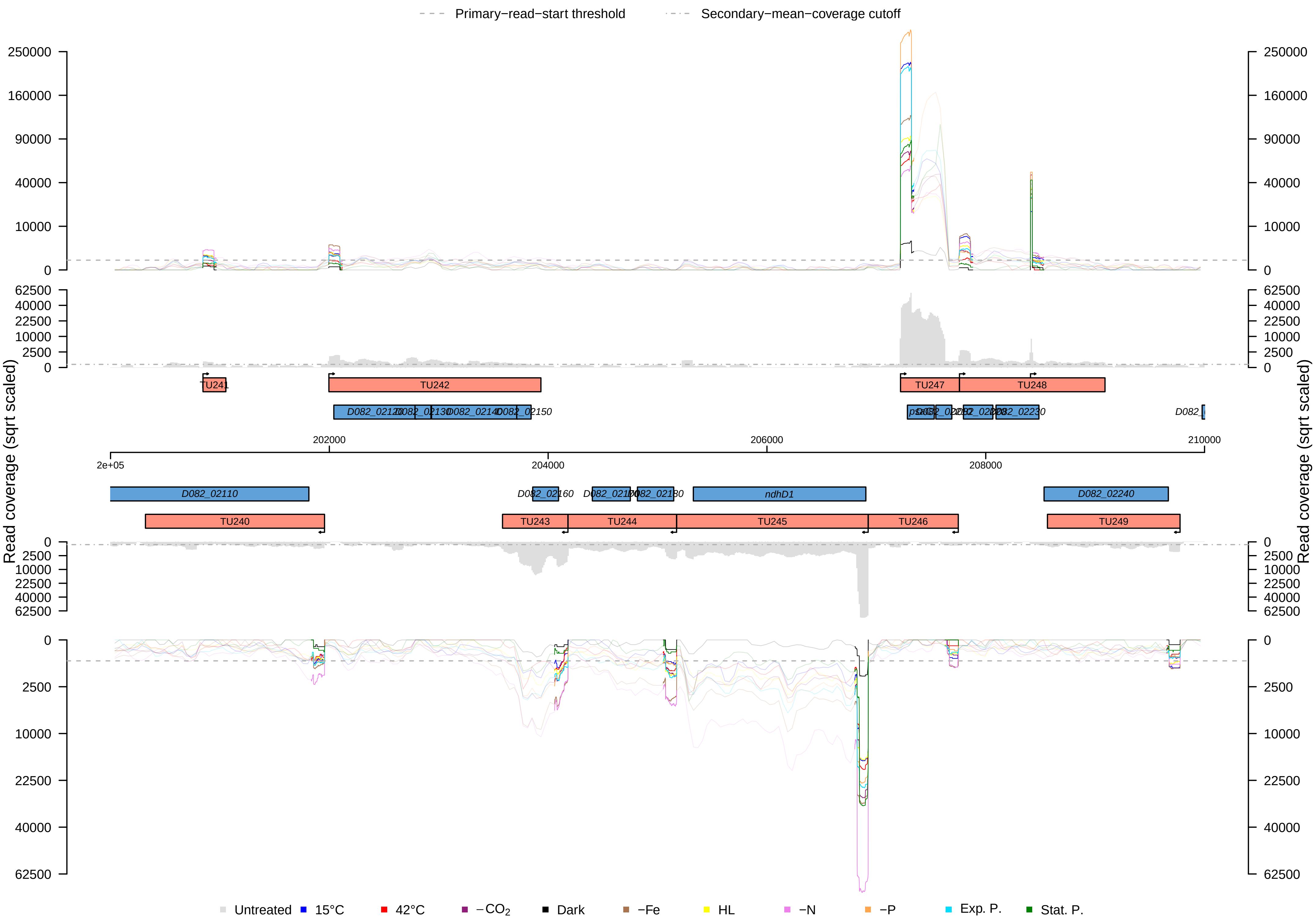

Primary−read−start threshold
Secondary−mean−coverage cutoff
Read coverage (sqrt scaled)
250000
160000
90000
40000
10000
0
62500
40000
22500
10000
2500
0
TU241
TU242
TU247
TU248
D082_02120
D082_02130
D082_02140
D082_02150
D082_02230
2e+05
202000
204000
206000
208000
210000
D082_02110
D082_02160
D082_02180
ndhD1
D082_02240
TU240
TU243
TU244
TU245
TU246
TU249
Untreated
15°C
42°C
−CO2
Dark
−Fe
HL
−N
−P
Exp. P.
Stat. P.

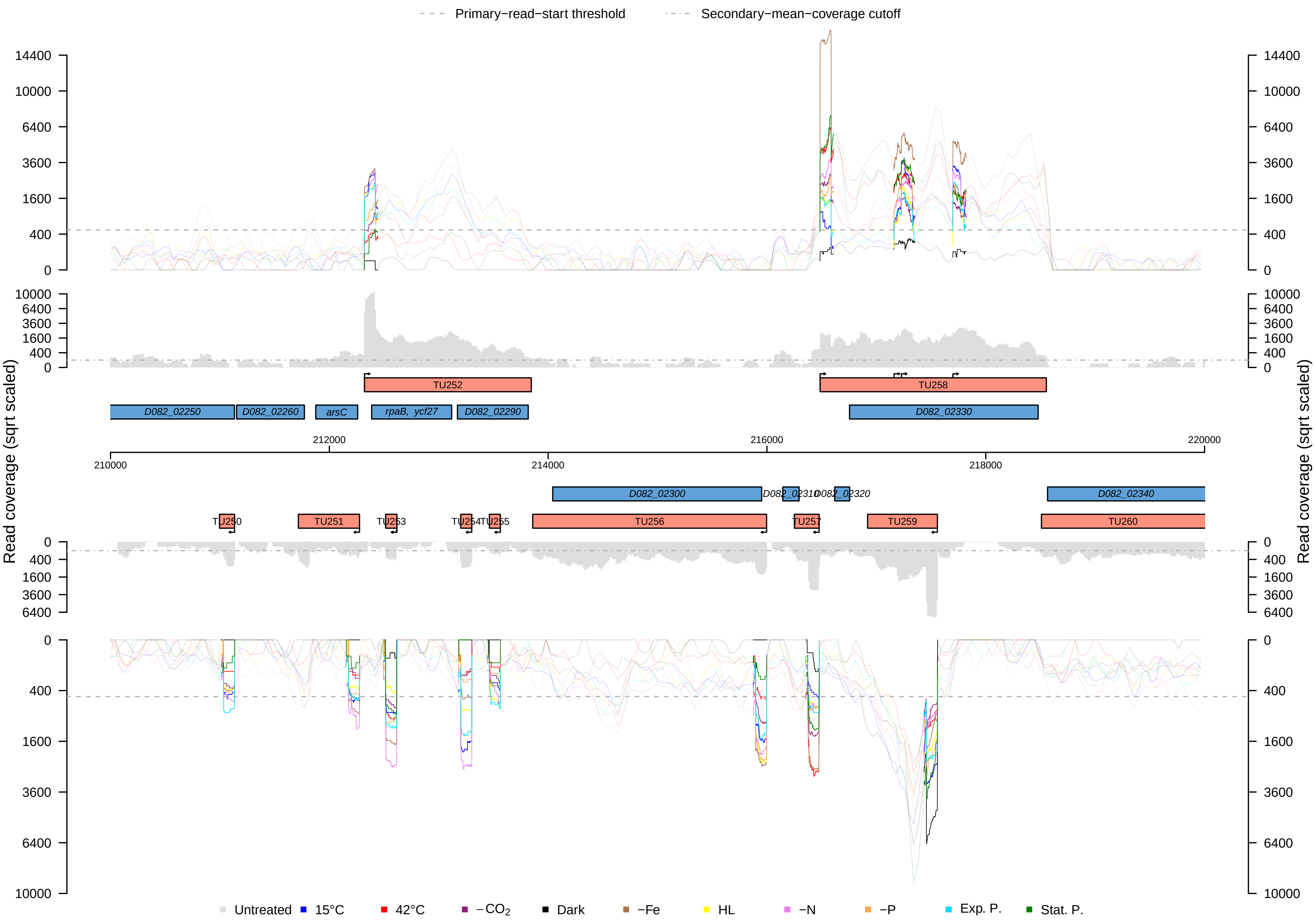

Primary−read−start threshold
Secondary−mean−coverage cutoff
Read coverage (sqrt scaled)
14400
10000
6400
3600
1600
400
0
TU252
TU258
D082_02250
D082_02260
arsC
rpaB, ycf27
D082_02290
D082_02330
210000
212000
214000
216000
218000
220000
D082_02300
D082_02310
D082_02320
D082_02340
TU250
TU251
TU253
TU254
TU255
TU256
TU257
TU259
TU260
Untreated
15°C
42°C
−CO2
Dark
−Fe
HL
−N
−P
Exp. P.
Stat. P.

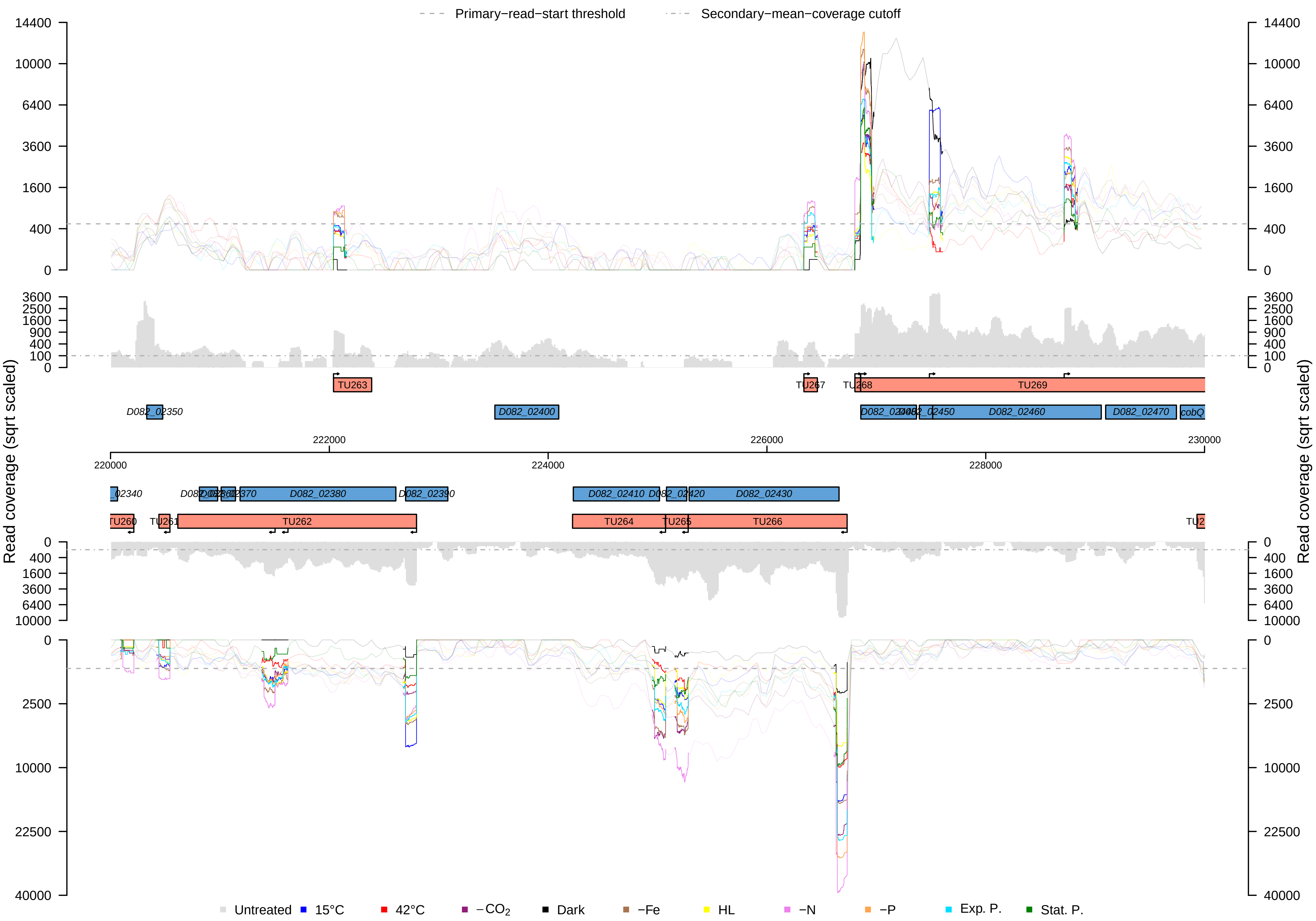

Primary−read−start threshold
Secondary−mean−coverage cutoff
Read coverage (sqrt scaled)
14400
10000
6400
3600
1600
400
0
2500
900
100
22500
40000
TU263
TU267
TU268
TU269
D082_02350
D082_02400
D082_02460
D082_02470
cobQ
220000
222000
224000
226000
228000
230000
D082_02380
D082_02390
D082_02410
D082_02430
TU260
TU261
TU262
TU264
TU265
TU266
Untreated
15°C
42°C
−CO2
Dark
−Fe
HL
−N
−P
Exp. P.
Stat. P.

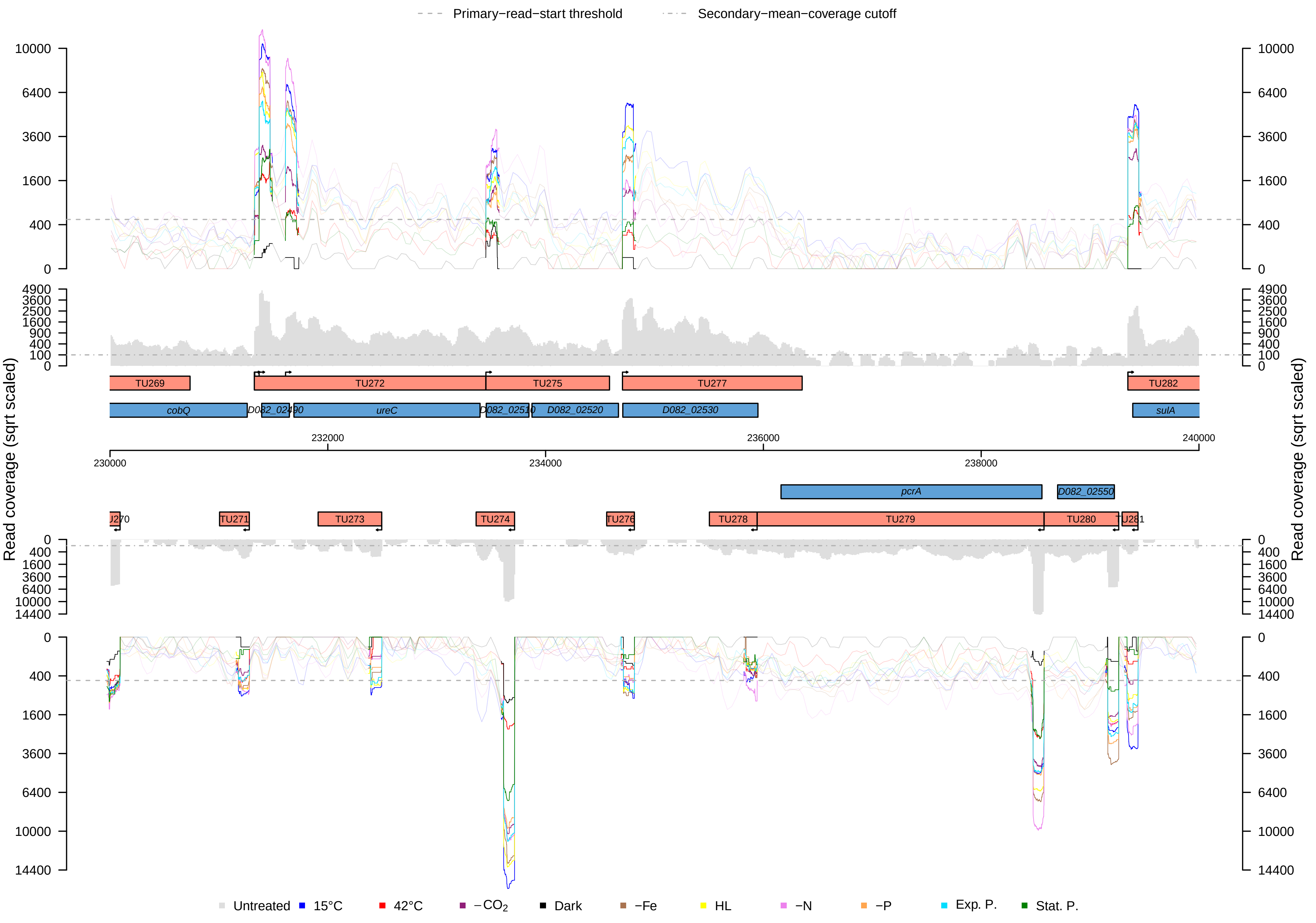

Primary−read−start threshold
Secondary−mean−coverage cutoff
Read coverage (sqrt scaled)
TU269
TU272
TU275
TU277
TU282
cobQ
D082_02490
ureC
D082_02510
D082_02520
D082_02530
sulA
230000
232000
234000
236000
238000
240000
pcrA
D082_02550
TU271
TU273
TU274
TU276
TU278
TU279
TU280
Untreated
15°C
42°C
−CO2
Dark
−Fe
HL
−N
−P
Exp. P.
Stat. P.

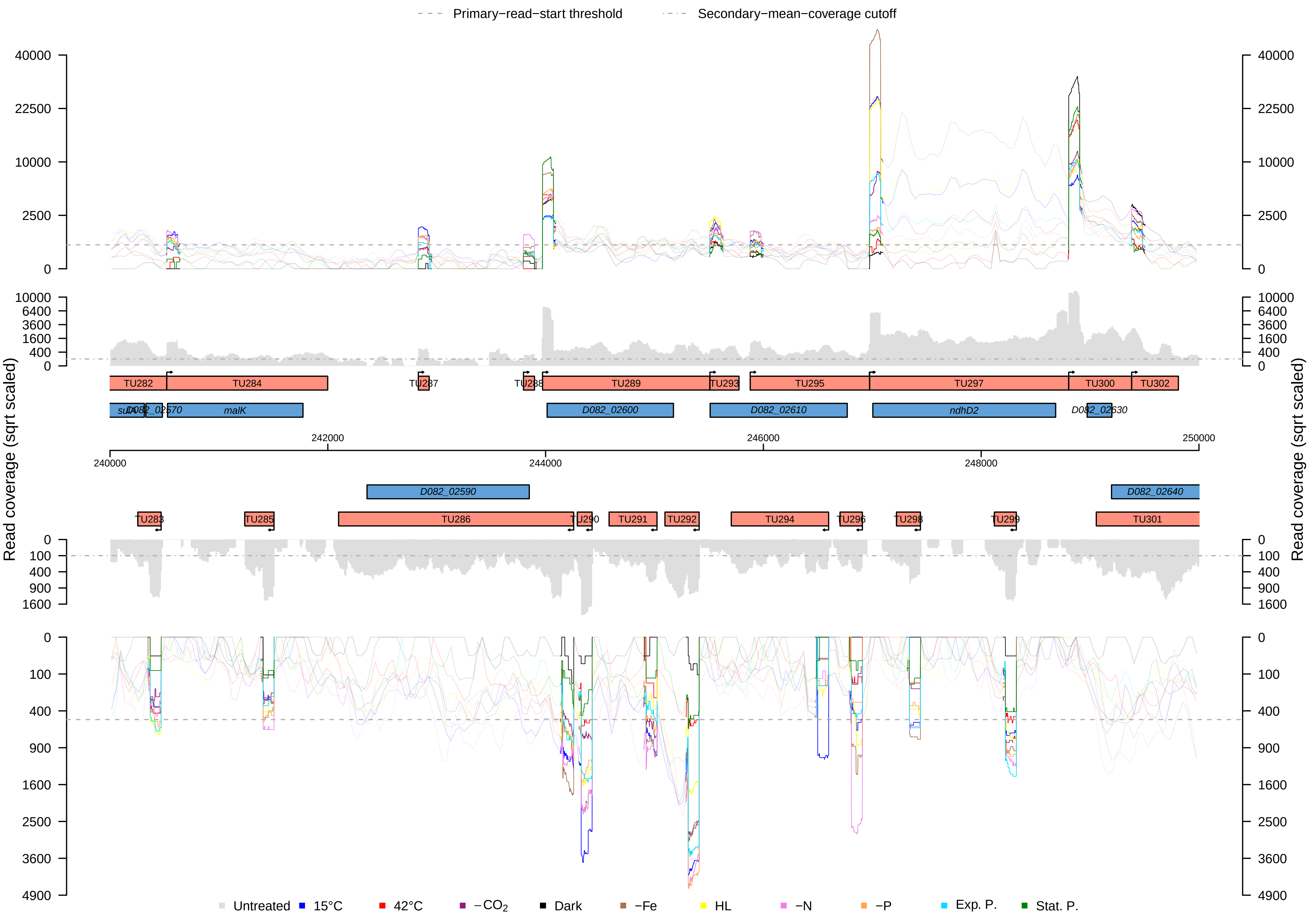

Primary−read−start threshold
Secondary−mean−coverage cutoff
Read coverage (sqrt scaled)
40000
22500
10000
2500
0
10000
6400
3600
1600
400
0
TU282
TU284
TU287
TU288
TU289
TU293
TU295
TU297
TU300
TU302
D082_02570
malK
D082_02600
D082_02610
ndhD2
D082_02630
240000
242000
244000
246000
248000
250000
D082_02590
D082_02640
TU283
TU285
TU286
TU290
TU291
TU292
TU294
TU296
TU298
TU299
TU301
100
900
1600
2500
3600
4900
Untreated
15°C
42°C
−CO2
Dark
−Fe
HL
−N
−P
Exp. P.
Stat. P.

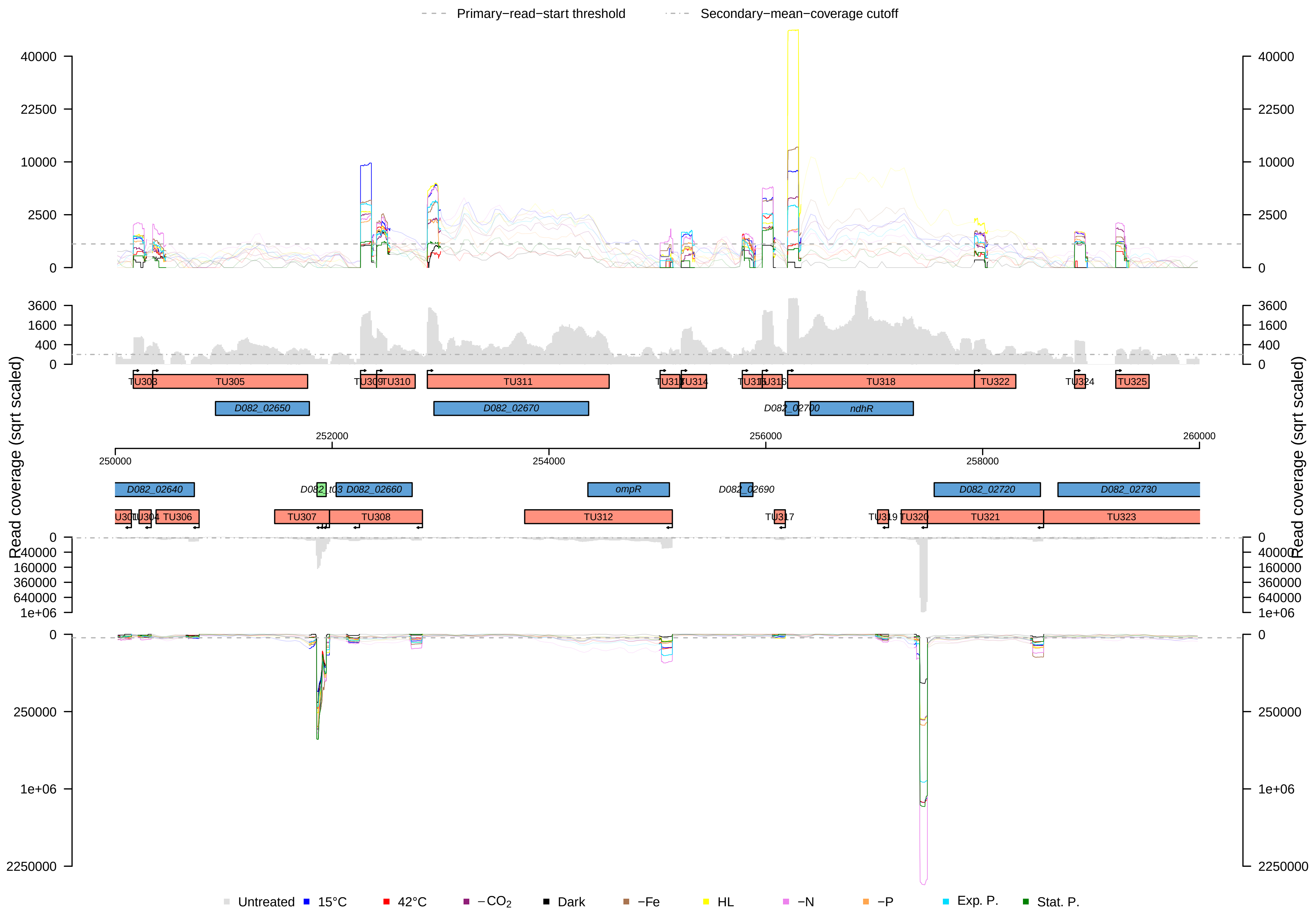

Primary−read−start threshold
Secondary−mean−coverage cutoff
Read coverage (sqrt scaled)
40000
22500
10000
2500
0
3600
1600
400
TU303
TU305
TU309
TU310
TU311
TU314
TU316
TU318
TU322
TU324
TU325
D082_02650
D082_02670
D082_02700
ndhR
250000
252000
254000
256000
258000
260000
D082_02640
D082_02660
ompR
D082_02690
D082_02720
D082_02730
TU304
TU306
TU307
TU308
TU312
TU317
TU319
TU320
TU321
TU323
160000
360000
640000
1e+06
250000
2250000
Untreated
15°C
42°C
−CO2
Dark
−Fe
HL
−N
−P
Exp. P.
Stat. P.

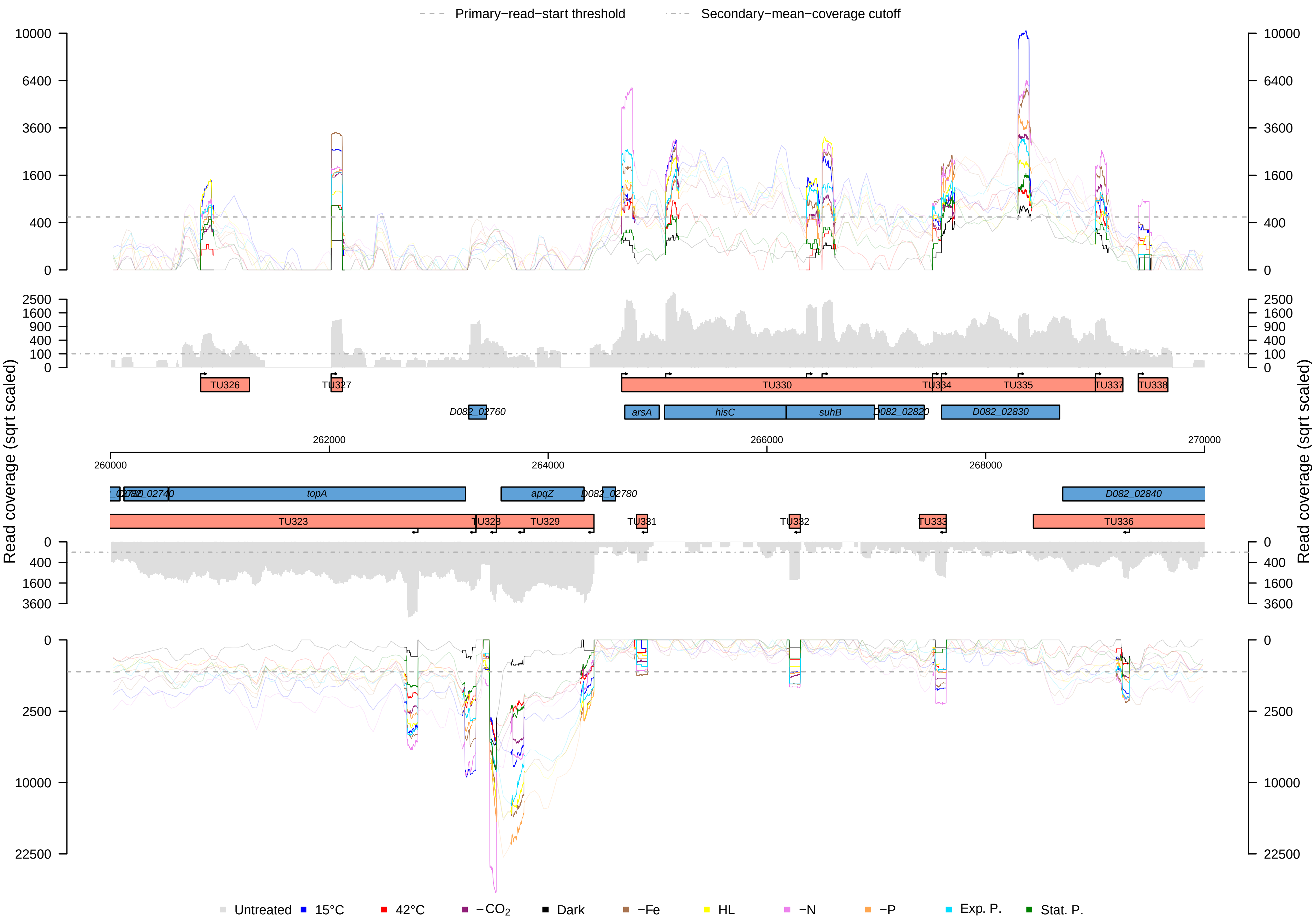

Primary−read−start threshold
Secondary−mean−coverage cutoff
Read coverage (sqrt scaled)
10000
6400
3600
1600
400
0
2500
1600
900
400
100
0
TU326
TU327
TU330
TU334
TU335
TU337
TU338
D082_02760
arsA
hisC
suhB
D082_02820
D082_02830
262000
266000
270000
260000
264000
268000
topA
apqZ
D082_02740
D082_02780
D082_02840
TU323
TU328
TU329
TU331
TU332
TU333
TU336
400
1600
3600
2500
10000
22500
Untreated
15°C
42°C
−CO_2
Dark
−Fe
HL
−N
−P
Exp. P.
Stat. P.

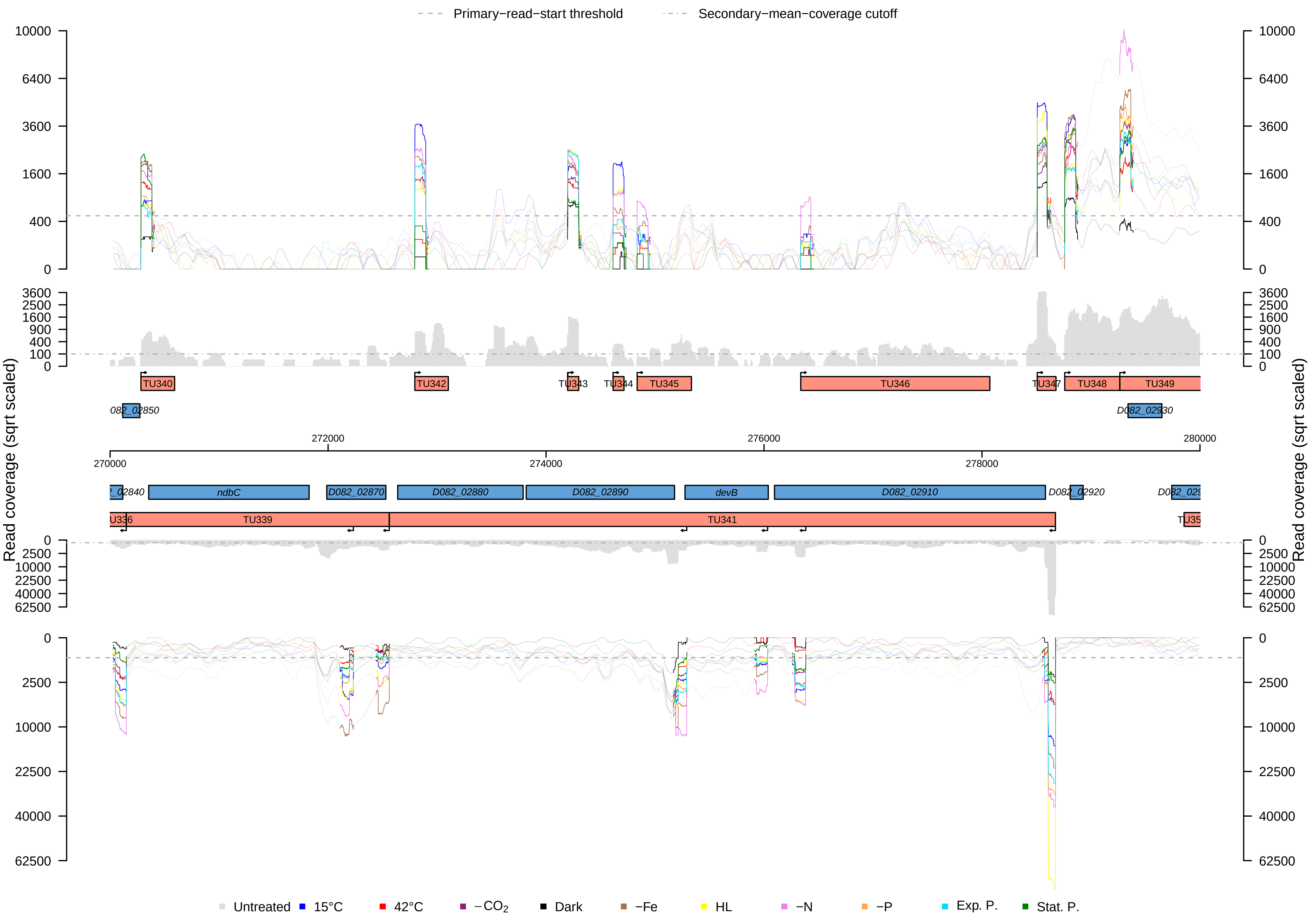

Primary−read−start threshold
Secondary−mean−coverage cutoff
Read coverage (sqrt scaled)
TU340
TU342
TU343
TU344
TU345
TU346
TU347
TU348
TU349
D082_02930
ndbC
D082_02870
D082_02880
D082_02890
devB
D082_02910
TU339
TU341
270000
272000
274000
276000
278000
280000
Untreated
15°C
42°C
−CO2
Dark
−Fe
HL
−N
−P
Exp. P.
Stat. P.

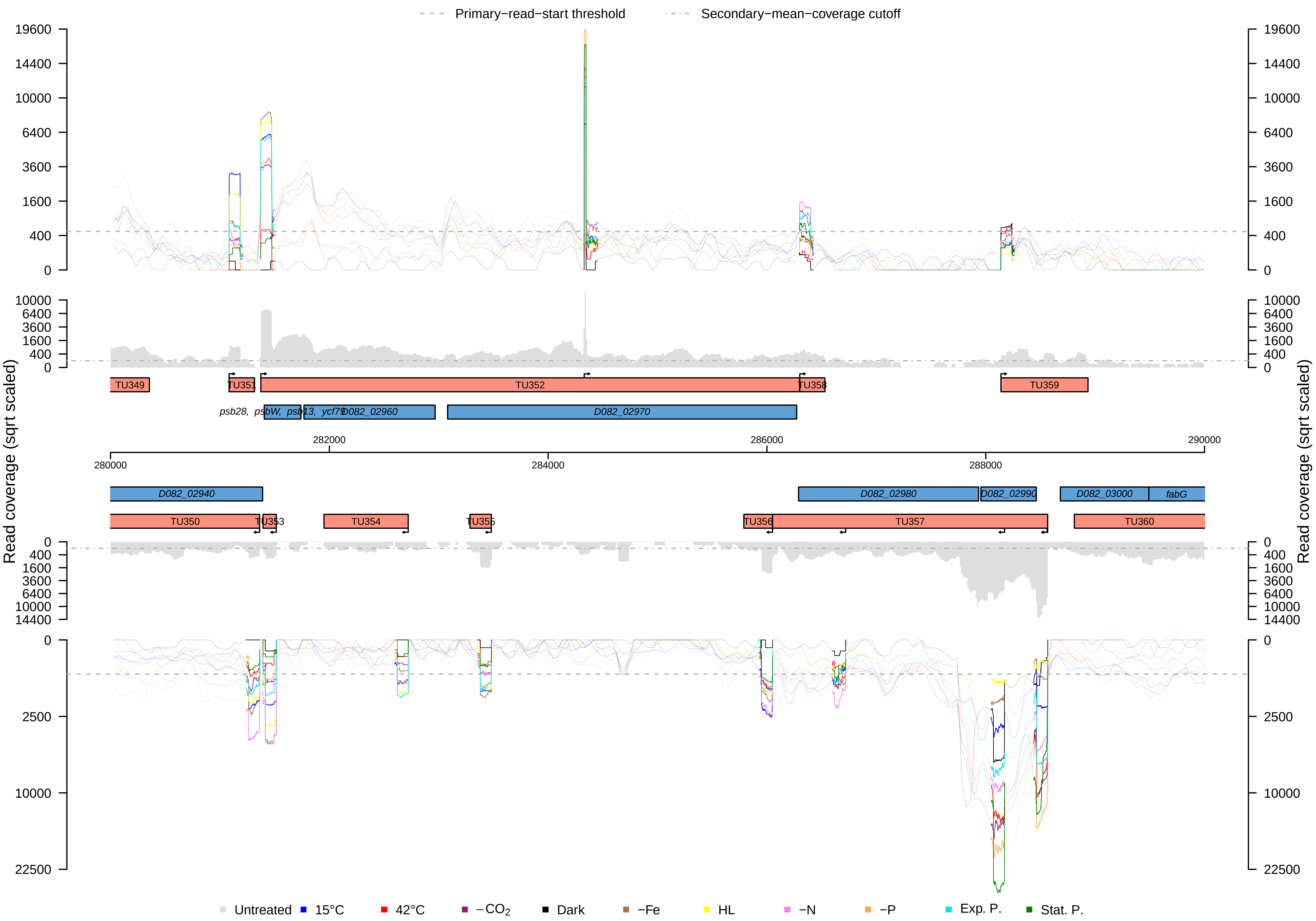

Primary−read−start threshold
Secondary−mean−coverage cutoff
Read coverage (sqrt scaled)
19600
14400
10000
6400
3600
1600
400
0
TU349
TU351
TU352
TU358
TU359
psb28, psbW, psb13, ycf79
D082_02960
D082_02970
280000
282000
284000
286000
288000
290000
D082_02940
D082_02980
D082_02990
D082_03000
fabG
TU350
TU353
TU354
TU355
TU356
TU357
TU360
2500
10000
22500
Untreated
15°C
42°C
−CO2
Dark
−Fe
HL
−N
−P
Exp. P.
Stat. P.

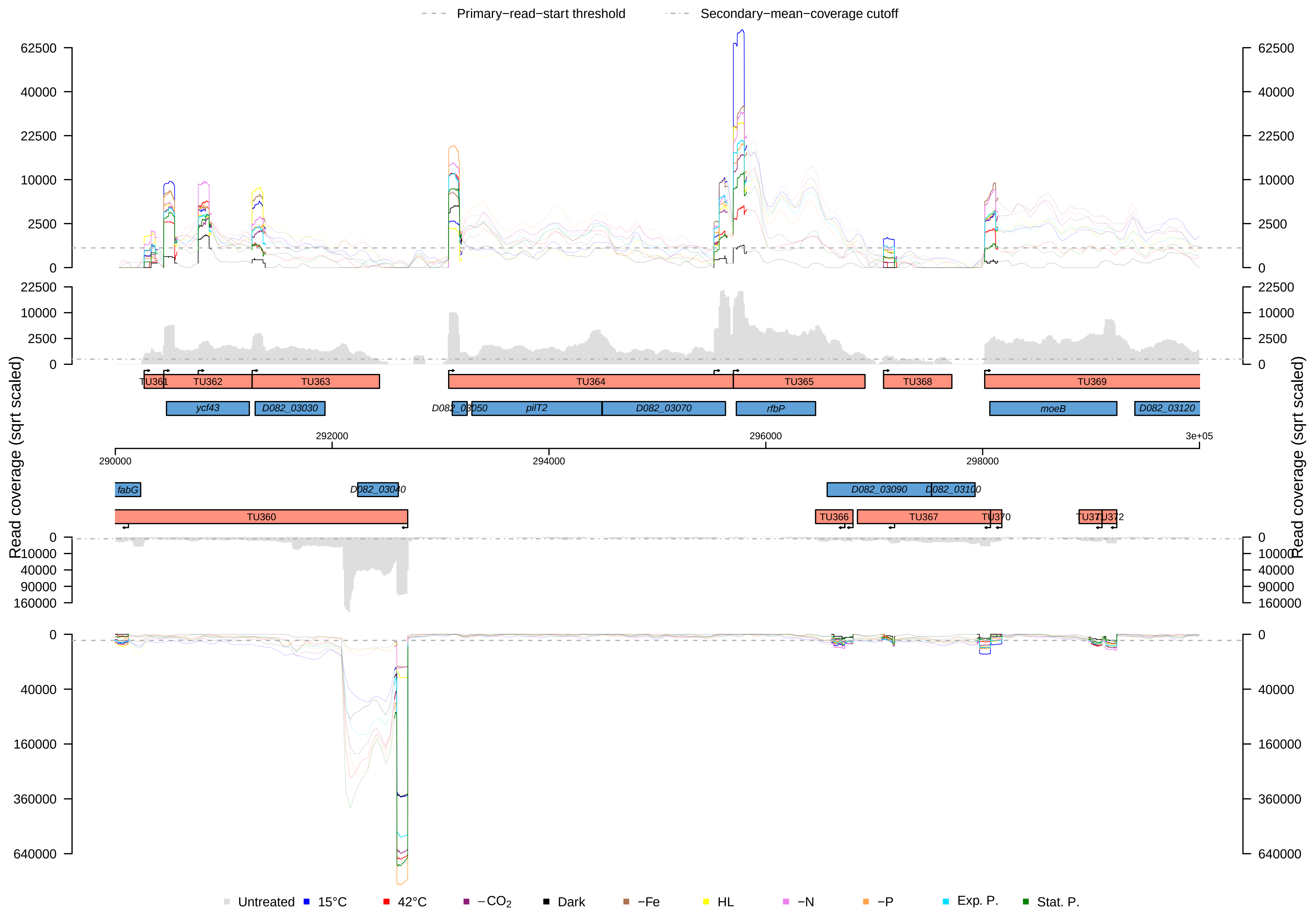

Primary−read−start threshold
Secondary−mean−coverage cutoff
Read coverage (sqrt scaled)
62500
40000
22500
10000
2500
0
TU361
TU362
TU363
TU364
TU365
TU368
TU369
ycf43
D082_03030
D082_03050
pilT2
D082_03070
rfbP
moeB
D082_03120
290000
292000
294000
296000
298000
3e+05
fabG
D082_03040
D082_03090
D082_03100
TU360
TU366
TU367
TU370
TU371
TU372
90000
160000
360000
640000
Untreated
15°C
42°C
−CO2
Dark
−Fe
HL
−N
−P
Exp. P.
Stat. P.

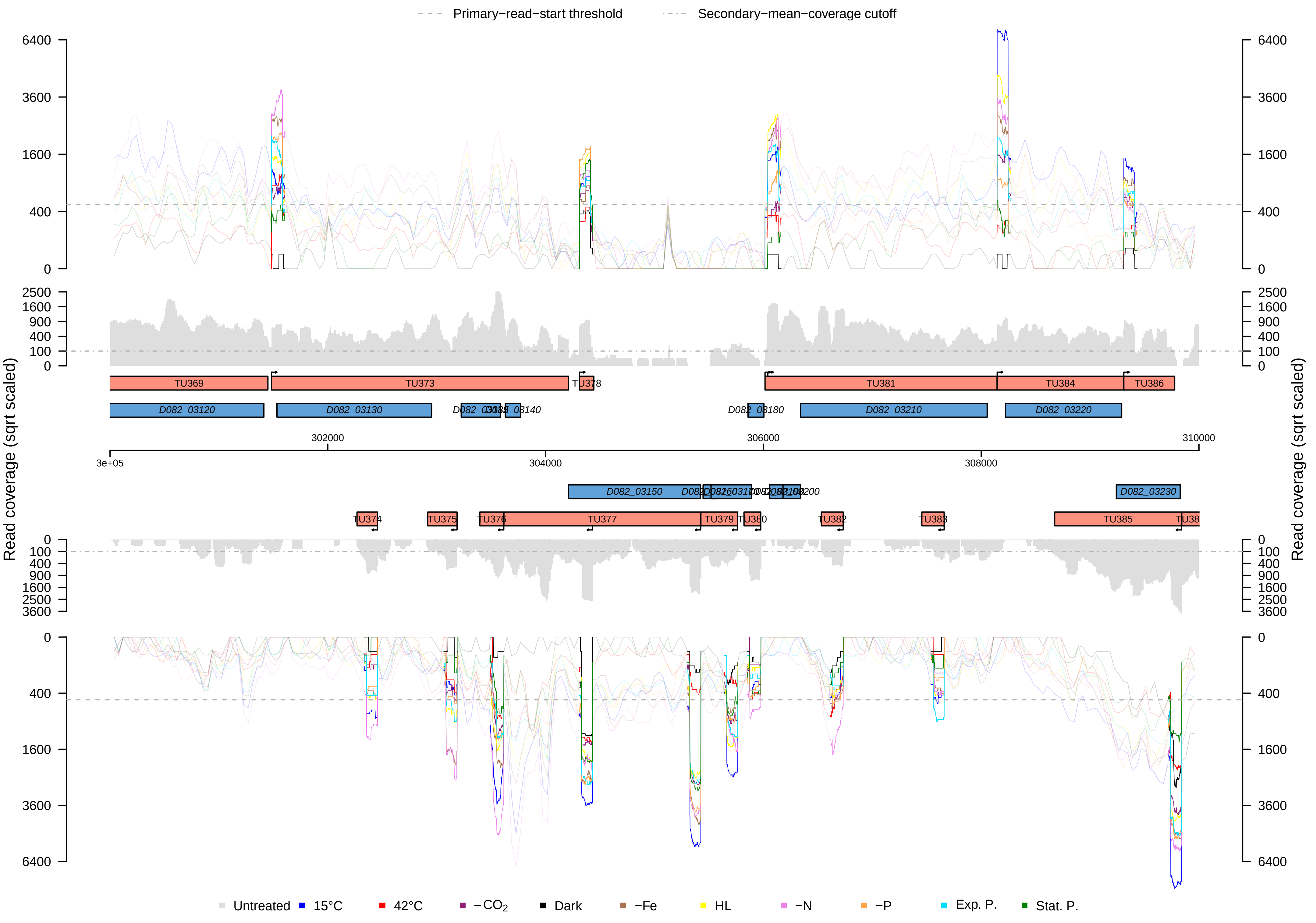

Primary−read−start threshold
Secondary−mean−coverage cutoff
Read coverage (sqrt scaled)
6400
3600
1600
400
0
2500
1600
900
400
100
0
TU369
TU373
TU378
TU381
TU384
TU386
D082_03120
D082_03130
D082_03140
D082_03180
D082_03210
D082_03220
3e+05
302000
304000
306000
308000
310000
D082_03150
D082_03230
TU374
TU375
TU376
TU377
TU379
TU380
TU382
TU383
TU385
Untreated
15°C
42°C
−CO_2
Dark
−Fe
HL
−N
−P
Exp. P.
Stat. P.

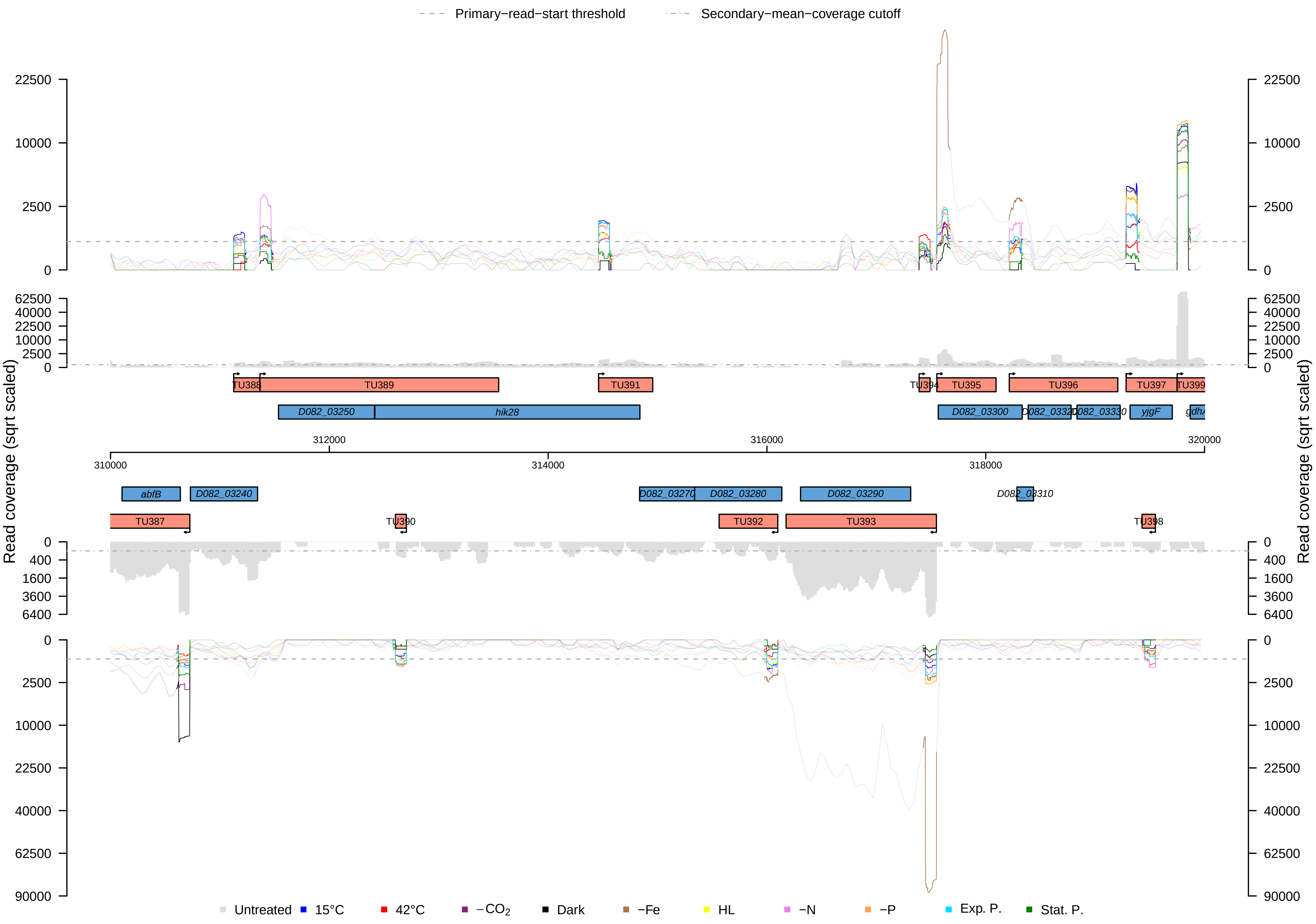

Primary−read−start threshold
Secondary−mean−coverage cutoff
Read coverage (sqrt scaled)
22500
10000
2500
0
62500
40000
22500
10000
2500
0
TU388
TU389
TU391
TU394
TU395
TU396
TU397
TU399
D082_03250
hik28
D082_03300
D082_03320
D082_03330
yjgF
gdhA
312000
316000
320000
310000
314000
318000
abfB
D082_03240
D082_03270
D082_03280
D082_03290
D082_03310
TU387
TU390
TU392
TU393
TU398
0
400
1600
3600
6400
0
2500
10000
22500
40000
62500
90000
Untreated
15°C
42°C
−CO2
Dark
−Fe
HL
−N
−P
Exp. P.
Stat. P.

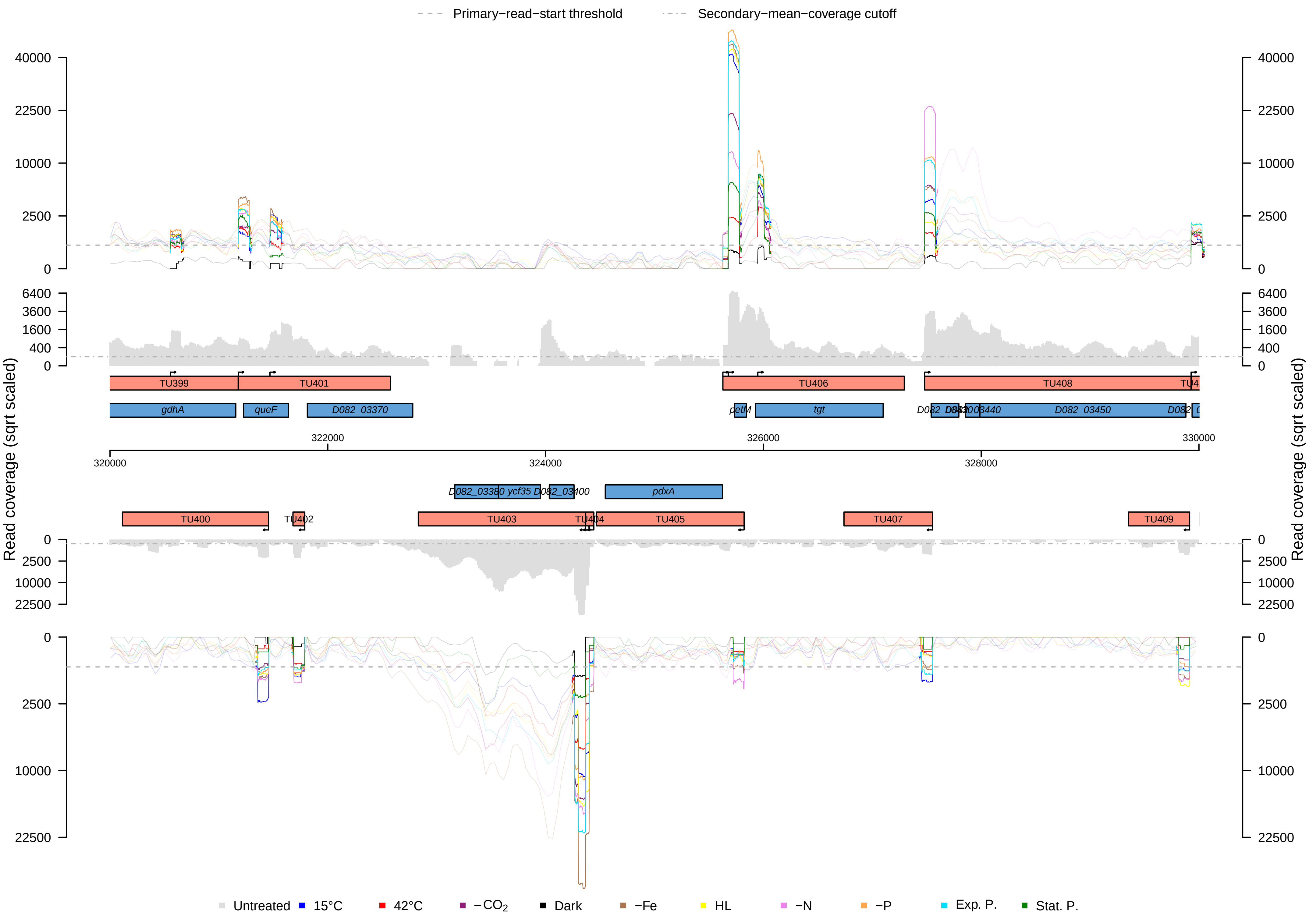

Primary−read−start threshold
Secondary−mean−coverage cutoff
40000
22500
10000
2500
0
6400
3600
1600
400
Read coverage (sqrt scaled)
TU399
TU401
TU406
TU408
gdhA
queF
D082_03370
petM
tgt
D082_03450
322000
326000
330000
320000
324000
328000
D082_03380
ycf35
pdxA
TU400
TU402
TU403
TU404
TU405
TU407
TU409
Untreated
15°C
42°C
−CO2
Dark
−Fe
HL
−N
−P
Exp. P.
Stat. P.

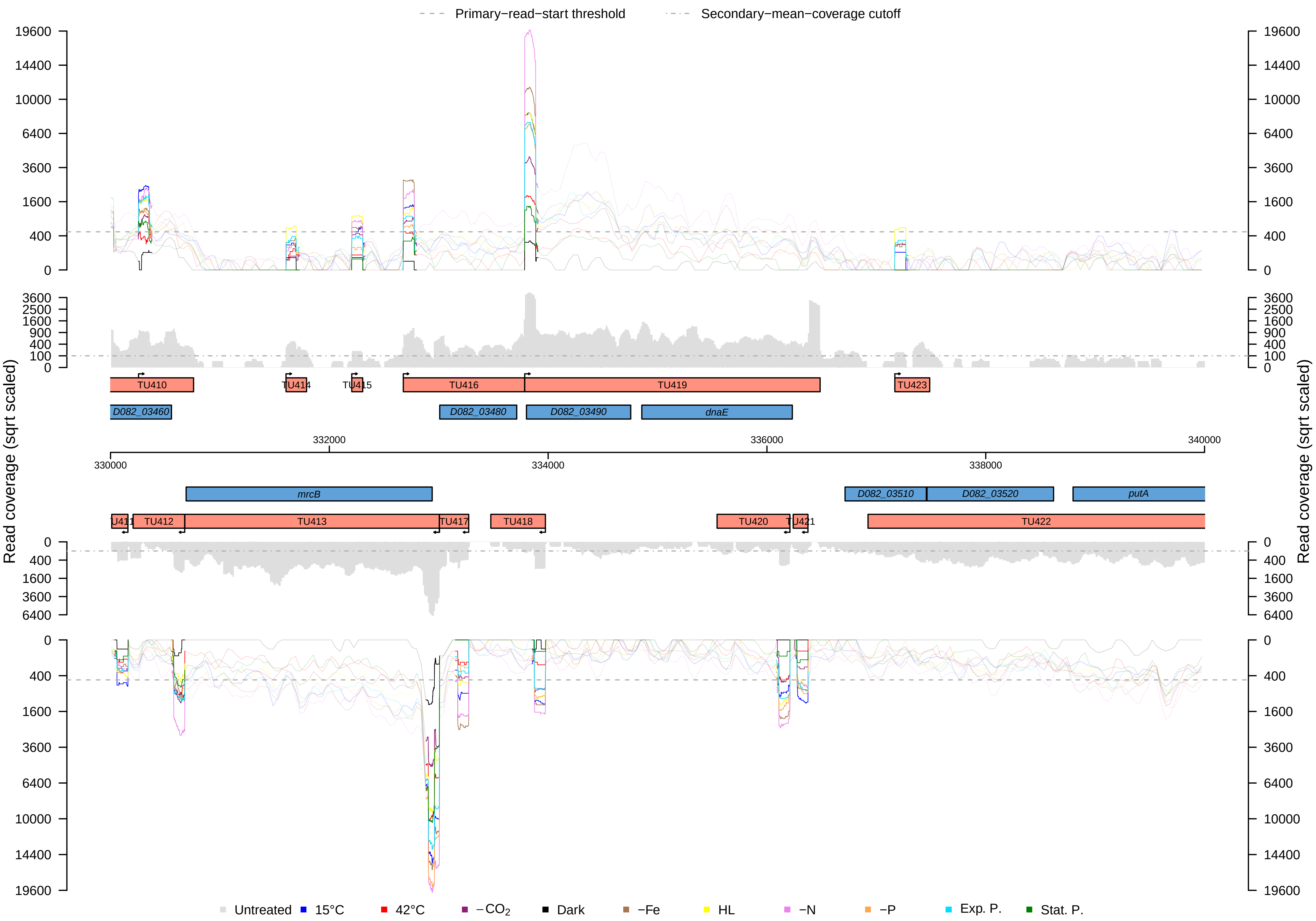

Primary−read−start threshold
Secondary−mean−coverage cutoff
Read coverage (sqrt scaled)
TU410
TU414
TU415
TU416
TU419
TU423
D082_03460
D082_03480
D082_03490
dnaE
330000
332000
334000
336000
338000
340000
mrcB
D082_03510
D082_03520
putA
TU411
TU412
TU413
TU417
TU418
TU420
TU421
TU422
Untreated
15°C
42°C
−CO2
Dark
−Fe
HL
−N
−P
Exp. P.
Stat. P.

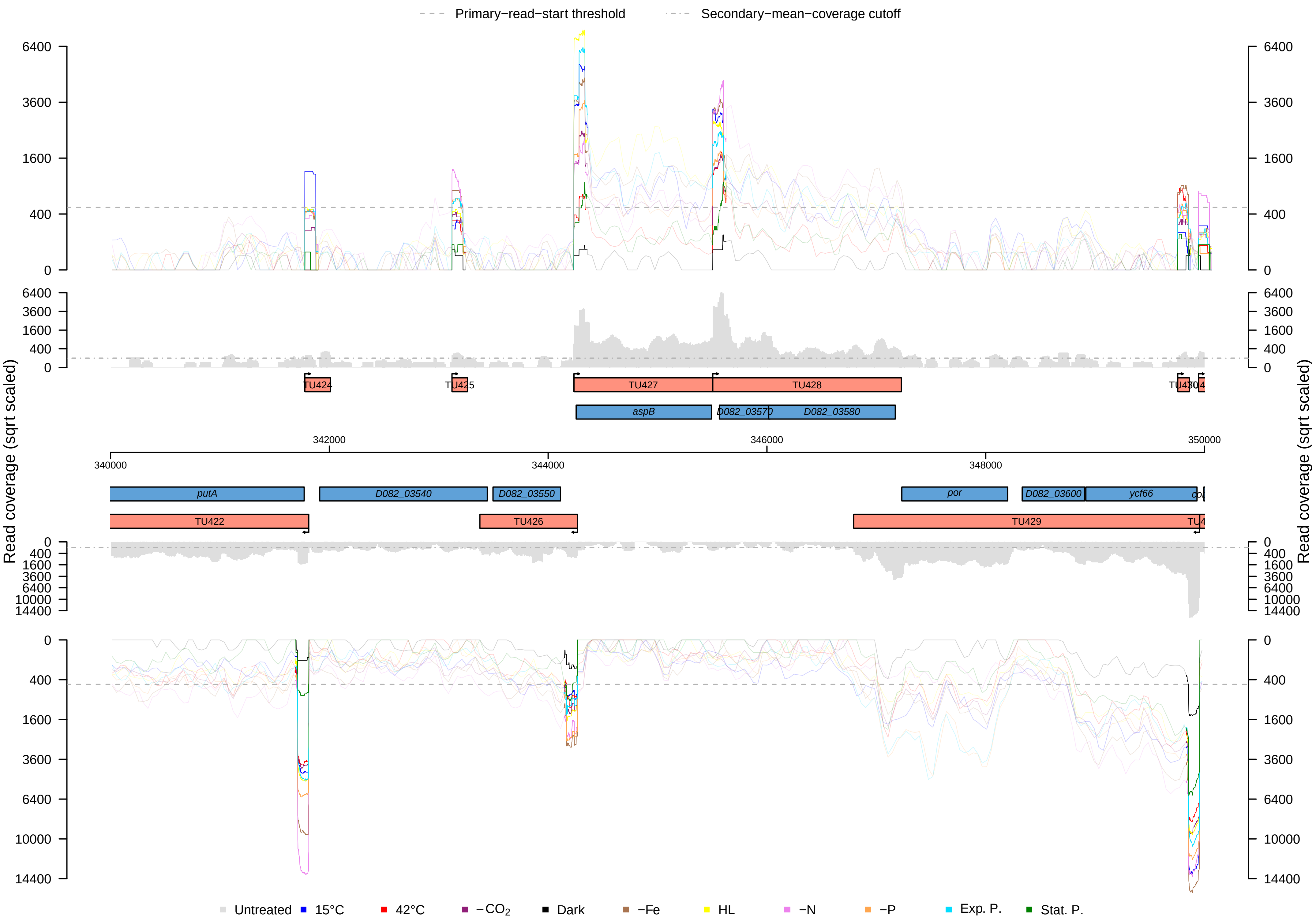

Primary−read−start threshold
Secondary−mean−coverage cutoff
Read coverage (sqrt scaled)
6400
3600
1600
400
0
TU424
TU425
TU427
TU428
aspB
D082_03570
D082_03580
340000
342000
344000
346000
348000
350000
putA
D082_03540
D082_03550
por
D082_03600
ycf66
TU422
TU426
TU429
10000
14400
Untreated
15°C
42°C
−CO_2
Dark
−Fe
HL
−N
−P
Exp. P.
Stat. P.

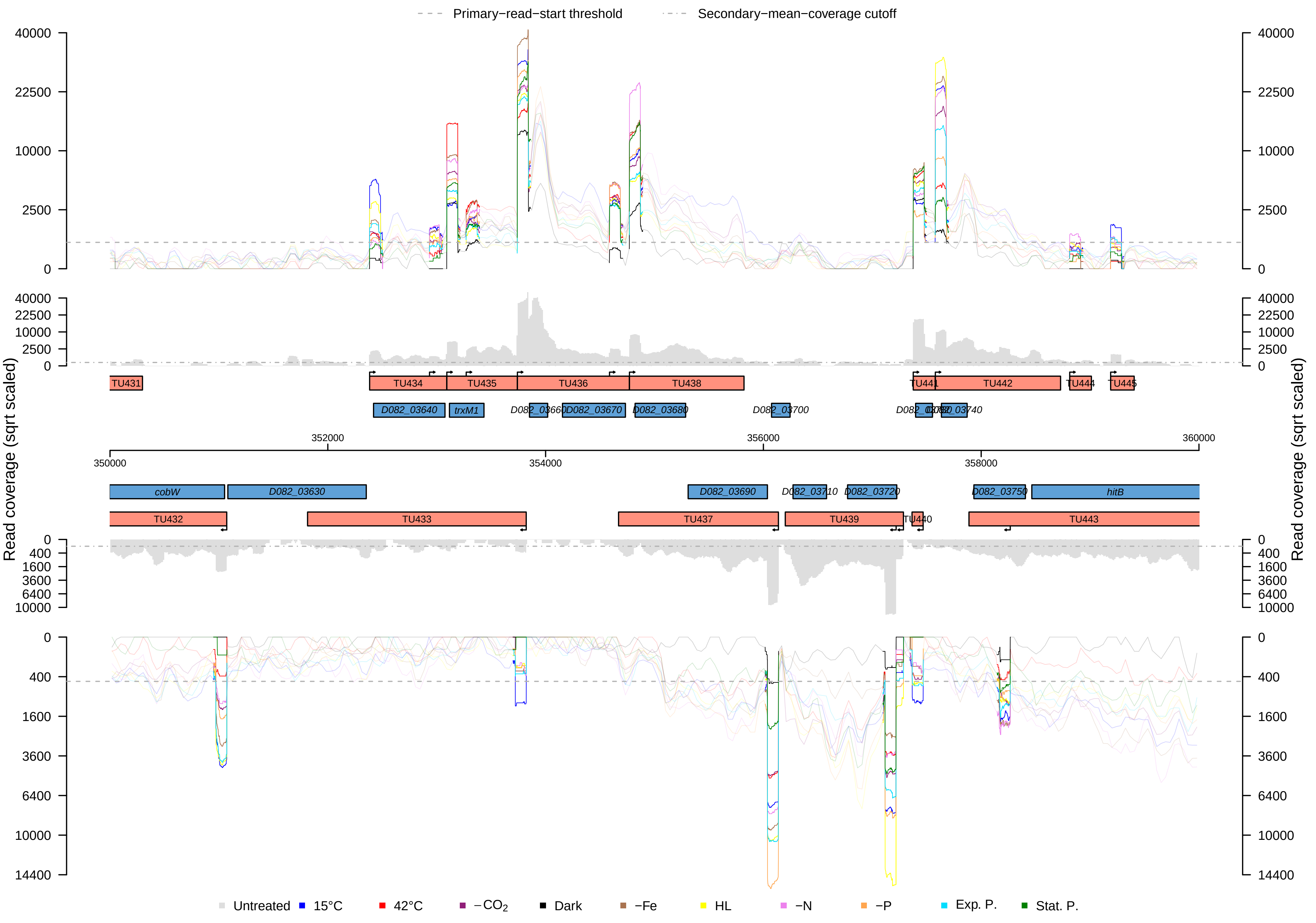

Primary−read−start threshold
Secondary−mean−coverage cutoff
Read coverage (sqrt scaled)
40000
22500
10000
2500
0
400
1600
3600
6400
10000
14400
TU431
TU432
TU433
TU434
TU435
TU436
TU437
TU438
TU439
TU440
TU441
TU442
TU443
TU444
TU445
cobW
D082_03630
D082_03640
trxM1
D082_03670
D082_03680
D082_03690
D082_03700
D082_03720
D082_03740
D082_03750
hitB
350000
352000
354000
356000
358000
360000
Untreated
15°C
42°C
−CO_2
Dark
−Fe
HL
−N
−P
Exp. P.
Stat. P.

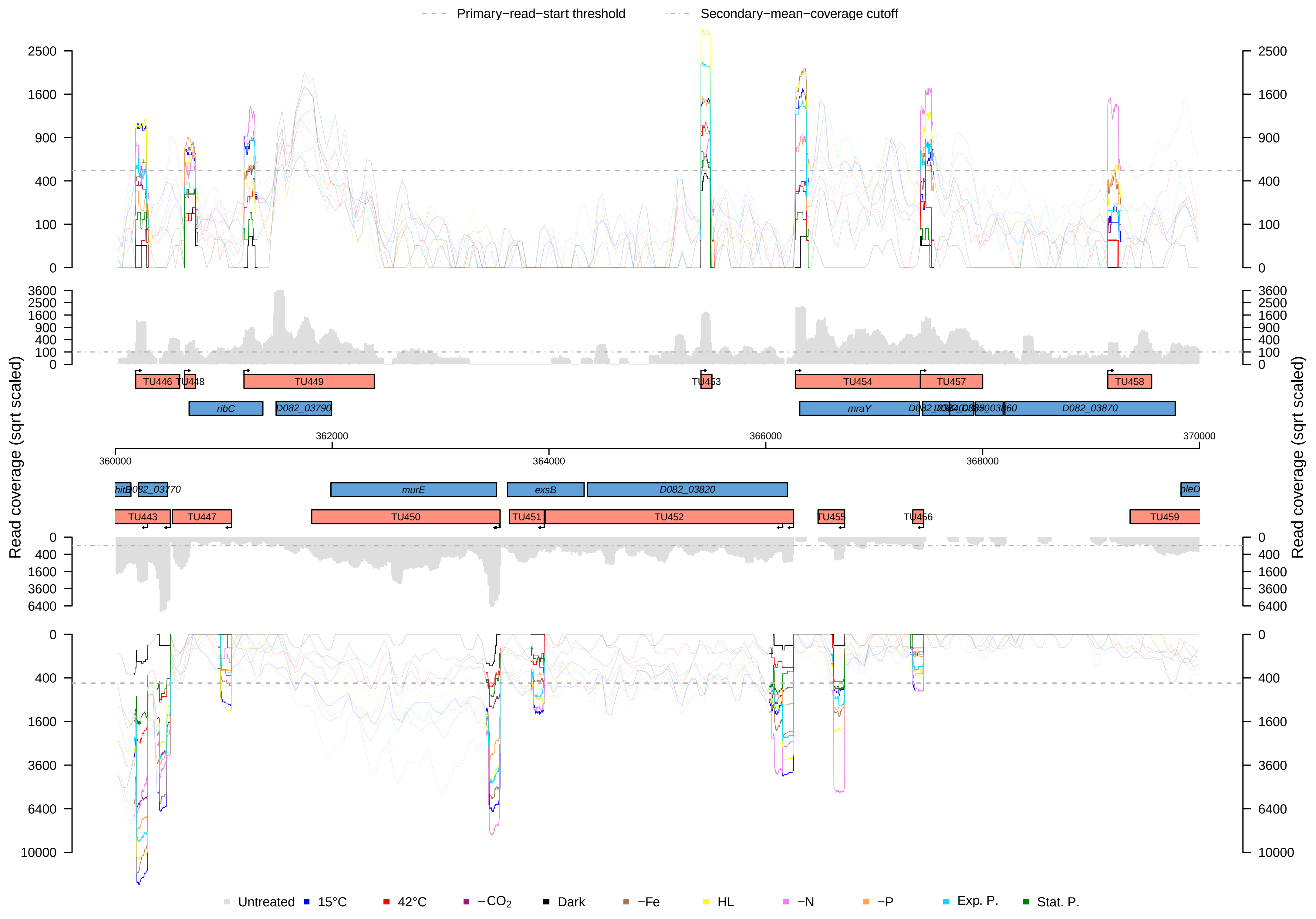

Primary−read−start threshold
Secondary−mean−coverage cutoff
Read coverage (sqrt scaled)
TU446
TU448
TU449
TU453
TU454
TU457
TU458
ribC
D082_03790
mraY
D082_03870
360000
362000
364000
366000
368000
370000
hitB
D082_03770
murE
exsB
D082_03820
pleD
TU443
TU447
TU450
TU451
TU452
TU455
TU456
TU459
Untreated
15°C
42°C
−CO2
Dark
−Fe
HL
−N
−P
Exp. P.
Stat. P.

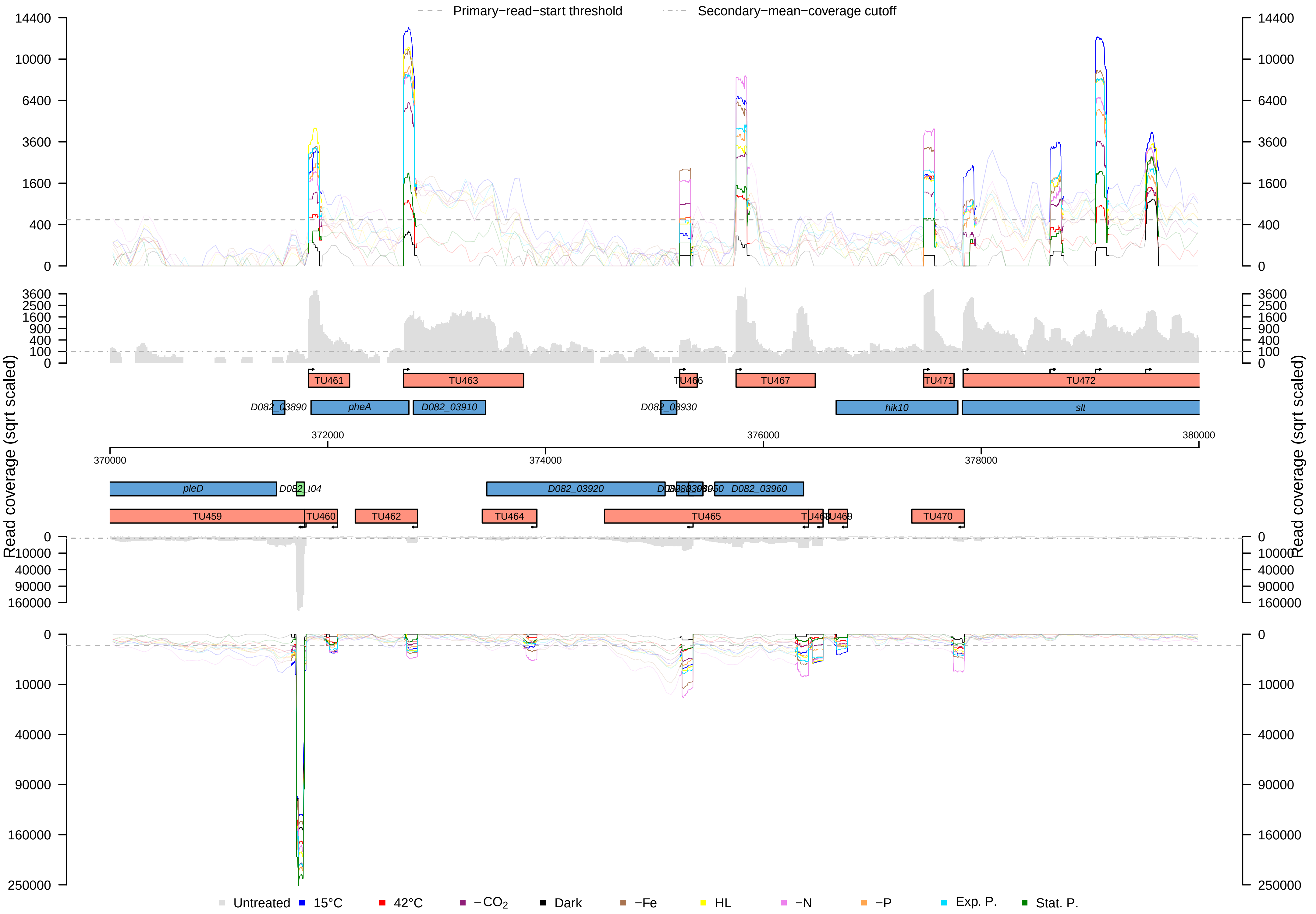

Primary−read−start threshold
Secondary−mean−coverage cutoff
Read coverage (sqrt scaled)
TU459
TU460
TU461
TU462
TU463
TU464
TU465
TU466
TU467
TU468
TU469
TU470
TU471
TU472
pleD
D082_t04
D082_03890
pheA
D082_03910
D082_03920
D082_03930
D082_03960
hik10
slt
370000
372000
374000
376000
378000
380000
Untreated
15°C
42°C
−CO2
Dark
−Fe
HL
−N
−P
Exp. P.
Stat. P.

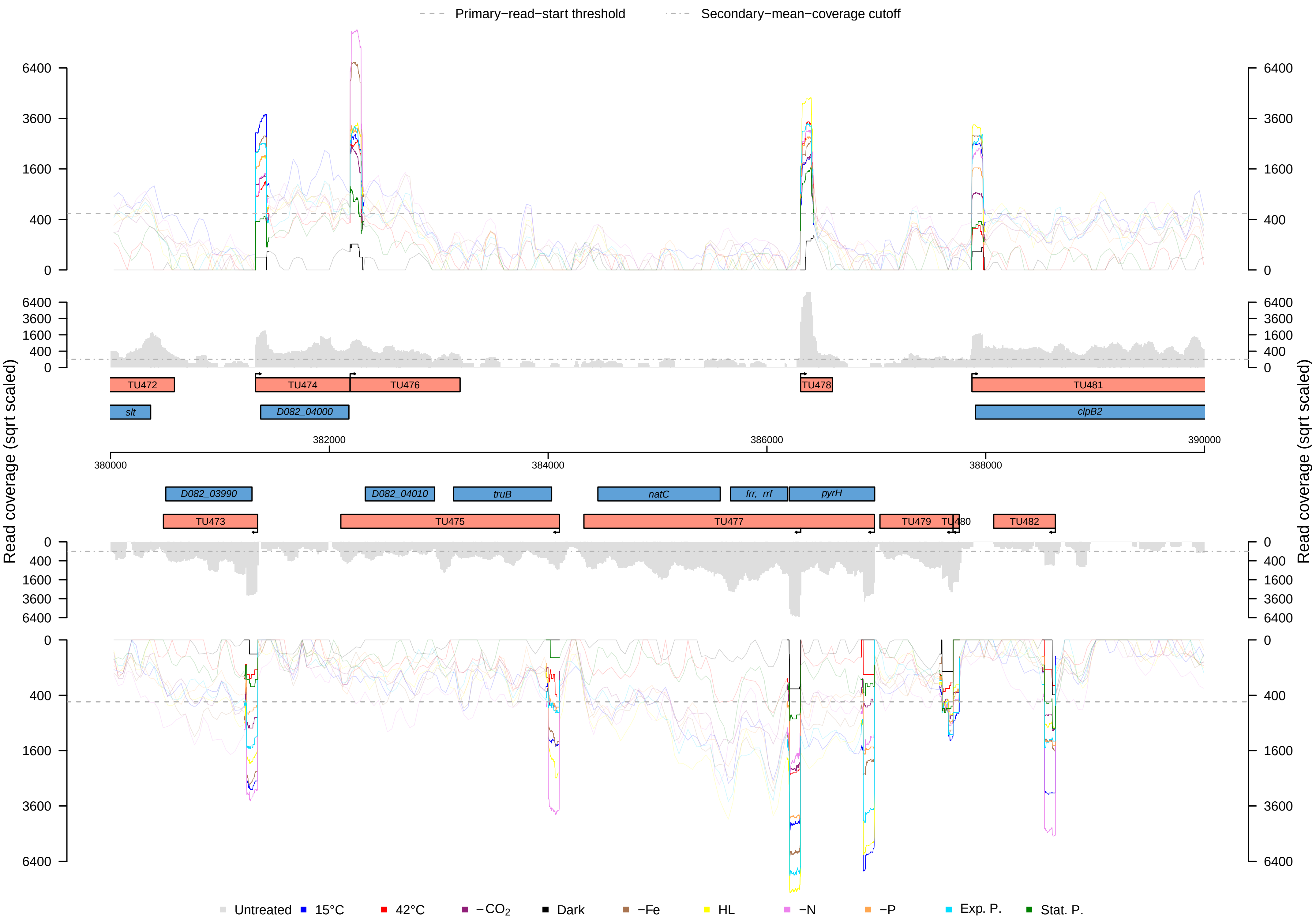

Primary−read−start threshold
Secondary−mean−coverage cutoff
Read coverage (sqrt scaled)
6400
3600
1600
400
0
TU472
TU474
TU476
TU478
TU481
slt
D082_04000
clpB2
380000
382000
384000
386000
388000
390000
D082_03990
D082_04010
truB
natC
frr, rrf
pyrH
TU473
TU475
TU477
TU479
TU480
TU482
Untreated
15°C
42°C
−CO2
Dark
−Fe
HL
−N
−P
Exp. P.
Stat. P.

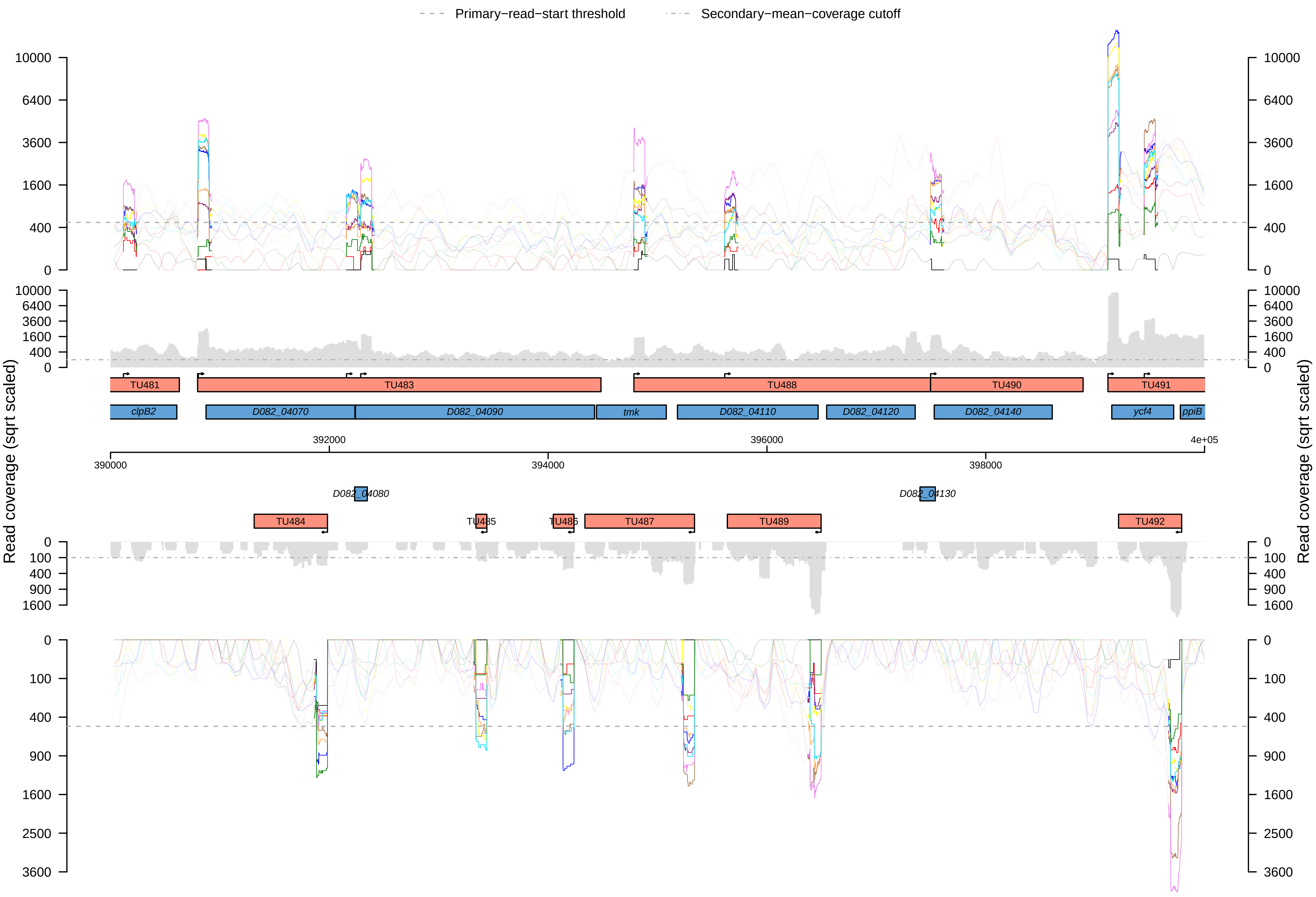

Primary−read−start threshold
Secondary−mean−coverage cutoff
Read coverage (sqrt scaled)
10000
6400
3600
1600
400
0
TU481
TU483
TU488
TU490
TU491
clpB2
D082_04070
D082_04090
tmk
D082_04110
D082_04120
D082_04140
ycf4
ppiB
392000
396000
4e+05
390000
394000
398000
D082_04080
D082_04130
TU484
TU485
TU486
TU487
TU489
TU492
100
900
2500
Untreated
15°C
42°C
−CO2
Dark
−Fe
HL
−N
−P
Exp. P.
Stat. P.

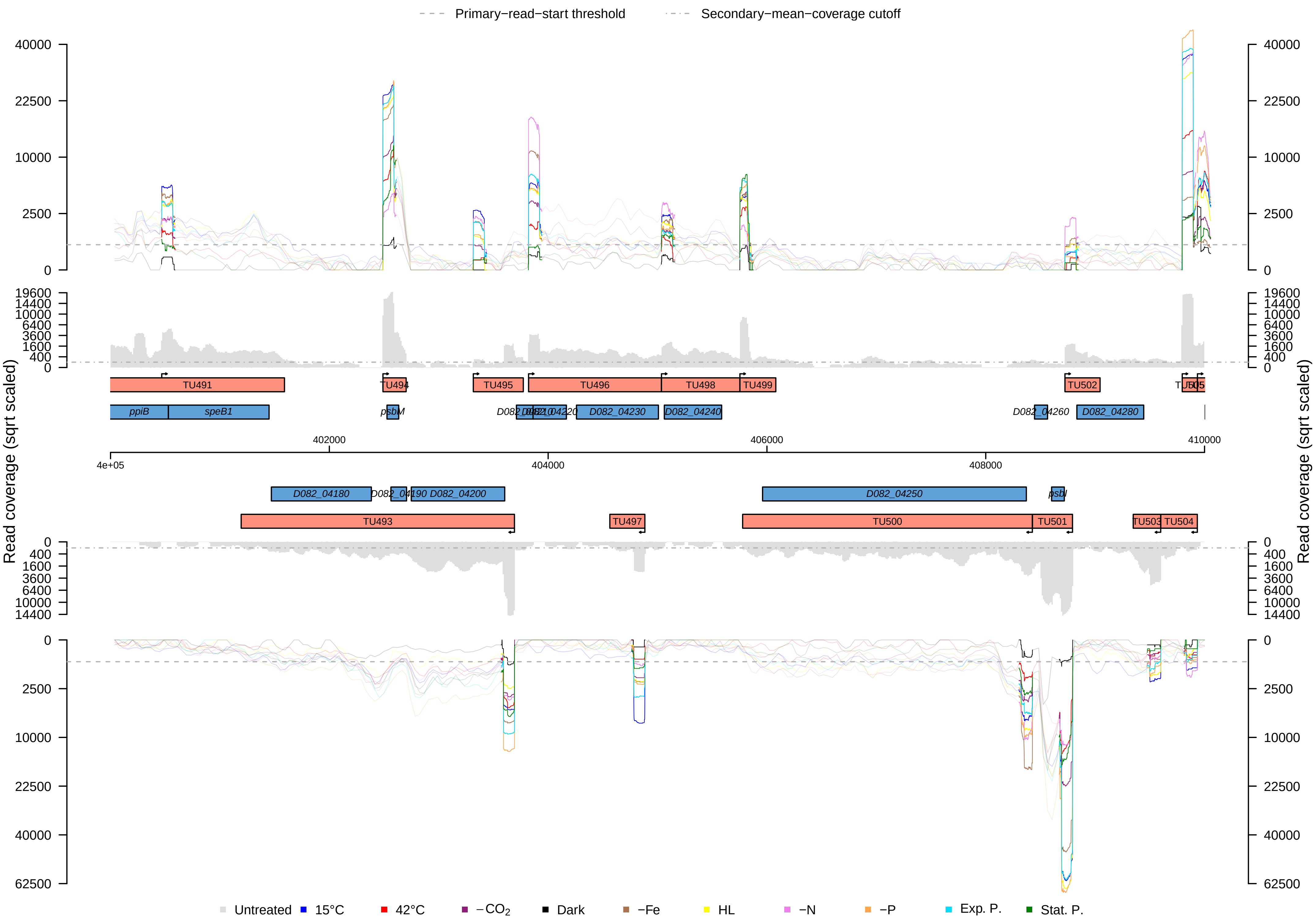

Primary−read−start threshold
Secondary−mean−coverage cutoff
Read coverage (sqrt scaled)
40000
22500
10000
2500
0
19600
14400
10000
6400
3600
1600
400
TU491
TU494
TU495
TU496
TU498
TU499
TU502
ppiB
speB1
psbM
D082_04230
D082_04240
D082_04280
402000
406000
410000
4e+05
404000
408000
D082_04180
D082_04200
D082_04250
TU493
TU497
TU500
TU501
TU503
TU504
62500
Untreated
15°C
42°C
−CO2
Dark
−Fe
HL
−N
−P
Exp. P.
Stat. P.

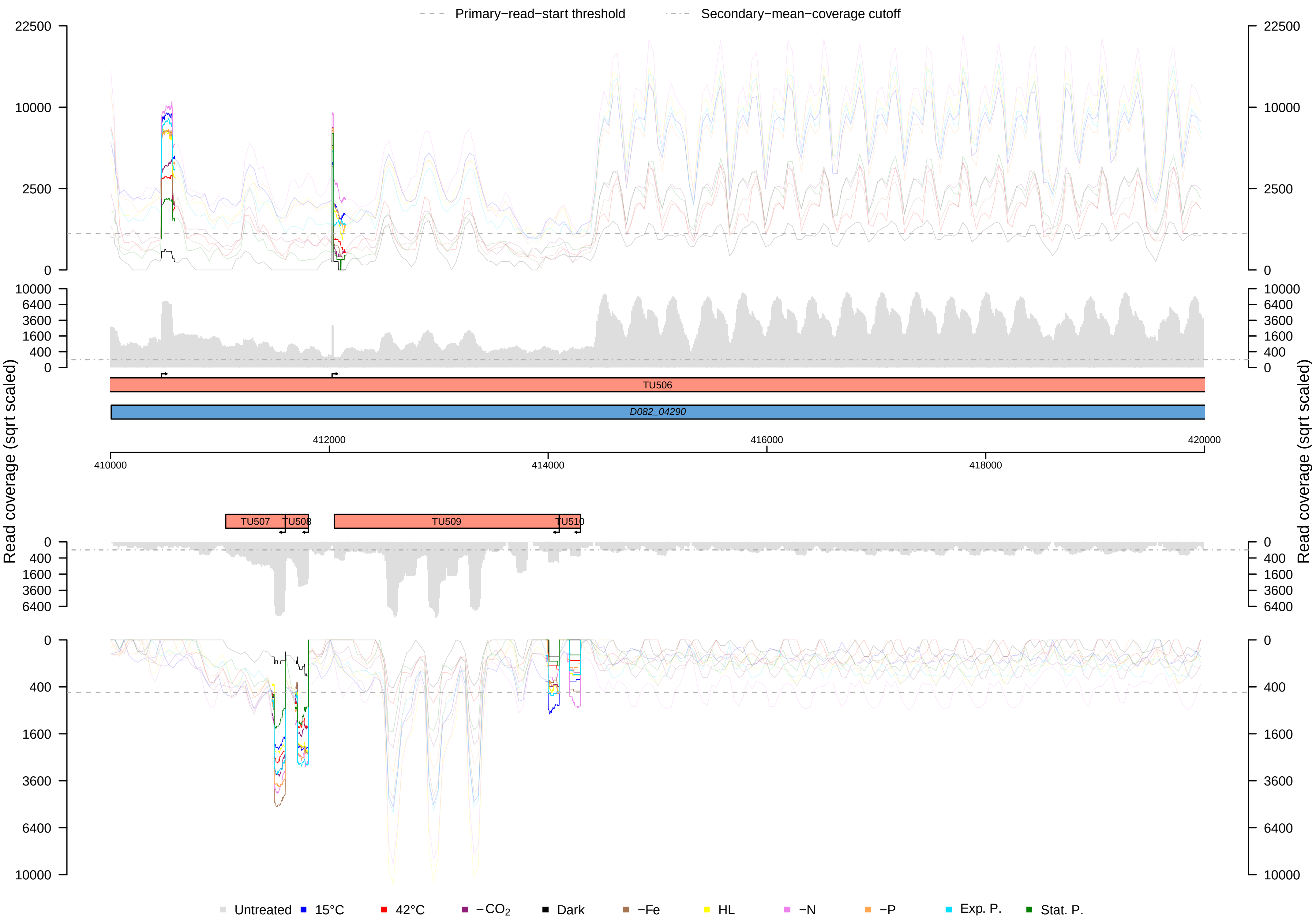

Primary−read−start threshold
Secondary−mean−coverage cutoff
Read coverage (sqrt scaled)
22500
10000
2500
0
6400
3600
1600
400
TU506
D082_04290
410000
412000
414000
416000
418000
420000
TU507
TU508
TU509
TU510
Untreated
15°C
42°C
−CO2
Dark
−Fe
HL
−N
−P
Exp. P.
Stat. P.

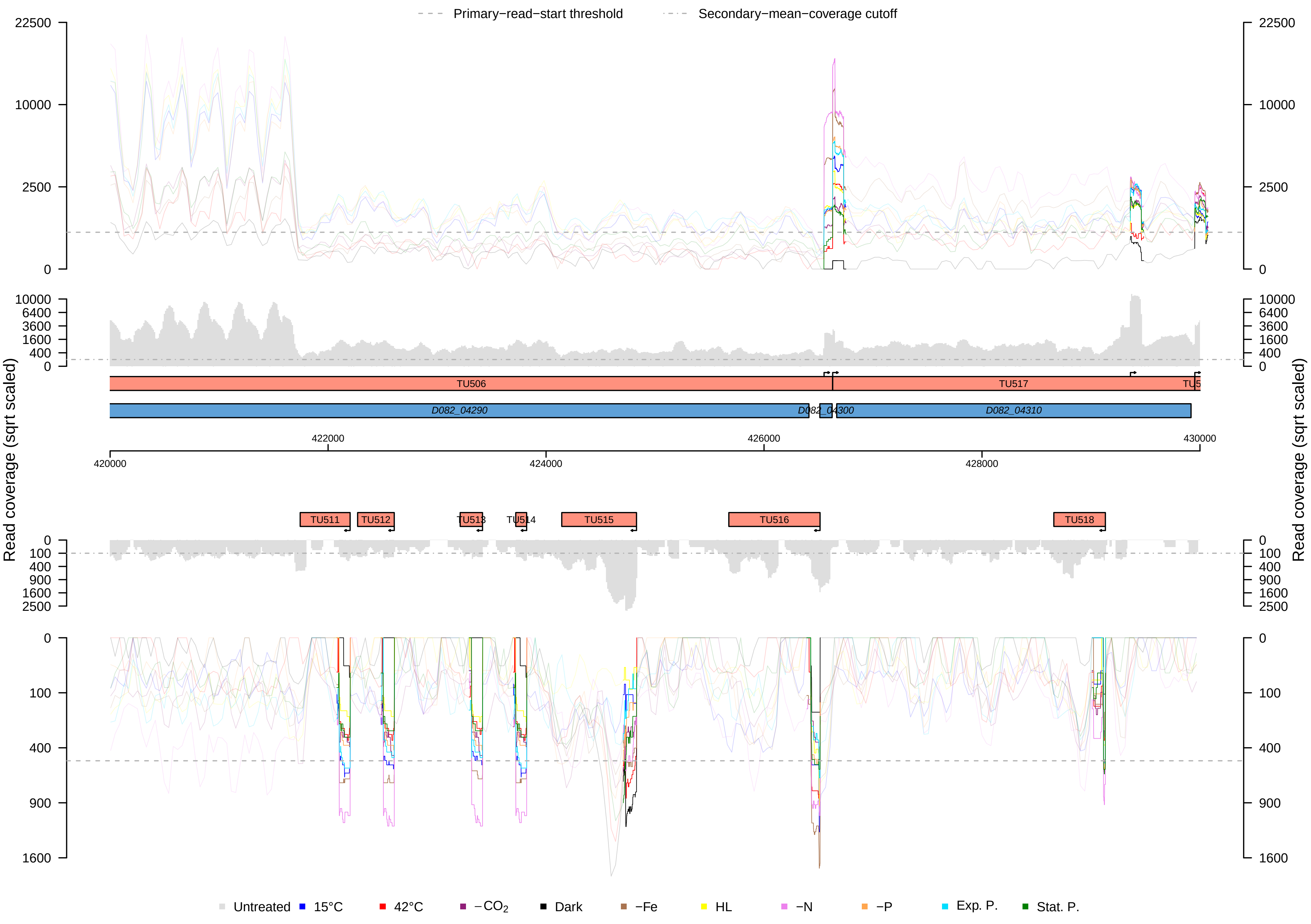

Primary−read−start threshold
Secondary−mean−coverage cutoff
Read coverage (sqrt scaled)
TU506
TU517
TU5
D082_04290
D082_04300
D082_04310
TU511
TU512
TU513
TU514
TU515
TU516
TU518
420000
422000
424000
426000
428000
430000
Untreated
15°C
42°C
−CO2
Dark
−Fe
HL
−N
−P
Exp. P.
Stat. P.

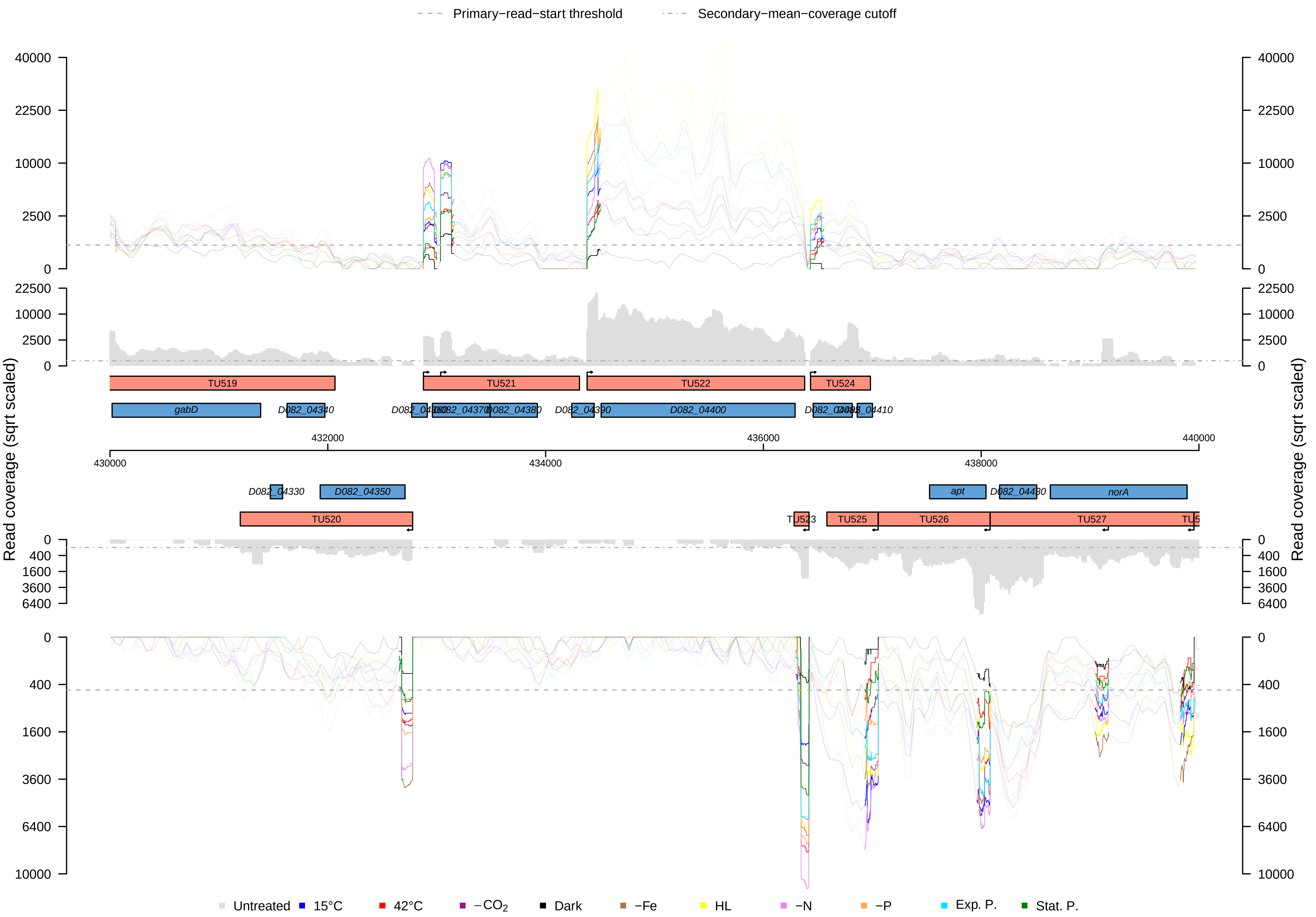
Primary−read−start threshold
Secondary−mean−coverage cutoff
Read coverage (sqrt scaled)
40000
22500
10000
2500
0
TU519
TU521
TU522
TU524
gabD
D082_04340
D082_04390
D082_04400
D082_04410
430000
432000
434000
436000
438000
440000
D082_04330
D082_04350
apt
D082_04430
norA
TU520
TU523
TU525
TU526
TU527
400
1600
3600
6400
Untreated
15°C
42°C
−CO_2
Dark
−Fe
HL
−N
−P
Exp. P.
Stat. P.

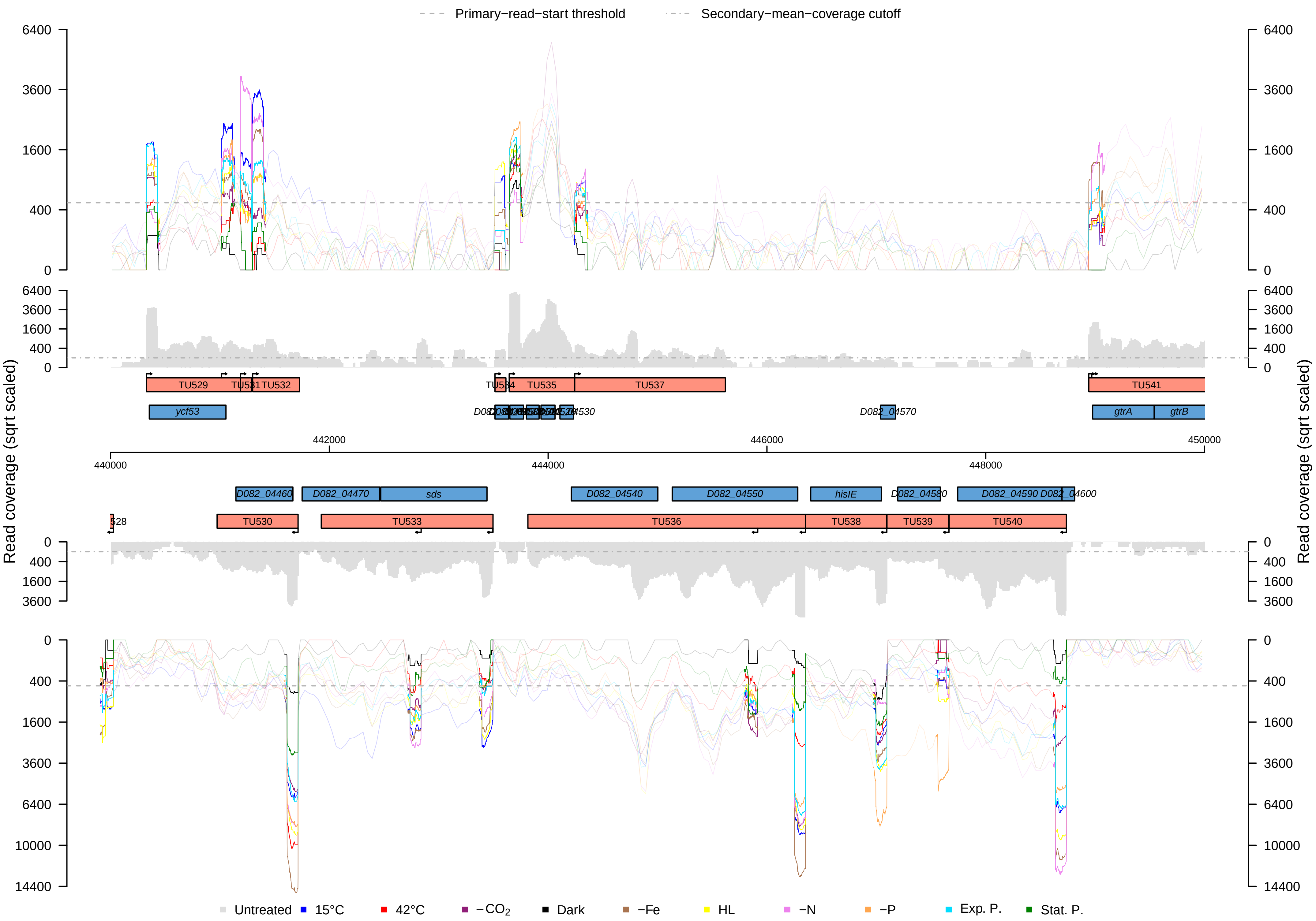

Primary−read−start threshold
Secondary−mean−coverage cutoff
Read coverage (sqrt scaled)
6400
3600
1600
400
0
10000
14400
TU529
TU531
TU532
TU534
TU535
TU537
TU541
ycf53
D082_04570
gtrA
gtrB
440000
442000
444000
446000
448000
450000
D082_04460
D082_04470
sds
D082_04540
D082_04550
hisIE
D082_04580
D082_04590
D082_04600
TU530
TU533
TU536
TU538
TU539
TU540
Untreated
15°C
42°C
−CO2
Dark
−Fe
HL
−N
−P
Exp. P.
Stat. P.

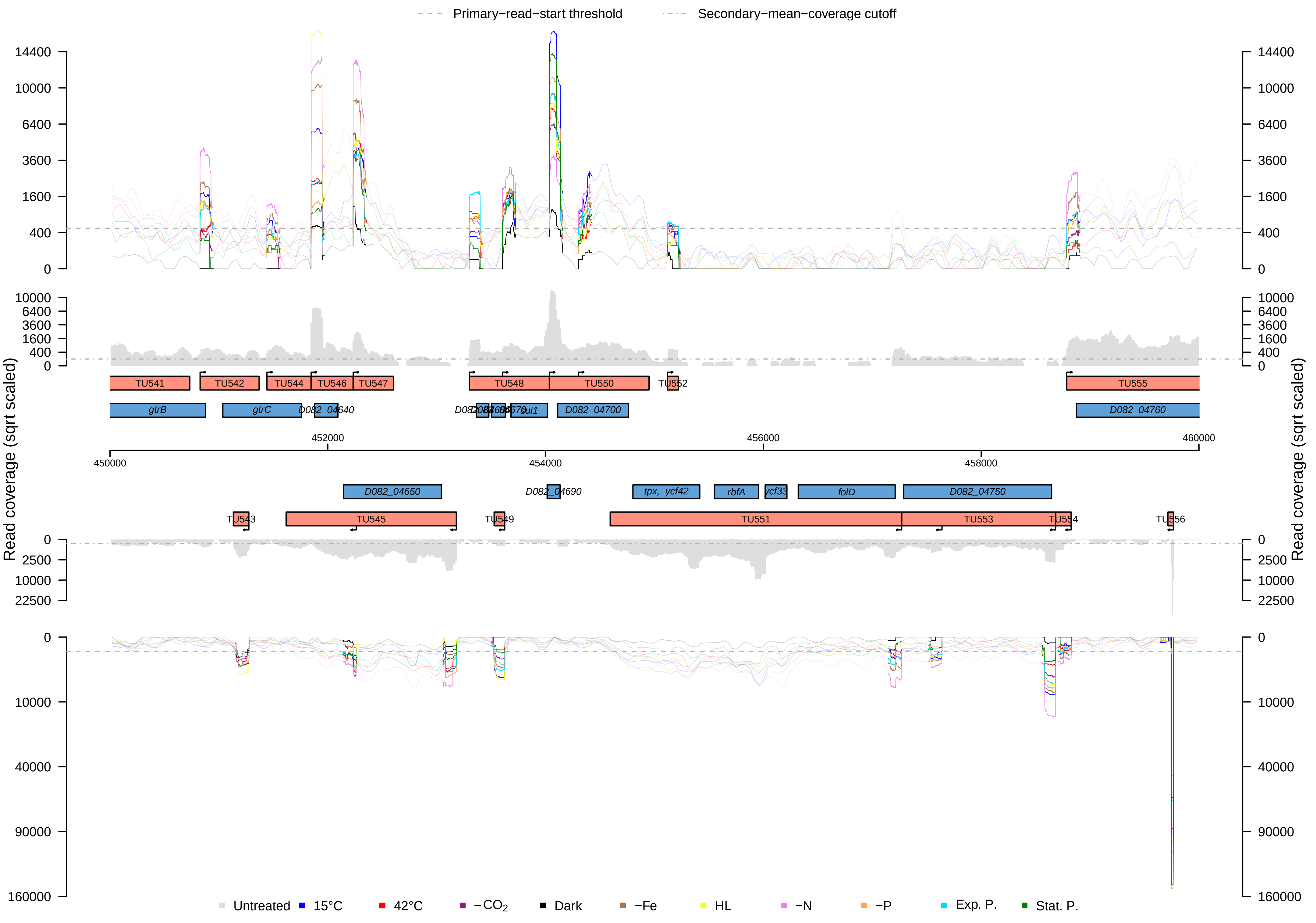

Primary−read−start threshold
Secondary−mean−coverage cutoff
Read coverage (sqrt scaled)
TU541
TU542
TU544
TU546
TU547
TU548
TU550
TU552
TU555
gtrB
gtrC
D082_04640
sui1
D082_04700
D082_04760
450000
452000
454000
456000
458000
460000
D082_04650
D082_04690
tpx, ycf42
rbfA
ycf33
folD
D082_04750
TU543
TU545
TU549
TU551
TU553
TU554
TU556
Untreated
15°C
42°C
−CO2
Dark
−Fe
HL
−N
−P
Exp. P.
Stat. P.

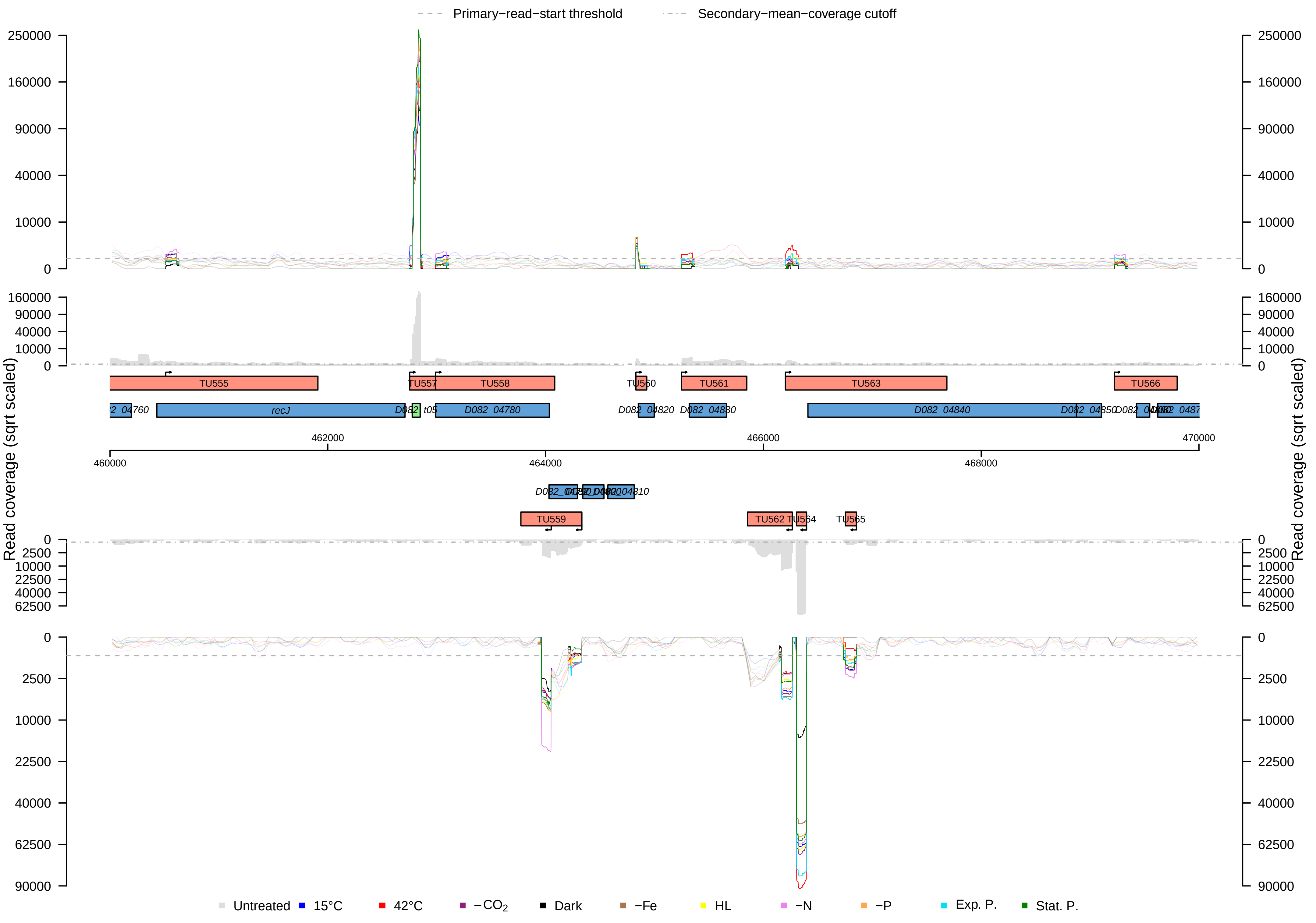

Primary−read−start threshold
Secondary−mean−coverage cutoff
Read coverage (sqrt scaled)
TU555
TU557
TU558
TU560
TU561
TU563
TU566
recJ
D082_04780
D082_04820
D082_04830
D082_04840
D082_04850
TU559
TU562
TU564
TU565
460000
462000
464000
466000
468000
470000
Untreated
15°C
42°C
−CO2
Dark
−Fe
HL
−N
−P
Exp. P.
Stat. P.

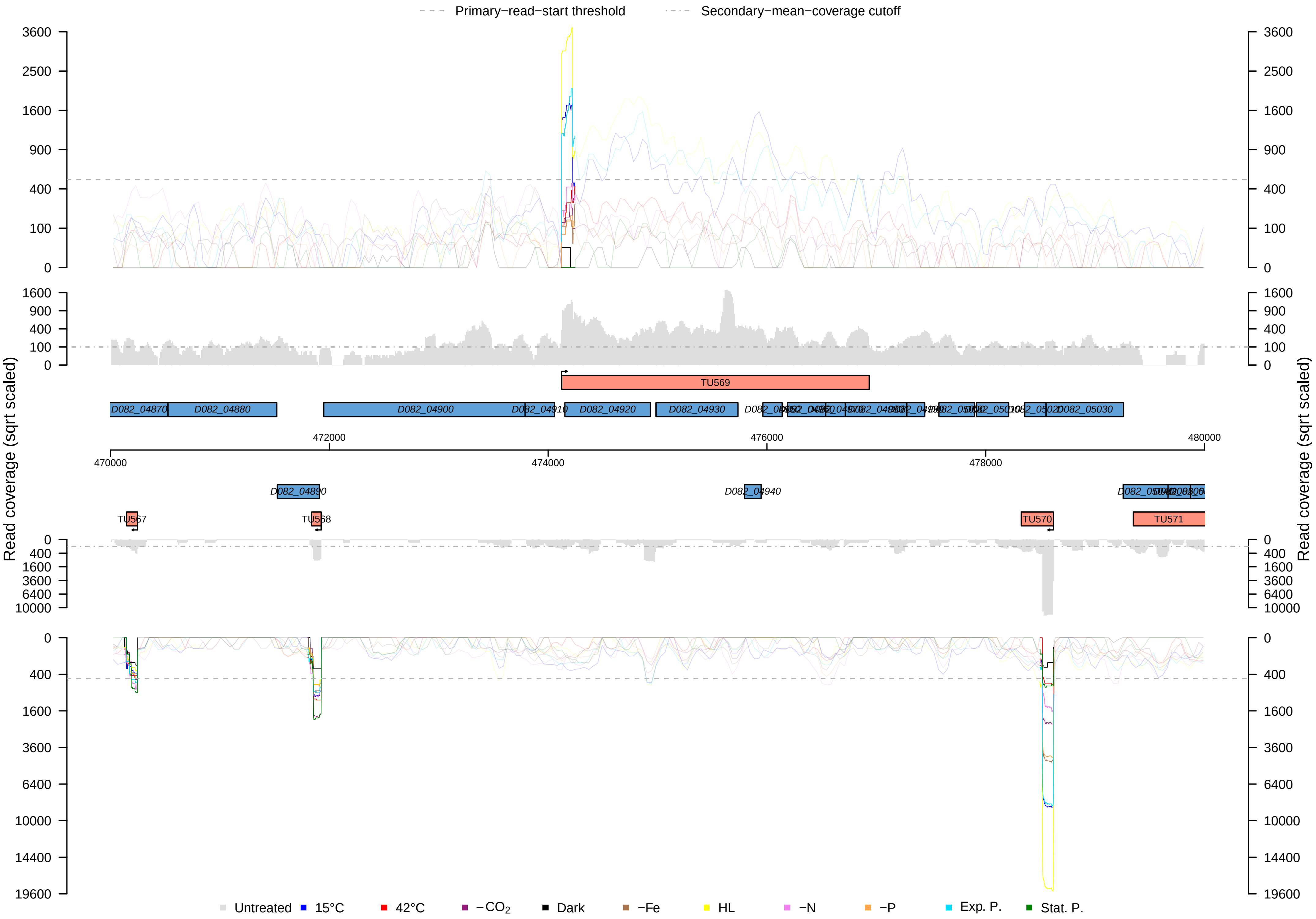

Primary−read−start threshold
Secondary−mean−coverage cutoff
Read coverage (sqrt scaled)
Read coverage (sqrt scaled)
3600
2500
1600
900
400
100
0
1600
900
400
100
0
TU569
D082_04870
D082_04880
D082_04900
D082_04910
D082_04920
D082_04930
D082_05030
470000
472000
474000
476000
478000
480000
D082_04890
D082_04940
TU567
TU568
TU570
TU571
0
400
1600
3600
6400
10000
14400
19600
Untreated
15°C
42°C
−CO$_2$
Dark
−Fe
HL
−N
−P
Exp. P.
Stat. P.

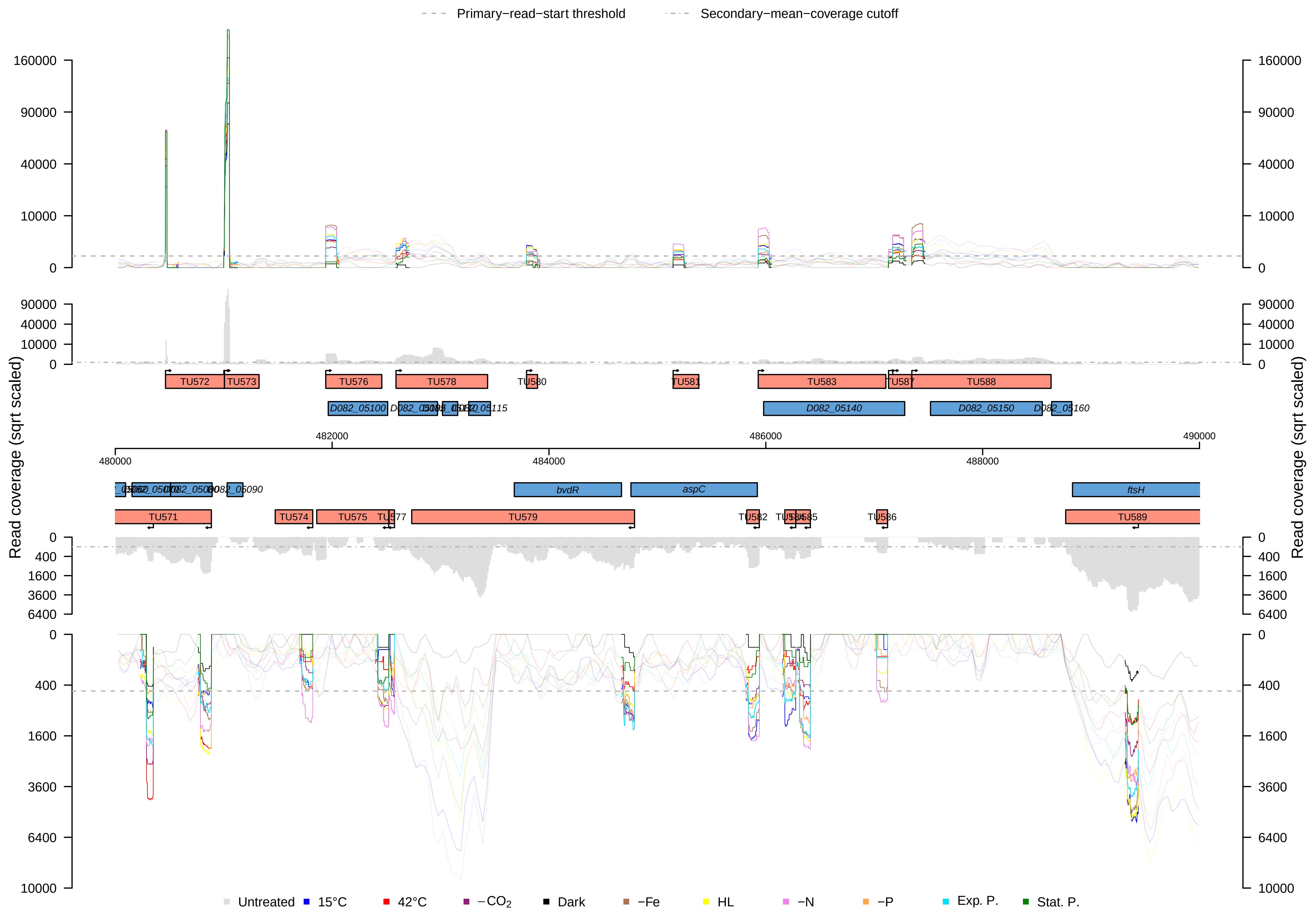

Primary−read−start threshold
Secondary−mean−coverage cutoff
Read coverage (sqrt scaled)
160000
90000
40000
10000
0
TU571
TU572
TU573
TU574
TU575
TU576
TU577
TU578
TU579
TU580
TU581
TU582
TU583
TU586
TU587
TU588
TU589
D082_05100
D082_05140
D082_05150
D082_05090
bvdR
aspC
ftsH
480000
482000
484000
486000
488000
490000
400
1600
3600
6400
10000
Untreated
15°C
42°C
−CO2
Dark
−Fe
HL
−N
−P
Exp. P.
Stat. P.

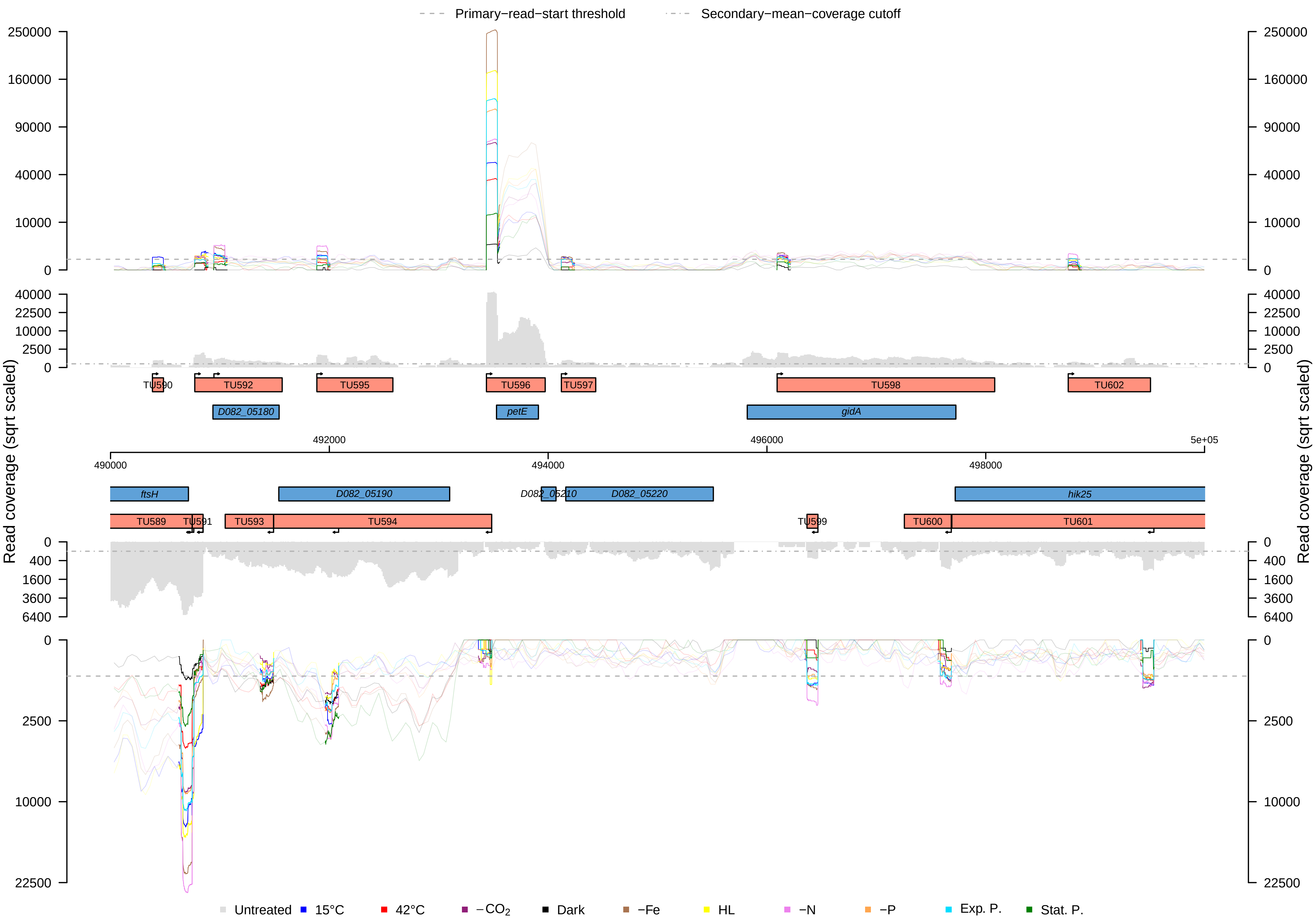

Primary−read−start threshold
Secondary−mean−coverage cutoff
250000
160000
90000
40000
10000
0
40000
22500
10000
2500
0
Read coverage (sqrt scaled)
TU590
TU592
TU595
TU596
TU597
TU598
TU602
D082_05180
petE
gidA
492000
496000
5e+05
490000
494000
498000
ftsH
D082_05190
D082_05210
D082_05220
hik25
TU589
TU591
TU593
TU594
TU599
TU600
TU601
400
1600
3600
6400
2500
10000
22500
Untreated
15°C
42°C
−CO2
Dark
−Fe
HL
−N
−P
Exp. P.
Stat. P.

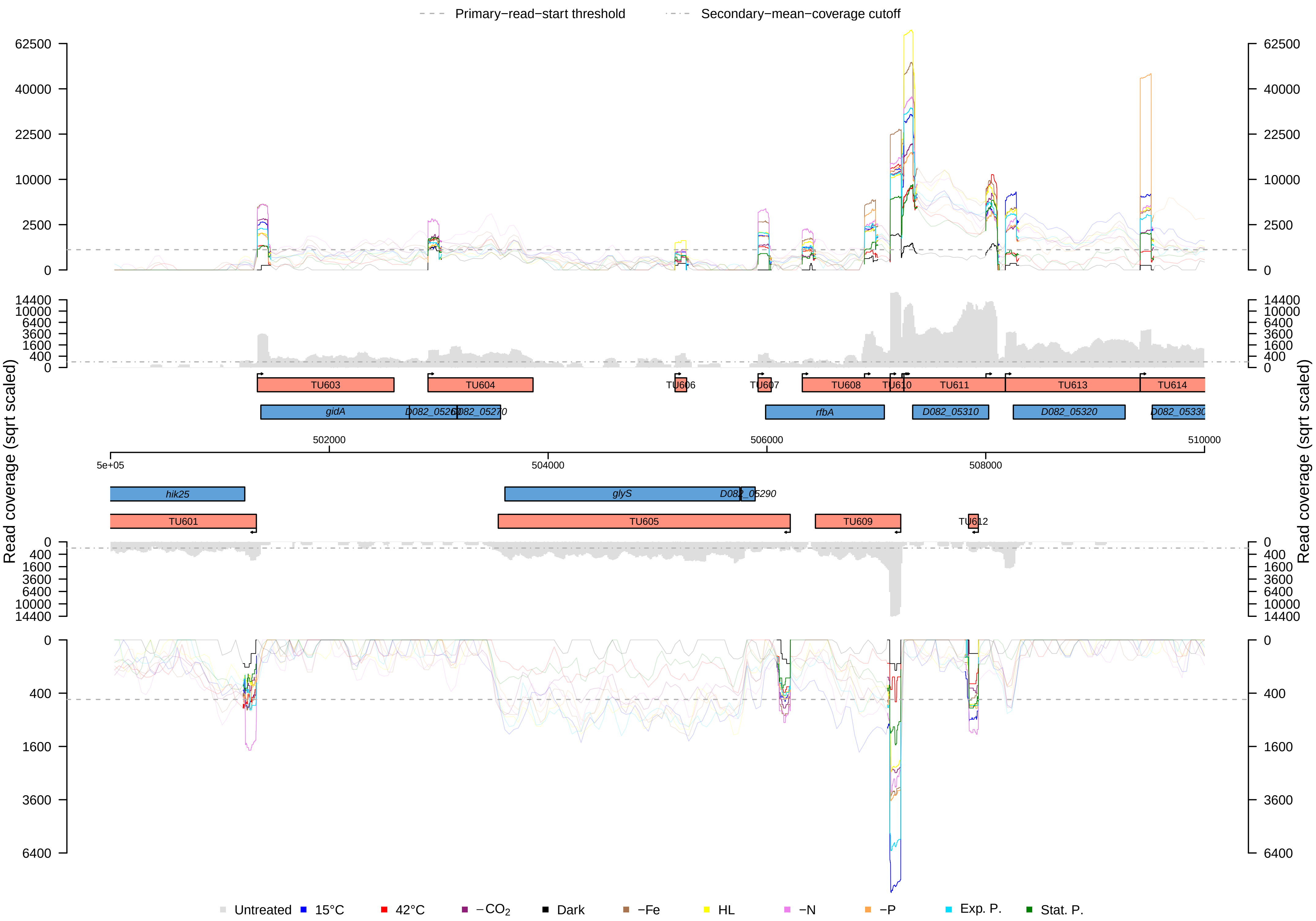

Primary−read−start threshold
Secondary−mean−coverage cutoff
Read coverage (sqrt scaled)
62500
40000
22500
10000
2500
0
14400
10000
6400
3600
1600
400
TU603
TU604
TU606
TU607
TU608
TU610
TU611
TU613
TU614
gidA
D082_05260
D082_05270
rfbA
D082_05310
D082_05320
D082_05330
502000
506000
510000
5e+05
504000
508000
hik25
glyS
D082_05290
TU601
TU605
TU609
TU612
6400
Untreated
15°C
42°C
−CO2
Dark
−Fe
HL
−N
−P
Exp. P.
Stat. P.

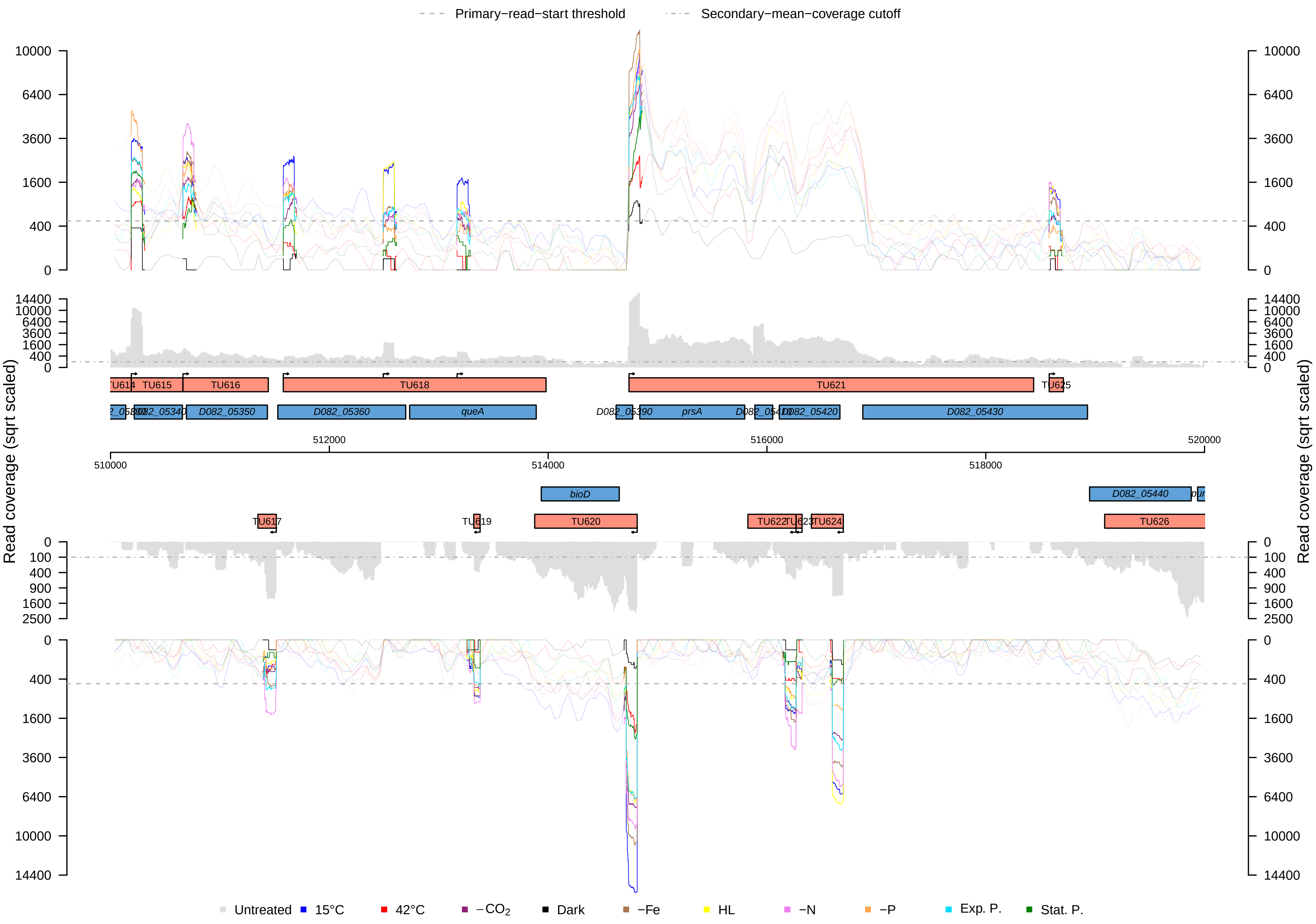

Primary−read−start threshold
Secondary−mean−coverage cutoff
Read coverage (sqrt scaled)
TU614
TU615
TU616
TU618
TU621
TU625
D082_05340
D082_05350
D082_05360
queA
D082_05390
prsA
D082_05420
D082_05430
bioD
D082_05440
TU617
TU619
TU620
TU622
TU624
TU626
510000
512000
514000
516000
518000
520000
Untreated
15°C
42°C
−CO2
Dark
−Fe
HL
−N
−P
Exp. P.
Stat. P.

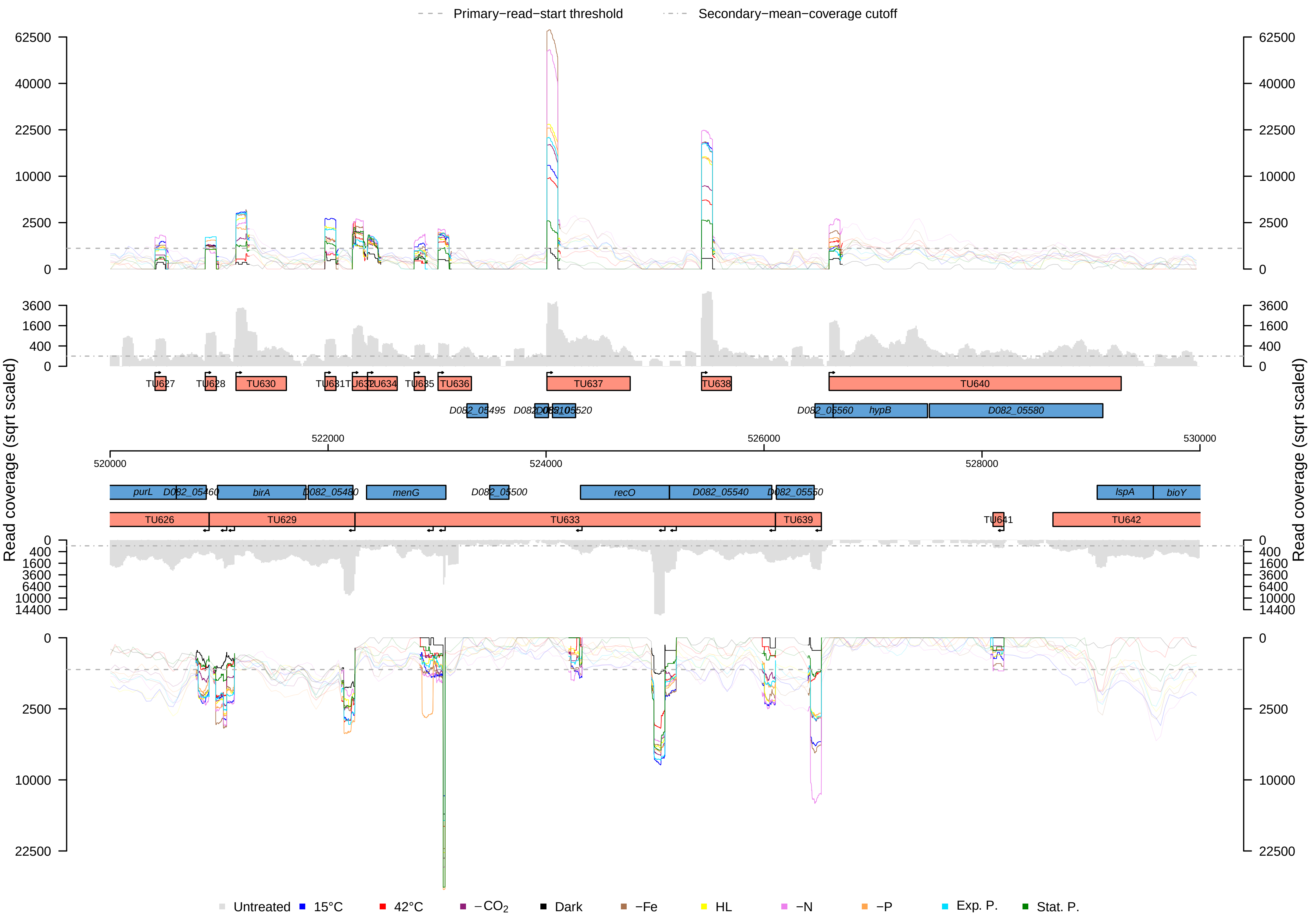

Primary−read−start threshold
Secondary−mean−coverage cutoff
Read coverage (sqrt scaled)
62500
40000
22500
10000
2500
0
3600
1600
400
TU627
TU628
TU630
TU631
TU632
TU634
TU635
TU636
TU637
TU638
TU640
D082_05495
D082_05520
D082_05560
hypB
D082_05580
520000
522000
524000
526000
528000
530000
purL
D082_05460
birA
D082_05480
menG
D082_05500
recO
D082_05540
D082_05550
lspA
bioY
TU626
TU629
TU633
TU639
TU641
TU642
6400
10000
14400
Untreated
15°C
42°C
−CO_2
Dark
−Fe
HL
−N
−P
Exp. P.
Stat. P.

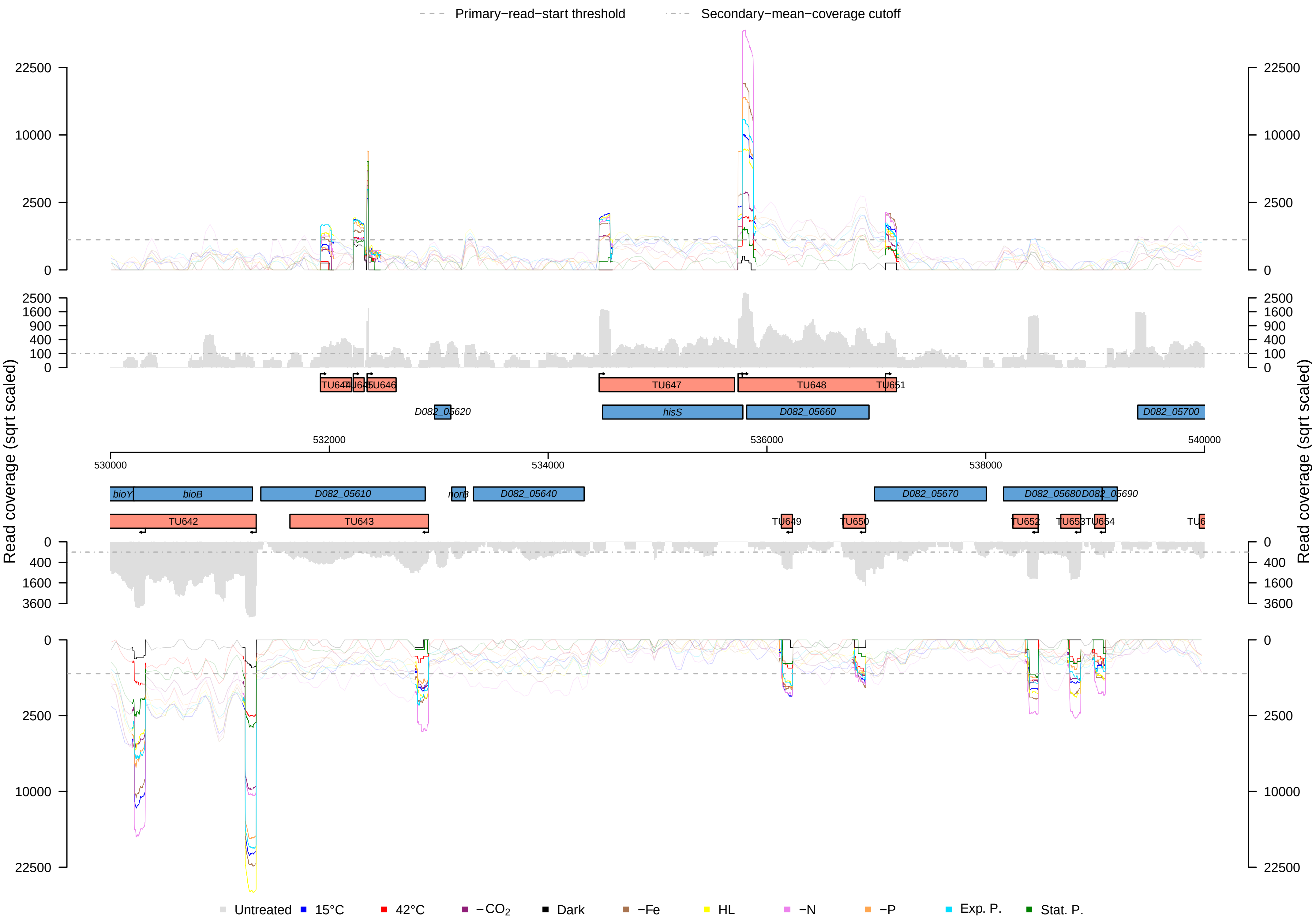

Primary−read−start threshold
Secondary−mean−coverage cutoff
Read coverage (sqrt scaled)
22500
10000
2500
0
1600
900
400
100
3600
TU644
TU645
TU646
TU647
TU648
TU651
D082_05620
hisS
D082_05660
D082_05700
530000
532000
534000
536000
538000
540000
bioY
bioB
D082_05610
norB
D082_05640
D082_05670
D082_05680
D082_05690
TU642
TU643
TU649
TU650
TU652
TU653
TU654
Untreated
15°C
42°C
−CO2
Dark
−Fe
HL
−N
−P
Exp. P.
Stat. P.

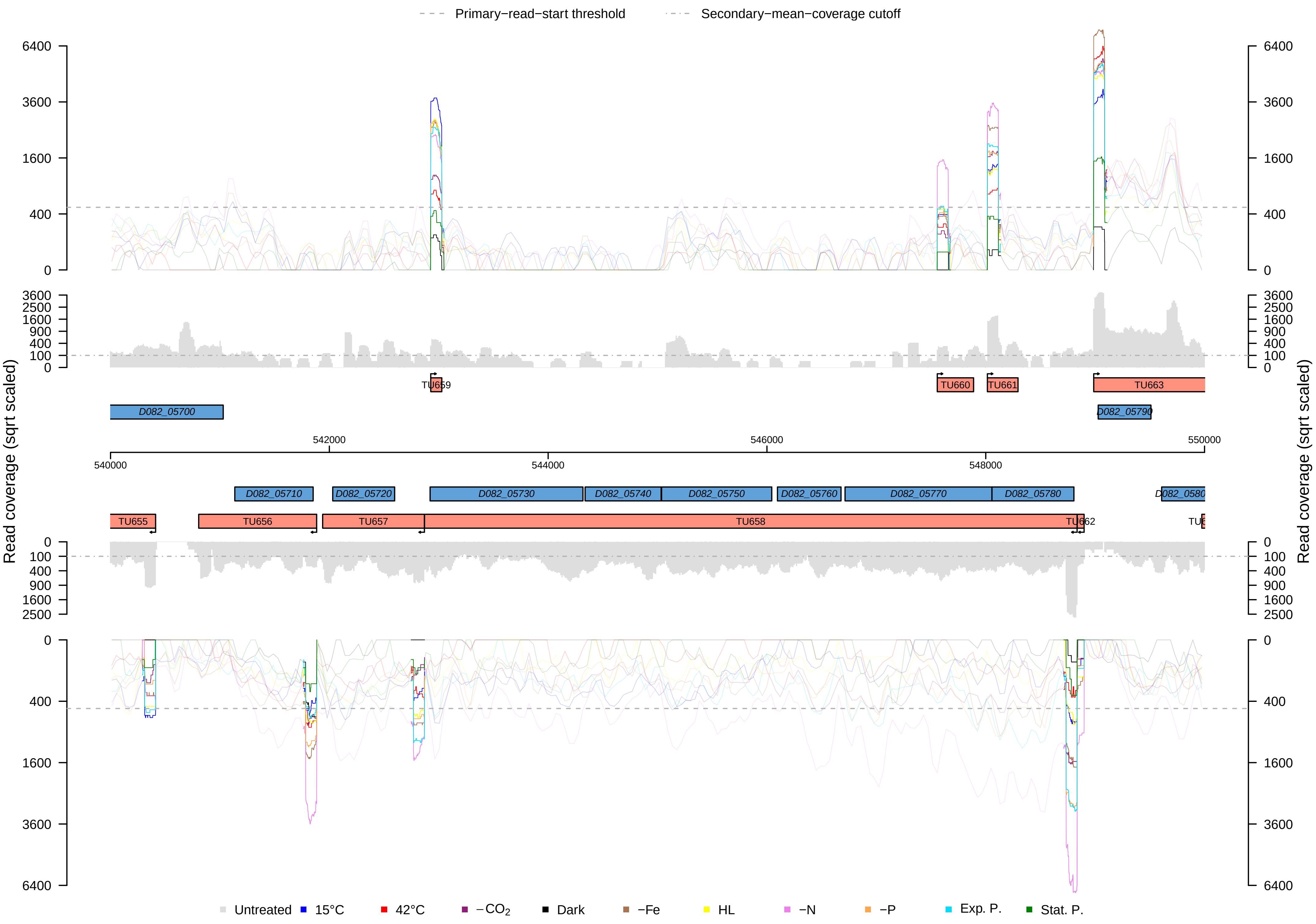

Primary−read−start threshold
Secondary−mean−coverage cutoff
Read coverage (sqrt scaled)
6400
3600
1600
400
0
2500
900
100
TU659
TU660
TU661
TU663
D082_05700
D082_05790
540000
542000
544000
546000
548000
550000
D082_05710
D082_05720
D082_05730
D082_05740
D082_05750
D082_05760
D082_05770
D082_05780
TU655
TU656
TU657
TU658
TU662
Untreated
15°C
42°C
−CO2
Dark
−Fe
HL
−N
−P
Exp. P.
Stat. P.

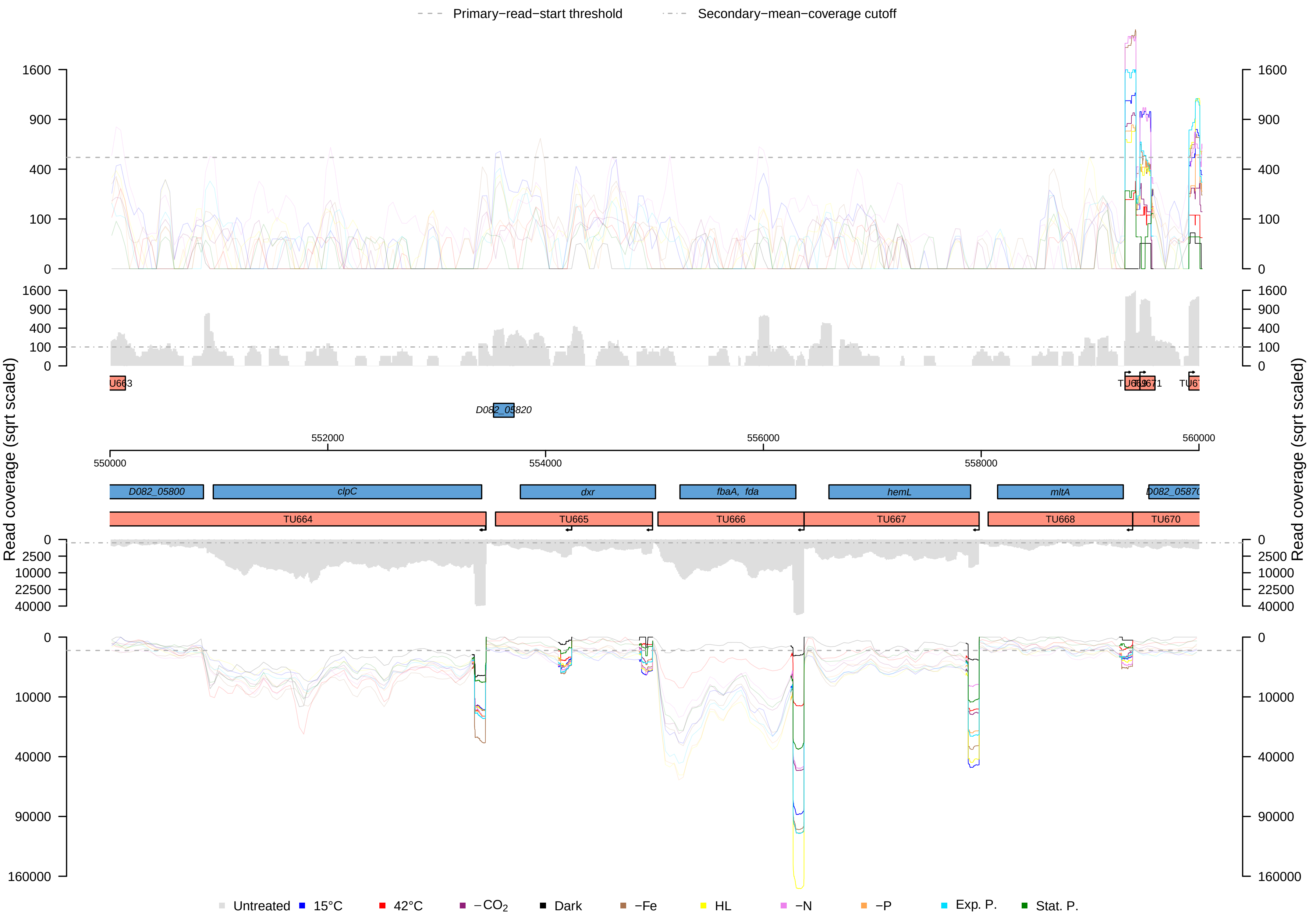

Primary−read−start threshold
Secondary−mean−coverage cutoff
Read coverage (sqrt scaled)
1600
900
400
100
0
2500
10000
22500
40000
90000
160000
D082_05820
550000
552000
554000
556000
558000
560000
D082_05800
clpC
dxr
fbaA, fda
hemL
mltA
TU664
TU665
TU666
TU667
TU668
TU670
Untreated
15°C
42°C
−CO2
Dark
−Fe
HL
−N
−P
Exp. P.
Stat. P.

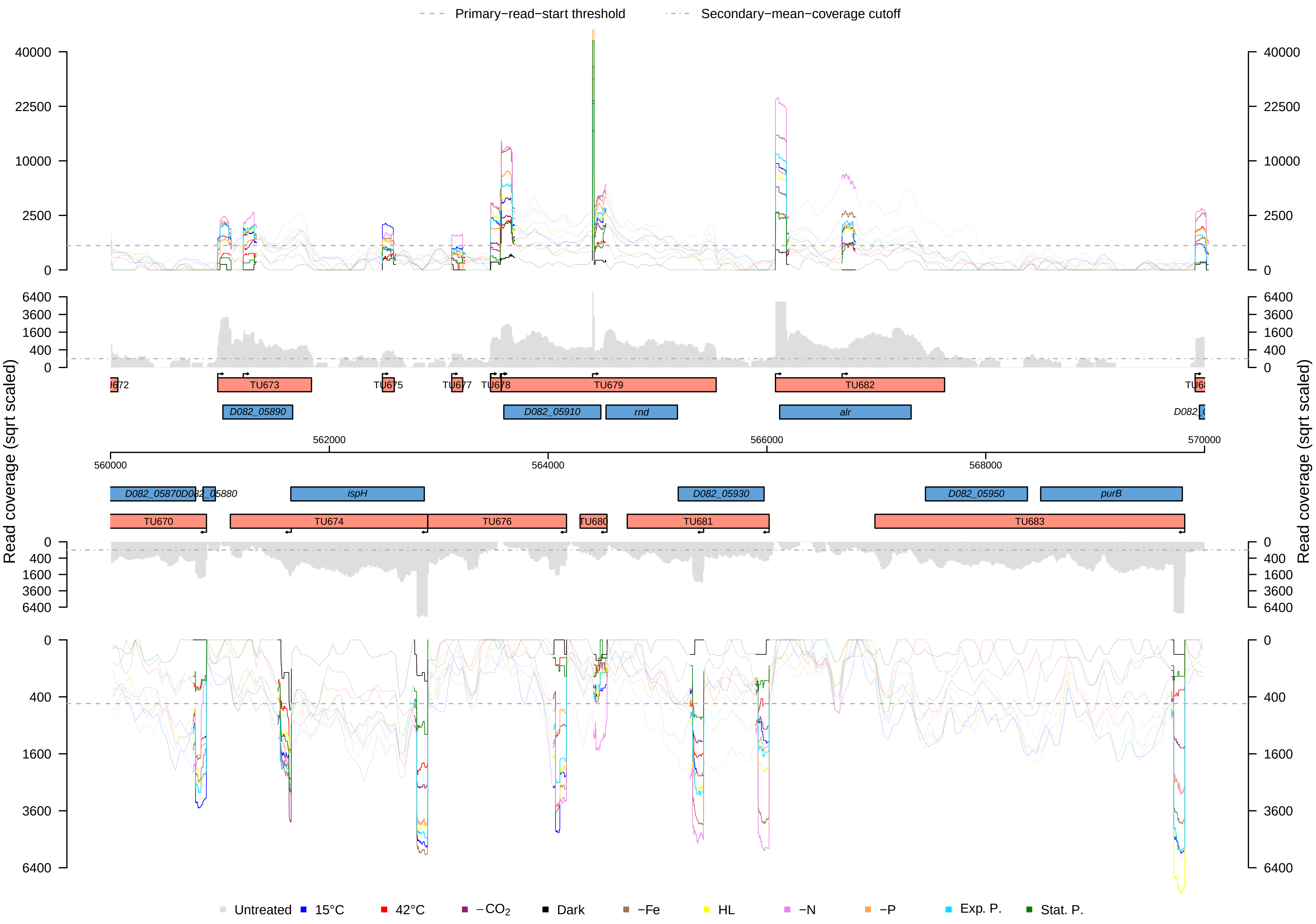

Primary−read−start threshold
Secondary−mean−coverage cutoff
Read coverage (sqrt scaled)
40000
22500
10000
2500
0
6400
3600
1600
400
TU673
TU675
TU677
TU678
TU679
TU682
D082_05890
D082_05910
rnd
alr
560000
562000
564000
566000
568000
570000
D082_05870
D082_05880
ispH
D082_05930
D082_05950
purB
TU670
TU674
TU676
TU680
TU681
TU683
Untreated
15°C
42°C
−CO2
Dark
−Fe
HL
−N
−P
Exp. P.
Stat. P.

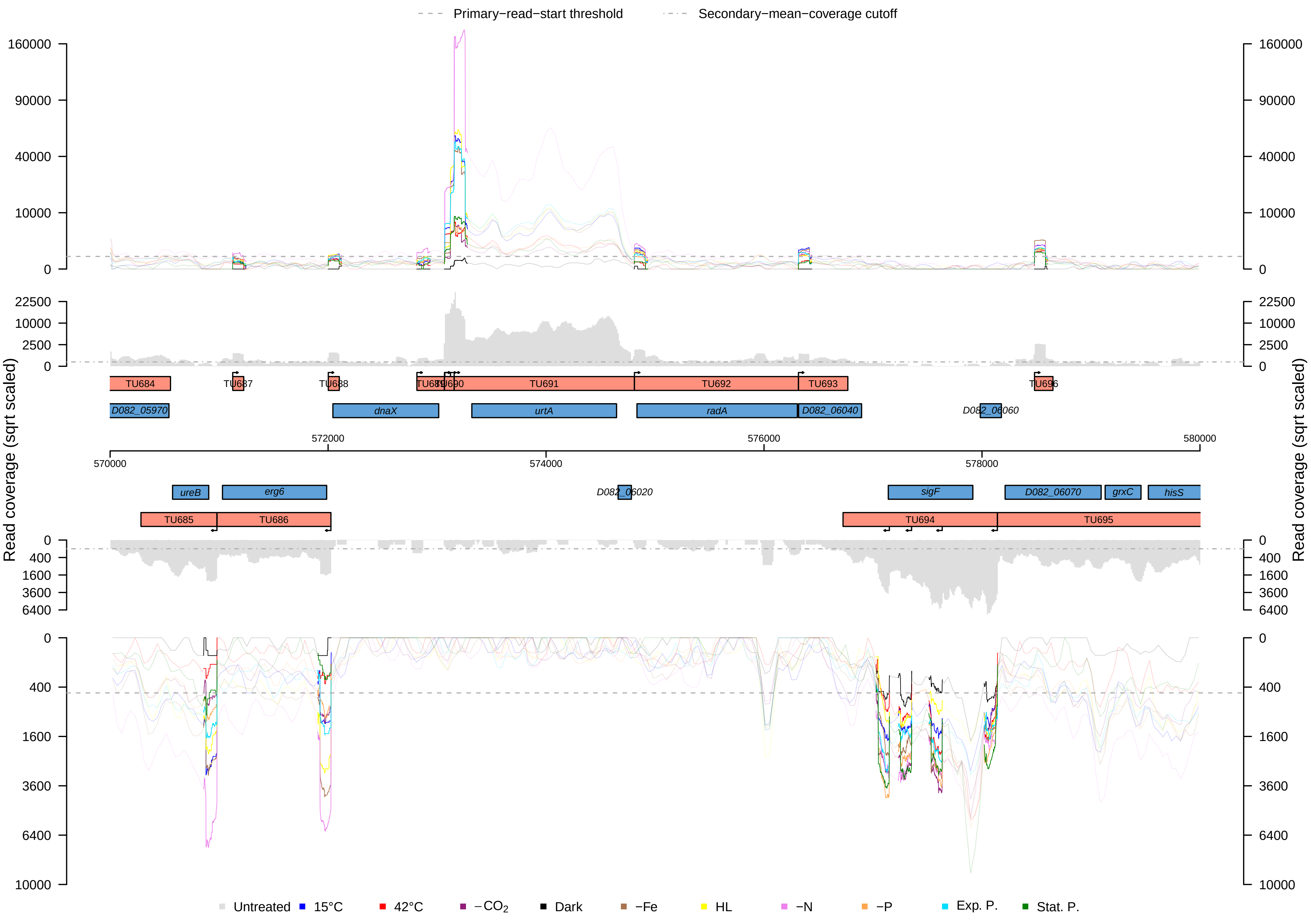
Primary−read−start threshold
Secondary−mean−coverage cutoff
160000
90000
40000
10000
0
22500
10000
2500
0
Read coverage (sqrt scaled)
TU684
TU687
TU688
TU689
TU690
TU691
TU692
TU693
TU696
D082_05970
dnaX
urtA
radA
D082_06040
D082_06060
570000
572000
574000
576000
578000
580000
ureB
erg6
D082_06020
sigF
D082_06070
grxC
hisS
TU685
TU686
TU694
TU695
0
400
1600
3600
6400
10000
Untreated
15°C
42°C
−CO2
Dark
−Fe
HL
−N
−P
Exp. P.
Stat. P.

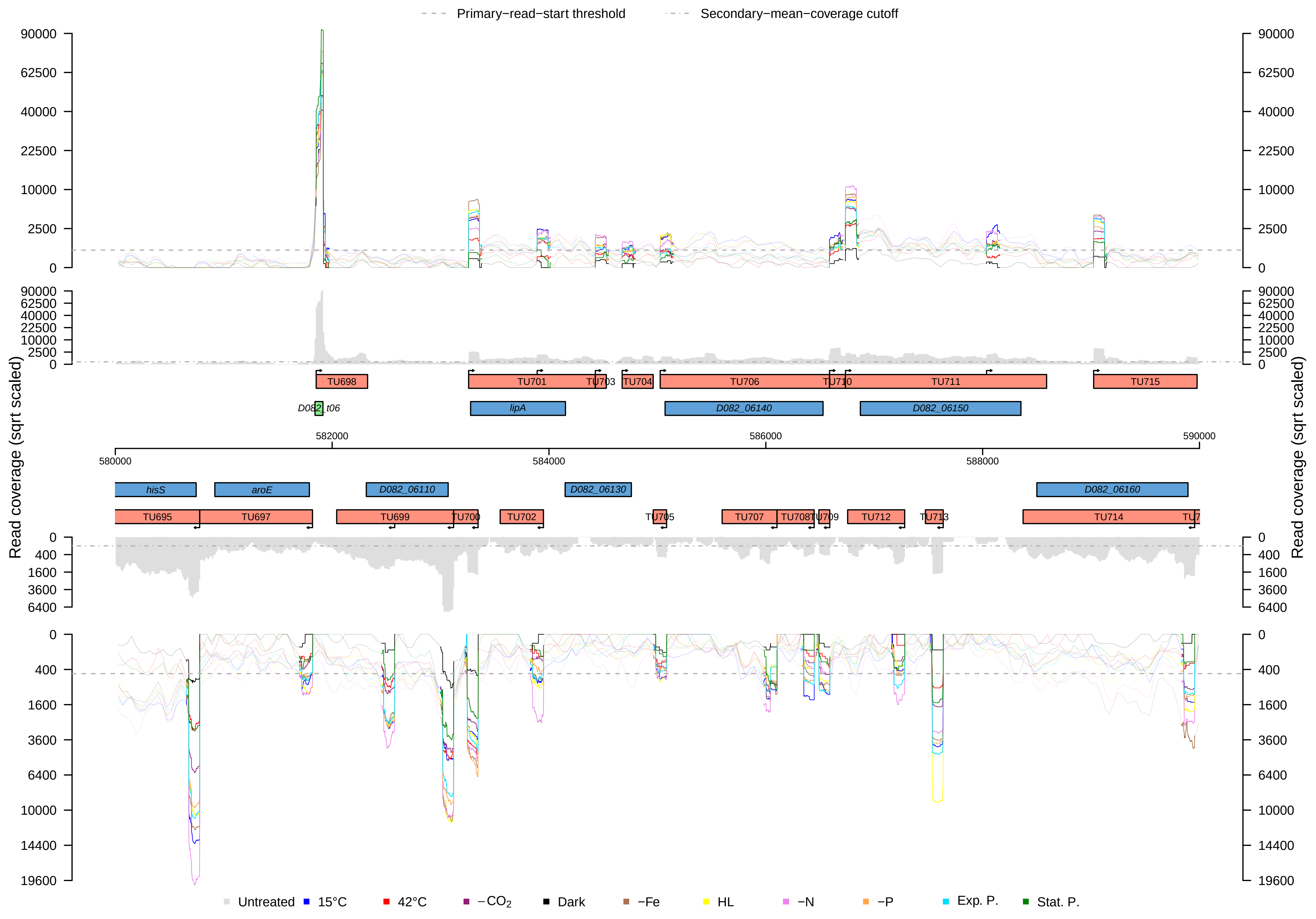

Primary−read−start threshold
Secondary−mean−coverage cutoff
Read coverage (sqrt scaled)
90000
62500
40000
22500
10000
2500
0
400
1600
3600
6400
14400
19600
TU698
TU701
TU703
TU704
TU706
TU710
TU711
TU715
D082_t06
lipA
D082_06140
D082_06150
580000
582000
584000
586000
588000
590000
hisS
aroE
D082_06110
D082_06130
D082_06160
TU695
TU697
TU699
TU700
TU702
TU705
TU707
TU708
TU709
TU712
TU713
TU714
Untreated
15°C
42°C
−CO2
Dark
−Fe
HL
−N
−P
Exp. P.
Stat. P.

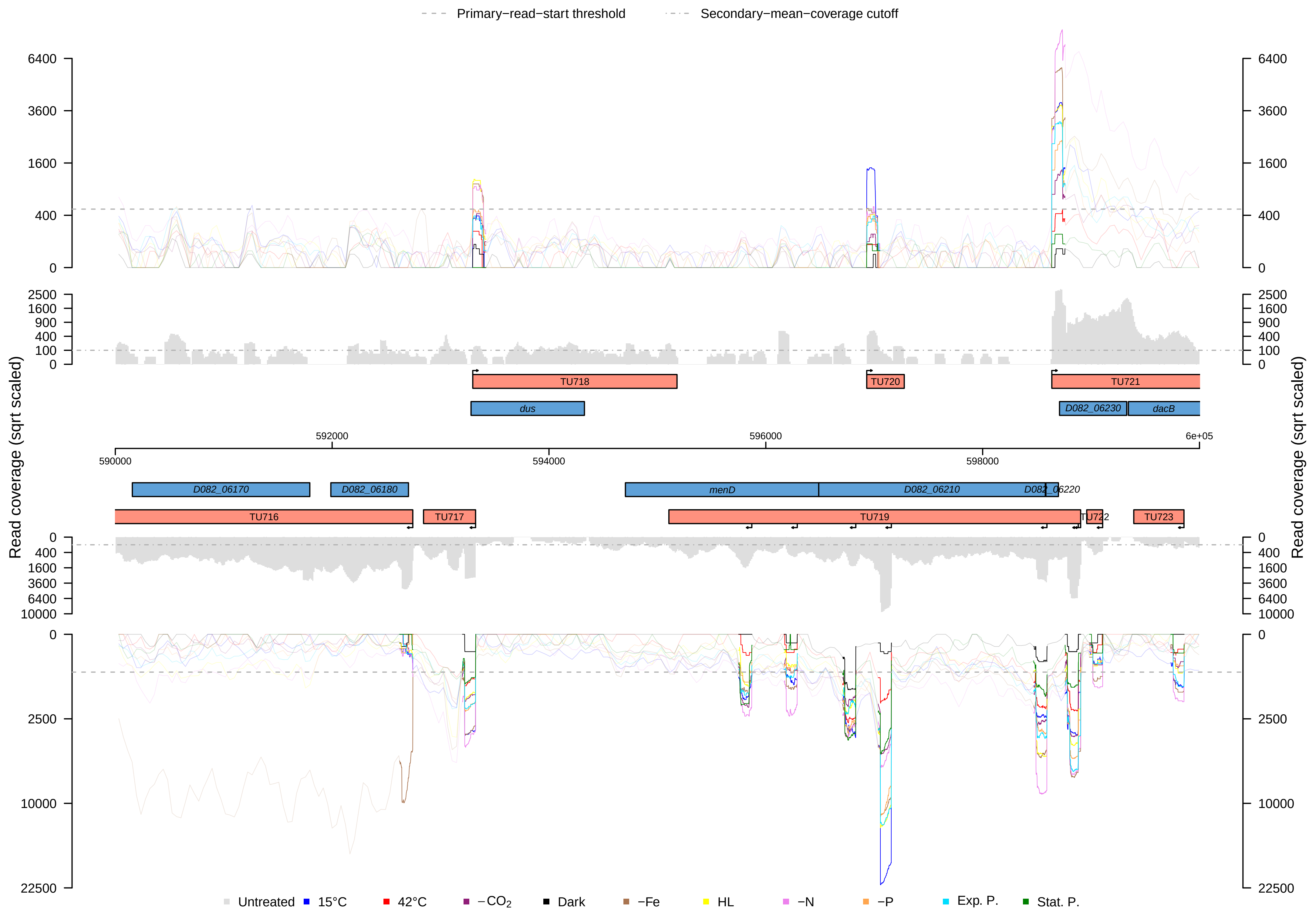

Primary−read−start threshold
Secondary−mean−coverage cutoff
Read coverage (sqrt scaled)
6400
3600
1600
400
0
2500
1600
900
400
100
0
TU718
TU720
TU721
dus
D082_06230
dacB
590000
592000
594000
596000
598000
6e+05
D082_06170
D082_06180
menD
D082_06210
D082_06220
TU716
TU717
TU719
TU722
TU723
400
1600
3600
6400
10000
2500
10000
22500
Untreated
15°C
42°C
−CO2
Dark
−Fe
HL
−N
−P
Exp. P.
Stat. P.

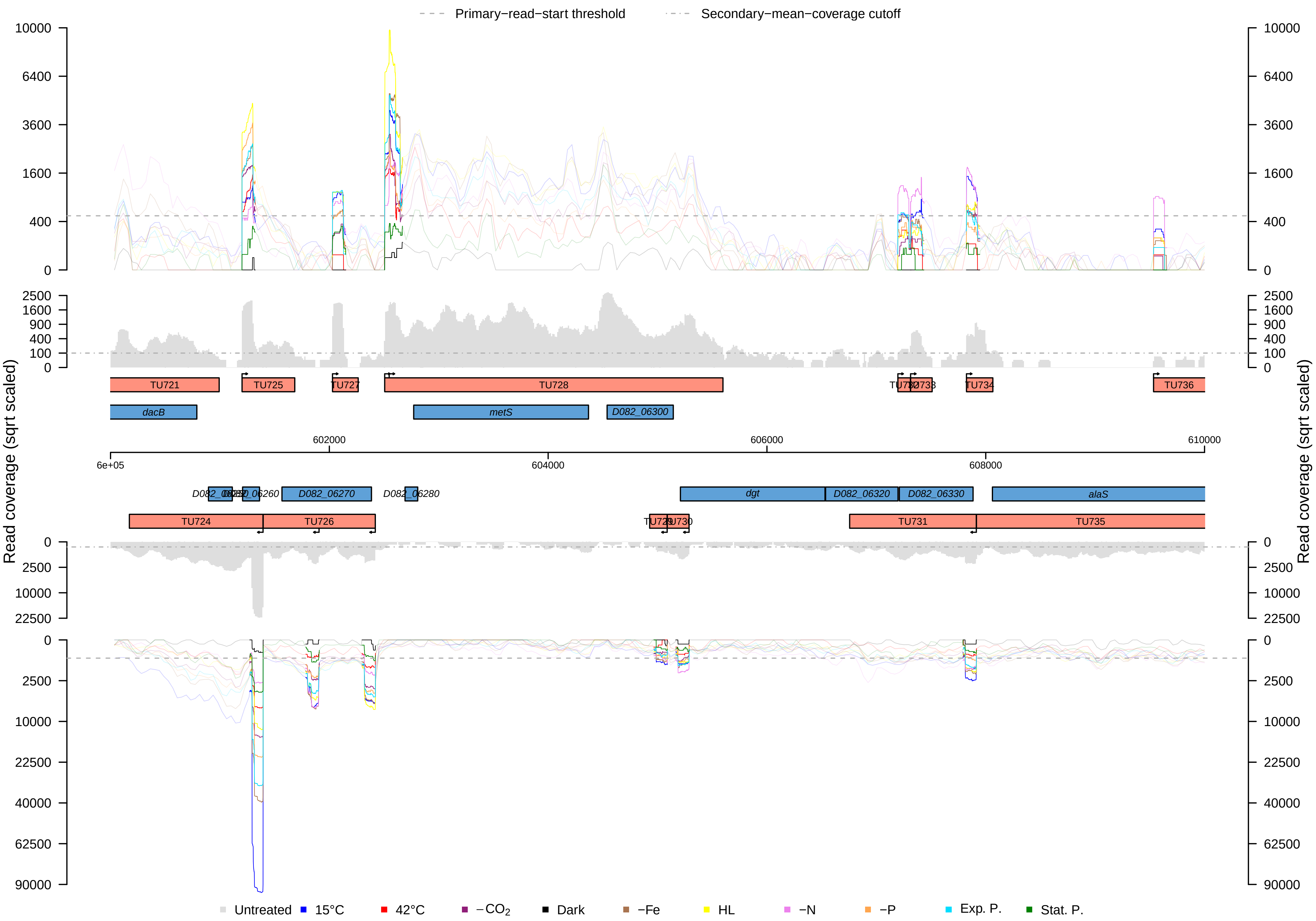
Primary−read−start threshold
Secondary−mean−coverage cutoff
Read coverage (sqrt scaled)
TU721
TU725
TU727
TU728
TU734
TU736
dacB
metS
D082_06300
6e+05
602000
604000
606000
608000
610000
D082_06270
D082_06280
dgt
D082_06320
D082_06330
alaS
TU724
TU726
TU731
TU735
Untreated
15°C
42°C
−CO2
Dark
−Fe
HL
−N
−P
Exp. P.
Stat. P.

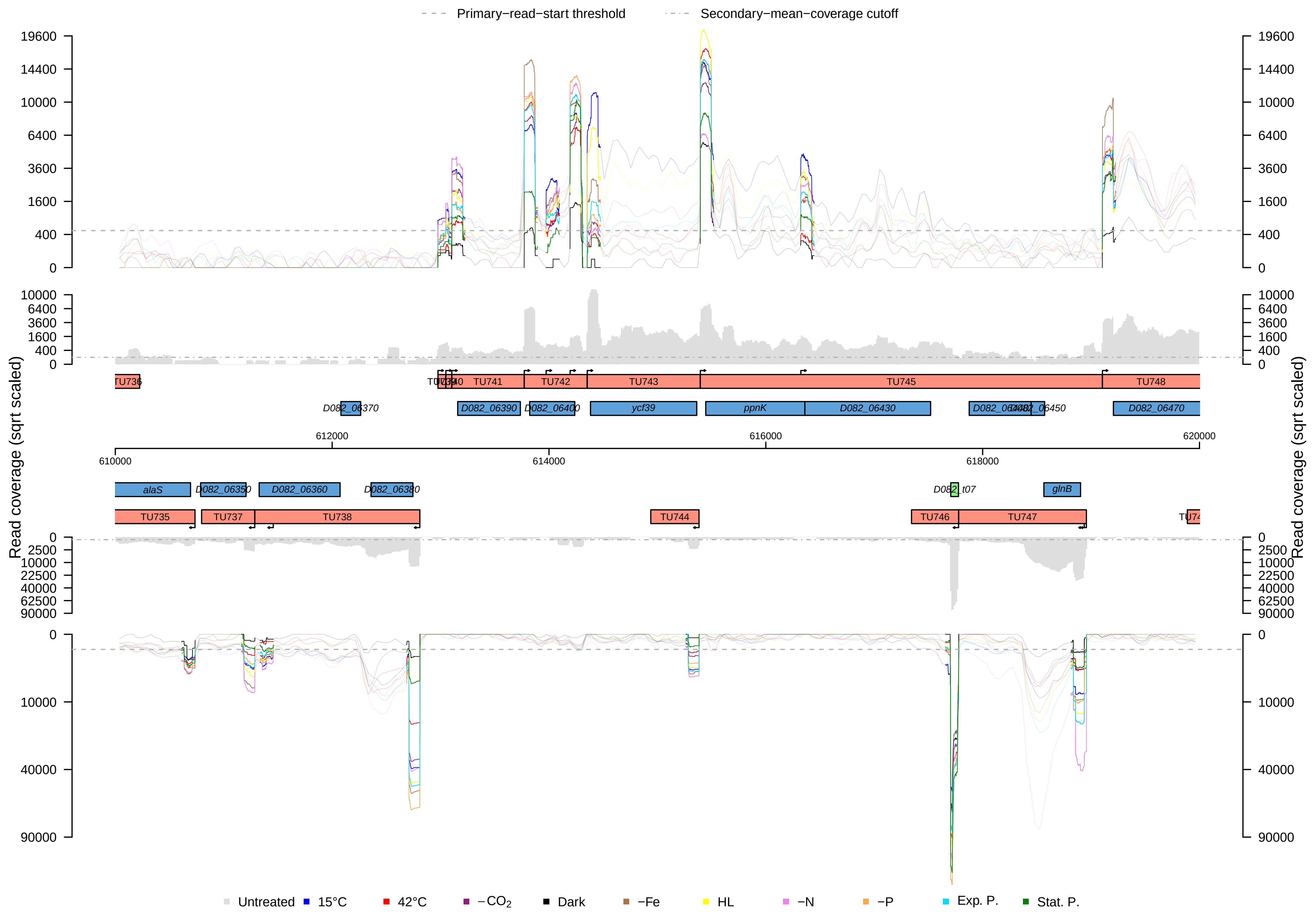

Primary−read−start threshold
Secondary−mean−coverage cutoff
Read coverage (sqrt scaled)
TU736
TU739
TU740
TU741
TU742
TU743
TU745
TU748
D082_06370
D082_06390
D082_06400
ycf39
ppnK
D082_06430
D082_06440
D082_06450
D082_06470
610000
612000
614000
616000
618000
620000
alaS
D082_06350
D082_06360
D082_06380
D082_t07
glnB
TU735
TU737
TU738
TU744
TU746
TU747
Untreated
15°C
42°C
−CO2
Dark
−Fe
HL
−N
−P
Exp. P.
Stat. P.

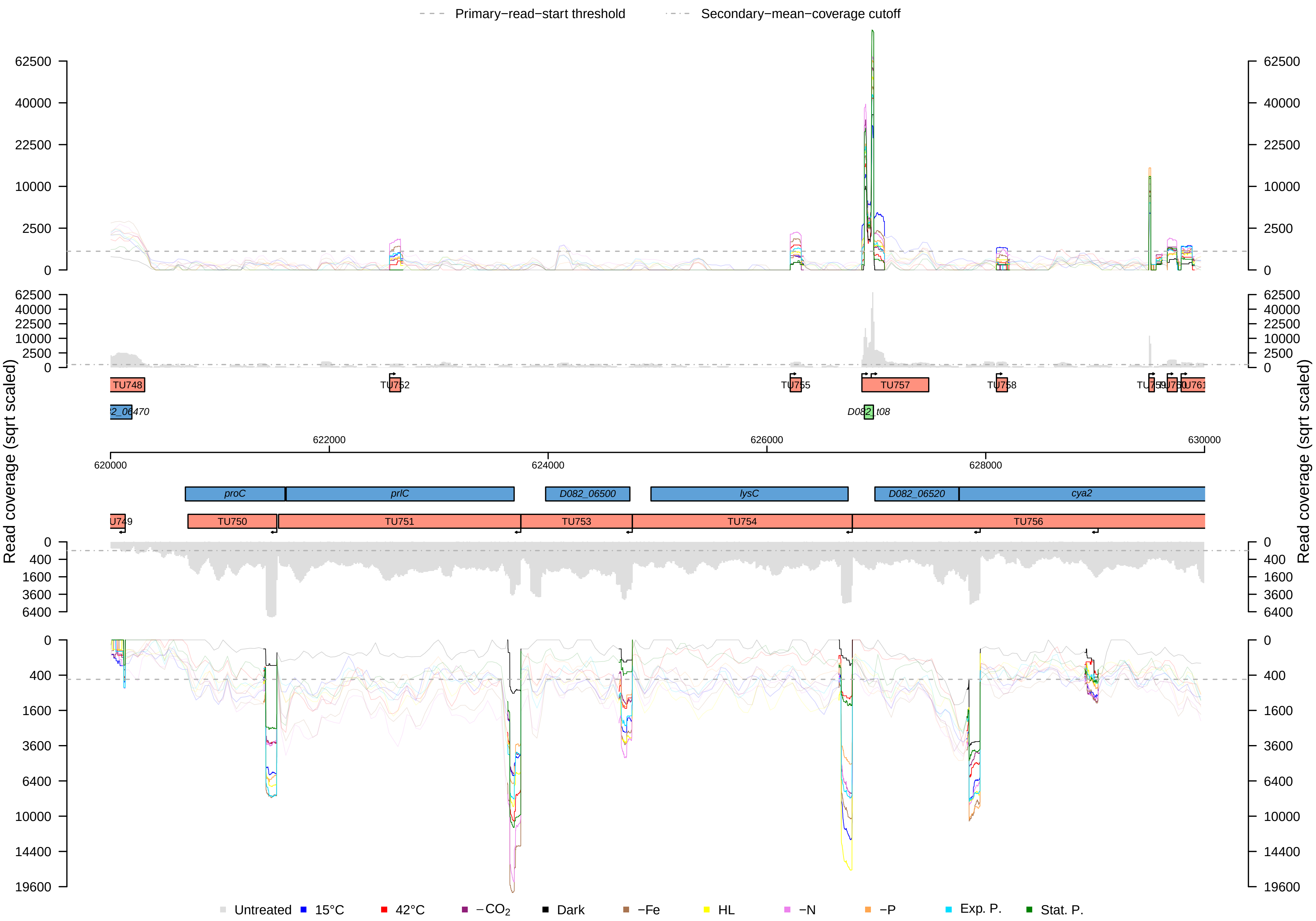

Primary−read−start threshold
Secondary−mean−coverage cutoff
Read coverage (sqrt scaled)
62500
40000
22500
10000
2500
0
400
1600
3600
6400
14400
19600
TU748
TU752
TU755
TU757
TU758
TU761
D082_06470
620000
622000
624000
626000
628000
630000
proC
prlC
D082_06500
lysC
D082_06520
cya2
TU749
TU750
TU751
TU753
TU754
TU756
Untreated
15°C
42°C
−CO_2
Dark
−Fe
HL
−N
−P
Exp. P.
Stat. P.

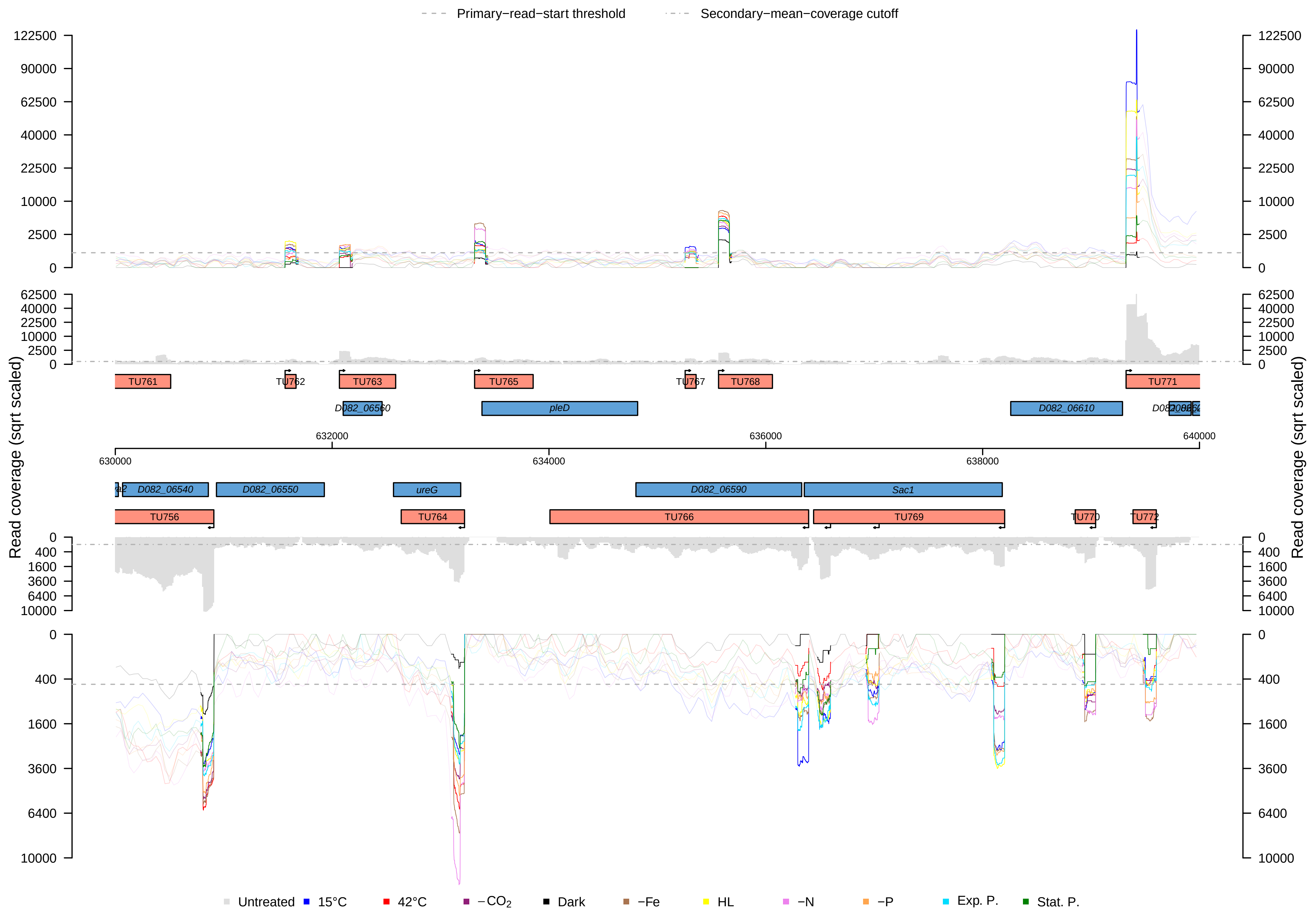

Primary−read−start threshold
Secondary−mean−coverage cutoff
Read coverage (sqrt scaled)
122500
90000
62500
40000
22500
10000
2500
0
62500
40000
22500
10000
2500
0
TU761
TU762
TU763
TU765
TU767
TU768
TU771
D082_06560
pleD
D082_06610
632000
636000
640000
630000
634000
638000
D082_06540
D082_06550
ureG
D082_06590
Sac1
TU756
TU764
TU766
TU769
TU770
TU772
400
1600
3600
6400
10000
Untreated
15°C
42°C
−CO_2
Dark
−Fe
HL
−N
−P
Exp. P.
Stat. P.

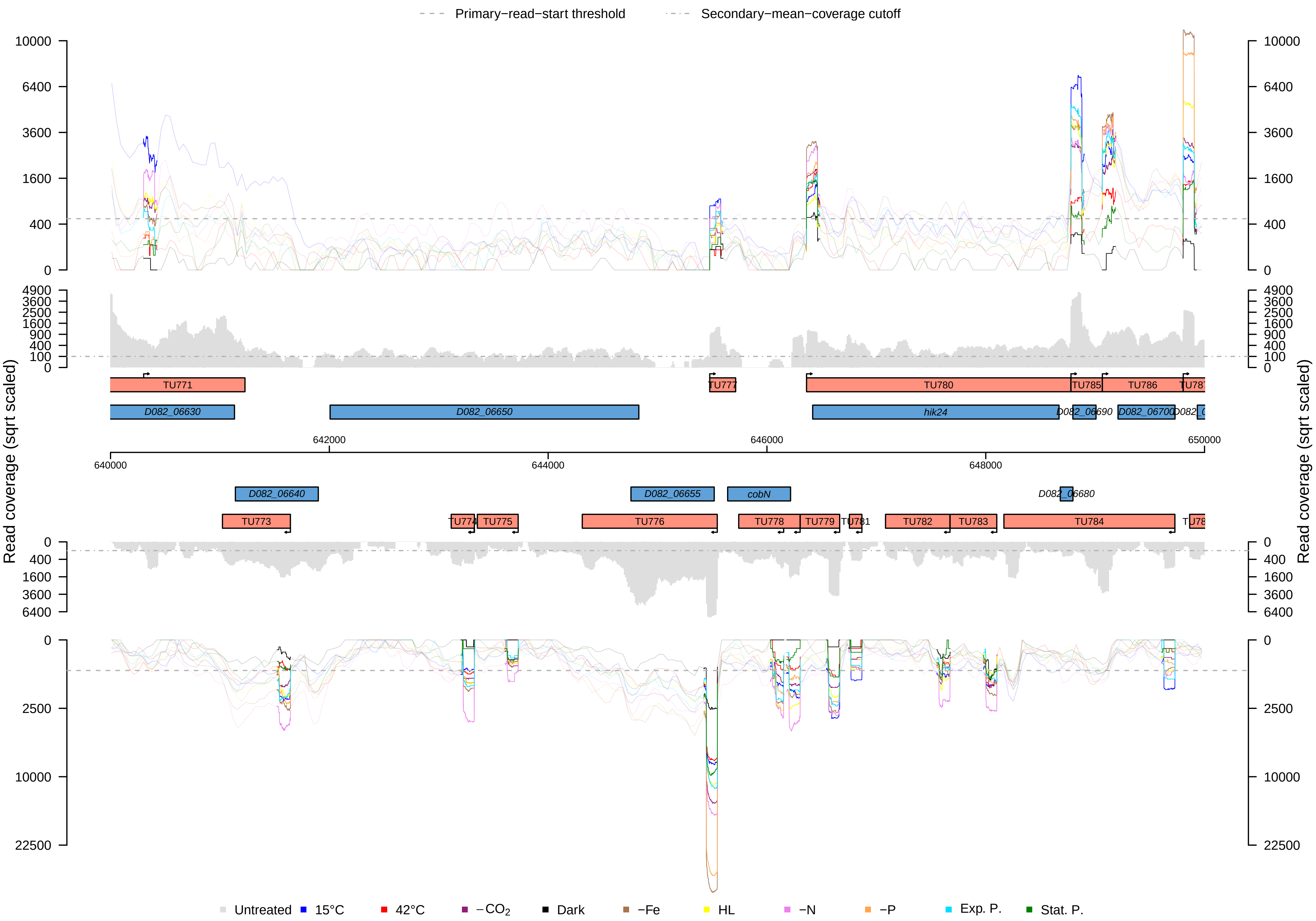

Primary−read−start threshold
Secondary−mean−coverage cutoff
Read coverage (sqrt scaled)
10000
6400
3600
1600
400
0
4900
3600
2500
1600
900
400
100
0
TU771
TU777
TU780
TU785
TU786
TU787
D082_06630
D082_06650
hik24
D082_06690
D082_06700
640000
642000
644000
646000
648000
650000
D082_06640
D082_06655
cobN
D082_06680
TU773
TU774
TU775
TU776
TU778
TU779
TU781
TU782
TU783
TU784
2500
10000
22500
Untreated
15°C
42°C
−CO_2
Dark
−Fe
HL
−N
−P
Exp. P.
Stat. P.

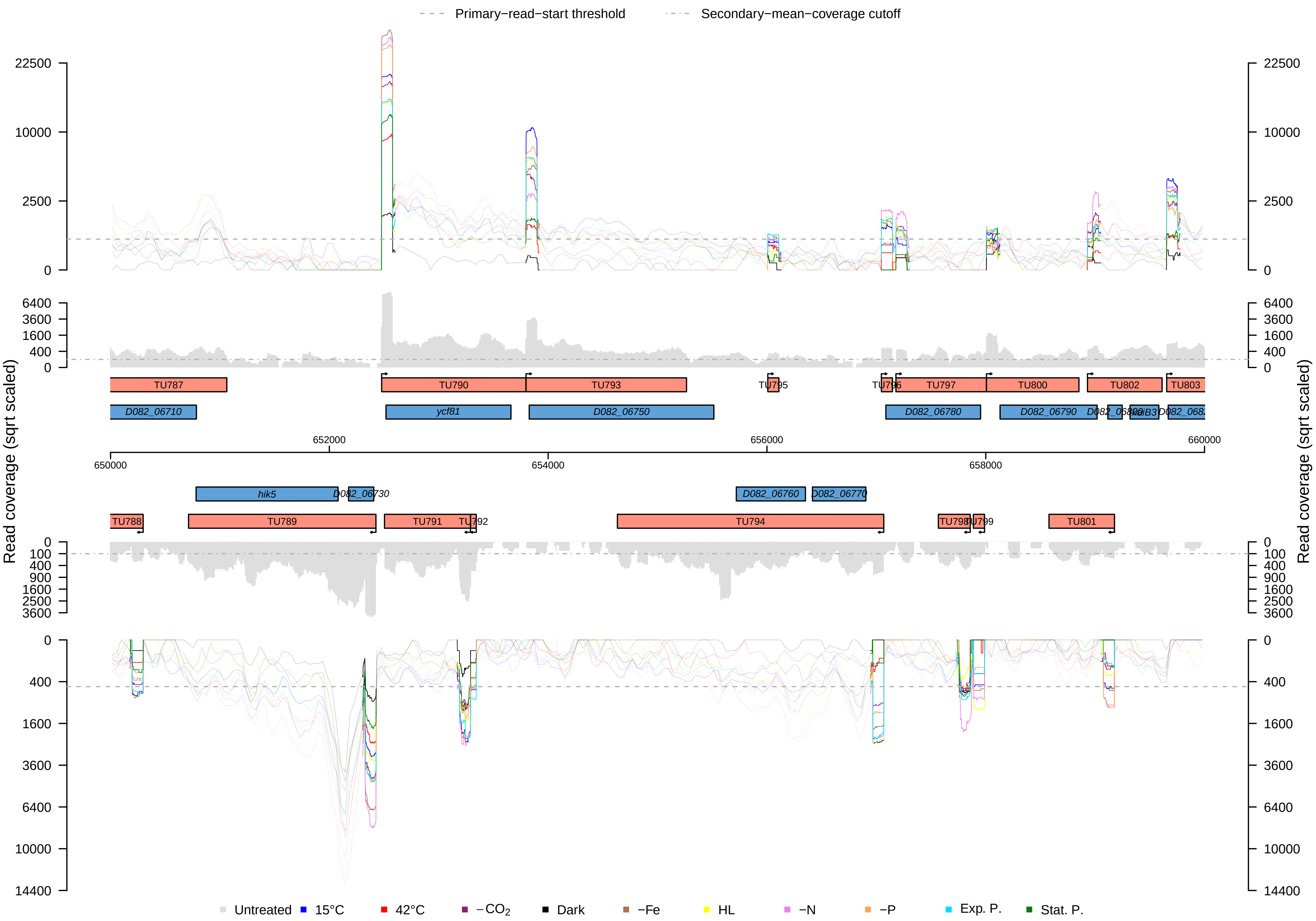
Primary−read−start threshold
Secondary−mean−coverage cutoff
Read coverage (sqrt scaled)
TU787
TU790
TU793
TU795
TU796
TU797
TU800
TU802
TU803
D082_06710
ycf81
D082_06750
D082_06780
D082_06790
hik5
D082_06730
D082_06760
D082_06770
TU788
TU789
TU791
TU792
TU794
TU798
TU799
TU801
650000
652000
654000
656000
658000
660000
Untreated
15°C
42°C
−CO2
Dark
−Fe
HL
−N
−P
Exp. P.
Stat. P.

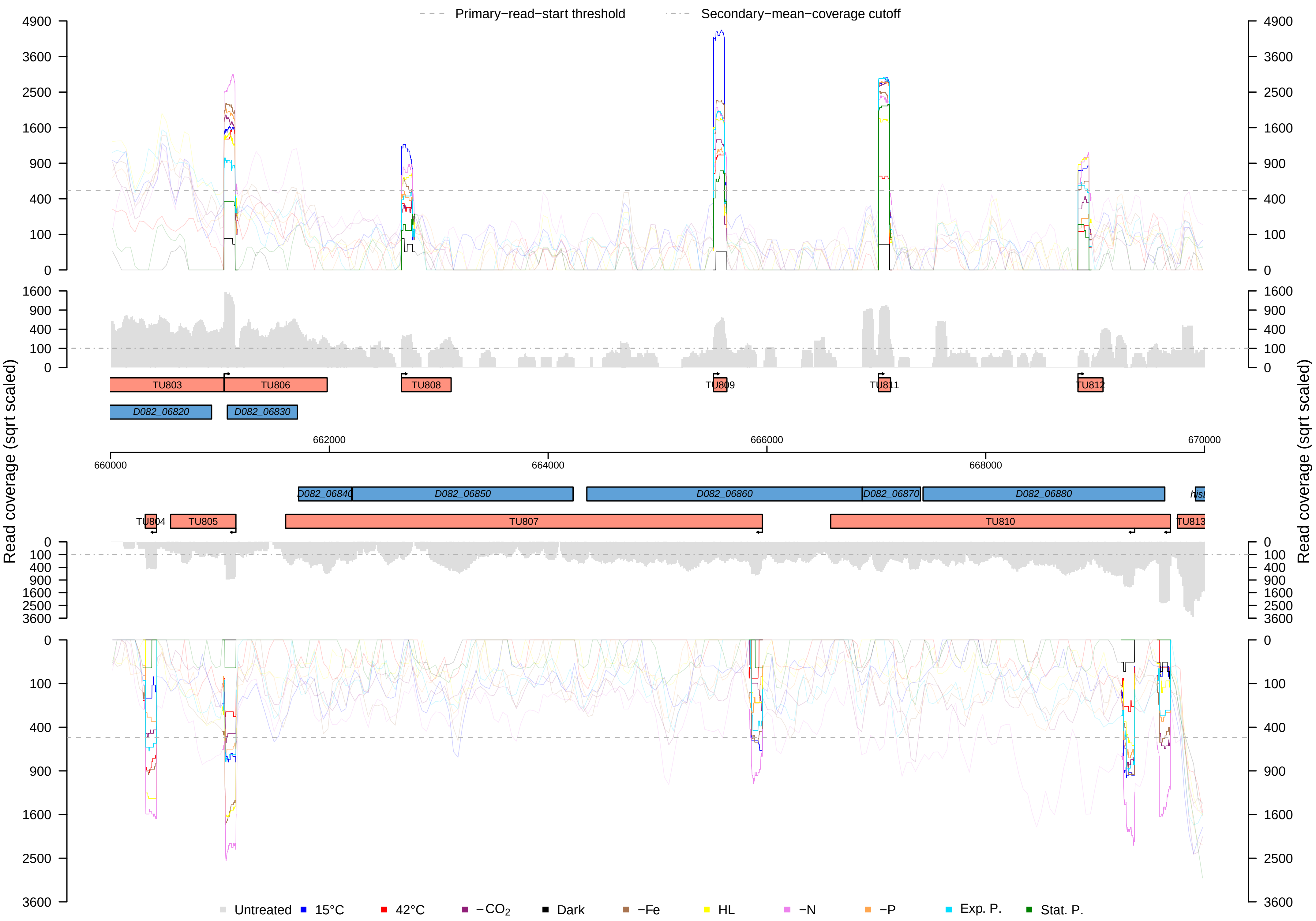

Primary−read−start threshold
Secondary−mean−coverage cutoff
Read coverage (sqrt scaled)
4900
3600
2500
1600
900
400
100
0
TU803
TU806
TU808
TU809
TU811
TU812
D082_06820
D082_06830
660000
662000
664000
666000
668000
670000
D082_06840
D082_06850
D082_06860
D082_06870
D082_06880
TU804
TU805
TU807
TU810
TU813
Untreated
15°C
42°C
−CO2
Dark
−Fe
HL
−N
−P
Exp. P.
Stat. P.

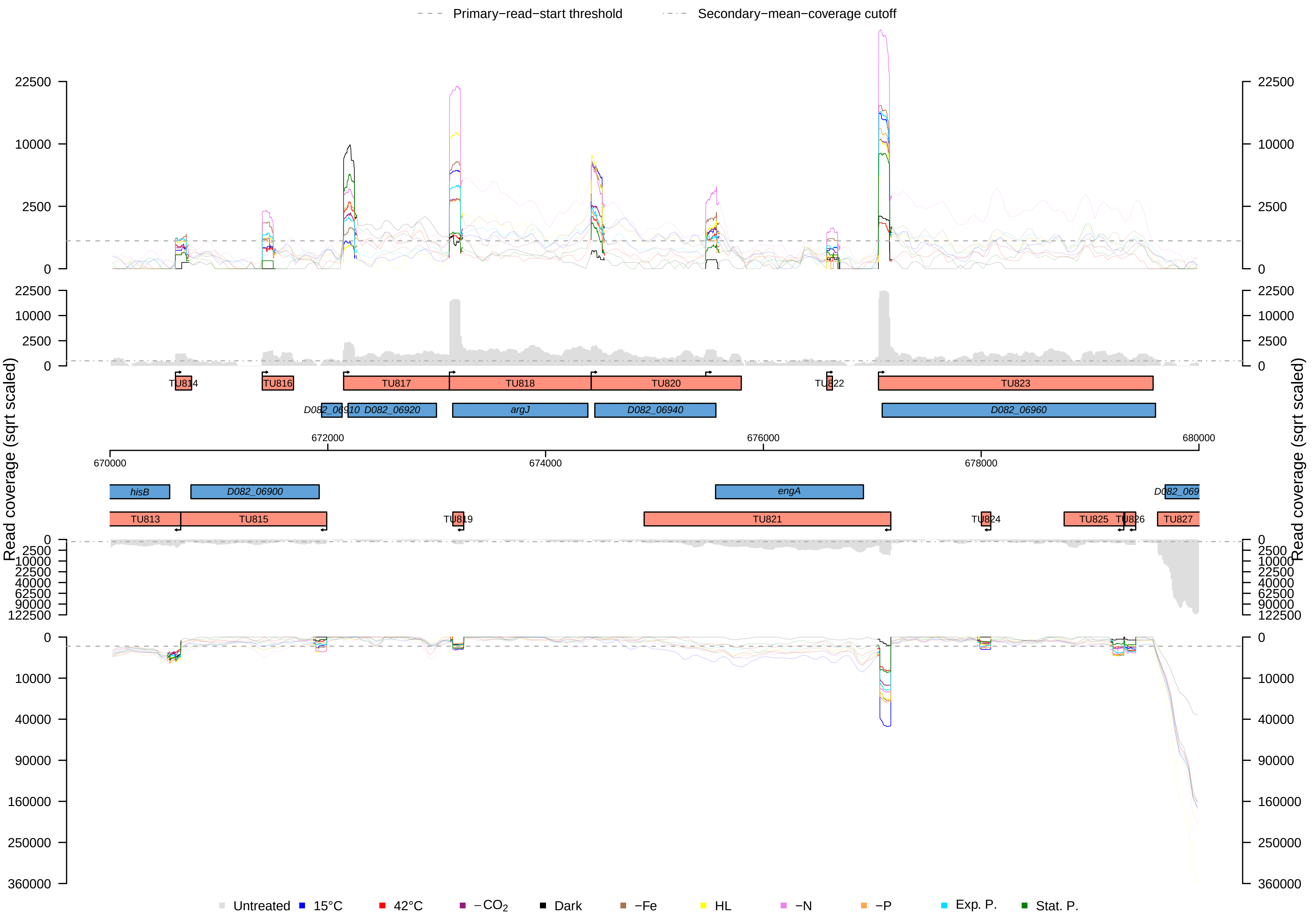

Primary−read−start threshold
Secondary−mean−coverage cutoff
Read coverage (sqrt scaled)
22500
10000
2500
0
TU813
TU814
TU815
TU816
TU817
TU818
TU819
TU820
TU821
TU822
TU823
TU824
TU825
TU826
TU827
hisB
D082_06900
D082_06910
D082_06920
argJ
D082_06940
engA
D082_06960
D082_069
670000
672000
674000
676000
678000
680000
40000
62500
90000
122500
160000
250000
360000
Untreated
15°C
42°C
−CO2
Dark
−Fe
HL
−N
−P
Exp. P.
Stat. P.

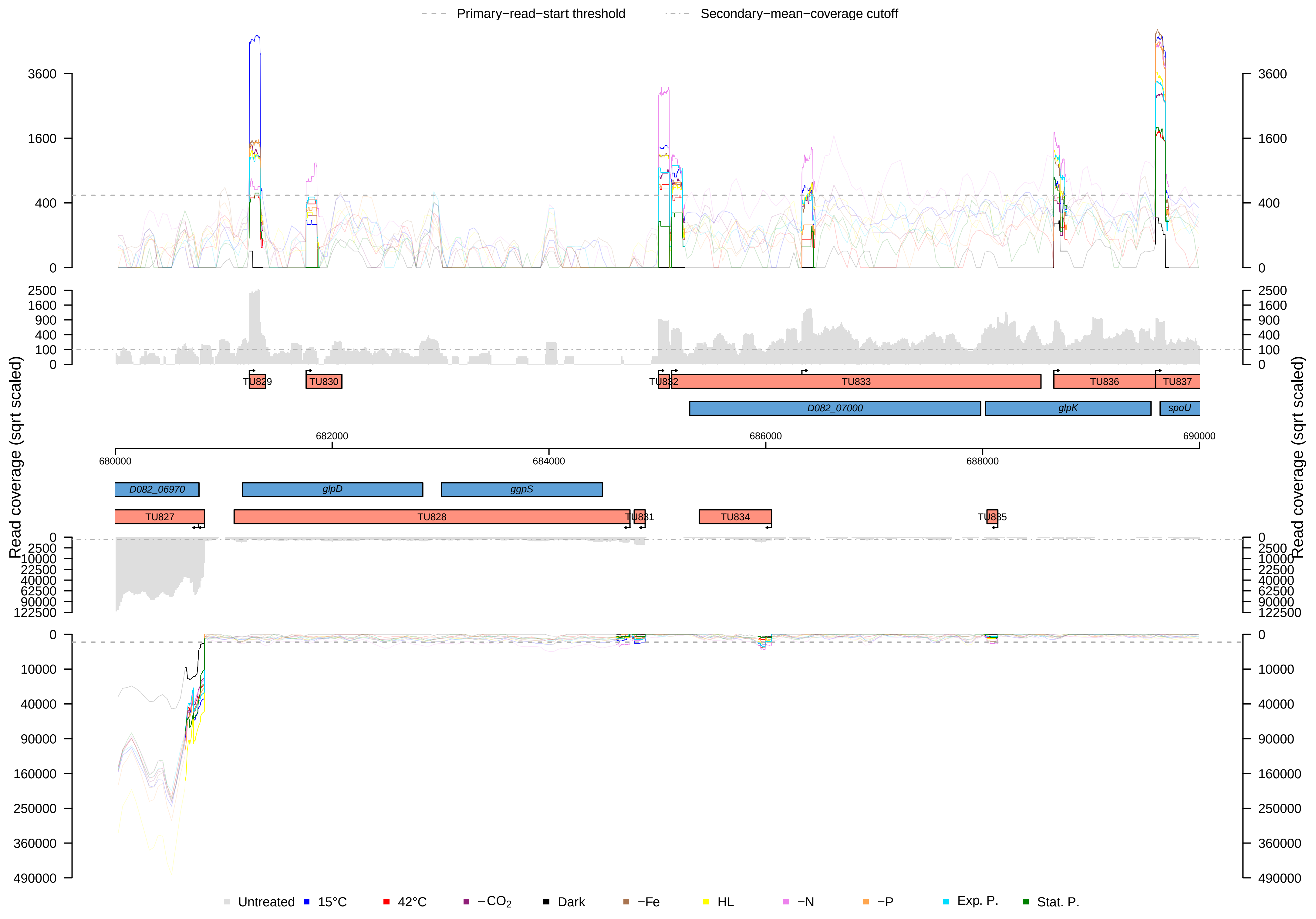

Primary−read−start threshold
Secondary−mean−coverage cutoff
Read coverage (sqrt scaled)
3600
1600
400
0
2500
1600
900
400
100
0
TU829
TU830
TU832
TU833
TU836
TU837
D082_07000
glpK
spoU
682000
686000
690000
680000
684000
688000
D082_06970
glpD
ggpS
TU827
TU828
TU831
TU834
TU835
2500
10000
22500
40000
62500
90000
122500
10000
40000
90000
160000
250000
360000
490000
Untreated
15°C
42°C
−CO2
Dark
−Fe
HL
−N
−P
Exp. P.
Stat. P.

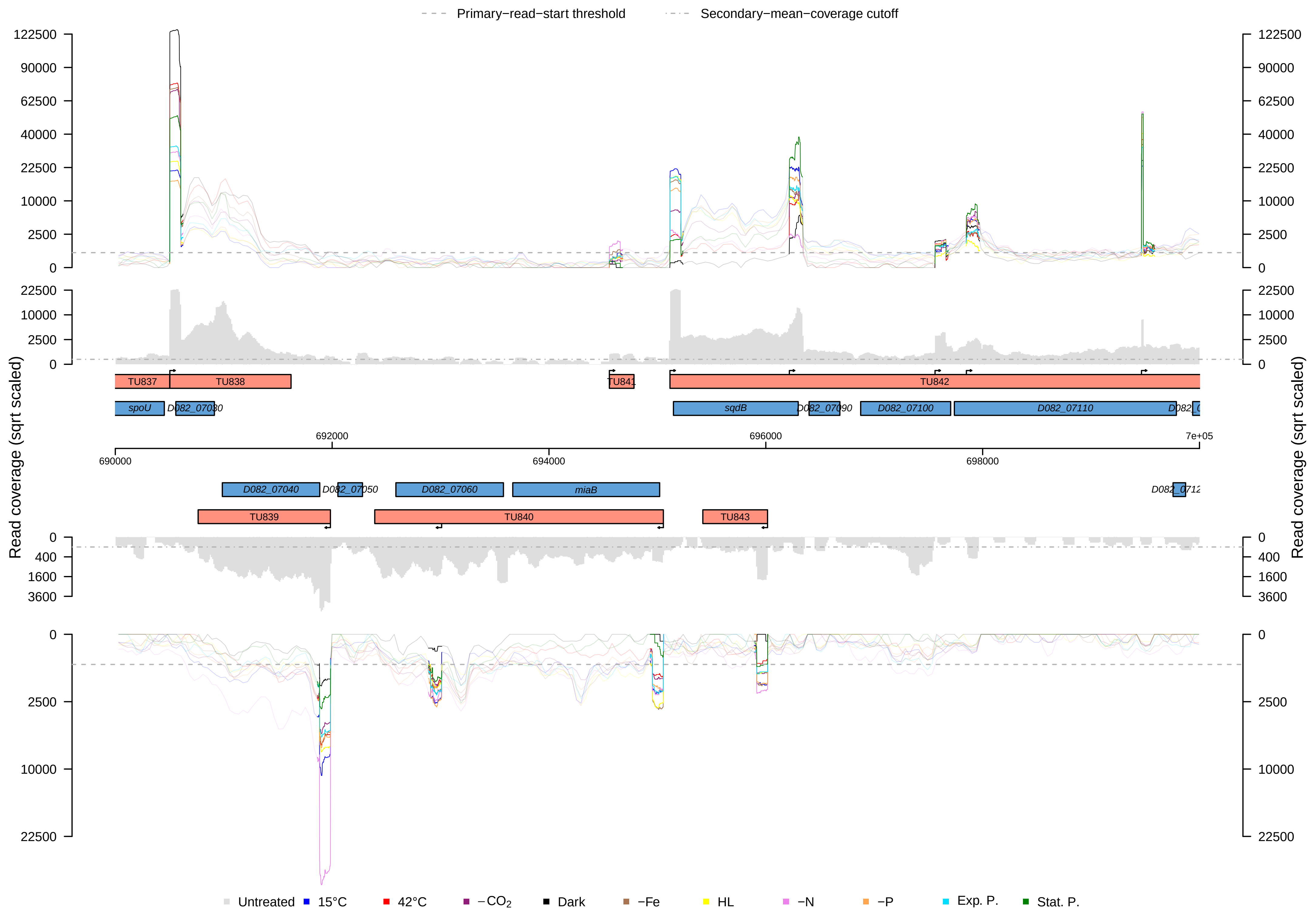

Primary−read−start threshold
Secondary−mean−coverage cutoff
Read coverage (sqrt scaled)
122500
90000
62500
40000
22500
10000
2500
0
400
1600
3600
TU837
TU838
TU839
TU840
TU841
TU842
TU843
spoU
D082_07030
D082_07040
D082_07050
D082_07060
miaB
sqdB
D082_07090
D082_07100
D082_07110
690000
692000
694000
696000
698000
7e+05
Untreated
15°C
42°C
−CO2
Dark
−Fe
HL
−N
−P
Exp. P.
Stat. P.

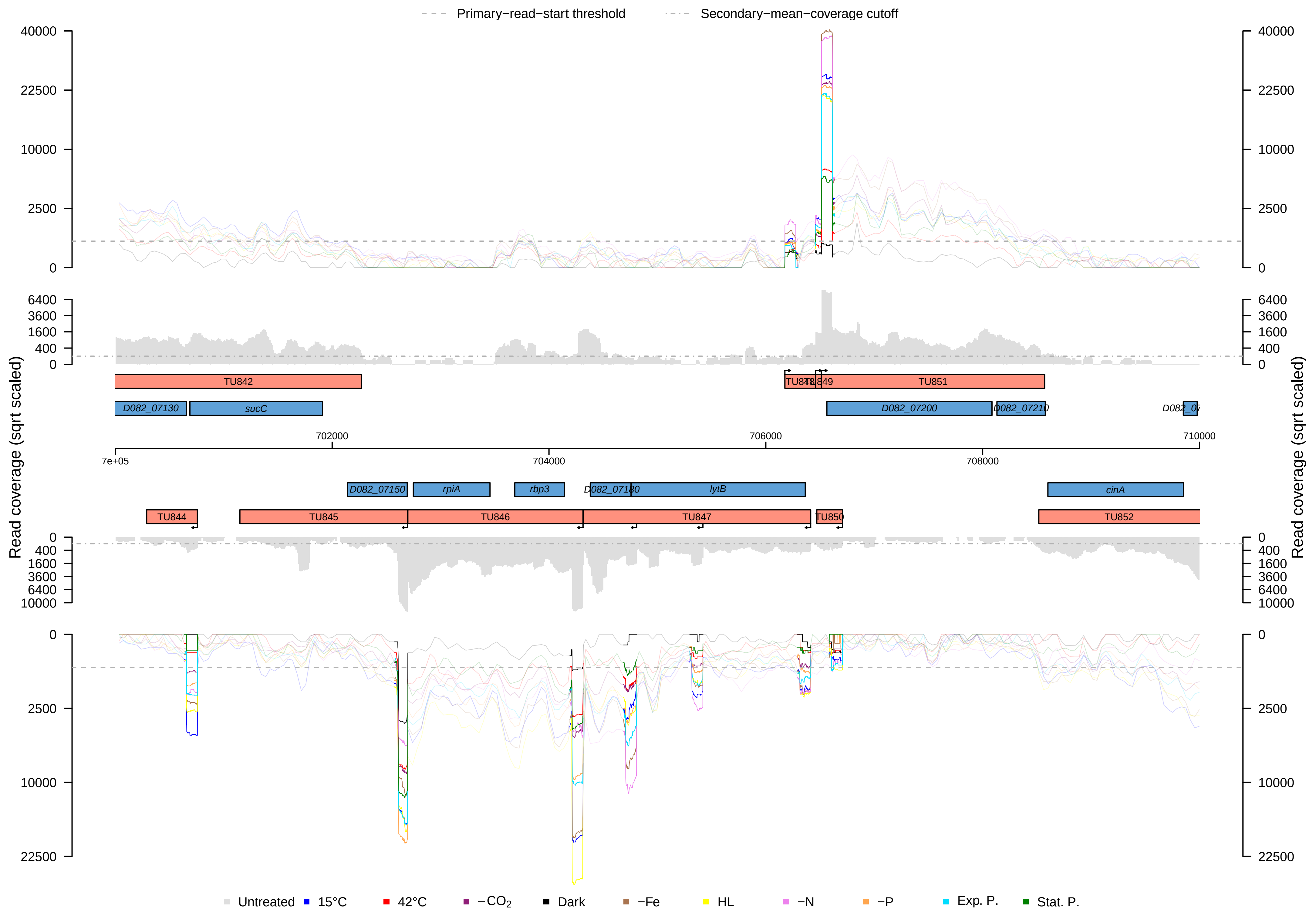

Primary−read−start threshold
Secondary−mean−coverage cutoff
Read coverage (sqrt scaled)
40000
22500
10000
2500
0
6400
3600
1600
400
0
TU842
TU848
TU849
TU851
D082_07130
sucC
D082_07200
D082_07210
D082_07
702000
706000
710000
7e+05
704000
708000
D082_07150
rpiA
rbp3
D082_07180
lytB
cinA
TU844
TU845
TU846
TU847
TU850
TU852
0
400
1600
3600
6400
10000
0
2500
10000
22500
Untreated
15°C
42°C
−CO2
Dark
−Fe
HL
−N
−P
Exp. P.
Stat. P.

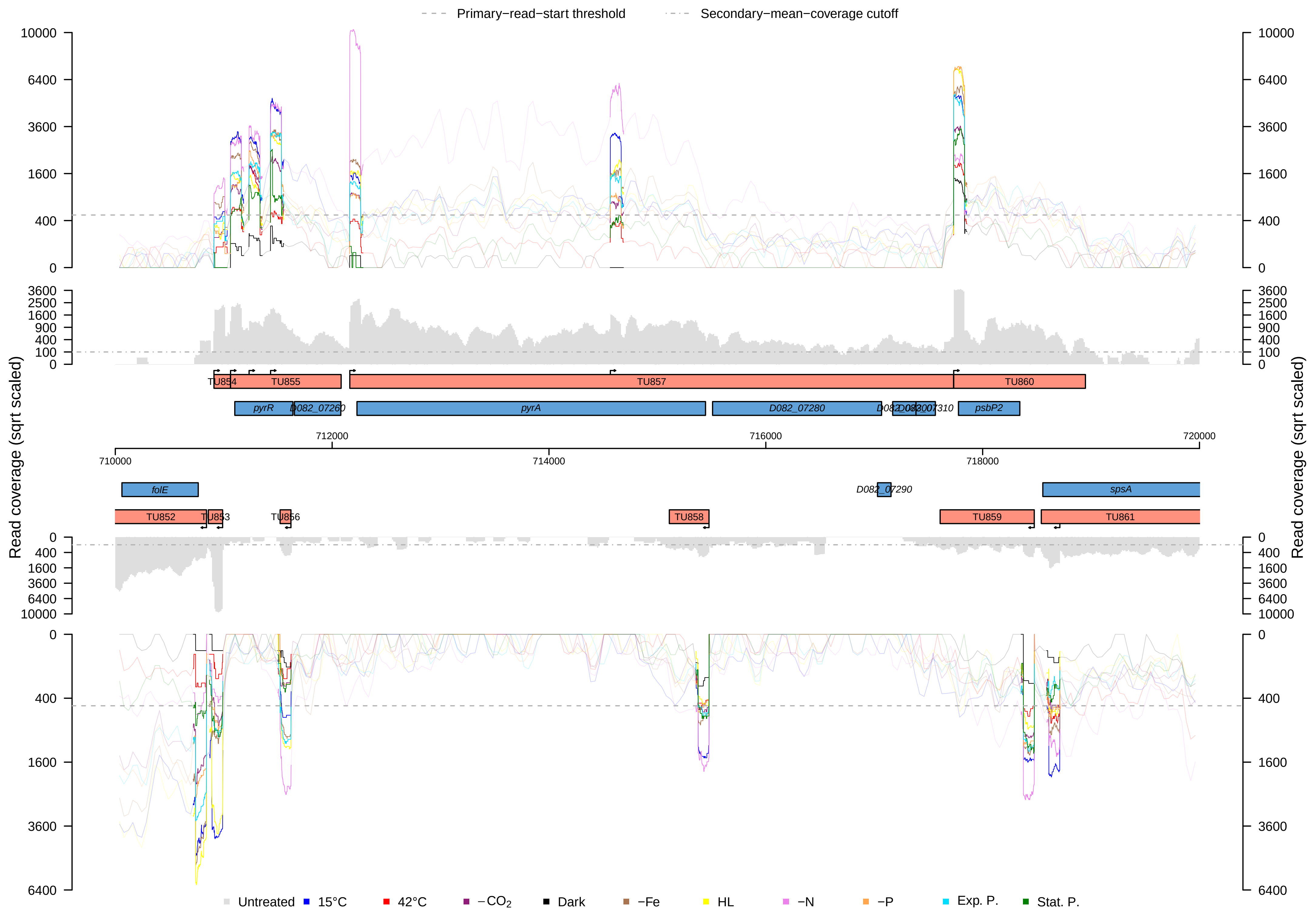
Primary−read−start threshold
Secondary−mean−coverage cutoff
Read coverage (sqrt scaled)
10000
6400
3600
1600
400
0
2500
900
100
TU854
TU855
TU857
TU860
pyrR
D082_07260
pyrA
D082_07280
D082_07310
psbP2
710000
712000
714000
716000
718000
720000
folE
D082_07290
spsA
TU852
TU853
TU856
TU858
TU859
TU861
Untreated
15°C
42°C
−CO_2
Dark
−Fe
HL
−N
−P
Exp. P.
Stat. P.

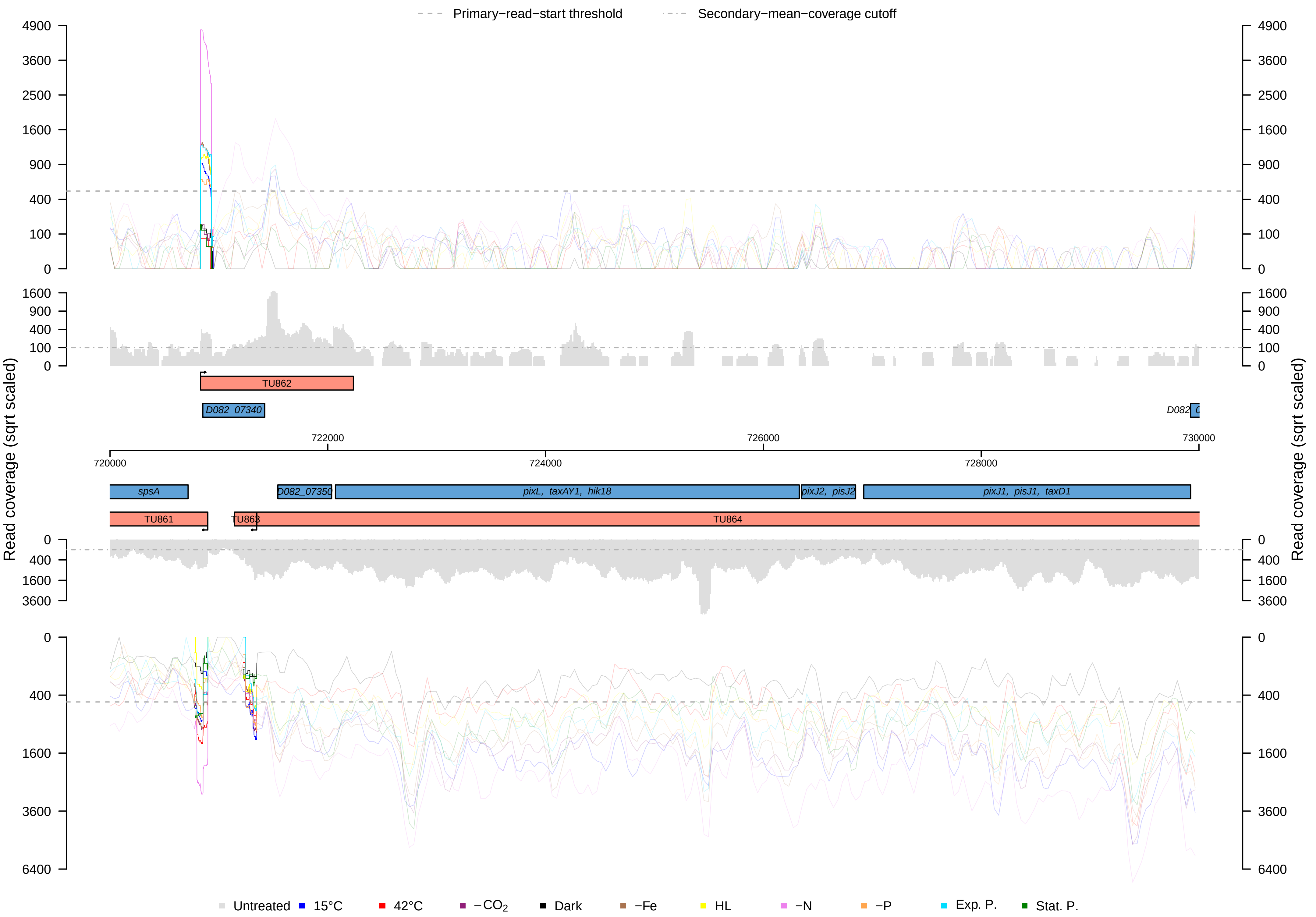

Primary−read−start threshold
Secondary−mean−coverage cutoff
4900
3600
2500
1600
900
400
100
0
TU862
D082_07340
722000
726000
730000
720000
724000
728000
spsA
D082_07350
pixL, taxAY1, hik18
pixJ2, pisJ2
pixJ1, pisJ1, taxD1
TU861
TU863
TU864
6400
Read coverage (sqrt scaled)
Untreated
15°C
42°C
−CO2
Dark
−Fe
HL
−N
−P
Exp. P.
Stat. P.

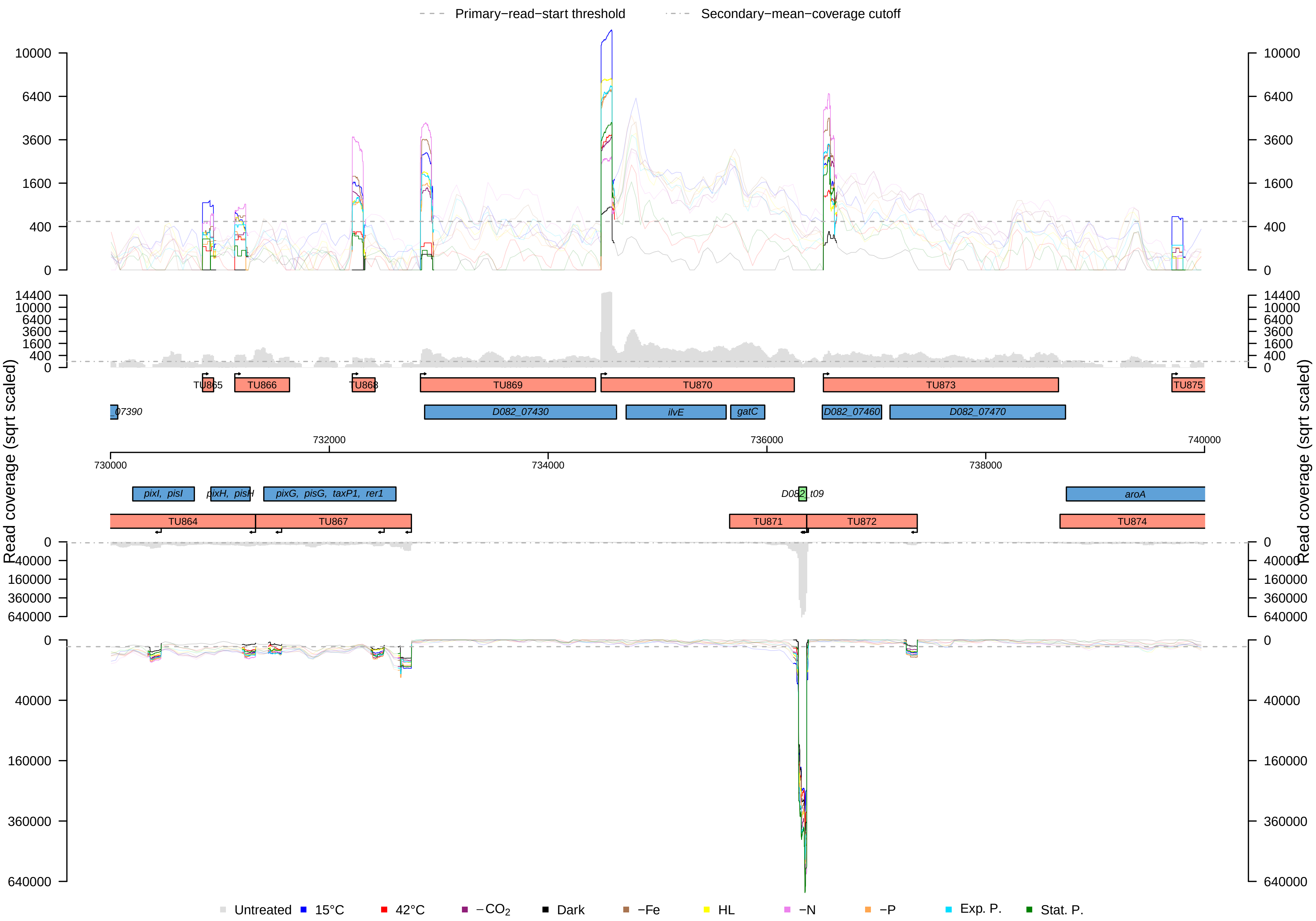

Primary−read−start threshold
Secondary−mean−coverage cutoff
Read coverage (sqrt scaled)
10000
6400
3600
1600
400
0
14400
640000
360000
160000
40000
TU864
TU865
TU866
TU867
TU868
TU869
TU870
TU871
TU872
TU873
TU874
TU875
_07390
D082_07430
ilvE
gatC
D082_07460
D082_07470
pixI, pisI
pixH, pisH
pixG, pisG, taxP1, rer1
D082_t09
aroA
730000
732000
734000
736000
738000
740000
Untreated
15°C
42°C
−CO2
Dark
−Fe
HL
−N
−P
Exp. P.
Stat. P.

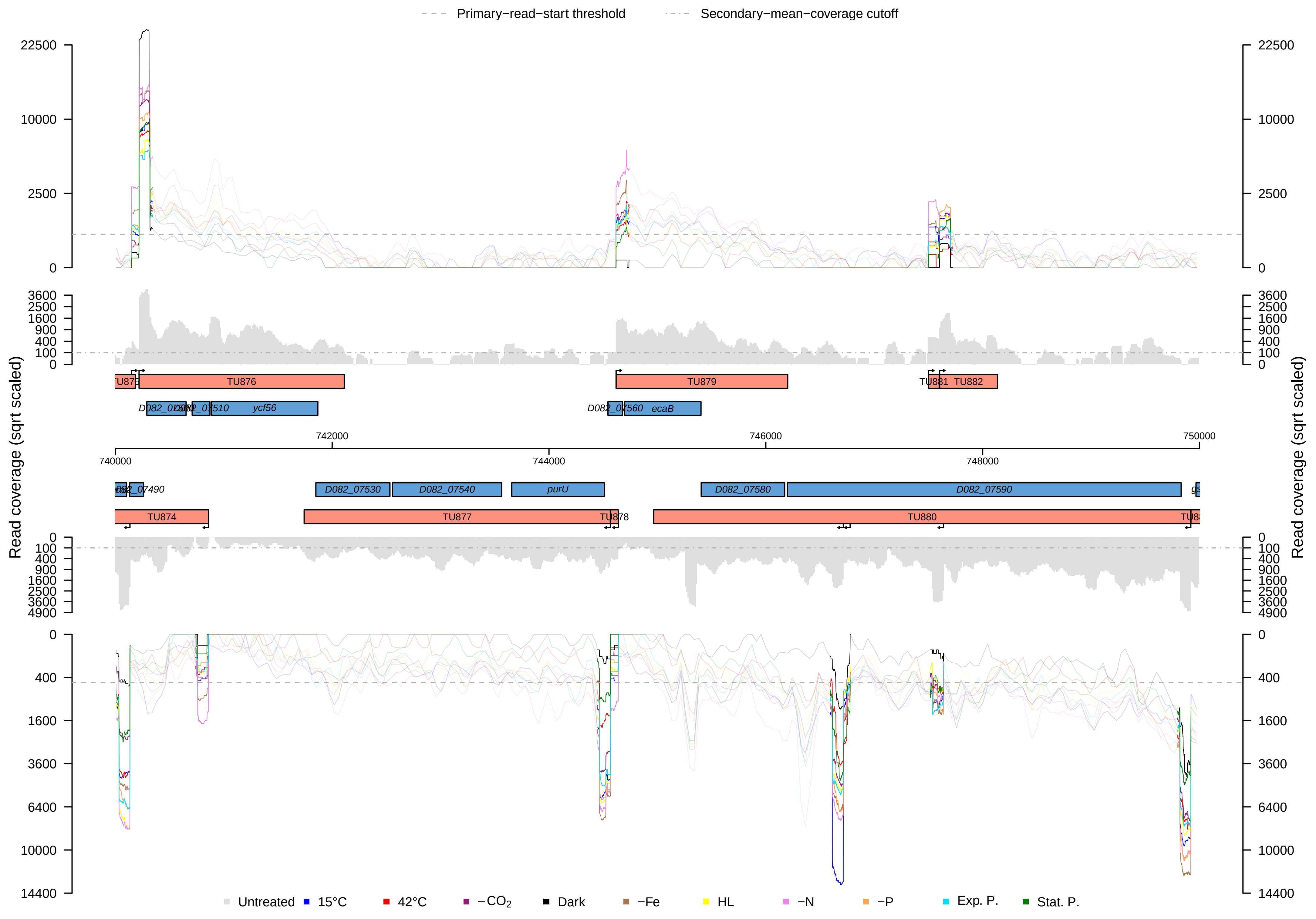

Primary−read−start threshold
Secondary−mean−coverage cutoff
Read coverage (sqrt scaled)
22500
10000
2500
0
3600
2500
1600
900
400
100
0
TU876
TU879
TU882
ycf56
ecaB
742000
746000
750000
740000
744000
748000
D082_07530
D082_07540
purU
D082_07580
D082_07590
TU874
TU877
TU880
4900
14400
6400
3600
1600
400
Untreated
15°C
42°C
−CO2
Dark
−Fe
HL
−N
−P
Exp. P.
Stat. P.

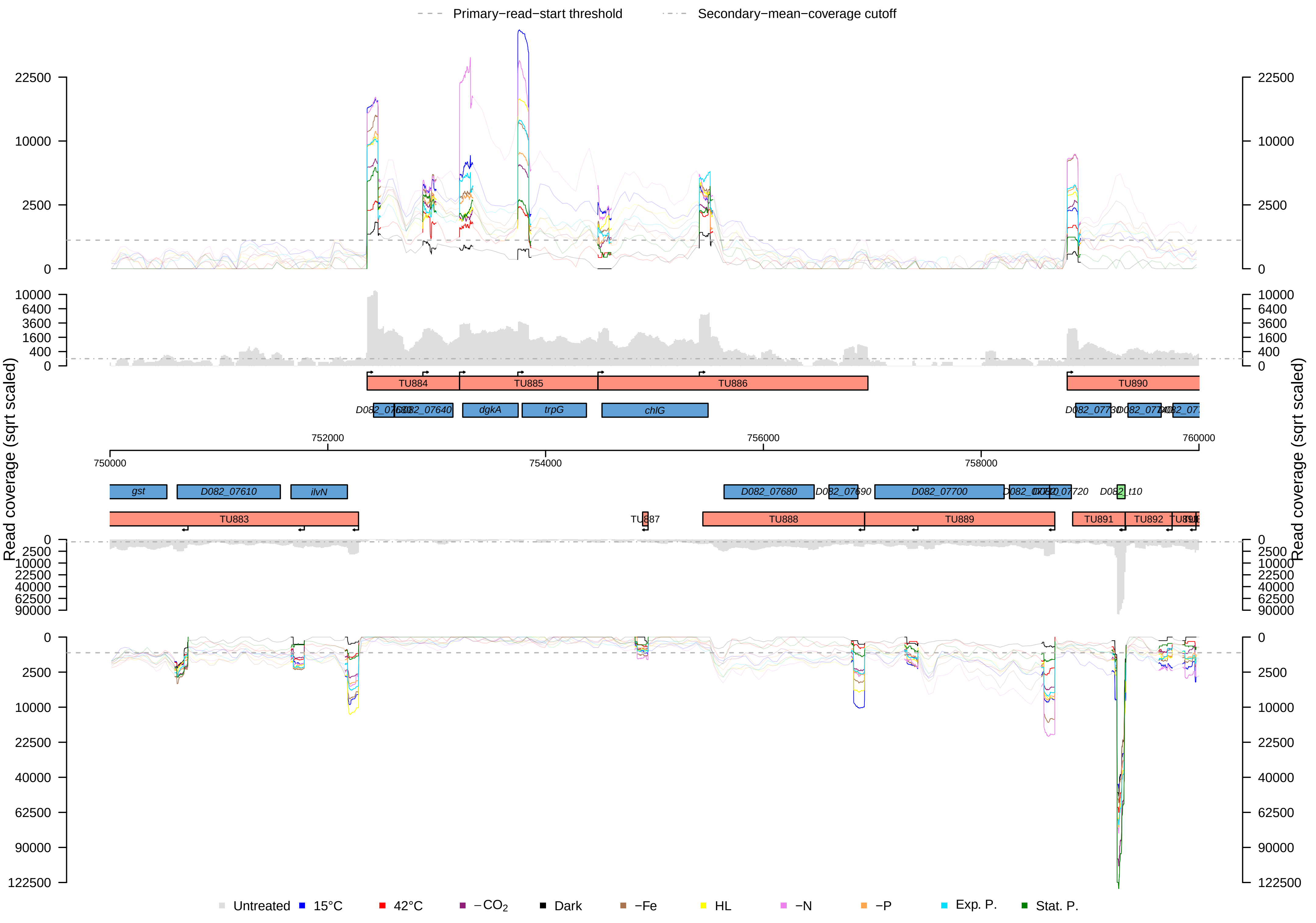

Primary−read−start threshold
Secondary−mean−coverage cutoff
Read coverage (sqrt scaled)
TU883
TU884
TU885
TU886
TU887
TU888
TU889
TU890
TU891
TU892
gst
D082_07610
ilvN
D082_07640
dgkA
trpG
chlG
D082_07680
D082_07700
750000
752000
754000
756000
758000
760000
Untreated
15°C
42°C
−CO2
Dark
−Fe
HL
−N
−P
Exp. P.
Stat. P.

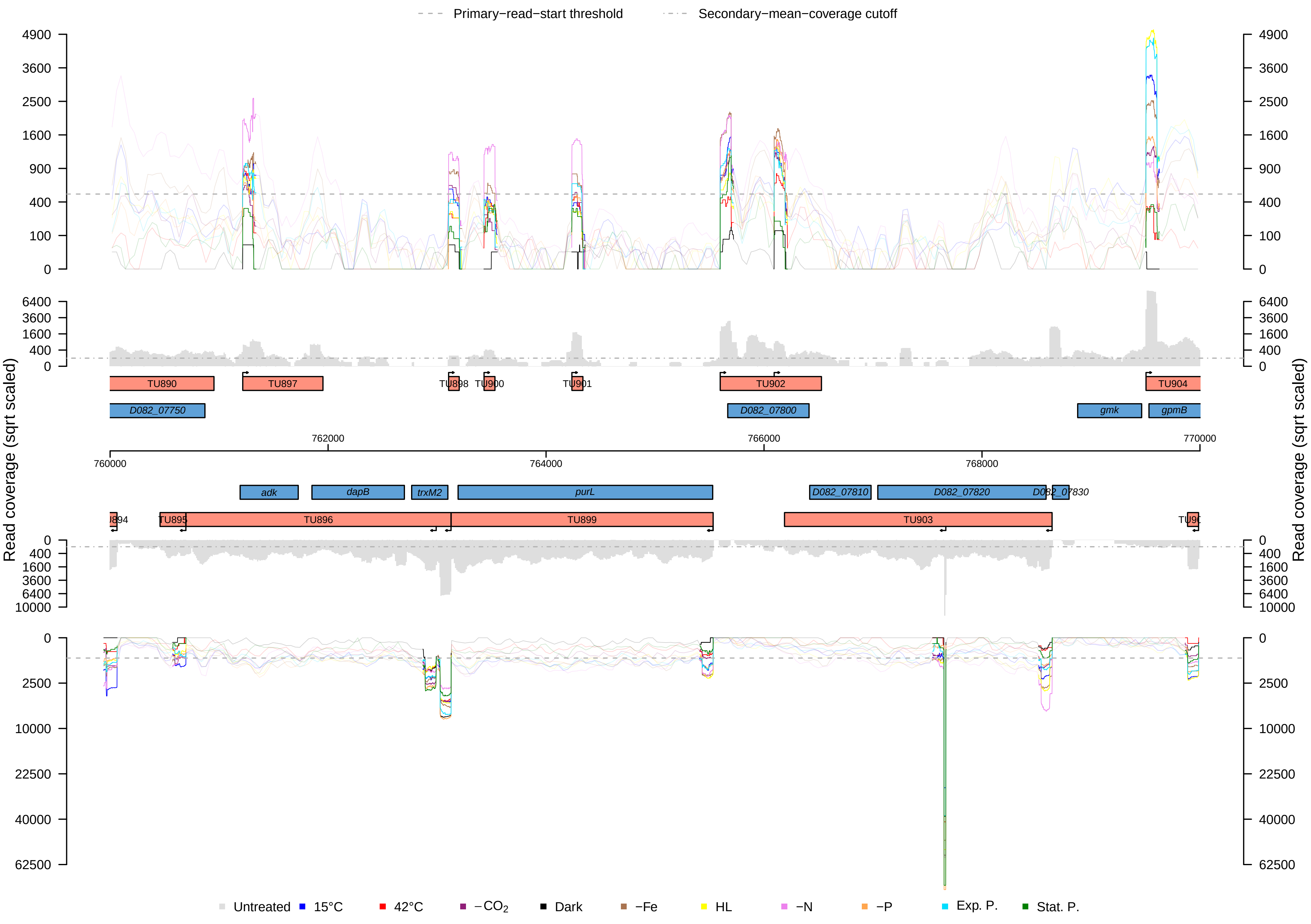

Primary−read−start threshold
Secondary−mean−coverage cutoff
Read coverage (sqrt scaled)
4900
3600
2500
1600
900
400
100
0
6400
3600
1600
400
0
TU890
TU897
TU898
TU900
TU901
TU902
TU904
D082_07750
D082_07800
gmk
gpmB
760000
762000
764000
766000
768000
770000
adk
dapB
trxM2
purL
D082_07810
D082_07820
D082_07830
TU895
TU896
TU899
TU903
10000
2500
22500
40000
62500
Untreated
15°C
42°C
−CO2
Dark
−Fe
HL
−N
−P
Exp. P.
Stat. P.

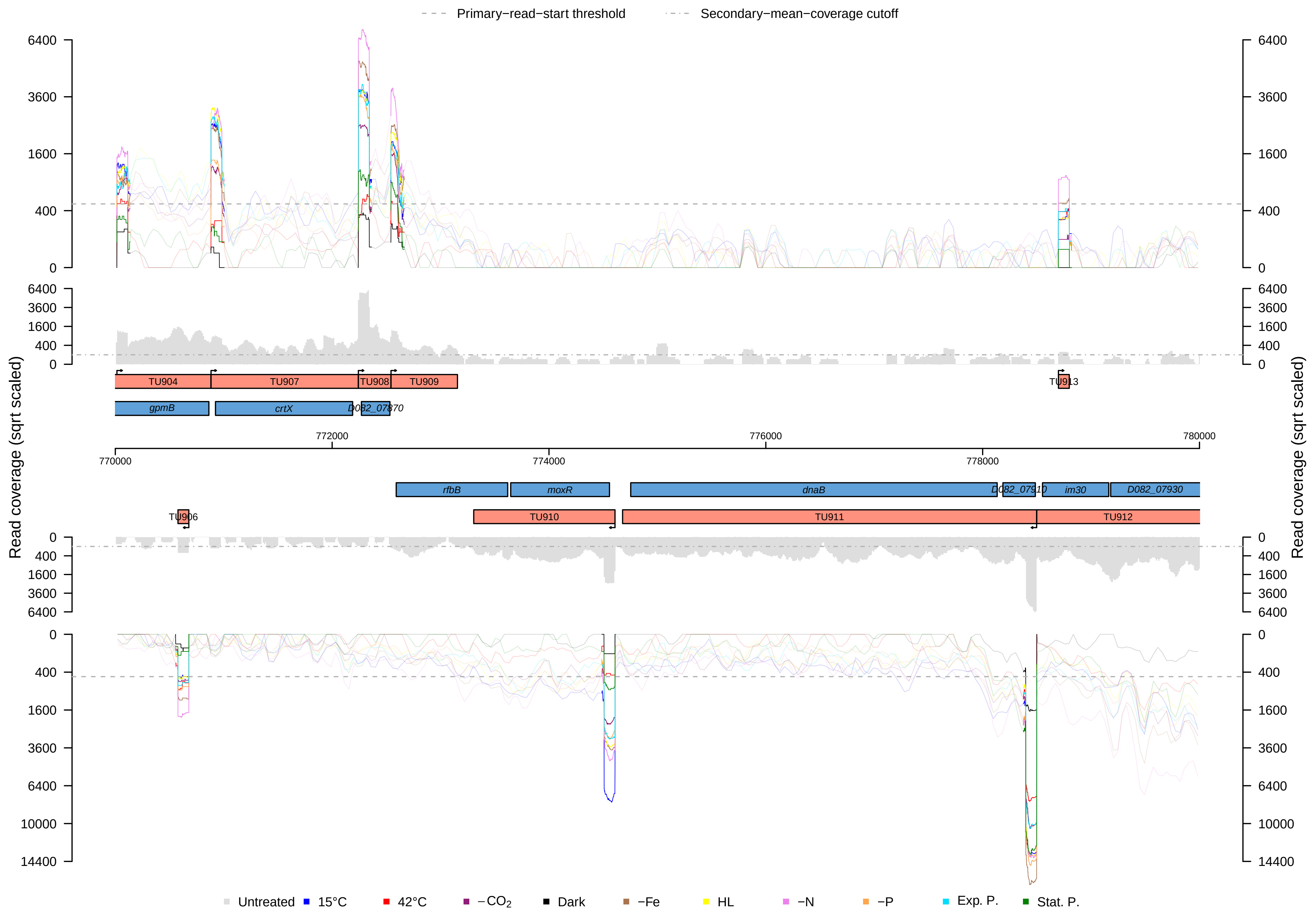

Primary−read−start threshold
Secondary−mean−coverage cutoff
Read coverage (sqrt scaled)
TU904
TU907
TU908
TU909
TU913
gpmB
crtX
D082_07870
770000
772000
774000
776000
778000
780000
rfbB
moxR
dnaB
D082_07910
im30
D082_07930
TU906
TU910
TU911
TU912
Untreated
15°C
42°C
−CO_2
Dark
−Fe
HL
−N
−P
Exp. P.
Stat. P.

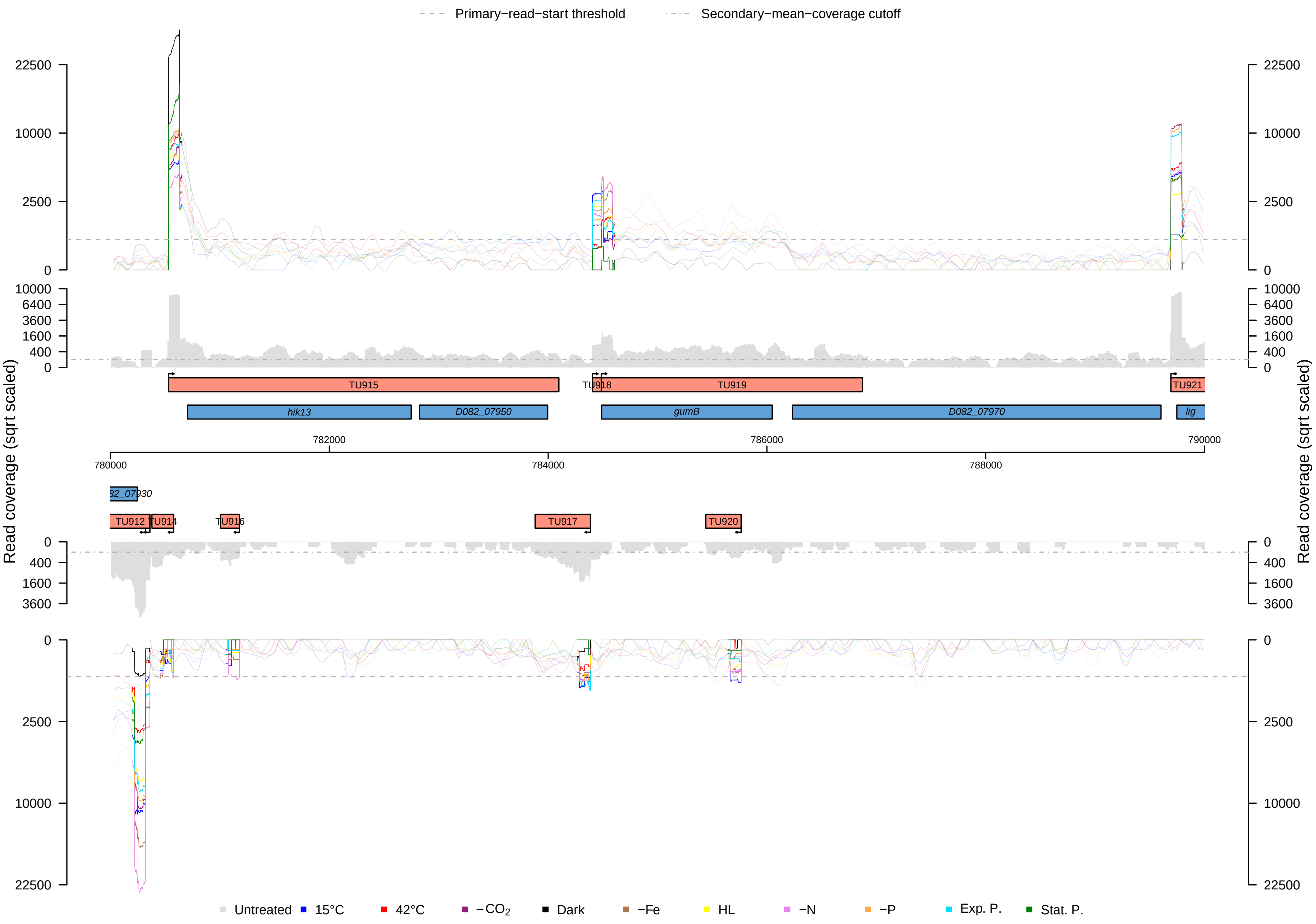

Primary−read−start threshold
Secondary−mean−coverage cutoff
Read coverage (sqrt scaled)
TU915
TU918
TU919
TU921
hik13
D082_07950
gumB
D082_07970
lig
780000
782000
784000
786000
788000
790000
TU912
TU914
TU916
TU917
TU920
Untreated
15°C
42°C
−CO2
Dark
−Fe
HL
−N
−P
Exp. P.
Stat. P.

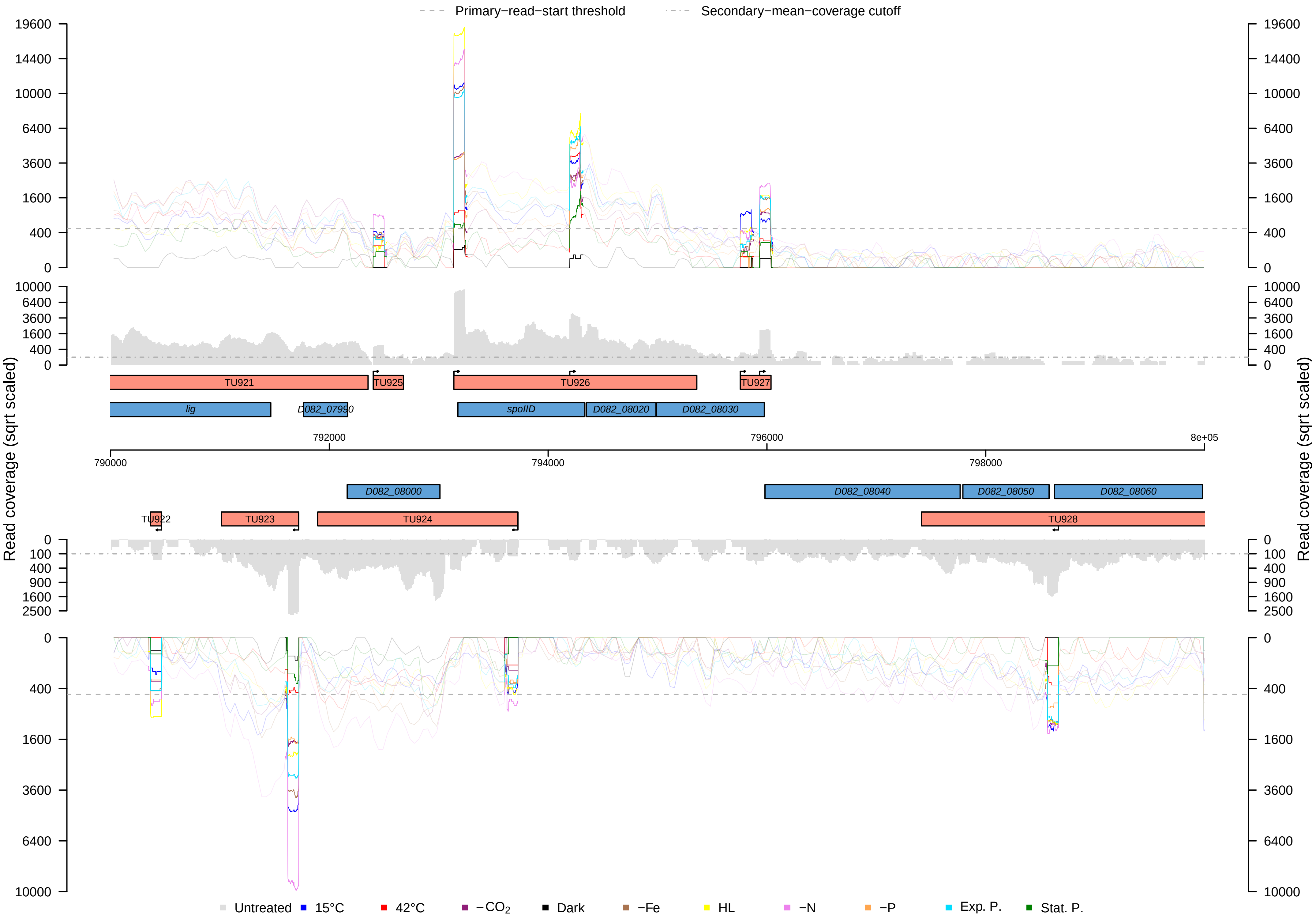

Primary−read−start threshold
Secondary−mean−coverage cutoff
Read coverage (sqrt scaled)
19600
14400
10000
6400
3600
1600
400
0
TU921
TU925
TU926
TU927
lig
D082_07990
spoIID
D082_08020
D082_08030
790000
792000
794000
796000
798000
8e+05
D082_08000
D082_08040
D082_08050
D082_08060
TU922
TU923
TU924
TU928
100
900
2500
Untreated
15°C
42°C
−CO2
Dark
−Fe
HL
−N
−P
Exp. P.
Stat. P.

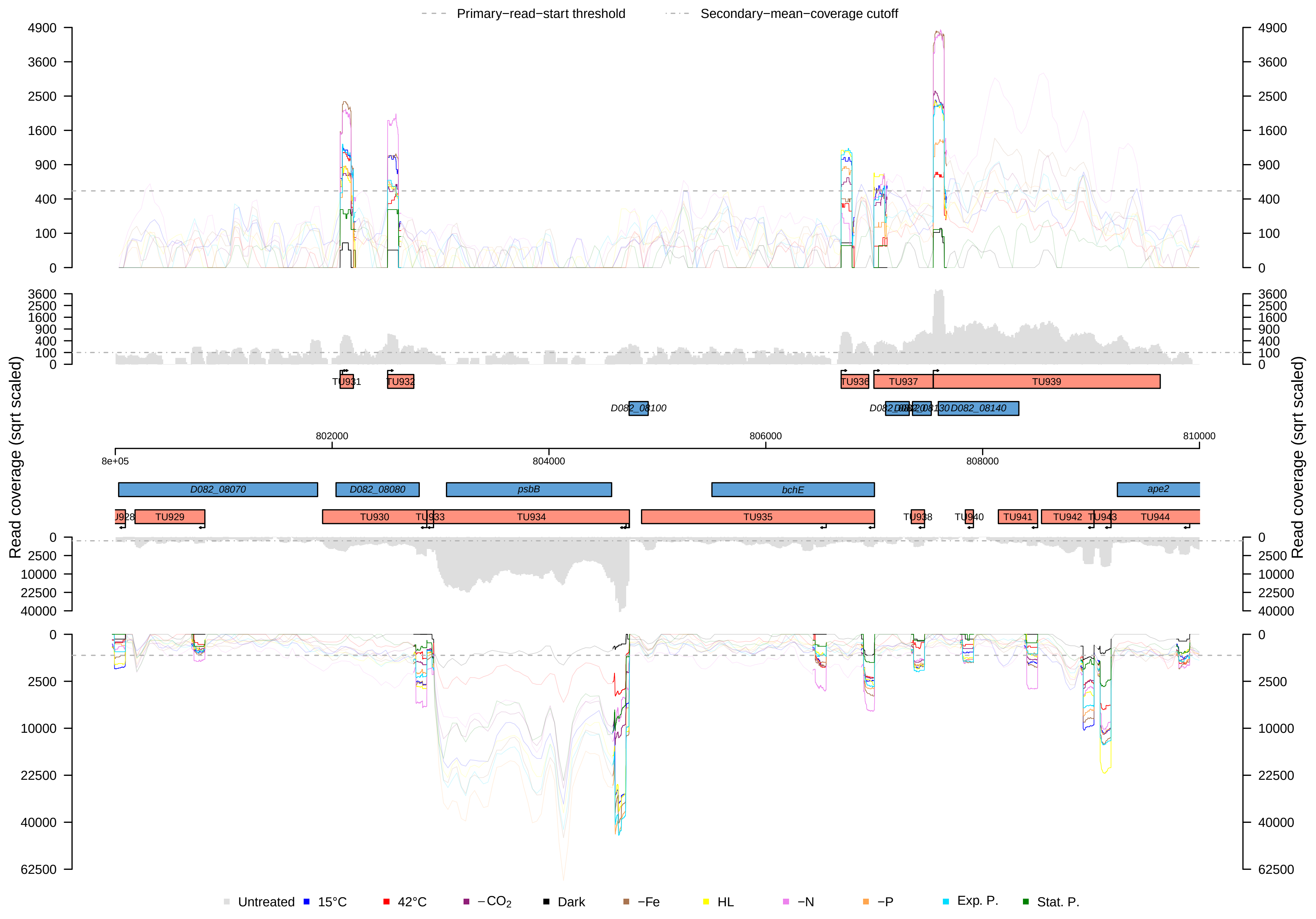

Primary−read−start threshold
Secondary−mean−coverage cutoff
Read coverage (sqrt scaled)
4900
3600
2500
1600
900
400
100
0
TU931
TU932
TU936
TU937
TU939
D082_08100
D082_08140
802000
804000
806000
808000
810000
8e+05
D082_08070
D082_08080
psbB
bchE
ape2
TU929
TU930
TU933
TU934
TU935
TU938
TU940
TU941
TU942
TU943
TU944
10000
22500
40000
62500
Untreated
15°C
42°C
−CO2
Dark
−Fe
HL
−N
−P
Exp. P.
Stat. P.

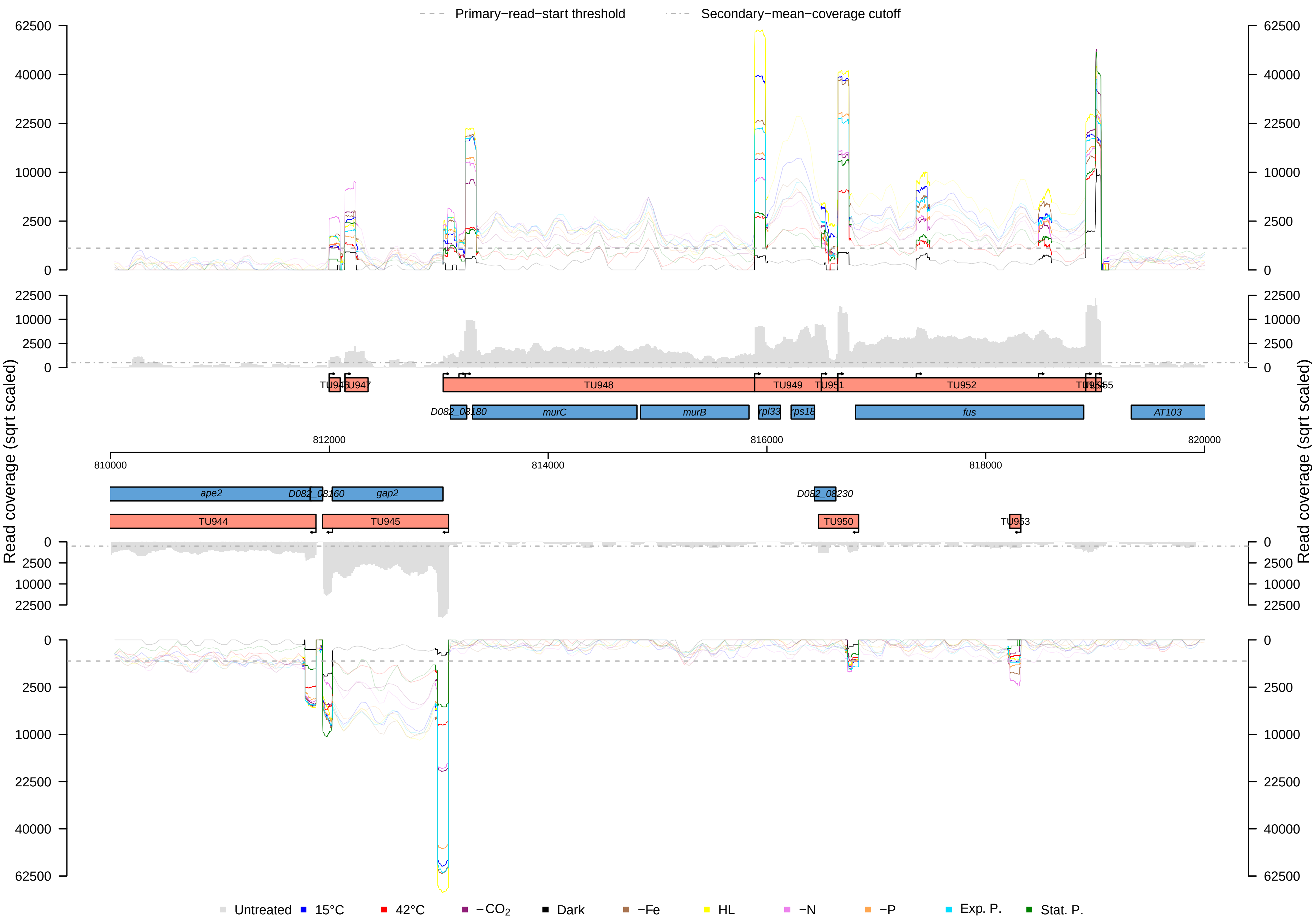

Primary−read−start threshold
Secondary−mean−coverage cutoff
Read coverage (sqrt scaled)
62500
40000
22500
10000
2500
0
TU946
TU947
TU948
TU949
TU951
TU952
TU955
D082_08180
murC
murB
rpl33
rps18
fus
AT103
810000
812000
814000
816000
818000
820000
ape2
D082_08160
gap2
D082_08230
TU944
TU945
TU950
TU953
Untreated
15°C
42°C
−CO2
Dark
−Fe
HL
−N
−P
Exp. P.
Stat. P.

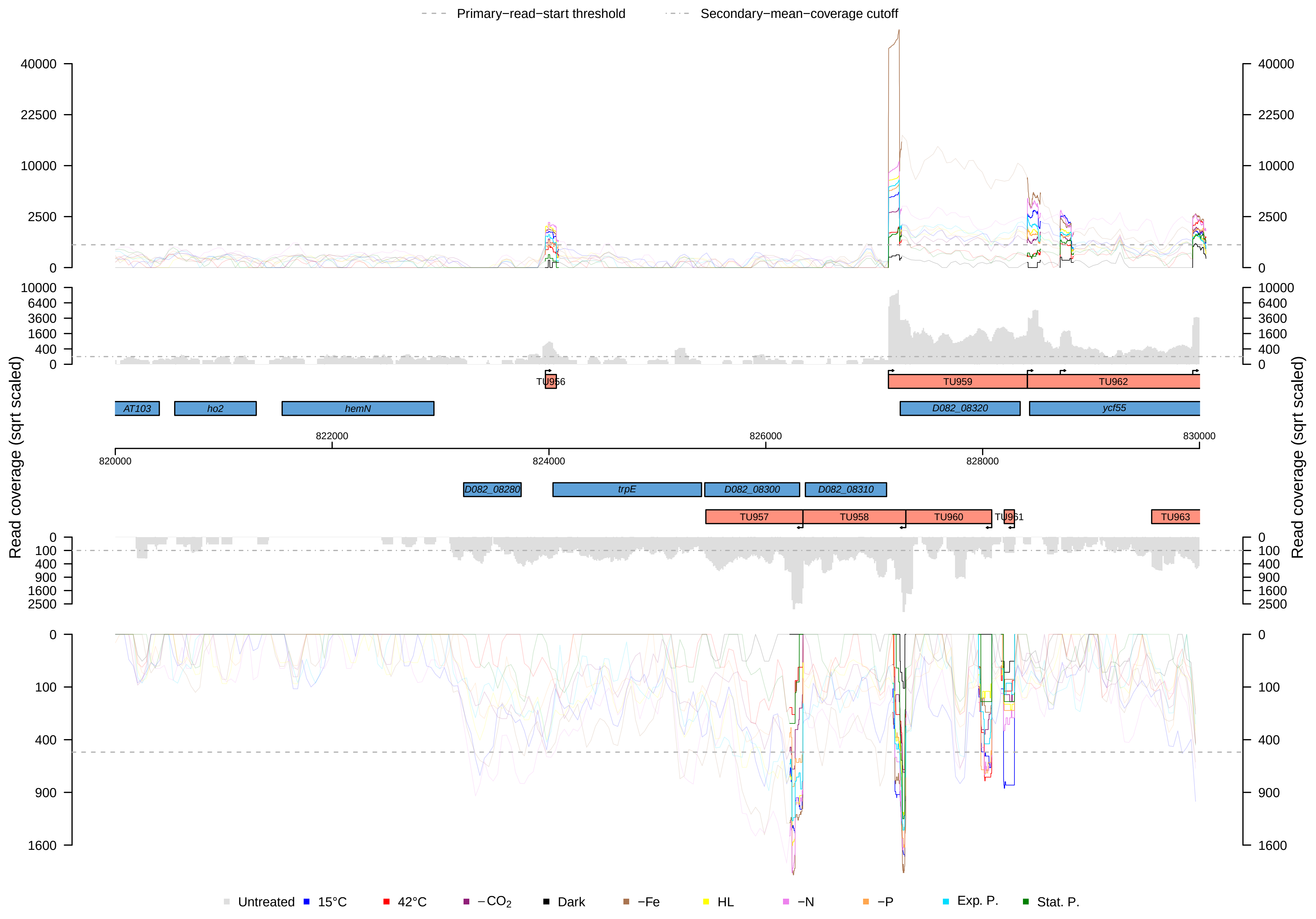

Primary−read−start threshold
Secondary−mean−coverage cutoff
Read coverage (sqrt scaled)
40000
22500
10000
2500
0
10000
6400
3600
1600
400
0
100
400
900
1600
2500
100
400
900
1600
TU956
TU959
TU962
AT103
ho2
hemN
D082_08320
ycf55
822000
826000
830000
820000
824000
828000
D082_08280
trpE
D082_08300
D082_08310
TU957
TU958
TU960
TU961
TU963
Untreated
15°C
42°C
−CO2
Dark
−Fe
HL
−N
−P
Exp. P.
Stat. P.

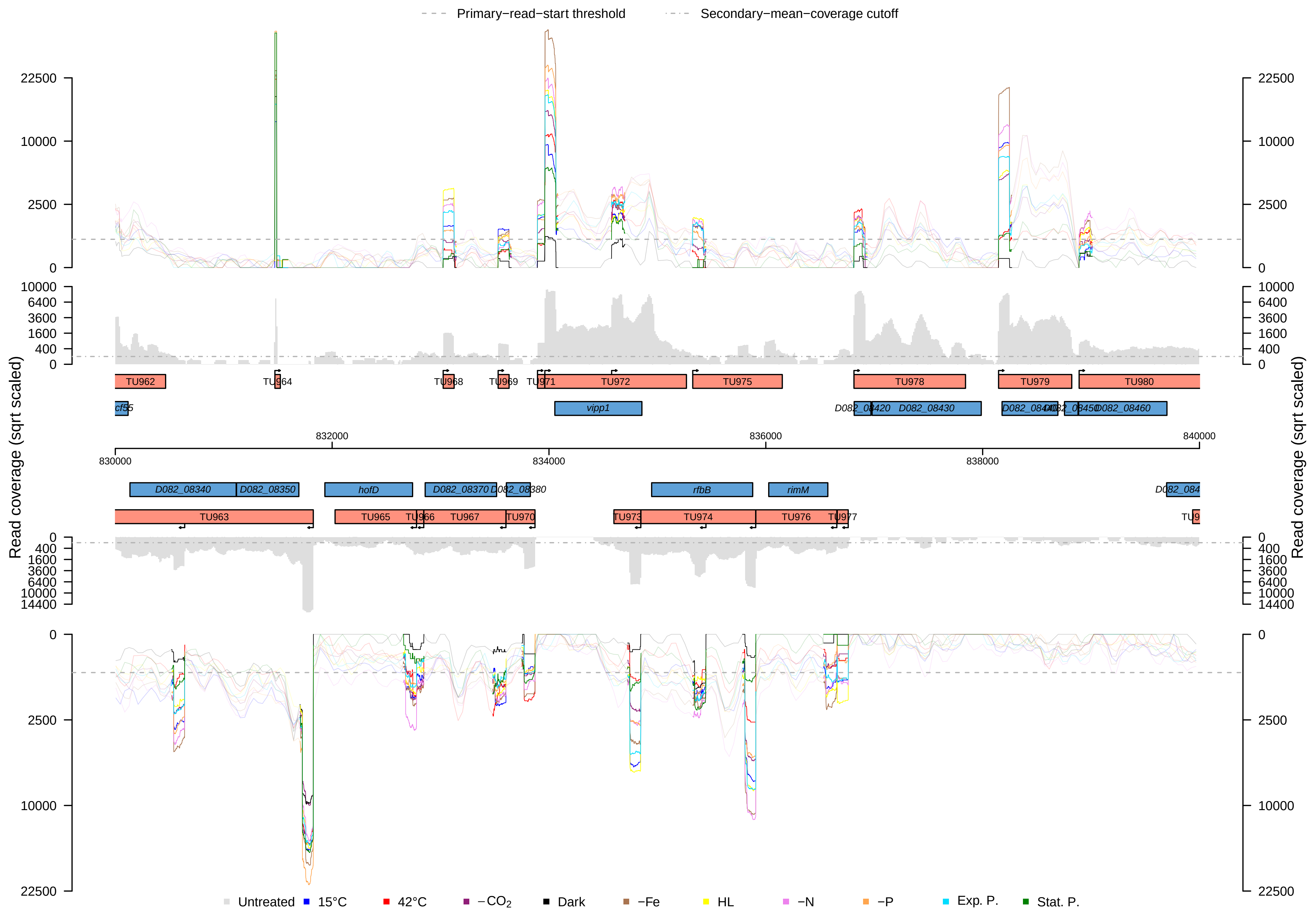

Primary−read−start threshold
Secondary−mean−coverage cutoff
Read coverage (sqrt scaled)
TU962
TU964
TU968
TU969
TU971
TU972
TU975
TU978
TU979
TU980
cf55
vipp1
D082_08420
D082_08430
D082_08460
D082_08340
D082_08350
hofD
D082_08370
D082_08380
rfbB
rimM
TU963
TU965
TU966
TU967
TU970
TU973
TU974
TU976
TU977
830000
832000
834000
836000
838000
840000
Untreated
15°C
42°C
−CO2
Dark
−Fe
HL
−N
−P
Exp. P.
Stat. P.

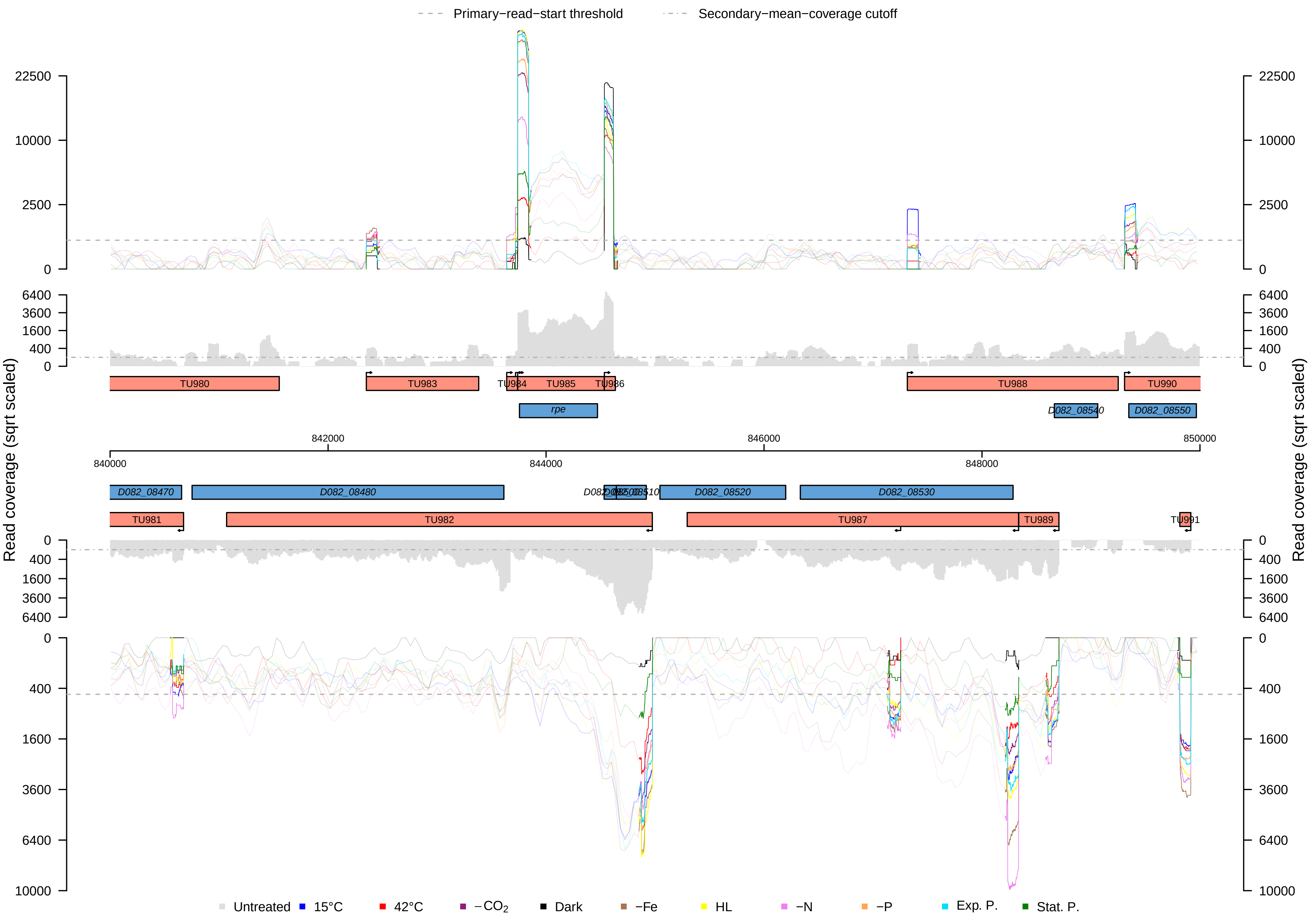

Primary−read−start threshold
Secondary−mean−coverage cutoff
Read coverage (sqrt scaled)
22500
10000
2500
0
6400
3600
1600
400
TU980
TU983
TU984
TU985
TU986
TU988
TU990
rpe
D082_08540
D082_08550
840000
842000
844000
846000
848000
850000
D082_08470
D082_08480
D082_08520
D082_08530
TU981
TU982
TU987
TU989
TU991
Untreated
15°C
42°C
−CO2
Dark
−Fe
HL
−N
−P
Exp. P.
Stat. P.

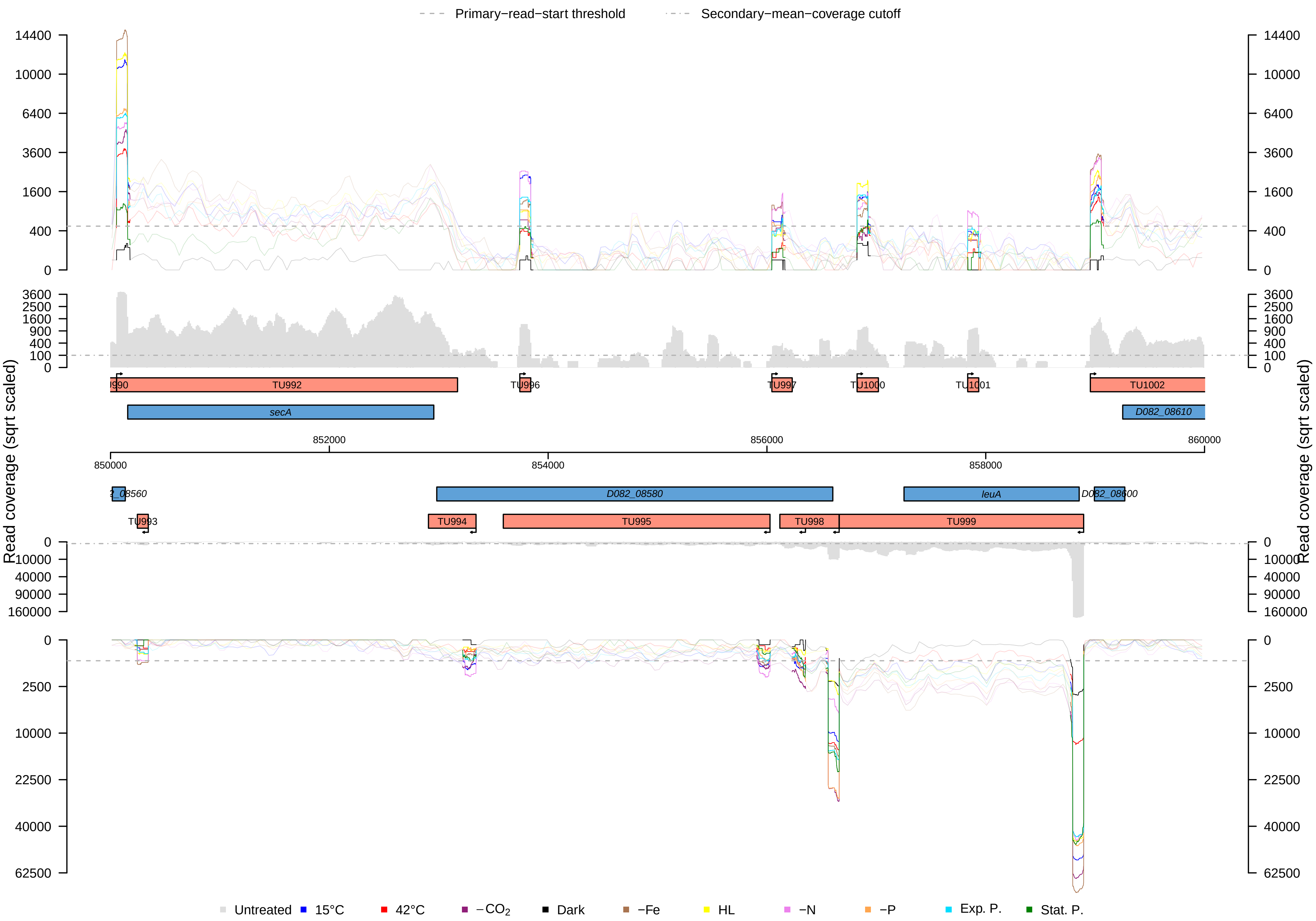

Primary−read−start threshold
Secondary−mean−coverage cutoff
Read coverage (sqrt scaled)
14400
10000
6400
3600
1600
400
0
3600
2500
1600
900
400
100
0
TU992
TU996
TU997
TU1000
TU1001
TU1002
secA
D082_08610
852000
856000
860000
850000
854000
858000
D082_08580
leuA
TU993
TU994
TU995
TU998
TU999
10000
40000
90000
160000
2500
22500
62500
Untreated
15°C
42°C
−CO2
Dark
−Fe
HL
−N
−P
Exp. P.
Stat. P.

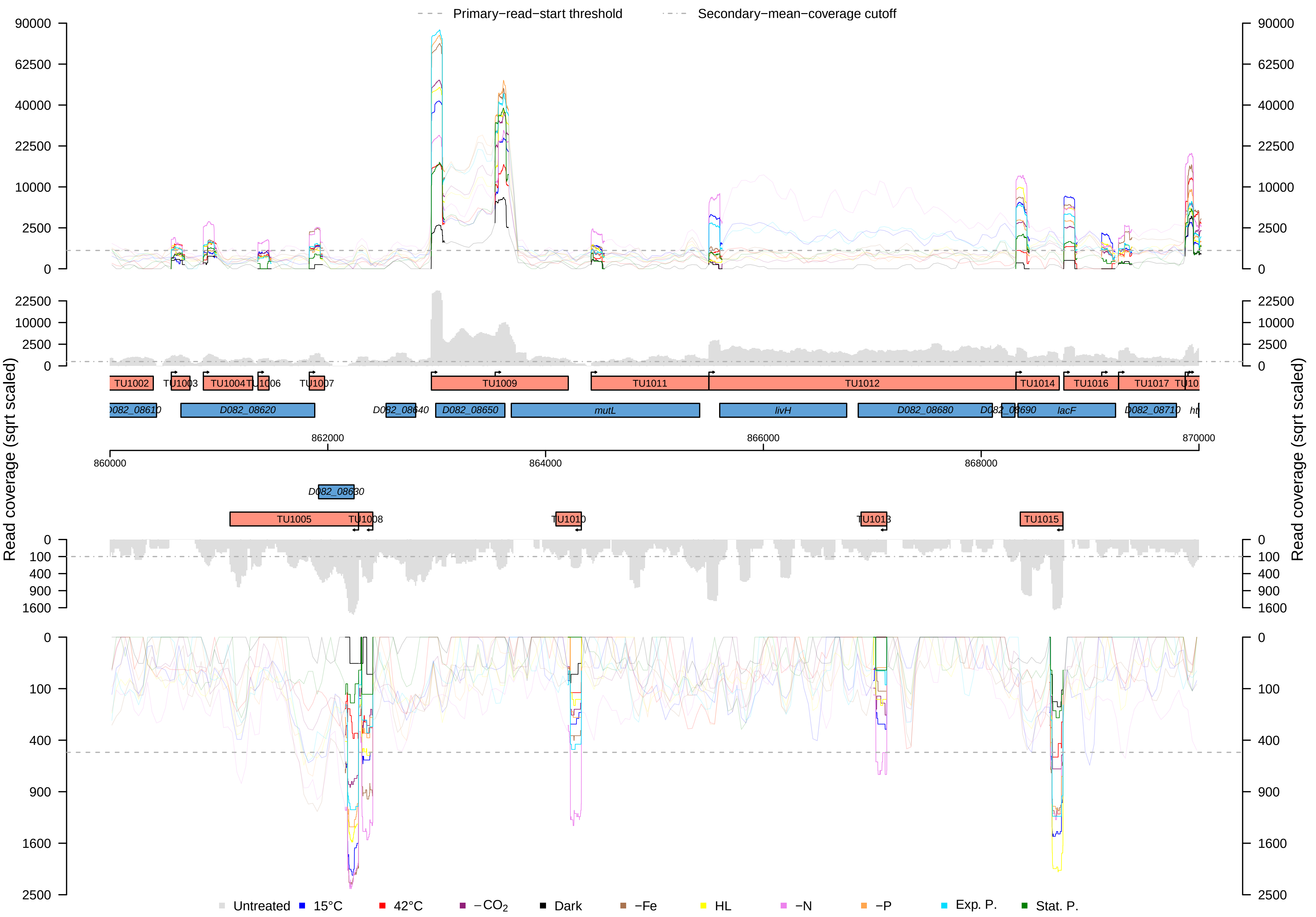

Primary−read−start threshold
Secondary−mean−coverage cutoff
Read coverage (sqrt scaled)
TU1002
TU1003
TU1004
TU1006
TU1007
TU1009
TU1011
TU1012
TU1014
TU1016
TU1017
D082_08610
D082_08620
D082_08640
D082_08650
mutL
livH
D082_08680
D082_08690
lacF
D082_08710
860000
862000
864000
866000
868000
870000
D082_08630
TU1005
TU1008
TU1010
TU1013
TU1015
Untreated
15°C
42°C
−CO2
Dark
−Fe
HL
−N
−P
Exp. P.
Stat. P.

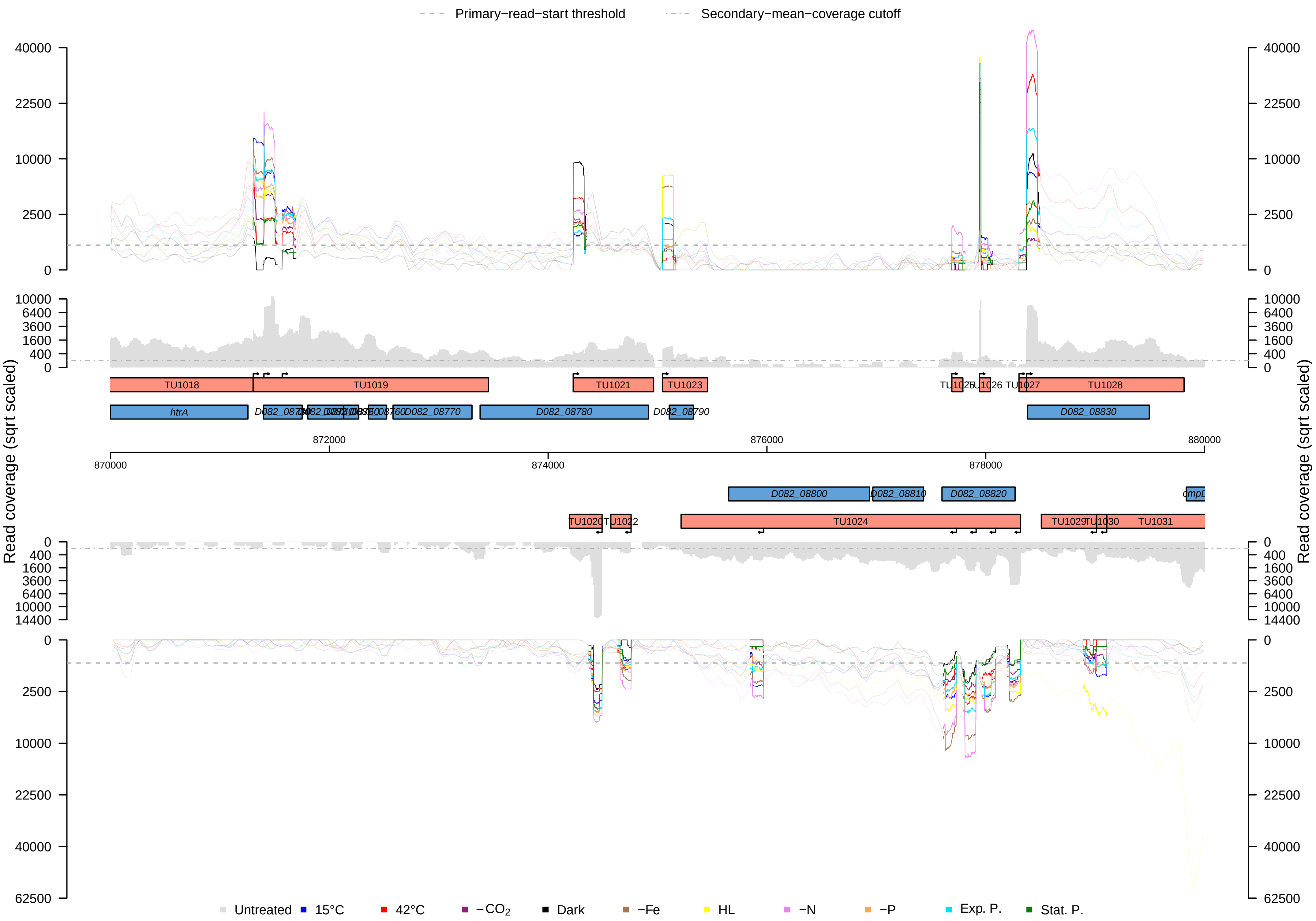

Primary−read−start threshold
Secondary−mean−coverage cutoff
Read coverage (sqrt scaled)
TU1018
TU1019
TU1021
TU1023
TU1028
htrA
D082_08780
D082_08770
D082_08830
870000
872000
874000
876000
878000
880000
D082_08800
D082_08820
TU1024
TU1031
Untreated
15°C
42°C
−CO2
Dark
−Fe
HL
−N
−P
Exp. P.
Stat. P.

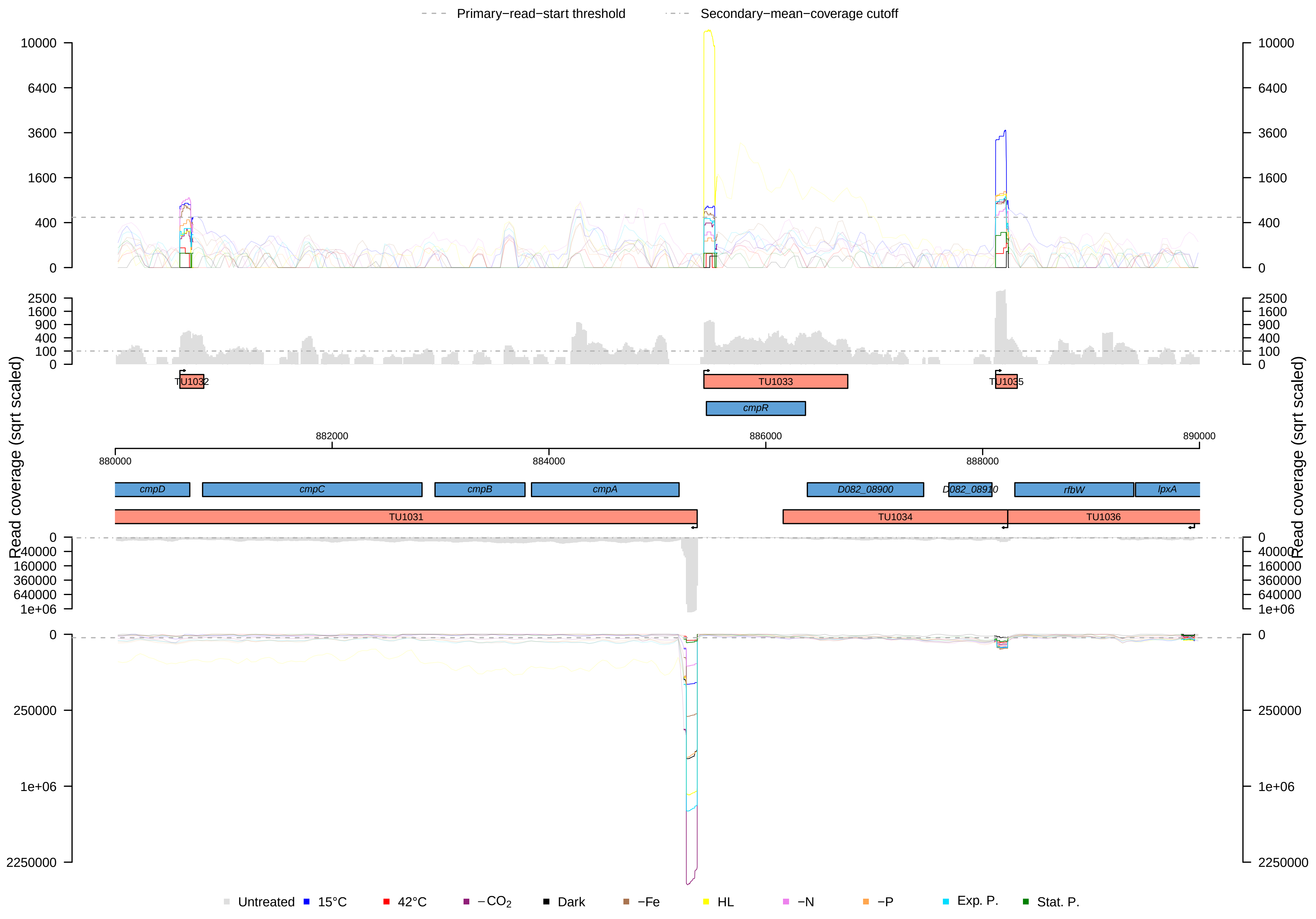

Primary−read−start threshold
Secondary−mean−coverage cutoff
Read coverage (sqrt scaled)
TU1031
TU1032
TU1033
TU1034
TU1035
TU1036
cmpR
cmpD
cmpC
cmpB
cmpA
D082_08900
D082_08910
rfbW
lpxA
880000
882000
884000
886000
888000
890000
Untreated
15°C
42°C
−CO2
Dark
−Fe
HL
−N
−P
Exp. P.
Stat. P.

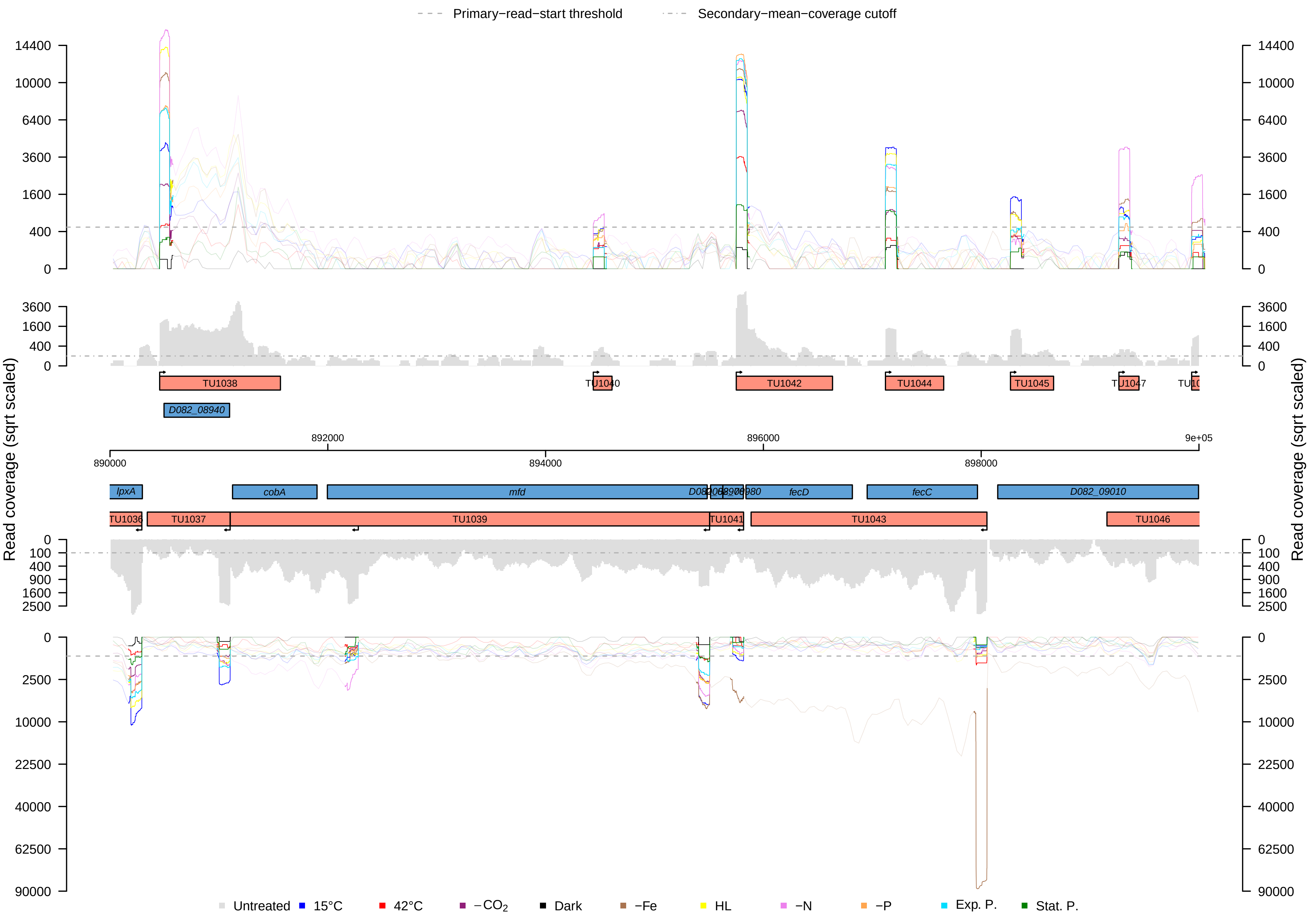

Primary−read−start threshold
Secondary−mean−coverage cutoff
Read coverage (sqrt scaled)
TU1038
D082_08940
TU1040
TU1042
TU1044
TU1045
TU1047
890000
892000
894000
896000
898000
9e+05
lpxA
cobA
mfd
fecD
fecC
D082_09010
TU1036
TU1037
TU1039
TU1041
TU1043
TU1046
Untreated
15°C
42°C
−CO2
Dark
−Fe
HL
−N
−P
Exp. P.
Stat. P.

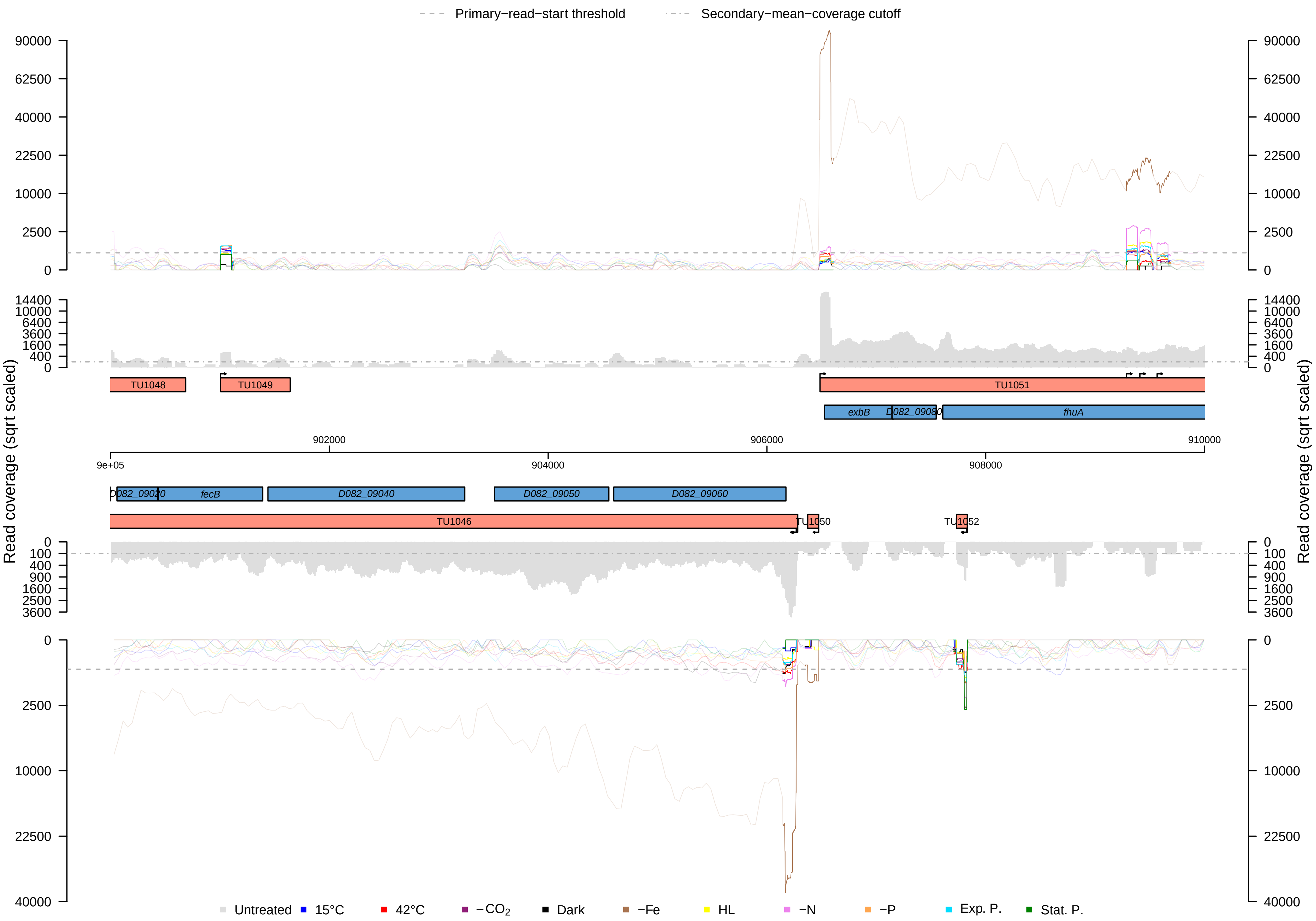

Primary−read−start threshold
Secondary−mean−coverage cutoff
Read coverage (sqrt scaled)
90000
62500
40000
22500
10000
2500
0
14400
10000
6400
3600
1600
400
100
TU1048
TU1049
TU1051
exbB
D082_09080
fhuA
902000
906000
910000
9e+05
904000
908000
D082_09020
fecB
D082_09040
D082_09050
D082_09060
TU1046
TU1050
TU1052
Untreated
15°C
42°C
−CO2
Dark
−Fe
HL
−N
−P
Exp. P.
Stat. P.

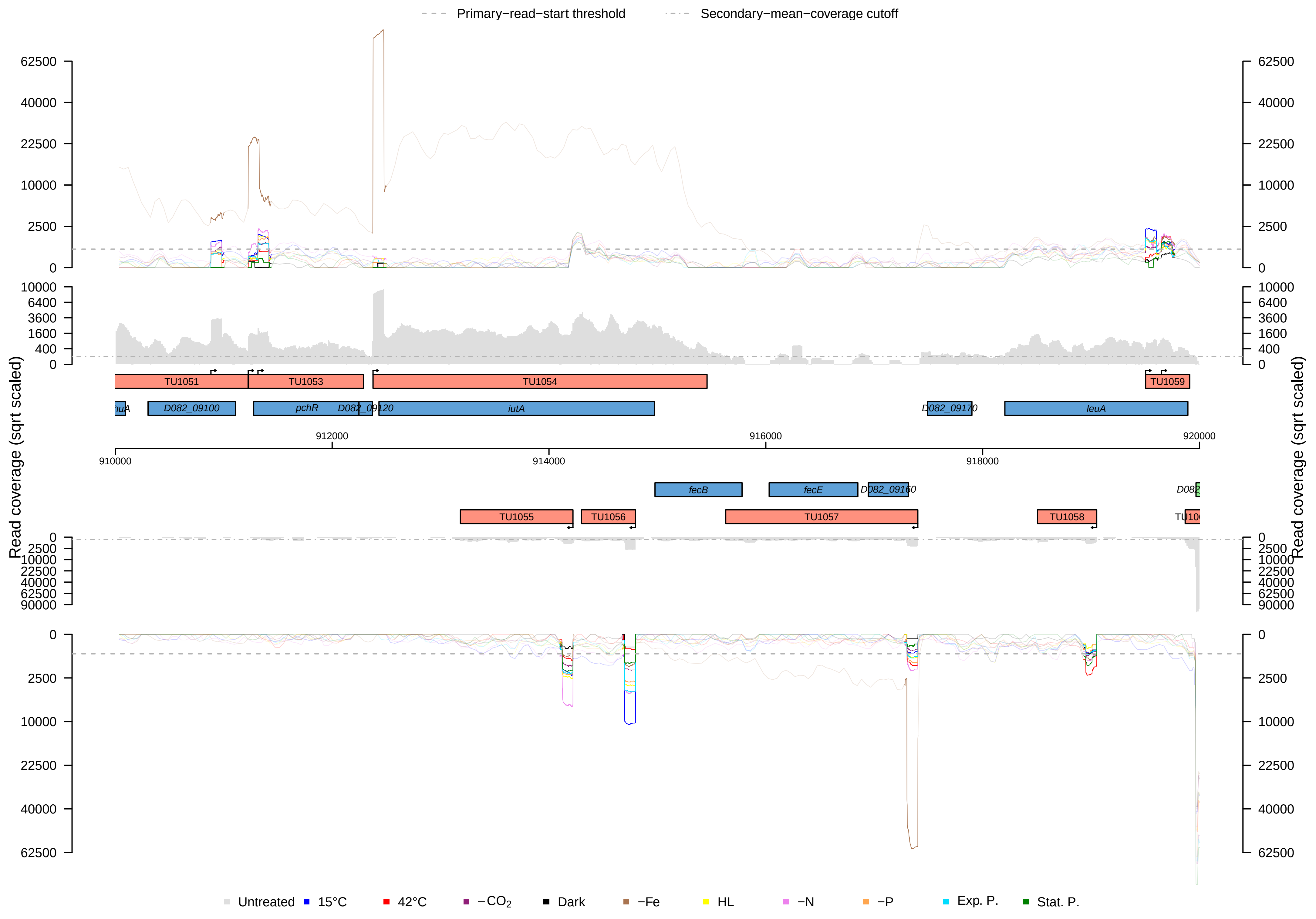

Primary−read−start threshold
Secondary−mean−coverage cutoff
Read coverage (sqrt scaled)
62500
40000
22500
10000
2500
0
10000
6400
3600
1600
400
TU1051
TU1053
TU1054
TU1059
D082_09100
pchR
D082_09120
iutA
D082_09170
leuA
910000
912000
914000
916000
918000
920000
fecB
fecE
D082_09160
TU1055
TU1056
TU1057
TU1058
90000
Untreated
15°C
42°C
−CO2
Dark
−Fe
HL
−N
−P
Exp. P.
Stat. P.

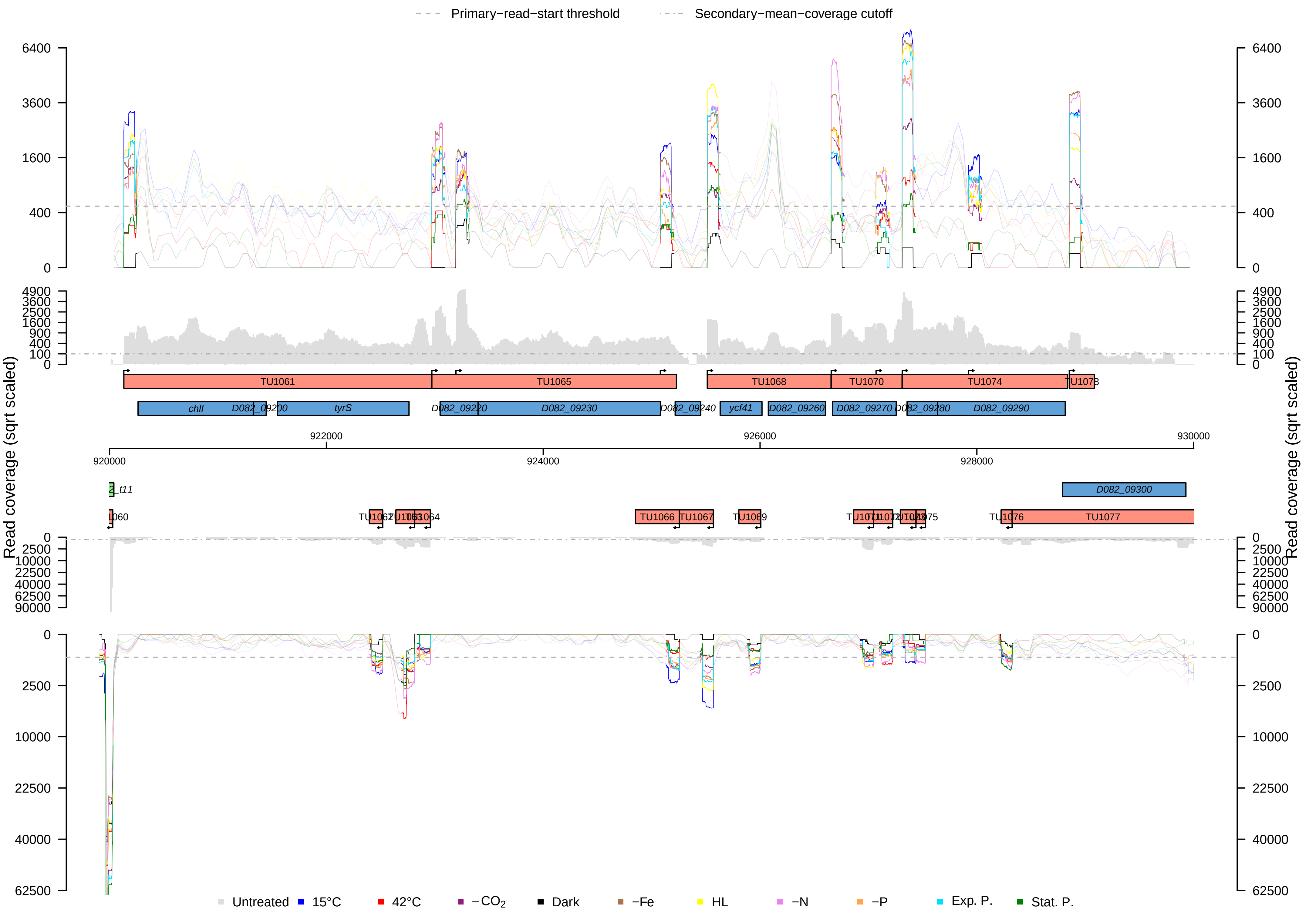

Primary−read−start threshold
Secondary−mean−coverage cutoff
Read coverage (sqrt scaled)
Read coverage (sqrt scaled)
6400
3600
1600
400
0
4900
3600
2500
1600
900
400
100
0
TU1061
TU1065
TU1068
TU1070
TU1074
TU1078
chlI
D082_09200
tyrS
D082_09220
D082_09230
D082_09240
ycf41
D082_09260
D082_09270
D082_09280
D082_09290
922000
926000
930000
920000
924000
928000
D082_09300
TU1066
TU1067
TU1069
TU1076
TU1077
2500
10000
22500
40000
62500
90000
Untreated
15°C
42°C
−CO2
Dark
−Fe
HL
−N
−P
Exp. P.
Stat. P.

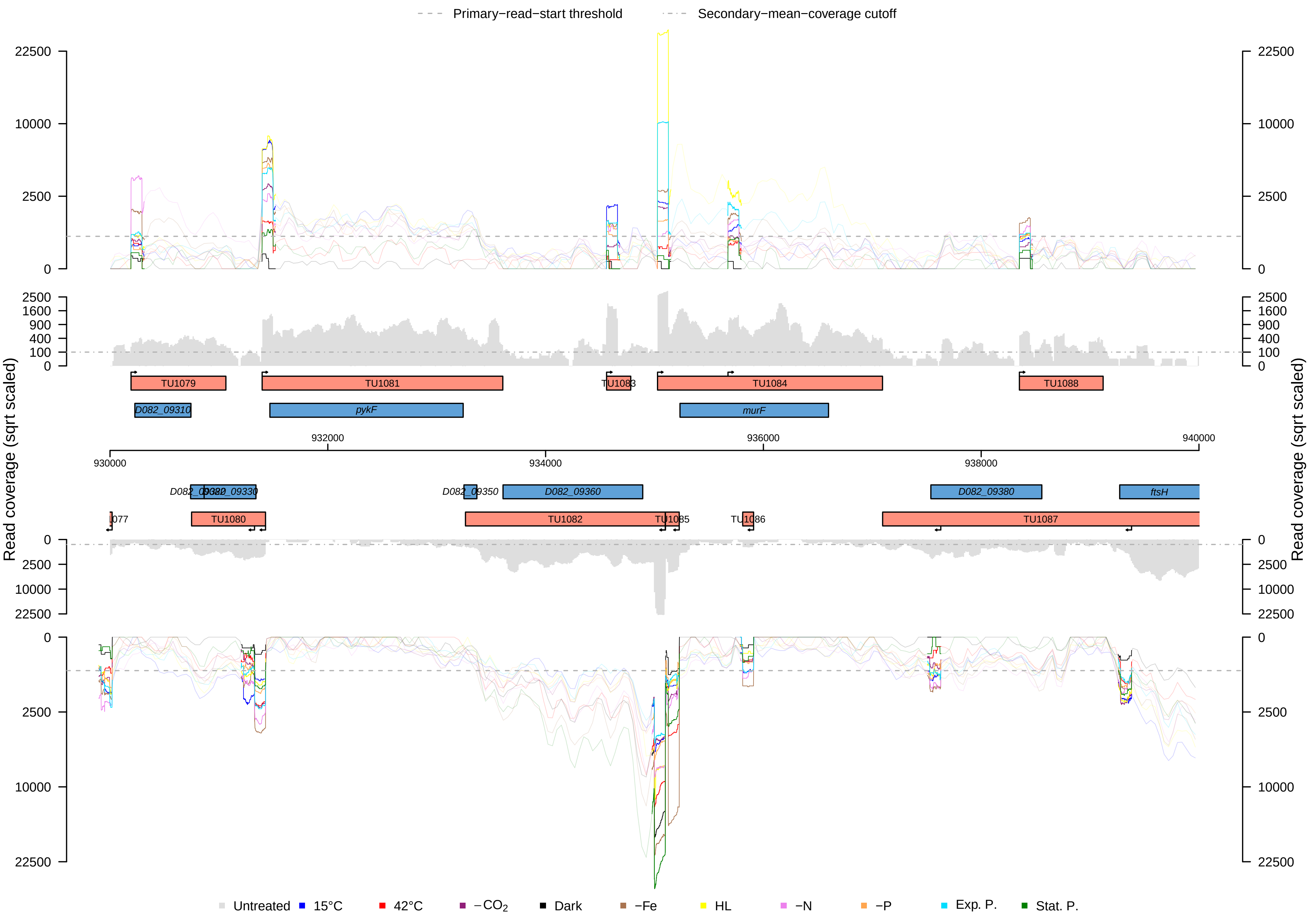

Primary−read−start threshold
Secondary−mean−coverage cutoff
Read coverage (sqrt scaled)
22500
10000
2500
0
1600
900
400
100
TU1079
TU1081
TU1083
TU1084
TU1088
D082_09310
pykF
murF
930000
932000
934000
936000
938000
940000
D082_09320
D082_09330
D082_09350
D082_09360
D082_09380
ftsH
077
TU1080
TU1082
TU1085
TU1086
TU1087
Untreated
15°C
42°C
−CO2
Dark
−Fe
HL
−N
−P
Exp. P.
Stat. P.

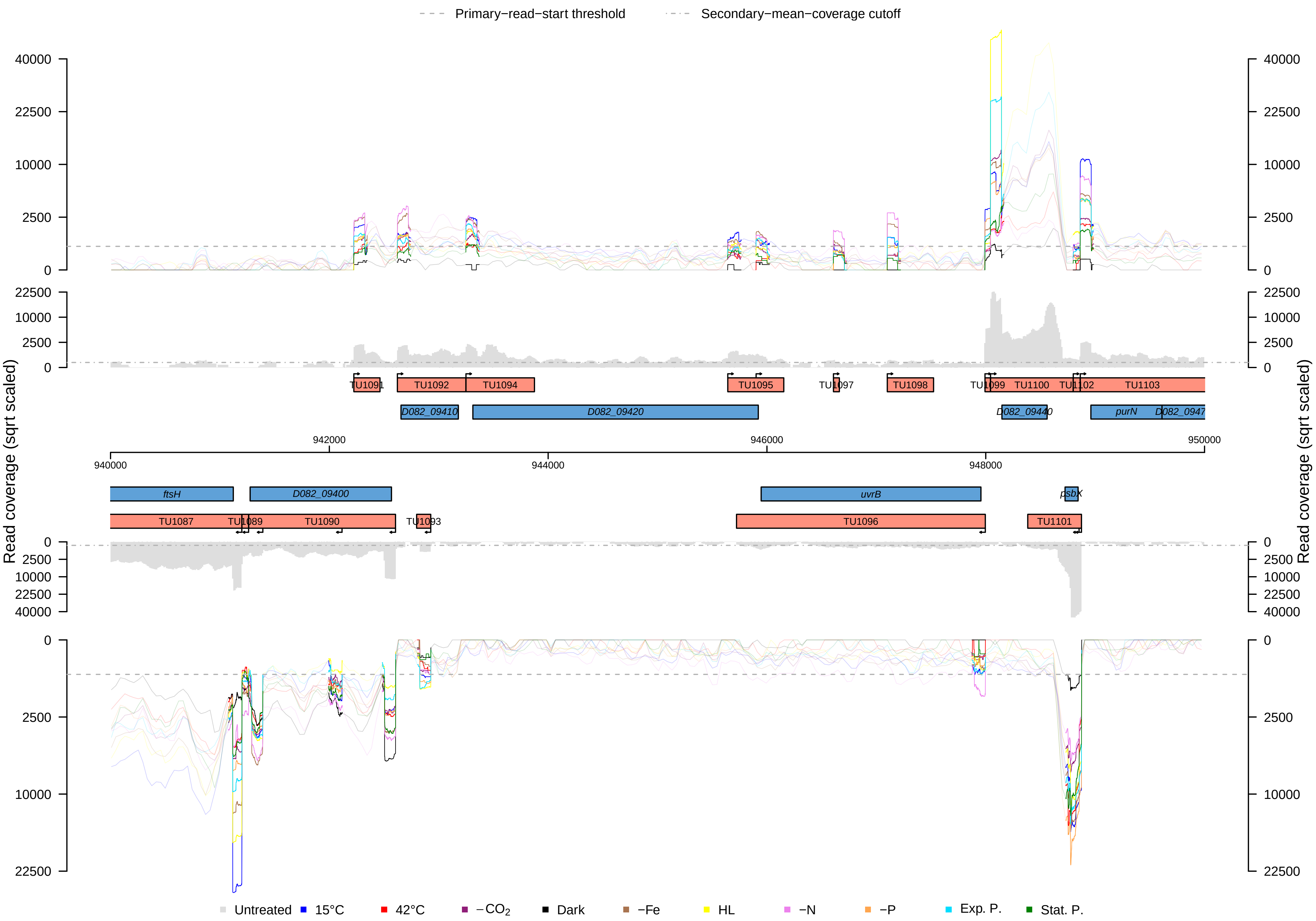

Primary−read−start threshold
Secondary−mean−coverage cutoff
Read coverage (sqrt scaled)
40000
22500
10000
2500
0
TU1091
TU1092
TU1094
TU1095
TU1097
TU1098
TU1099
TU1100
TU1102
TU1103
D082_09410
D082_09420
D082_09440
purN
D082_0947
942000
946000
950000
940000
944000
948000
ftsH
D082_09400
uvrB
psbX
TU1087
TU1089
TU1090
TU1093
TU1096
TU1101
Untreated
15°C
42°C
−CO2
Dark
−Fe
HL
−N
−P
Exp. P.
Stat. P.

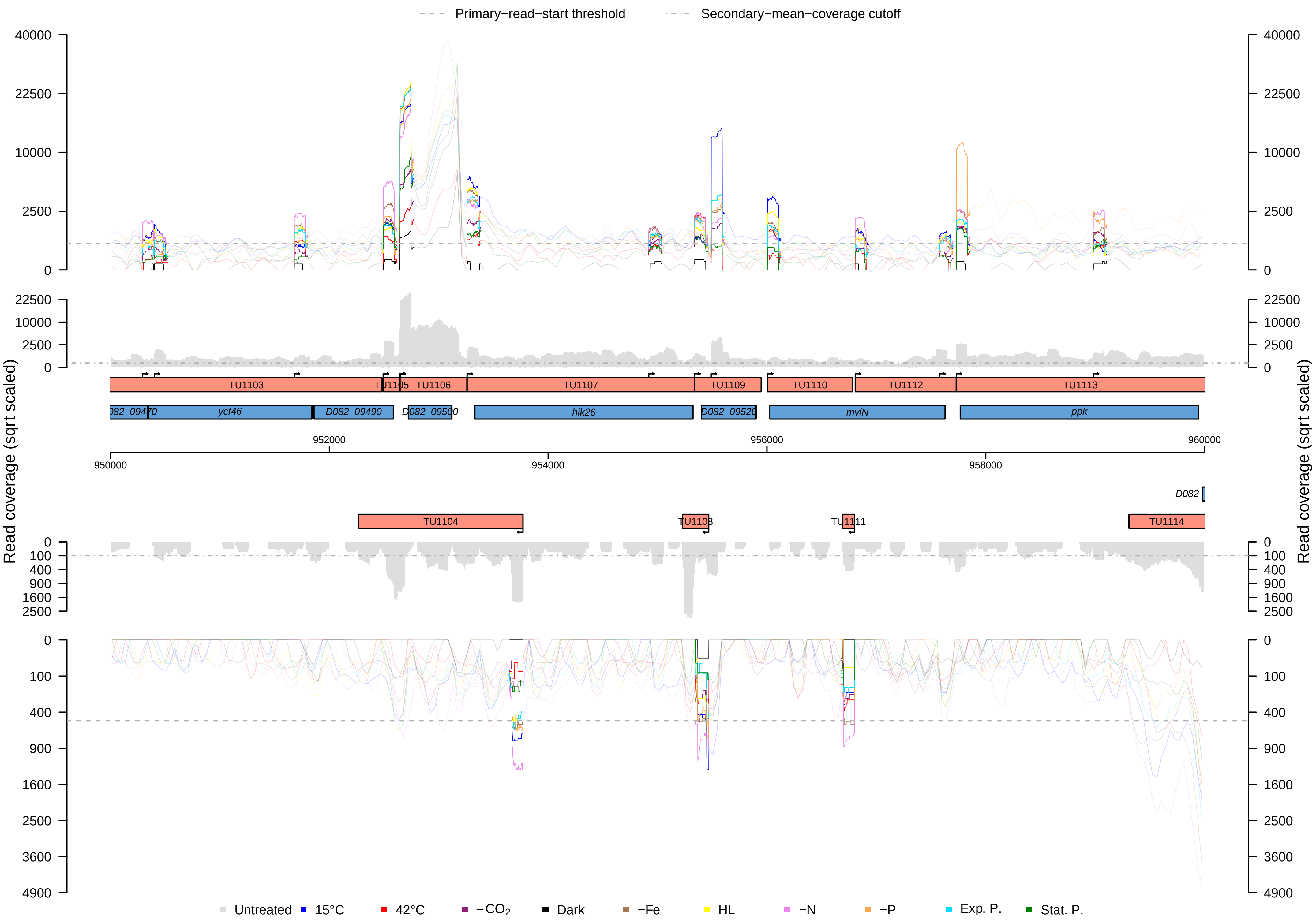

Primary−read−start threshold
Secondary−mean−coverage cutoff
Read coverage (sqrt scaled)
40000
22500
10000
2500
0
TU1103
TU1105
TU1106
TU1107
TU1109
TU1110
TU1112
TU1113
082_09470
ycf46
D082_09490
D082_09500
hik26
D082_09520
mviN
ppk
950000
952000
954000
956000
958000
960000
D082_
TU1104
TU1108
TU1111
TU1114
100
400
900
1600
3600
4900
Untreated
15°C
42°C
−CO2
Dark
−Fe
HL
−N
−P
Exp. P.
Stat. P.

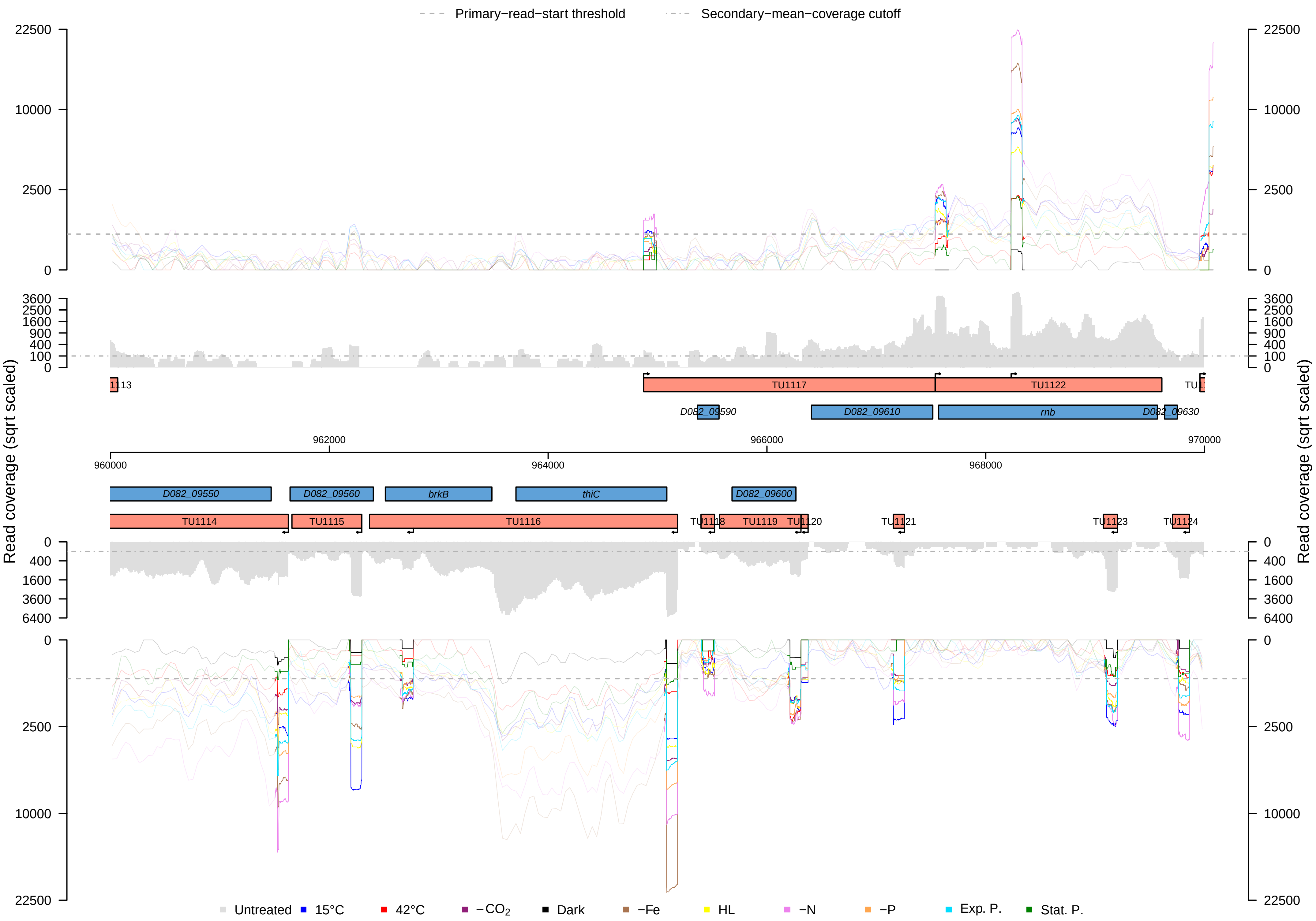

Primary−read−start threshold
Secondary−mean−coverage cutoff
22500
10000
2500
0
3600
2500
1600
900
400
100
0
Read coverage (sqrt scaled)
1113
TU1117
TU1122
D082_09590
D082_09610
rnb
D082_09630
962000
966000
970000
960000
964000
968000
D082_09550
D082_09560
brkB
thiC
D082_09600
TU1114
TU1115
TU1116
TU1118
TU1119
TU1120
TU1121
TU1123
TU1124
0
400
1600
3600
6400
0
2500
10000
22500
Untreated
15°C
42°C
−CO2
Dark
−Fe
HL
−N
−P
Exp. P.
Stat. P.

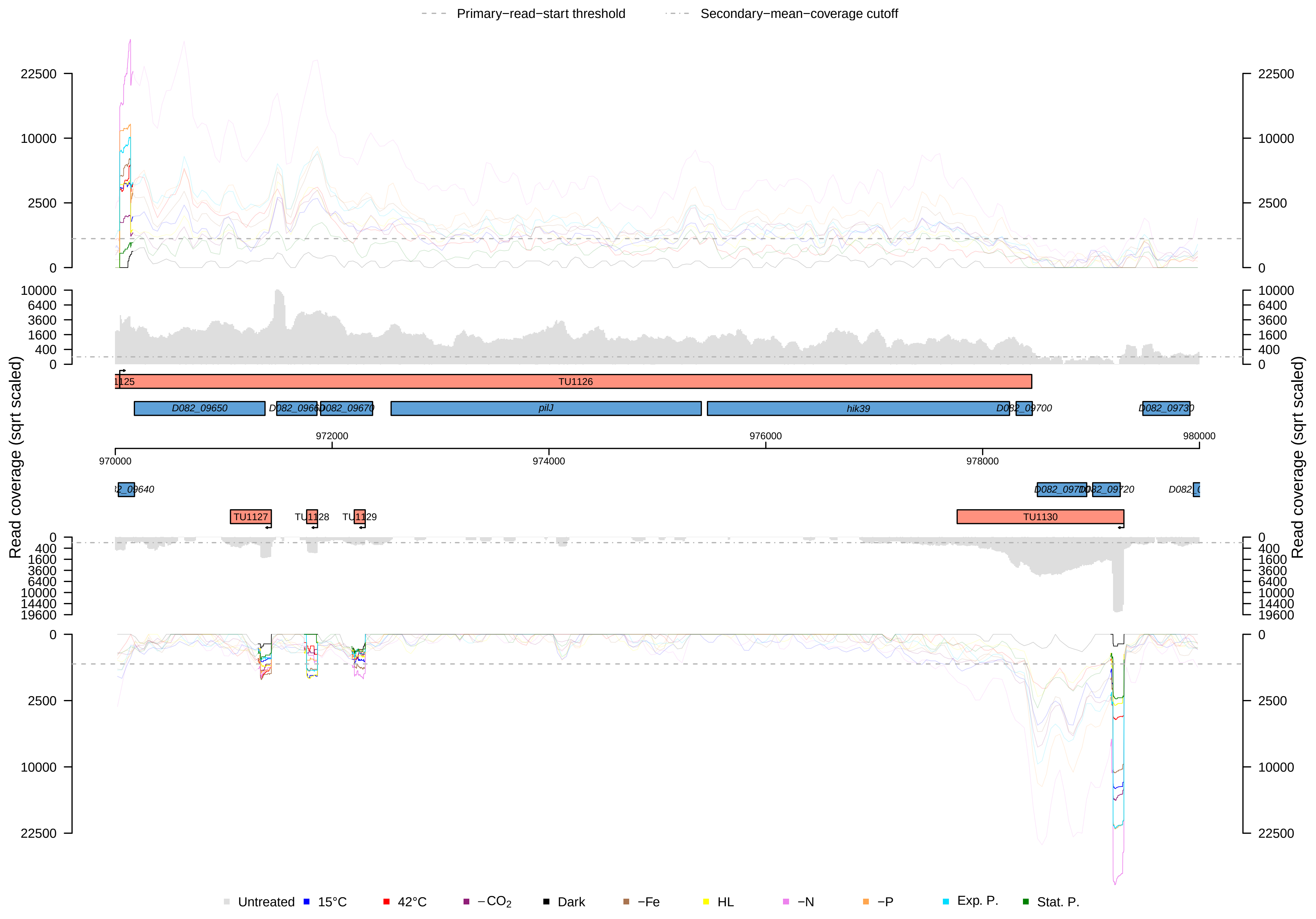

Primary−read−start threshold
Secondary−mean−coverage cutoff
Read coverage (sqrt scaled)
22500
10000
2500
0
10000
6400
3600
1600
400
0
TU1126
D082_09650
pilJ
hik39
D082_09730
972000
976000
980000
970000
974000
978000
TU1127
TU1130
Untreated
15°C
42°C
−CO2
Dark
−Fe
HL
−N
−P
Exp. P.
Stat. P.

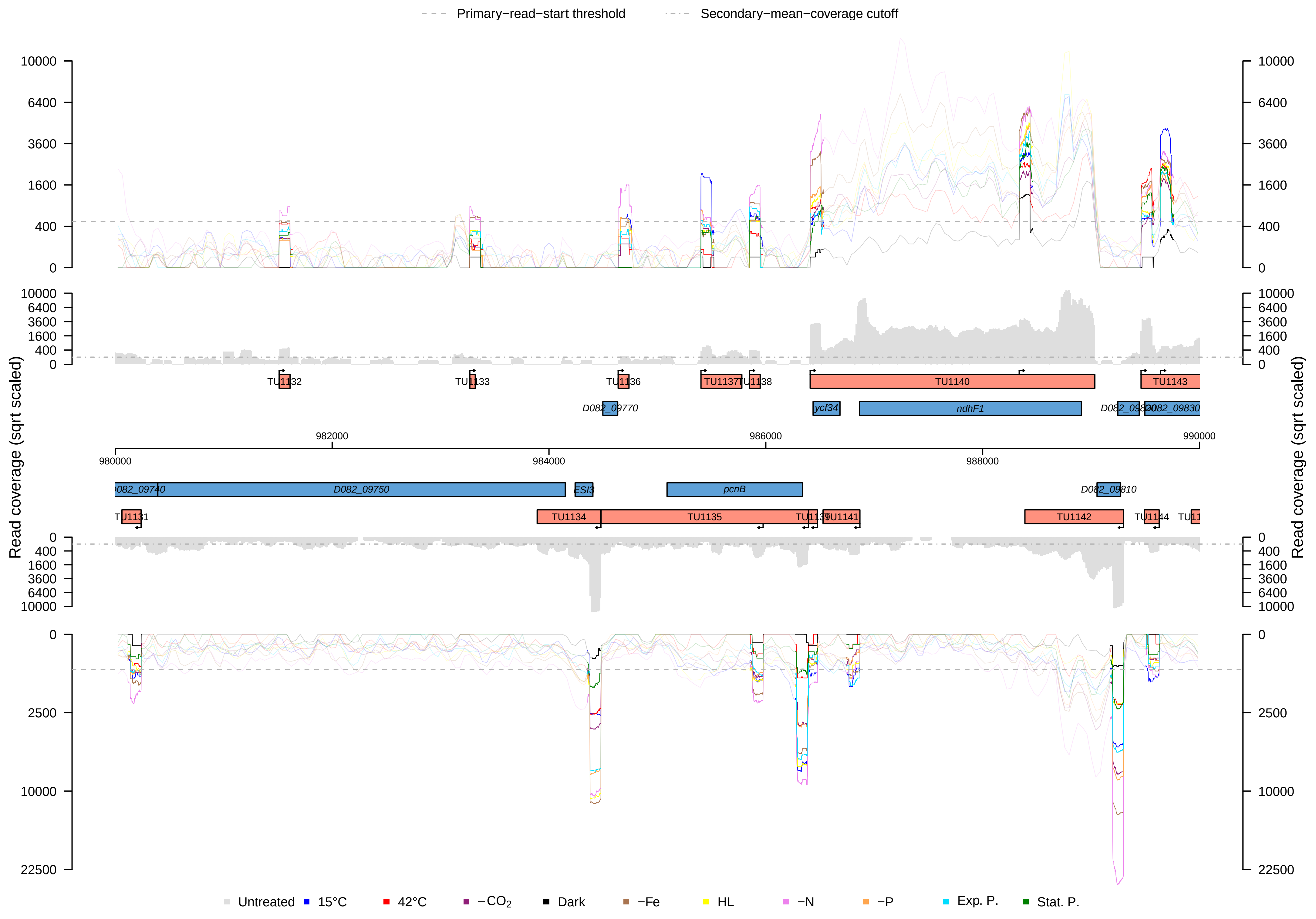

Primary−read−start threshold
Secondary−mean−coverage cutoff
Read coverage (sqrt scaled)
10000
6400
3600
1600
400
0
2500
22500
TU1131
TU1132
TU1133
TU1134
TU1135
TU1136
TU1137
TU1138
TU1140
TU1141
TU1142
TU1143
D082_09740
D082_09750
D082_09770
D082_09810
D082_09830
ESI3
pcnB
ycf34
ndhF1
980000
982000
984000
986000
988000
990000
Untreated
15°C
42°C
−CO2
Dark
−Fe
HL
−N
−P
Exp. P.
Stat. P.

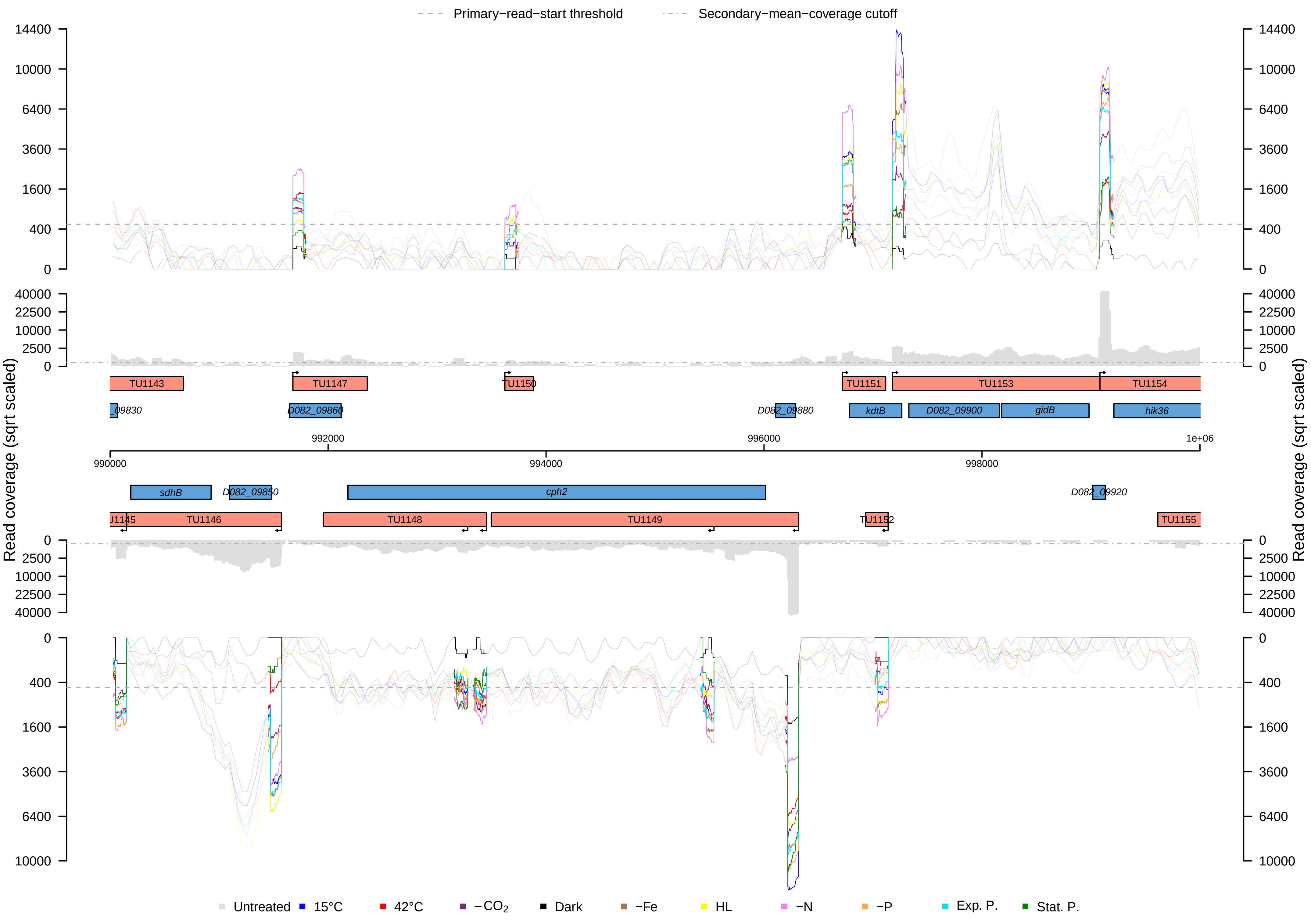

Primary−read−start threshold
Secondary−mean−coverage cutoff
Read coverage (sqrt scaled)
14400
10000
6400
3600
1600
400
0
40000
22500
10000
2500
0
TU1143
TU1147
TU1150
TU1151
TU1153
TU1154
_09830
D082_09860
D082_09880
kdtB
D082_09900
gidB
hik36
990000
992000
994000
996000
998000
1e+06
sdhB
D082_09850
cph2
D082_09920
J1145
TU1146
TU1148
TU1149
TU1152
TU1155
Untreated
15°C
42°C
−CO2
Dark
−Fe
HL
−N
−P
Exp. P.
Stat. P.

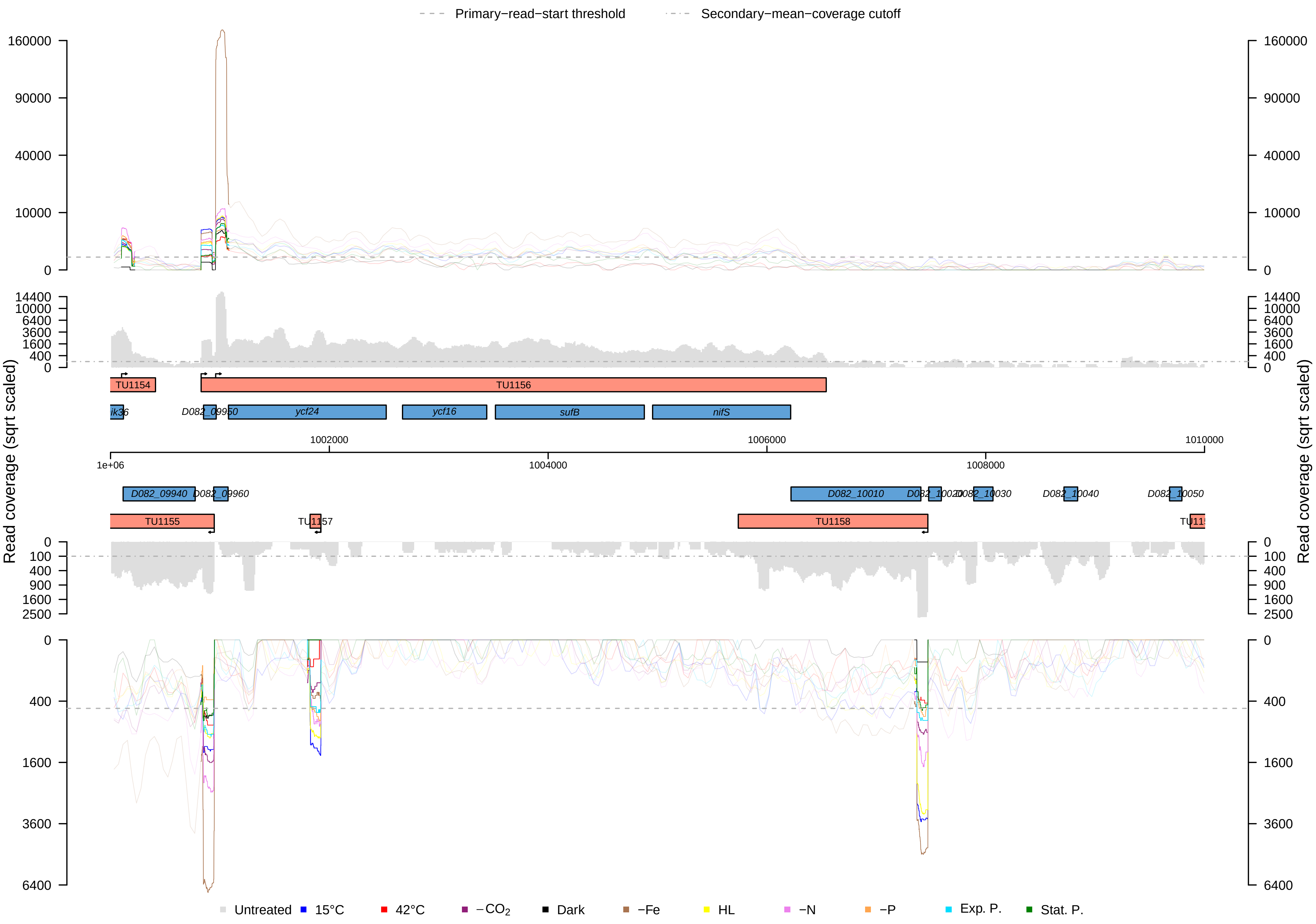

Primary−read−start threshold
Secondary−mean−coverage cutoff
Read coverage (sqrt scaled)
TU1154
TU1156
ik36
D082_09950
ycf24
ycf16
sufB
nifS
1e+06
1002000
1004000
1006000
1008000
1010000
D082_09940
D082_09960
D082_10010
D082_10020
D082_10030
D082_10040
D082_10050
TU1155
TU1157
TU1158
Untreated
15°C
42°C
−CO2
Dark
−Fe
HL
−N
−P
Exp. P.
Stat. P.

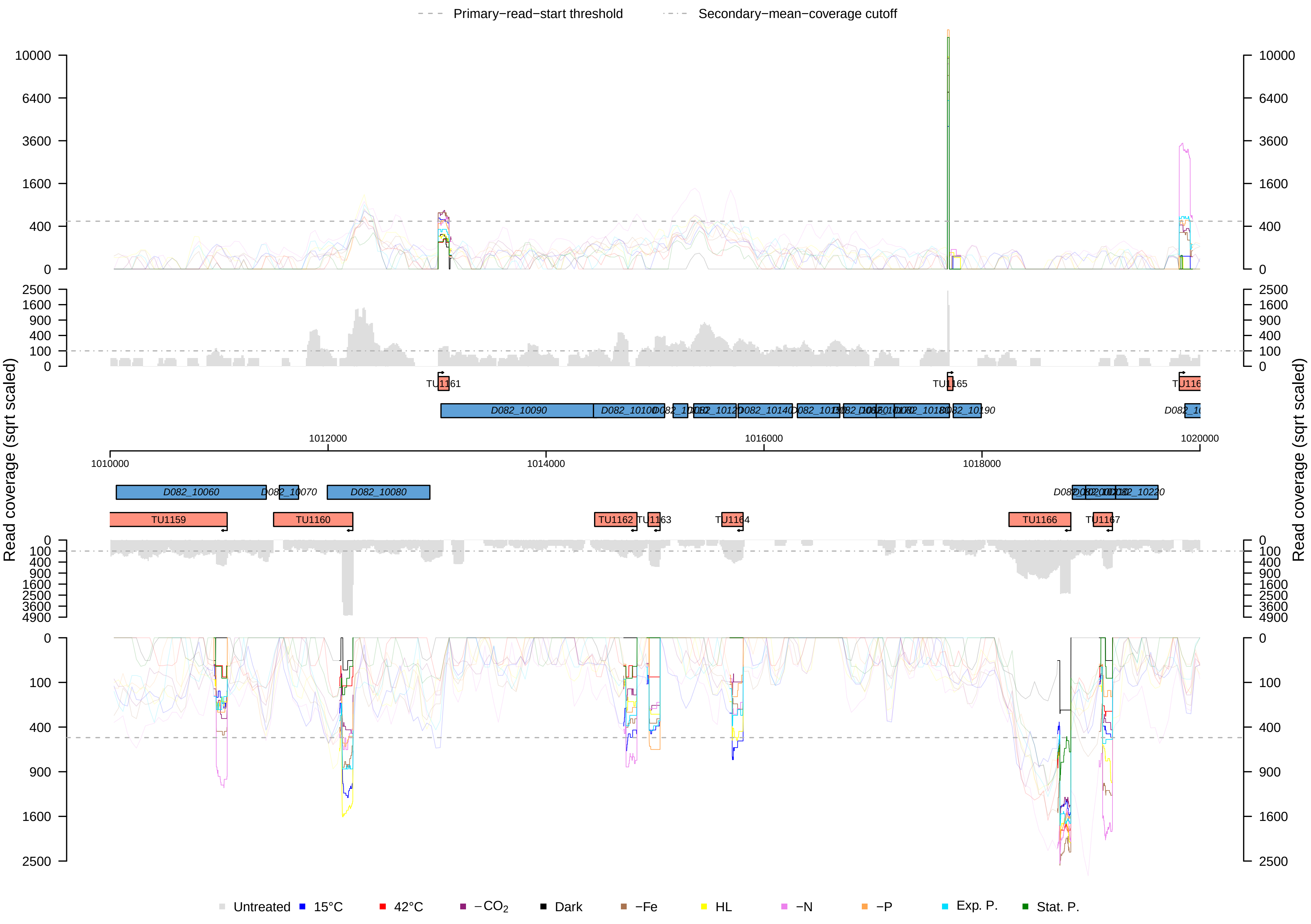

Primary−read−start threshold
Secondary−mean−coverage cutoff
Read coverage (sqrt scaled)
TU1159
TU1160
TU1161
TU1162
TU1163
TU1164
TU1165
TU1166
TU1167
D082_10060
D082_10070
D082_10080
D082_10090
D082_10190
D082_10220
1010000
1012000
1014000
1016000
1018000
1020000
Untreated
15°C
42°C
−CO2
Dark
−Fe
HL
−N
−P
Exp. P.
Stat. P.

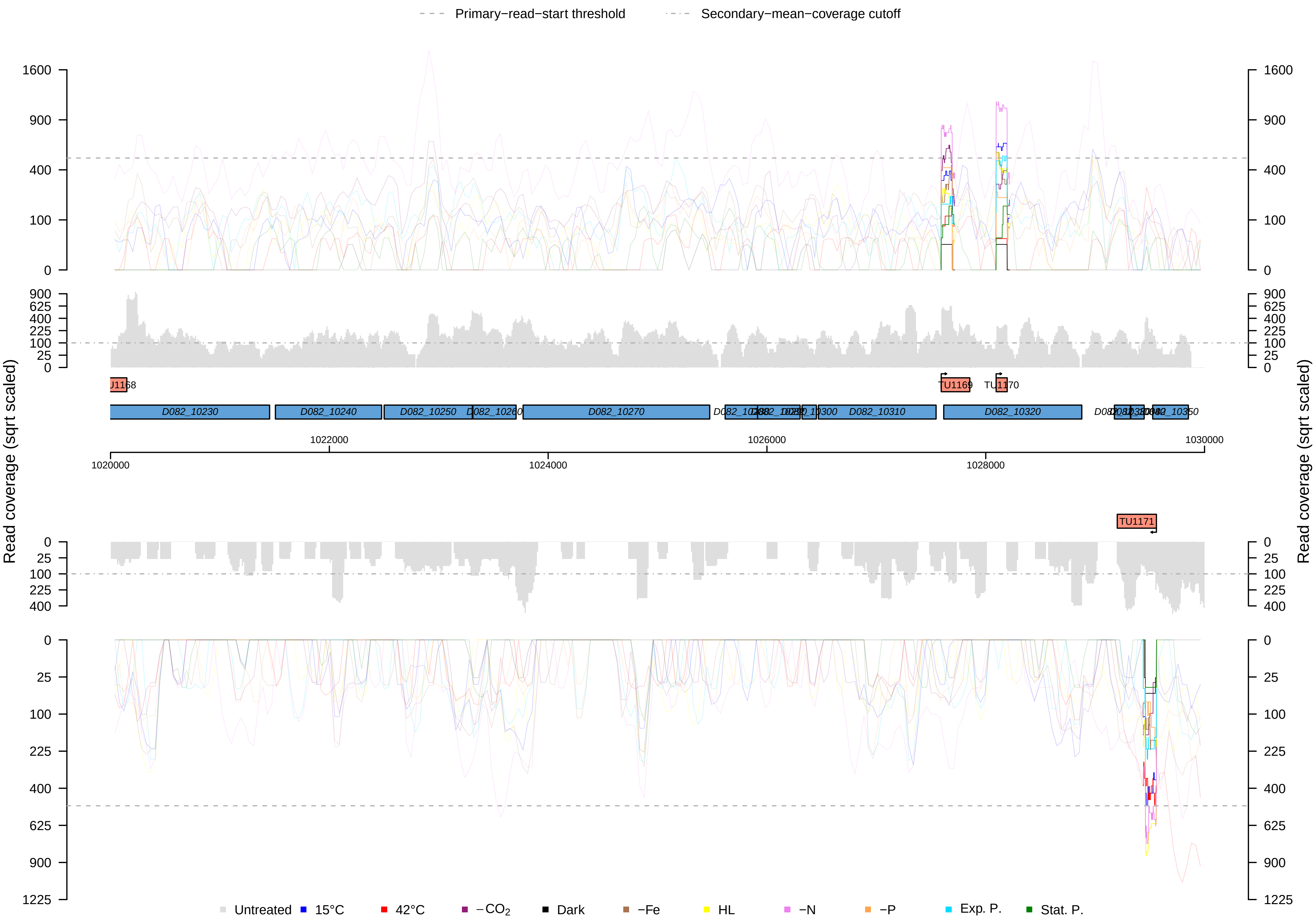

Primary−read−start threshold
Secondary−mean−coverage cutoff
Read coverage (sqrt scaled)
1600
900
400
100
0
900
625
400
225
100
25
0
TU1168
TU1169
TU1170
TU1171
D082_10230
D082_10240
D082_10250
D082_10260
D082_10270
D082_10300
D082_10310
D082_10320
1020000
1022000
1024000
1026000
1028000
1030000
1225
Untreated
15°C
42°C
−CO_2
Dark
−Fe
HL
−N
−P
Exp. P.
Stat. P.

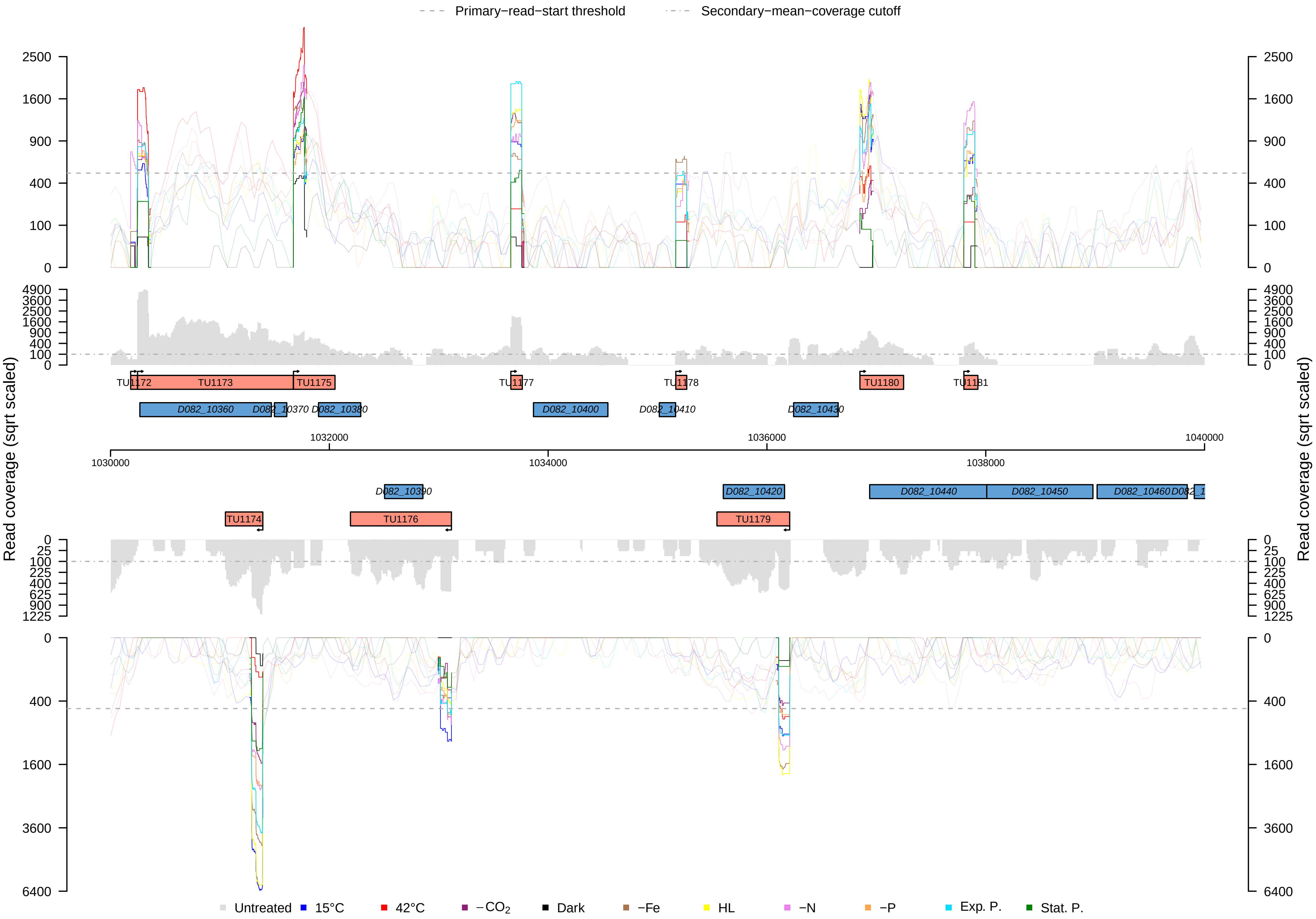

Primary−read−start threshold
Secondary−mean−coverage cutoff
Read coverage (sqrt scaled)
2500
1600
900
400
100
0
4900
3600
2500
1600
900
400
100
0
TU1172
TU1173
TU1175
TU1177
TU1178
TU1180
TU1181
D082_10360
D082_10370
D082_10380
D082_10400
D082_10410
D082_10430
1030000
1032000
1034000
1036000
1038000
1040000
D082_10390
D082_10420
D082_10440
D082_10450
D082_10460
TU1174
TU1176
TU1179
0
25
100
225
400
625
900
1225
0
400
1600
3600
6400
Untreated
15°C
42°C
−CO2
Dark
−Fe
HL
−N
−P
Exp. P.
Stat. P.

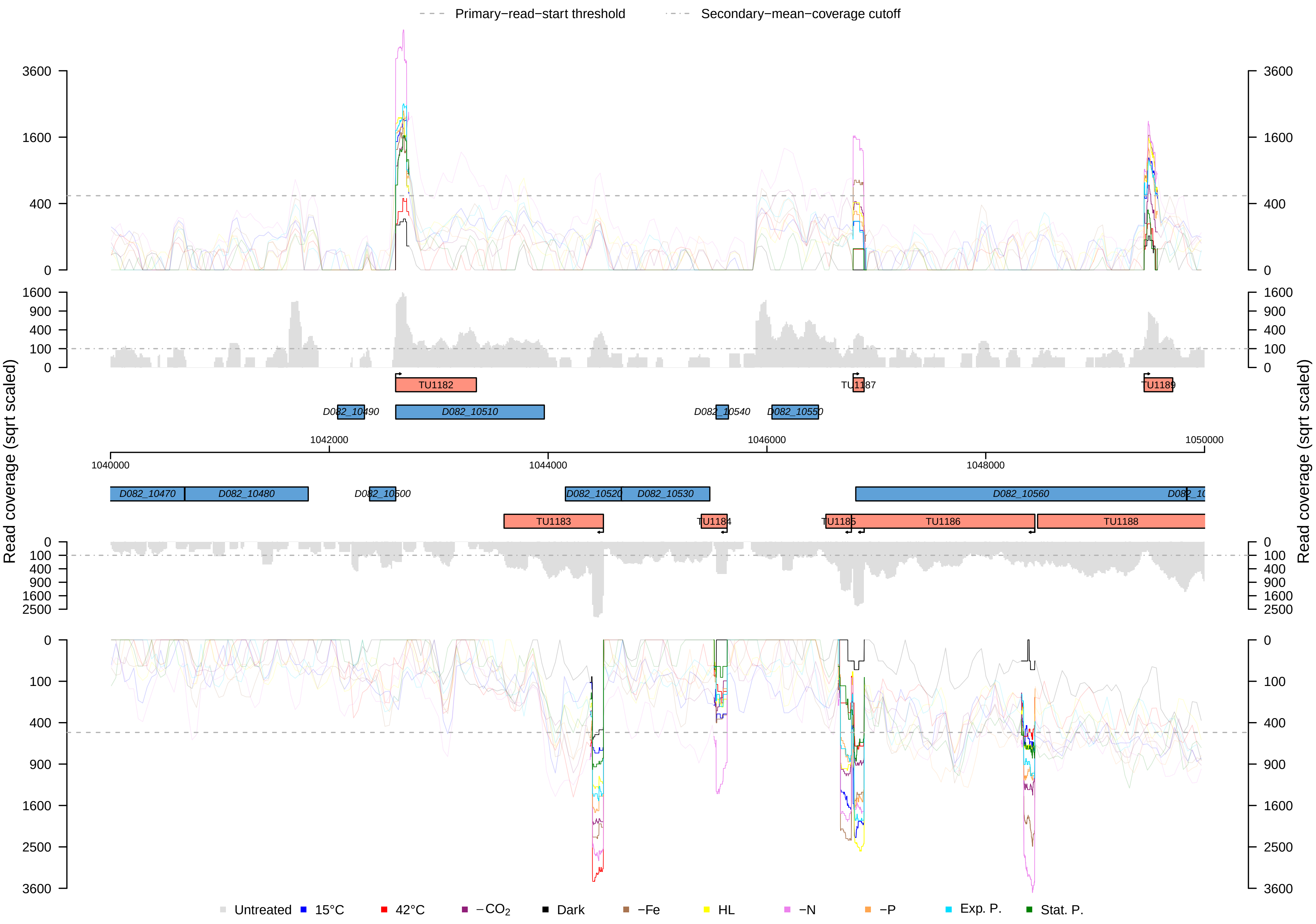

Primary−read−start threshold
Secondary−mean−coverage cutoff
Read coverage (sqrt scaled)
3600
1600
400
0
1600
900
400
100
0
TU1182
TU1187
TU1189
D082_10490
D082_10510
D082_10540
D082_10550
1040000
1042000
1044000
1046000
1048000
1050000
D082_10470
D082_10480
D082_10500
D082_10520
D082_10530
D082_10560
TU1183
TU1184
TU1185
TU1186
TU1188
0
100
400
900
1600
2500
3600
Untreated
15°C
42°C
−CO2
Dark
−Fe
HL
−N
−P
Exp. P.
Stat. P.

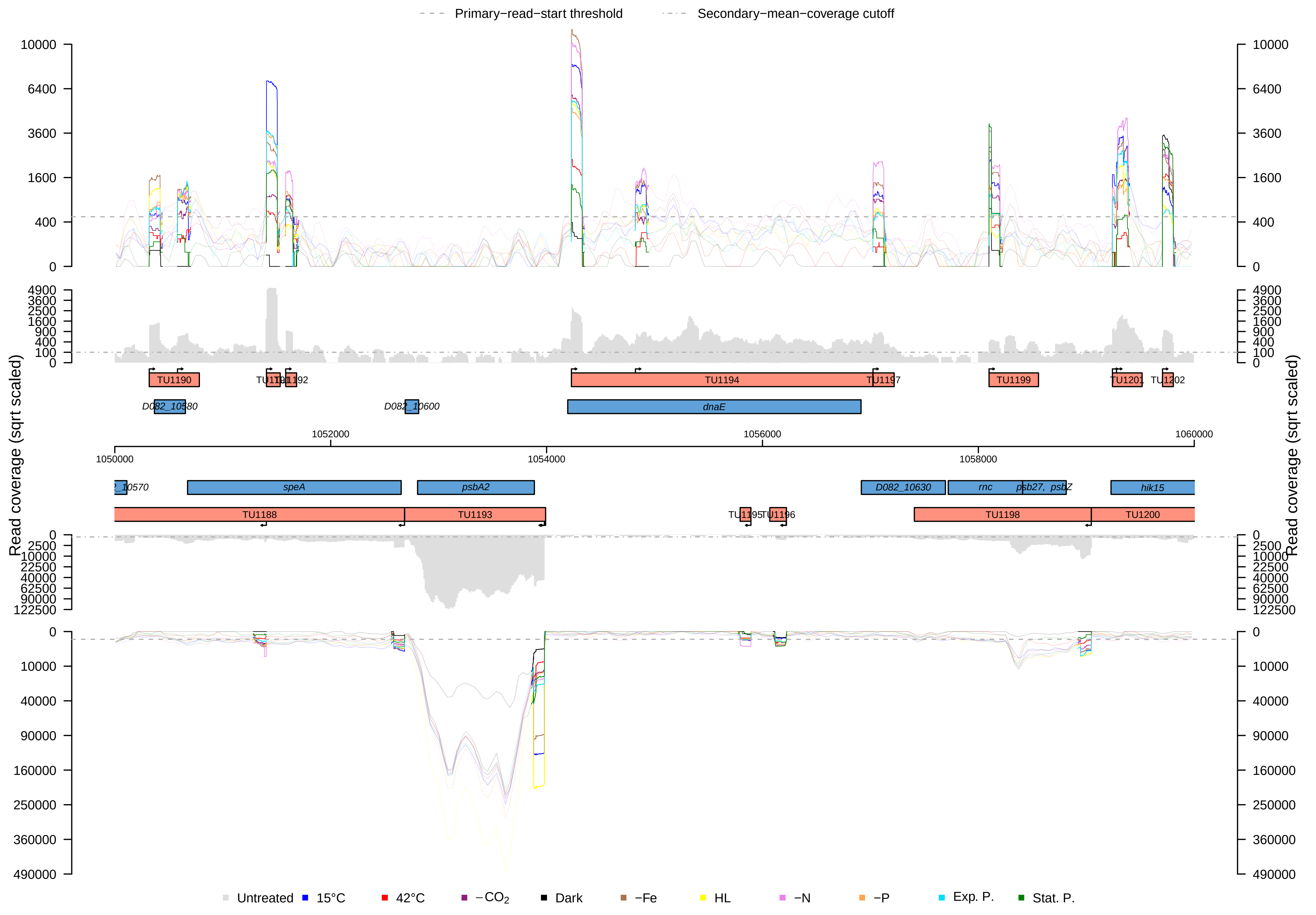

Primary−read−start threshold
Secondary−mean−coverage cutoff
Read coverage (sqrt scaled)
TU1190
TU1191
TU1192
TU1194
TU1197
TU1199
TU1201
TU1202
D082_10580
D082_10600
dnaE
1050000
1052000
1054000
1056000
1058000
1060000
_10570
speA
psbA2
D082_10630
rnc
psb27, psbZ
hik15
TU1188
TU1193
TU1195
TU1196
TU1198
TU1200
Untreated
15°C
42°C
−CO2
Dark
−Fe
HL
−N
−P
Exp. P.
Stat. P.

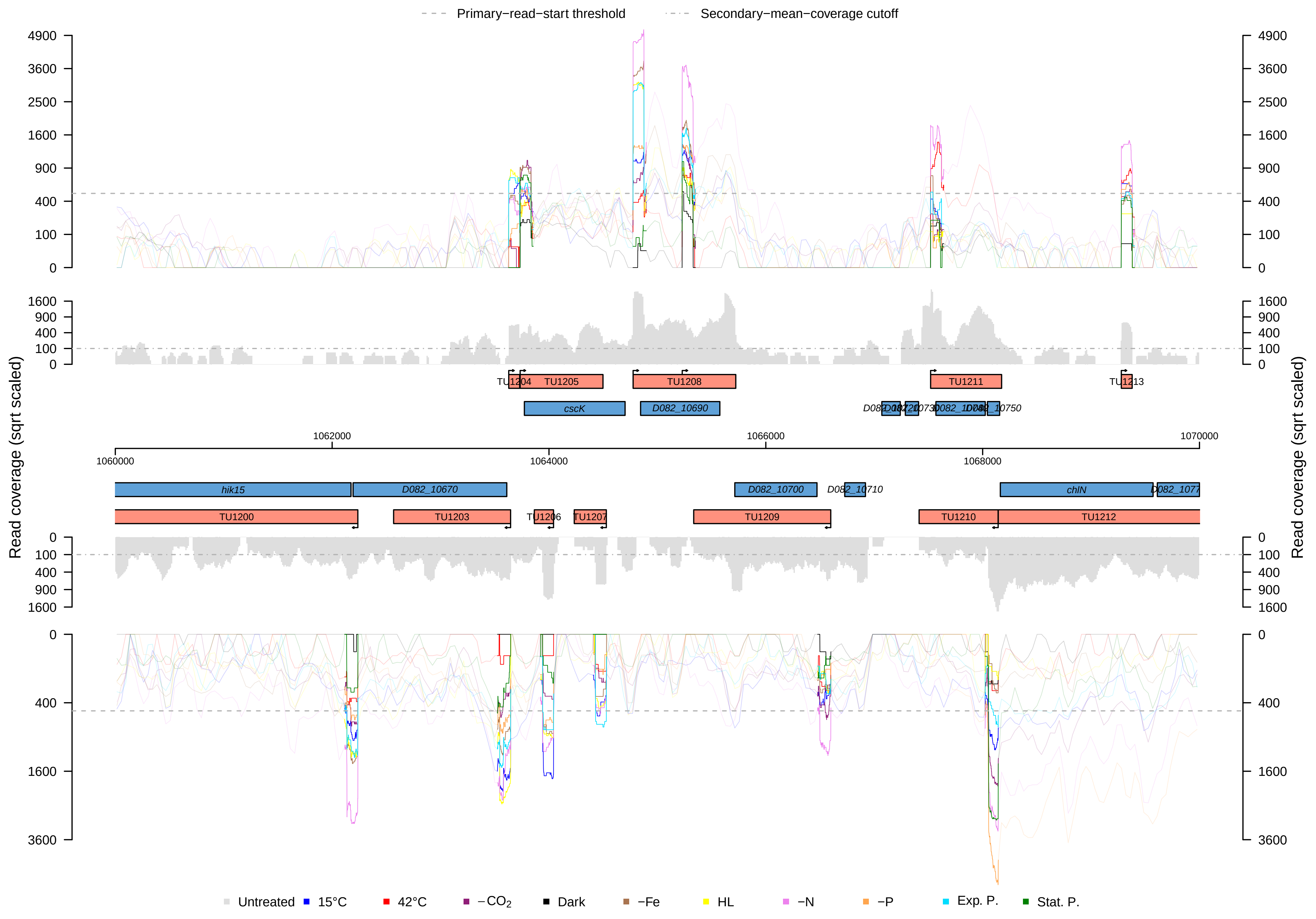

Primary−read−start threshold
Secondary−mean−coverage cutoff
Read coverage (sqrt scaled)
4900
3600
2500
1600
900
400
100
0
TU1204
TU1205
TU1208
TU1211
TU1213
cscK
D082_10690
D082_10750
1060000
1062000
1064000
1066000
1068000
1070000
hik15
D082_10670
D082_10700
D082_10710
chlN
TU1200
TU1203
TU1206
TU1207
TU1209
TU1210
TU1212
Untreated
15°C
42°C
−CO2
Dark
−Fe
HL
−N
−P
Exp. P.
Stat. P.

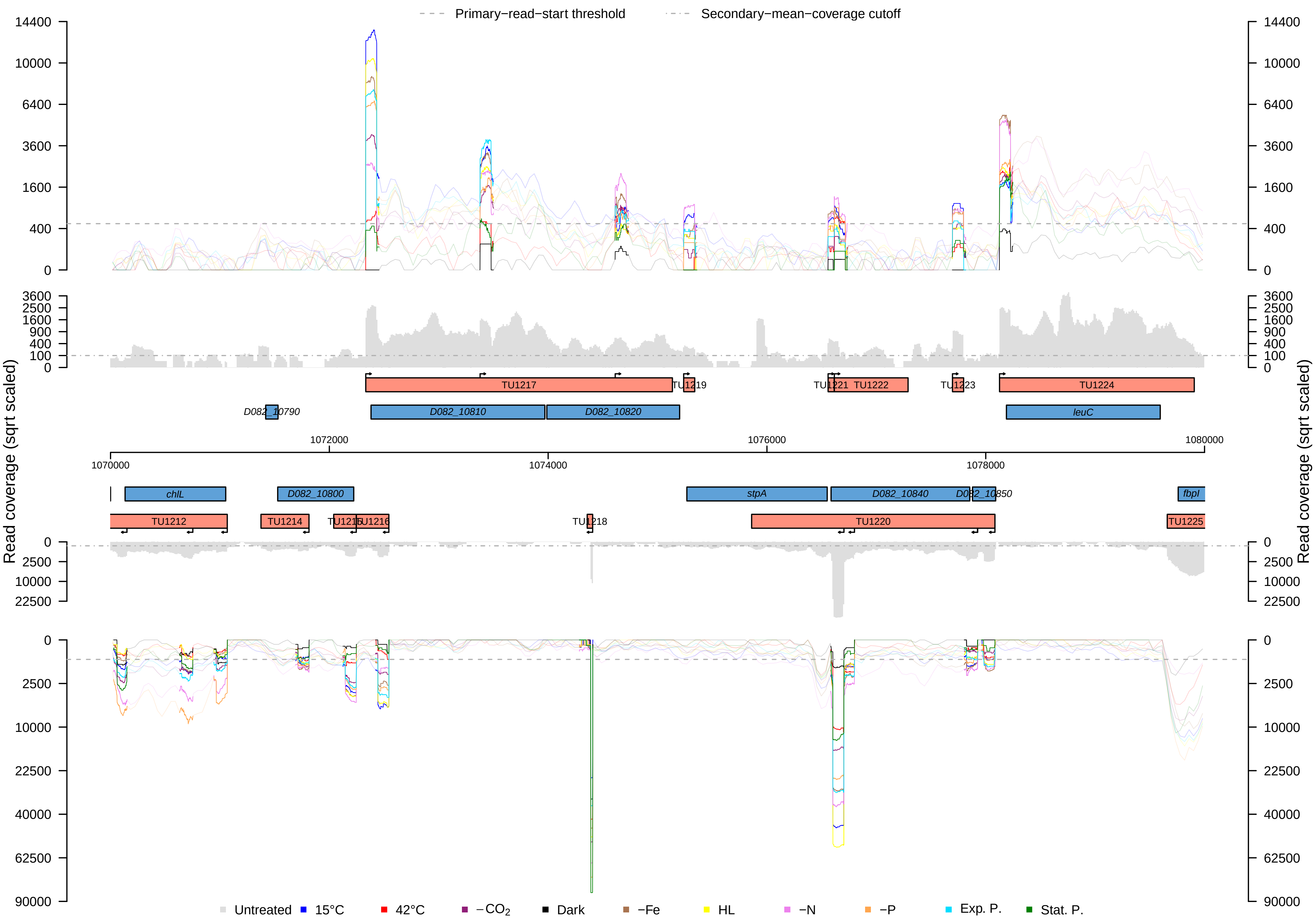

Primary−read−start threshold
Secondary−mean−coverage cutoff
Read coverage (sqrt scaled)
14400
10000
6400
3600
1600
400
0
2500
900
100
22500
40000
62500
90000
TU1217
TU1219
TU1221
TU1222
TU1223
TU1224
D082_10790
D082_10810
D082_10820
leuC
1070000
1072000
1074000
1076000
1078000
1080000
chlL
D082_10800
stpA
D082_10840
D082_10850
fbpI
TU1212
TU1214
TU1215
TU1216
TU1218
TU1220
TU1225
Untreated
15°C
42°C
$-CO_2$
Dark
−Fe
HL
−N
−P
Exp. P.
Stat. P.

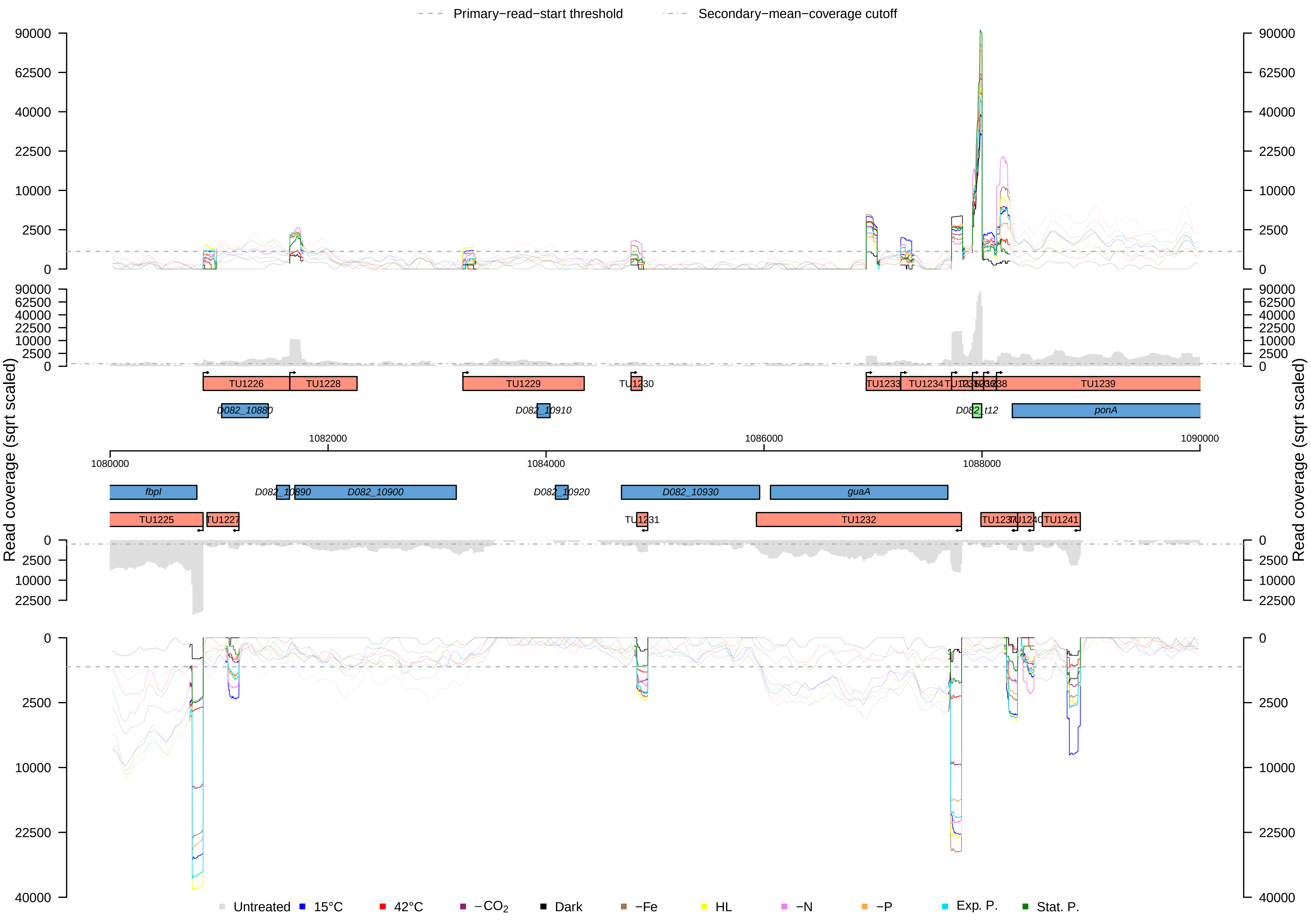

Primary−read−start threshold
Secondary−mean−coverage cutoff
Read coverage (sqrt scaled)
TU1226
TU1228
TU1229
TU1230
TU1233
TU1234
TU1239
D082_10880
D082_10910
ponA
fbpI
D082_10890
D082_10900
D082_10920
D082_10930
guaA
TU1225
TU1227
TU1231
TU1232
TU1241
1080000
1082000
1084000
1086000
1088000
1090000
Untreated
15°C
42°C
−CO2
Dark
−Fe
HL
−N
−P
Exp. P.
Stat. P.

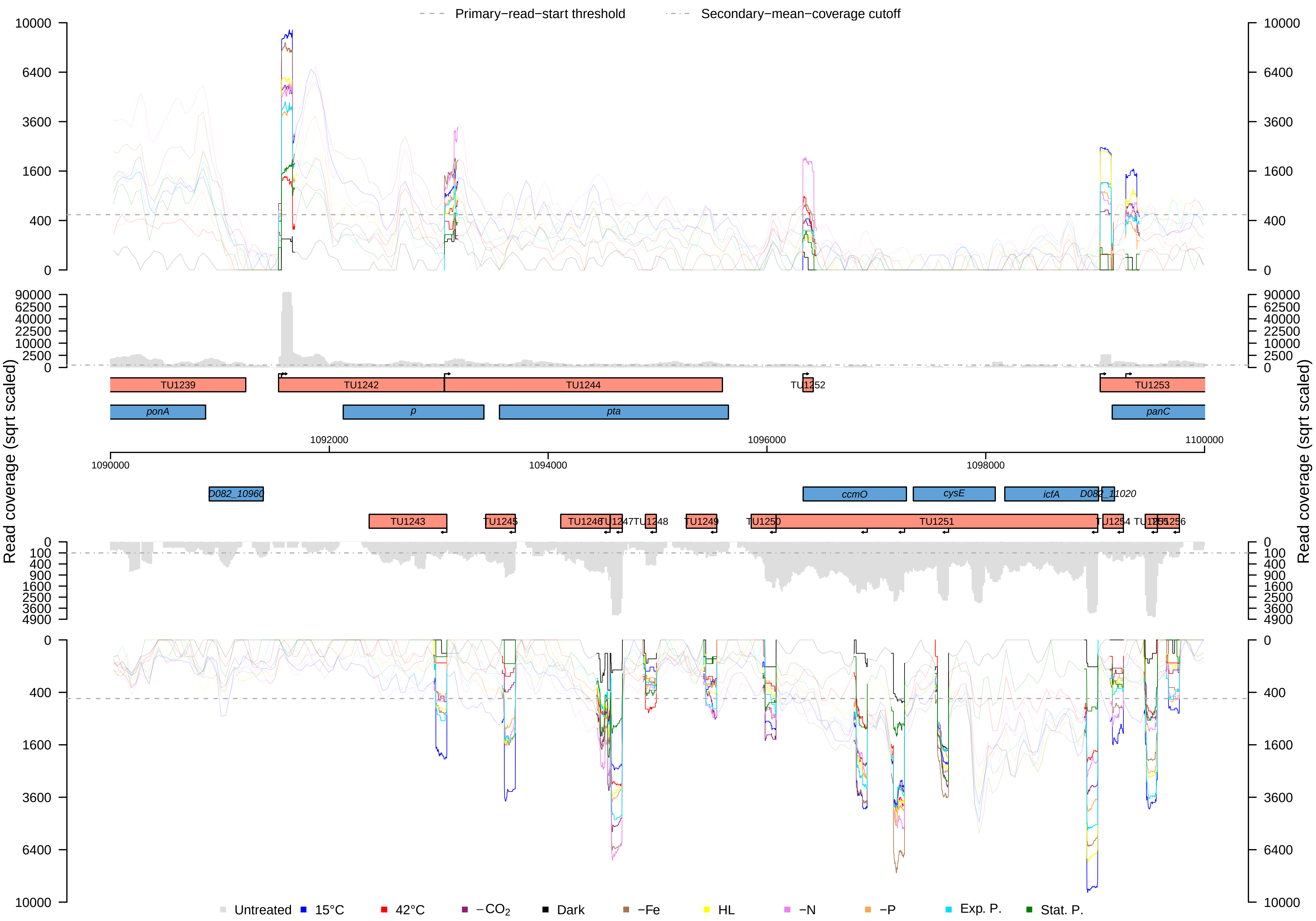

Primary−read−start threshold
Secondary−mean−coverage cutoff
Read coverage (sqrt scaled)
TU1239
TU1242
TU1244
TU1252
TU1253
ponA
p
pta
panC
1090000
1092000
1094000
1096000
1098000
1100000
D082_10960
ccmO
cysE
icfA
D082_11020
TU1243
TU1245
TU1246
TU1247
TU1248
TU1249
TU1250
TU1251
TU1254
TU1256
Untreated
15°C
42°C
−CO_2
Dark
−Fe
HL
−N
−P
Exp. P.
Stat. P.

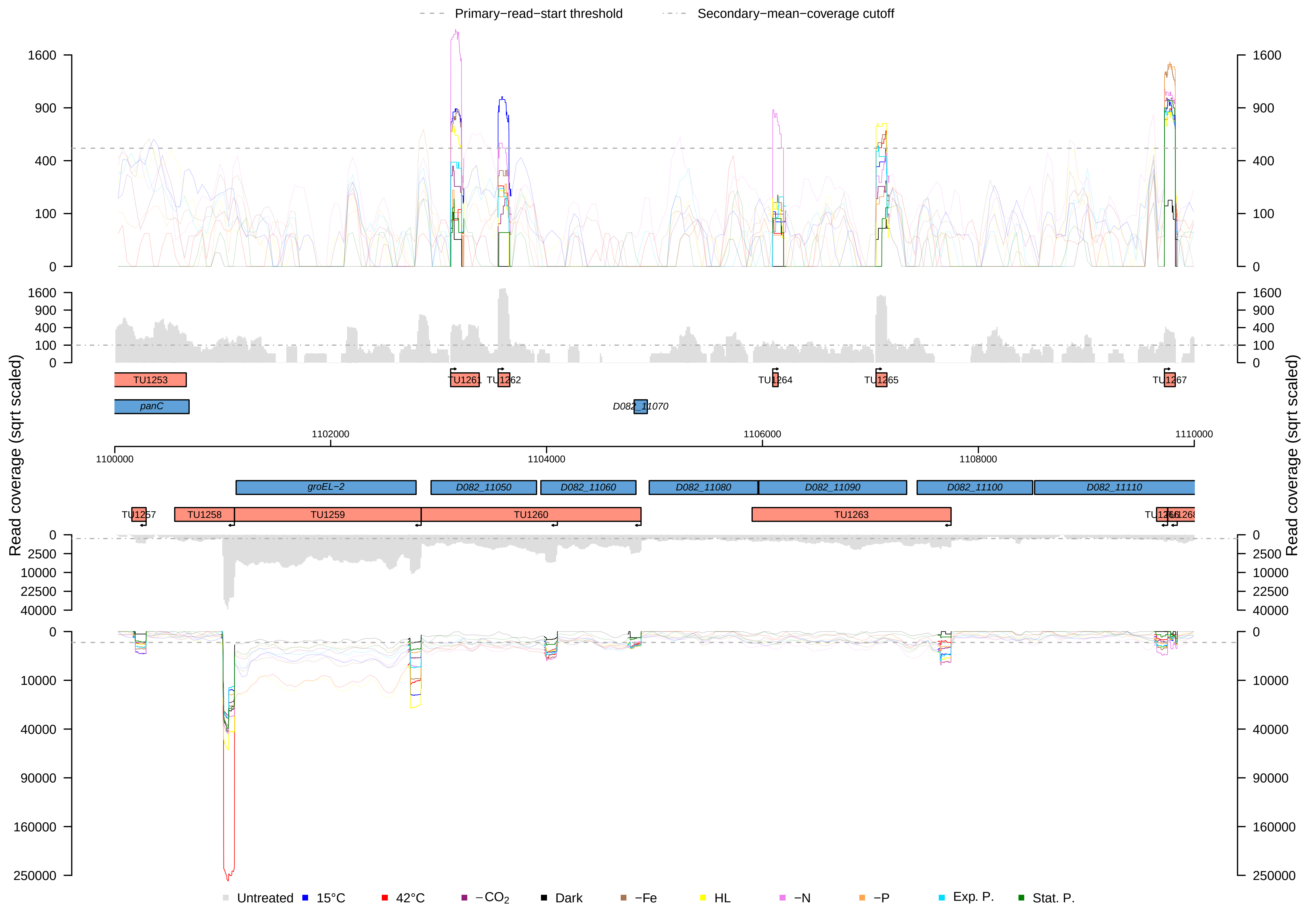

Primary−read−start threshold
Secondary−mean−coverage cutoff
Read coverage (sqrt scaled)
1600
900
400
100
0
TU1253
panC
TU1261
TU1262
TU1264
TU1265
TU1267
D082_11070
1100000
1102000
1104000
1106000
1108000
1110000
groEL−2
D082_11050
D082_11060
D082_11080
D082_11090
D082_11100
D082_11110
TU1257
TU1258
TU1259
TU1260
TU1263
2500
10000
22500
40000
90000
160000
250000
Untreated
15°C
42°C
−CO2
Dark
−Fe
HL
−N
−P
Exp. P.
Stat. P.

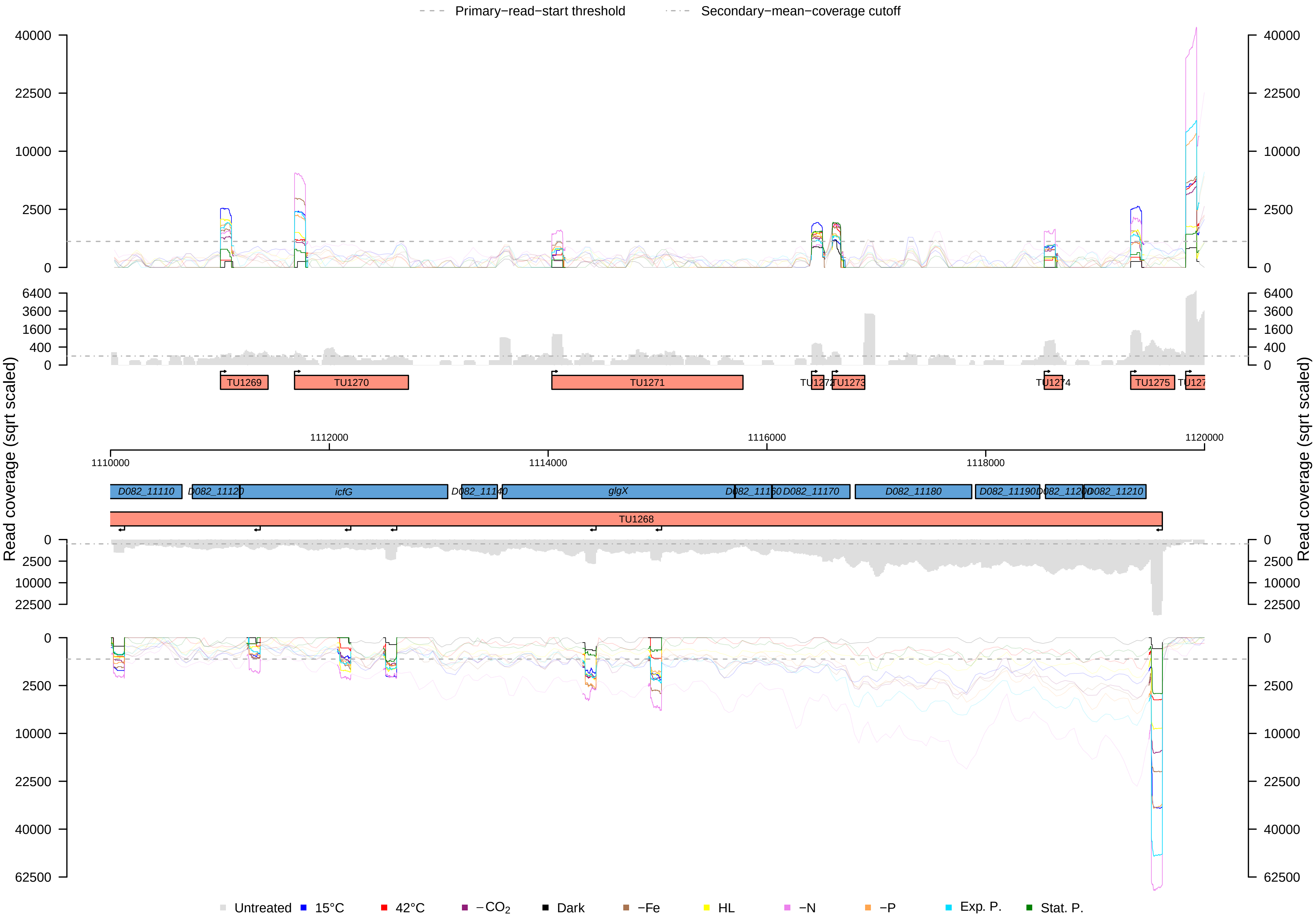

Primary−read−start threshold
Secondary−mean−coverage cutoff
Read coverage (sqrt scaled)
TU1268
TU1269
TU1270
TU1271
TU1273
TU1275
D082_11110
icfG
glgX
D082_11170
D082_11180
D082_11210
1110000
1112000
1114000
1116000
1118000
1120000
Untreated
15°C
42°C
−CO2
Dark
−Fe
HL
−N
−P
Exp. P.
Stat. P.

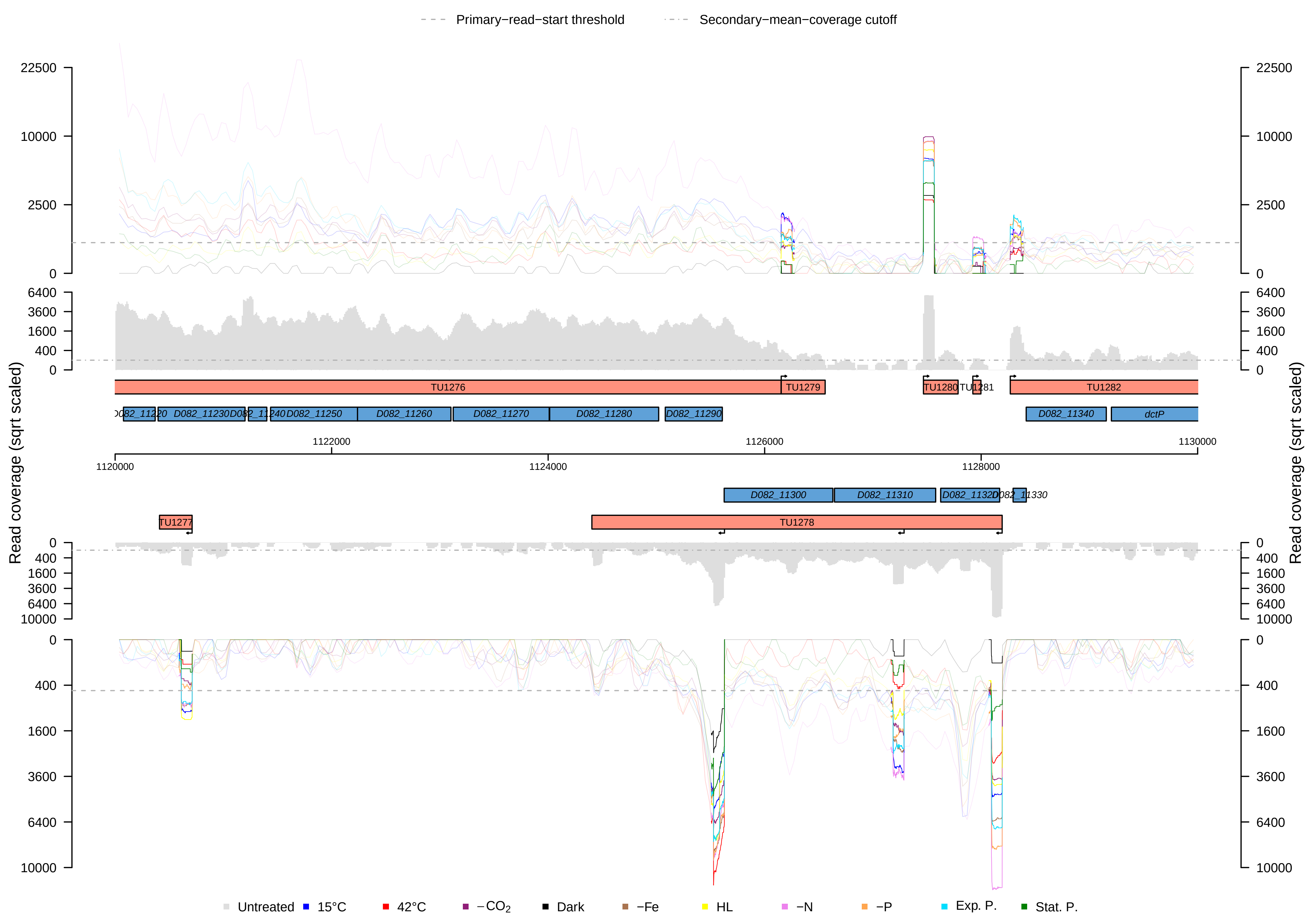

Primary−read−start threshold
Secondary−mean−coverage cutoff
Read coverage (sqrt scaled)
TU1276
TU1279
TU1280
TU1281
TU1282
TU1277
TU1278
D082_11220
D082_11230
D082_11240
D082_11250
D082_11260
D082_11270
D082_11280
D082_11290
D082_11300
D082_11310
D082_11320
D082_11330
D082_11340
dctP
1120000
1122000
1124000
1126000
1128000
1130000
Untreated
15°C
42°C
−CO2
Dark
−Fe
HL
−N
−P
Exp. P.
Stat. P.

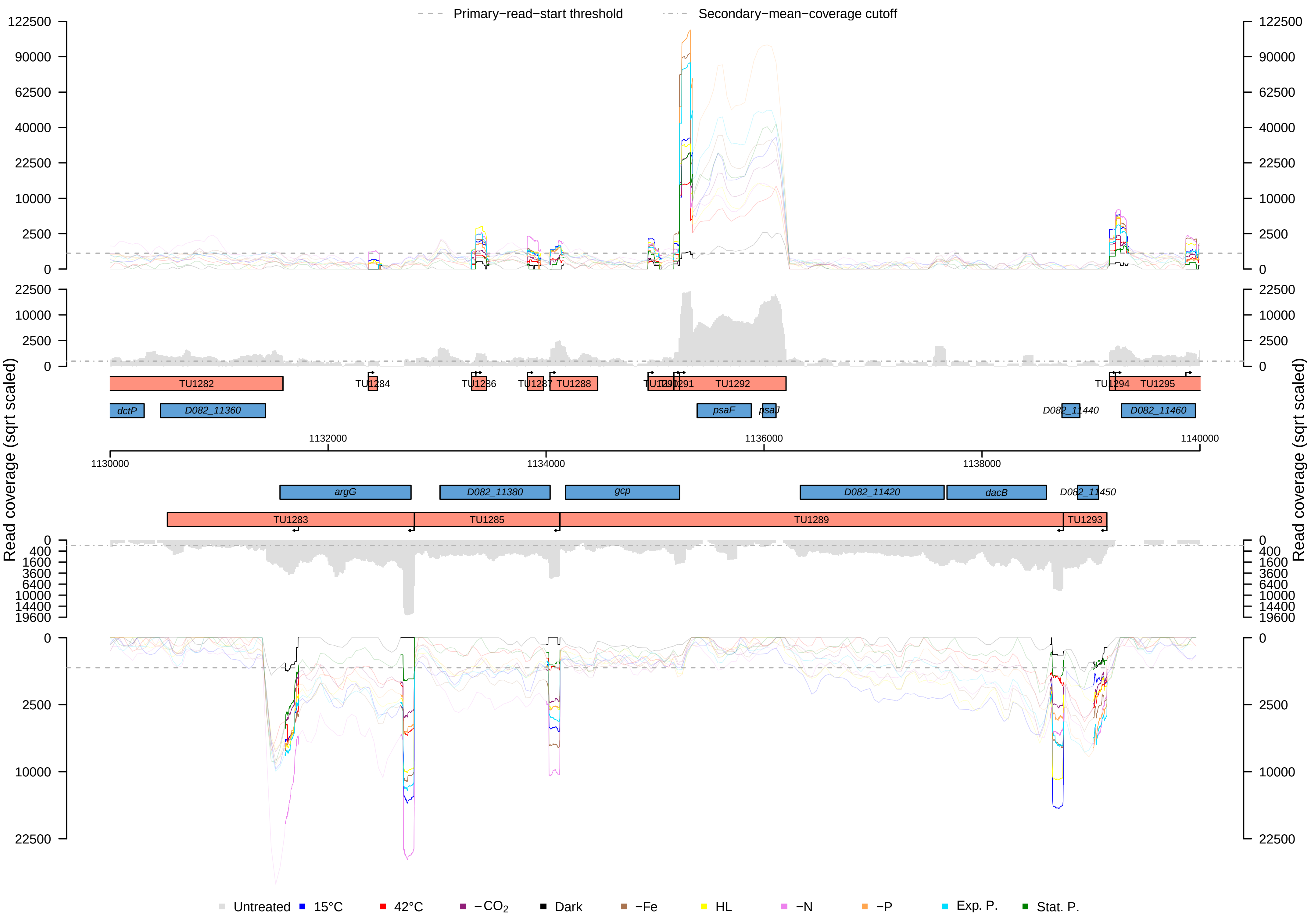

Primary−read−start threshold
Secondary−mean−coverage cutoff
Read coverage (sqrt scaled)
TU1282
TU1284
TU1286
TU1287
TU1288
TU1292
TU1294
TU1295
dctP
D082_11360
psaF
psaJ
D082_11440
D082_11460
1130000
1132000
1134000
1136000
1138000
1140000
argG
D082_11380
gcp
D082_11420
dacB
D082_11450
TU1283
TU1285
TU1289
TU1293
Untreated
15°C
42°C
−CO2
Dark
−Fe
HL
−N
−P
Exp. P.
Stat. P.

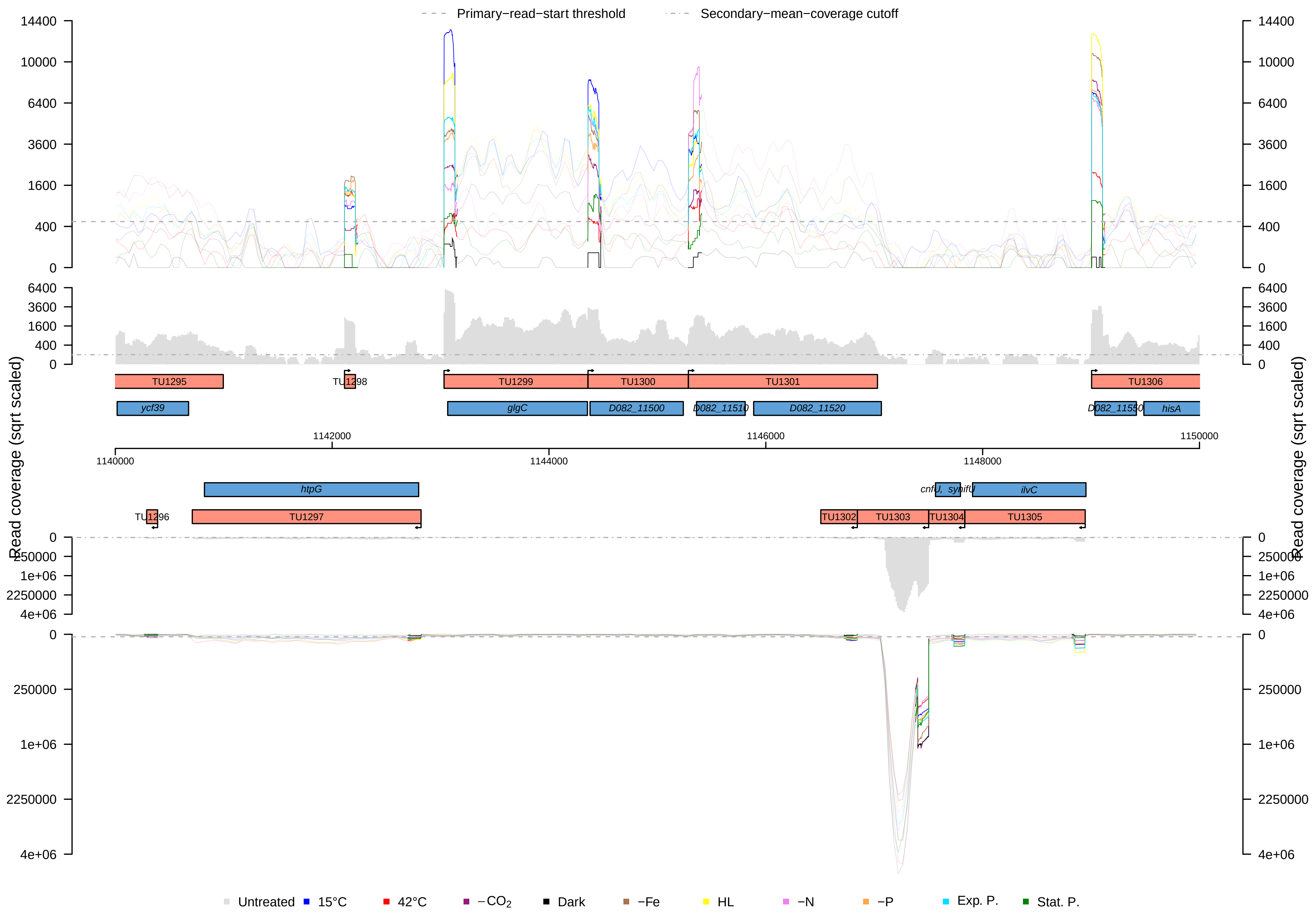

Primary−read−start threshold
Secondary−mean−coverage cutoff
Read coverage (sqrt scaled)
14400
10000
6400
3600
1600
400
0
TU1295
TU1298
TU1299
TU1300
TU1301
TU1306
ycf39
glgC
D082_11500
D082_11510
D082_11520
D082_11550
hisA
1140000
1142000
1144000
1146000
1148000
1150000
htpG
cnfU, synifU
ilvC
TU1296
TU1297
TU1302
TU1303
TU1304
TU1305
250000
1e+06
2250000
4e+06
Untreated
15°C
42°C
−CO2
Dark
−Fe
HL
−N
−P
Exp. P.
Stat. P.

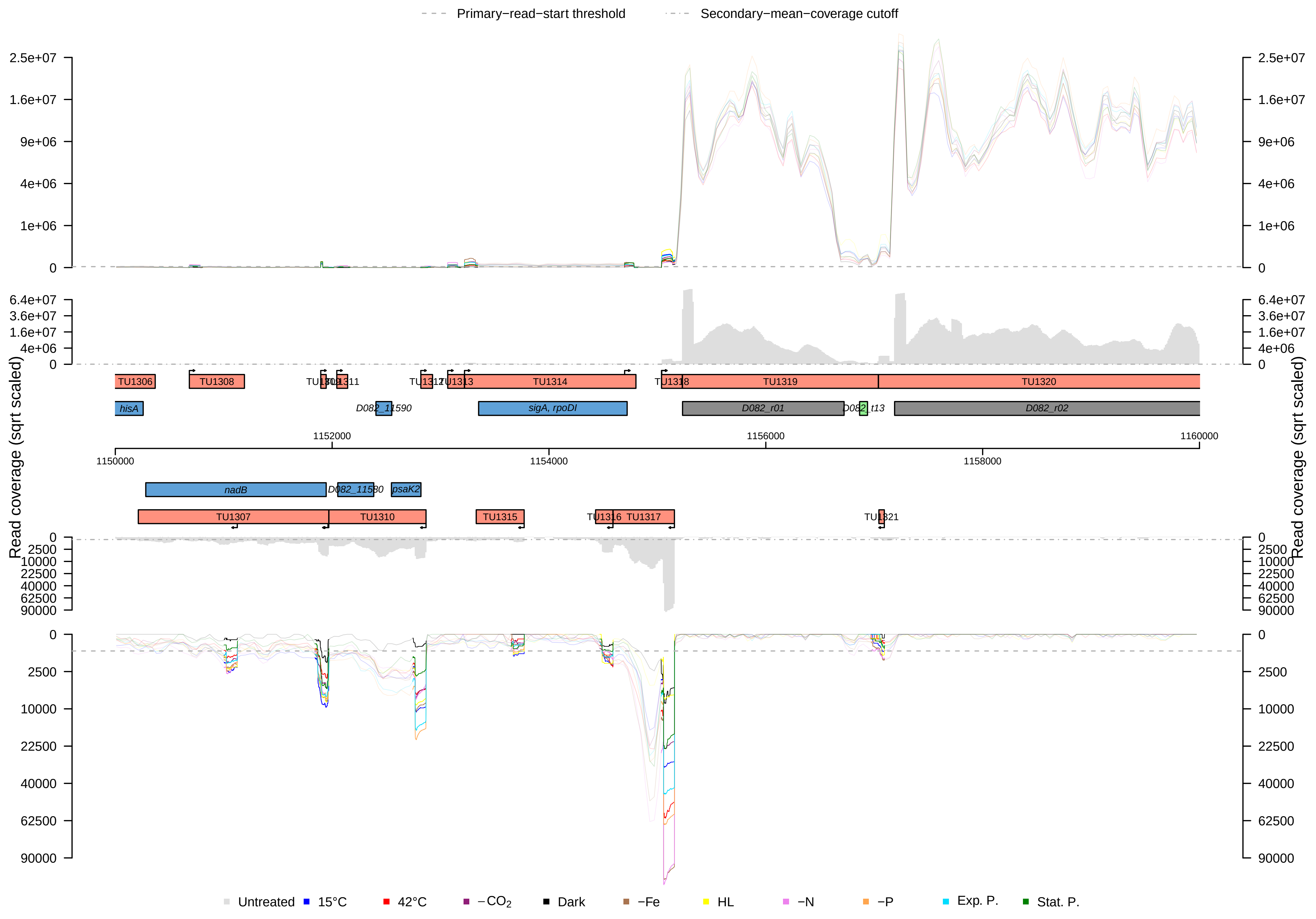

Primary−read−start threshold
Secondary−mean−coverage cutoff
Read coverage (sqrt scaled)
2.5e+07
1.6e+07
9e+06
4e+06
1e+06
0
6.4e+07
3.6e+07
1.6e+07
4e+06
TU1306
TU1308
TU1314
TU1319
TU1320
hisA
D082_11590
sigA, rpoDI
D082_r01
D082_t13
D082_r02
1150000
1152000
1154000
1156000
1158000
1160000
nadB
D082_11580
psaK2
TU1307
TU1310
TU1315
TU1316
TU1317
2500
10000
22500
40000
62500
90000
Untreated
15°C
42°C
−CO2
Dark
−Fe
HL
−N
−P
Exp. P.
Stat. P.

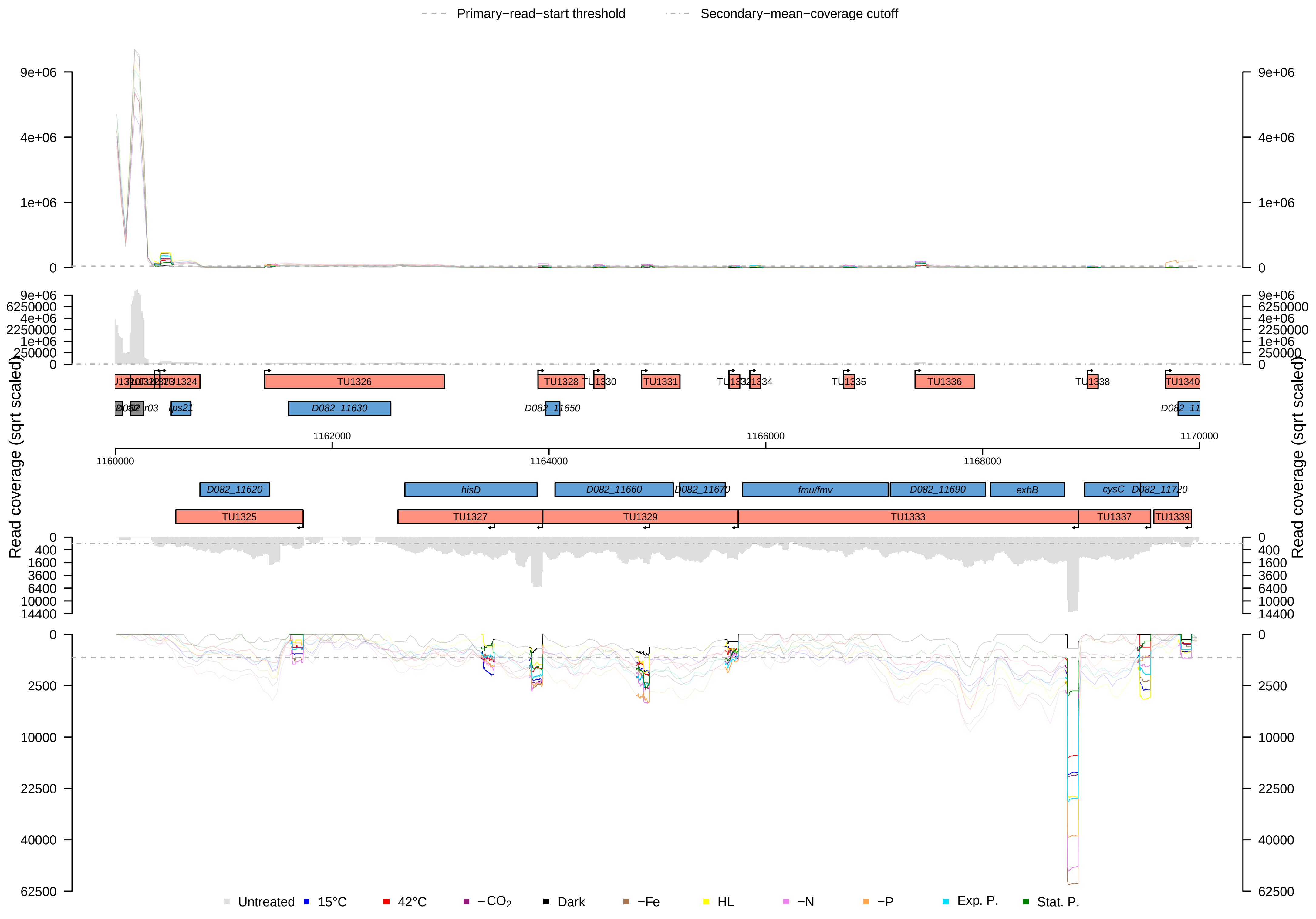

Primary−read−start threshold
Secondary−mean−coverage cutoff
Read coverage (sqrt scaled)
TU1325
TU1326
TU1327
TU1328
TU1329
TU1330
TU1331
TU1333
TU1336
TU1337
TU1339
TU1340
D082_11620
D082_11630
hisD
D082_11660
fmu/fmv
D082_11690
exbB
cysC
rps21
1160000
1162000
1164000
1166000
1168000
1170000
Untreated
15°C
42°C
−CO_2
Dark
−Fe
HL
−N
−P
Exp. P.
Stat. P.

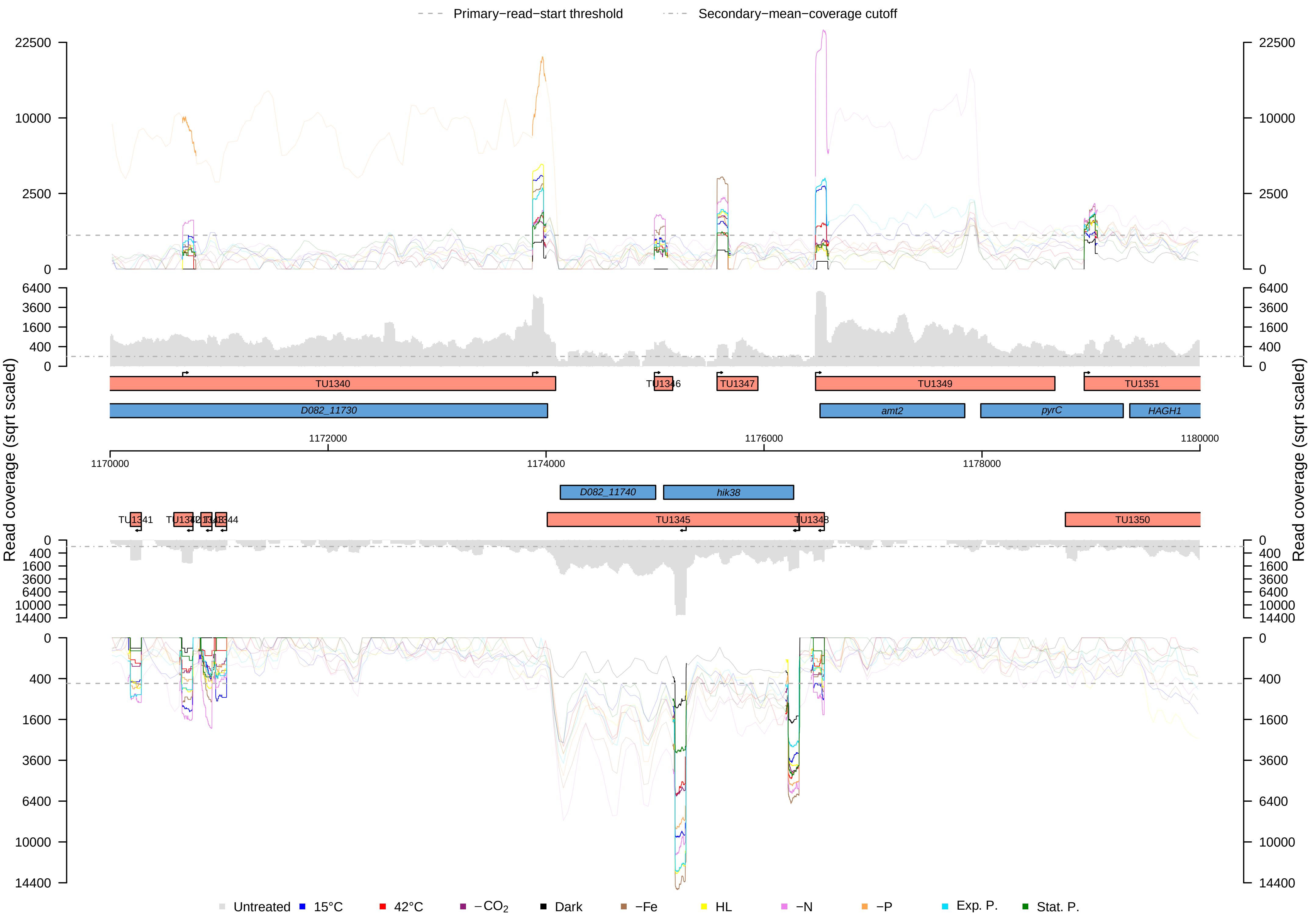

Primary−read−start threshold
Secondary−mean−coverage cutoff
Read coverage (sqrt scaled)
22500
10000
2500
0
6400
3600
1600
400
TU1340
TU1346
TU1347
TU1349
TU1351
D082_11730
amt2
pyrC
HAGH1
1170000
1172000
1174000
1176000
1178000
1180000
D082_11740
hik38
TU1341
TU1345
TU1348
TU1350
14400
Untreated
15°C
42°C
−CO2
Dark
−Fe
HL
−N
−P
Exp. P.
Stat. P.

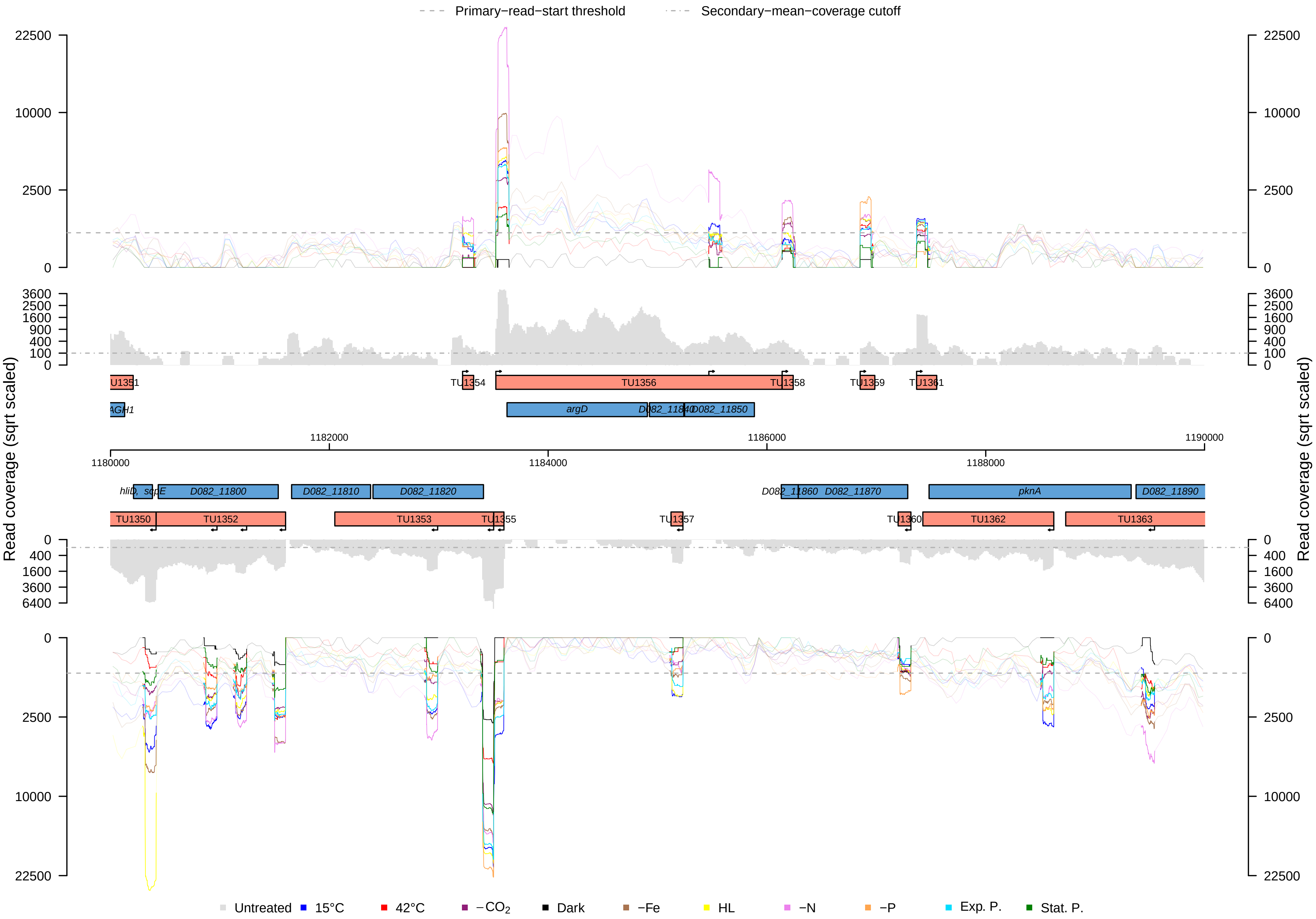

Primary−read−start threshold
Secondary−mean−coverage cutoff
Read coverage (sqrt scaled)
22500
10000
2500
0
3600
2500
1600
900
400
100
6400
U1351
TU1354
TU1356
TU1358
TU1359
TU1361
AGH1
argD
D082_11840
D082_11850
1182000
1186000
1190000
1180000
1184000
1188000
hliD
scpE
D082_11800
D082_11810
D082_11820
D082_11860
D082_11870
pknA
D082_11890
TU1350
TU1352
TU1353
TU1355
TU1357
TU1360
TU1362
TU1363
Untreated
15°C
42°C
$-CO_2$
Dark
−Fe
HL
−N
−P
Exp. P.
Stat. P.

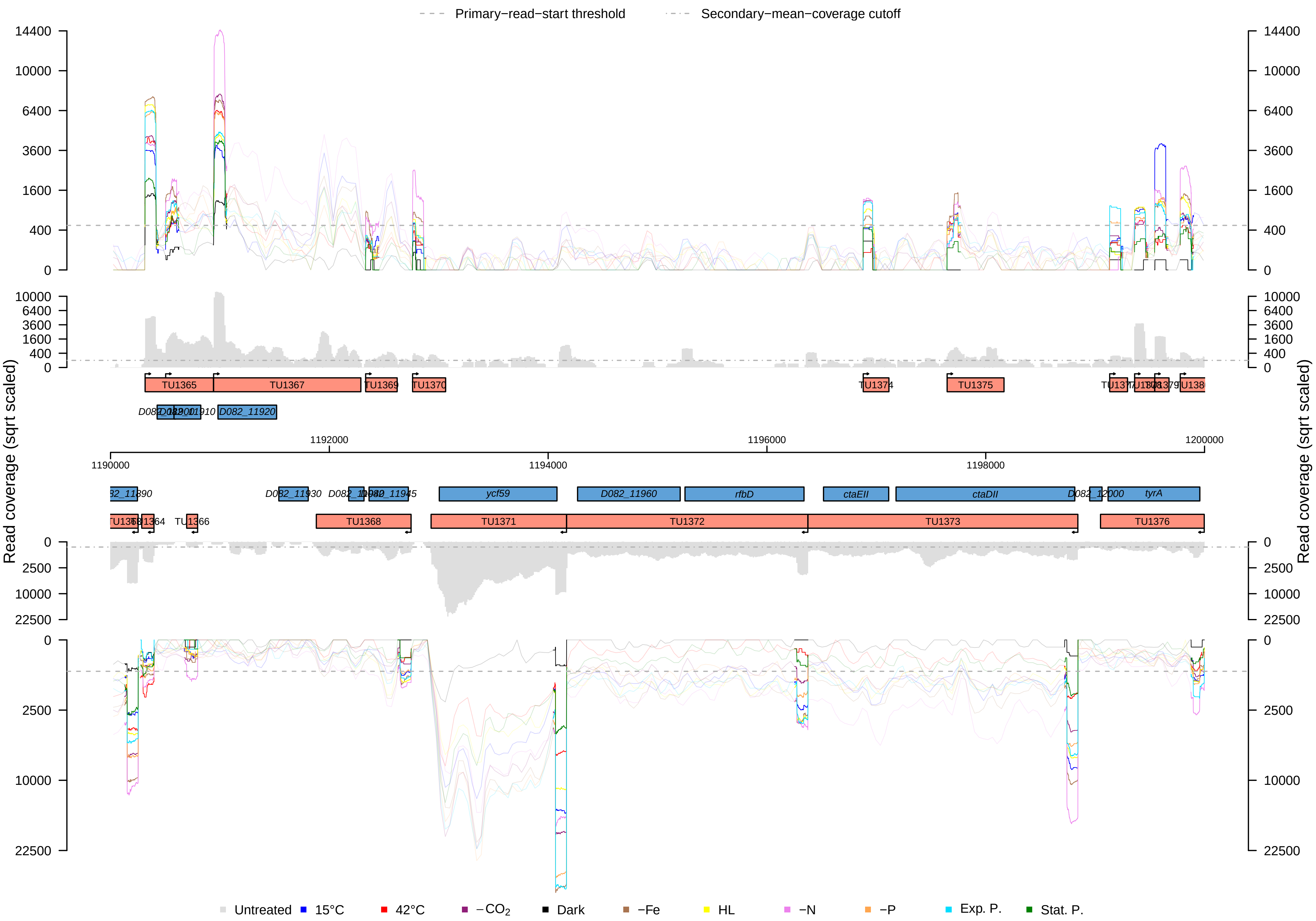

Primary−read−start threshold
Secondary−mean−coverage cutoff
Read coverage (sqrt scaled)
TU1365
TU1367
TU1369
TU1370
TU1374
TU1375
D082_11920
1190000
1192000
1194000
1196000
1198000
1200000
ycf59
D082_11960
rfbD
ctaEII
ctaDII
tyrA
D082_11930
TU1368
TU1371
TU1372
TU1373
TU1376
Untreated
15°C
42°C
−CO2
Dark
−Fe
HL
−N
−P
Exp. P.
Stat. P.

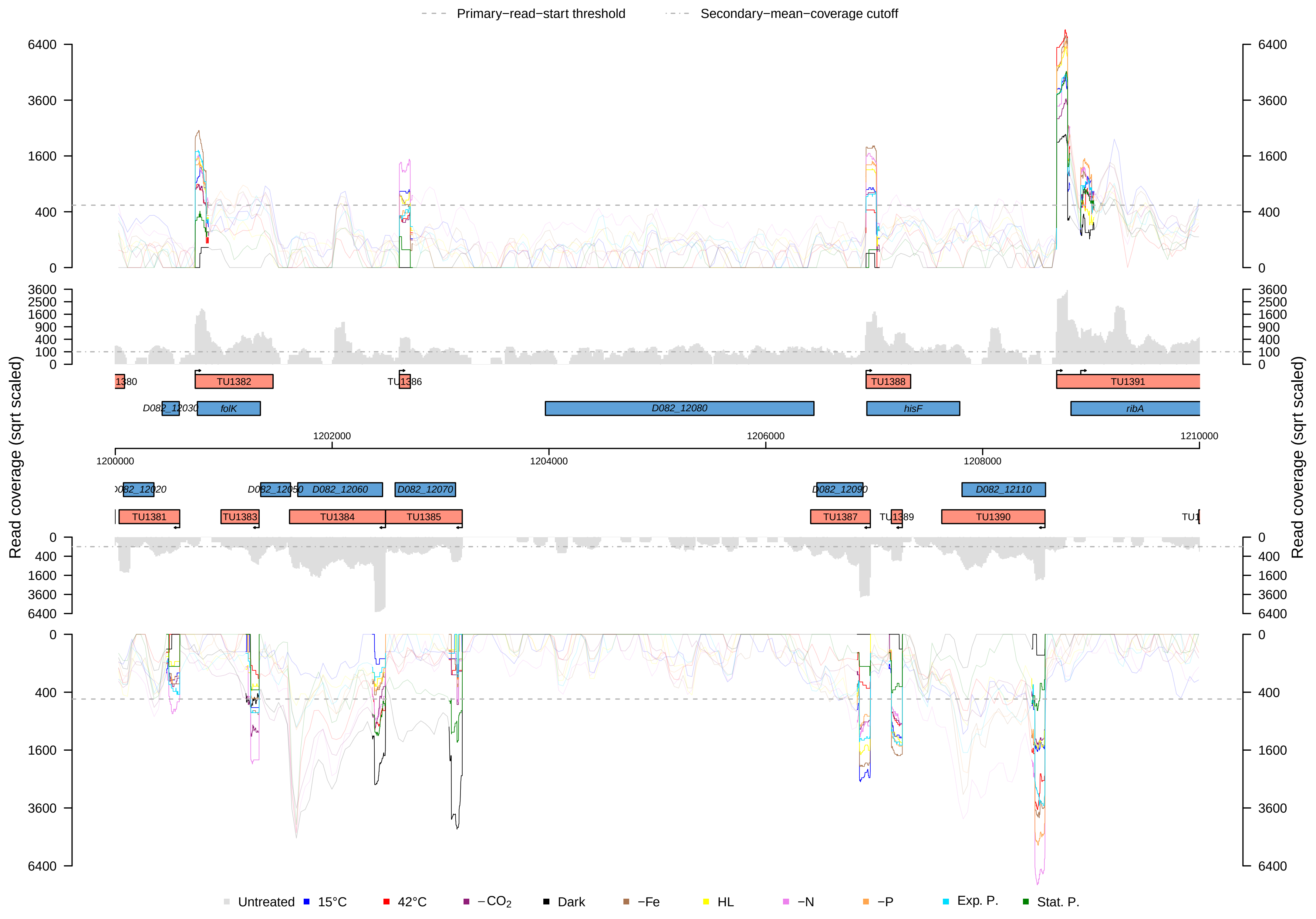

Primary−read−start threshold
Secondary−mean−coverage cutoff
Read coverage (sqrt scaled)
TU1382
TU1386
TU1388
TU1391
folK
D082_12030
D082_12080
hisF
ribA
1200000
1202000
1204000
1206000
1208000
1210000
D082_12050
D082_12060
D082_12070
D082_12090
D082_12110
TU1381
TU1383
TU1384
TU1385
TU1387
TU1390
Untreated
15°C
42°C
$-CO_2$
Dark
−Fe
HL
−N
−P
Exp. P.
Stat. P.

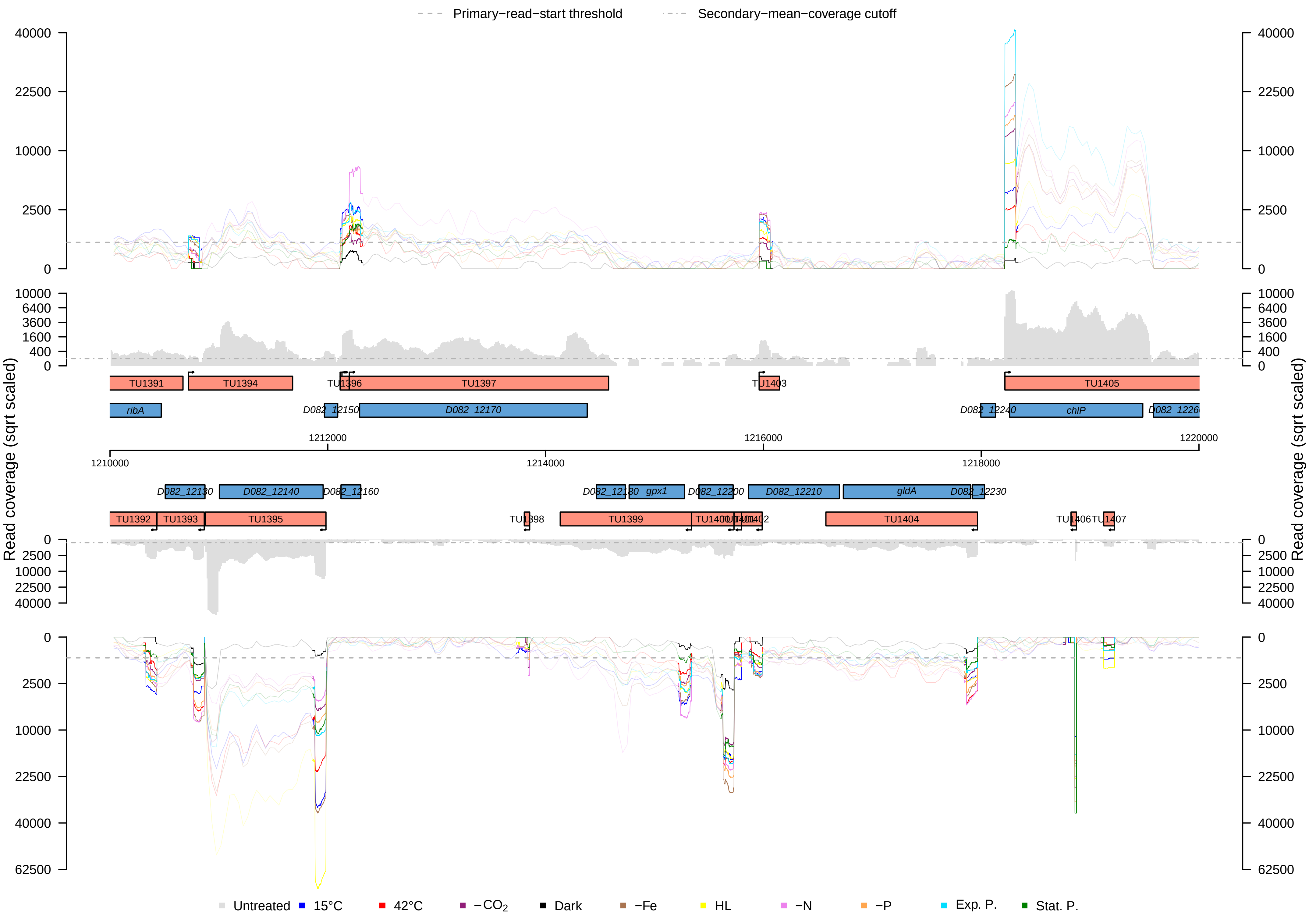

Primary−read−start threshold
Secondary−mean−coverage cutoff
Read coverage (sqrt scaled)
Read coverage (sqrt scaled)
40000
22500
10000
2500
0
10000
6400
3600
1600
400
0
TU1391
TU1394
TU1396
TU1397
TU1403
TU1405
ribA
D082_12150
D082_12170
D082_12240
chlP
D082_1226
1210000
1212000
1214000
1216000
1218000
1220000
D082_12130
D082_12140
D082_12160
D082_12180
gpx1
D082_12200
D082_12210
gldA
D082_12230
TU1392
TU1393
TU1395
TU1398
TU1399
TU1402
TU1404
TU1406
TU1407
62500
Untreated
15°C
42°C
−CO2
Dark
−Fe
HL
−N
−P
Exp. P.
Stat. P.

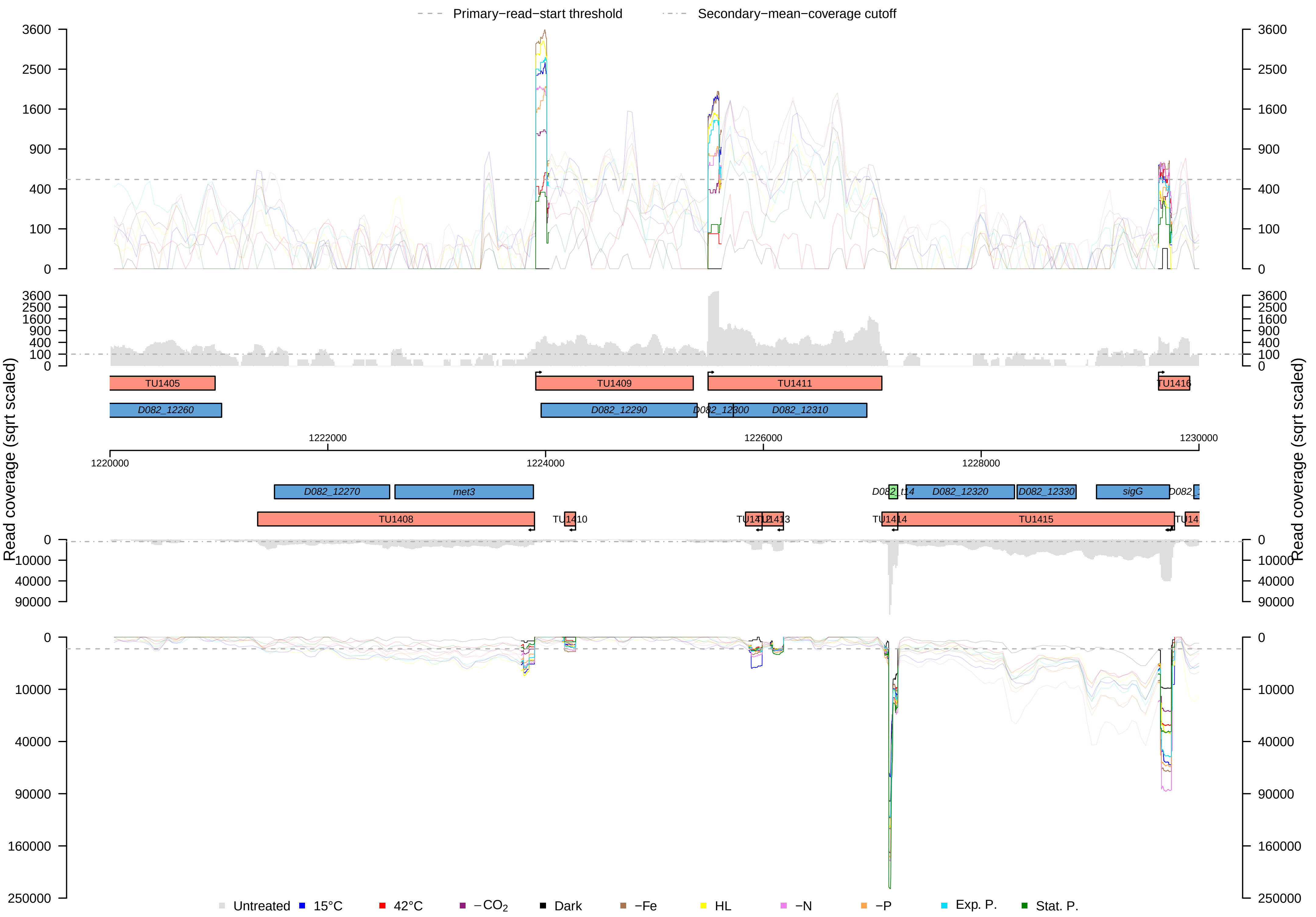

Primary−read−start threshold
Secondary−mean−coverage cutoff
Read coverage (sqrt scaled)
3600
2500
1600
900
400
100
0
TU1405
TU1409
TU1411
TU1416
D082_12260
D082_12290
D082_12300
D082_12310
1220000
1222000
1224000
1226000
1228000
1230000
D082_12270
met3
D082_12320
D082_12330
sigG
TU1408
TU1410
TU1413
TU1414
TU1415
10000
40000
90000
160000
250000
Untreated
15°C
42°C
$-CO_2$
Dark
−Fe
HL
−N
−P
Exp. P.
Stat. P.

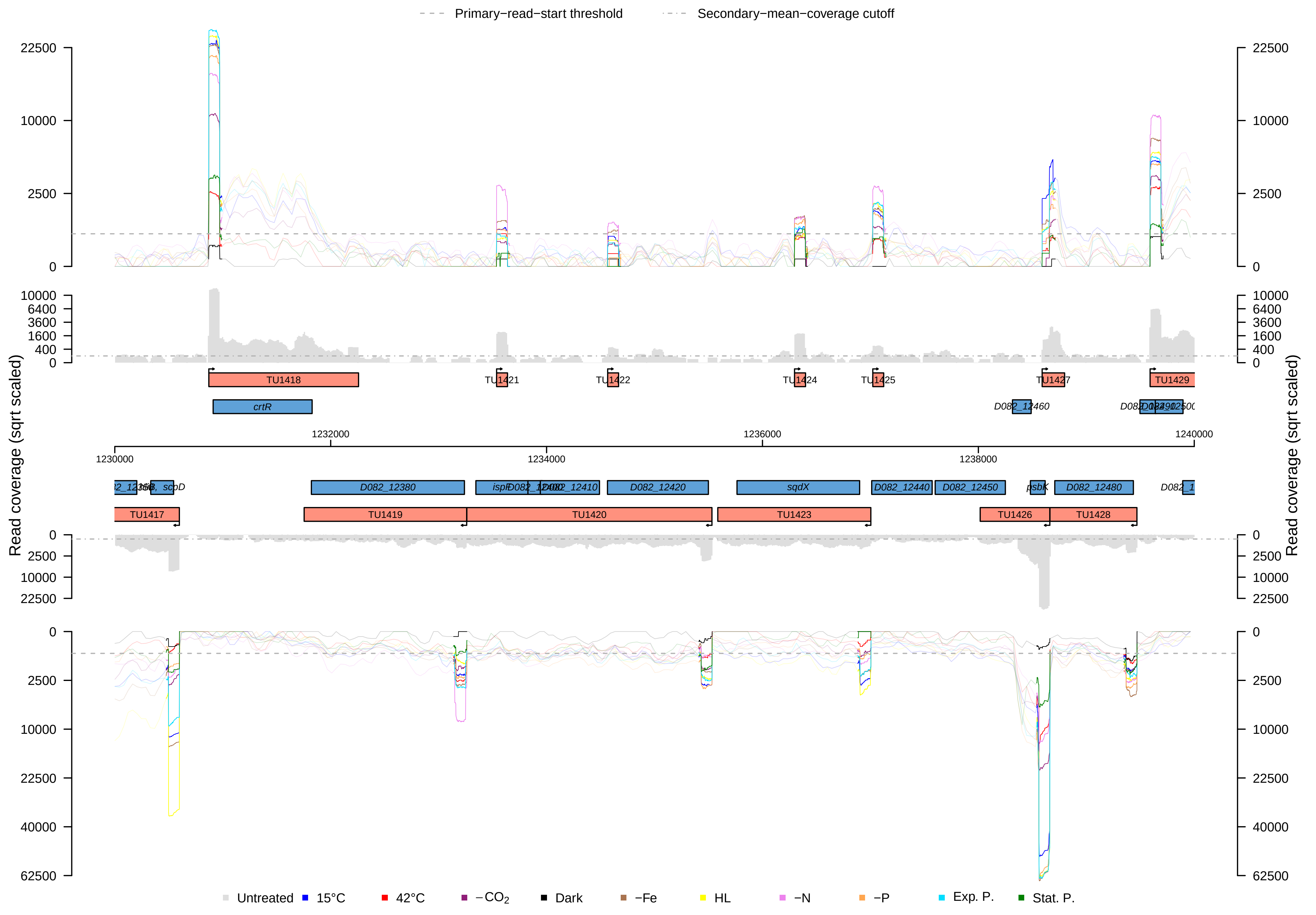

Primary−read−start threshold
Secondary−mean−coverage cutoff
Read coverage (sqrt scaled)
0
2500
10000
22500
400
1600
3600
6400
40000
62500
TU1417
TU1418
TU1419
TU1420
TU1421
TU1422
TU1423
TU1424
TU1425
TU1426
TU1427
TU1428
TU1429
crtR
scoD
D082_12380
D082_12410
D082_12420
sqdX
D082_12440
D082_12450
D082_12460
D082_12480
1230000
1232000
1234000
1236000
1238000
1240000
Untreated
15°C
42°C
−CO2
Dark
−Fe
HL
−N
−P
Exp. P.
Stat. P.

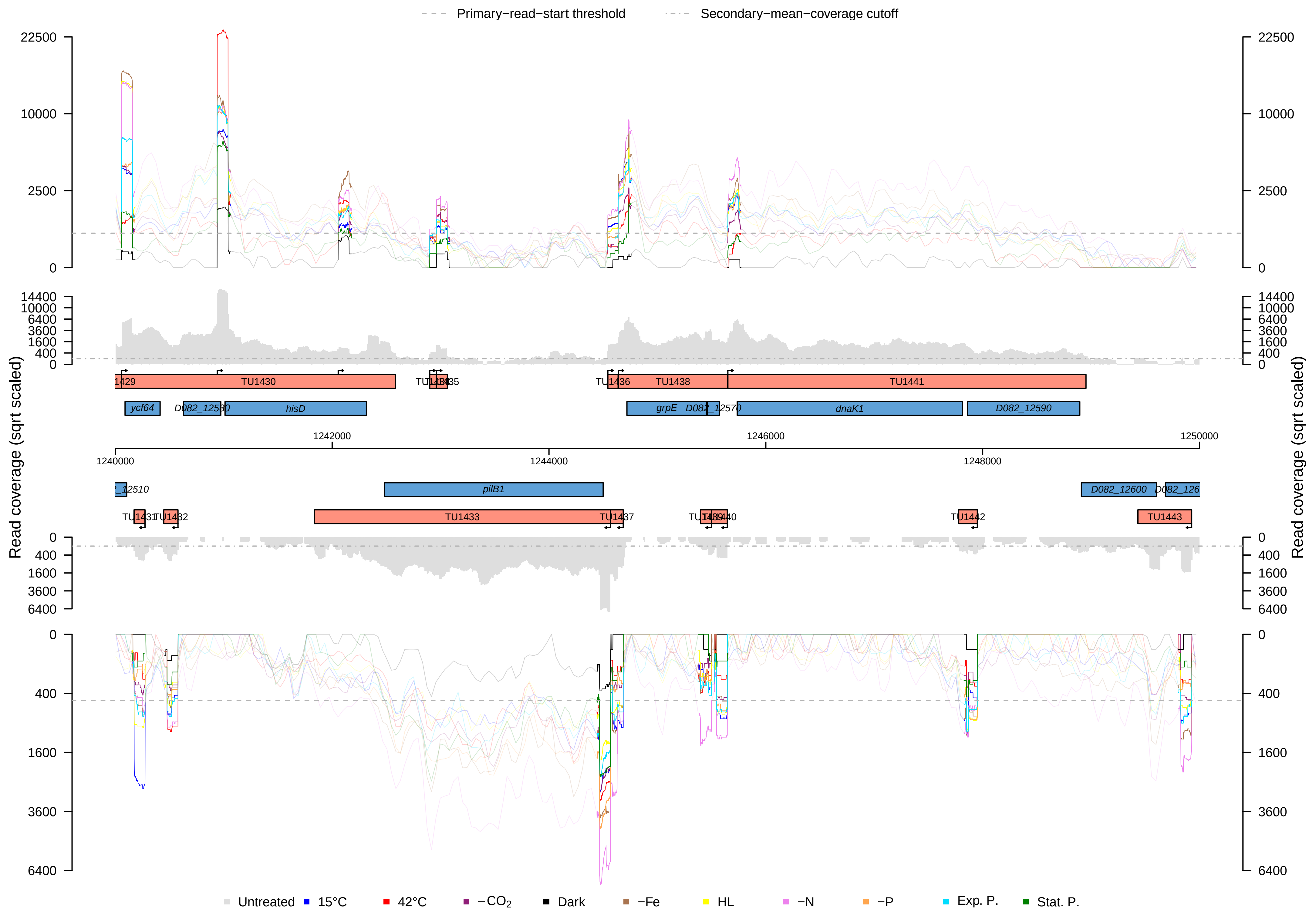

Primary−read−start threshold
Secondary−mean−coverage cutoff
Read coverage (sqrt scaled)
22500
10000
2500
0
14400
10000
6400
3600
1600
400
0
1429
TU1430
TU1434
TU1435
TU1436
TU1438
TU1441
ycf64
D082_12530
hisD
grpE
D082_12570
dnaK1
D082_12590
1240000
1242000
1244000
1246000
1248000
1250000
_12510
pilB1
D082_12600
D082_126
TU1431
TU1432
TU1433
TU1437
TU1439
TU1440
TU1442
TU1443
6400
3600
1600
400
0
Untreated
15°C
42°C
−CO2
Dark
−Fe
HL
−N
−P
Exp. P.
Stat. P.

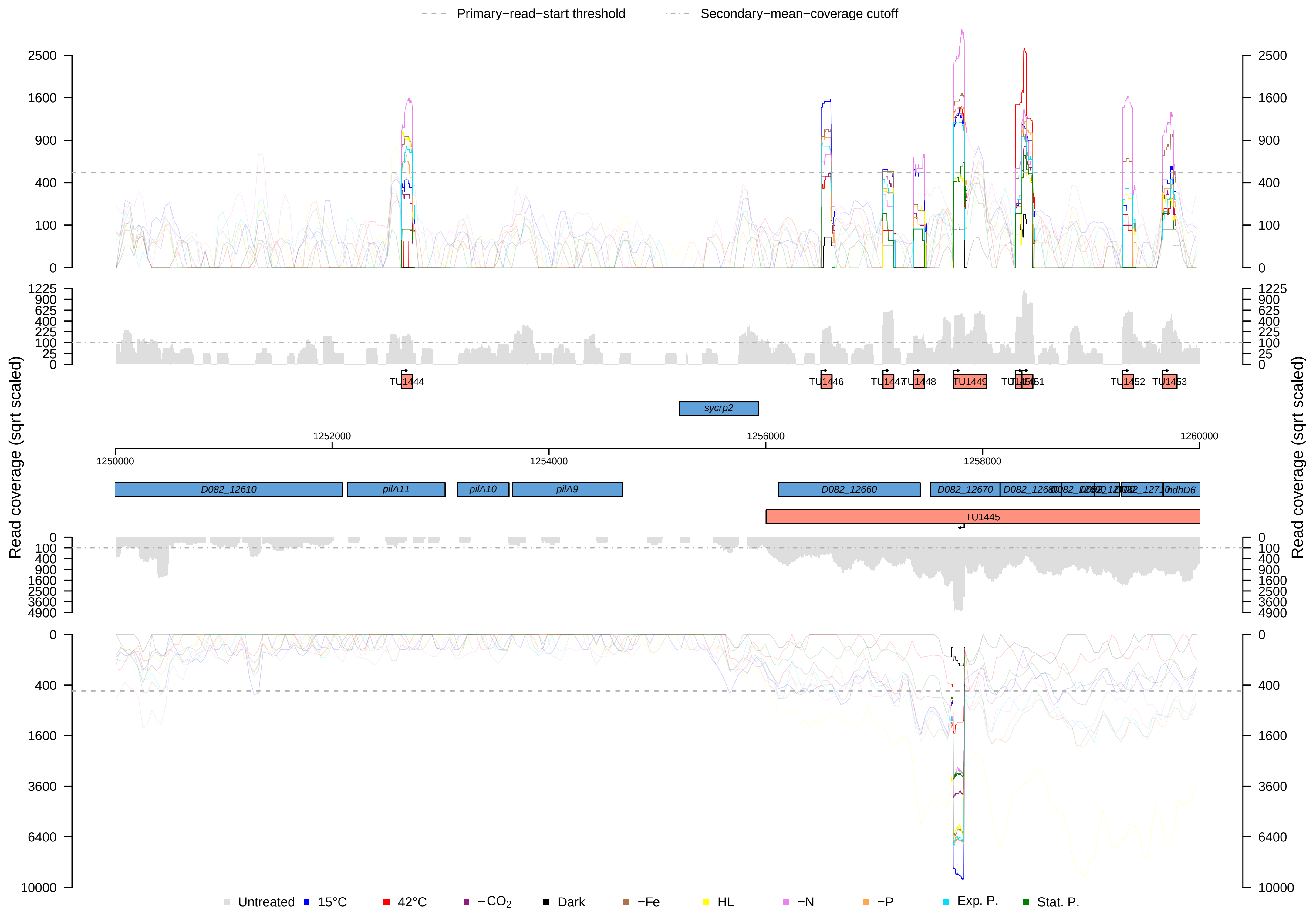

Primary−read−start threshold
Secondary−mean−coverage cutoff
Read coverage (sqrt scaled)
TU1444
TU1446
TU1447
TU1448
TU1449
TU1452
TU1453
sycrp2
1250000
1252000
1254000
1256000
1258000
1260000
D082_12610
pilA11
pilA10
pilA9
D082_12660
D082_12670
ndhD6
TU1445
Untreated
15°C
42°C
−CO2
Dark
−Fe
HL
−N
−P
Exp. P.
Stat. P.

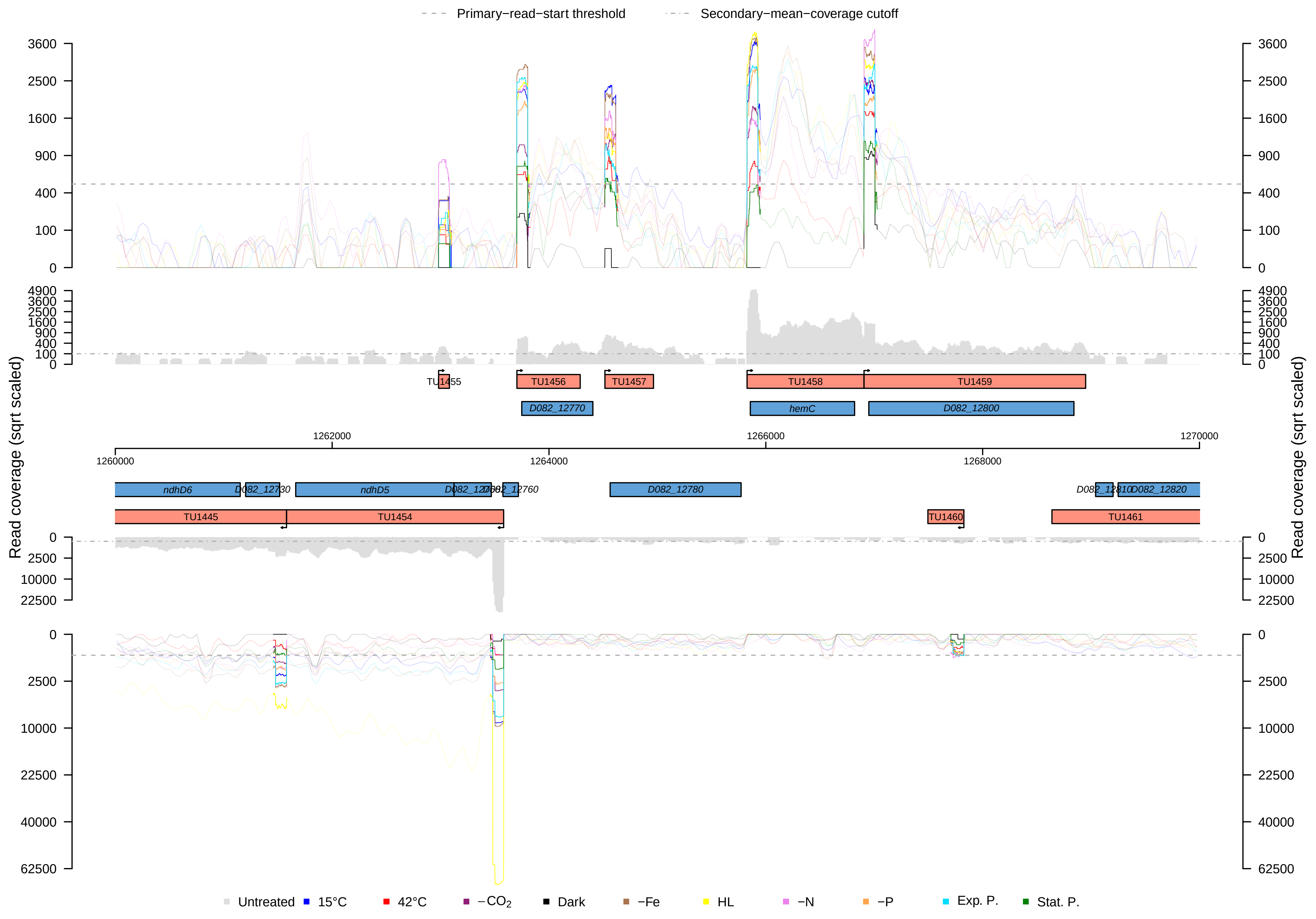

Primary−read−start threshold
Secondary−mean−coverage cutoff
Read coverage (sqrt scaled)
3600
2500
1600
900
400
100
0
4900
TU1455
TU1456
TU1457
TU1458
TU1459
D082_12770
hemC
D082_12800
1260000
1262000
1264000
1266000
1268000
1270000
ndhD6
D082_12730
ndhD5
D082_12780
D082_12810
D082_12820
TU1445
TU1454
TU1460
TU1461
10000
22500
40000
62500
Untreated
15°C
42°C
−CO2
Dark
−Fe
HL
−N
−P
Exp. P.
Stat. P.

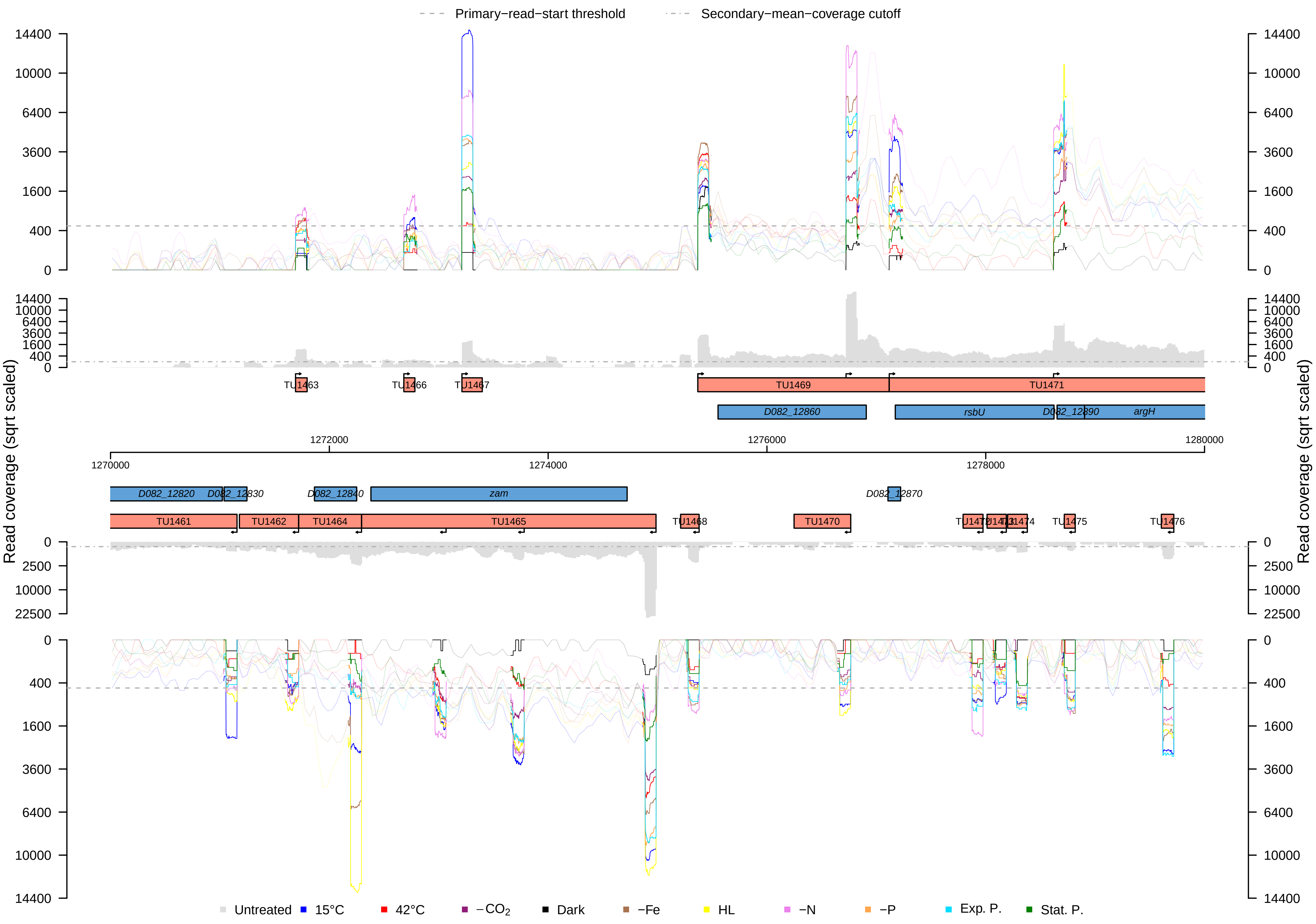

Primary−read−start threshold
Secondary−mean−coverage cutoff
Read coverage (sqrt scaled)
14400
10000
6400
3600
1600
400
0
2500
22500
TU1463
TU1466
TU1467
TU1469
TU1471
D082_12860
rsbU
D082_12890
argH
1270000
1272000
1274000
1276000
1278000
1280000
D082_12820
D082_12830
D082_12840
zam
D082_12870
TU1461
TU1462
TU1464
TU1465
TU1468
TU1470
TU1472
TU1473
TU1474
TU1475
TU1476
Untreated
15°C
42°C
−CO_2
Dark
−Fe
HL
−N
−P
Exp. P.
Stat. P.

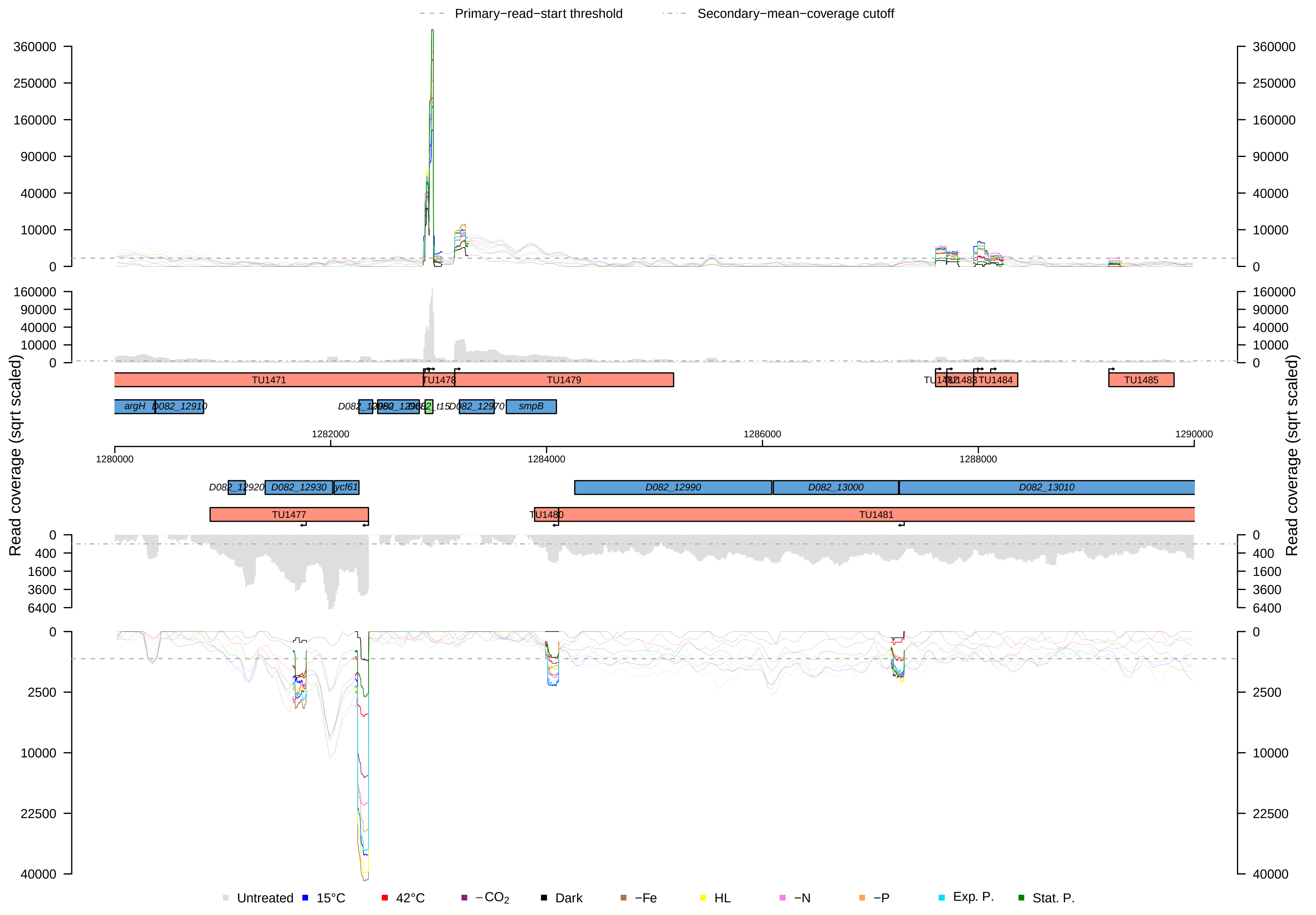

Primary−read−start threshold
Secondary−mean−coverage cutoff
Read coverage (sqrt scaled)
360000
250000
160000
90000
40000
10000
0
TU1471
TU1478
TU1479
TU1484
TU1485
argH
D082_12910
smpB
D082_12970
1280000
1282000
1284000
1286000
1288000
1290000
D082_12920
D082_12930
ycf61
D082_12990
D082_13000
D082_13010
TU1477
TU1480
TU1481
400
1600
3600
6400
2500
10000
22500
40000
Untreated
15°C
42°C
−CO2
Dark
−Fe
HL
−N
−P
Exp. P.
Stat. P.

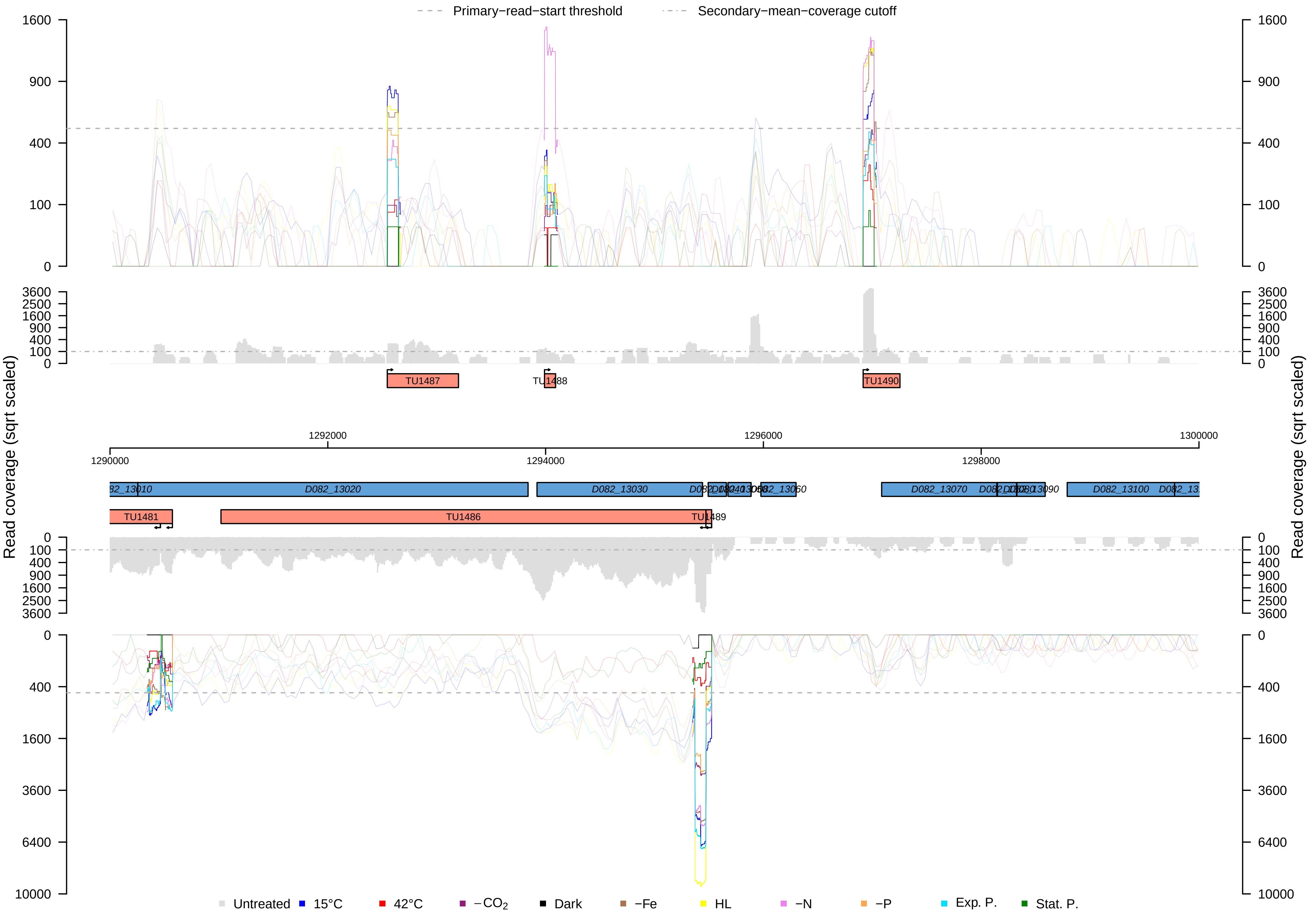

Primary−read−start threshold
Secondary−mean−coverage cutoff
Read coverage (sqrt scaled)
TU1487
TU1488
TU1490
1290000
1292000
1294000
1296000
1298000
1300000
D082_13020
D082_13030
D082_13060
D082_13070
D082_13100
TU1481
TU1486
TU1489
Untreated
15°C
42°C
−CO2
Dark
−Fe
HL
−N
−P
Exp. P.
Stat. P.

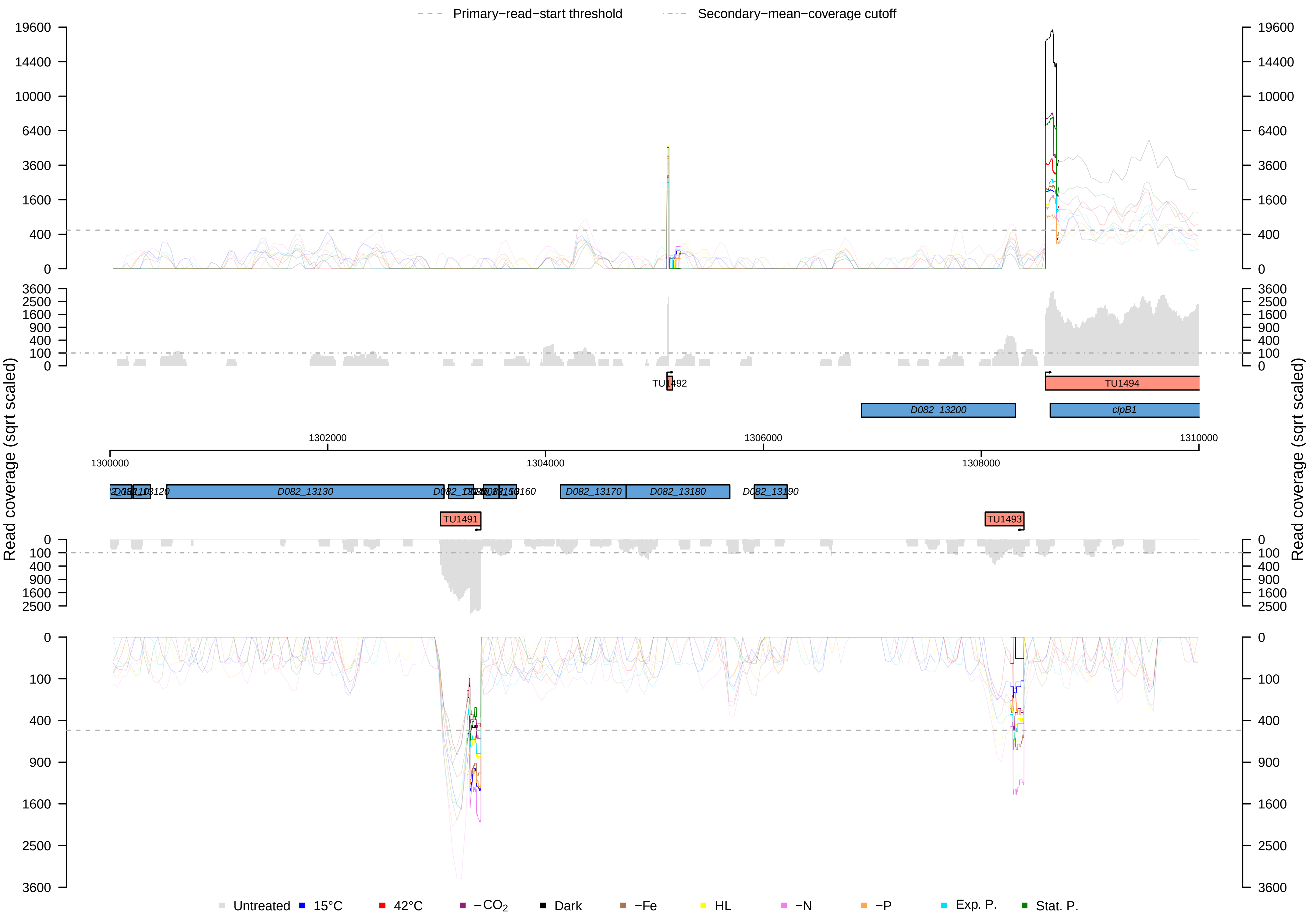

Primary−read−start threshold
Secondary−mean−coverage cutoff
Read coverage (sqrt scaled)
TU1492
TU1494
D082_13200
clpB1
1300000
1302000
1304000
1306000
1308000
1310000
D082_13130
D082_13170
D082_13180
D082_13190
TU1491
TU1493
Untreated
15°C
42°C
−CO2
Dark
−Fe
HL
−N
−P
Exp. P.
Stat. P.

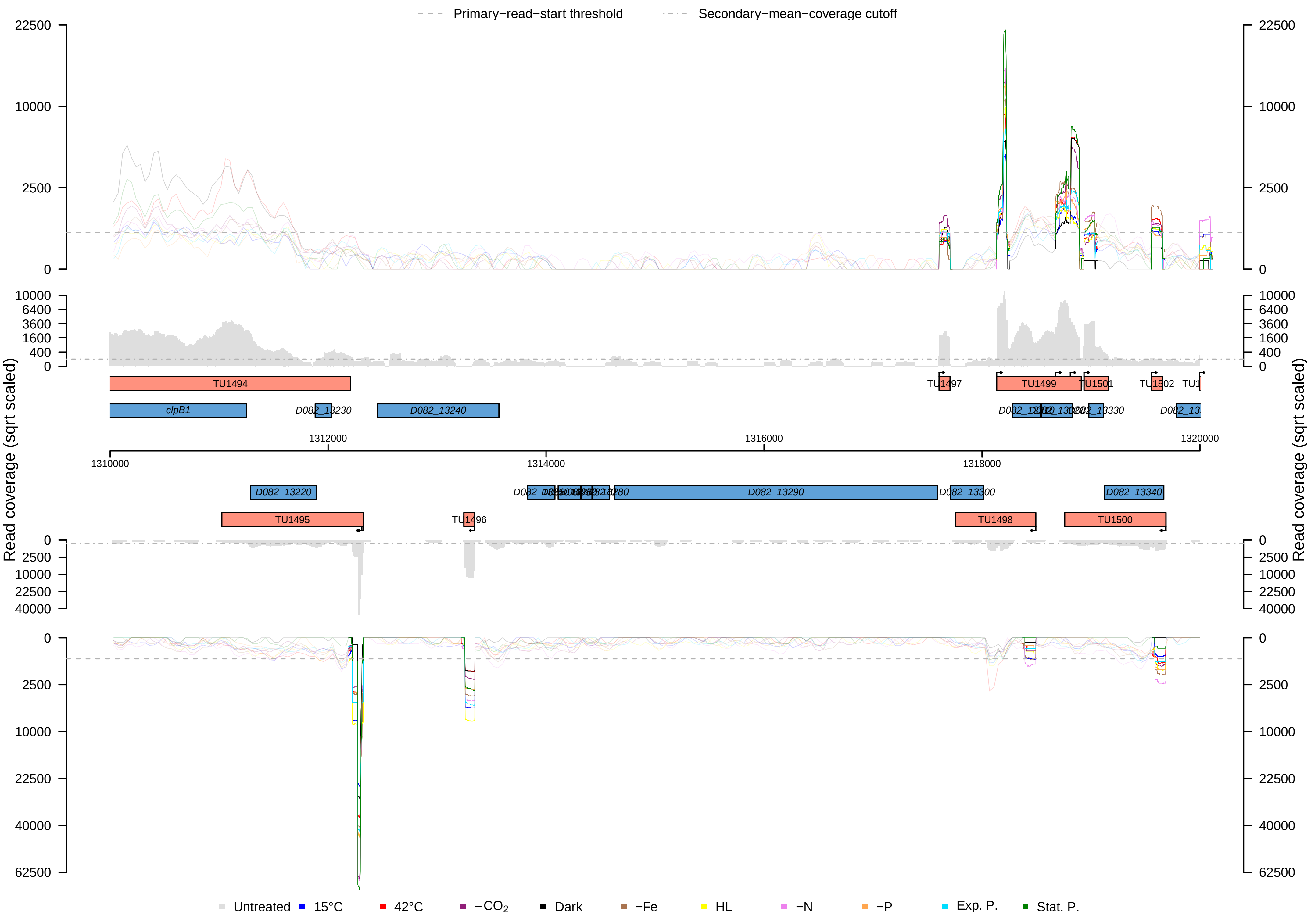

Primary−read−start threshold
Secondary−mean−coverage cutoff
Read coverage (sqrt scaled)
TU1494
clpB1
D082_13230
D082_13240
TU1497
TU1499
TU1501
TU1502
D082_13330
D082_13220
D082_13290
D082_13300
D082_13340
TU1495
TU1496
TU1498
TU1500
1310000
1312000
1314000
1316000
1318000
1320000
Untreated
15°C
42°C
−CO2
Dark
−Fe
HL
−N
−P
Exp. P.
Stat. P.

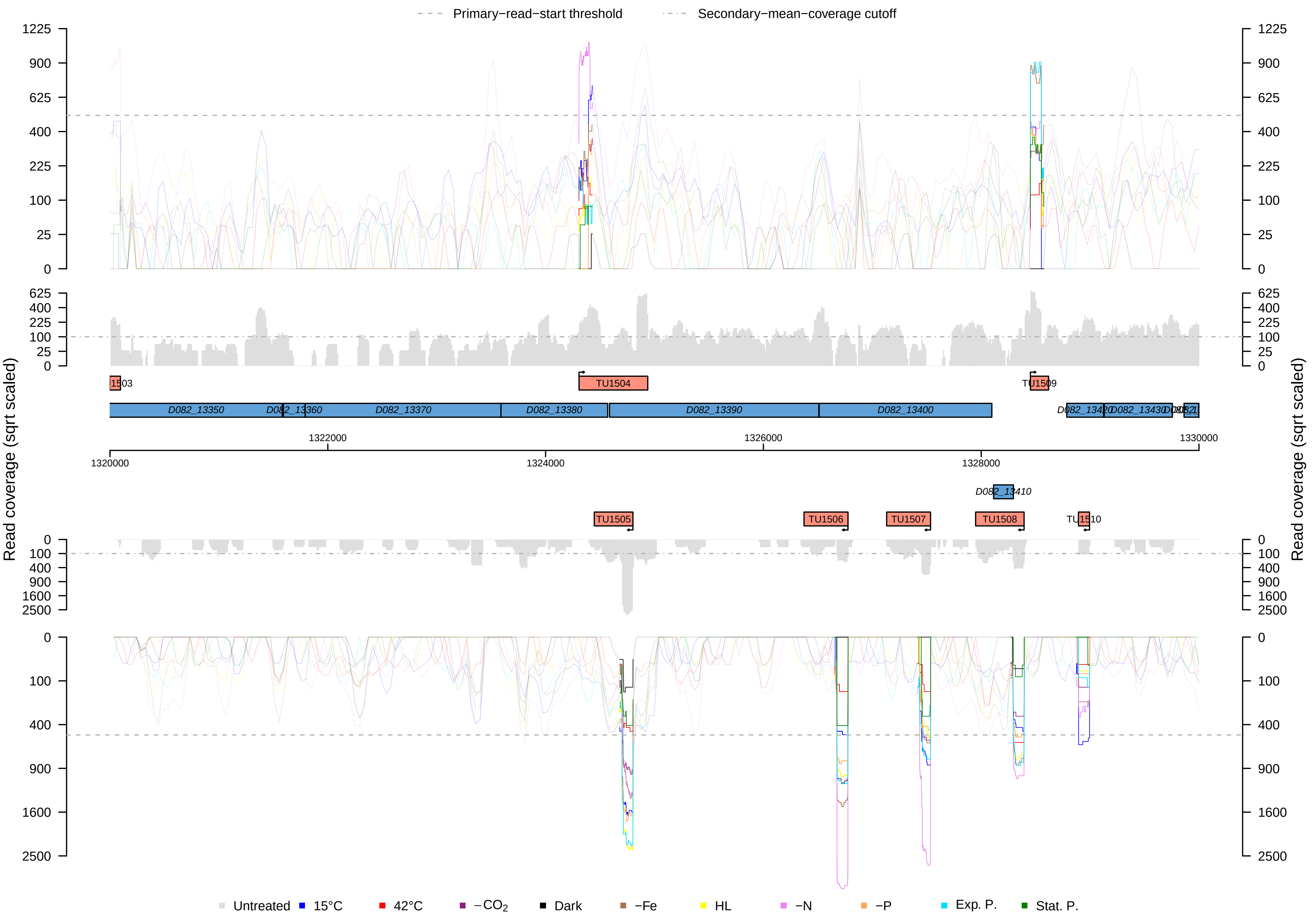

Primary−read−start threshold
Secondary−mean−coverage cutoff
Read coverage (sqrt scaled)
TU1504
TU1509
D082_13350
D082_13360
D082_13370
D082_13380
D082_13390
D082_13400
1320000
1322000
1324000
1326000
1328000
1330000
D082_13410
TU1505
TU1506
TU1507
TU1508
Untreated
15°C
42°C
−CO2
Dark
−Fe
HL
−N
−P
Exp. P.
Stat. P.

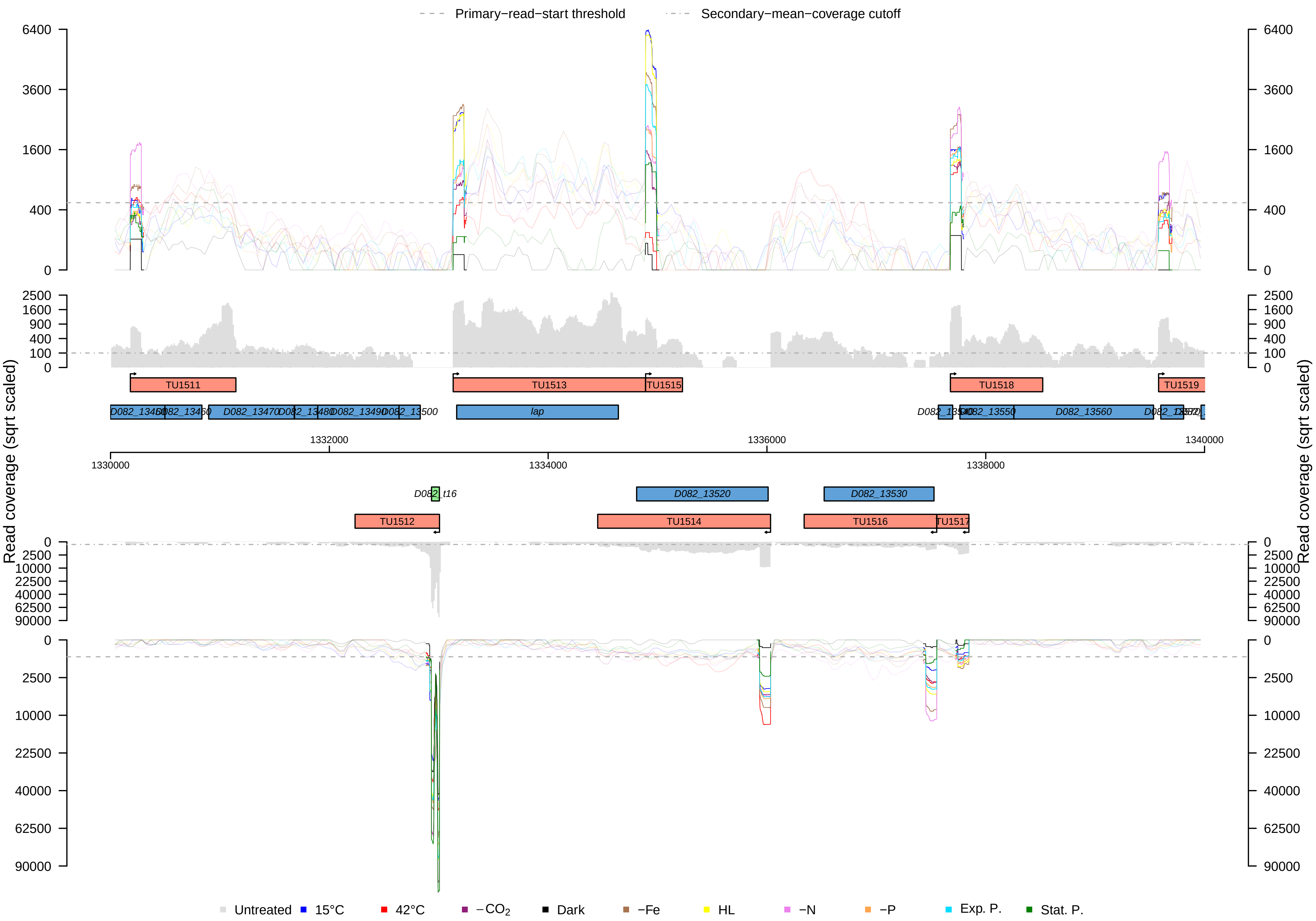

Primary−read−start threshold
Secondary−mean−coverage cutoff
Read coverage (sqrt scaled)
6400
3600
1600
400
0
2500
1600
900
400
100
0
TU1511
TU1513
TU1515
TU1518
TU1519
D082_13450
D082_13460
D082_13470
D082_13480
D082_13490
D082_13500
lap
D082_13550
D082_13560
1330000
1332000
1334000
1336000
1338000
1340000
D082_t16
D082_13520
D082_13530
TU1512
TU1514
TU1516
TU1517
10000
22500
40000
62500
90000
Untreated
15°C
42°C
−CO2
Dark
−Fe
HL
−N
−P
Exp. P.
Stat. P.

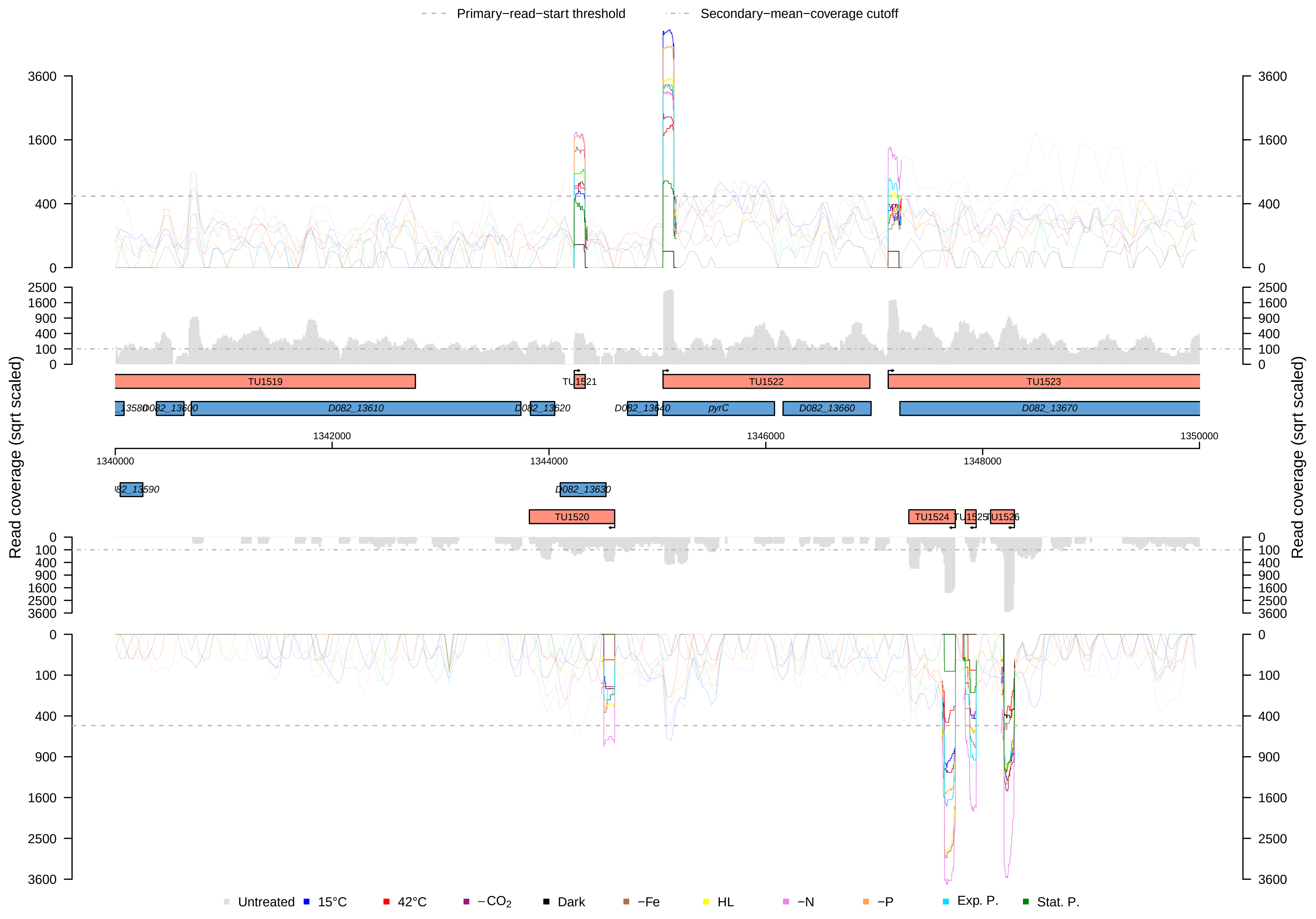

Primary−read−start threshold
Secondary−mean−coverage cutoff
Read coverage (sqrt scaled)
Read coverage (sqrt scaled)
3600
1600
400
0
2500
1600
900
400
100
0
TU1519
TU1521
TU1522
TU1523
D082_13580
D082_13600
D082_13610
D082_13620
D082_13640
pyrC
D082_13660
D082_13670
1342000
1346000
1350000
1340000
1344000
1348000
D082_13590
D082_13630
TU1520
TU1524
TU1525
TU1526
Untreated
15°C
42°C
−CO2
Dark
−Fe
HL
−N
−P
Exp. P.
Stat. P.

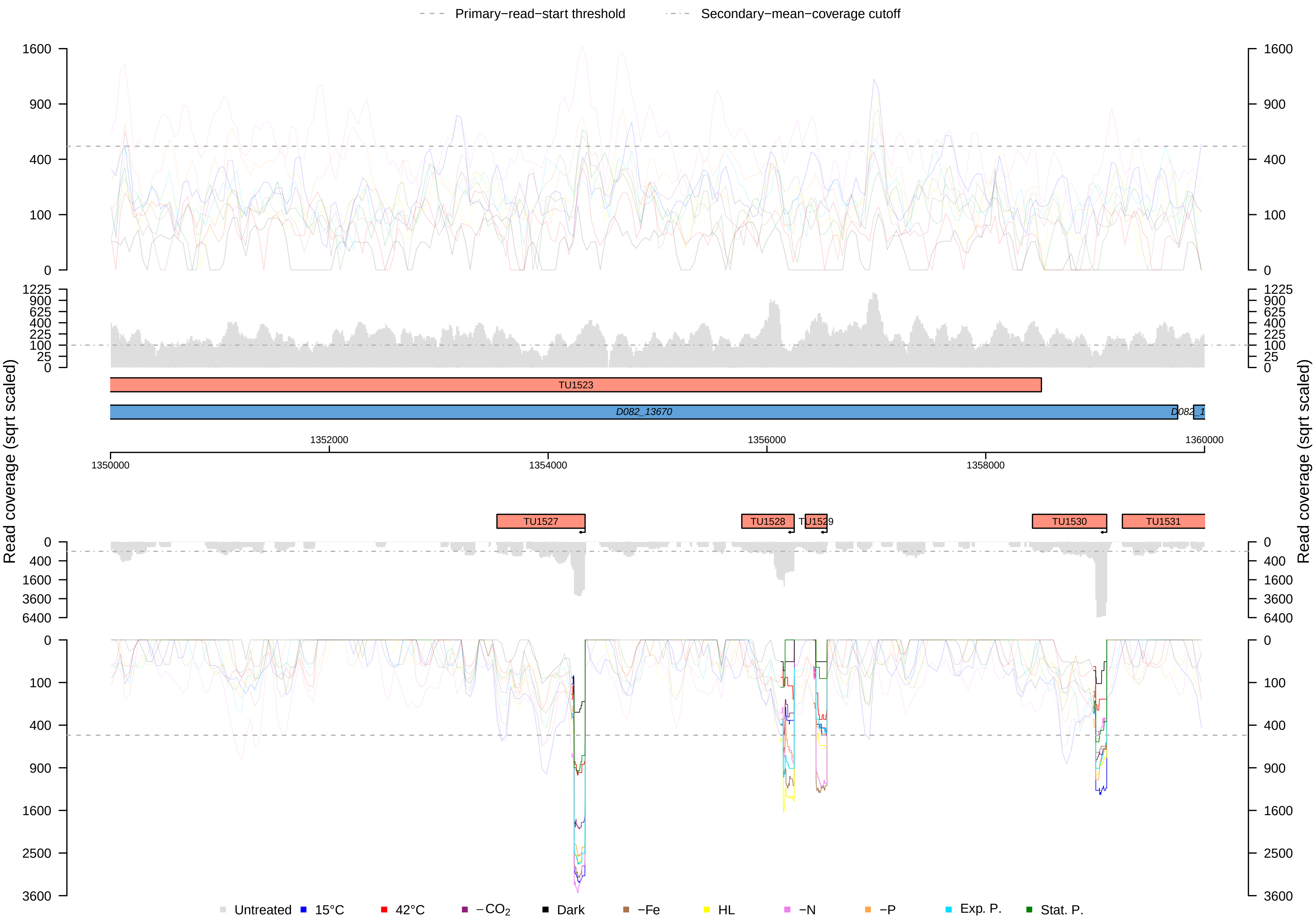

Primary−read−start threshold
Secondary−mean−coverage cutoff
Read coverage (sqrt scaled)
1600
900
400
100
0
1225
900
625
400
225
100
25
0
TU1523
D082_13670
D082_1
1352000
1356000
1360000
1350000
1354000
1358000
TU1527
TU1528
TU1529
TU1530
TU1531
0
400
1600
3600
6400
0
100
400
900
1600
2500
3600
Untreated
15°C
42°C
−CO2
Dark
−Fe
HL
−N
−P
Exp. P.
Stat. P.

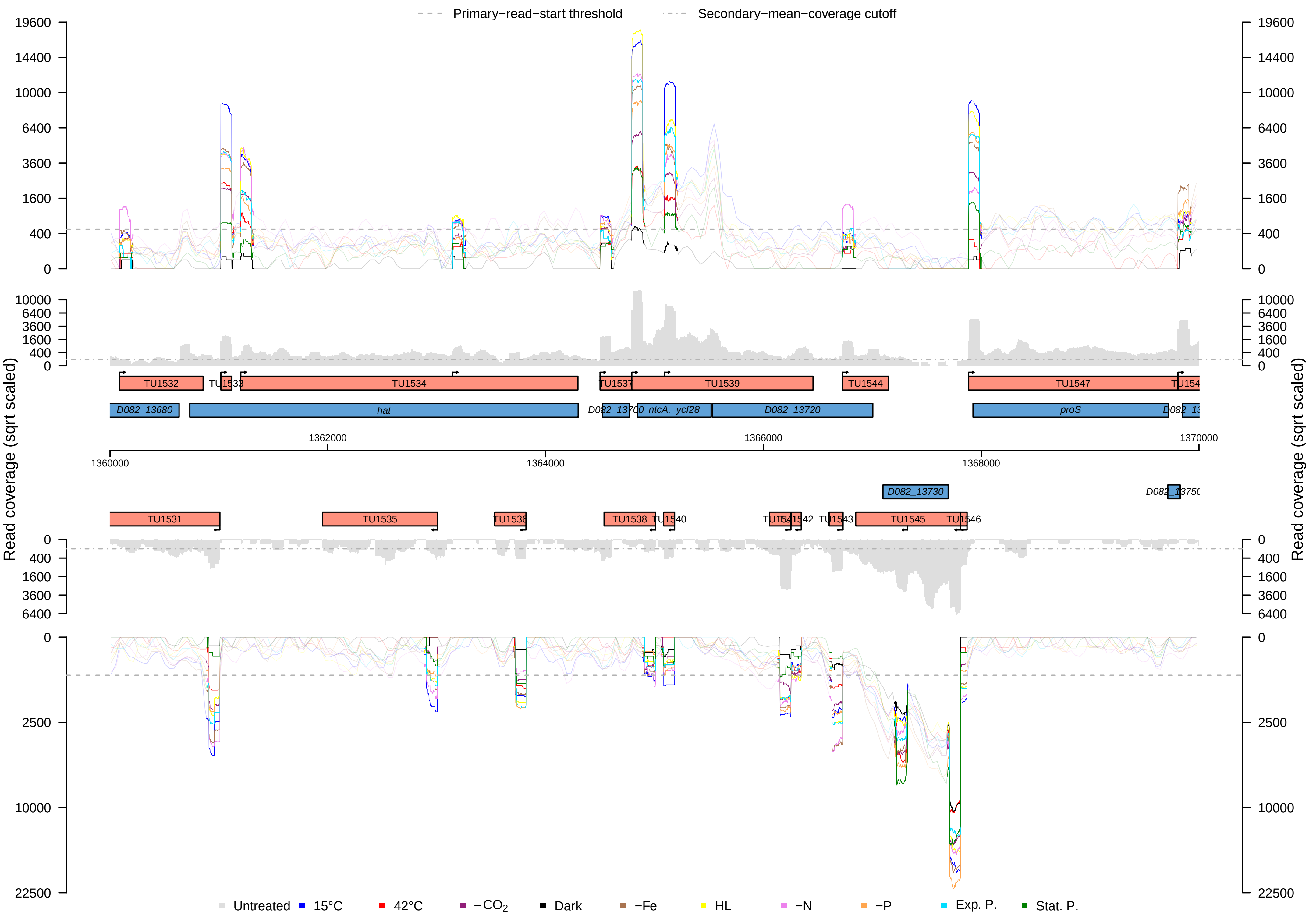

Primary−read−start threshold
Secondary−mean−coverage cutoff
Read coverage (sqrt scaled)
TU1532
TU1533
TU1534
TU1537
TU1539
TU1544
TU1547
D082_13680
hat
D082_13700
ntcA, ycf28
D082_13720
proS
1360000
1362000
1364000
1366000
1368000
1370000
D082_13730
TU1531
TU1535
TU1536
TU1538
TU1540
TU1542
TU1543
TU1545
TU1546
Untreated
15°C
42°C
−CO2
Dark
−Fe
HL
−N
−P
Exp. P.
Stat. P.

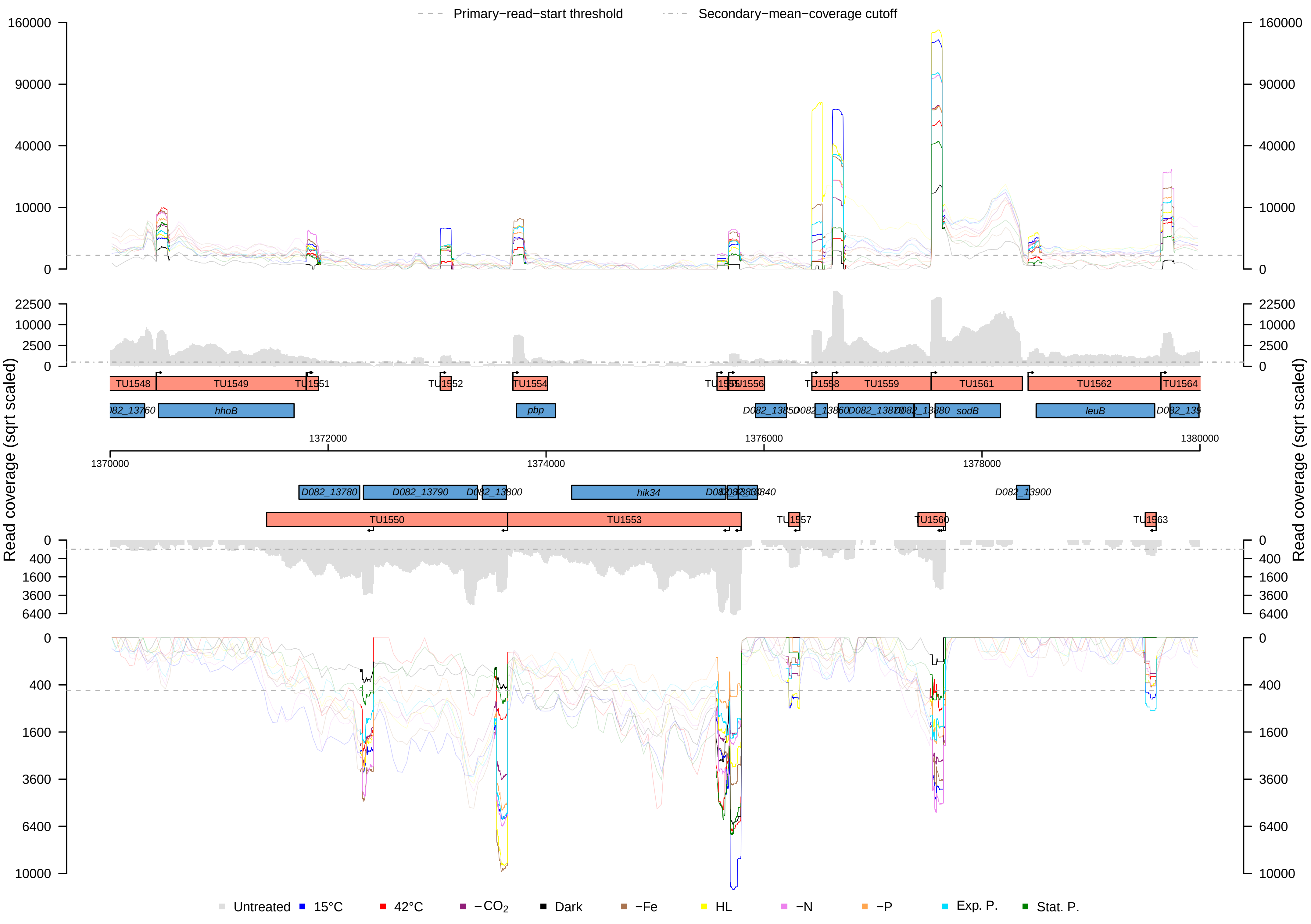

Primary−read−start threshold
Secondary−mean−coverage cutoff
Read coverage (sqrt scaled)
TU1548
TU1549
TU1551
TU1552
TU1554
TU1556
TU1559
TU1561
TU1562
TU1564
hhoB
pbp
sodB
leuB
D082_13780
D082_13790
hik34
TU1550
TU1553
TU1557
TU1560
TU1563
Untreated
15°C
42°C
−CO2
Dark
−Fe
HL
−N
−P
Exp. P.
Stat. P.

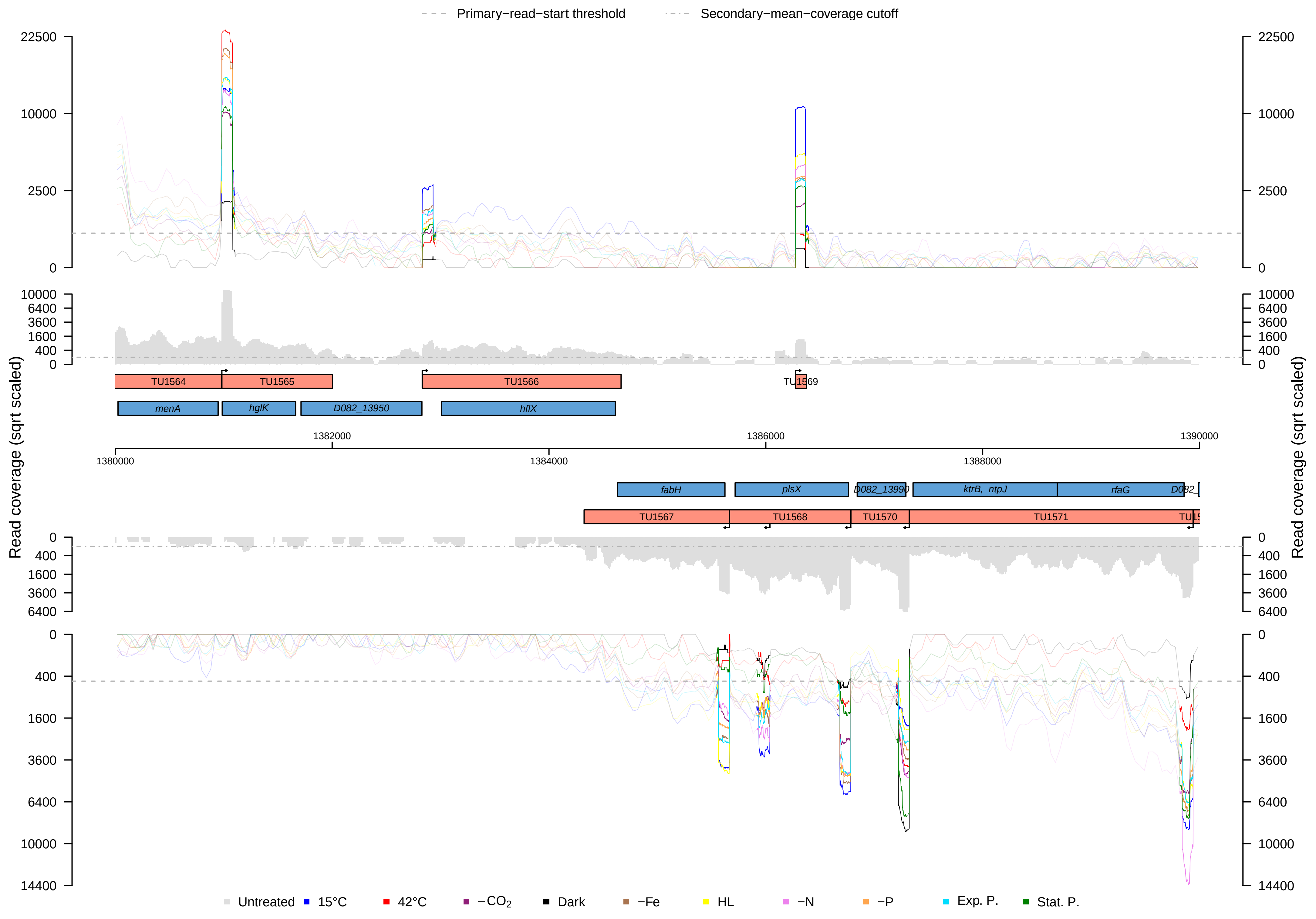

Primary−read−start threshold
Secondary−mean−coverage cutoff
Read coverage (sqrt scaled)
22500
10000
2500
0
6400
3600
1600
400
14400
TU1564
TU1565
TU1566
TU1569
menA
hglK
D082_13950
hflX
1380000
1382000
1384000
1386000
1388000
1390000
fabH
plsX
D082_13990
ktrB, ntpJ
rfaG
TU1567
TU1568
TU1570
TU1571
Untreated
15°C
42°C
−CO2
Dark
−Fe
HL
−N
−P
Exp. P.
Stat. P.

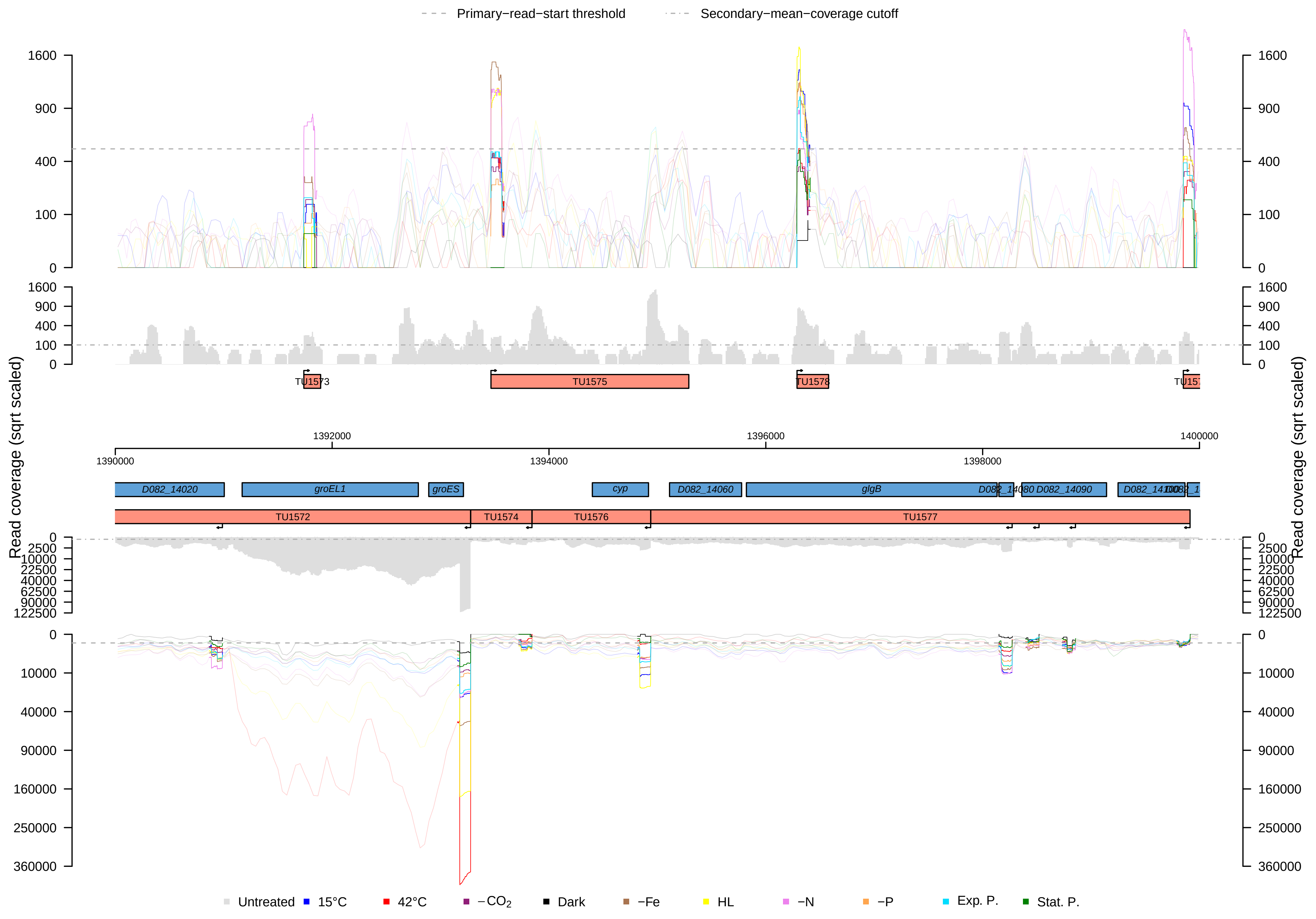

Primary−read−start threshold
Secondary−mean−coverage cutoff
Read coverage (sqrt scaled)
TU1573
TU1575
TU1578
TU157
1390000
1392000
1394000
1396000
1398000
1400000
D082_14020
groEL1
groES
cyp
D082_14060
glgB
D082_14090
TU1572
TU1574
TU1576
TU1577
Untreated
15°C
42°C
−CO2
Dark
−Fe
HL
−N
−P
Exp. P.
Stat. P.

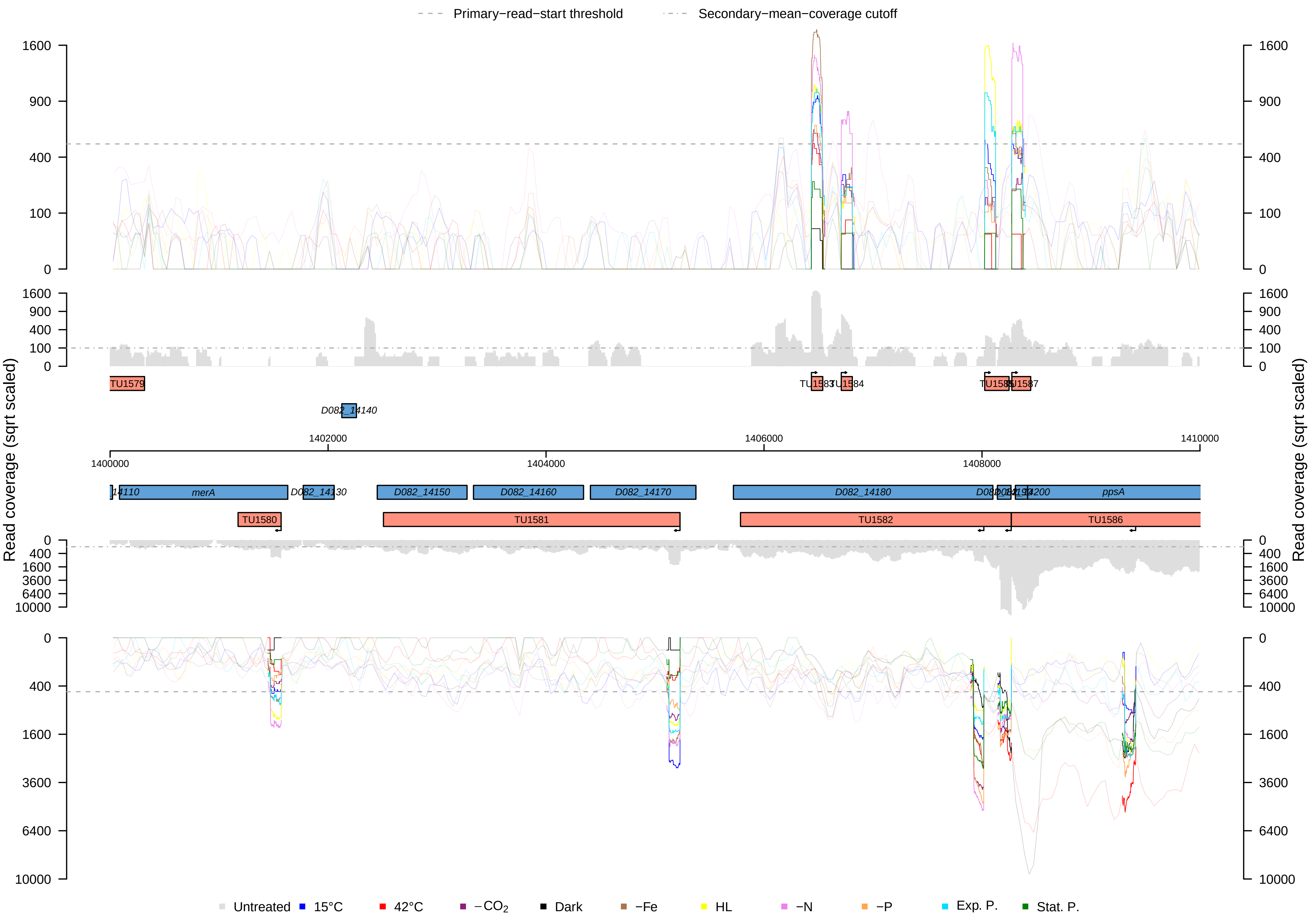

Primary−read−start threshold
Secondary−mean−coverage cutoff
Read coverage (sqrt scaled)
1600
900
400
100
0
TU1579
D082_14140
TU1583
TU1584
TU1587
1400000
1402000
1404000
1406000
1408000
1410000
merA
D082_14130
D082_14150
D082_14160
D082_14170
D082_14180
ppsA
TU1580
TU1581
TU1582
TU1586
3600
6400
10000
Untreated
15°C
42°C
−CO2
Dark
−Fe
HL
−N
−P
Exp. P.
Stat. P.

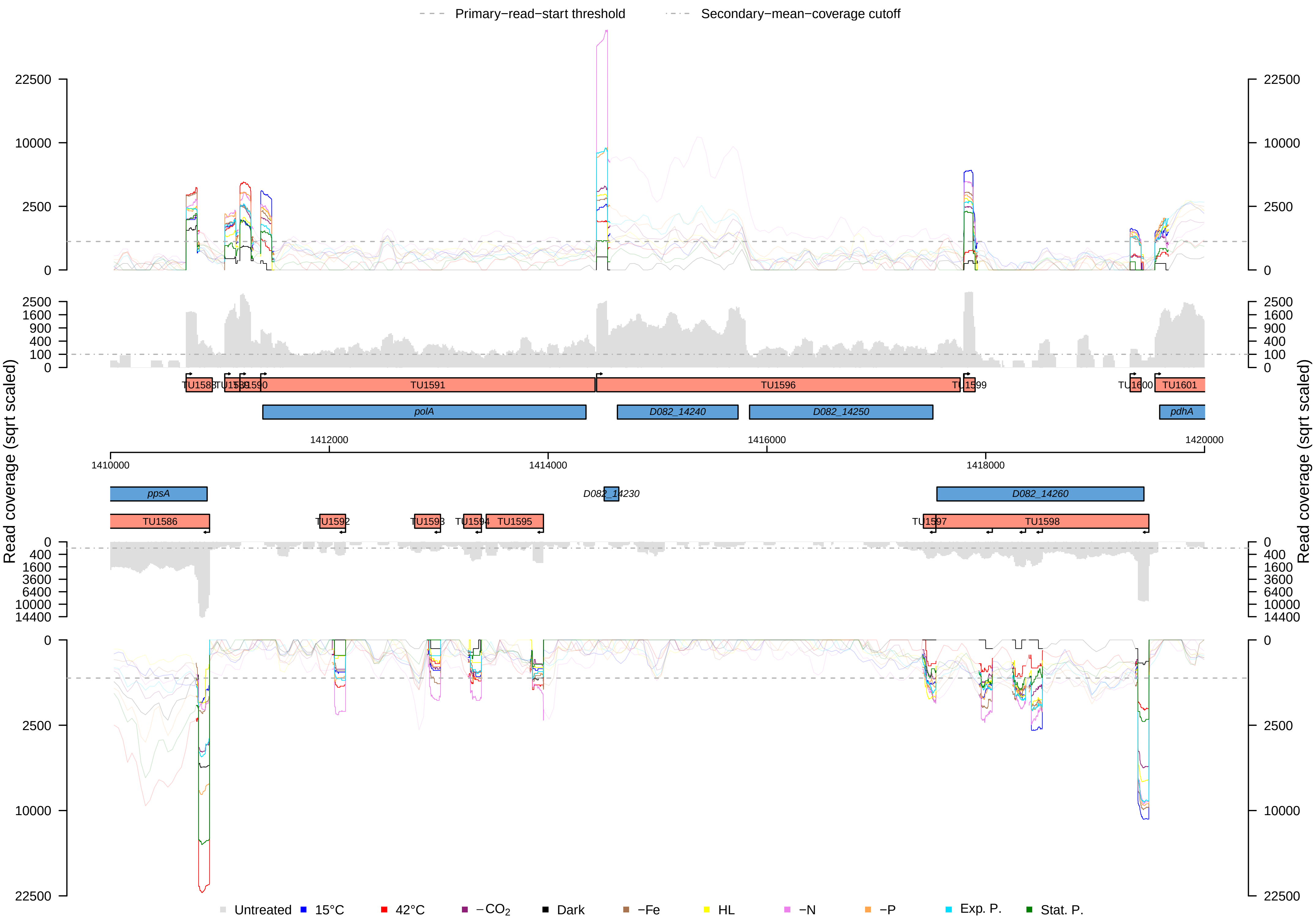

Primary−read−start threshold
Secondary−mean−coverage cutoff
Read coverage (sqrt scaled)
22500
10000
2500
0
2500
1600
900
400
100
0
TU1588
TU1589
TU1590
TU1591
TU1596
TU1599
TU1600
TU1601
polA
D082_14240
D082_14250
pdhA
1410000
1412000
1414000
1416000
1418000
1420000
ppsA
D082_14230
D082_14260
TU1586
TU1592
TU1593
TU1594
TU1595
TU1597
TU1598
400
1600
3600
6400
10000
14400
Untreated
15°C
42°C
−CO2
Dark
−Fe
HL
−N
−P
Exp. P.
Stat. P.

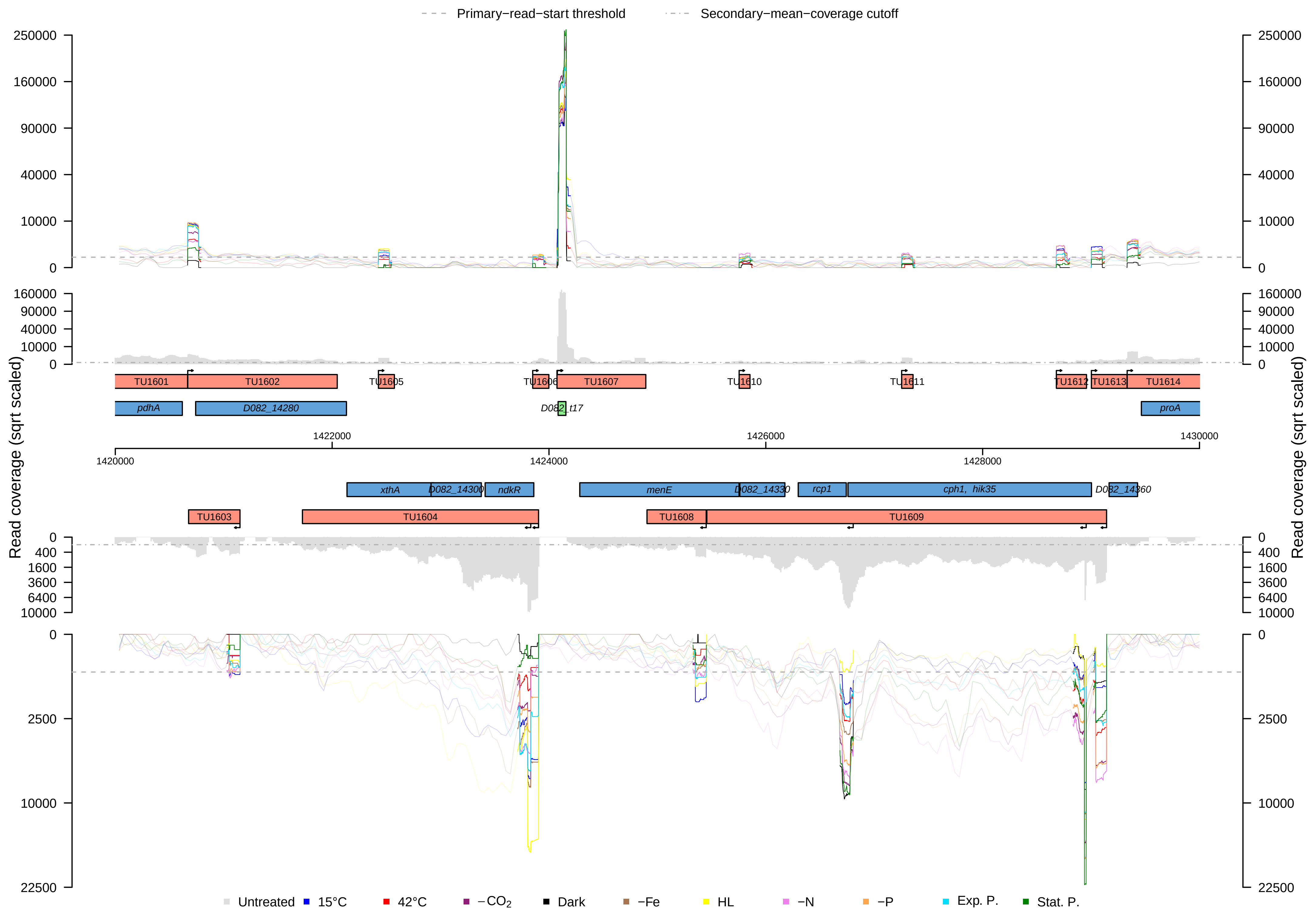

Primary−read−start threshold
Secondary−mean−coverage cutoff
Read coverage (sqrt scaled)
250000
160000
90000
40000
10000
0
400
1600
3600
6400
2500
22500
TU1601
TU1602
TU1605
TU1606
TU1607
TU1610
TU1611
TU1612
TU1613
TU1614
pdhA
D082_14280
D082_t17
proA
1420000
1422000
1424000
1426000
1428000
1430000
xthA
D082_14300
ndkR
menE
D082_14330
rcp1
cph1, hik35
D082_14360
TU1603
TU1604
TU1608
TU1609
Untreated
15°C
42°C
−CO2
Dark
−Fe
HL
−N
−P
Exp. P.
Stat. P.

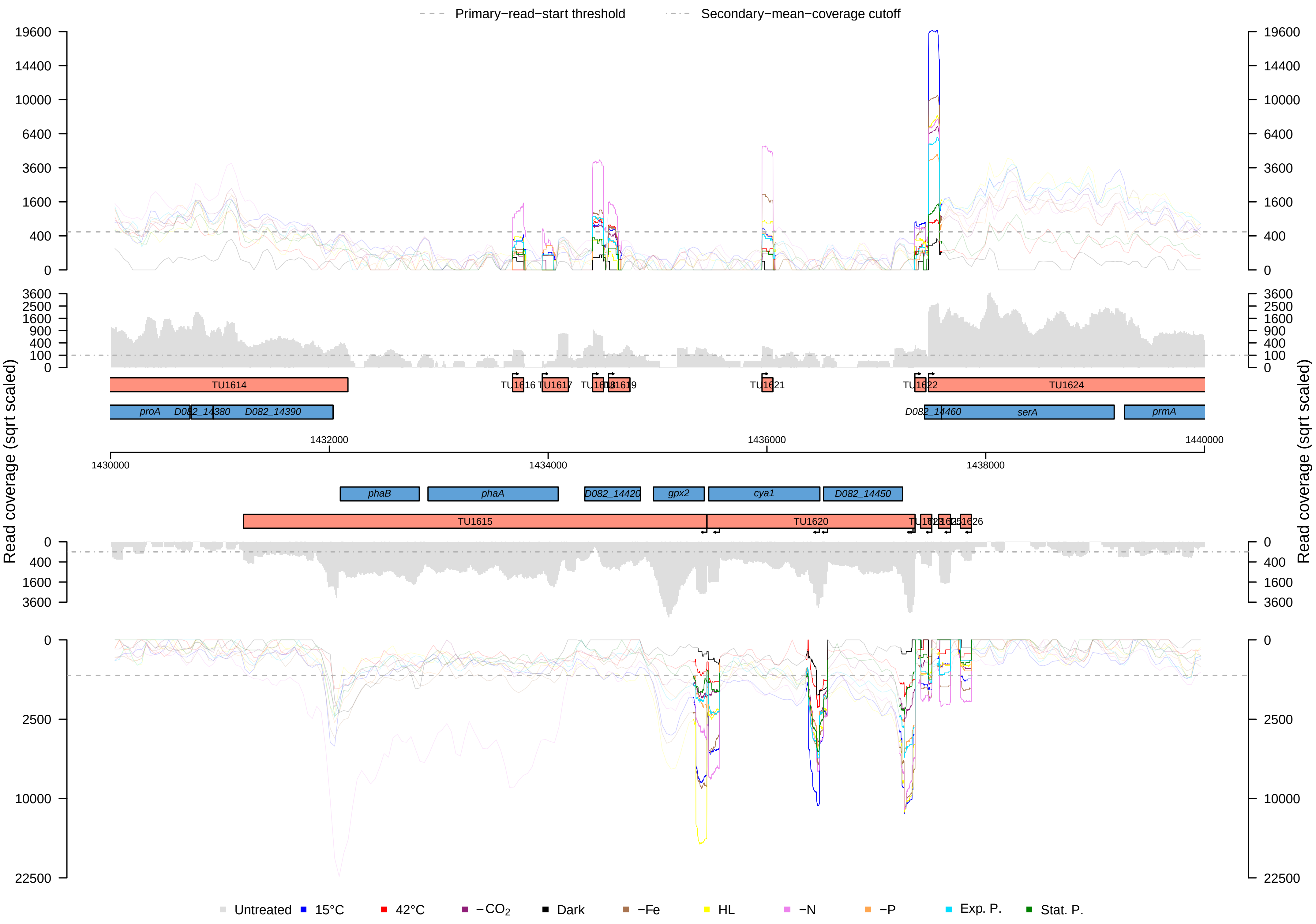

Primary−read−start threshold
Secondary−mean−coverage cutoff
Read coverage (sqrt scaled)
19600
14400
10000
6400
3600
1600
400
0
3600
2500
1600
900
400
100
0
TU1614
TU1616
TU1617
TU1619
TU1621
TU1622
TU1624
proA
D082_14380
D082_14390
D082_14460
serA
prmA
1430000
1432000
1434000
1436000
1438000
1440000
phaB
phaA
D082_14420
gpx2
cya1
D082_14450
TU1615
TU1620
2500
10000
22500
Untreated
15°C
42°C
−CO2
Dark
−Fe
HL
−N
−P
Exp. P.
Stat. P.

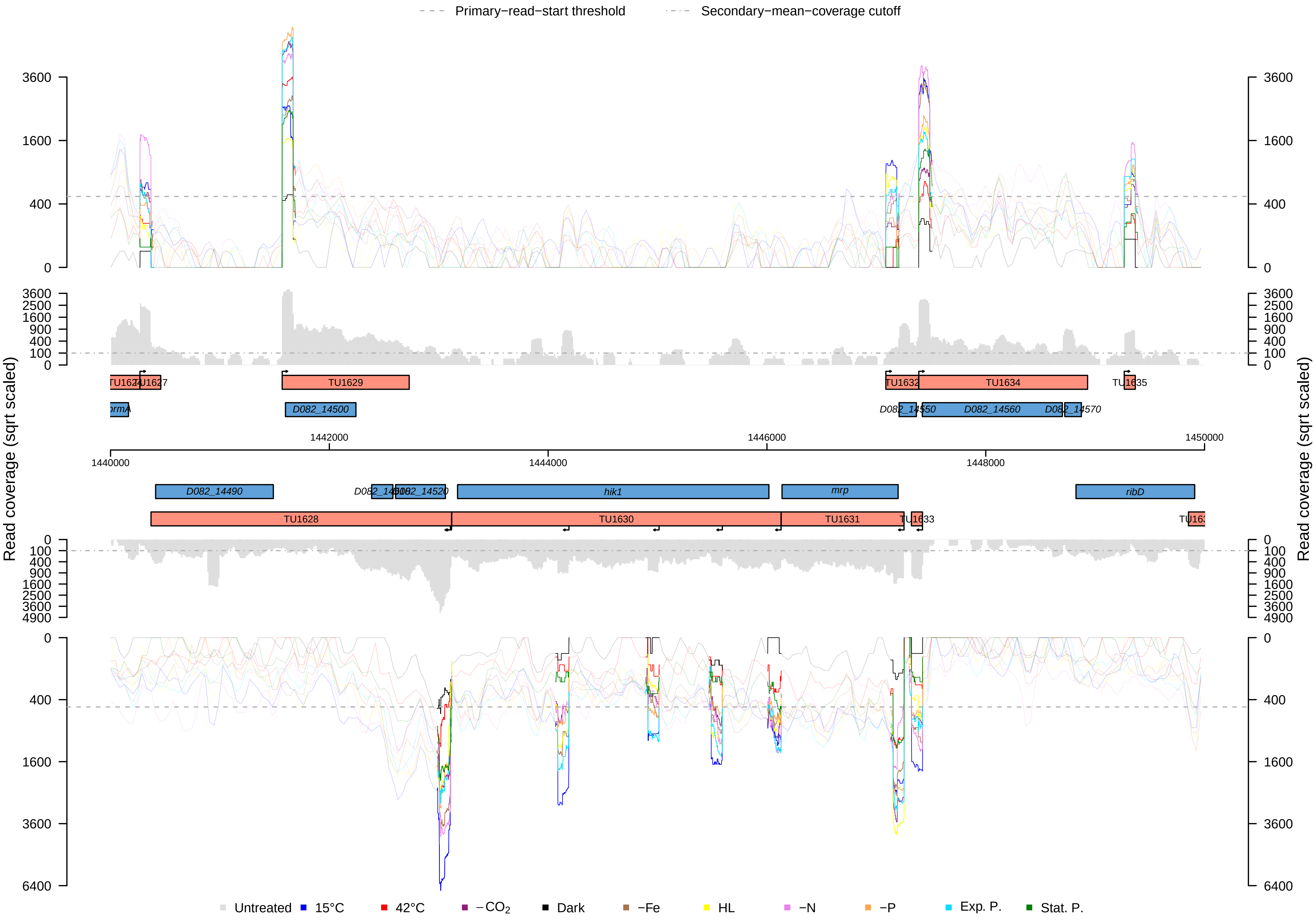

Primary−read−start threshold
Secondary−mean−coverage cutoff
Read coverage (sqrt scaled)
3600
1600
400
0
3600
2500
1600
900
400
100
0
TU1626
TU1627
TU1629
TU1632
TU1634
TU1635
D082_14500
D082_14550
D082_14560
D082_14570
1440000
1442000
1444000
1446000
1448000
1450000
D082_14490
D082_14510
D082_14520
hik1
mrp
ribD
TU1628
TU1630
TU1631
TU1633
4900
6400
Untreated
15°C
42°C
−CO2
Dark
−Fe
HL
−N
−P
Exp. P.
Stat. P.

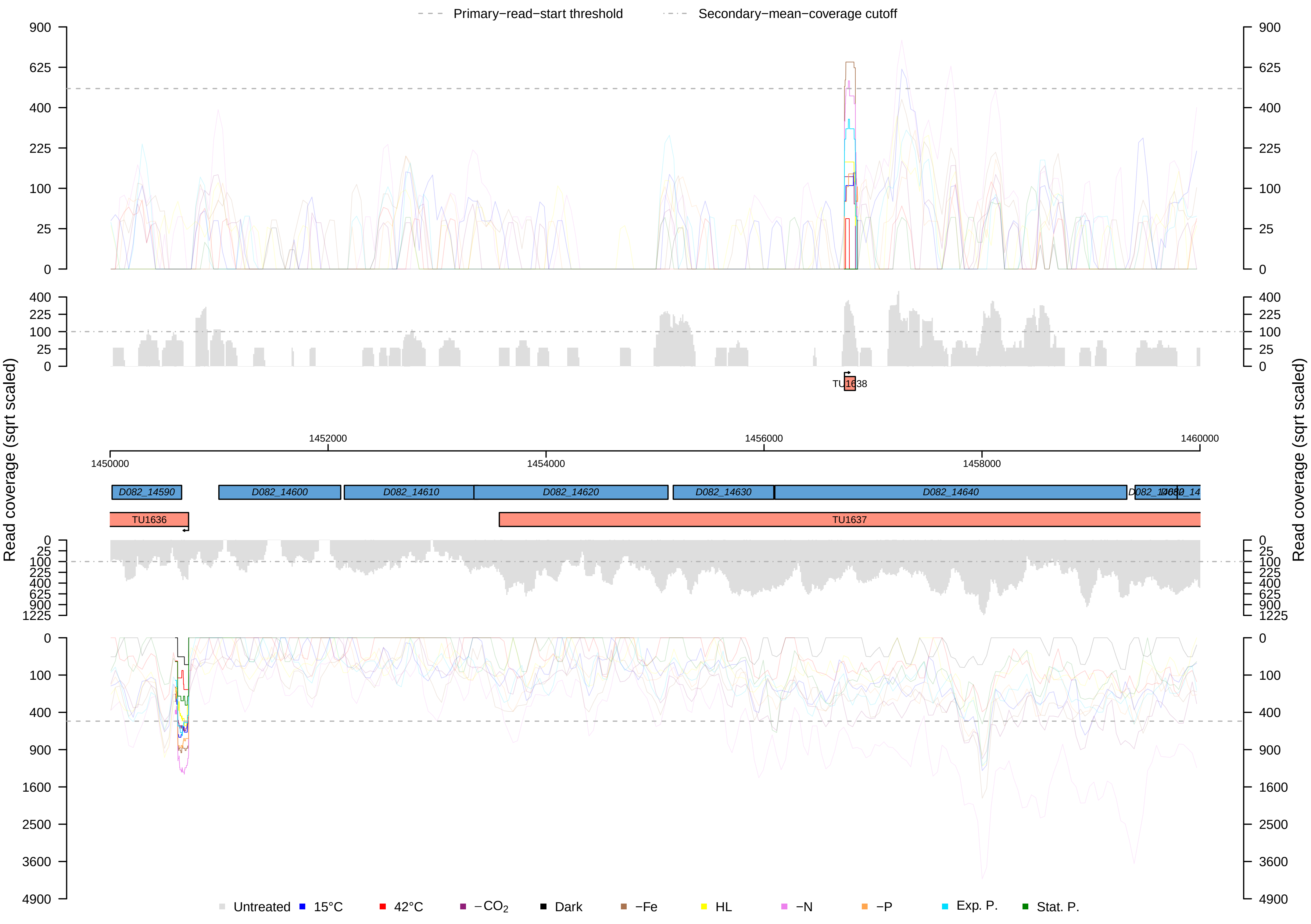

Primary−read−start threshold
Secondary−mean−coverage cutoff
Read coverage (sqrt scaled)
Read coverage (sqrt scaled)
900
625
400
225
100
25
0
TU1638
1450000
1452000
1454000
1456000
1458000
1460000
D082_14590
D082_14600
D082_14610
D082_14620
D082_14630
D082_14640
TU1636
TU1637
1225
1600
2500
3600
4900
Untreated
15°C
42°C
−CO2
Dark
−Fe
HL
−N
−P
Exp. P.
Stat. P.

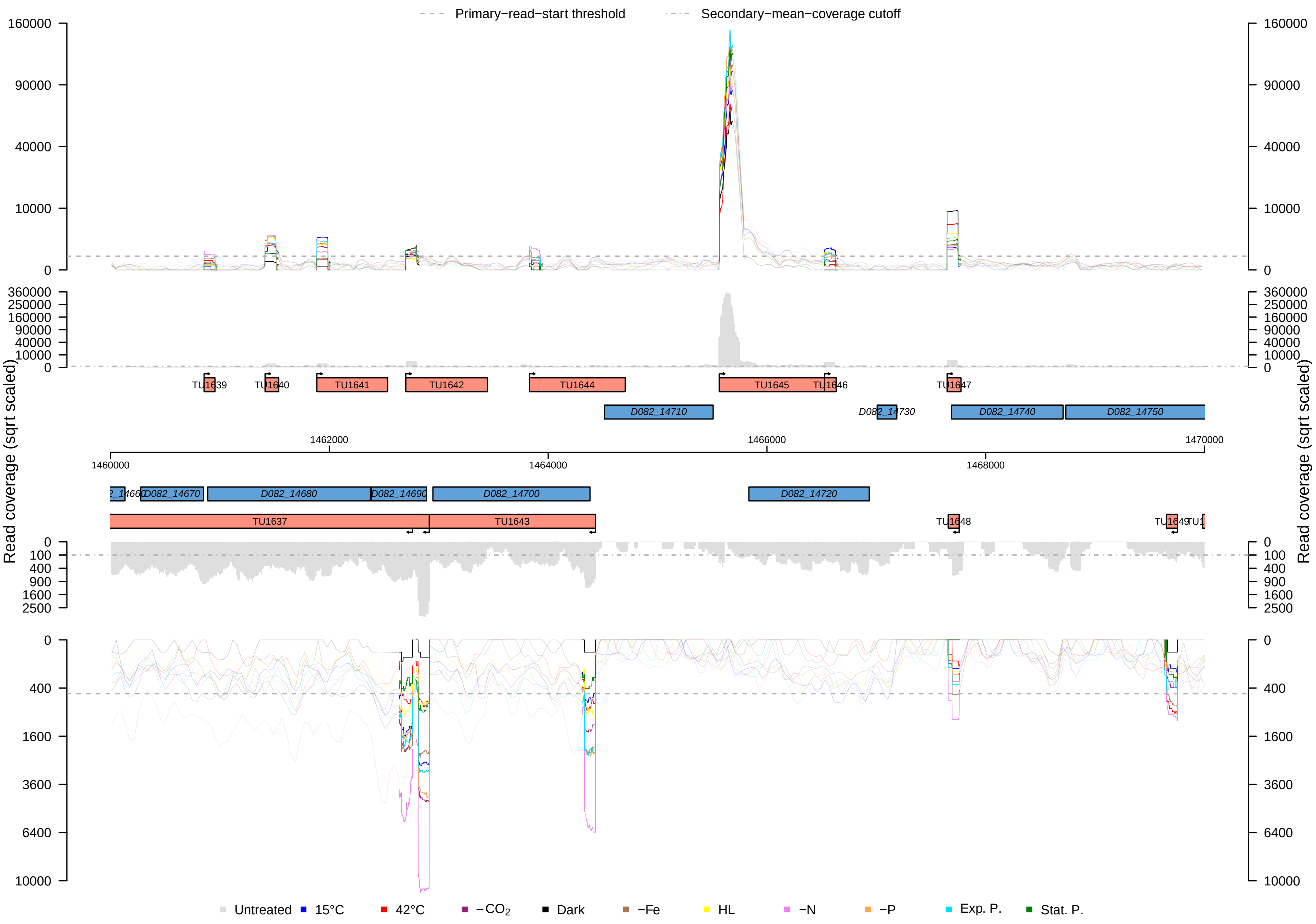

Primary−read−start threshold
Secondary−mean−coverage cutoff
Read coverage (sqrt scaled)
TU1639
TU1640
TU1641
TU1642
TU1644
TU1645
TU1646
TU1647
D082_14710
D082_14730
D082_14740
D082_14750
1460000
1462000
1464000
1466000
1468000
1470000
D082_14670
D082_14680
D082_14690
D082_14700
D082_14720
TU1637
TU1643
TU1648
TU1649
Untreated
15°C
42°C
−CO2
Dark
−Fe
HL
−N
−P
Exp. P.
Stat. P.

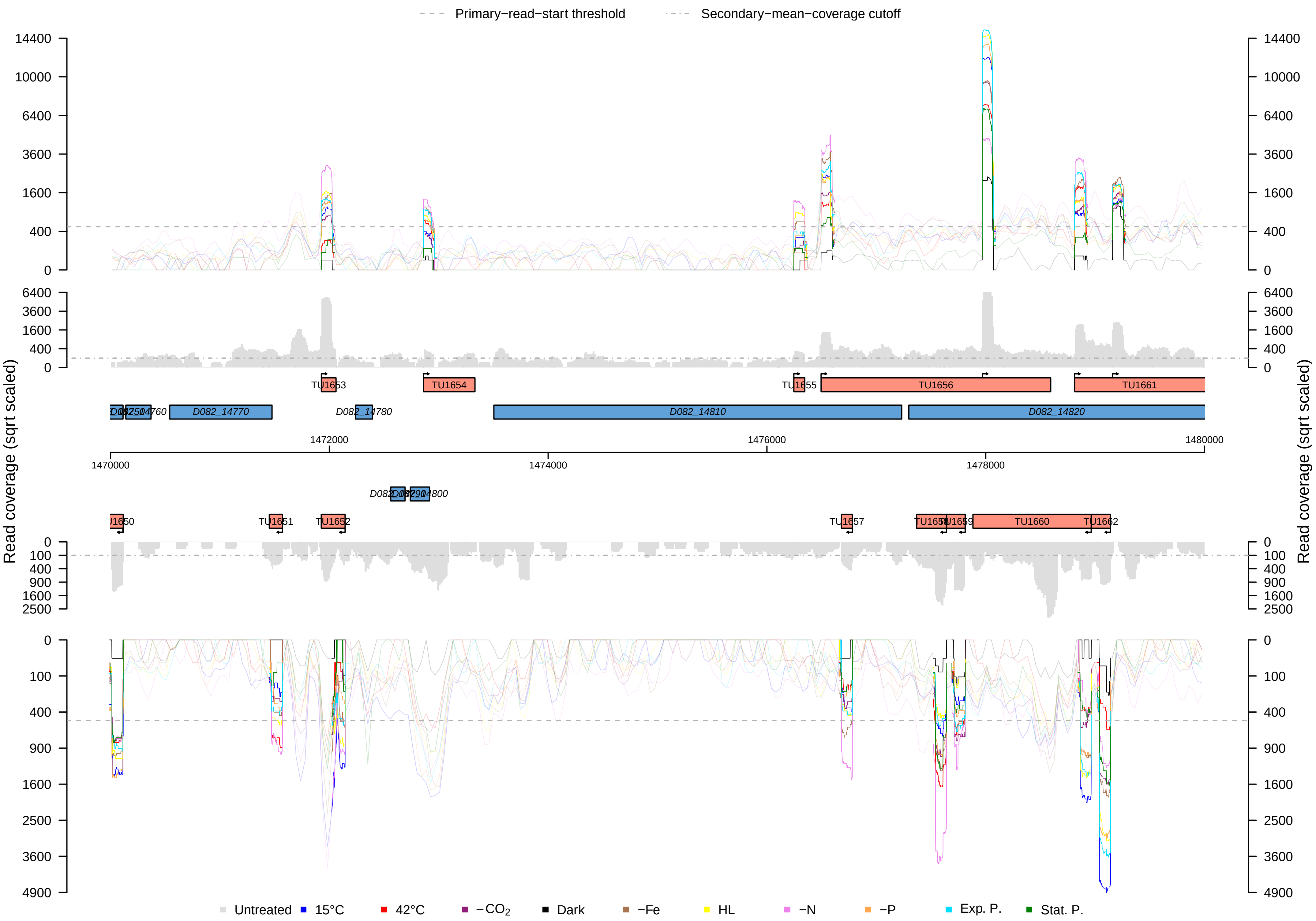

Primary−read−start threshold
Secondary−mean−coverage cutoff
Read coverage (sqrt scaled)
14400
10000
6400
3600
1600
400
0
TU1653
TU1654
TU1655
TU1656
TU1661
D082_14770
D082_14780
D082_14810
D082_14820
1470000
1472000
1474000
1476000
1478000
1480000
TU1651
TU1652
TU1657
TU1660
TU1662
Untreated
15°C
42°C
−CO2
Dark
−Fe
HL
−N
−P
Exp. P.
Stat. P.

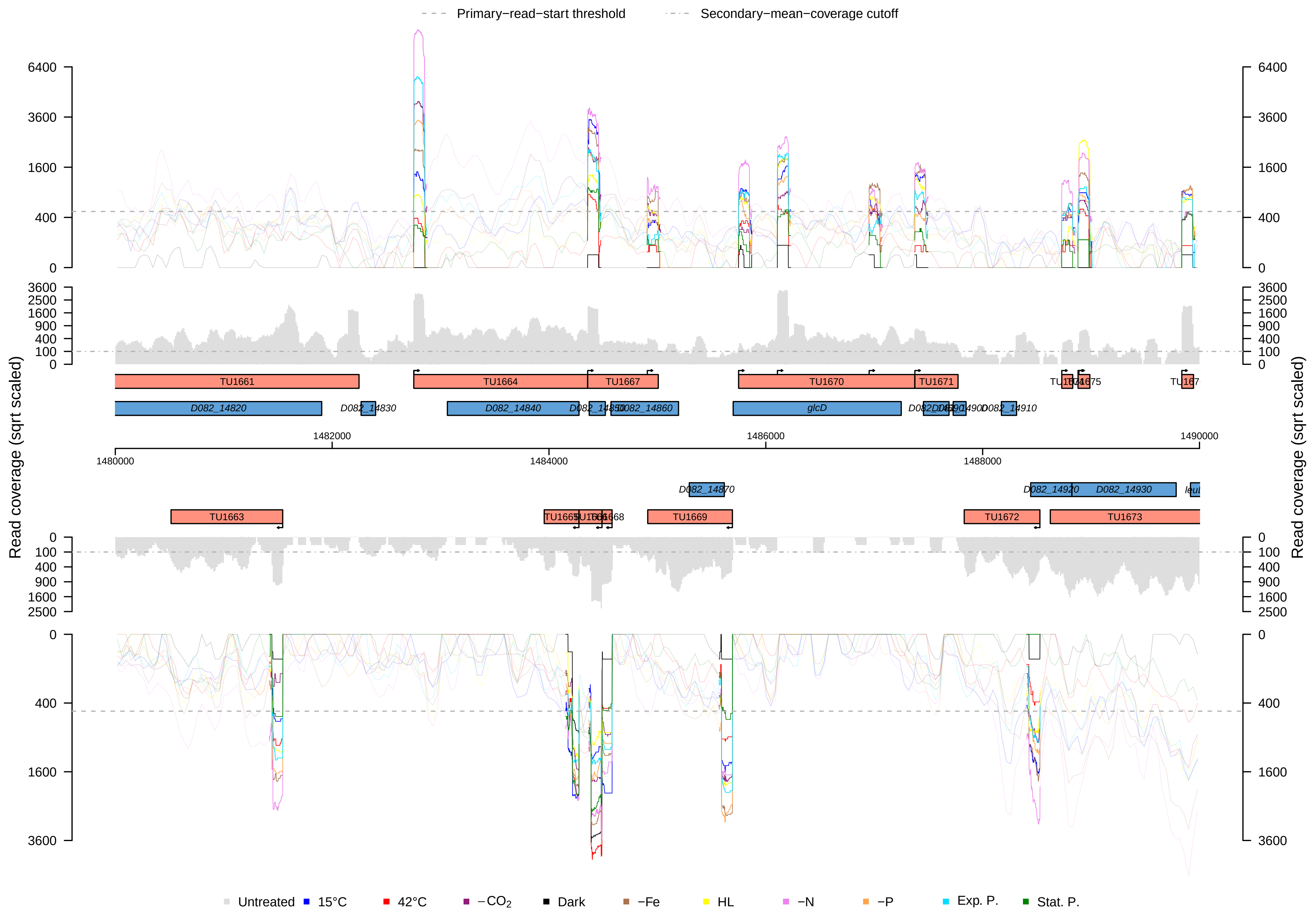

Primary−read−start threshold
Secondary−mean−coverage cutoff
Read coverage (sqrt scaled)
TU1661
TU1664
TU1667
TU1670
TU1671
D082_14820
D082_14830
D082_14840
D082_14860
glcD
D082_14910
D082_14870
D082_14920
D082_14930
TU1663
TU1669
TU1672
TU1673
1480000
1482000
1484000
1486000
1488000
1490000
Untreated
15°C
42°C
−CO2
Dark
−Fe
HL
−N
−P
Exp. P.
Stat. P.

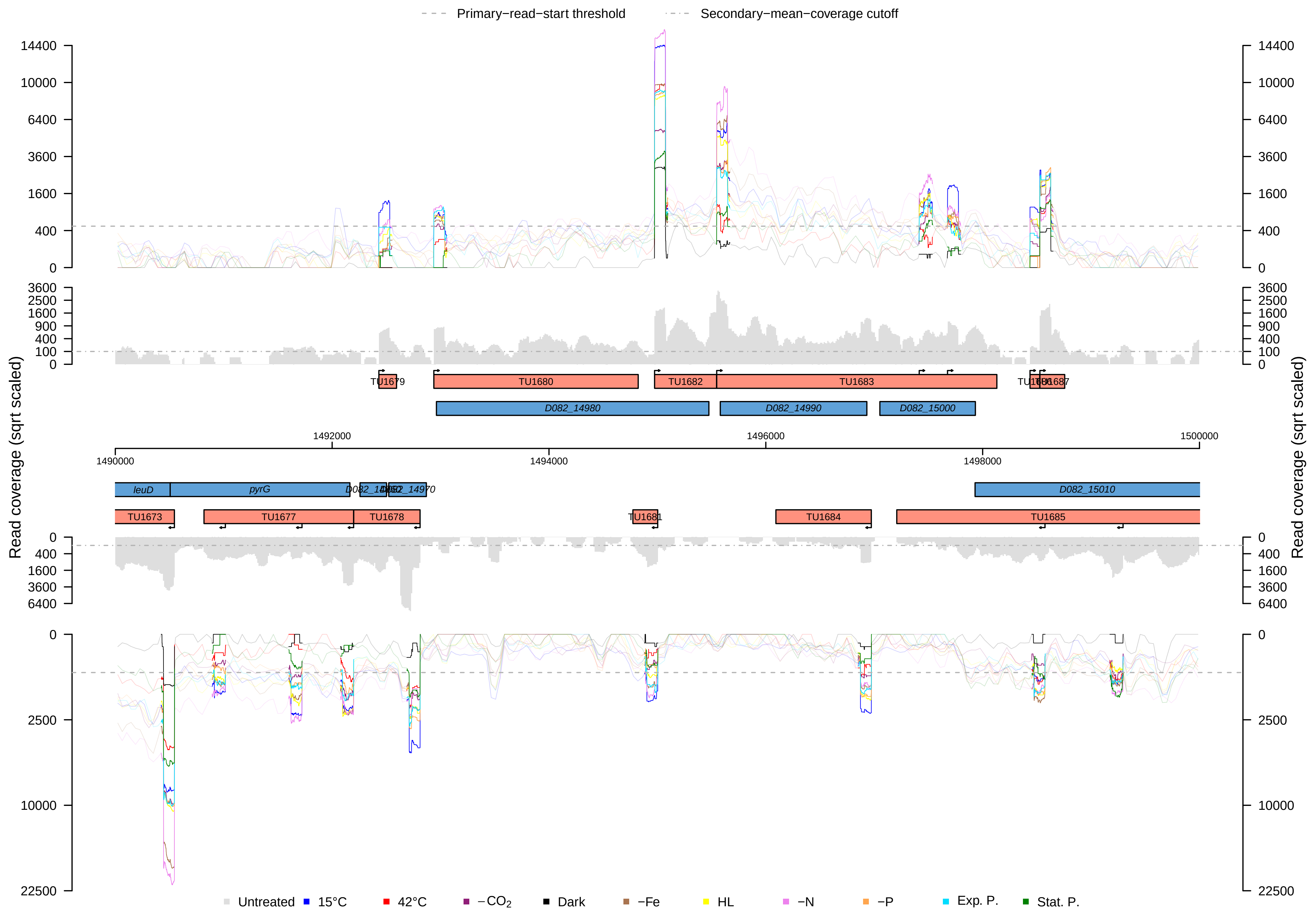

Primary−read−start threshold
Secondary−mean−coverage cutoff
14400
10000
6400
3600
1600
400
0
3600
2500
1600
900
400
100
0
Read coverage (sqrt scaled)
TU1679
TU1680
TU1682
TU1683
D082_14980
D082_14990
D082_15000
1490000
1492000
1494000
1496000
1498000
1500000
leuD
pyrG
D082_15010
TU1673
TU1677
TU1678
TU1681
TU1684
TU1685
2500
10000
22500
Untreated
15°C
42°C
−CO2
Dark
−Fe
HL
−N
−P
Exp. P.
Stat. P.

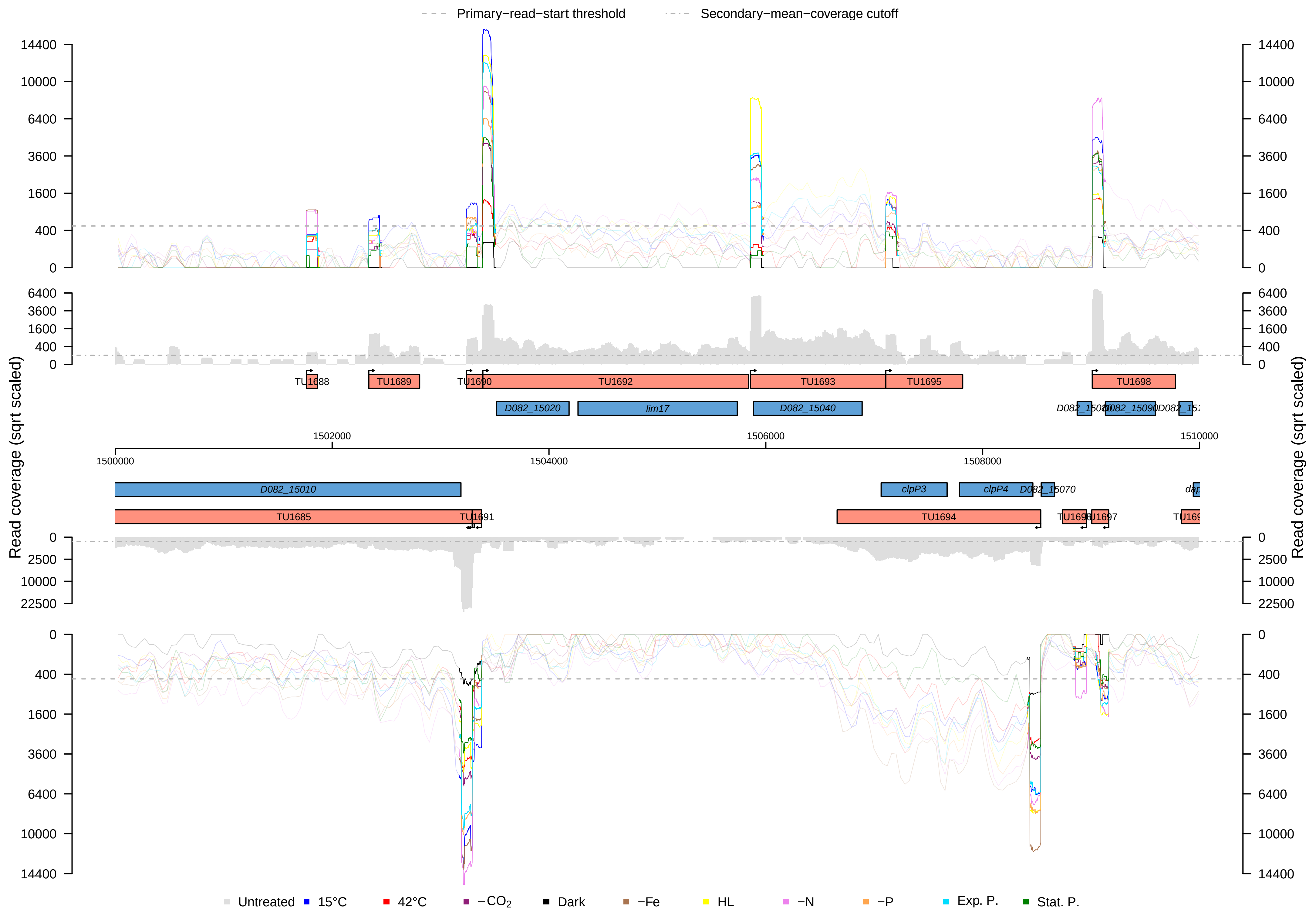

Primary−read−start threshold
Secondary−mean−coverage cutoff
Read coverage (sqrt scaled)
TU1688
TU1689
TU1690
TU1692
TU1693
TU1695
TU1698
D082_15020
lim17
D082_15040
1500000
1502000
1504000
1506000
1508000
1510000
D082_15010
clpP3
clpP4
D082_15070
TU1685
TU1691
TU1694
TU1697
Untreated
15°C
42°C
−CO_2
Dark
−Fe
HL
−N
−P
Exp. P.
Stat. P.

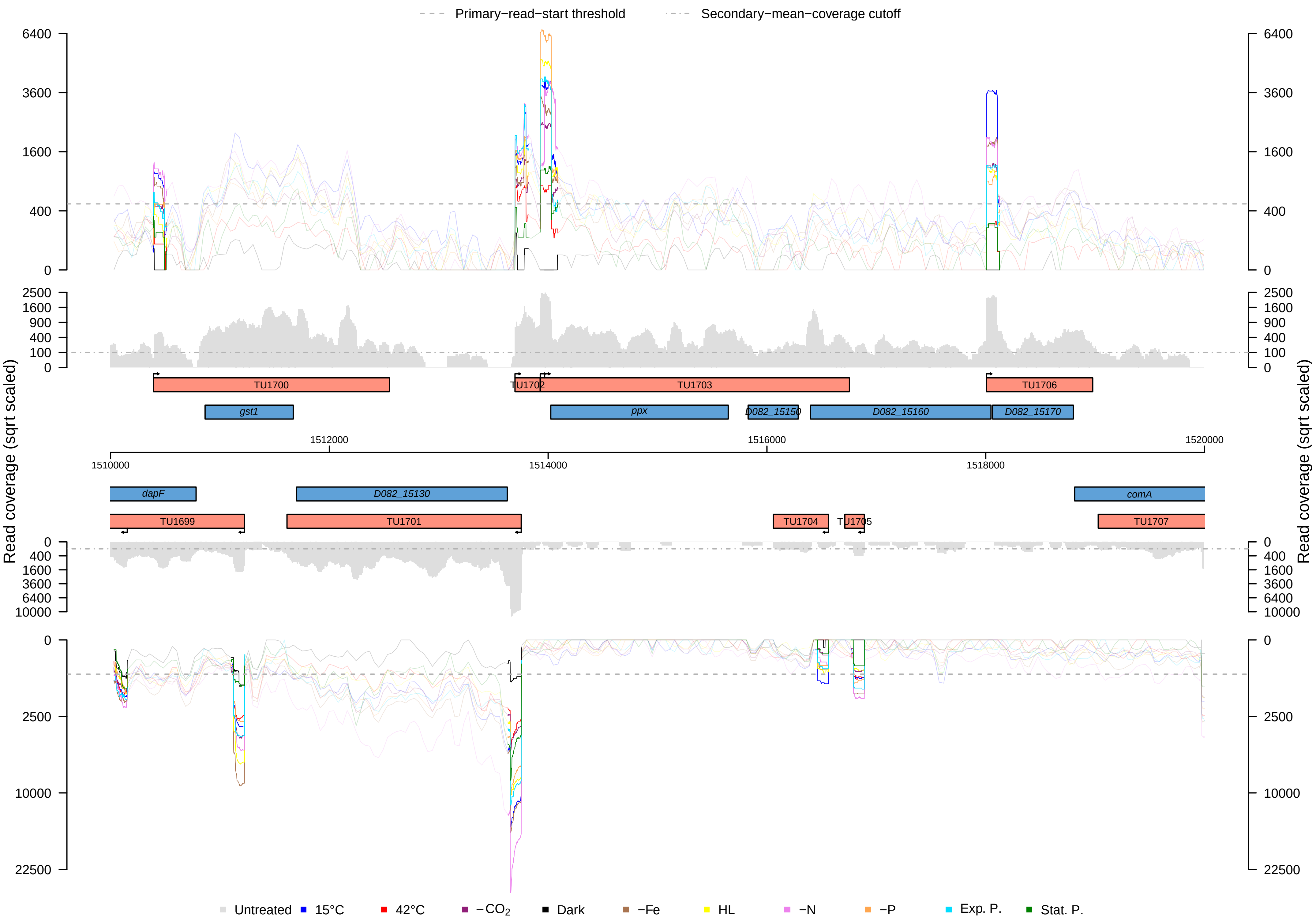

Primary−read−start threshold
Secondary−mean−coverage cutoff
Read coverage (sqrt scaled)
6400
3600
1600
400
0
2500
1600
900
400
100
0
TU1700
TU1702
TU1703
TU1706
gst1
ppx
D082_15150
D082_15160
D082_15170
1510000
1512000
1514000
1516000
1518000
1520000
dapF
D082_15130
comA
TU1699
TU1701
TU1704
TU1705
TU1707
10000
22500
Untreated
15°C
42°C
−CO2
Dark
−Fe
HL
−N
−P
Exp. P.
Stat. P.

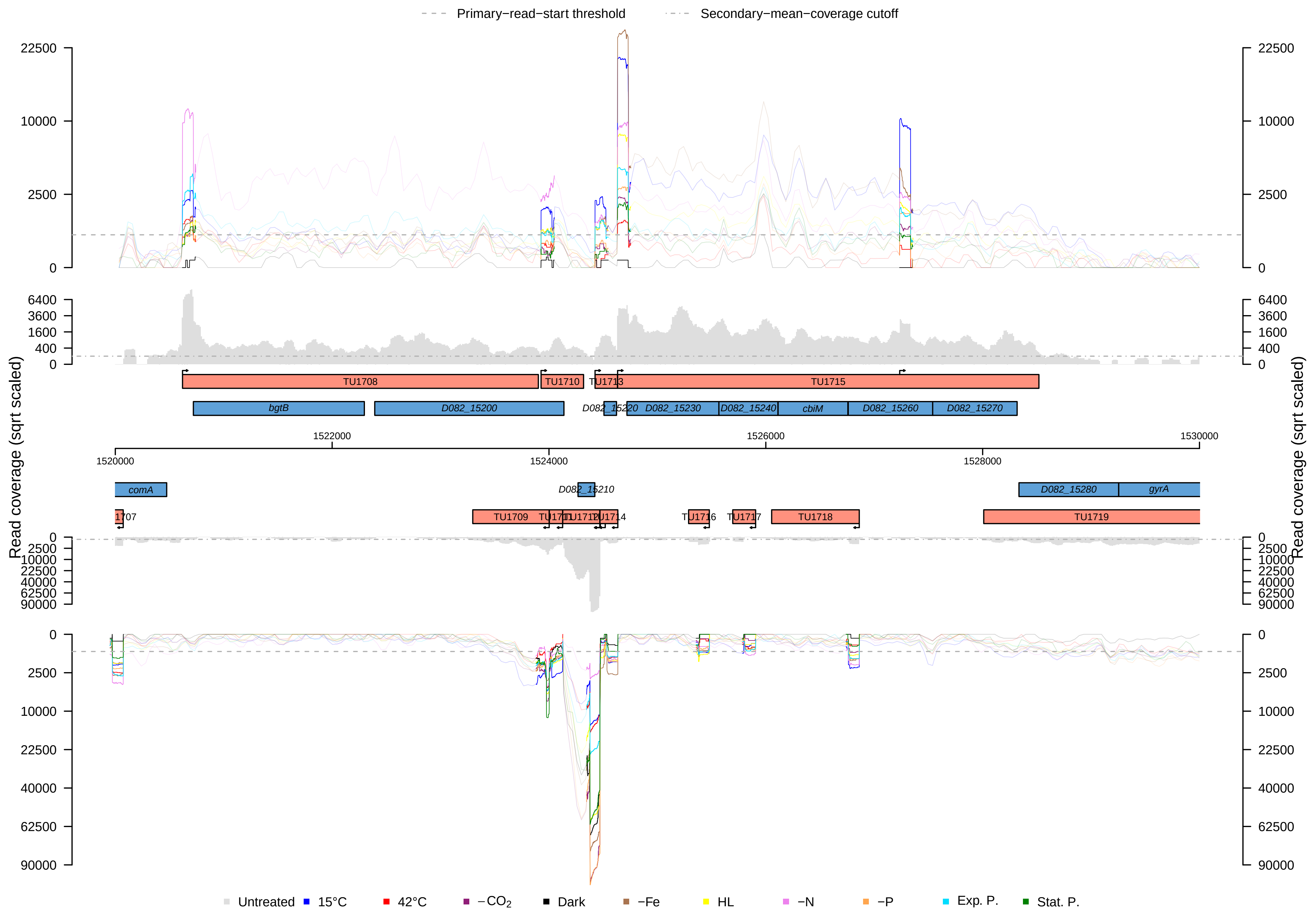

Primary−read−start threshold
Secondary−mean−coverage cutoff
Read coverage (sqrt scaled)
22500
10000
2500
0
6400
3600
1600
400
90000
62500
40000
TU1708
TU1710
TU1713
TU1715
bgtB
D082_15200
D082_15220
D082_15230
D082_15240
cbiM
D082_15260
D082_15270
1520000
1522000
1524000
1526000
1528000
1530000
comA
D082_15210
D082_15280
gyrA
1707
TU1709
TU1711
TU1712
TU1714
TU1716
TU1717
TU1718
TU1719
Untreated
15°C
42°C
−CO_2
Dark
−Fe
HL
−N
−P
Exp. P.
Stat. P.

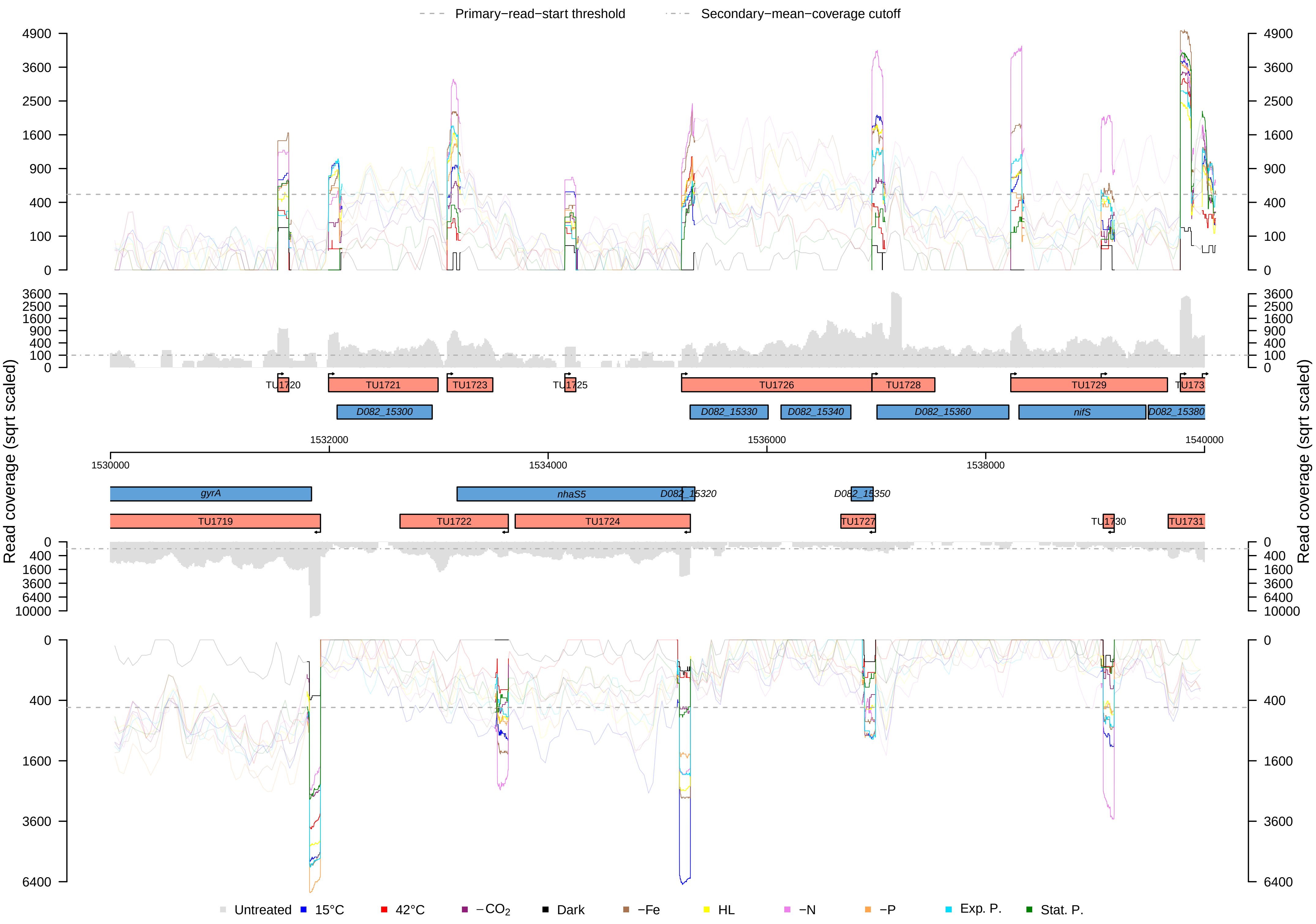

Primary−read−start threshold
Secondary−mean−coverage cutoff
Read coverage (sqrt scaled)
TU1719
TU1720
TU1721
TU1722
TU1723
TU1724
TU1725
TU1726
TU1727
TU1728
TU1729
TU1730
TU173
TU1731
gyrA
nhaS5
D082_15300
D082_15320
D082_15330
D082_15340
D082_15350
D082_15360
nifS
D082_15380
1530000
1532000
1534000
1536000
1538000
1540000
Untreated
15°C
42°C
−CO2
Dark
−Fe
HL
−N
−P
Exp. P.
Stat. P.

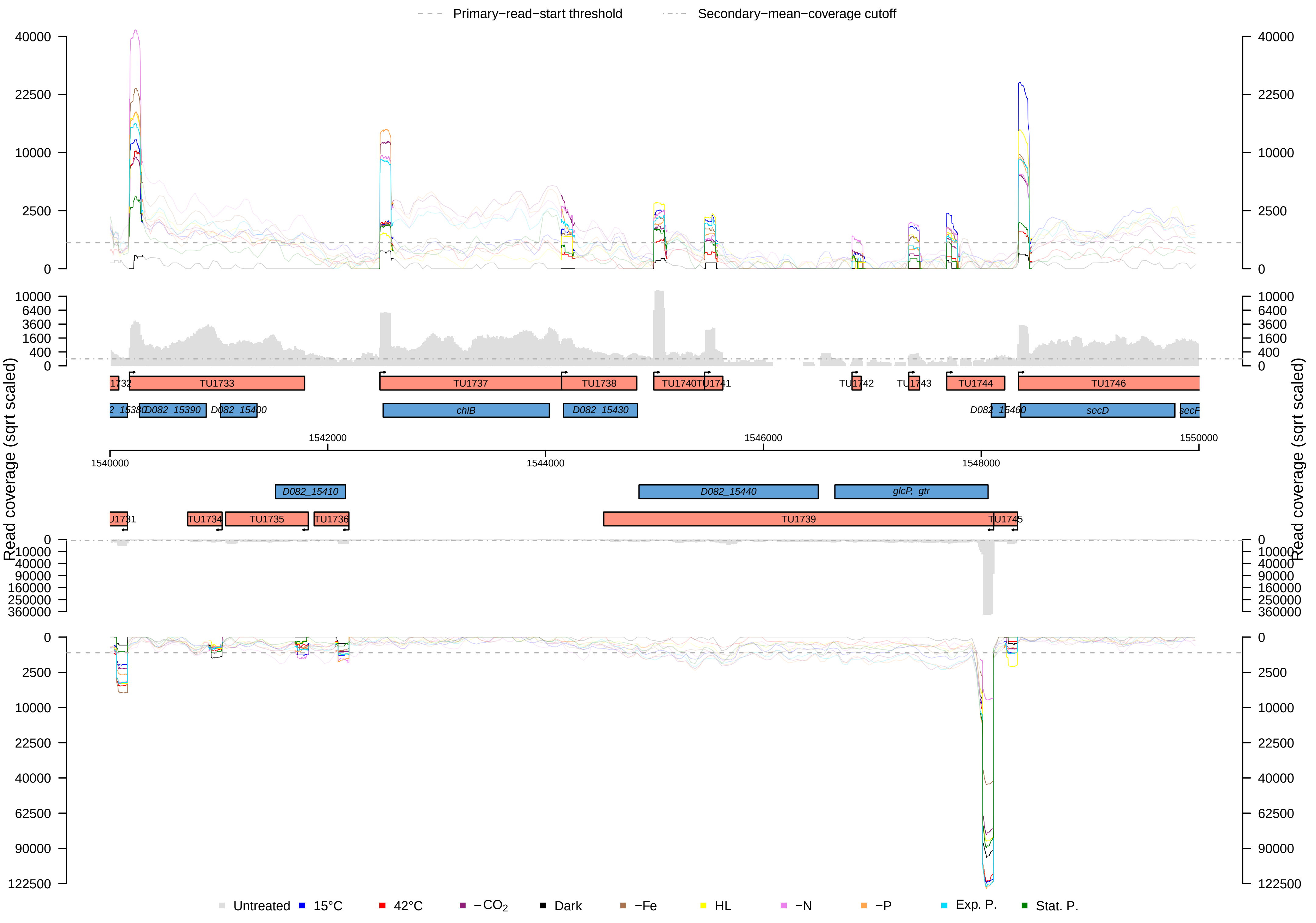

Primary−read−start threshold
Secondary−mean−coverage cutoff
Read coverage (sqrt scaled)
TU1733
TU1737
TU1738
TU1740
TU1741
TU1742
TU1743
TU1744
TU1746
D082_15390
chlB
D082_15430
secD
D082_15410
D082_15440
glcP, gtr
TU1734
TU1735
TU1736
TU1739
TU1745
1540000
1542000
1544000
1546000
1548000
1550000
Untreated
15°C
42°C
−CO2
Dark
−Fe
HL
−N
−P
Exp. P.
Stat. P.

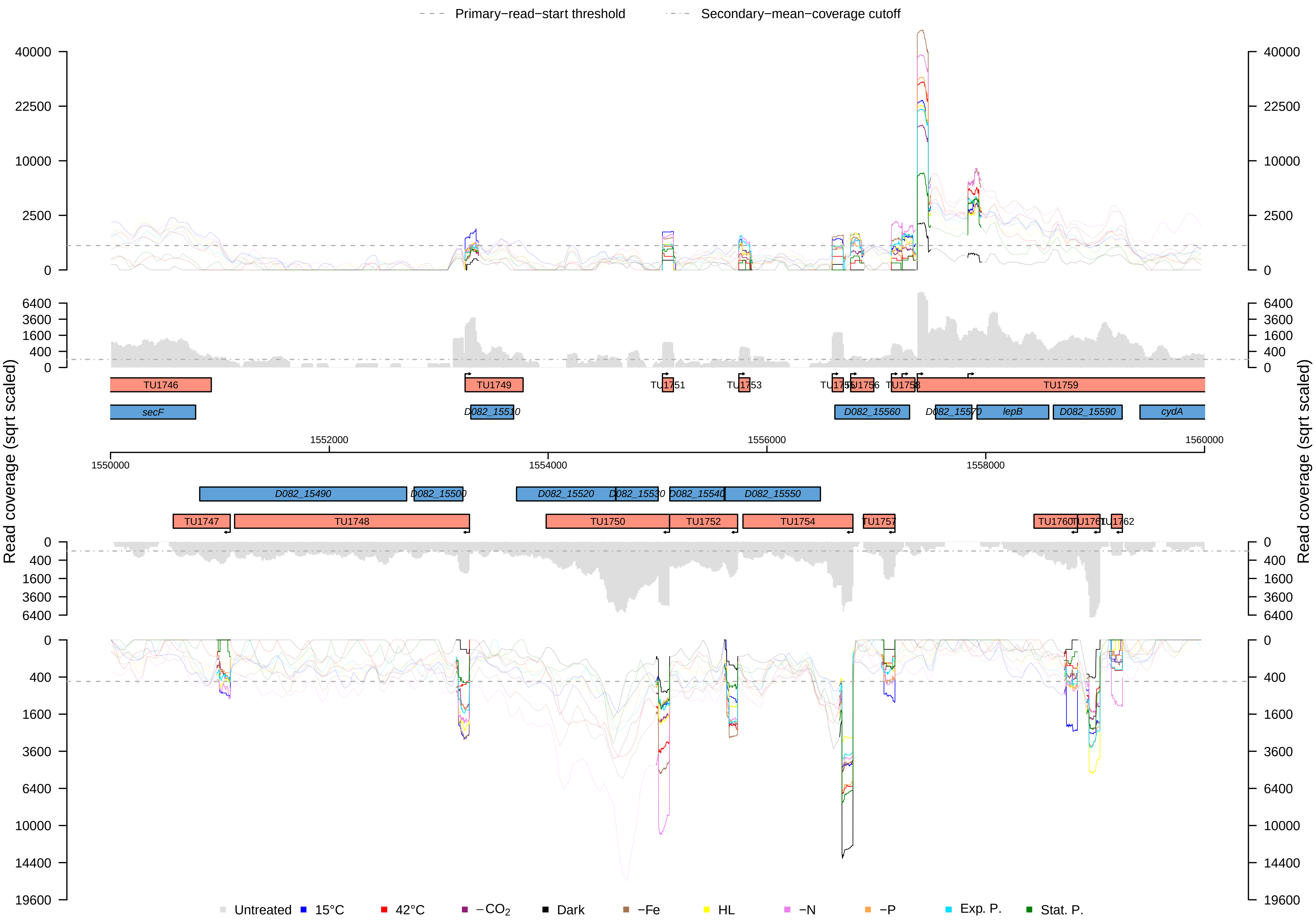

Primary−read−start threshold
Secondary−mean−coverage cutoff
Read coverage (sqrt scaled)
TU1746
TU1749
TU1751
TU1753
TU1756
TU1759
secF
D082_15510
D082_15560
D082_15570
lepB
D082_15590
cydA
1550000
1552000
1554000
1556000
1558000
1560000
D082_15490
D082_15500
D082_15520
D082_15530
D082_15540
D082_15550
TU1747
TU1748
TU1750
TU1752
TU1754
TU1757
TU1760
Untreated
15°C
42°C
−CO2
Dark
−Fe
HL
−N
−P
Exp. P.
Stat. P.

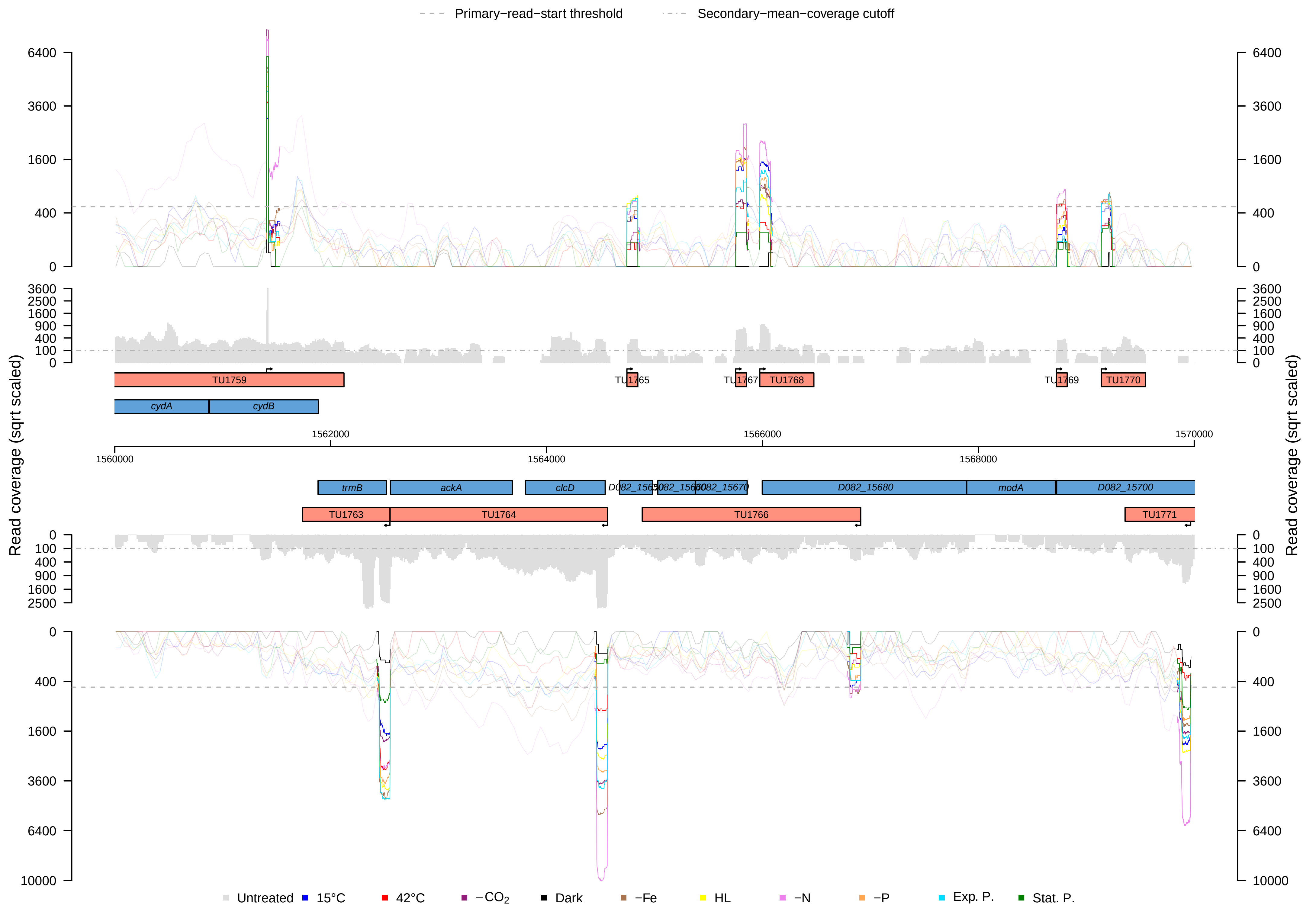

Primary−read−start threshold
Secondary−mean−coverage cutoff
Read coverage (sqrt scaled)
TU1759
TU1765
TU1767
TU1768
TU1769
TU1770
cydA
cydB
1560000
1562000
1564000
1566000
1568000
1570000
trmB
ackA
clcD
D082_15650
D082_15660
D082_15670
D082_15680
modA
D082_15700
TU1763
TU1764
TU1766
TU1771
Untreated
15°C
42°C
−CO2
Dark
−Fe
HL
−N
−P
Exp. P.
Stat. P.

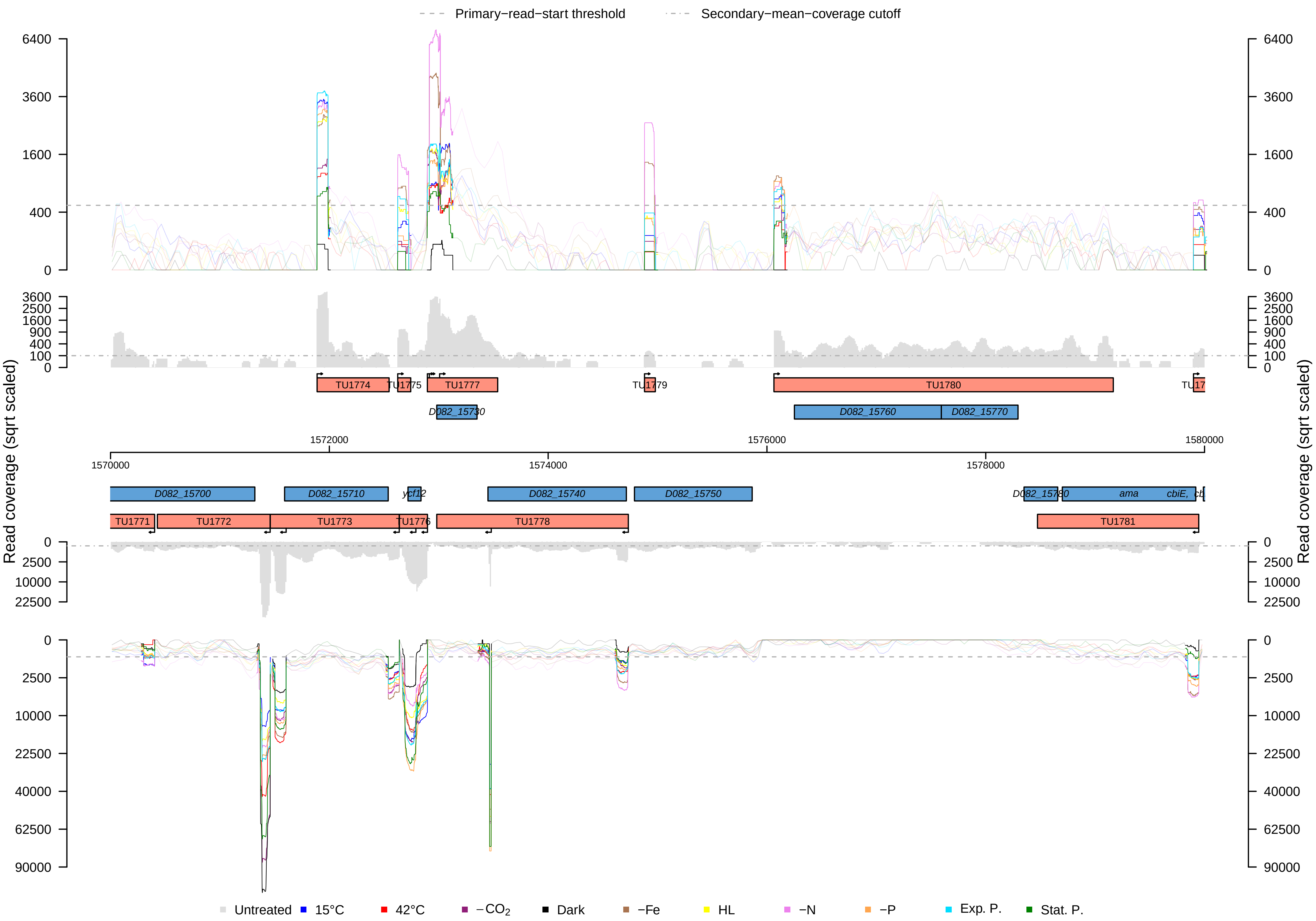

Primary−read−start threshold
Secondary−mean−coverage cutoff
Read coverage (sqrt scaled)
6400
3600
1600
400
0
2500
900
100
10000
22500
40000
62500
90000
TU1774
TU1775
TU1777
TU1779
TU1780
D082_15730
D082_15760
D082_15770
1570000
1572000
1574000
1576000
1578000
1580000
D082_15700
D082_15710
ycf12
D082_15740
D082_15750
D082_15780
ama
cbiE,
TU1771
TU1772
TU1773
TU1776
TU1778
TU1781
Untreated
15°C
42°C
$-CO_2$
Dark
−Fe
HL
−N
−P
Exp. P.
Stat. P.

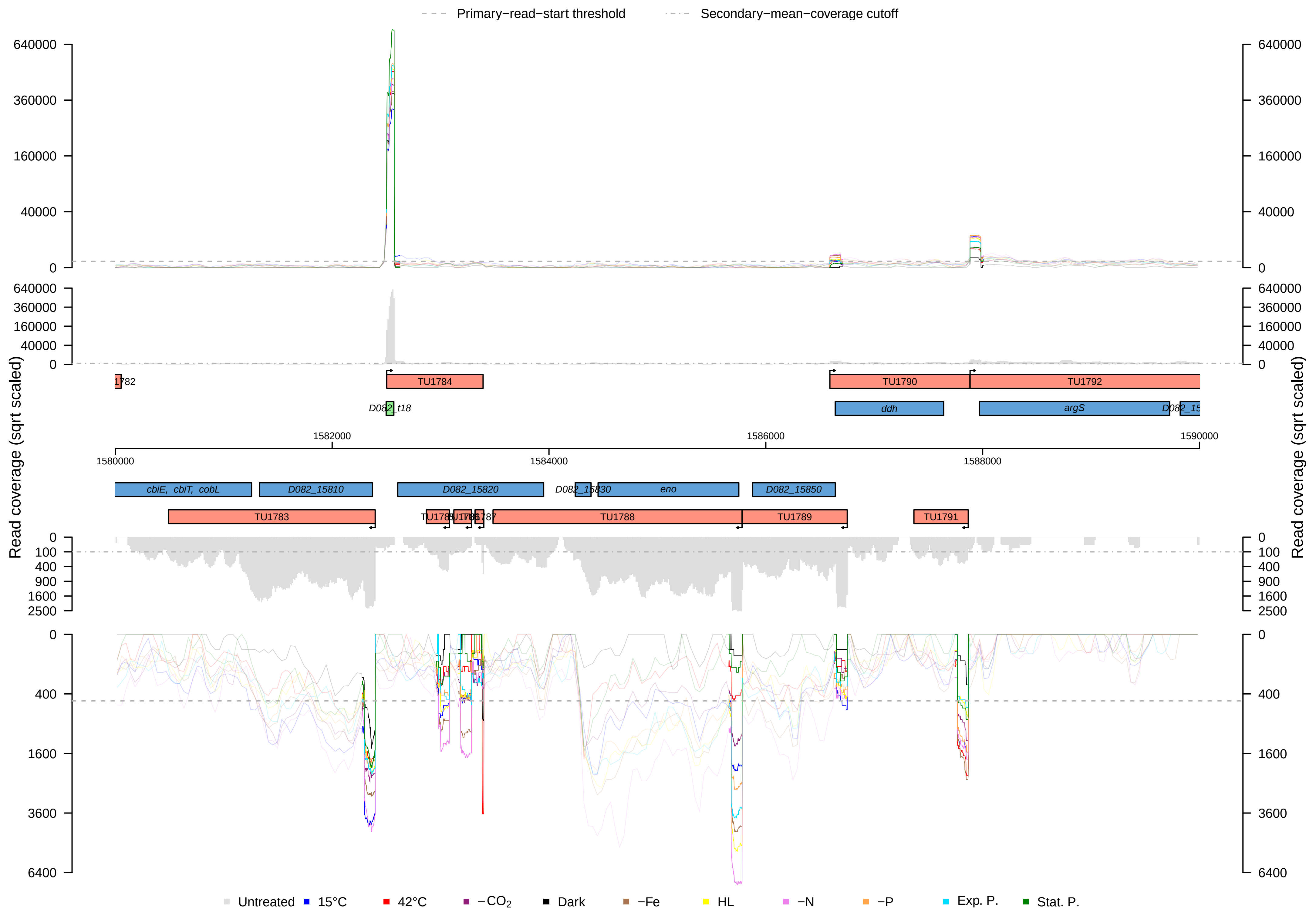

Primary−read−start threshold
Secondary−mean−coverage cutoff
Read coverage (sqrt scaled)
640000
360000
160000
40000
0
TU1784
TU1790
TU1792
D082_t18
ddh
argS
1582000
1586000
1590000
1580000
1584000
1588000
cbiE, cbiT, cobL
D082_15810
D082_15820
eno
D082_15850
TU1783
TU1788
TU1789
TU1791
100
400
900
1600
2500
3600
6400
Untreated
15°C
42°C
−CO2
Dark
−Fe
HL
−N
−P
Exp. P.
Stat. P.

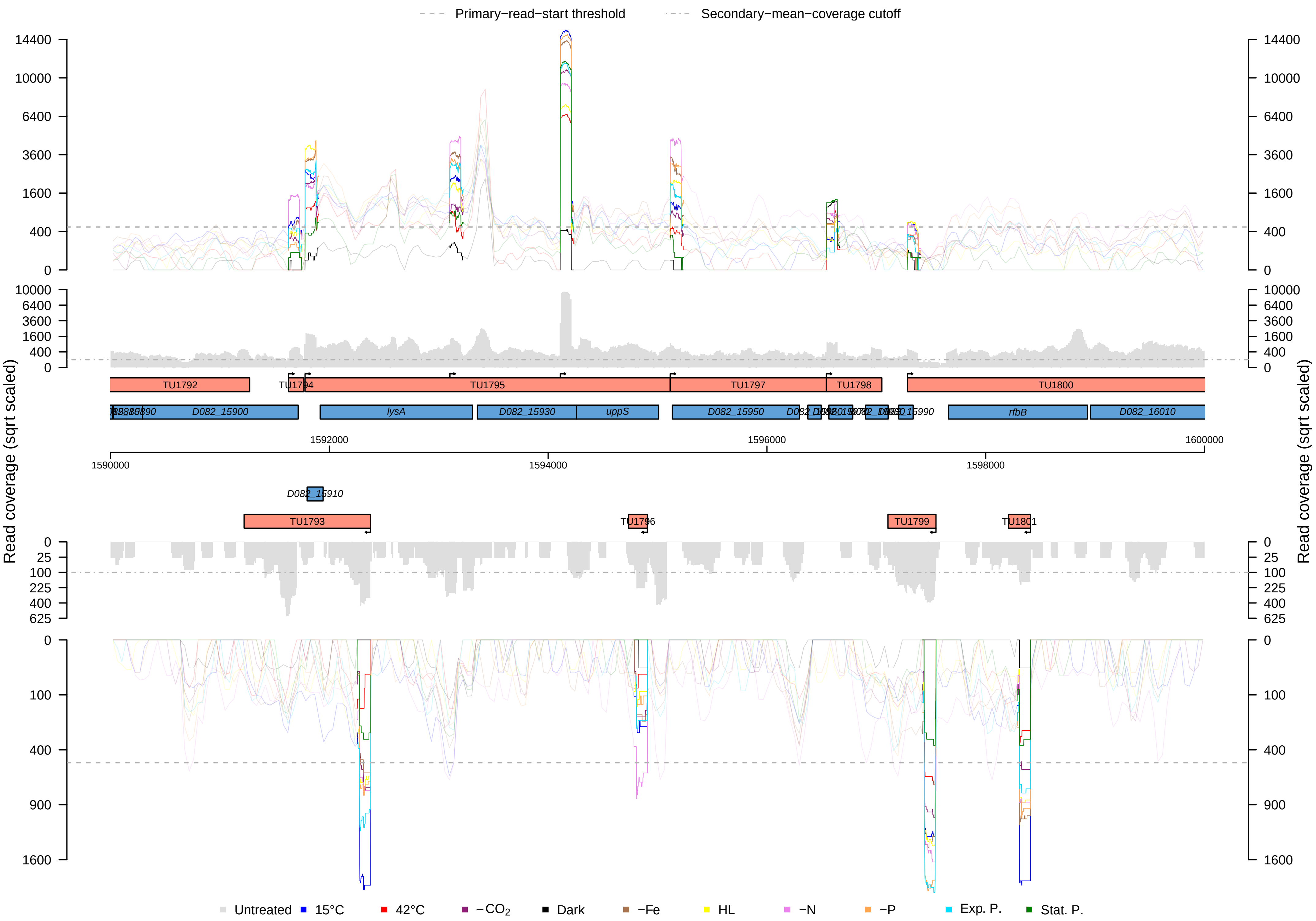

Primary−read−start threshold
Secondary−mean−coverage cutoff
14400
10000
6400
3600
1600
400
0
10000
6400
3600
1600
400
0
TU1792
TU1794
TU1795
TU1797
TU1798
TU1800
D082_15900
lysA
D082_15930
uppS
D082_15950
D082_15990
rfbB
D082_16010
1592000
1596000
1600000
1590000
1594000
1598000
D082_15910
TU1793
TU1796
TU1799
TU1801
0
25
100
225
400
625
0
100
400
900
1600
Read coverage (sqrt scaled)
Read coverage (sqrt scaled)
Untreated
15°C
42°C
−CO2
Dark
−Fe
HL
−N
−P
Exp. P.
Stat. P.

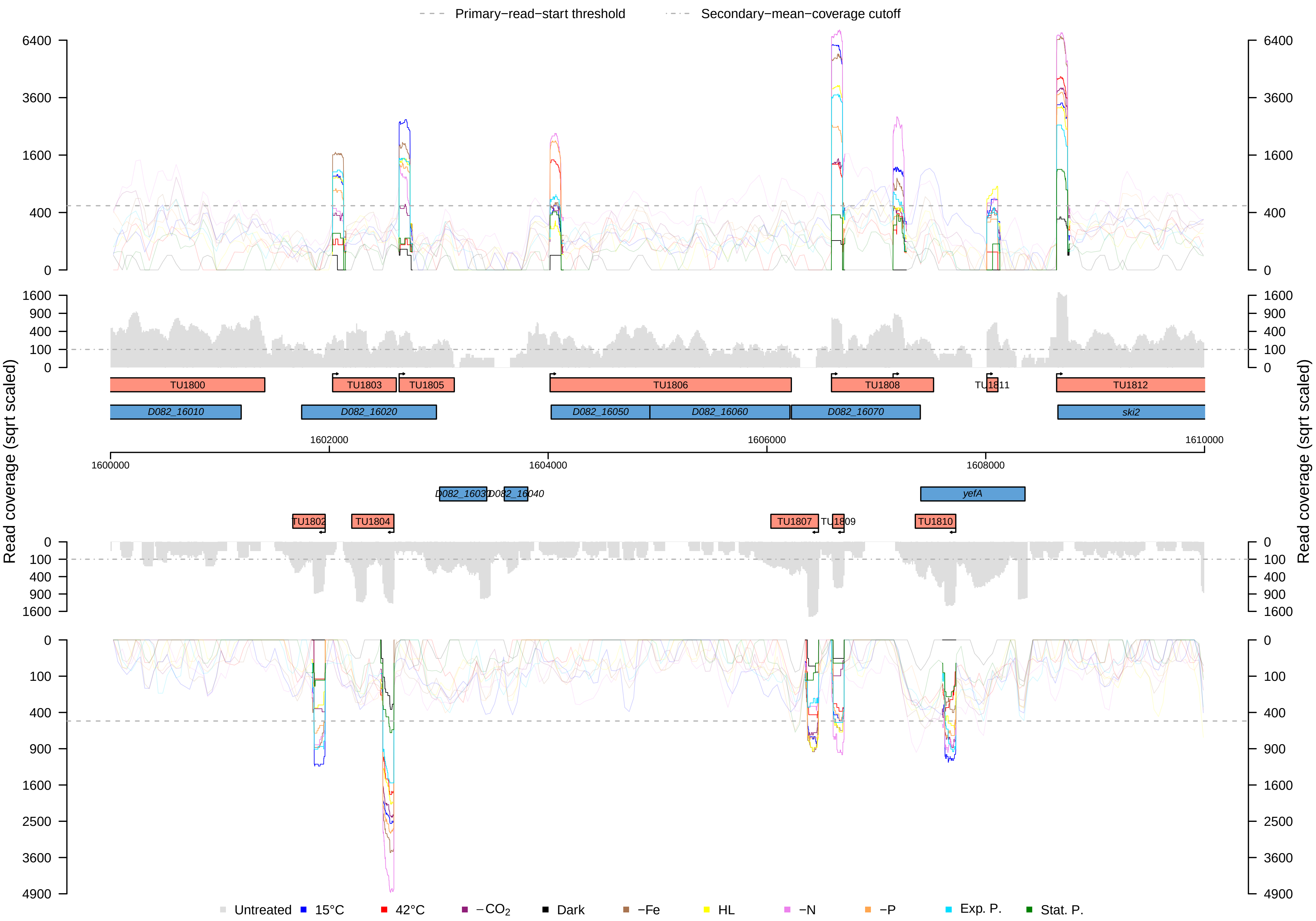

Primary−read−start threshold
Secondary−mean−coverage cutoff
Read coverage (sqrt scaled)
TU1800
TU1803
TU1805
TU1806
TU1808
TU1811
TU1812
D082_16010
D082_16020
D082_16050
D082_16060
D082_16070
ski2
D082_16030
D082_16040
yefA
TU1802
TU1804
TU1807
TU1809
TU1810
1600000
1602000
1604000
1606000
1608000
1610000
Untreated
15°C
42°C
−CO2
Dark
−Fe
HL
−N
−P
Exp. P.
Stat. P.

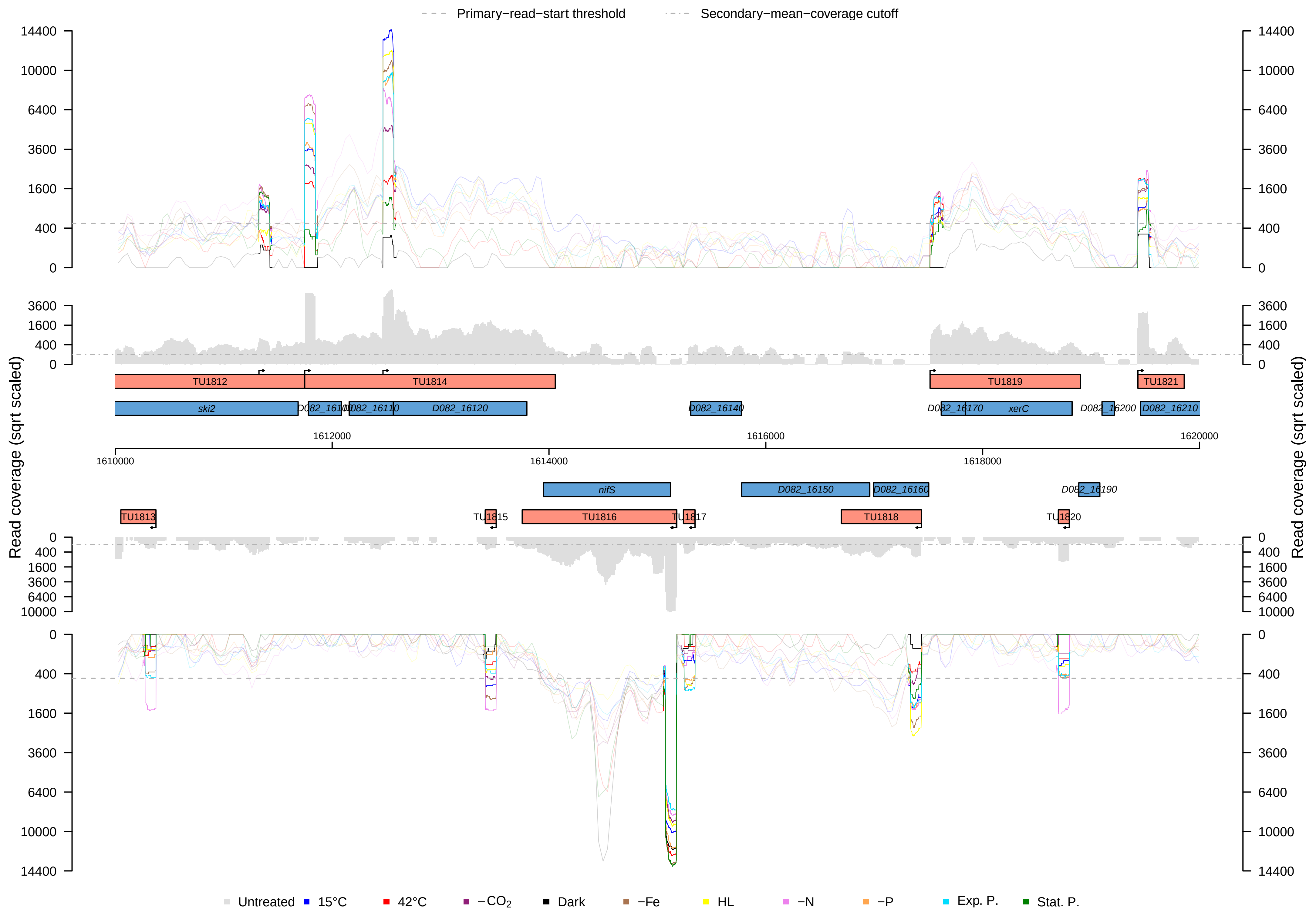

Primary−read−start threshold
Secondary−mean−coverage cutoff
Read coverage (sqrt scaled)
14400
10000
6400
3600
1600
400
0
TU1812
TU1814
TU1819
TU1821
ski2
D082_16100
D082_16110
D082_16120
D082_16140
D082_16170
xerC
D082_16200
D082_16210
1610000
1612000
1614000
1616000
1618000
1620000
nifS
D082_16150
D082_16160
D082_16190
TU1813
TU1815
TU1816
TU1817
TU1818
TU1820
Untreated
15°C
42°C
−CO2
Dark
−Fe
HL
−N
−P
Exp. P.
Stat. P.

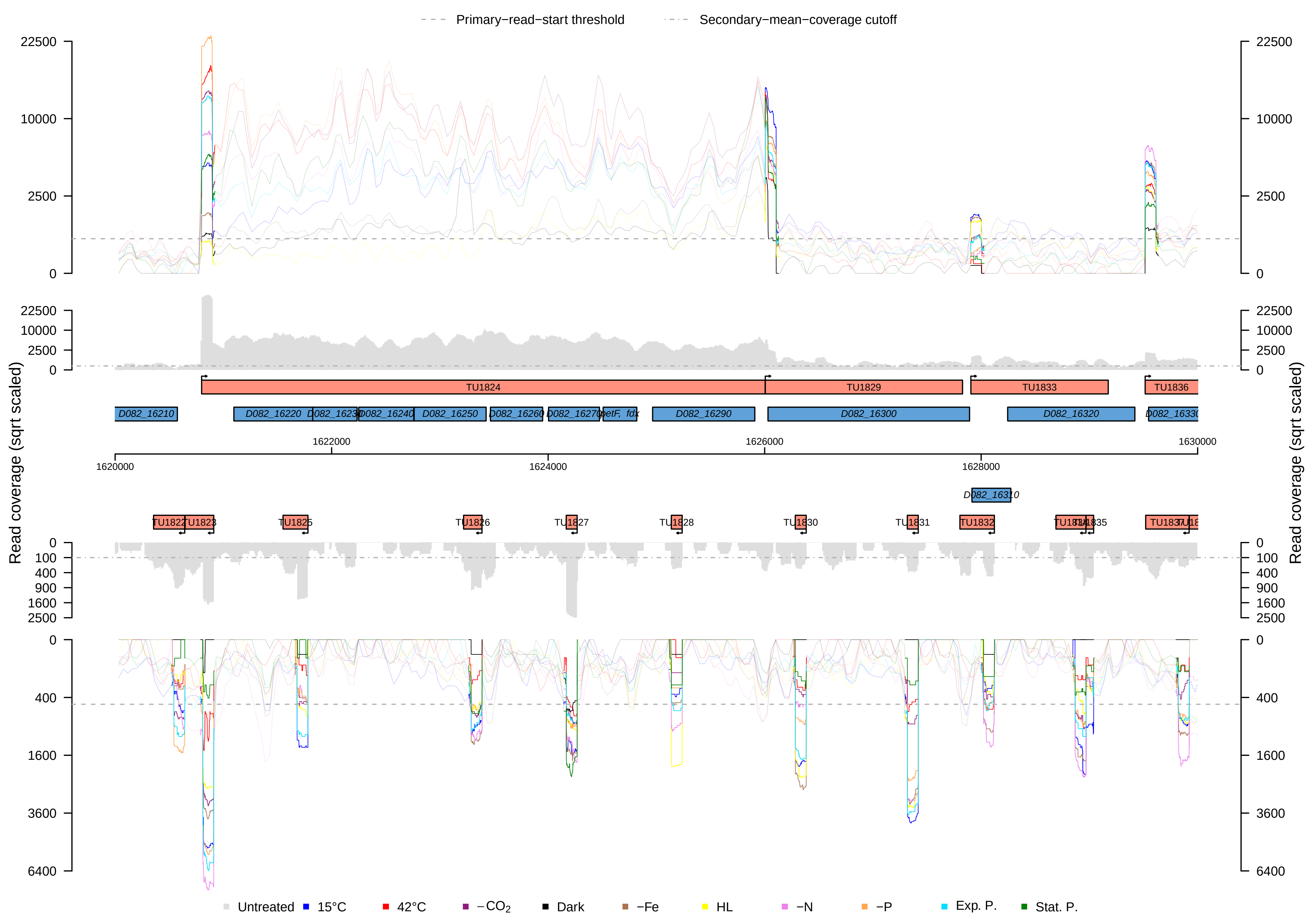

Primary−read−start threshold
Secondary−mean−coverage cutoff
Read coverage (sqrt scaled)
22500
10000
2500
0
TU1824
TU1829
TU1833
TU1836
D082_16210
D082_16220
D082_16230
D082_16240
D082_16250
D082_16260
D082_16270
petF, fdx
D082_16290
D082_16300
D082_16320
D082_16330
1620000
1622000
1624000
1626000
1628000
1630000
D082_16310
TU1822
TU1823
TU1825
TU1826
TU1827
TU1828
TU1830
TU1831
TU1832
TU1835
TU1837
100
400
900
1600
2500
3600
6400
Untreated
15°C
42°C
−CO_2
Dark
−Fe
HL
−N
−P
Exp. P.
Stat. P.

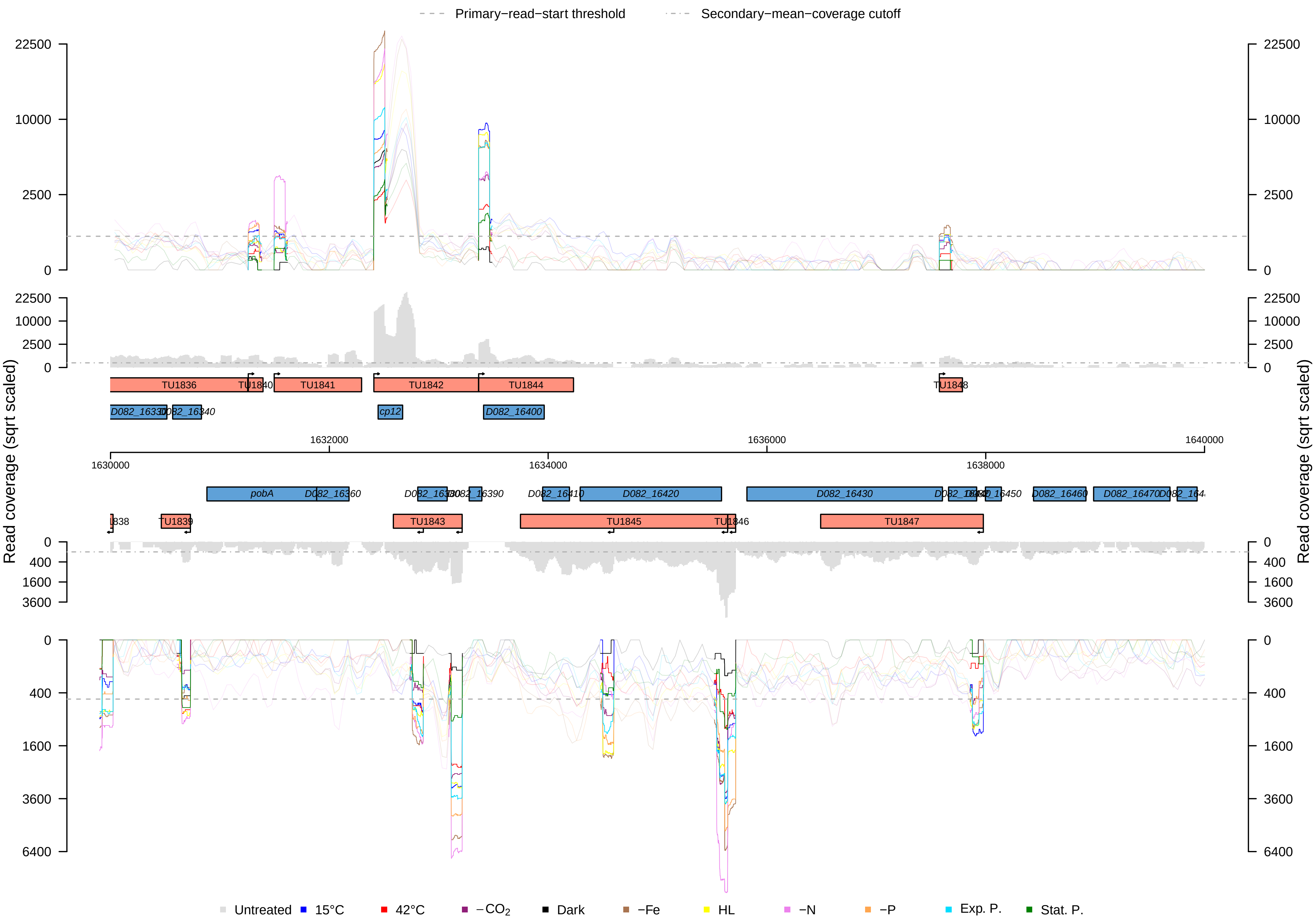

Primary−read−start threshold
Secondary−mean−coverage cutoff
Read coverage (sqrt scaled)
22500
10000
2500
0
400
1600
3600
6400
TU1836
TU1840
TU1841
TU1842
TU1844
TU1848
D082_16330
D082_16340
cp12
D082_16400
1630000
1632000
1634000
1636000
1638000
1640000
pobA
D082_16360
D082_16410
D082_16420
D082_16430
D082_16450
D082_16460
D082_16470
TU1839
TU1843
TU1845
TU1846
TU1847
Untreated
15°C
42°C
−CO2
Dark
−Fe
HL
−N
−P
Exp. P.
Stat. P.

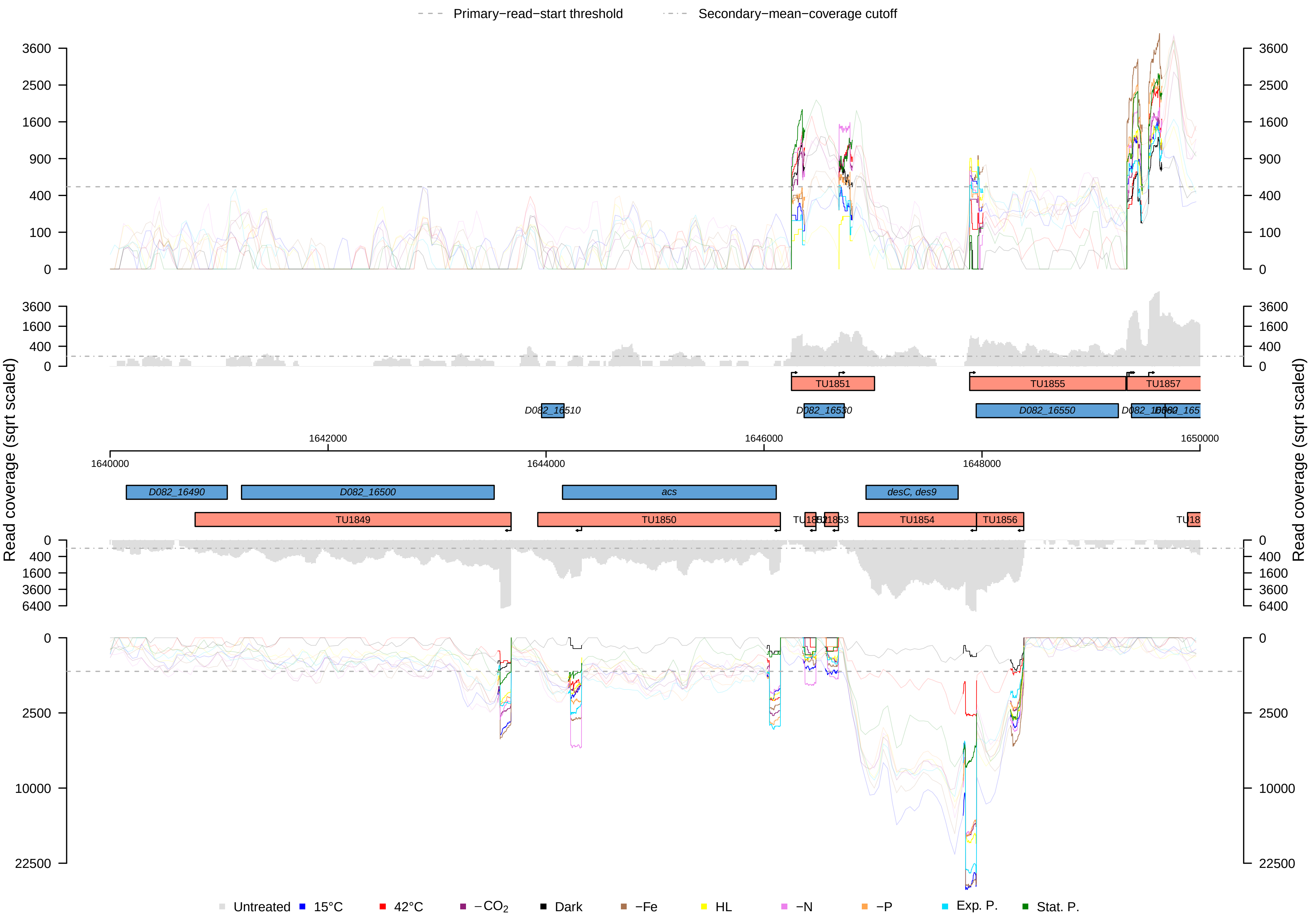

Primary−read−start threshold
Secondary−mean−coverage cutoff
Read coverage (sqrt scaled)
TU1851
TU1855
TU1857
D082_16510
D082_16530
D082_16550
1640000
1642000
1644000
1646000
1648000
1650000
D082_16490
D082_16500
acs
desC, des9
TU1849
TU1850
TU1853
TU1854
TU1856
Untreated
15°C
42°C
−CO2
Dark
−Fe
HL
−N
−P
Exp. P.
Stat. P.

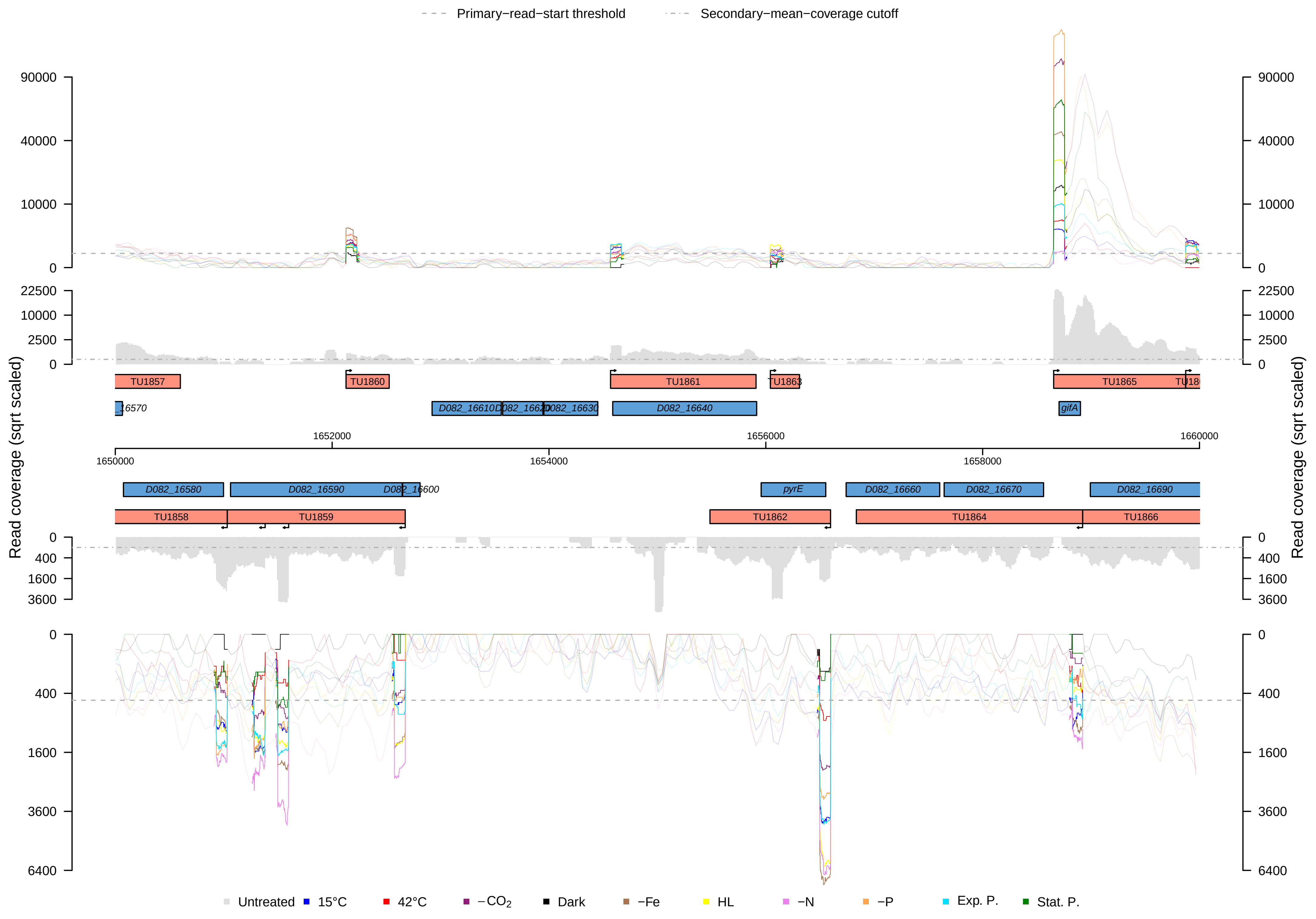

Primary−read−start threshold
Secondary−mean−coverage cutoff
90000
40000
10000
0
22500
10000
2500
0
TU1857
TU1860
TU1861
TU1863
TU1865
_16570
D082_16610
D082_16630
D082_16640
gifA
1650000
1652000
1654000
1656000
1658000
1660000
D082_16580
D082_16590
pyrE
D082_16660
D082_16670
D082_16690
TU1858
TU1859
TU1862
TU1864
TU1866
400
1600
3600
6400
Read coverage (sqrt scaled)
Untreated
15°C
42°C
−CO2
Dark
−Fe
HL
−N
−P
Exp. P.
Stat. P.

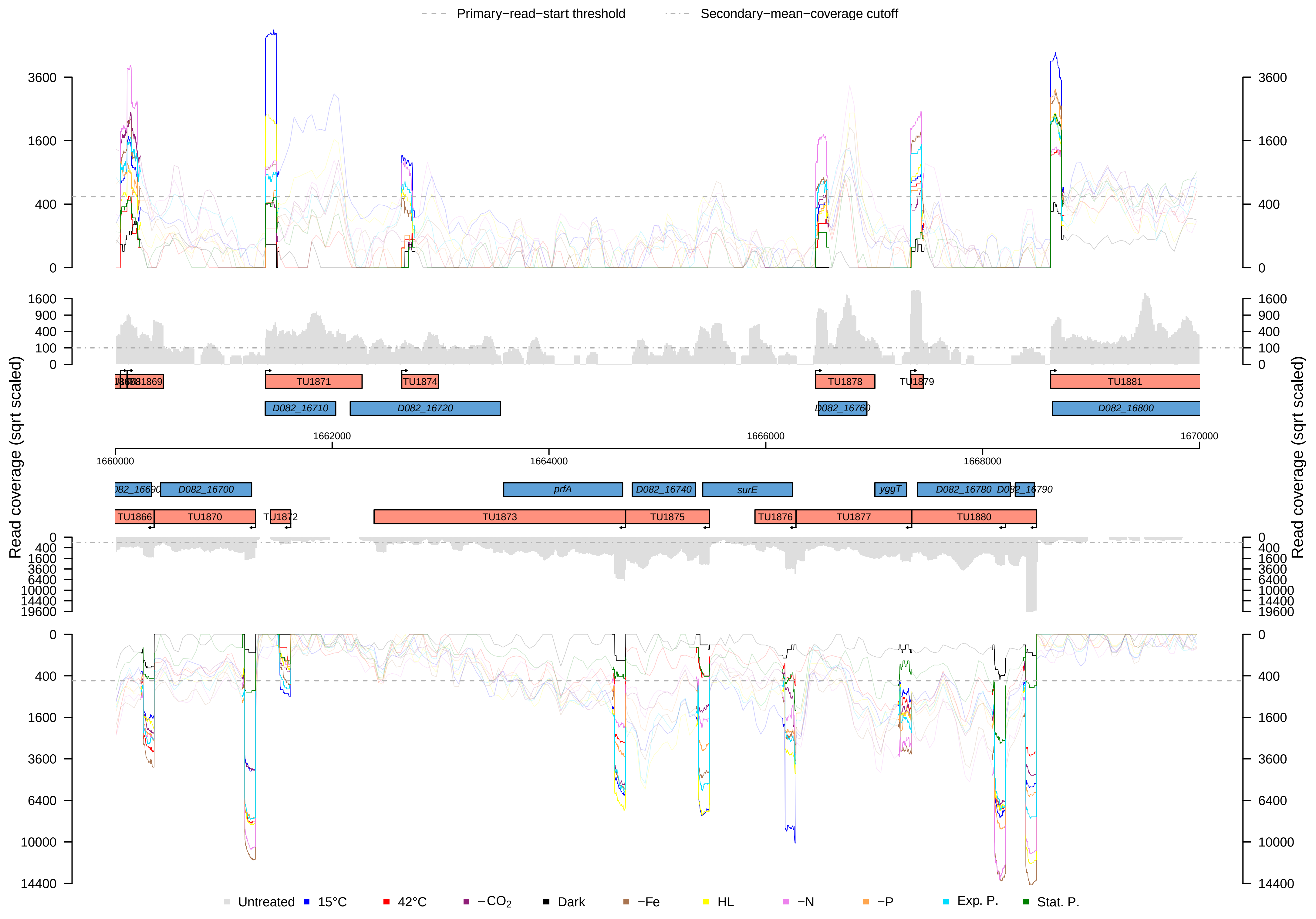

Primary−read−start threshold
Secondary−mean−coverage cutoff
Read coverage (sqrt scaled)
3600
1600
400
0
900
100
14400
10000
6400
19600
TU1871
TU1874
TU1878
TU1879
TU1881
D082_16710
D082_16720
D082_16760
D082_16800
1660000
1662000
1664000
1666000
1668000
1670000
prfA
D082_16740
surE
yggT
D082_16780
D082_16790
D082_16700
TU1866
TU1870
TU1872
TU1873
TU1875
TU1876
TU1877
TU1880
Untreated
15°C
42°C
$-CO_2$
Dark
−Fe
HL
−N
−P
Exp. P.
Stat. P.

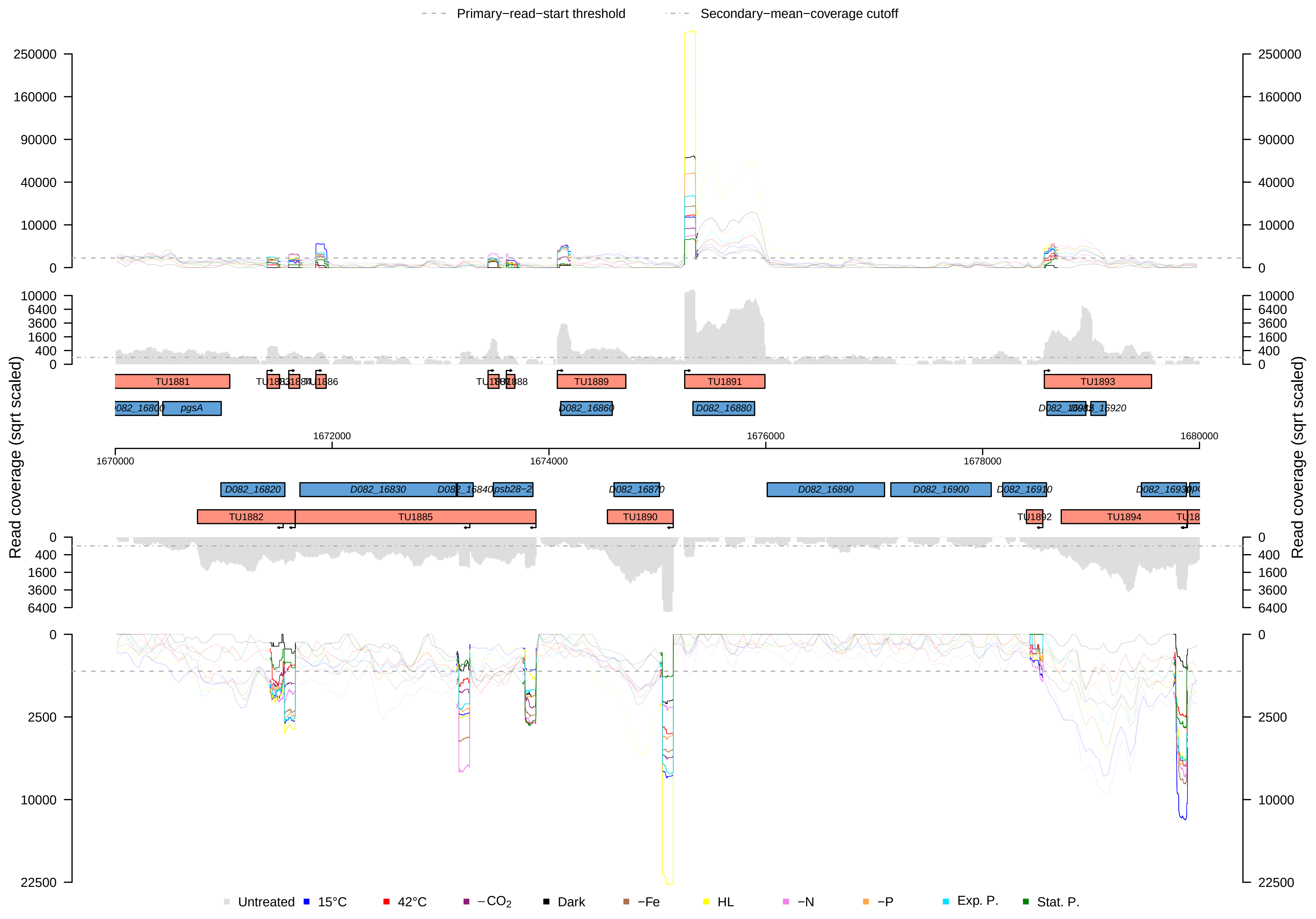

Primary−read−start threshold
Secondary−mean−coverage cutoff
Read coverage (sqrt scaled)
250000
160000
90000
40000
10000
0
10000
6400
3600
1600
400
TU1881
TU1886
TU1889
TU1891
TU1893
pgsA
D082_16860
D082_16880
1670000
1672000
1674000
1676000
1678000
1680000
D082_16820
D082_16830
psb28−2
D082_16870
D082_16890
D082_16900
D082_16910
TU1882
TU1885
TU1890
TU1894
2500
22500
Untreated
15°C
42°C
−CO2
Dark
−Fe
HL
−N
−P
Exp. P.
Stat. P.

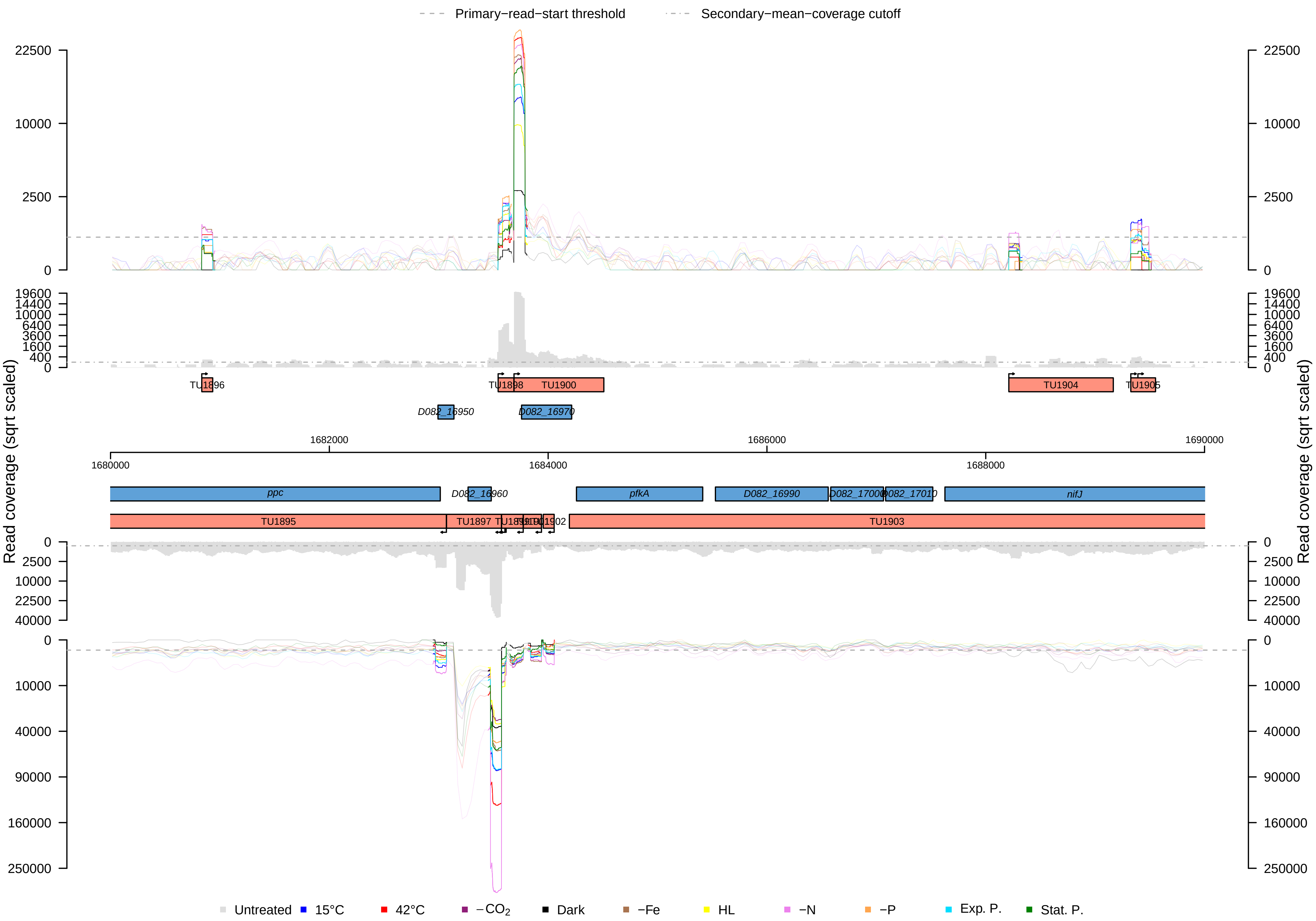

Primary−read−start threshold
Secondary−mean−coverage cutoff
Read coverage (sqrt scaled)
22500
10000
2500
0
19600
14400
10000
6400
3600
1600
400
0
TU1896
TU1898
TU1900
TU1904
TU1905
D082_16950
D082_16970
1680000
1682000
1684000
1686000
1688000
1690000
ppc
D082_16960
pfkA
D082_16990
D082_17000
D082_17010
nifJ
TU1895
TU1897
TU1902
TU1903
2500
10000
22500
40000
90000
160000
250000
Untreated
15°C
42°C
−CO2
Dark
−Fe
HL
−N
−P
Exp. P.
Stat. P.

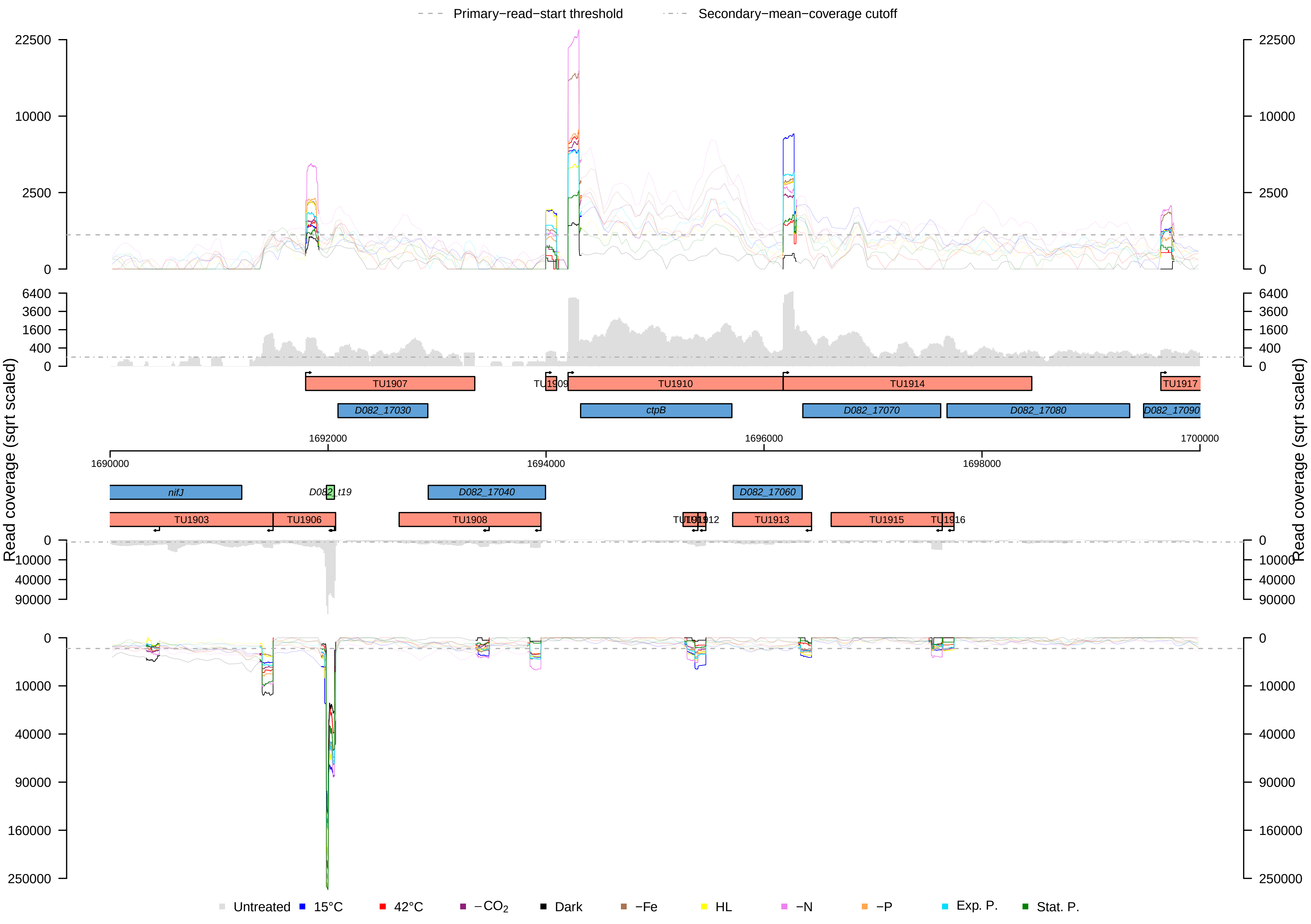

Primary−read−start threshold
Secondary−mean−coverage cutoff
Read coverage (sqrt scaled)
22500
10000
2500
0
6400
3600
1600
400
40000
90000
160000
250000
TU1907
TU1909
TU1910
TU1914
TU1917
D082_17030
ctpB
D082_17070
D082_17080
D082_17090
1690000
1692000
1694000
1696000
1698000
1700000
nifJ
D082_t19
D082_17040
D082_17060
TU1903
TU1906
TU1908
TU1912
TU1913
TU1915
TU1916
Untreated
15°C
42°C
−CO2
Dark
−Fe
HL
−N
−P
Exp. P.
Stat. P.

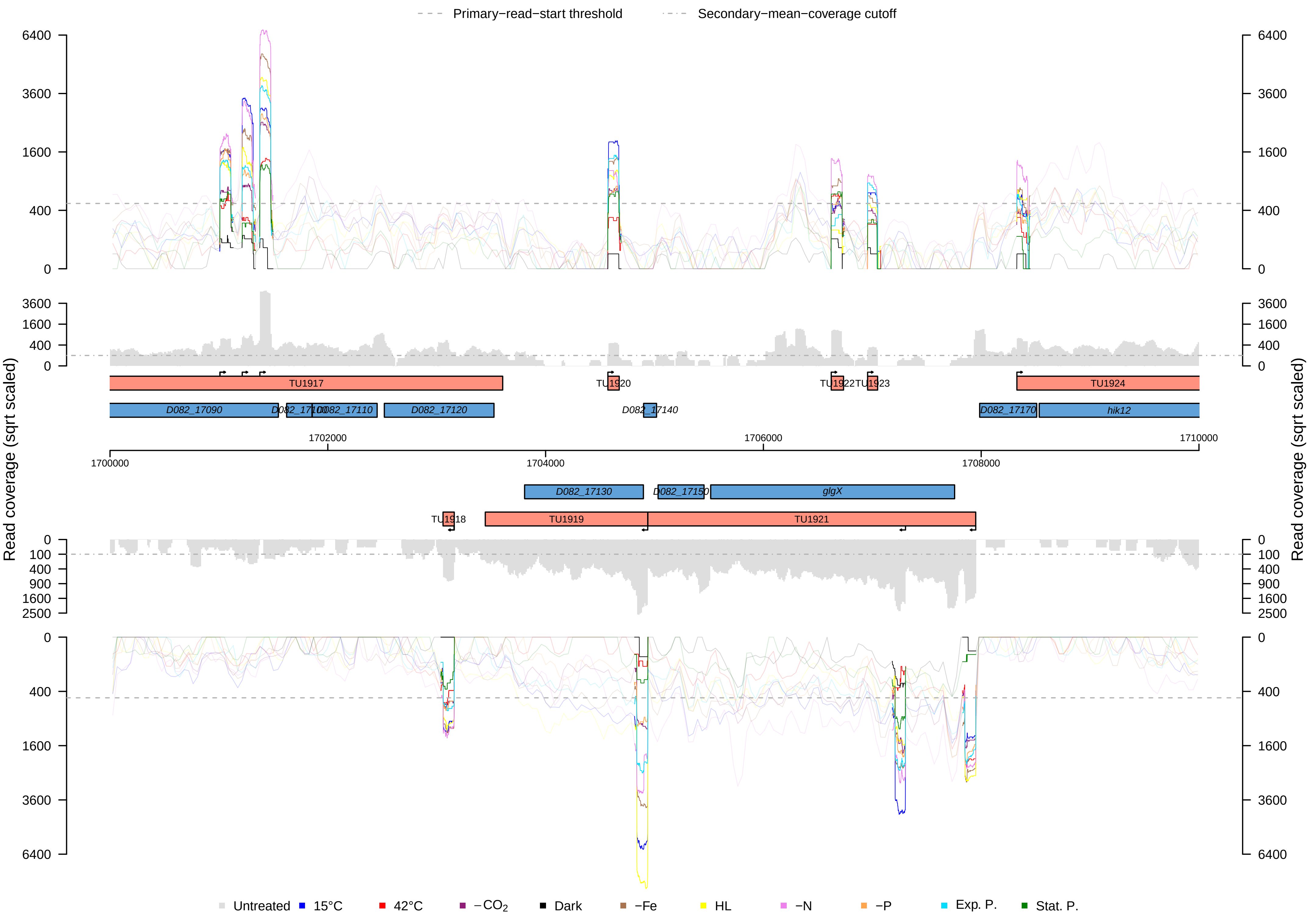

Primary−read−start threshold
Secondary−mean−coverage cutoff
6400
3600
1600
400
0
3600
1600
400
0
TU1917
TU1920
TU1922
TU1923
TU1924
D082_17090
D082_17100
D082_17110
D082_17120
D082_17140
D082_17170
hik12
1700000
1702000
1704000
1706000
1708000
1710000
D082_17130
D082_17150
glgX
TU1918
TU1919
TU1921
0
100
400
900
1600
2500
0
400
1600
3600
6400
Read coverage (sqrt scaled)
Untreated
15°C
42°C
−CO2
Dark
−Fe
HL
−N
−P
Exp. P.
Stat. P.

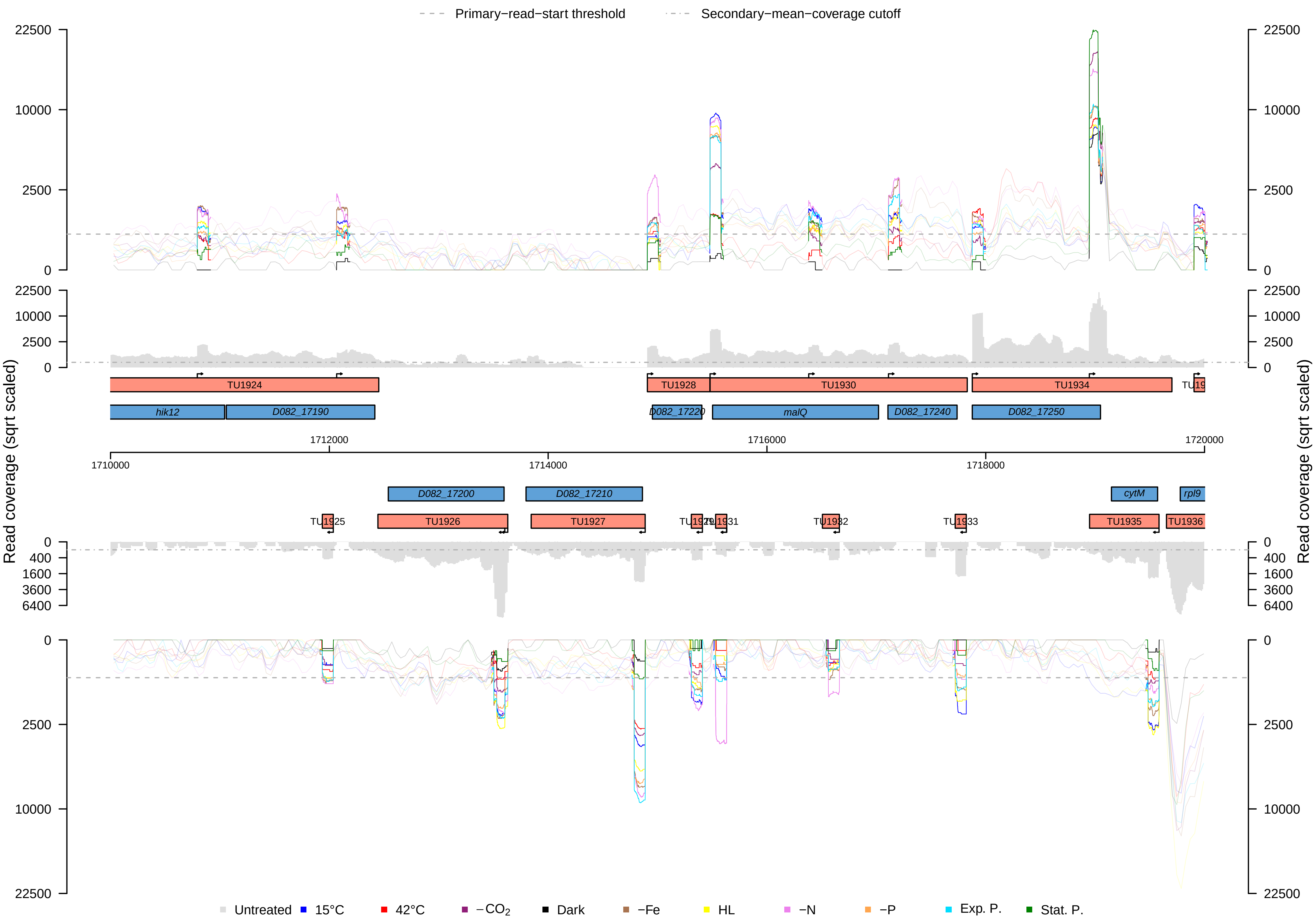

Primary−read−start threshold
Secondary−mean−coverage cutoff
Read coverage (sqrt scaled)
22500
10000
2500
0
400
1600
3600
6400
TU1924
TU1928
TU1930
TU1934
hik12
D082_17190
D082_17220
malQ
D082_17240
D082_17250
1710000
1712000
1714000
1716000
1718000
1720000
D082_17200
D082_17210
cytM
rpl9
TU1925
TU1926
TU1927
TU1932
TU1933
TU1935
TU1936
Untreated
15°C
42°C
−CO2
Dark
−Fe
HL
−N
−P
Exp. P.
Stat. P.

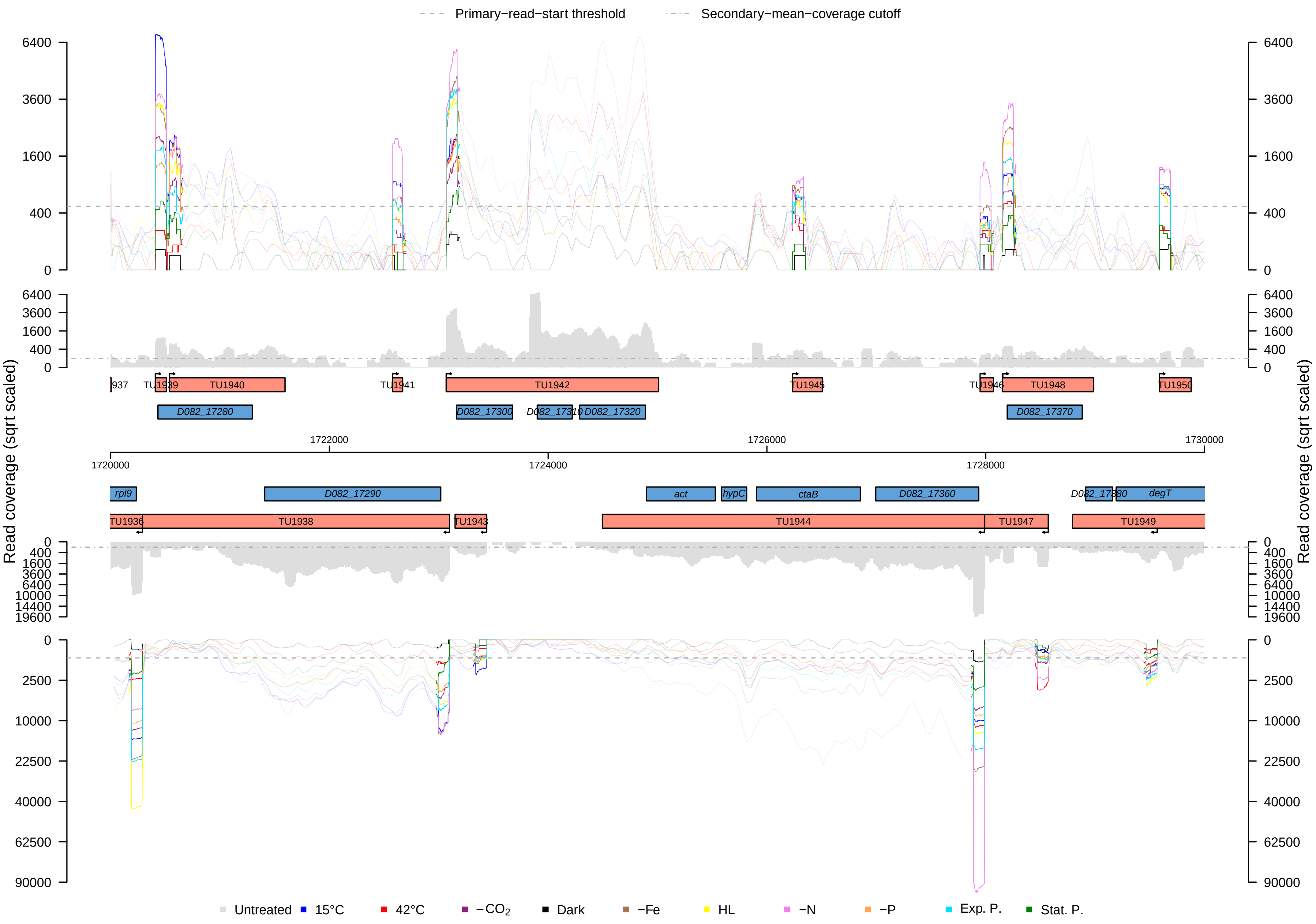

Primary−read−start threshold
Secondary−mean−coverage cutoff
Read coverage (sqrt scaled)
6400
3600
1600
400
0
TU1940
TU1941
TU1942
TU1945
TU1946
TU1948
TU1950
D082_17280
D082_17300
D082_17310
D082_17320
D082_17370
1720000
1722000
1724000
1726000
1728000
1730000
rpl9
D082_17290
act
hypC
ctaB
D082_17360
degT
TU1936
TU1938
TU1943
TU1944
TU1947
TU1949
10000
14400
19600
2500
22500
40000
62500
90000
Untreated
15°C
42°C
−CO2
Dark
−Fe
HL
−N
−P
Exp. P.
Stat. P.

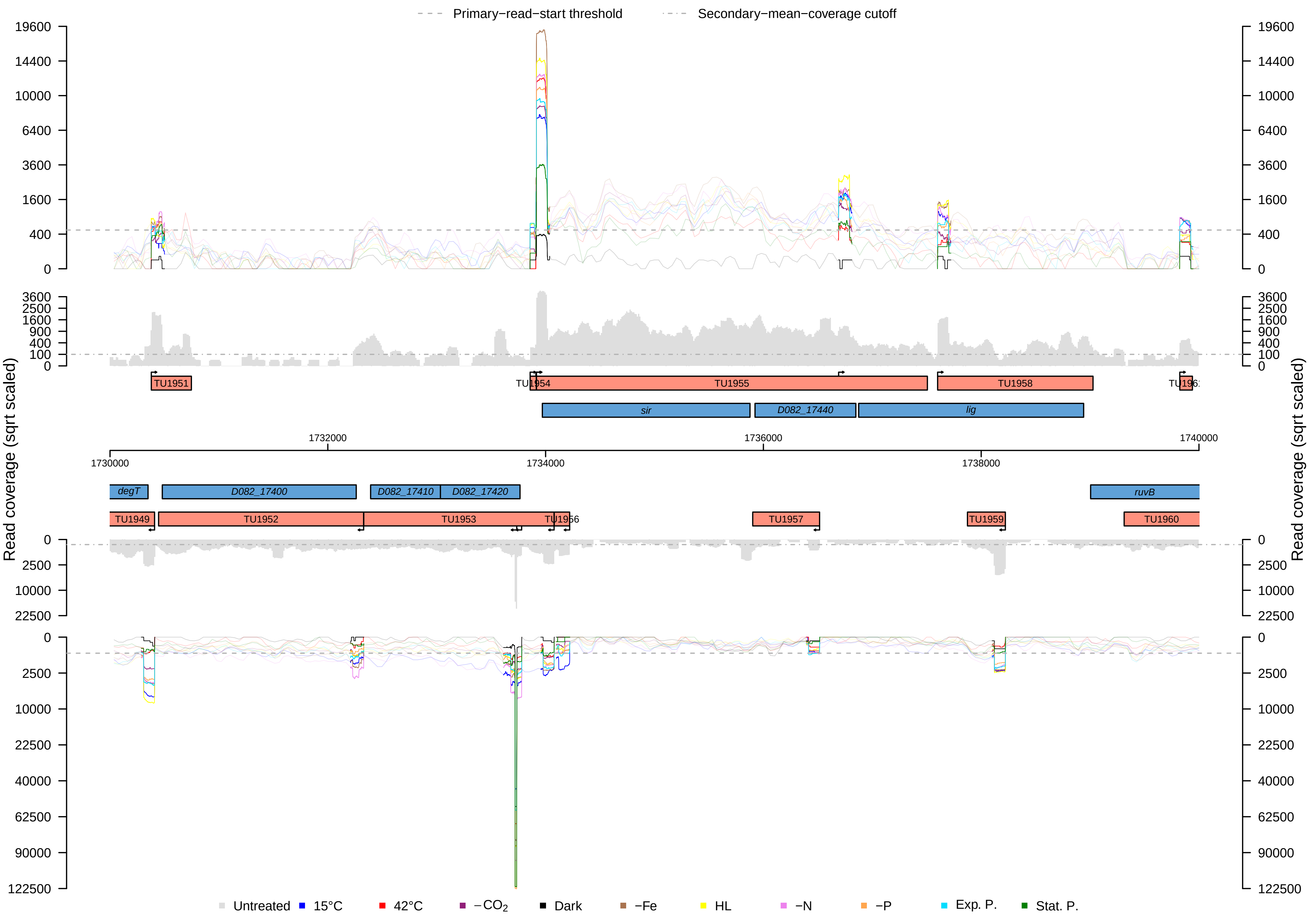

Primary−read−start threshold
Secondary−mean−coverage cutoff
Read coverage (sqrt scaled)
19600
14400
10000
6400
3600
1600
400
0
2500
900
100
TU1951
TU1954
TU1955
TU1958
sir
D082_17440
lig
1730000
1732000
1734000
1736000
1738000
1740000
degT
D082_17400
D082_17410
D082_17420
ruvB
TU1949
TU1952
TU1953
TU1956
TU1957
TU1959
TU1960
22500
40000
62500
90000
122500
Untreated
15°C
42°C
−CO2
Dark
−Fe
HL
−N
−P
Exp. P.
Stat. P.

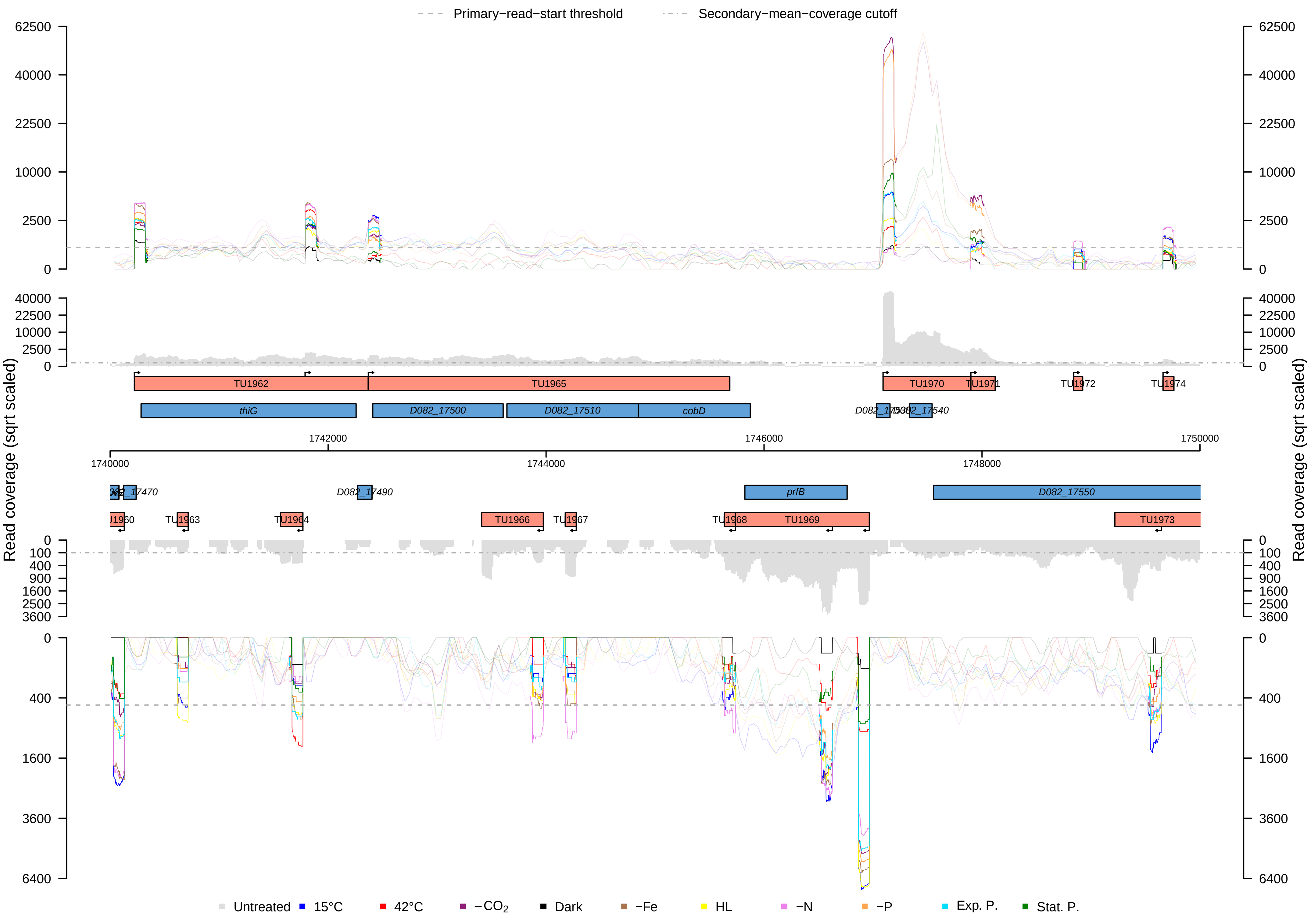

Primary−read−start threshold
Secondary−mean−coverage cutoff
Read coverage (sqrt scaled)
62500
40000
22500
10000
2500
0
TU1962
TU1965
TU1970
TU1971
TU1972
TU1974
thiG
D082_17500
D082_17510
cobD
1742000
1746000
1750000
1740000
1744000
1748000
D082_17490
prfB
D082_17550
TU1963
TU1964
TU1966
TU1967
TU1968
TU1969
TU1973
100
400
900
1600
2500
3600
6400
Untreated
15°C
42°C
−CO2
Dark
−Fe
HL
−N
−P
Exp. P.
Stat. P.

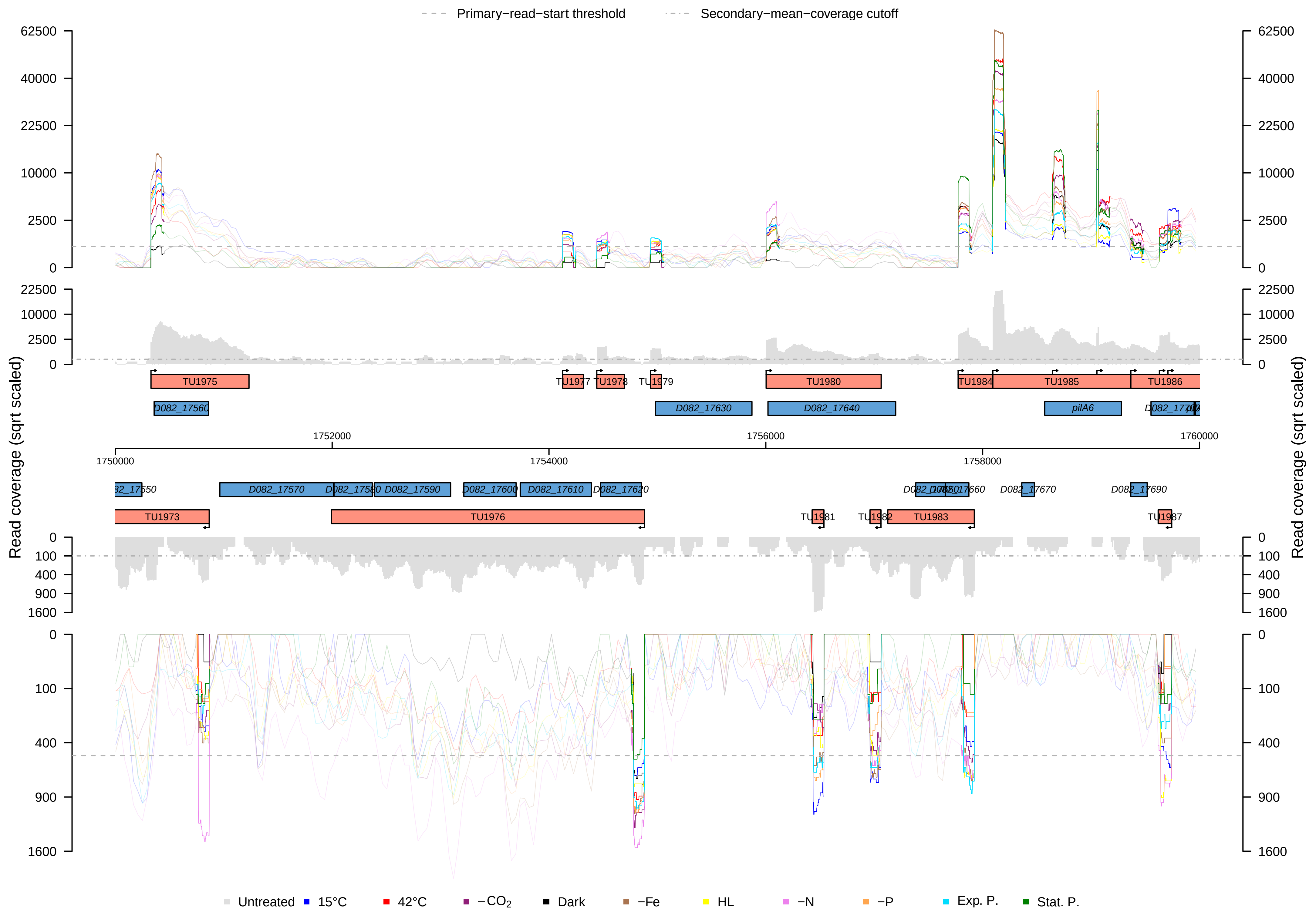

Primary−read−start threshold
Secondary−mean−coverage cutoff
Read coverage (sqrt scaled)
62500
40000
22500
10000
2500
0
1600
900
400
100
TU1975
TU1977
TU1978
TU1979
TU1980
TU1984
TU1985
TU1986
D082_17560
D082_17630
D082_17640
pilA6
1750000
1752000
1754000
1756000
1758000
1760000
D082_17570
D082_17590
D082_17600
D082_17610
D082_17620
D082_17670
D082_17690
TU1973
TU1976
TU1981
TU1982
TU1983
TU1987
Untreated
15°C
42°C
−CO_2
Dark
−Fe
HL
−N
−P
Exp. P.
Stat. P.

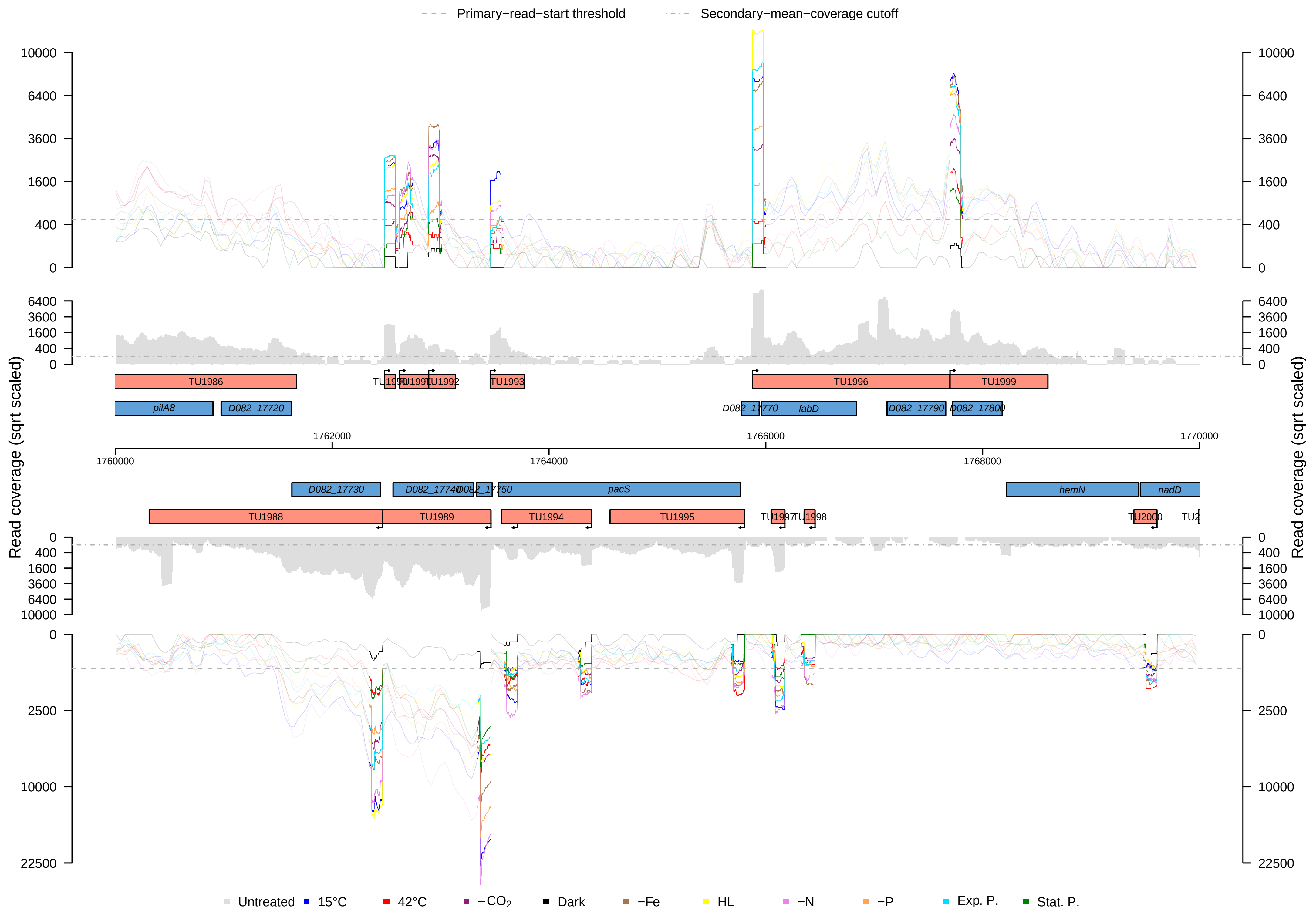

Primary−read−start threshold
Secondary−mean−coverage cutoff
Read coverage (sqrt scaled)
10000
6400
3600
1600
400
0
TU1986
TU1990
TU1991
TU1992
TU1993
TU1996
TU1999
pilA8
D082_17720
D082_17770
fabD
D082_17790
D082_17800
1760000
1762000
1764000
1766000
1768000
1770000
D082_17730
D082_17740
D082_17750
pacS
hemN
nadD
TU1988
TU1989
TU1994
TU1995
TU1997
TU1998
TU2000
2500
10000
22500
Untreated
15°C
42°C
−CO2
Dark
−Fe
HL
−N
−P
Exp. P.
Stat. P.

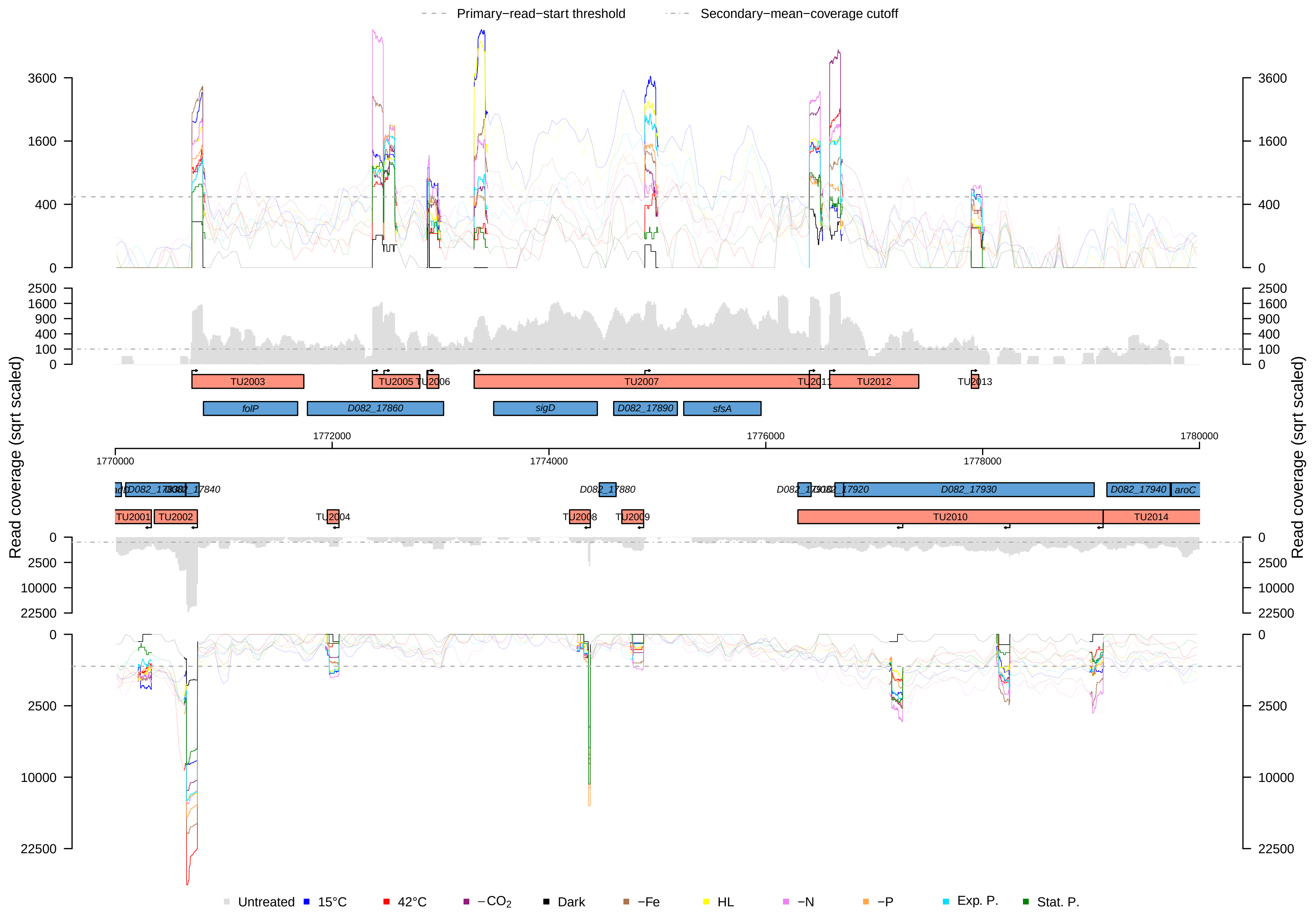

Primary−read−start threshold
Secondary−mean−coverage cutoff
Read coverage (sqrt scaled)
3600
1600
400
0
2500
1600
900
400
100
0
TU2003
TU2005
TU2006
TU2007
TU2011
TU2012
TU2013
folP
D082_17860
sigD
D082_17890
sfsA
1770000
1772000
1774000
1776000
1778000
1780000
D082_17840
D082_17880
D082_17920
D082_17930
D082_17940
aroC
TU2001
TU2002
TU2004
TU2008
TU2009
TU2010
TU2014
10000
22500
Untreated
15°C
42°C
−CO2
Dark
−Fe
HL
−N
−P
Exp. P.
Stat. P.

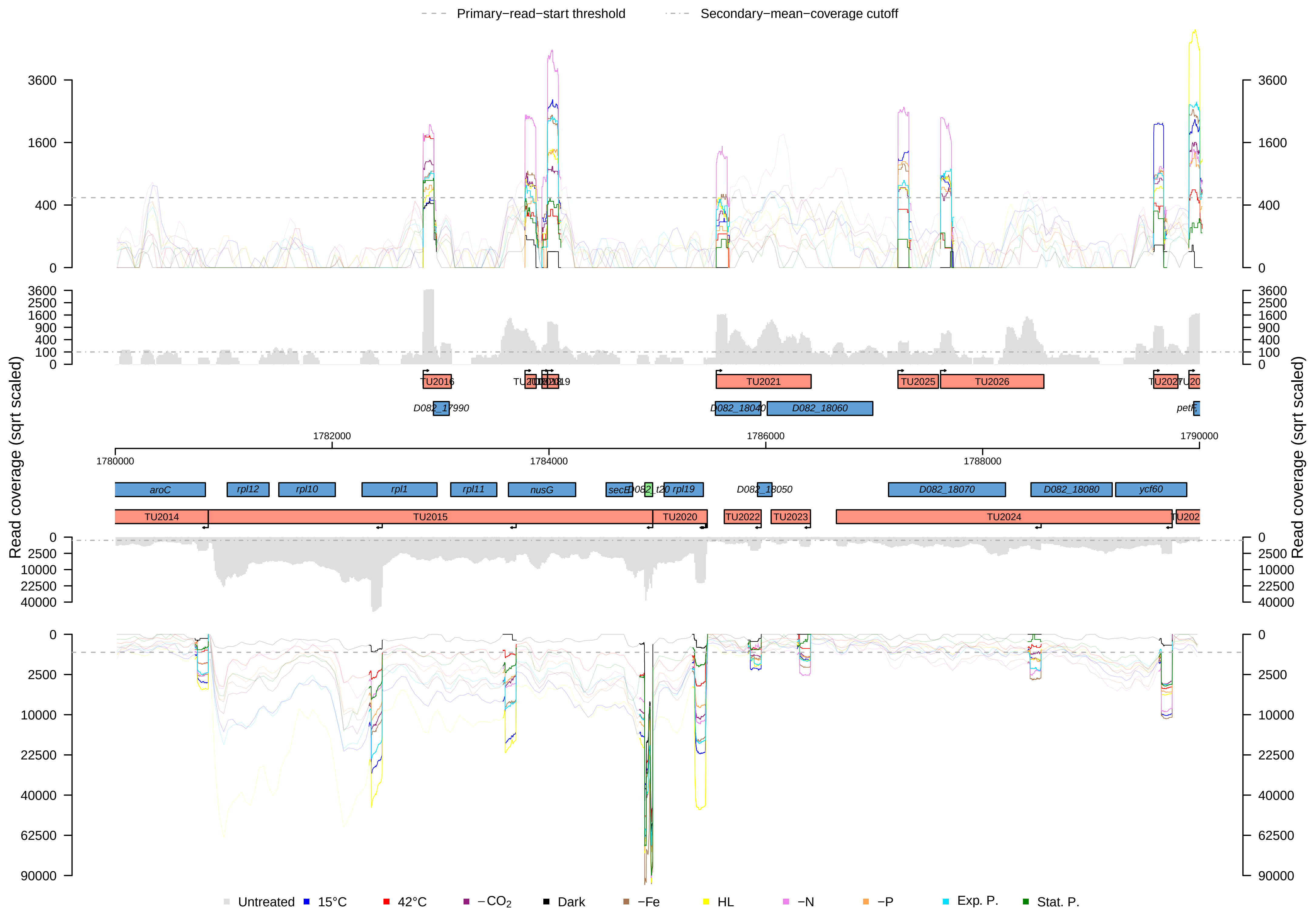

Primary−read−start threshold
Secondary−mean−coverage cutoff
Read coverage (sqrt scaled)
3600
1600
400
0
2500
900
100
10000
22500
40000
62500
90000
TU2016
TU2021
TU2025
TU2026
D082_17990
D082_18040
D082_18060
petF
1780000
1782000
1784000
1786000
1788000
1790000
aroC
rpl12
rpl10
rpl1
rpl11
nusG
secE
rpl19
D082_18050
D082_18070
D082_18080
ycf60
TU2014
TU2015
TU2020
TU2022
TU2023
TU2024
Untreated
15°C
42°C
−CO2
Dark
−Fe
HL
−N
−P
Exp. P.
Stat. P.

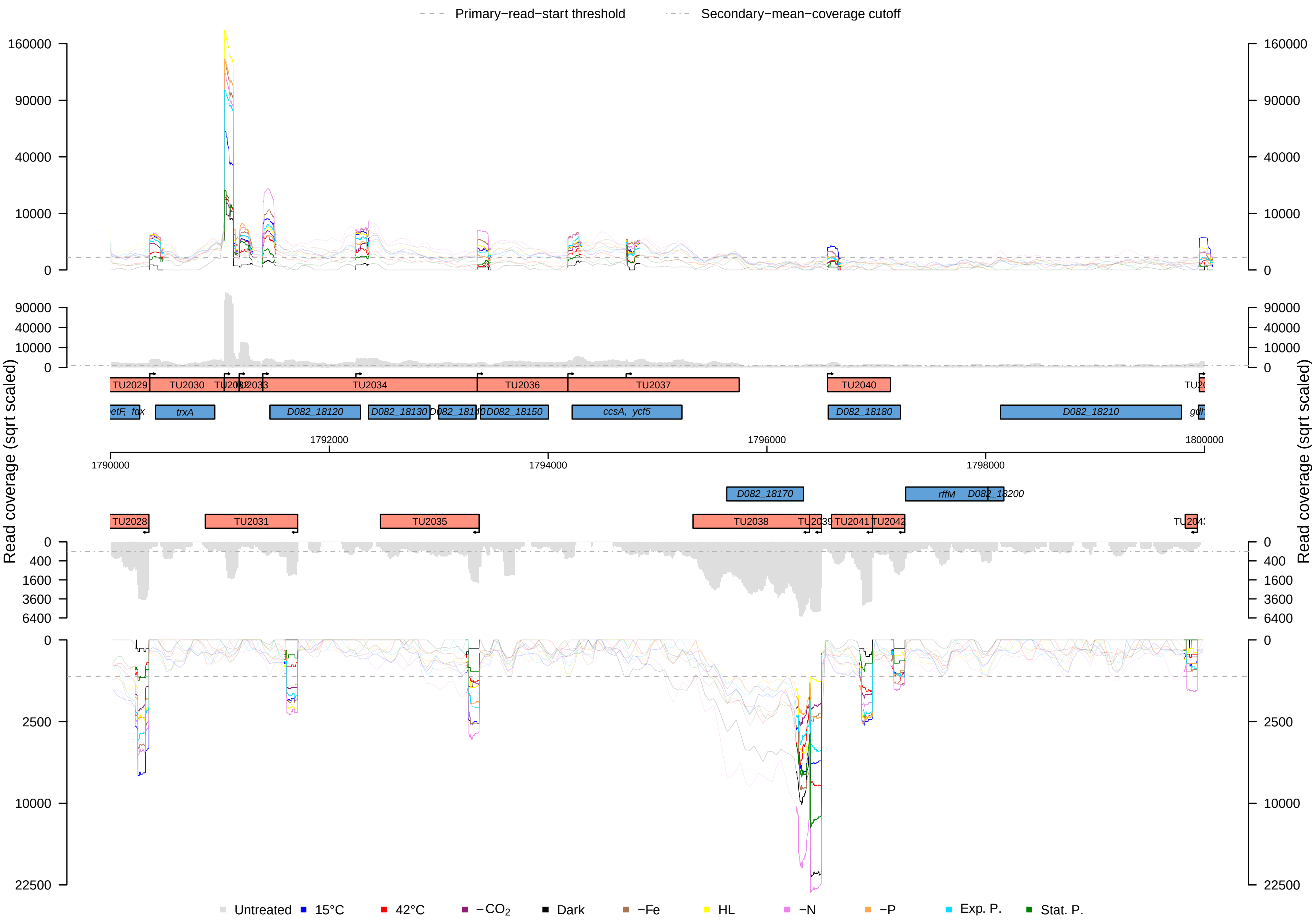

Primary−read−start threshold
Secondary−mean−coverage cutoff
160000
90000
40000
10000
0
90000
40000
10000
0
Read coverage (sqrt scaled)
TU2029
TU2030
TU2034
TU2036
TU2037
TU2040
trxA
D082_18120
D082_18130
D082_18150
ccsA, ycf5
D082_18180
D082_18210
1792000
1796000
1800000
1790000
1794000
1798000
D082_18170
rffM
TU2028
TU2031
TU2035
TU2038
TU2041
TU2042
0
400
1600
3600
6400
0
2500
10000
22500
Untreated
15°C
42°C
−CO2
Dark
−Fe
HL
−N
−P
Exp. P.
Stat. P.

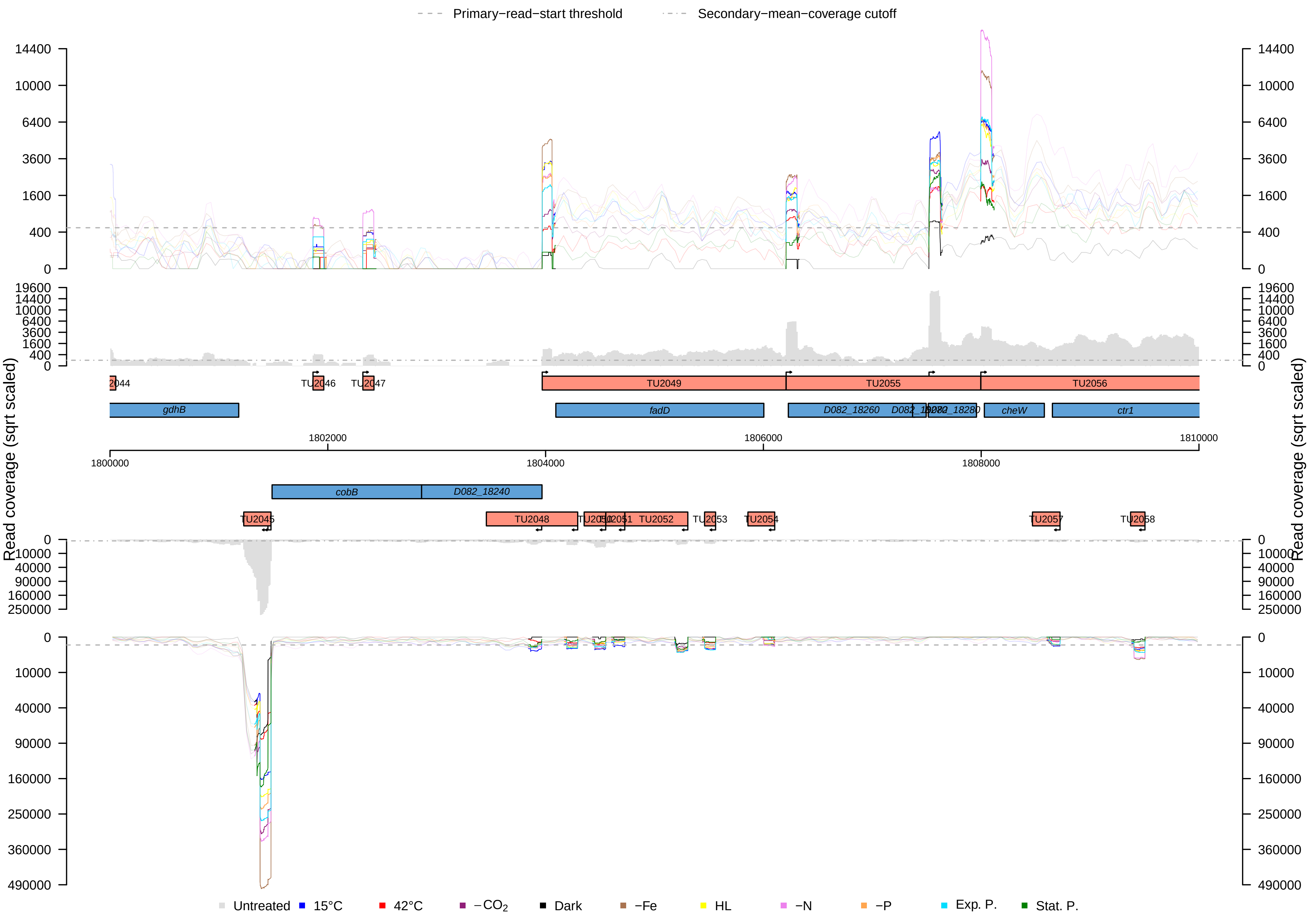

Primary−read−start threshold
Secondary−mean−coverage cutoff
Read coverage (sqrt scaled)
14400
10000
6400
3600
1600
400
0
19600
TU2049
TU2055
TU2056
TU2046
TU2047
gdhB
fadD
D082_18260
cheW
ctr1
1800000
1802000
1804000
1806000
1808000
1810000
cobB
D082_18240
TU2045
TU2048
TU2052
TU2053
TU2054
TU2057
TU2058
40000
90000
160000
250000
360000
490000
Untreated
15°C
42°C
−CO2
Dark
−Fe
HL
−N
−P
Exp. P.
Stat. P.

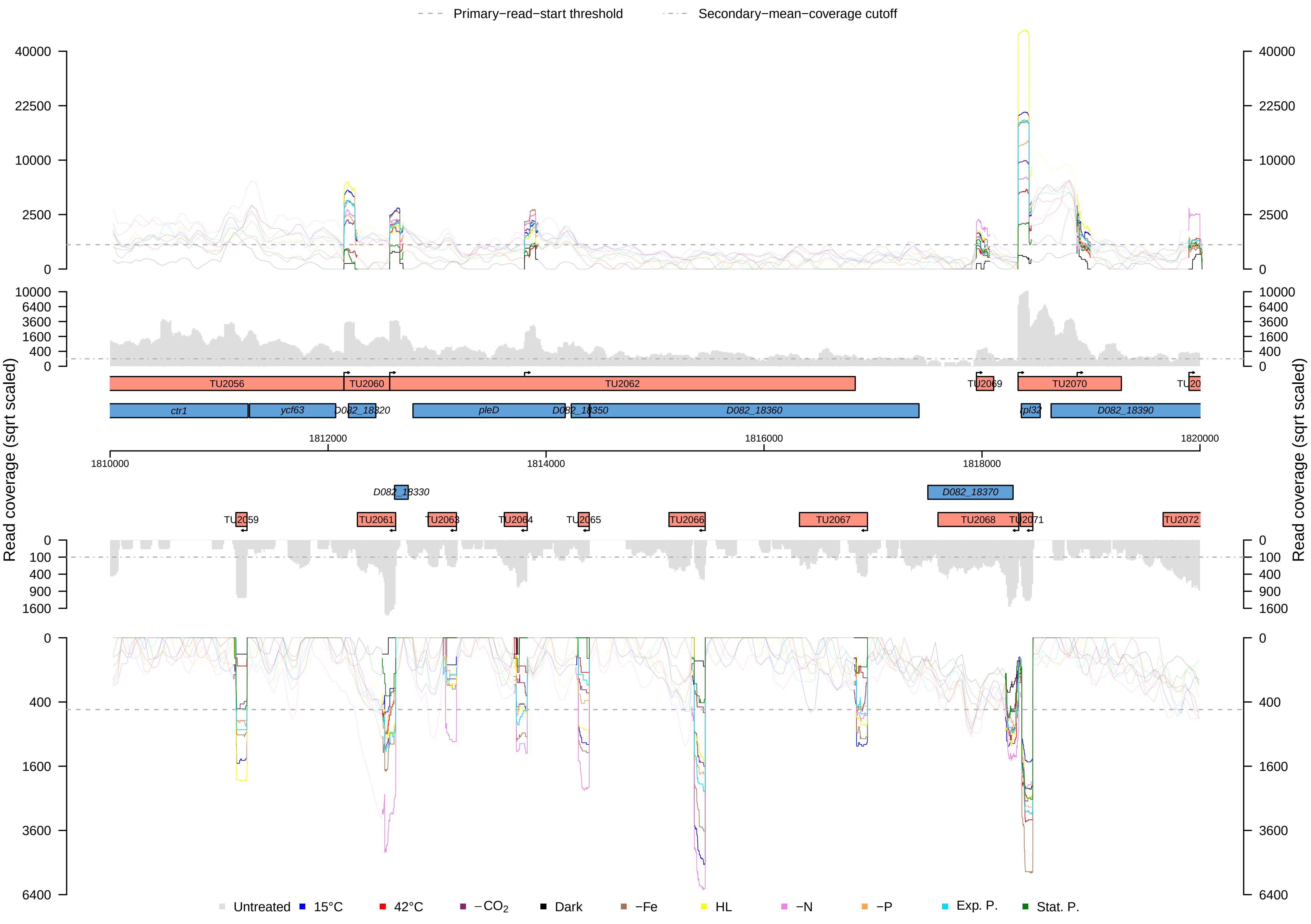

Primary−read−start threshold
Secondary−mean−coverage cutoff
Read coverage (sqrt scaled)
40000
22500
10000
2500
0
10000
6400
3600
1600
400
100
900
TU2056
TU2060
TU2062
TU2069
TU2070
ctr1
ycf63
D082_18320
pleD
D082_18350
D082_18360
rpl32
D082_18390
1810000
1812000
1814000
1816000
1818000
1820000
D082_18330
D082_18370
TU2059
TU2061
TU2063
TU2064
TU2065
TU2066
TU2067
TU2068
TU2071
TU2072
Untreated
15°C
42°C
−CO2
Dark
−Fe
HL
−N
−P
Exp. P.
Stat. P.

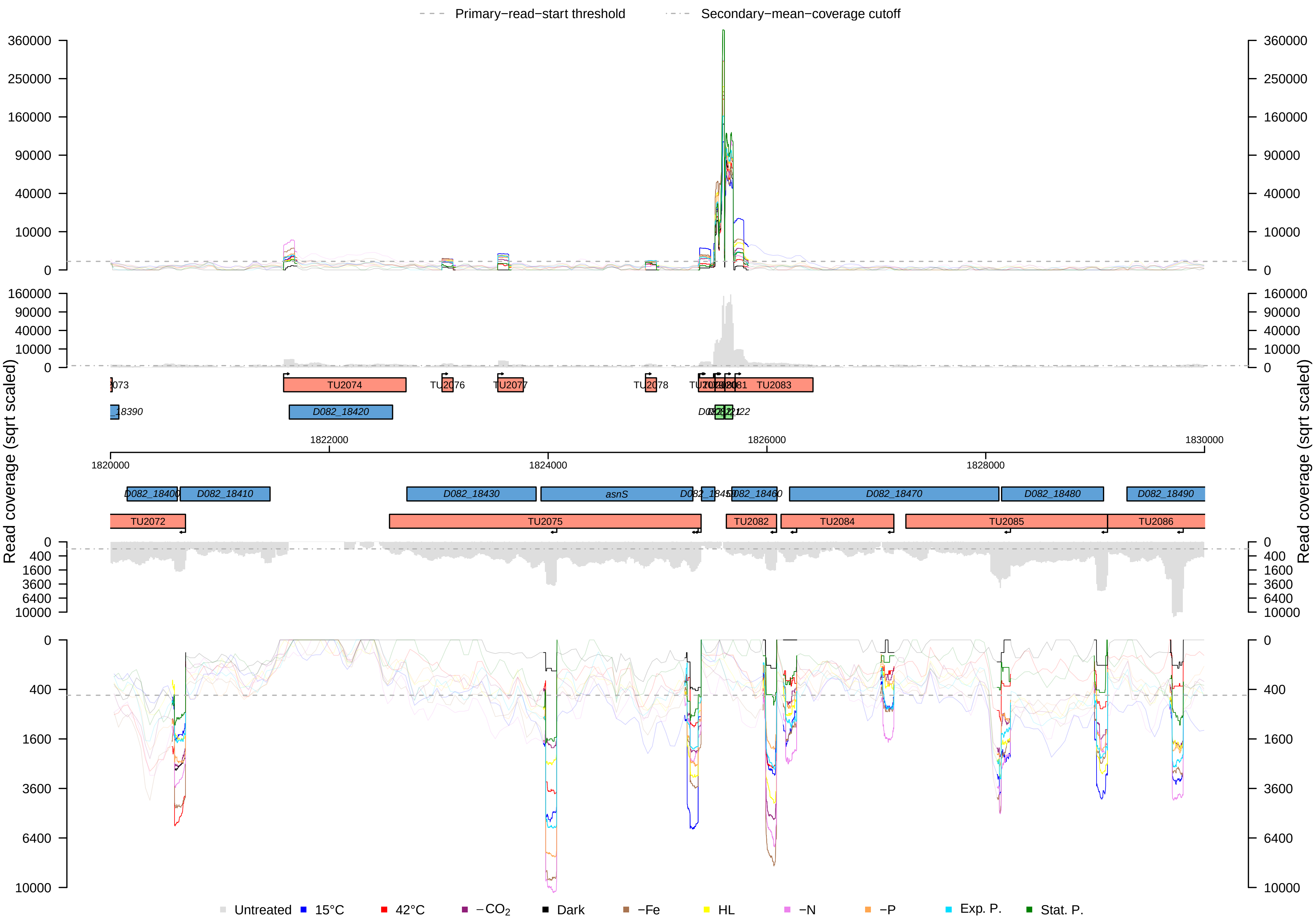

Primary−read−start threshold
Secondary−mean−coverage cutoff
Read coverage (sqrt scaled)
360000
250000
160000
90000
40000
10000
0
TU2074
TU2076
TU2077
TU2078
TU2083
D082_18420
1820000
1822000
1824000
1826000
1828000
1830000
D082_18400
D082_18410
D082_18430
asnS
D082_18460
D082_18470
D082_18480
D082_18490
TU2072
TU2075
TU2082
TU2084
TU2085
TU2086
400
1600
3600
6400
Untreated
15°C
42°C
−CO2
Dark
−Fe
HL
−N
−P
Exp. P.
Stat. P.

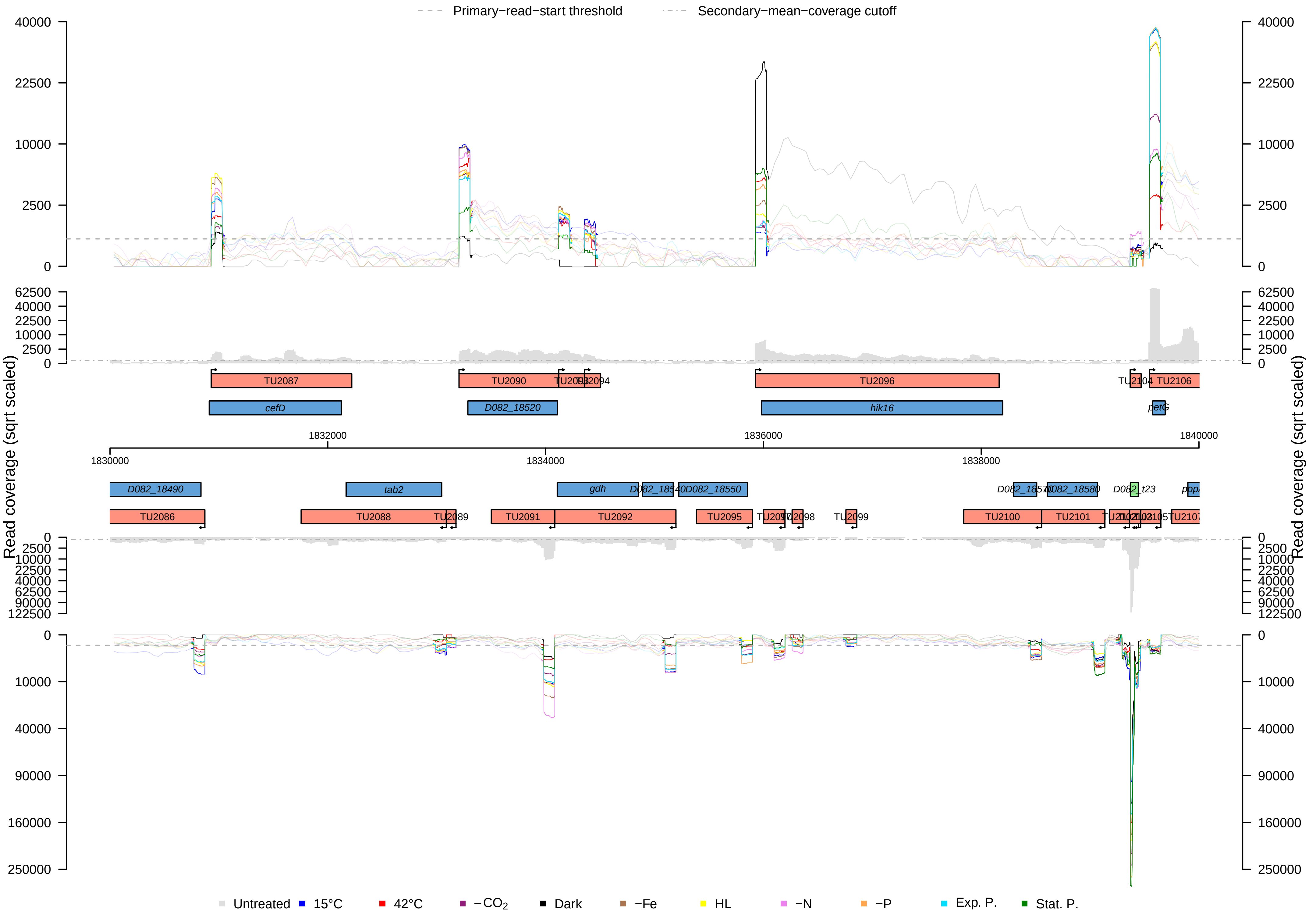

Primary−read−start threshold
Secondary−mean−coverage cutoff
Read coverage (sqrt scaled)
TU2087
TU2090
TU2093
TU2094
TU2096
TU2104
TU2106
cefD
D082_18520
hik16
petG
1830000
1832000
1834000
1836000
1838000
1840000
D082_18490
tab2
gdh
D082_18550
D082_18580
TU2086
TU2088
TU2089
TU2091
TU2092
TU2095
TU2098
TU2099
TU2100
TU2101
TU2107
Untreated
15°C
42°C
−CO_2
Dark
−Fe
HL
−N
−P
Exp. P.
Stat. P.

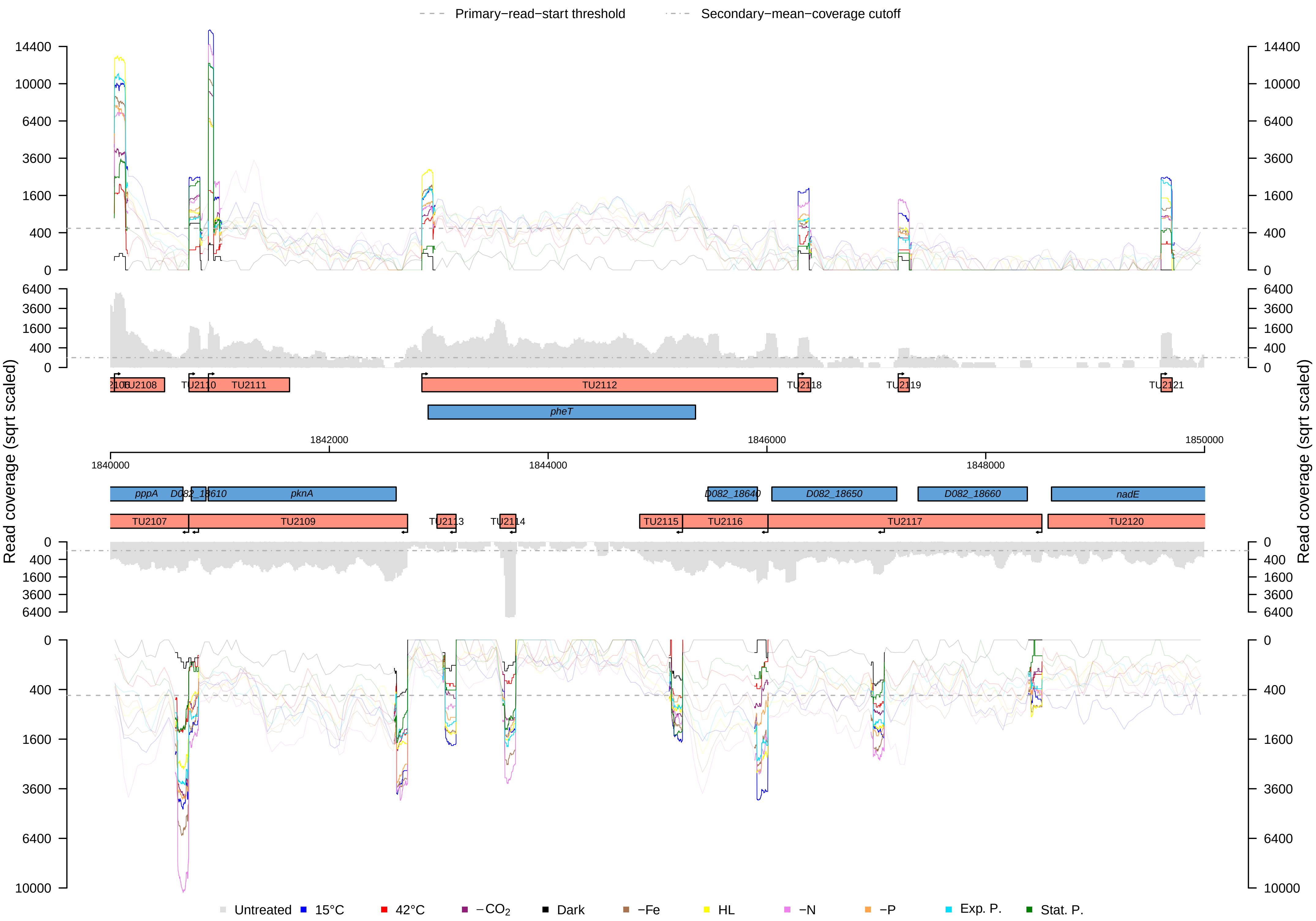

Primary−read−start threshold
Secondary−mean−coverage cutoff
Read coverage (sqrt scaled)
14400
10000
6400
3600
1600
400
0
TU2108
TU2110
TU2111
TU2112
TU2118
TU2119
TU2121
pheT
1840000
1842000
1844000
1846000
1848000
1850000
pppA
D082_18610
pknA
D082_18640
D082_18650
D082_18660
nadE
TU2107
TU2109
TU2113
TU2114
TU2115
TU2116
TU2117
TU2120
Untreated
15°C
42°C
−CO2
Dark
−Fe
HL
−N
−P
Exp. P.
Stat. P.

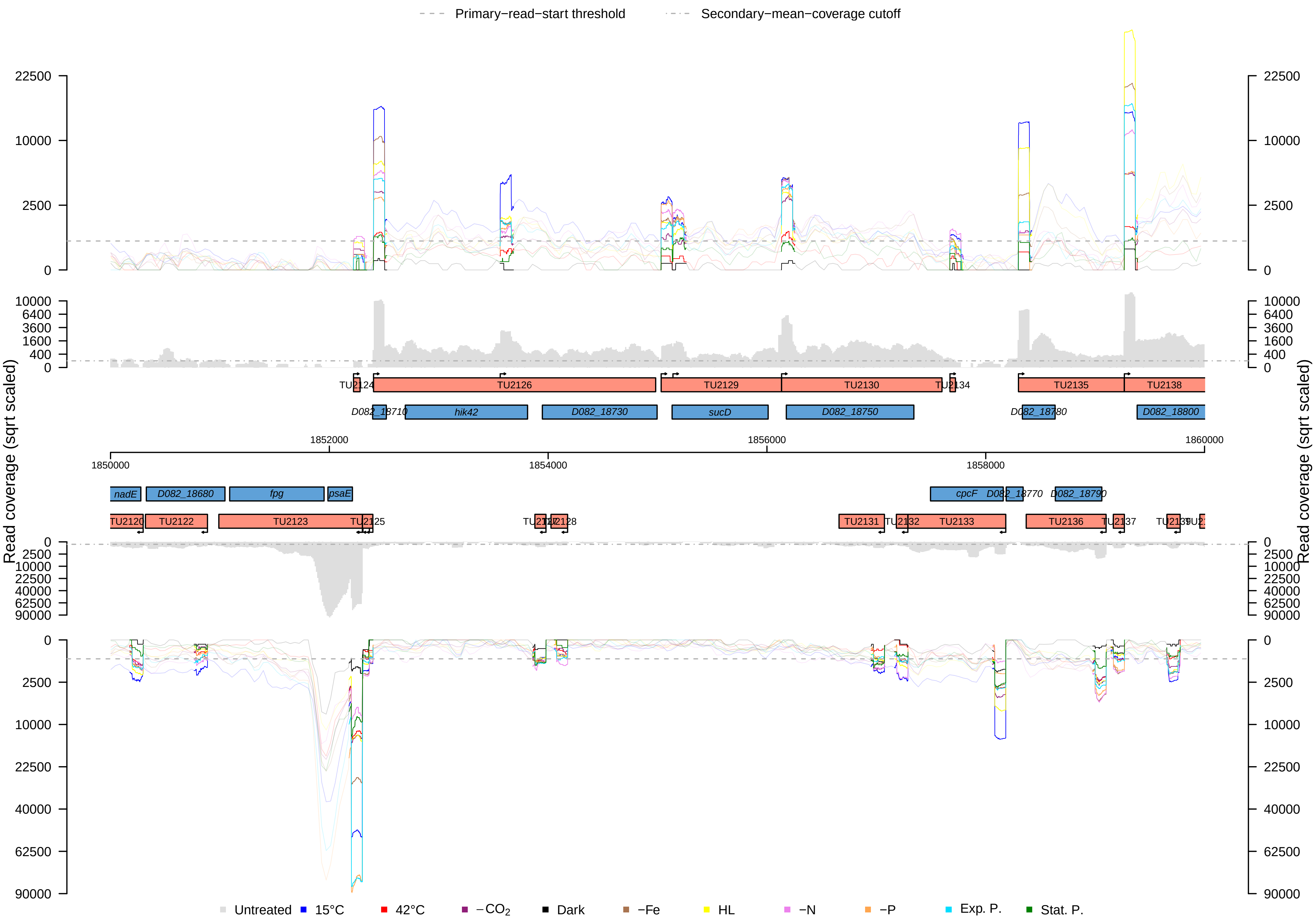

Primary−read−start threshold
Secondary−mean−coverage cutoff
Read coverage (sqrt scaled)
22500
10000
2500
0
6400
3600
1600
400
40000
62500
90000
TU2124
TU2126
TU2129
TU2130
TU2134
TU2135
TU2138
D082_18710
hik42
D082_18730
sucD
D082_18750
D082_18780
D082_18800
1850000
1852000
1854000
1856000
1858000
1860000
nadE
D082_18680
fpg
psaE
cpcF
D082_18770
D082_18790
TU2120
TU2122
TU2123
TU2125
TU2127
TU2128
TU2131
TU2132
TU2133
TU2136
TU2137
TU2139
Untreated
15°C
42°C
−CO2
Dark
−Fe
HL
−N
−P
Exp. P.
Stat. P.

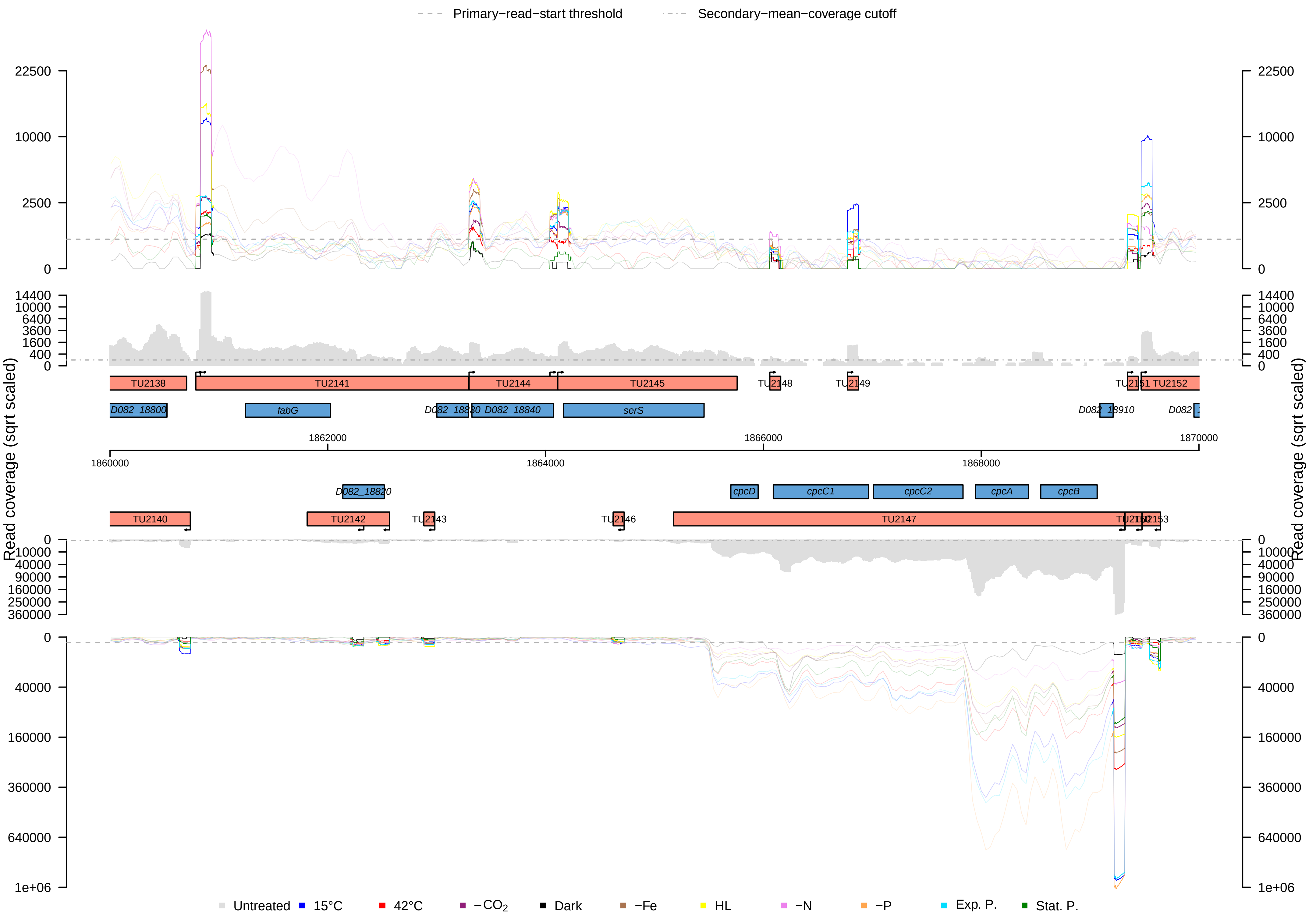

Primary−read−start threshold
Secondary−mean−coverage cutoff
Read coverage (sqrt scaled)
22500
10000
2500
0
14400
10000
6400
3600
1600
400
TU2138
TU2141
TU2144
TU2145
TU2148
TU2149
TU2151
TU2152
D082_18800
fabG
D082_18830
D082_18840
serS
D082_18910
1860000
1862000
1864000
1866000
1868000
1870000
D082_18820
cpcD
cpcC1
cpcC2
cpcA
cpcB
TU2140
TU2142
TU2143
TU2146
TU2147
TU2153
40000
90000
160000
250000
360000
640000
1e+06
Untreated
15°C
42°C
−CO2
Dark
−Fe
HL
−N
−P
Exp. P.
Stat. P.

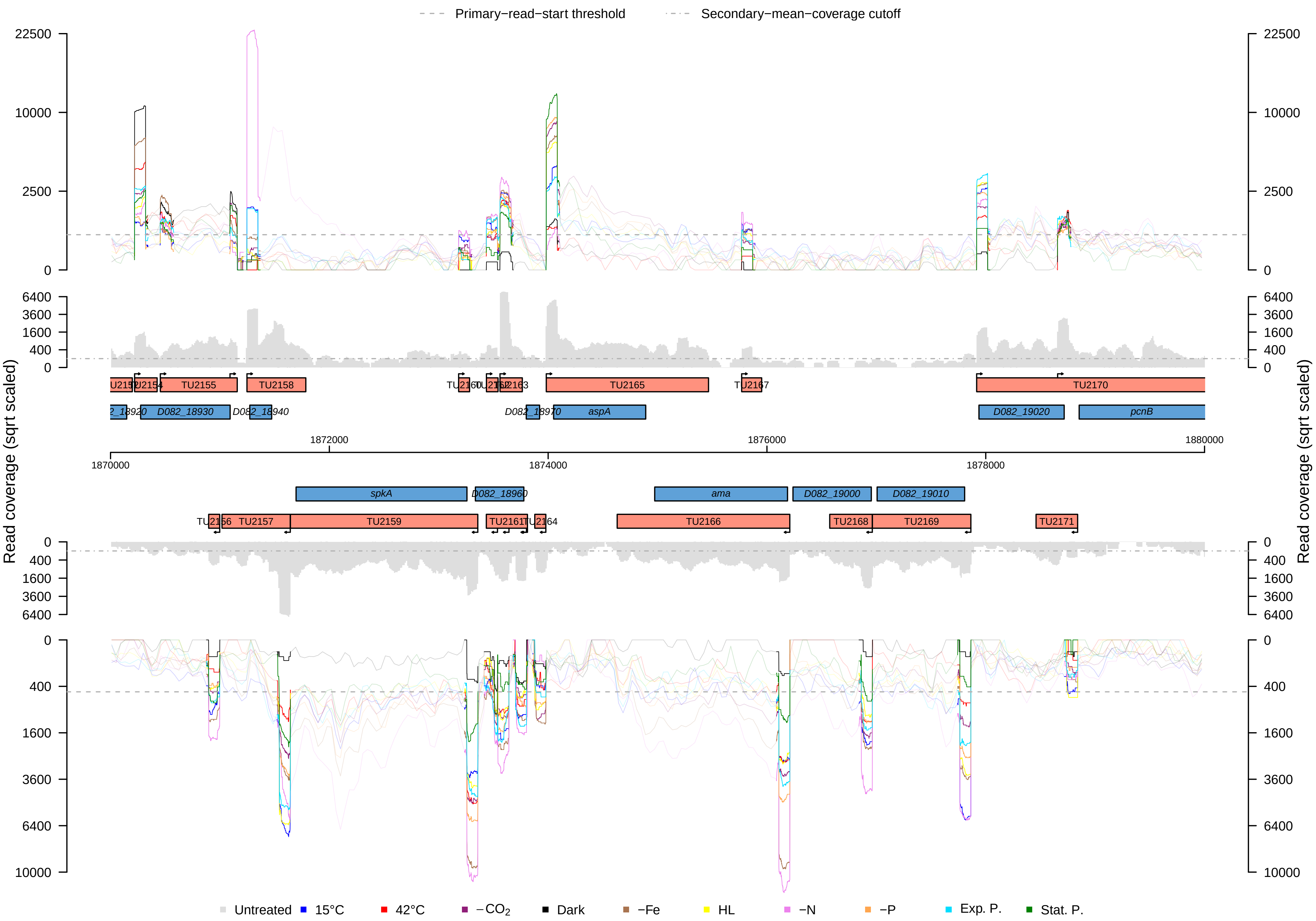

Primary−read−start threshold
Secondary−mean−coverage cutoff
Read coverage (sqrt scaled)
TU2155
TU2158
TU2165
TU2167
TU2170
D082_18930
D082_18940
D082_18970
aspA
D082_19020
pcnB
1870000
1872000
1874000
1876000
1878000
1880000
spkA
D082_18960
ama
D082_19000
D082_19010
TU2157
TU2159
TU2166
TU2168
TU2169
TU2171
Untreated
15°C
42°C
−CO2
Dark
−Fe
HL
−N
−P
Exp. P.
Stat. P.

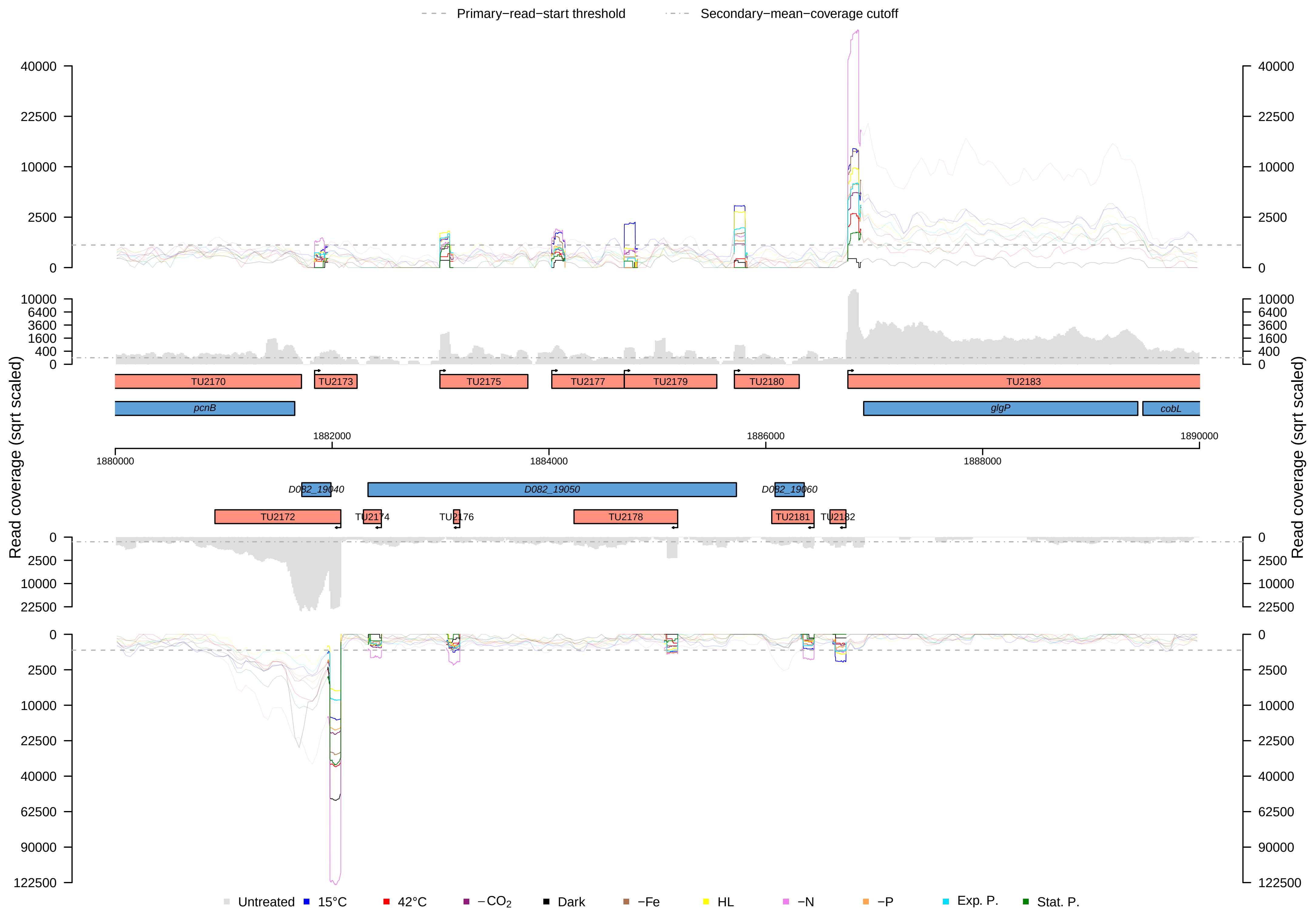

Primary−read−start threshold
Secondary−mean−coverage cutoff
Read coverage (sqrt scaled)
40000
22500
10000
2500
0
6400
3600
1600
400
TU2170
TU2173
TU2175
TU2177
TU2179
TU2180
TU2183
pcnB
glgP
cobL
1880000
1882000
1884000
1886000
1888000
1890000
D082_19040
D082_19050
D082_19060
TU2172
TU2174
TU2176
TU2178
TU2181
TU2182
62500
90000
122500
Untreated
15°C
42°C
−CO2
Dark
−Fe
HL
−N
−P
Exp. P.
Stat. P.

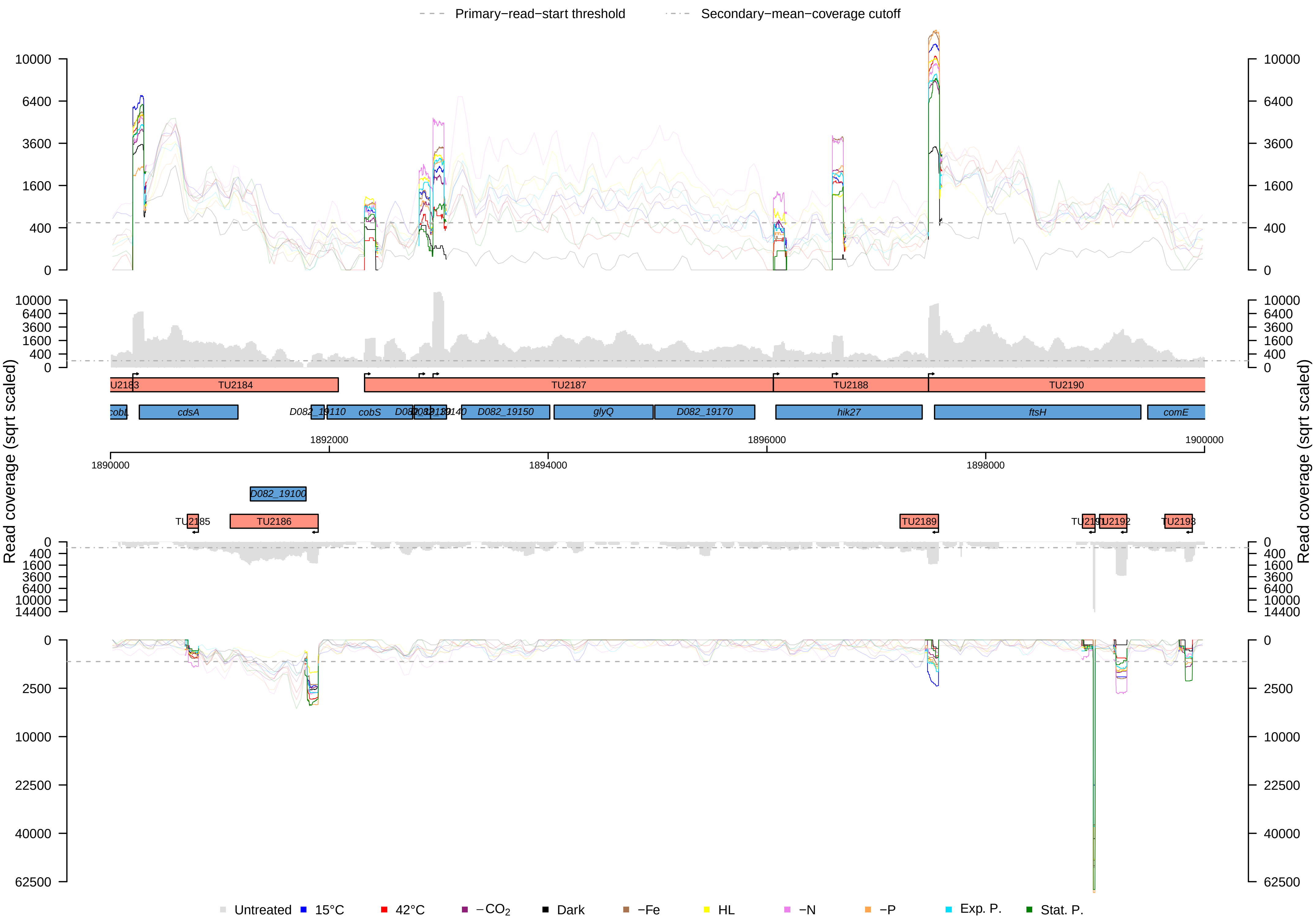

Primary−read−start threshold
Secondary−mean−coverage cutoff
Read coverage (sqrt scaled)
10000
6400
3600
1600
400
0
TU2183
TU2184
TU2187
TU2188
TU2190
cobL
cdsA
D082_19110
cobS
D082_19140
D082_19150
glyQ
D082_19170
hik27
ftsH
comE
1890000
1892000
1894000
1896000
1898000
1900000
D082_19100
TU2185
TU2186
TU2189
TU2191
TU2192
TU2193
14400
2500
22500
40000
62500
Untreated
15°C
42°C
−CO2
Dark
−Fe
HL
−N
−P
Exp. P.
Stat. P.

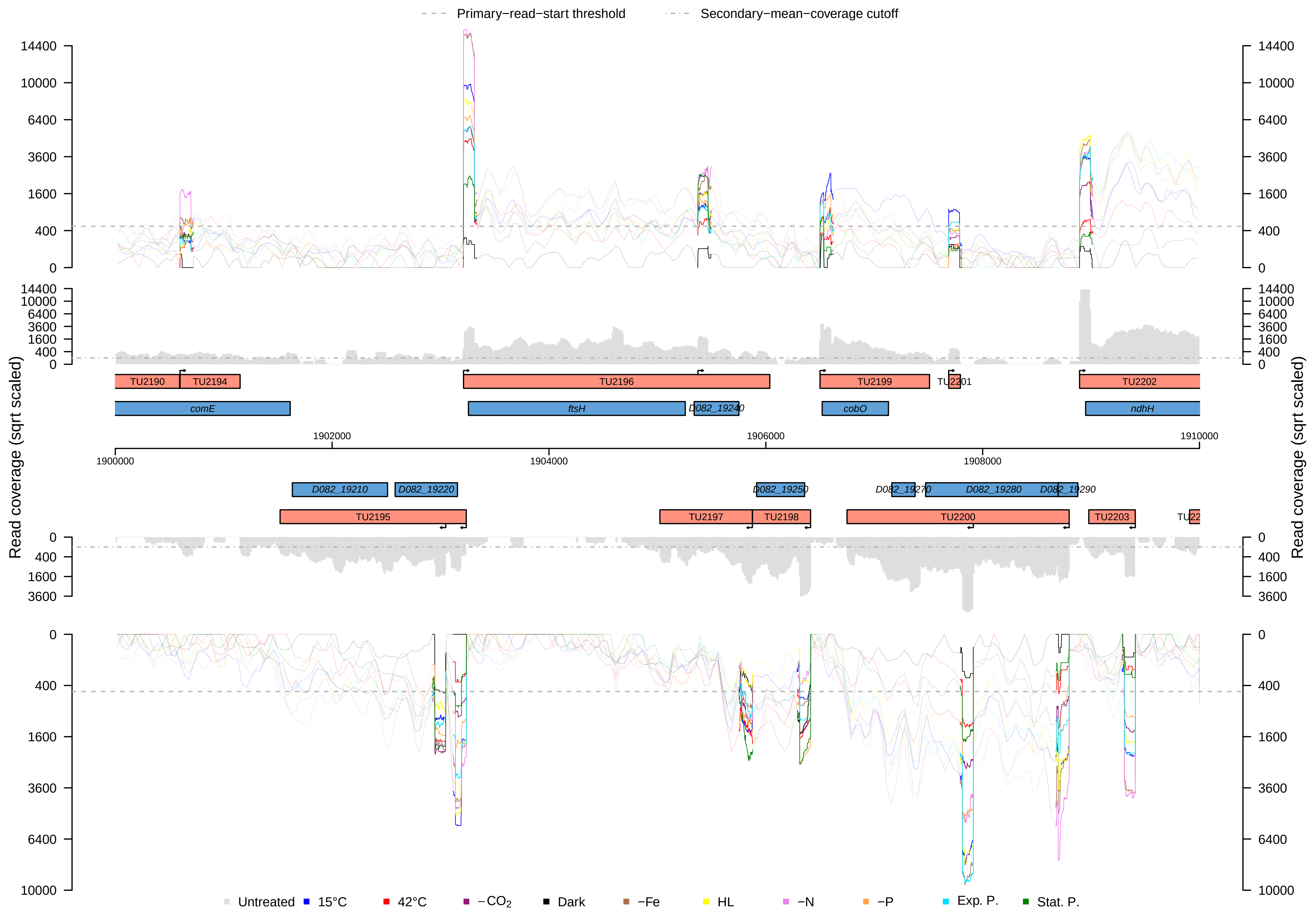

Primary−read−start threshold
Secondary−mean−coverage cutoff
Read coverage (sqrt scaled)
14400
10000
6400
3600
1600
400
0
TU2190
TU2194
TU2196
TU2199
TU2201
TU2202
comE
ftsH
D082_19240
cobO
ndhH
1900000
1902000
1904000
1906000
1908000
1910000
D082_19210
D082_19220
D082_19250
D082_19270
D082_19280
D082_19290
TU2195
TU2197
TU2198
TU2200
TU2203
TU22
Untreated
15°C
42°C
−CO2
Dark
−Fe
HL
−N
−P
Exp. P.
Stat. P.

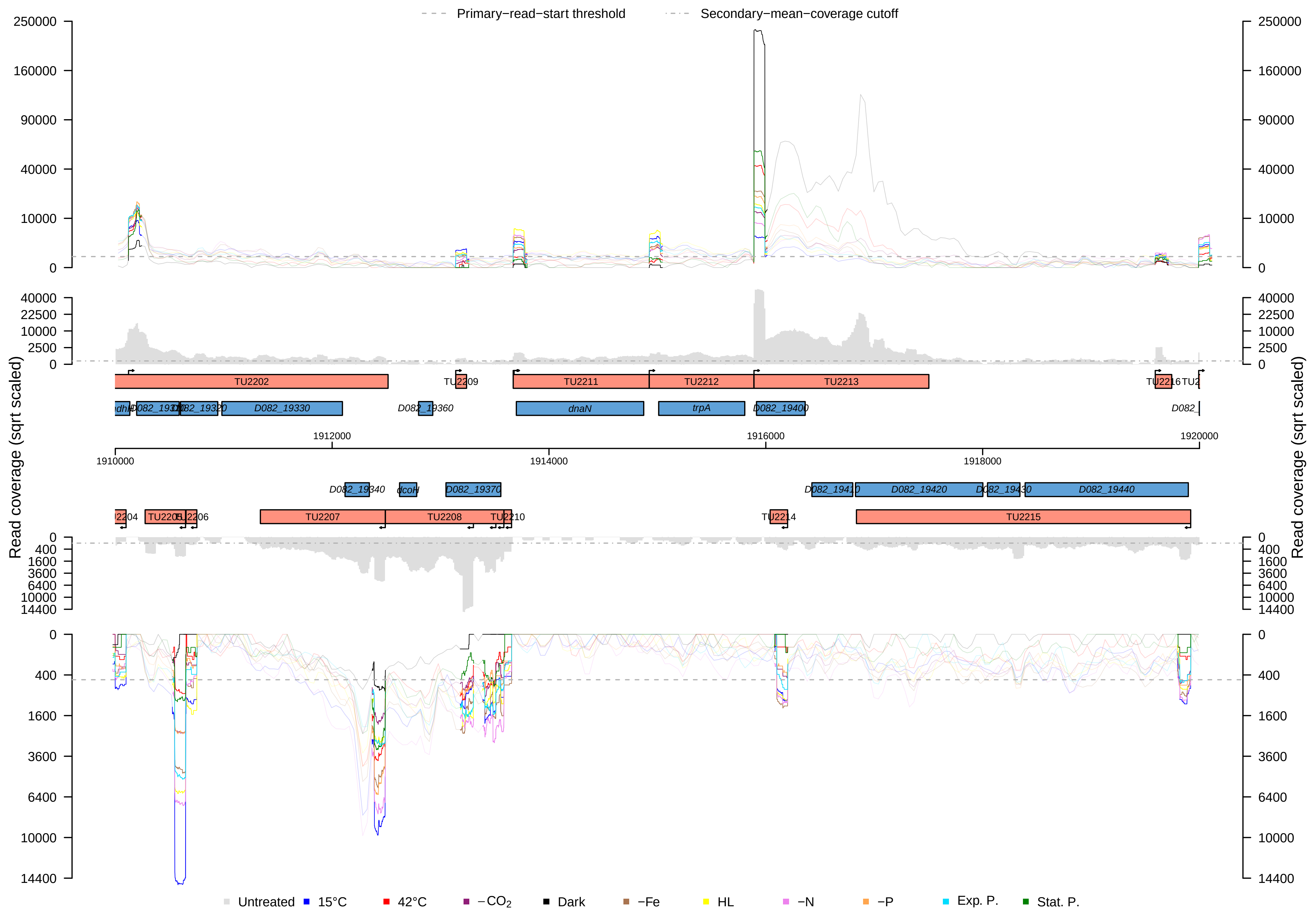

Primary−read−start threshold
Secondary−mean−coverage cutoff
Read coverage (sqrt scaled)
TU2202
TU2209
TU2211
TU2212
TU2213
D082_19330
D082_19360
dnaN
trpA
D082_19400
1910000
1912000
1914000
1916000
1918000
1920000
D082_19340
dcoH
D082_19370
D082_19420
D082_19440
TU2207
TU2208
TU2215
Untreated
15°C
42°C
−CO2
Dark
−Fe
HL
−N
−P
Exp. P.
Stat. P.

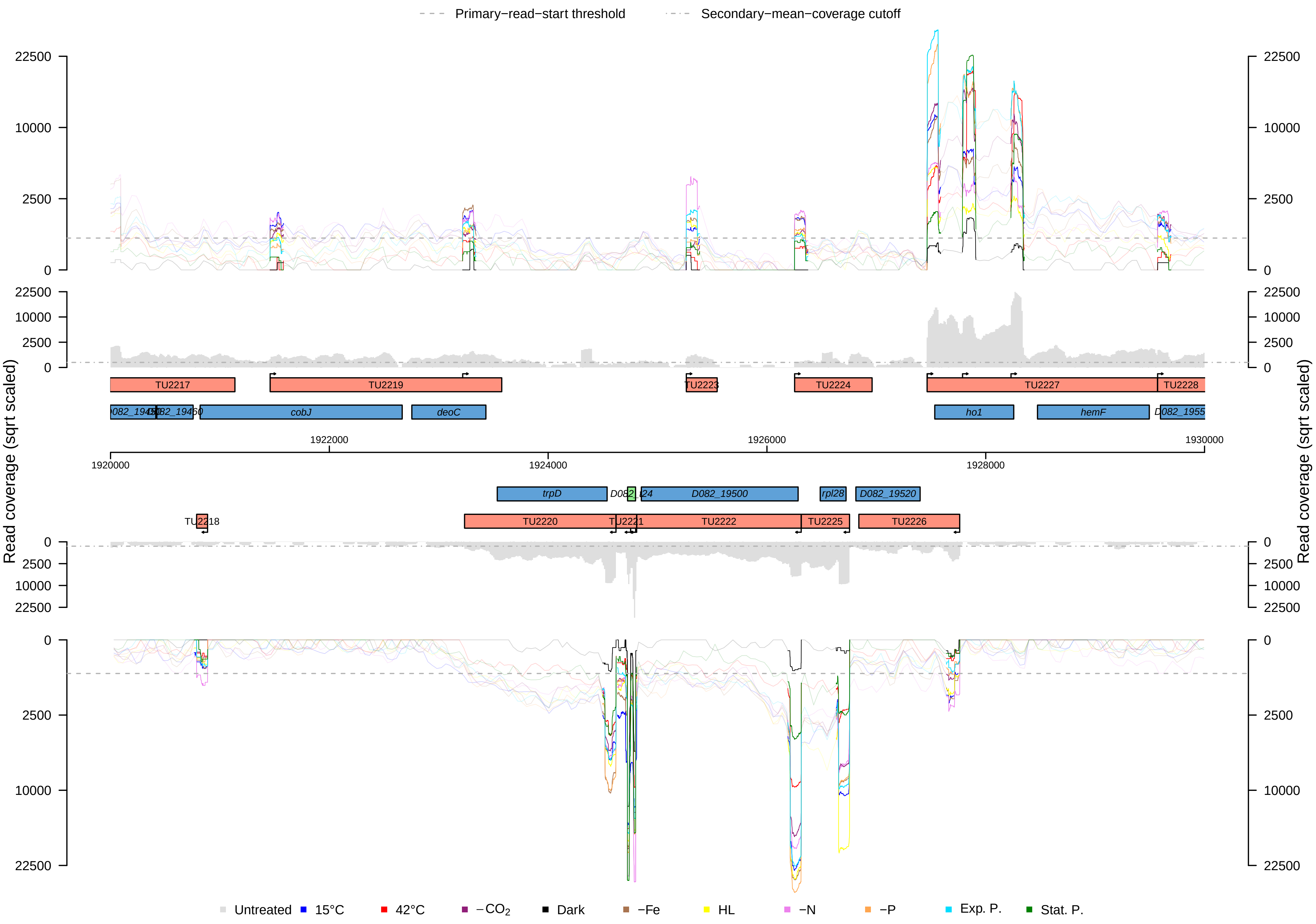

Primary−read−start threshold
Secondary−mean−coverage cutoff
Read coverage (sqrt scaled)
22500
10000
2500
0
TU2217
TU2219
TU2223
TU2224
TU2227
TU2228
cobJ
deoC
ho1
hemF
1920000
1922000
1924000
1926000
1928000
1930000
trpD
D082_19500
rpl28
D082_19520
TU2218
TU2220
TU2221
TU2222
TU2225
TU2226
Untreated
15°C
42°C
−CO2
Dark
−Fe
HL
−N
−P
Exp. P.
Stat. P.

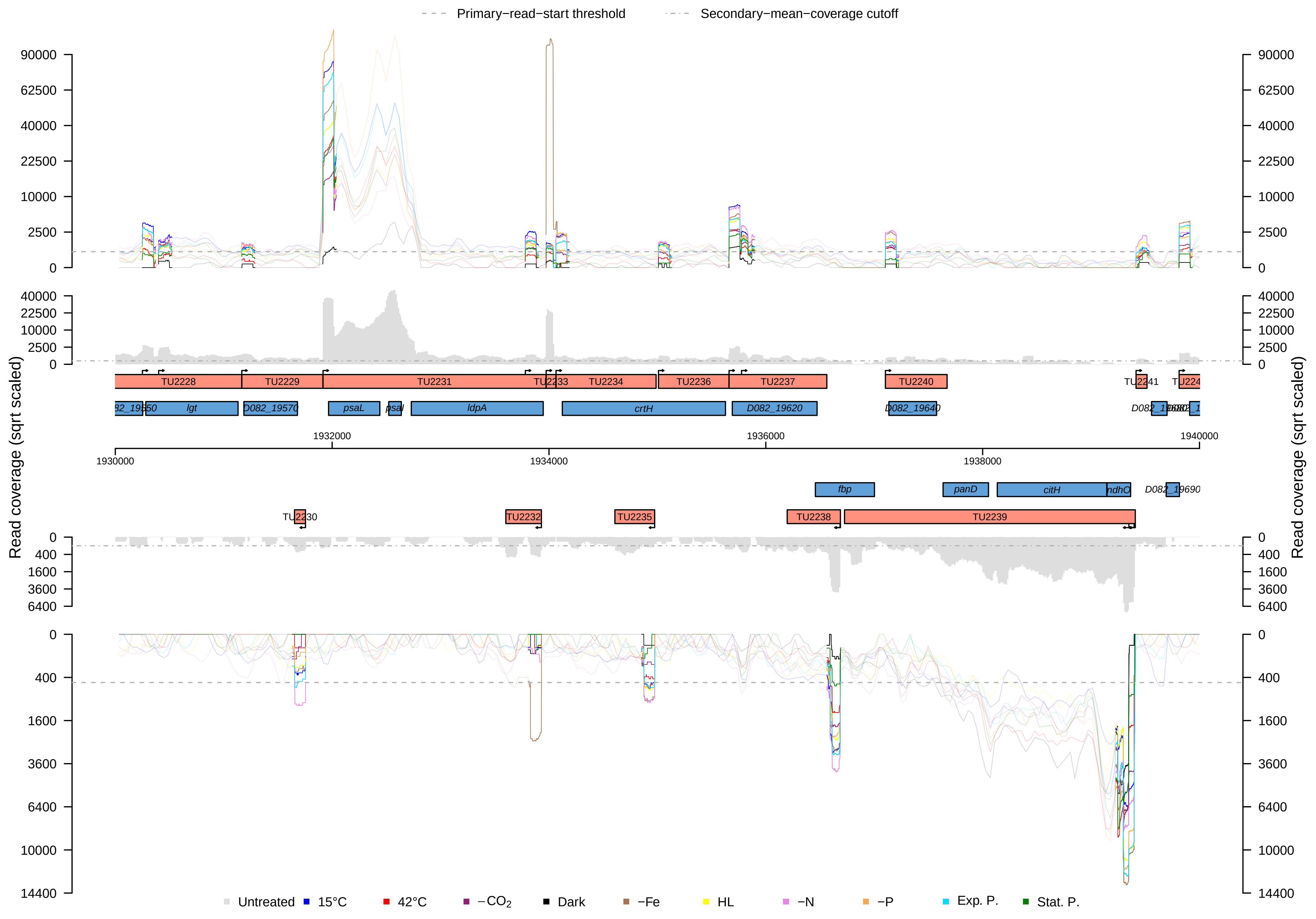

Primary−read−start threshold
Secondary−mean−coverage cutoff
Read coverage (sqrt scaled)
90000
62500
40000
22500
10000
2500
0
400
1600
3600
6400
14400
TU2228
TU2229
TU2231
TU2233
TU2234
TU2236
TU2237
TU2240
TU2241
TU2230
TU2232
TU2235
TU2238
TU2239
lgt
D082_19570
psaL
psaI
ldpA
crtH
D082_19620
D082_19640
fbp
panD
citH
ndhO
D082_19690
1930000
1932000
1934000
1936000
1938000
1940000
Untreated
15°C
42°C
−CO2
Dark
−Fe
HL
−N
−P
Exp. P.
Stat. P.

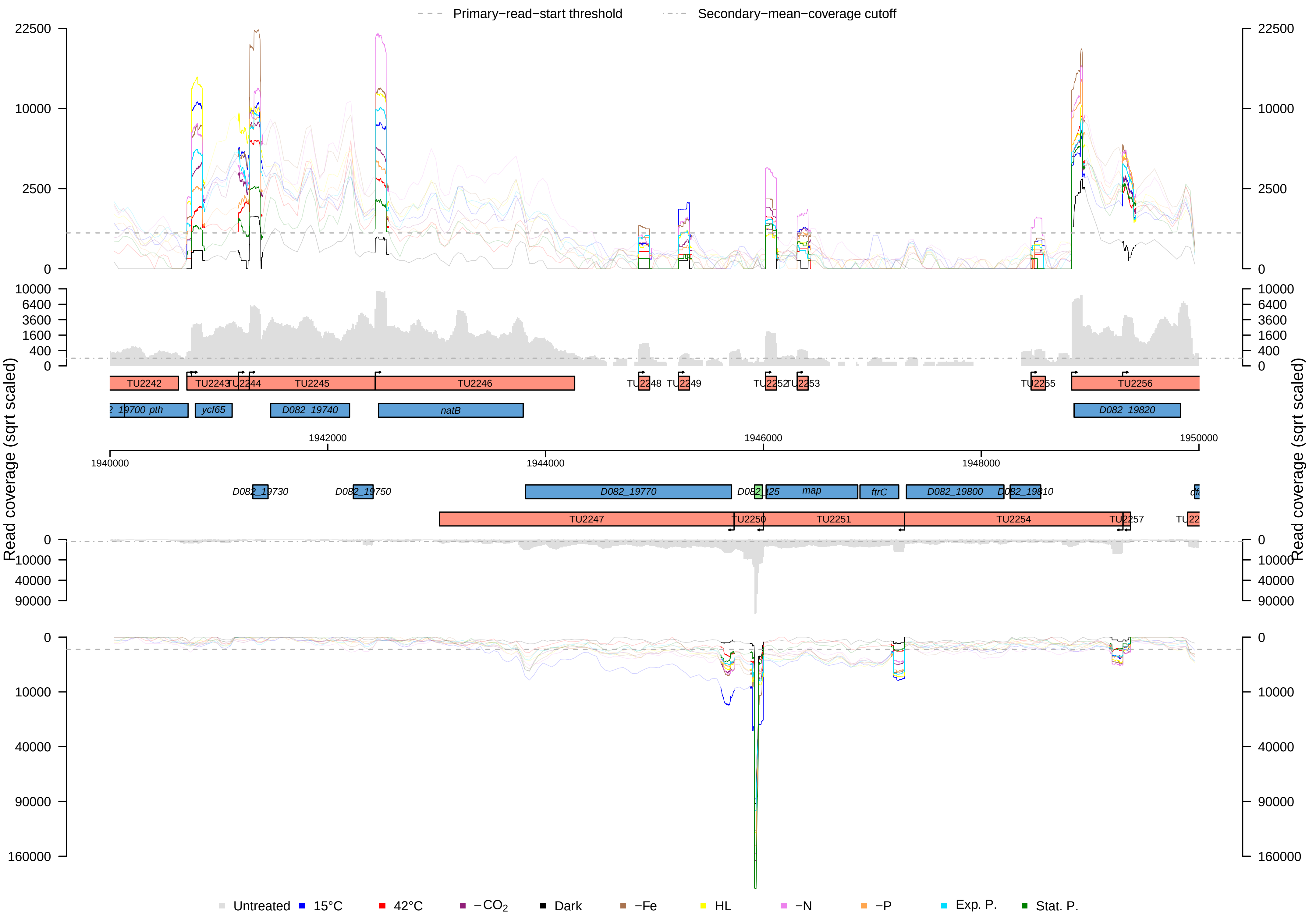

Primary−read−start threshold
Secondary−mean−coverage cutoff
Read coverage (sqrt scaled)
22500
10000
2500
0
6400
3600
1600
400
40000
90000
160000
TU2242
TU2243
TU2244
TU2245
TU2246
TU2248
TU2249
TU2252
TU2253
TU2255
TU2256
pth
ycf65
D082_19740
natB
D082_19820
1940000
1942000
1944000
1946000
1948000
1950000
D082_19730
D082_19750
D082_19770
map
ftrC
D082_19800
D082_19810
TU2247
TU2250
TU2251
TU2254
TU2257
Untreated
15°C
42°C
−CO2
Dark
−Fe
HL
−N
−P
Exp. P.
Stat. P.

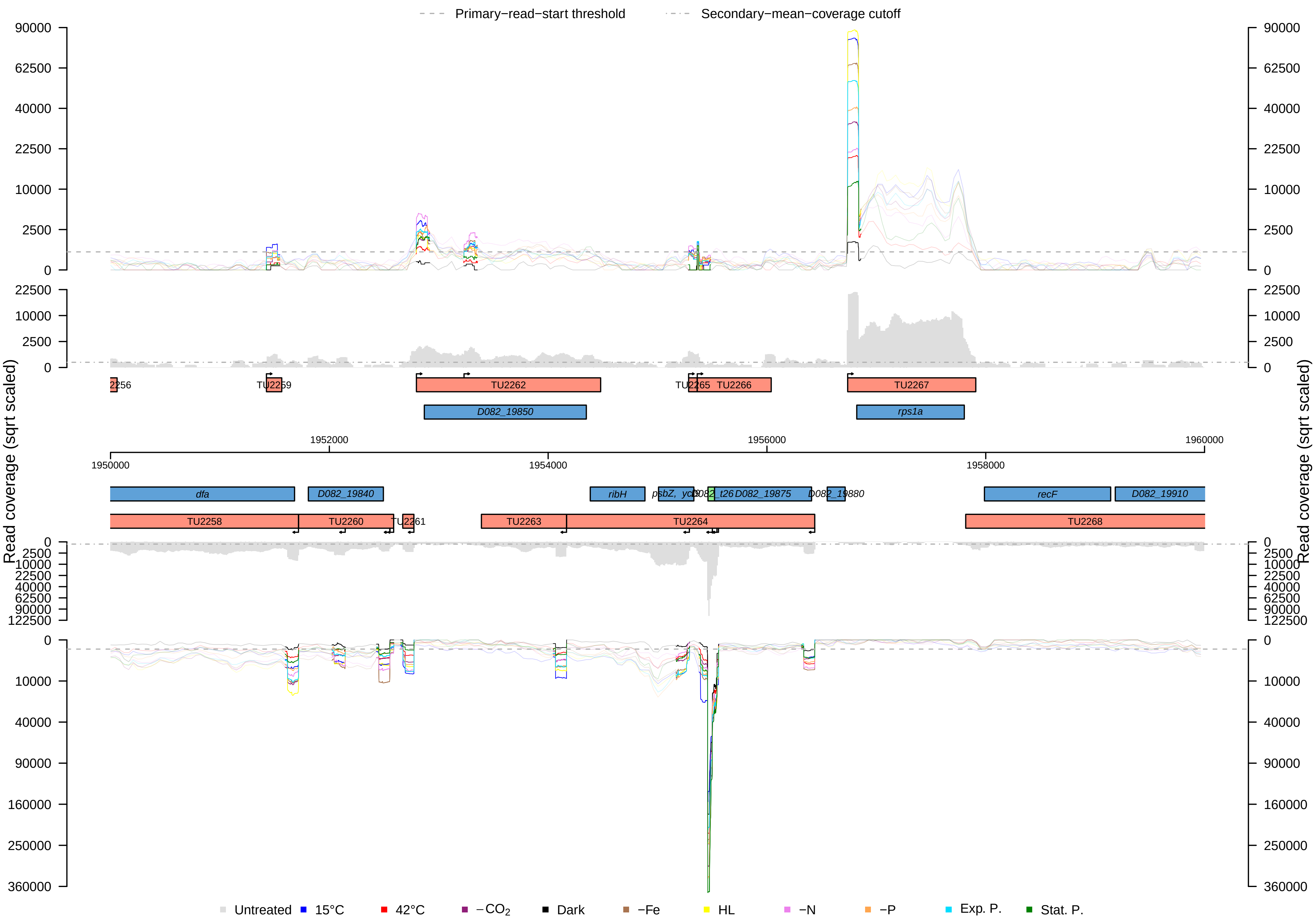

Primary−read−start threshold
Secondary−mean−coverage cutoff
Read coverage (sqrt scaled)
TU2262
TU2265
TU2266
TU2267
D082_19850
rps1a
1950000
1952000
1954000
1956000
1958000
1960000
dfa
D082_19840
ribH
D082_19875
recF
D082_19910
TU2258
TU2260
TU2261
TU2263
TU2264
TU2268
Untreated
15°C
42°C
−CO2
Dark
−Fe
HL
−N
−P
Exp. P.
Stat. P.

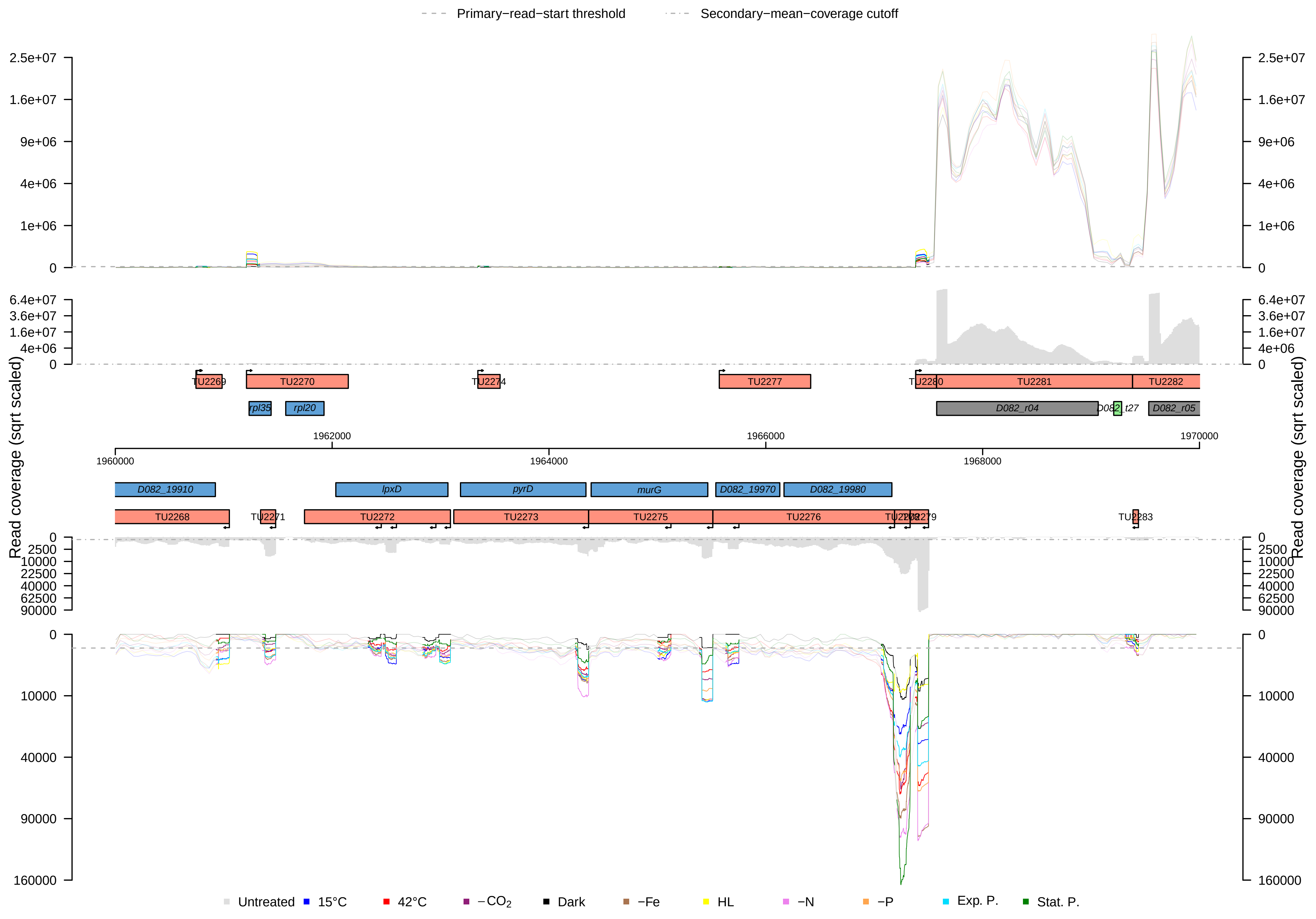

Primary−read−start threshold
Secondary−mean−coverage cutoff
Read coverage (sqrt scaled)
2.5e+07
1.6e+07
9e+06
4e+06
1e+06
0
6.4e+07
3.6e+07
1.6e+07
4e+06
0
TU2269
TU2270
TU2274
TU2277
TU2280
TU2281
TU2282
rpl35
rpl20
D082_r04
D082_t27
D082_r05
1962000
1966000
1970000
1960000
1964000
1968000
D082_19910
lpxD
pyrD
murG
D082_19970
D082_19980
TU2268
TU2271
TU2272
TU2273
TU2275
TU2276
TU2278
TU2279
TU2283
0
2500
10000
22500
40000
62500
90000
0
10000
40000
90000
160000
Untreated
15°C
42°C
−CO2
Dark
−Fe
HL
−N
−P
Exp. P.
Stat. P.

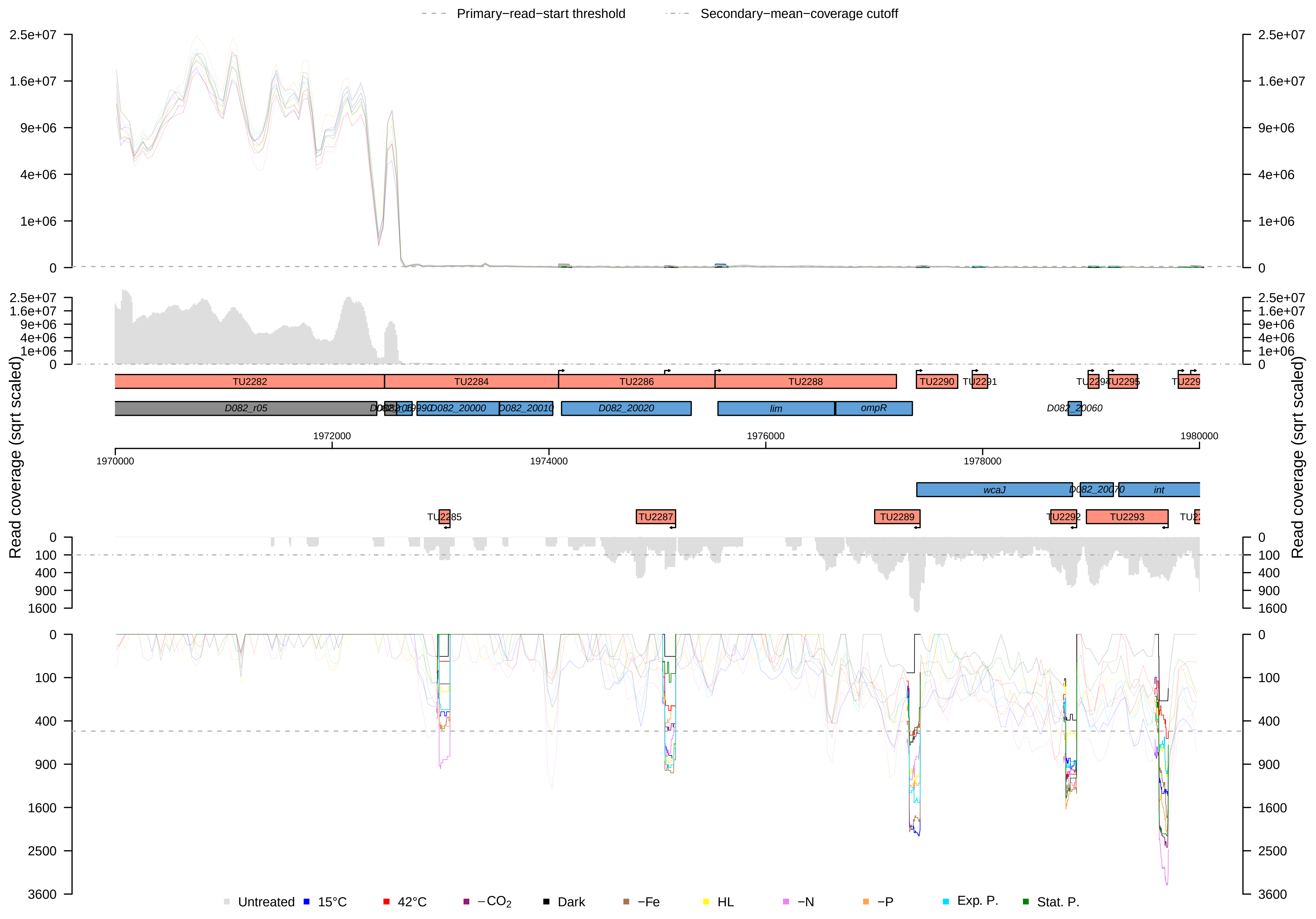

Primary−read−start threshold
Secondary−mean−coverage cutoff
Read coverage (sqrt scaled)
2.5e+07
1.6e+07
9e+06
4e+06
1e+06
0
100
400
900
1600
2500
3600
TU2282
TU2284
TU2286
TU2288
TU2290
TU2291
TU2295
TU2285
TU2287
TU2289
TU2292
TU2293
D082_r05
D082_20000
D082_20010
D082_20020
lim
ompR
D082_20060
wcaJ
int
1970000
1972000
1974000
1976000
1978000
1980000
Untreated
15°C
42°C
−CO2
Dark
−Fe
HL
−N
−P
Exp. P.
Stat. P.

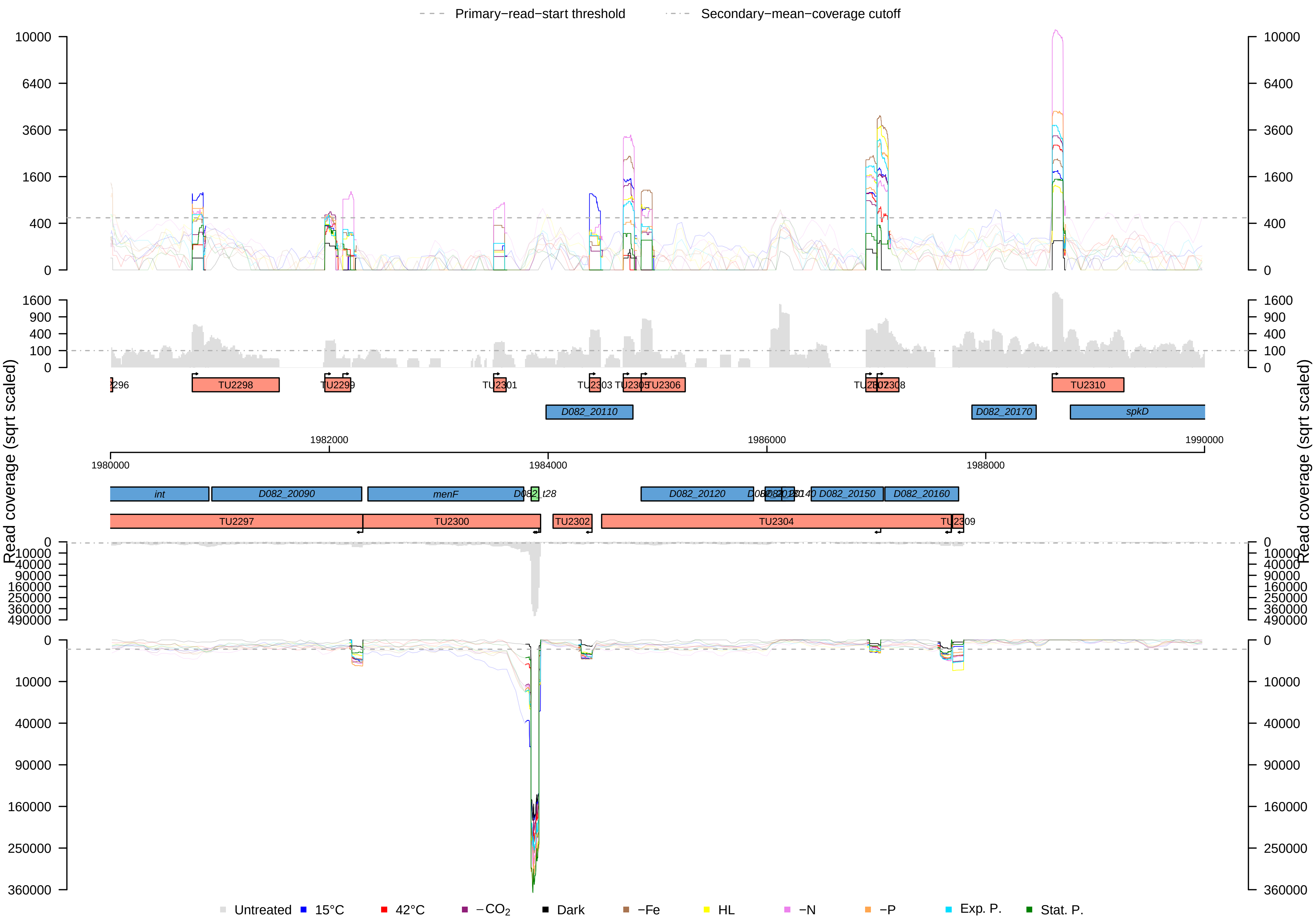

Primary−read−start threshold
Secondary−mean−coverage cutoff
Read coverage (sqrt scaled)
TU2298
TU2299
TU2301
TU2303
TU2305
TU2306
TU2308
TU2310
D082_20110
D082_20170
spkD
1980000
1982000
1984000
1986000
1988000
1990000
int
D082_20090
menF
t28
D082_20120
D082_20150
D082_20160
TU2297
TU2300
TU2302
TU2304
TU2309
Untreated
15°C
42°C
−CO2
Dark
−Fe
HL
−N
−P
Exp. P.
Stat. P.

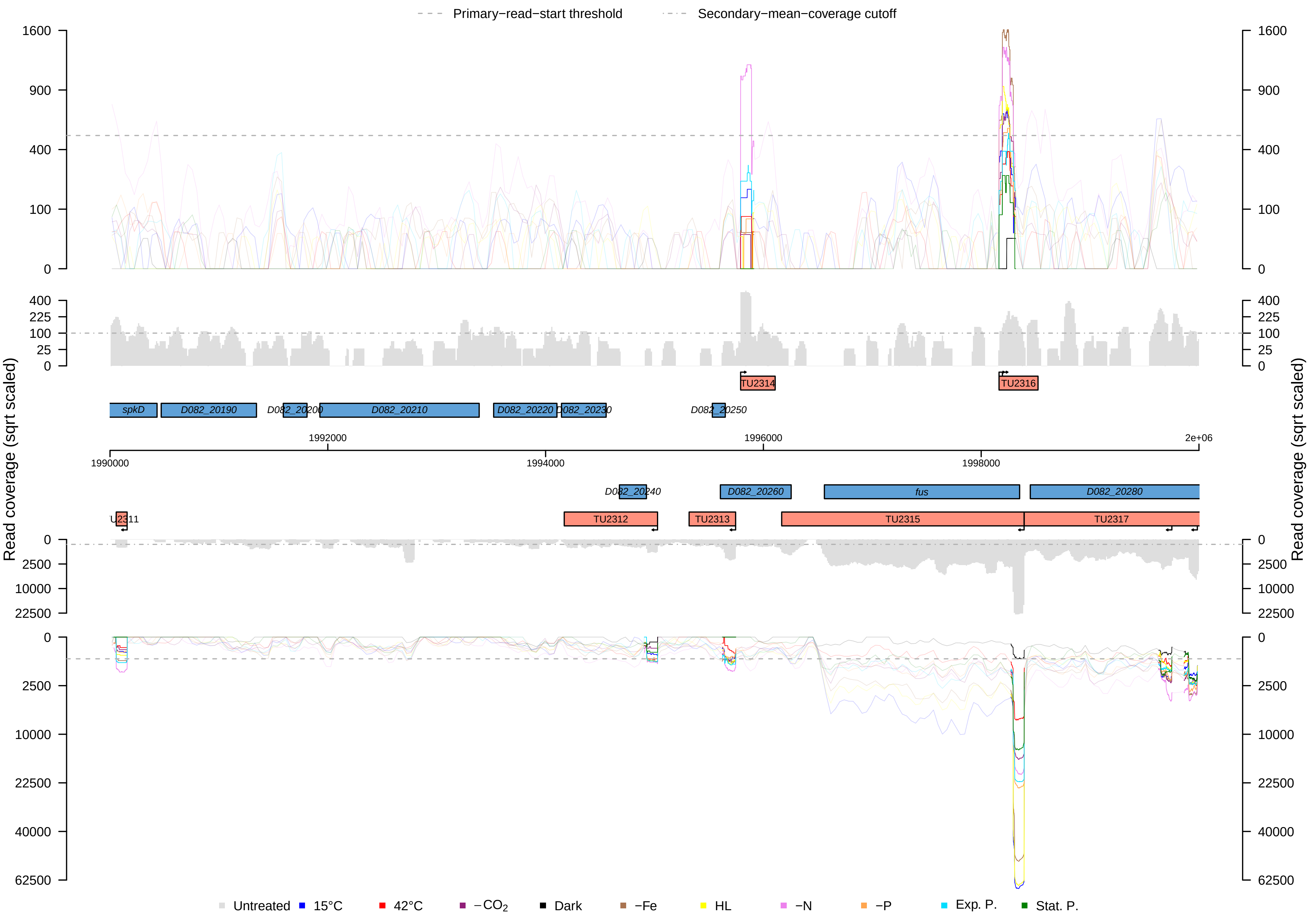

Primary−read−start threshold
Secondary−mean−coverage cutoff
Read coverage (sqrt scaled)
1600
900
400
100
0
225
25
2500
10000
22500
40000
62500
TU2314
TU2316
spkD
D082_20190
D082_20200
D082_20210
D082_20220
D082_20230
D082_20250
1990000
1992000
1994000
1996000
1998000
2e+06
D082_20240
D082_20260
fus
D082_20280
TU2311
TU2312
TU2313
TU2315
TU2317
Untreated
15°C
42°C
−CO2
Dark
−Fe
HL
−N
−P
Exp. P.
Stat. P.

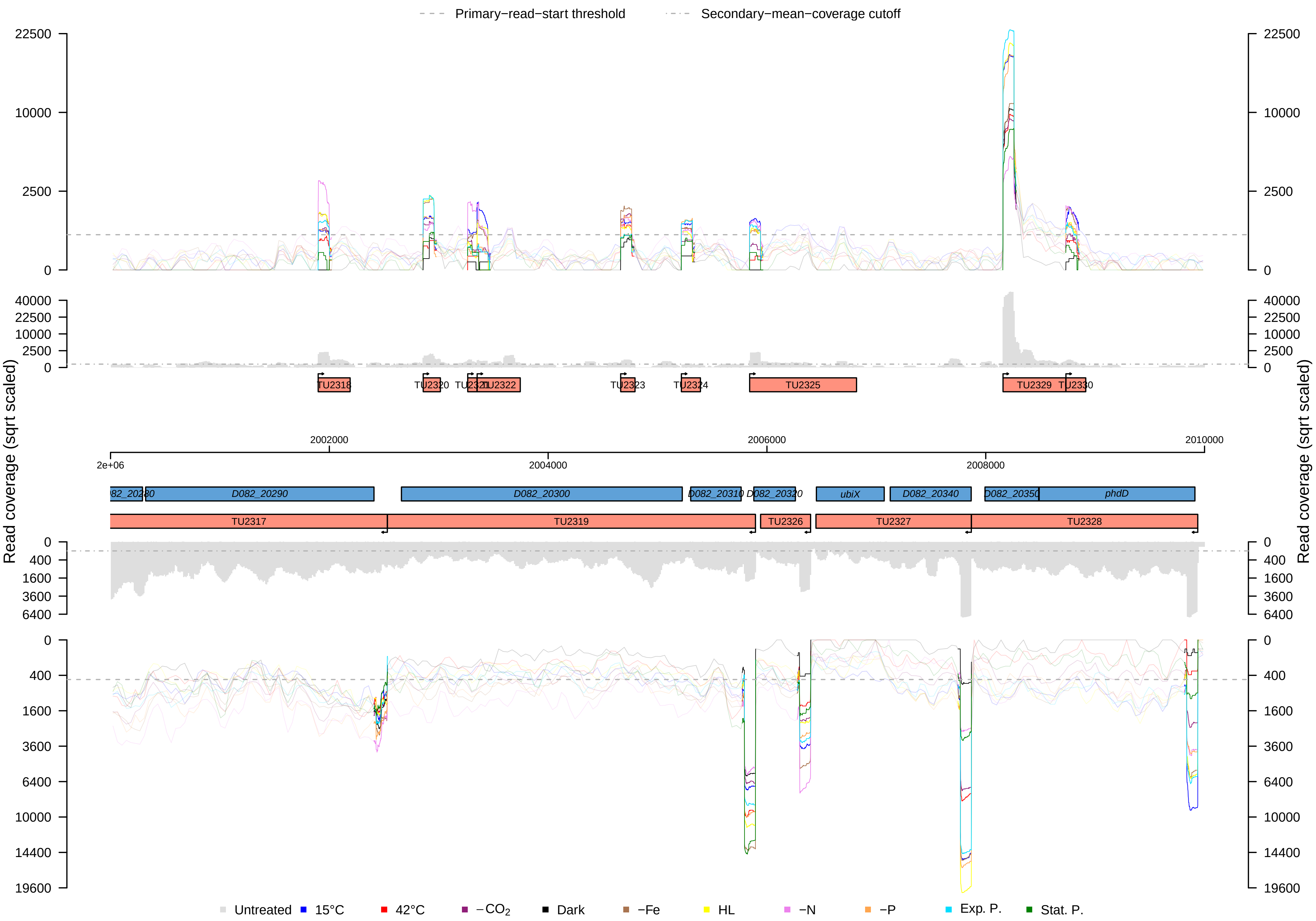

Primary−read−start threshold
Secondary−mean−coverage cutoff
Read coverage (sqrt scaled)
TU2318
TU2320
TU2321
TU2322
TU2323
TU2324
TU2325
TU2329
TU2330
2e+06
2002000
2004000
2006000
2008000
2010000
D082_20290
D082_20300
D082_20310
D082_20320
ubiX
D082_20340
D082_20350
phdD
TU2317
TU2319
TU2326
TU2327
TU2328
Untreated
15°C
42°C
−CO2
Dark
−Fe
HL
−N
−P
Exp. P.
Stat. P.

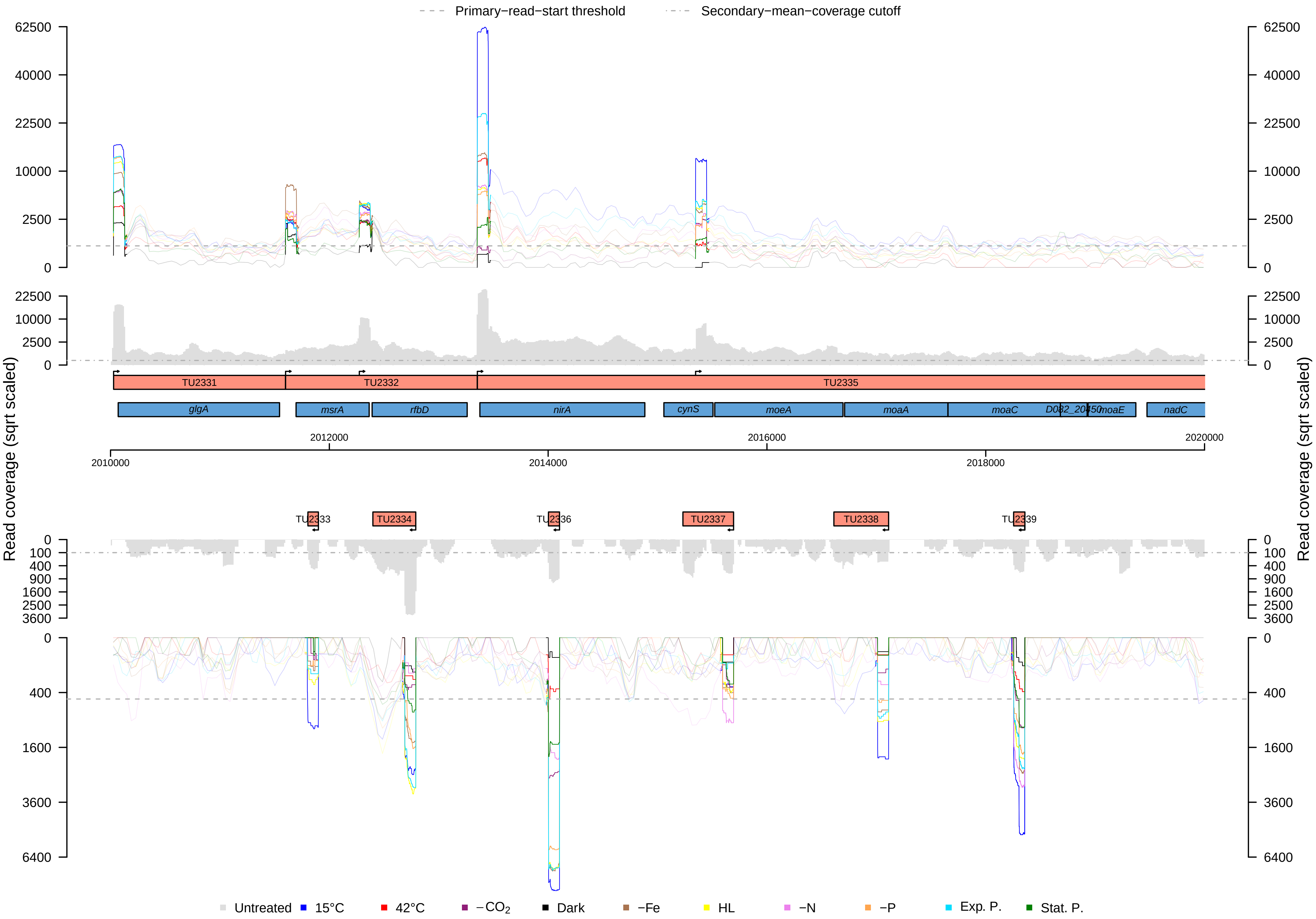

Primary−read−start threshold
Secondary−mean−coverage cutoff
Read coverage (sqrt scaled)
62500
40000
22500
10000
2500
0
100
400
900
1600
3600
6400
TU2331
TU2332
TU2335
glgA
msrA
rfbD
nirA
cynS
moeA
moaA
moaC
D082_20450
moaE
nadC
2010000
2012000
2014000
2016000
2018000
2020000
TU2333
TU2334
TU2336
TU2337
TU2338
TU2339
Untreated
15°C
42°C
−CO_2
Dark
−Fe
HL
−N
−P
Exp. P.
Stat. P.

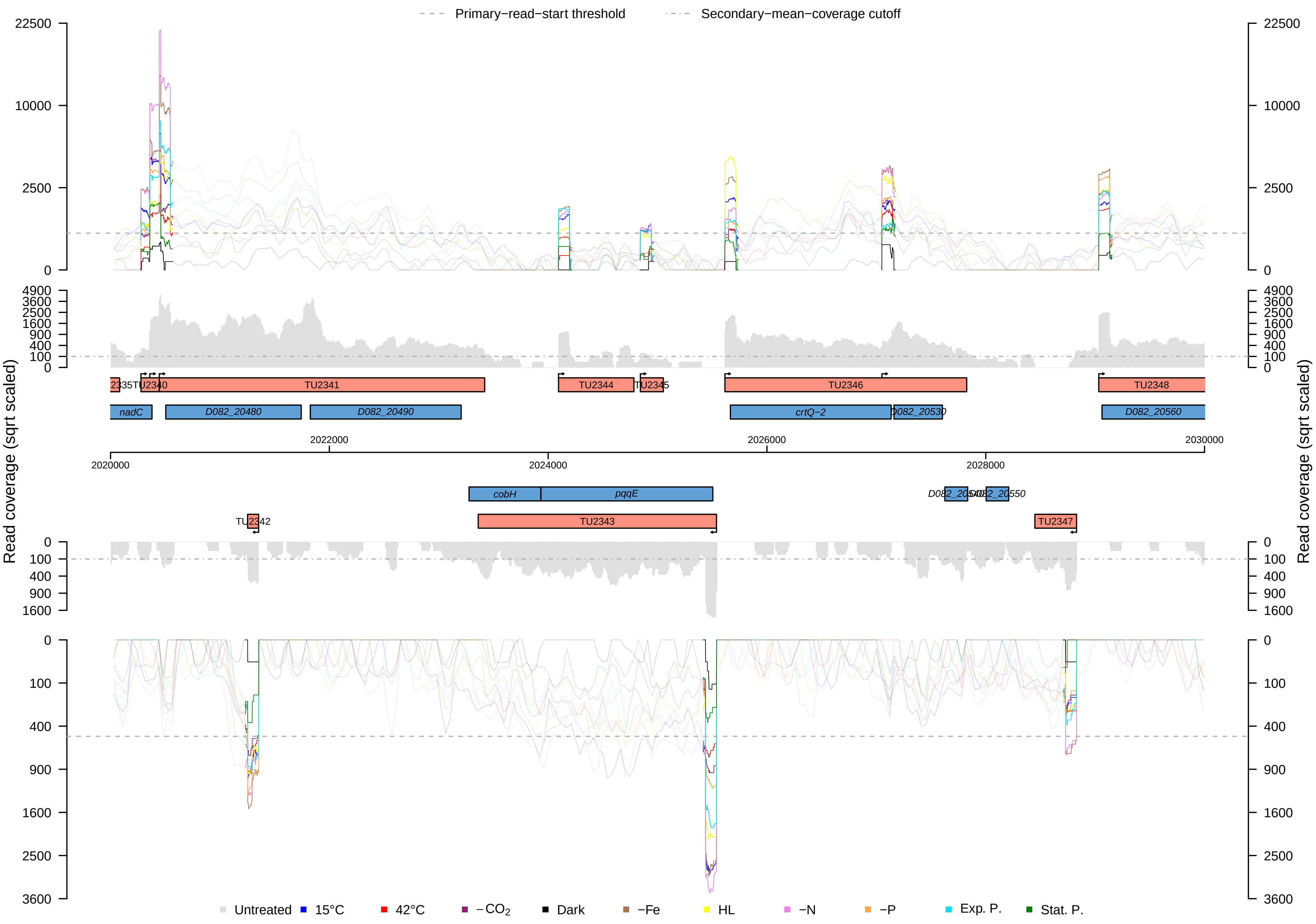

Primary−read−start threshold
Secondary−mean−coverage cutoff
Read coverage (sqrt scaled)
TU2335
TU2340
TU2341
TU2344
TU2345
TU2346
TU2348
nadC
D082_20480
D082_20490
crtQ−2
D082_20530
D082_20560
2020000
2022000
2024000
2026000
2028000
2030000
cobH
pqqE
D082_20540
D082_20550
TU2342
TU2343
TU2347
Untreated
15°C
42°C
−CO2
Dark
−Fe
HL
−N
−P
Exp. P.
Stat. P.

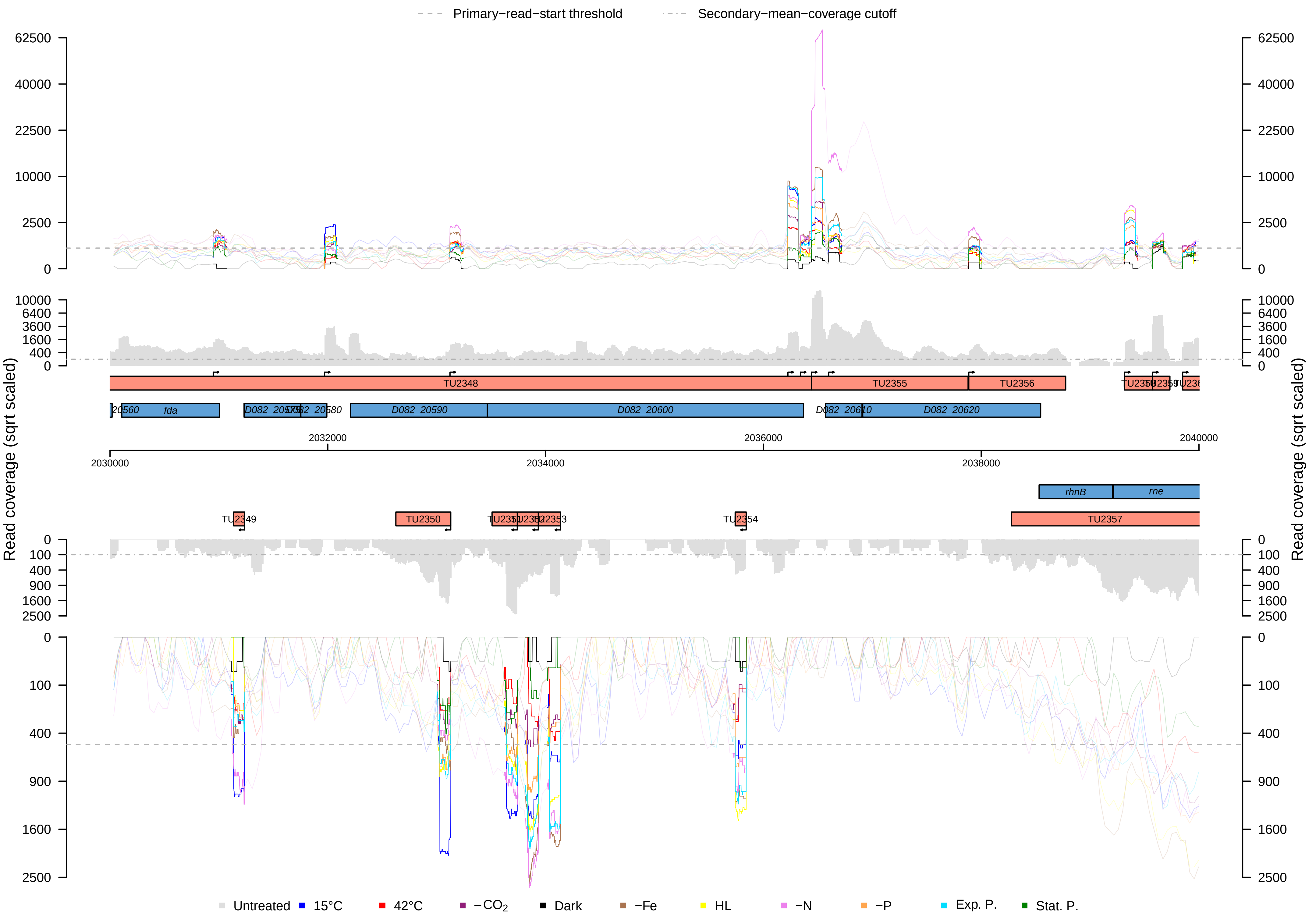

Primary−read−start threshold
Secondary−mean−coverage cutoff
Read coverage (sqrt scaled)
62500
40000
22500
10000
2500
0
10000
6400
3600
1600
400
100
900
2500
TU2348
TU2355
TU2356
fda
D082_20590
D082_20600
D082_20620
2030000
2032000
2034000
2036000
2038000
2040000
rhnB
rne
TU2349
TU2350
TU2354
TU2357
Untreated
15°C
42°C
−CO2
Dark
−Fe
HL
−N
−P
Exp. P.
Stat. P.

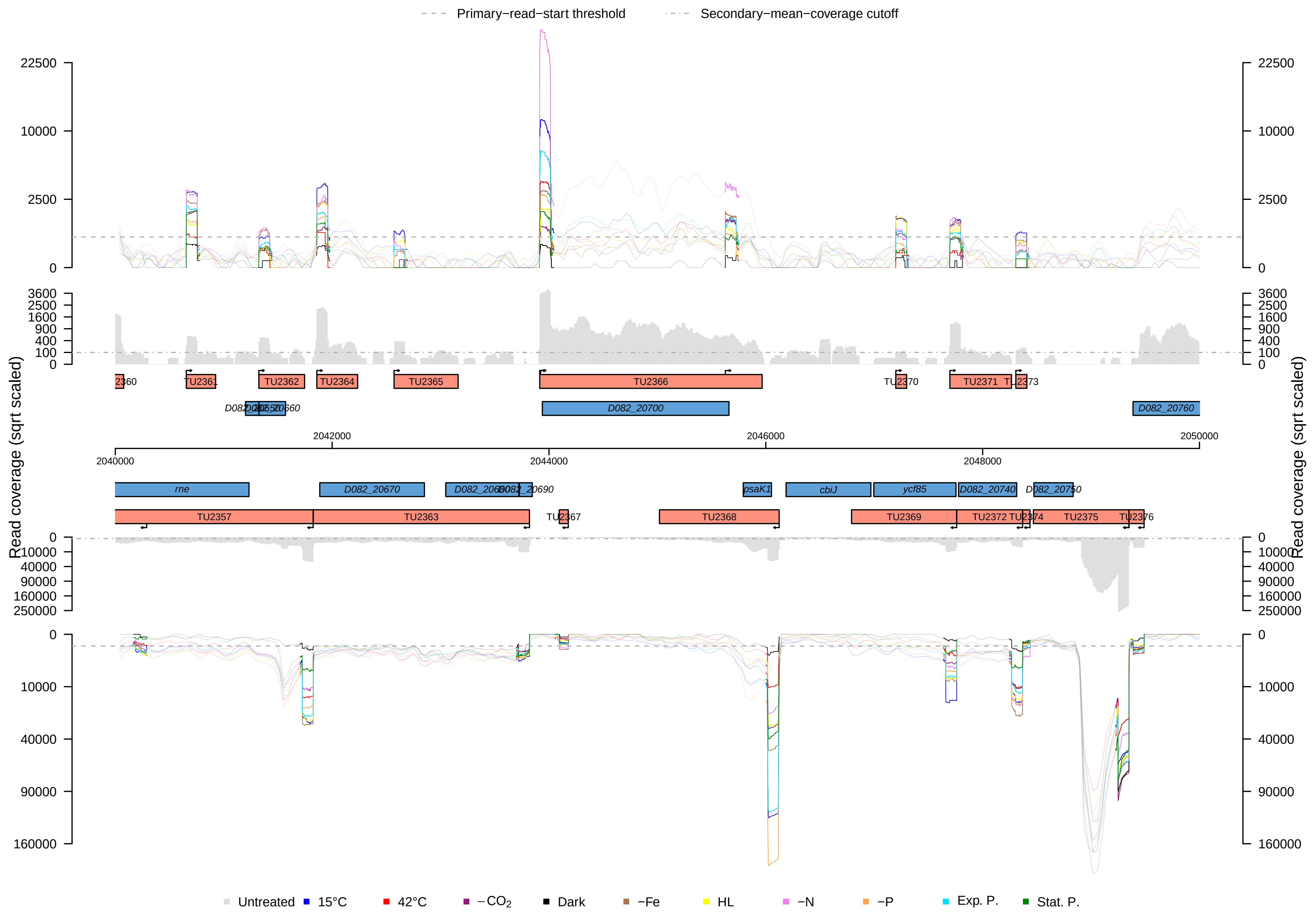

Primary−read−start threshold
Secondary−mean−coverage cutoff
Read coverage (sqrt scaled)
22500
10000
2500
0
3600
2500
1600
900
400
100
TU2361
TU2362
TU2364
TU2365
TU2366
TU2371
D082_20700
D082_20760
2042000
2046000
2050000
2040000
2044000
2048000
rne
D082_20670
D082_20690
psaK1
cbiJ
ycf85
D082_20740
D082_20750
TU2357
TU2363
TU2368
TU2369
TU2375
40000
90000
160000
250000
Untreated
15°C
42°C
−CO2
Dark
−Fe
HL
−N
−P
Exp. P.
Stat. P.

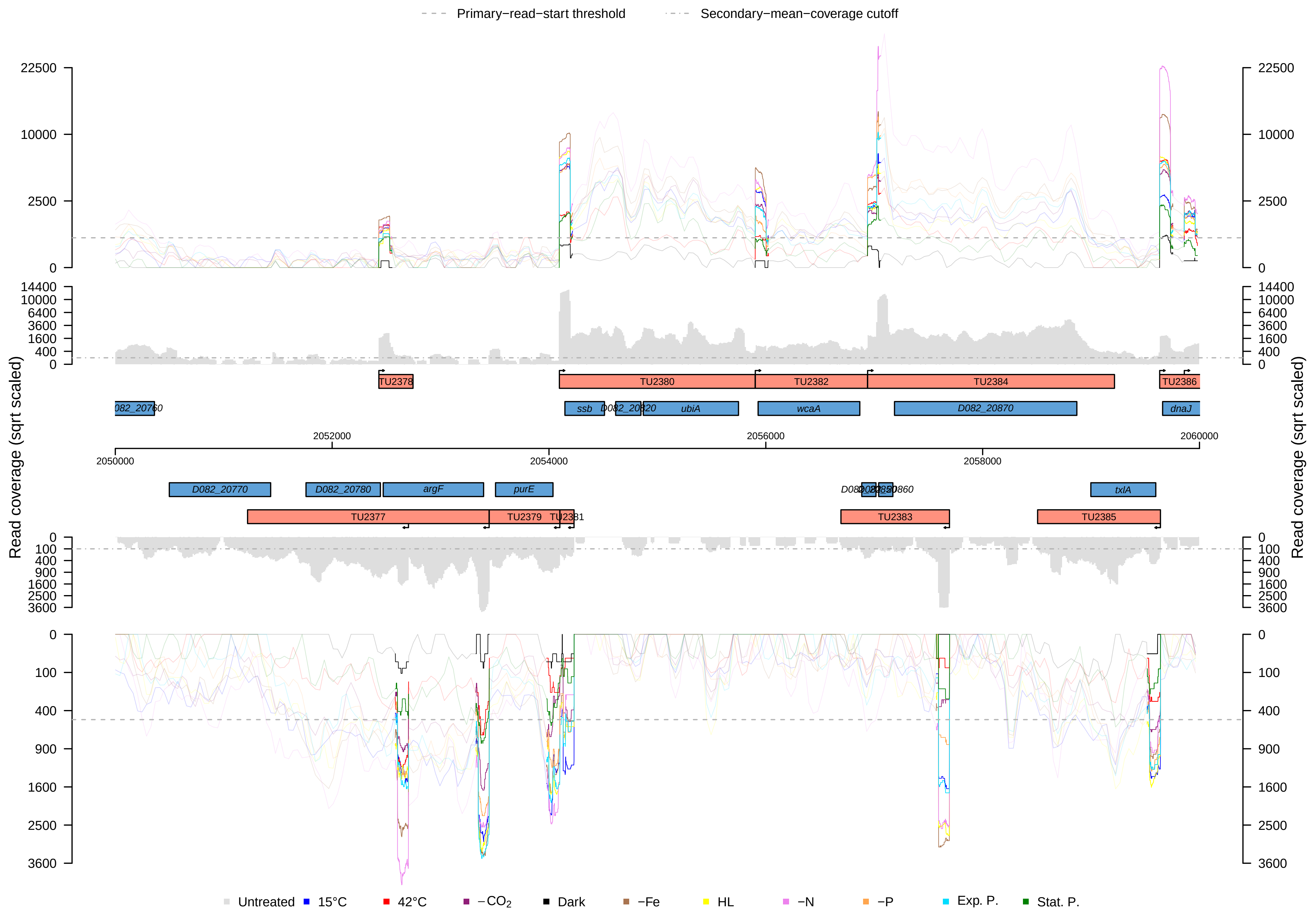

Primary−read−start threshold
Secondary−mean−coverage cutoff
Read coverage (sqrt scaled)
22500
10000
2500
0
14400
10000
6400
3600
1600
400
0
TU2378
TU2380
TU2382
TU2384
TU2386
082_20760
ssb
D082_20820
ubiA
wcaA
D082_20870
dnaJ
2050000
2052000
2054000
2056000
2058000
2060000
D082_20770
D082_20780
argF
purE
txlA
TU2377
TU2379
TU2381
TU2383
TU2385
100
900
Untreated
15°C
42°C
−CO_2
Dark
−Fe
HL
−N
−P
Exp. P.
Stat. P.

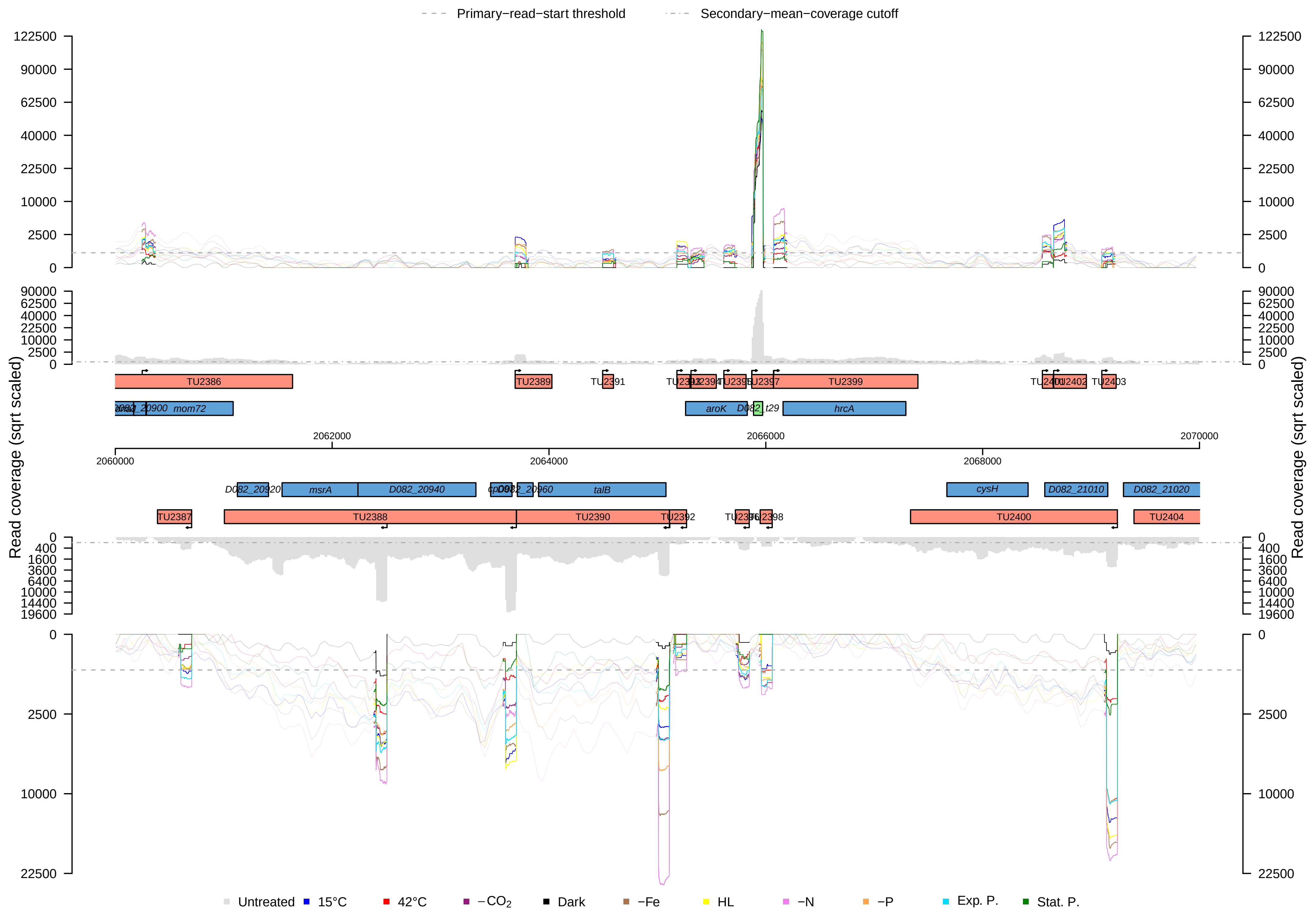

Primary−read−start threshold
Secondary−mean−coverage cutoff
Read coverage (sqrt scaled)
122500
90000
62500
40000
22500
10000
2500
0
TU2386
TU2389
TU2391
TU2399
TU2402
TU2403
aroK
hrcA
mom72
2060000
2062000
2064000
2066000
2068000
2070000
msrA
D082_20940
talB
cysH
D082_21010
D082_21020
TU2387
TU2388
TU2390
TU2400
TU2404
400
1600
3600
6400
14400
19600
Untreated
15°C
42°C
−CO_2
Dark
−Fe
HL
−N
−P
Exp. P.
Stat. P.

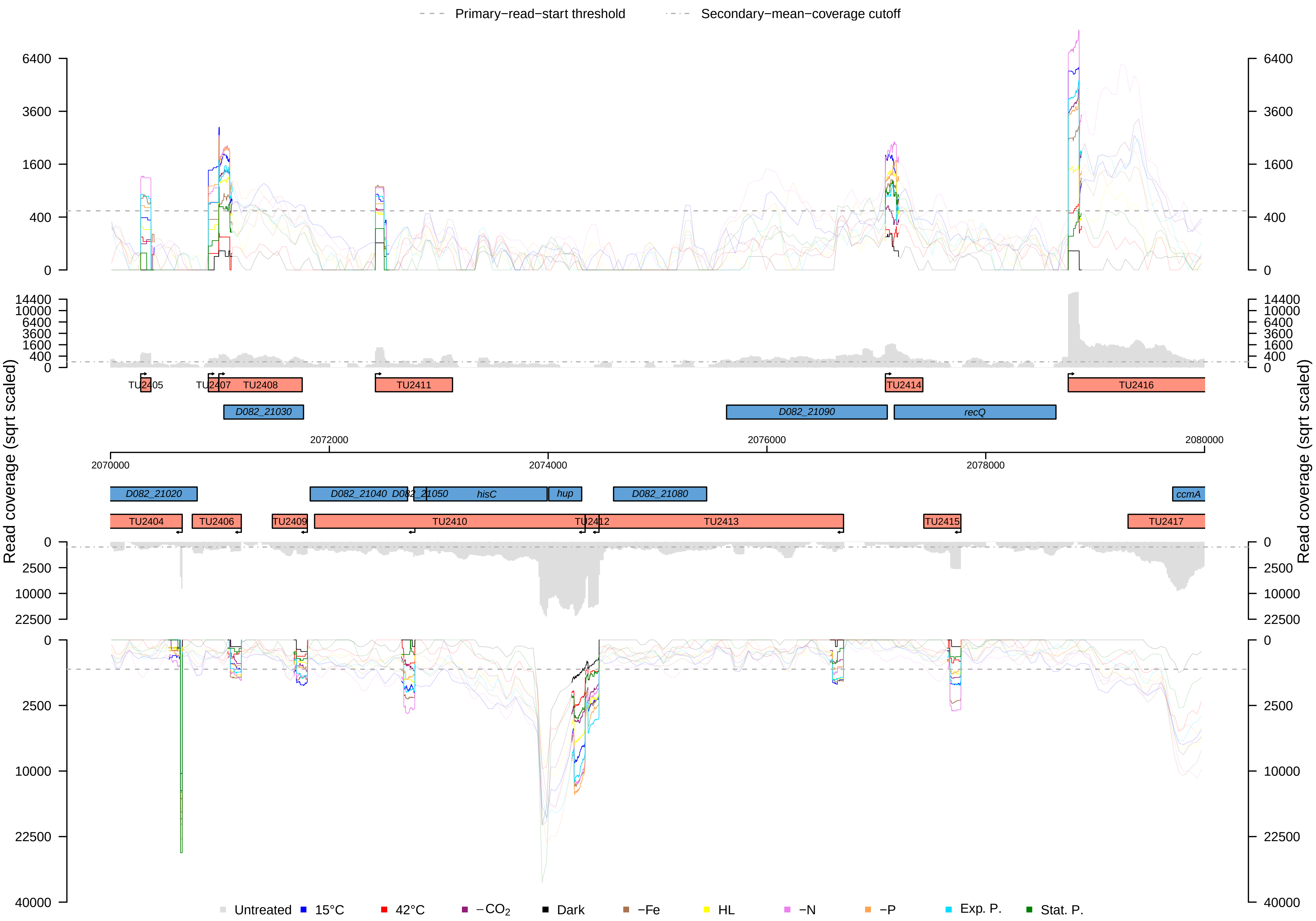

Primary−read−start threshold
Secondary−mean−coverage cutoff
Read coverage (sqrt scaled)
6400
3600
1600
400
0
14400
10000
6400
3600
1600
400
0
TU2405
TU2407
TU2408
TU2411
TU2414
TU2416
D082_21030
D082_21090
recQ
2070000
2072000
2074000
2076000
2078000
2080000
D082_21020
D082_21040
D082_21050
hisC
hup
D082_21080
ccmA
TU2404
TU2406
TU2409
TU2410
TU2412
TU2413
TU2415
TU2417
0
2500
10000
22500
40000
Untreated
15°C
42°C
−CO2
Dark
−Fe
HL
−N
−P
Exp. P.
Stat. P.

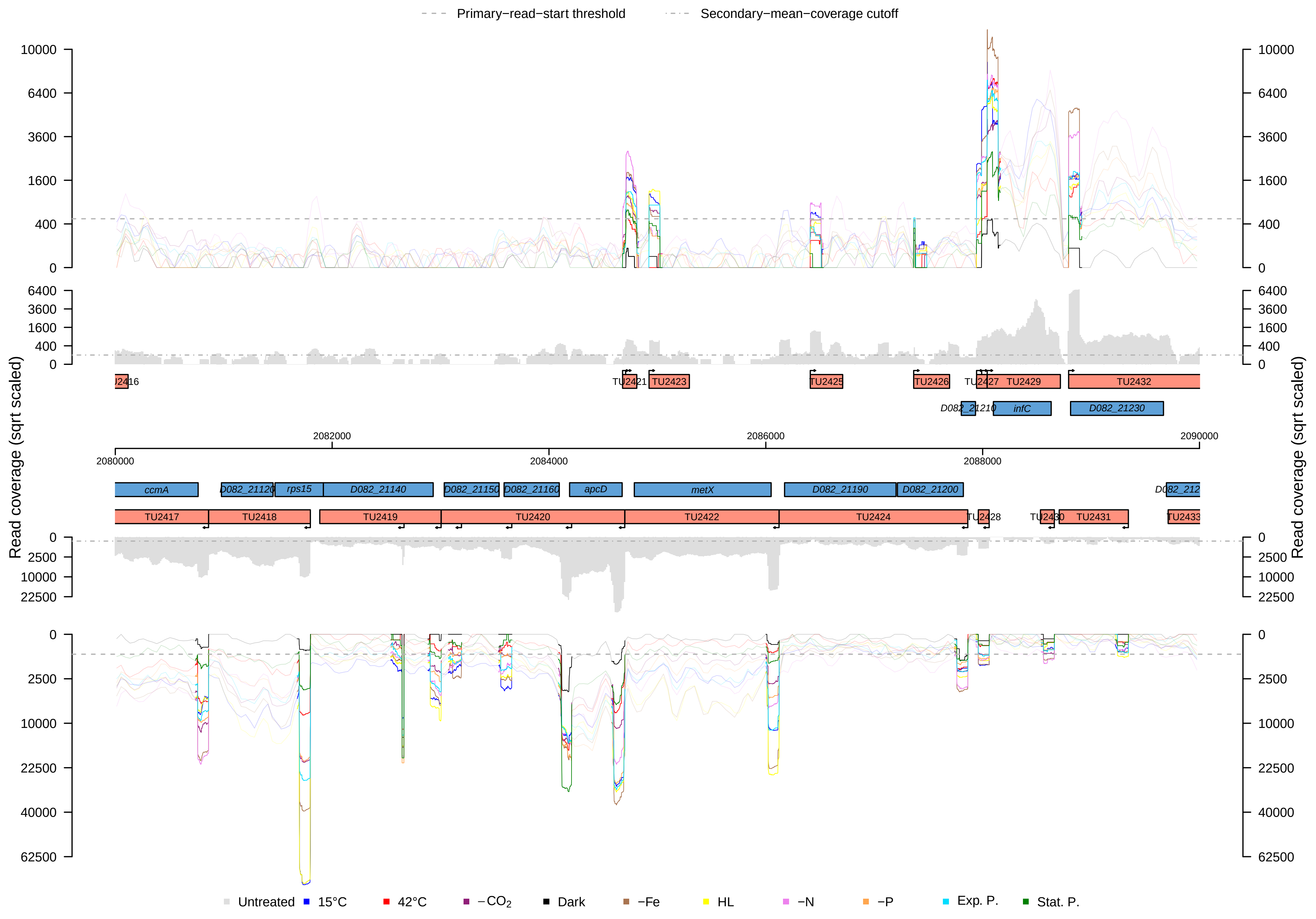

Primary−read−start threshold
Secondary−mean−coverage cutoff
Read coverage (sqrt scaled)
10000
6400
3600
1600
400
0
2500
22500
40000
62500
TU2421
TU2423
TU2425
TU2426
TU2427
TU2429
TU2432
D082_21210
infC
D082_21230
2080000
2082000
2084000
2086000
2088000
2090000
ccmA
D082_21120
rps15
D082_21140
D082_21150
D082_21160
apcD
metX
D082_21190
D082_21200
TU2417
TU2418
TU2419
TU2420
TU2422
TU2424
TU2428
TU2430
TU2431
TU2433
Untreated
15°C
42°C
−CO2
Dark
−Fe
HL
−N
−P
Exp. P.
Stat. P.

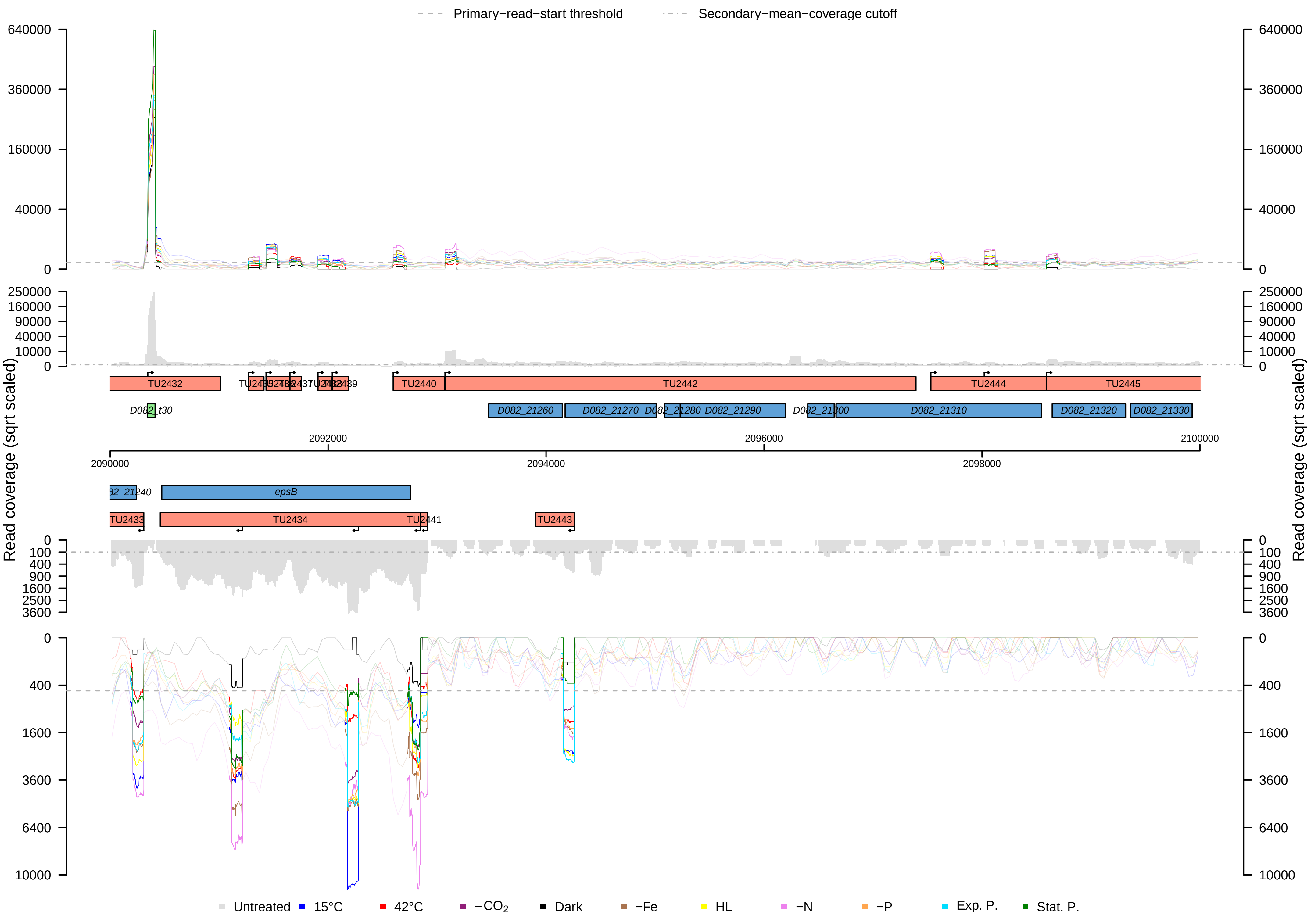

Primary−read−start threshold
Secondary−mean−coverage cutoff
Read coverage (sqrt scaled)
TU2432
TU2440
TU2442
TU2444
TU2445
D082_t30
D082_21260
D082_21270
D082_21290
D082_21310
D082_21320
D082_21330
2090000
2092000
2094000
2096000
2098000
2100000
epsB
TU2433
TU2434
TU2443
Untreated
15°C
42°C
−CO2
Dark
−Fe
HL
−N
−P
Exp. P.
Stat. P.

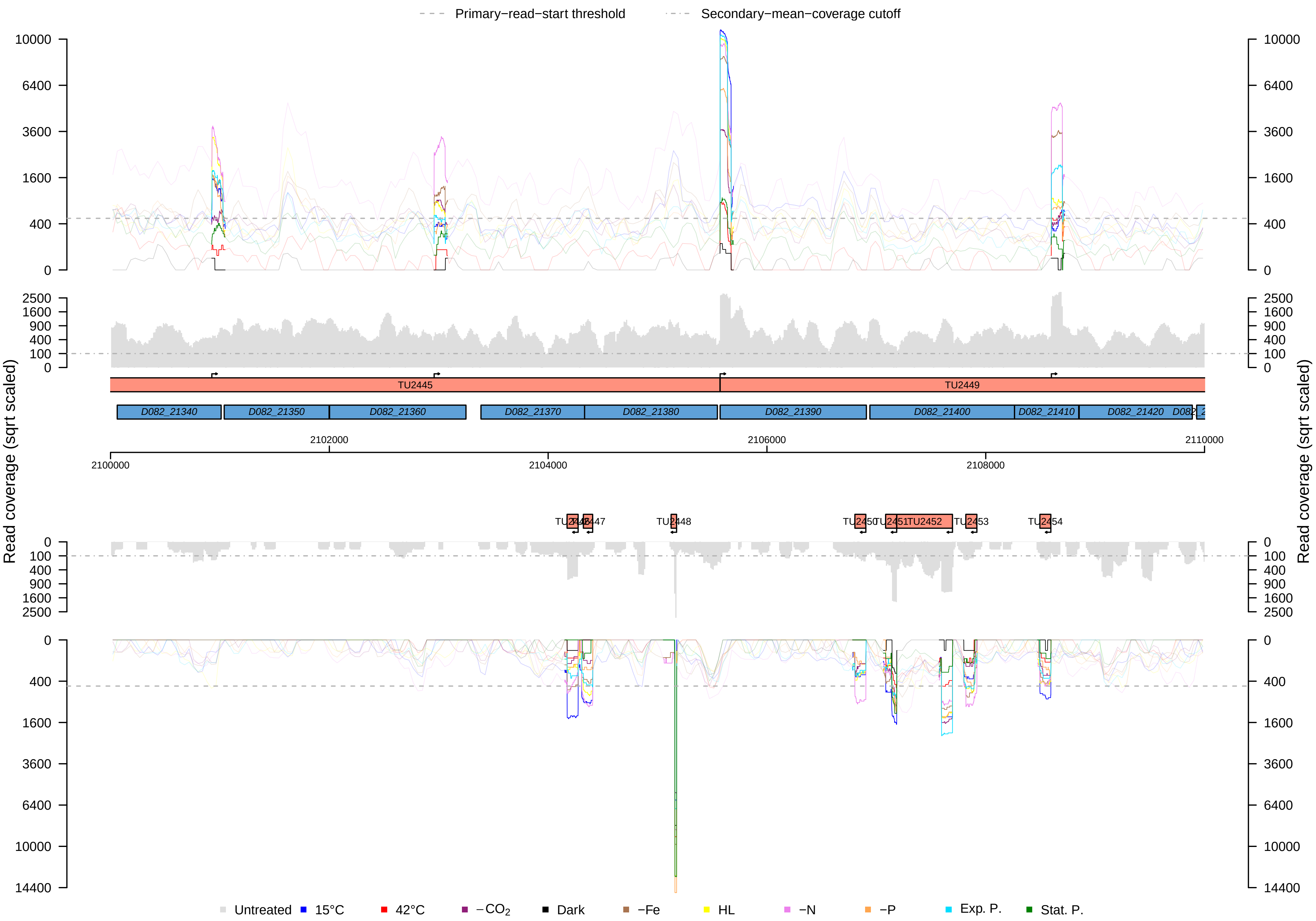

Primary−read−start threshold
Secondary−mean−coverage cutoff
Read coverage (sqrt scaled)
TU2445
TU2449
D082_21340
D082_21350
D082_21360
D082_21370
D082_21380
D082_21390
D082_21400
D082_21410
D082_21420
2100000
2102000
2104000
2106000
2108000
2110000
TU2448
Untreated
15°C
42°C
−CO2
Dark
−Fe
HL
−N
−P
Exp. P.
Stat. P.

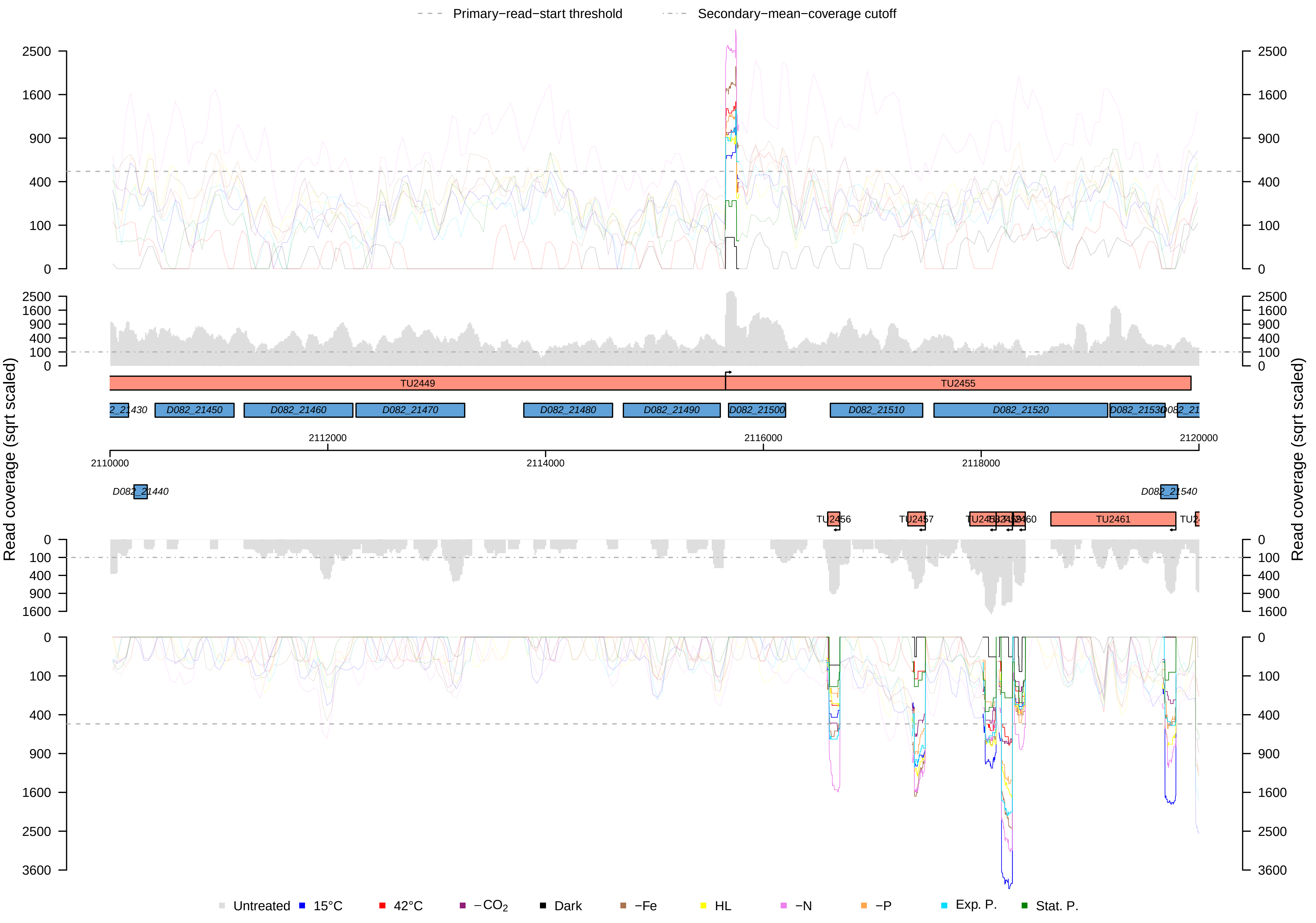

Primary−read−start threshold
Secondary−mean−coverage cutoff
Read coverage (sqrt scaled)
TU2449
TU2455
D082_21450
D082_21460
D082_21470
D082_21480
D082_21490
D082_21500
D082_21510
D082_21520
D082_21440
D082_21540
TU2456
TU2457
TU2461
2110000
2112000
2114000
2116000
2118000
2120000
Untreated
15°C
42°C
−CO2
Dark
−Fe
HL
−N
−P
Exp. P.
Stat. P.

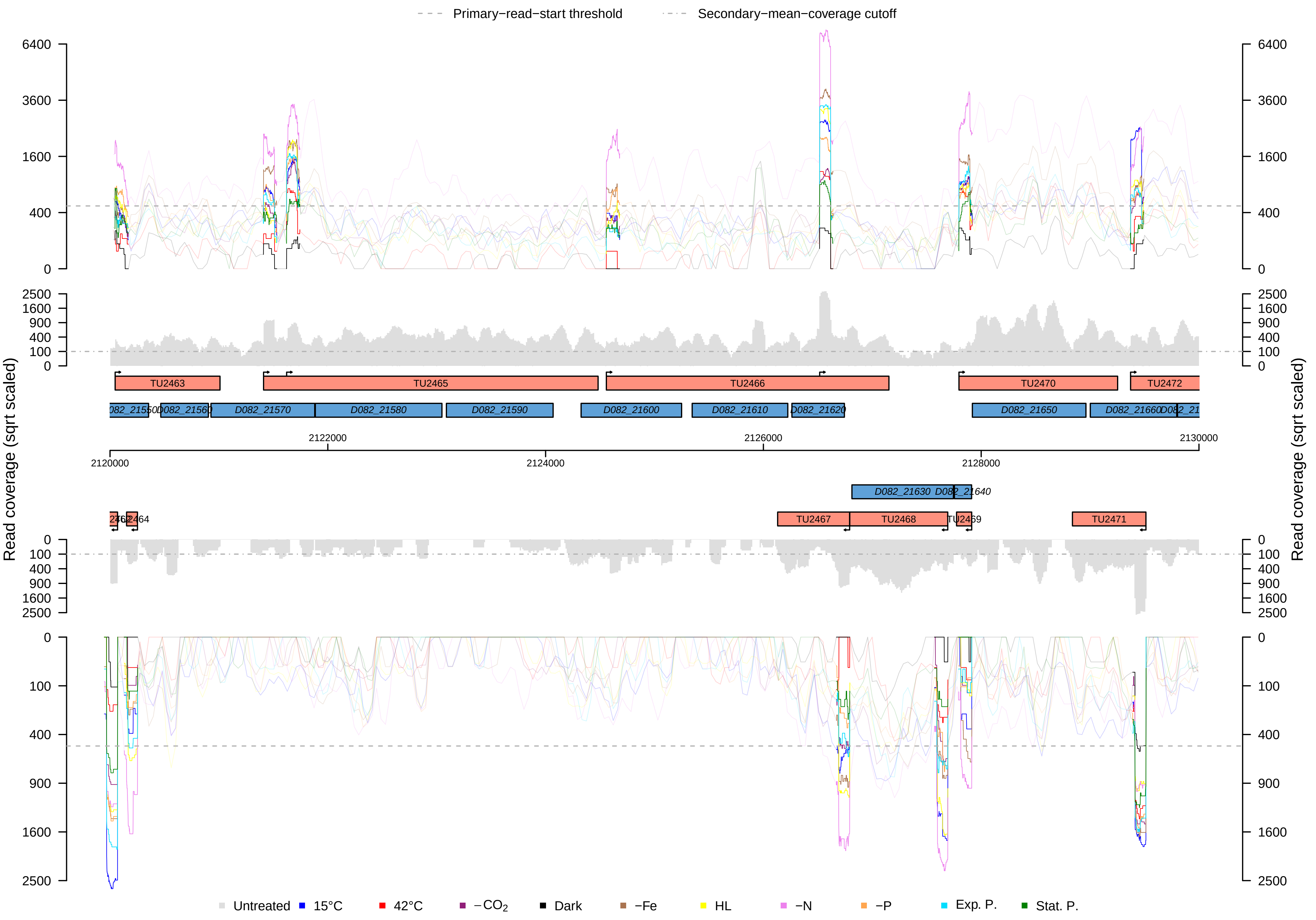

Primary−read−start threshold
Secondary−mean−coverage cutoff
Read coverage (sqrt scaled)
6400
3600
1600
400
0
2500
1600
900
400
100
0
TU2463
TU2465
TU2466
TU2470
TU2472
D082_21570
D082_21580
D082_21590
D082_21600
D082_21610
D082_21650
2120000
2122000
2124000
2126000
2128000
2130000
D082_21630
TU2467
TU2468
TU2471
Untreated
15°C
42°C
−CO2
Dark
−Fe
HL
−N
−P
Exp. P.
Stat. P.

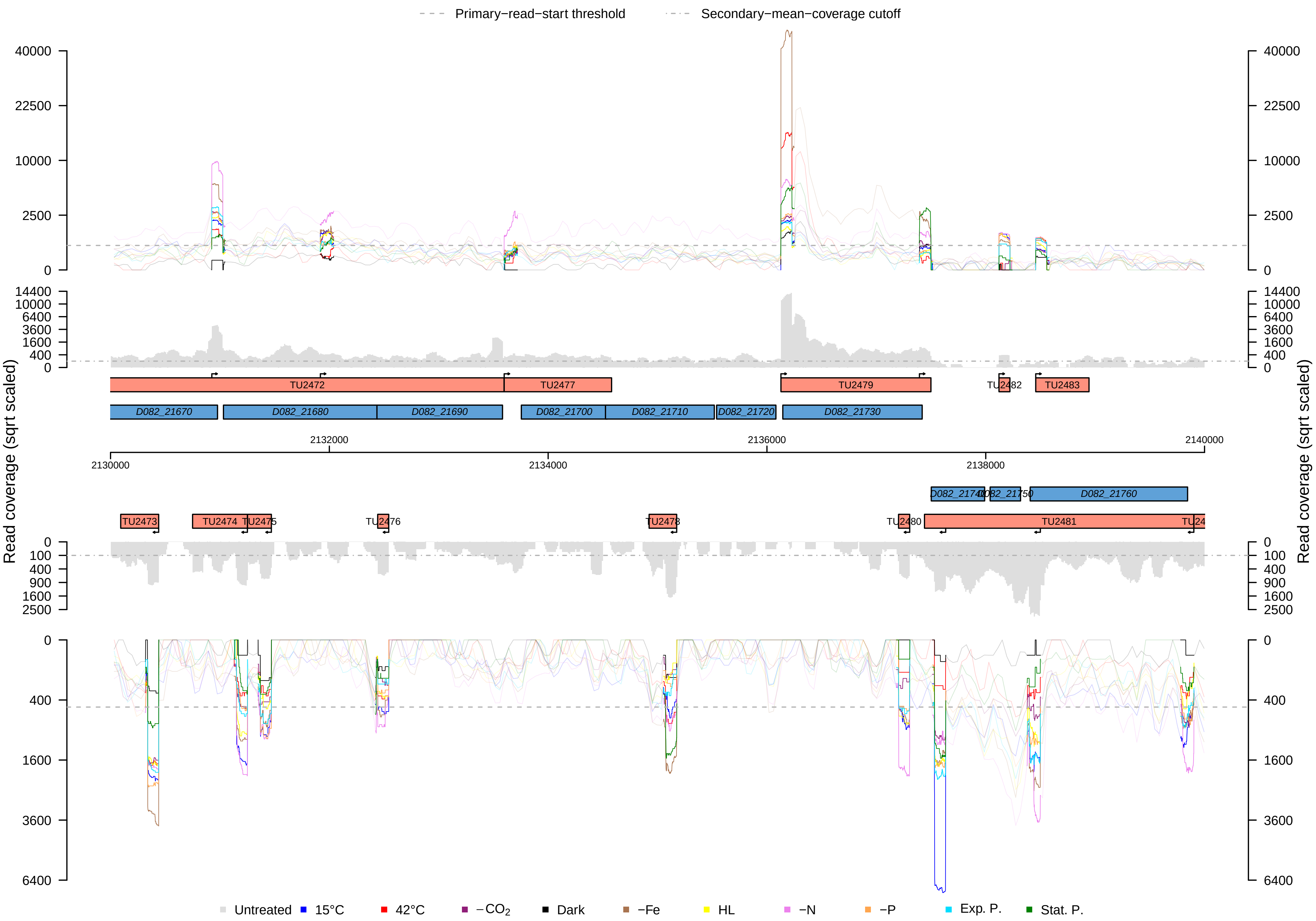

Primary−read−start threshold
Secondary−mean−coverage cutoff
Read coverage (sqrt scaled)
40000
22500
10000
2500
0
14400
10000
6400
3600
1600
400
100
900
2500
3600
6400
TU2472
TU2477
TU2479
TU2482
TU2483
D082_21670
D082_21680
D082_21690
D082_21700
D082_21710
D082_21720
D082_21730
2130000
2132000
2134000
2136000
2138000
2140000
D082_21740
D082_21750
D082_21760
TU2473
TU2474
TU2475
TU2476
TU2478
TU2480
TU2481
Untreated
15°C
42°C
−CO2
Dark
−Fe
HL
−N
−P
Exp. P.
Stat. P.

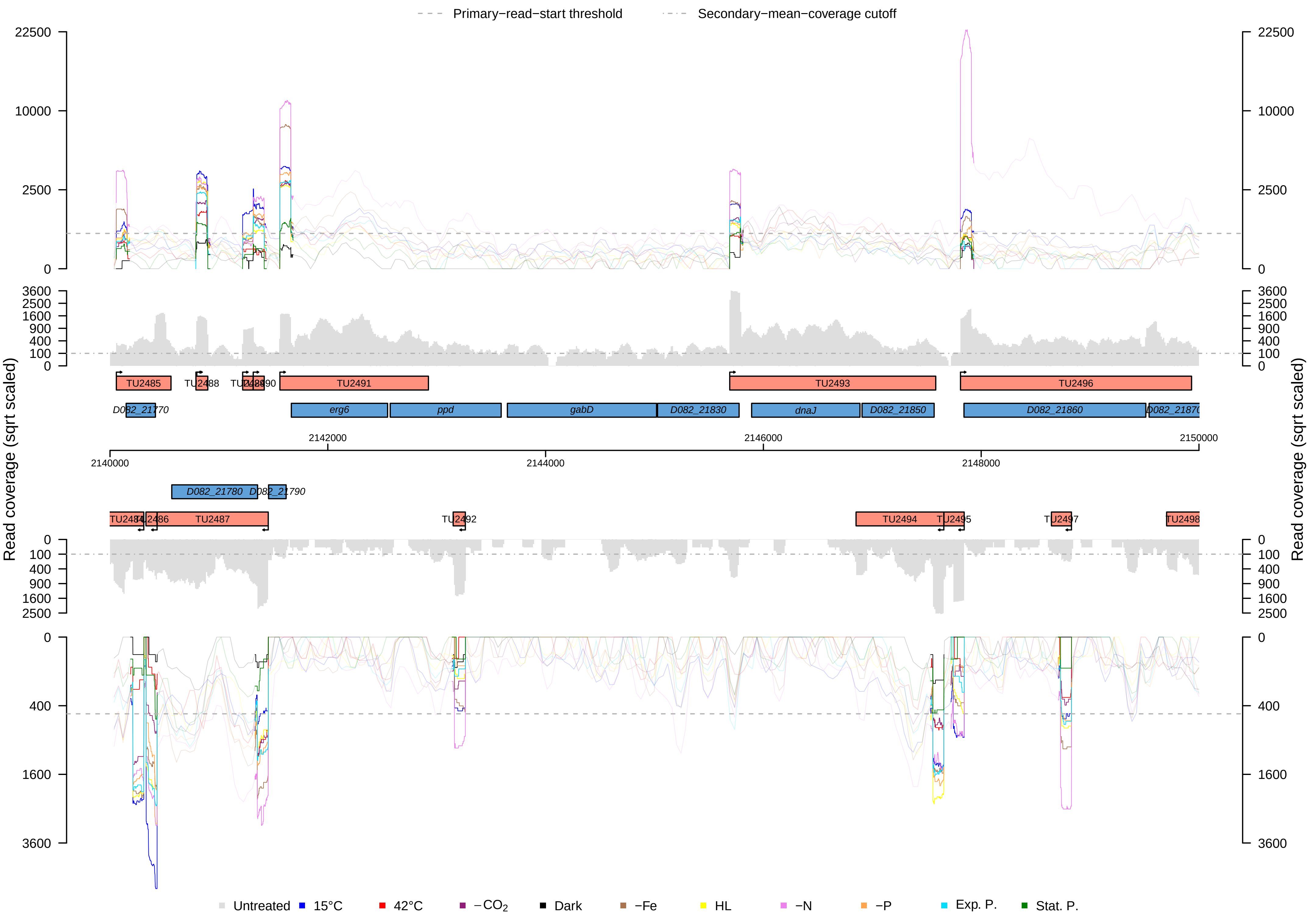

Primary−read−start threshold
Secondary−mean−coverage cutoff
Read coverage (sqrt scaled)
TU2485
TU2488
TU2491
TU2493
TU2496
D082_21770
erg6
ppd
gabD
D082_21830
dnaJ
D082_21850
D082_21860
D082_21780
TU2487
TU2492
TU2494
TU2497
TU2498
Untreated
15°C
42°C
−CO2
Dark
−Fe
HL
−N
−P
Exp. P.
Stat. P.

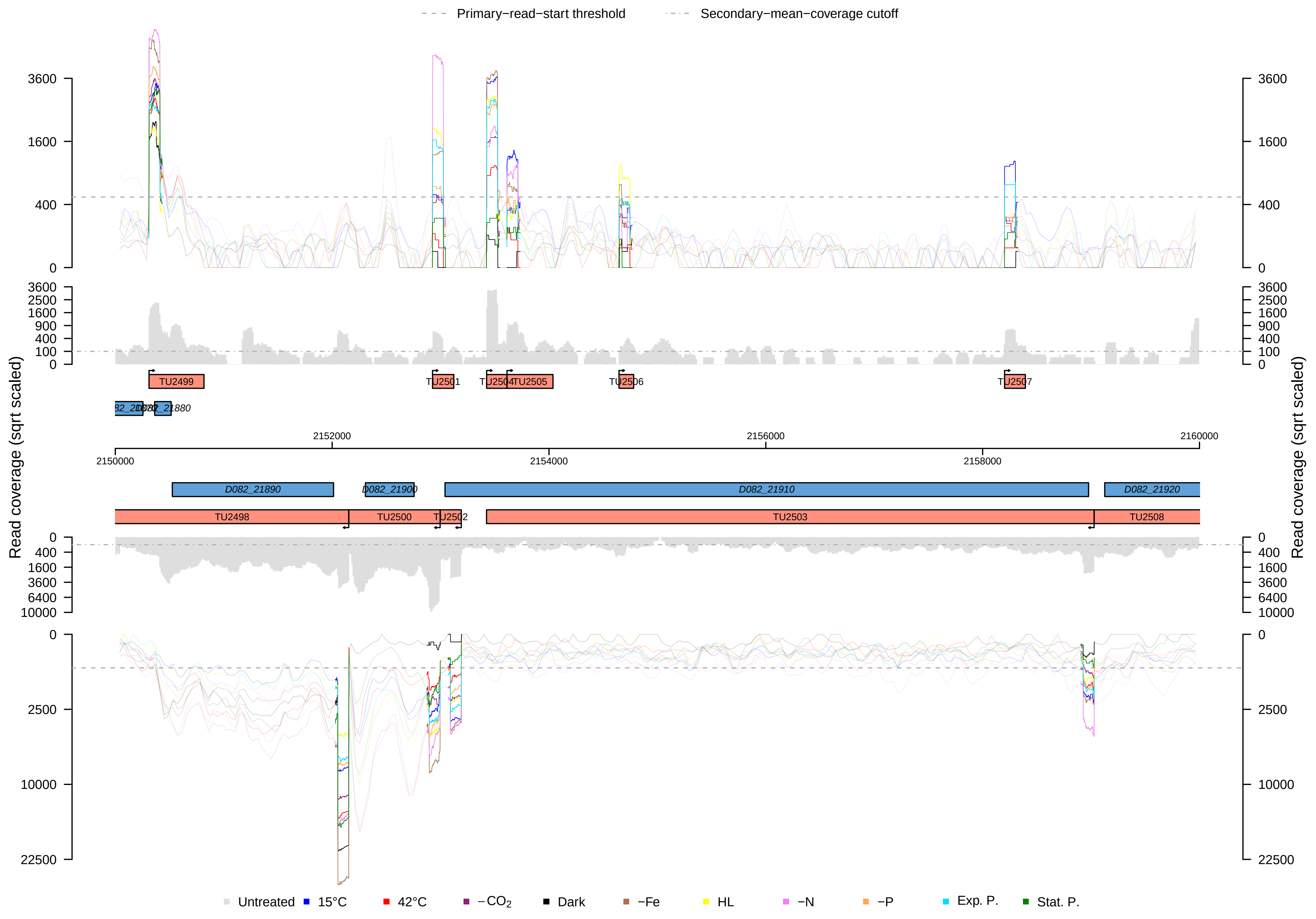

Primary−read−start threshold
Secondary−mean−coverage cutoff
Read coverage (sqrt scaled)
3600
1600
400
0
2500
900
100
TU2499
TU2501
TU2504
TU2505
TU2506
TU2507
D082_21880
2150000
2152000
2154000
2156000
2158000
2160000
D082_21890
D082_21900
D082_21910
D082_21920
TU2498
TU2500
TU2502
TU2503
TU2508
6400
10000
22500
Untreated
15°C
42°C
−CO2
Dark
−Fe
HL
−N
−P
Exp. P.
Stat. P.

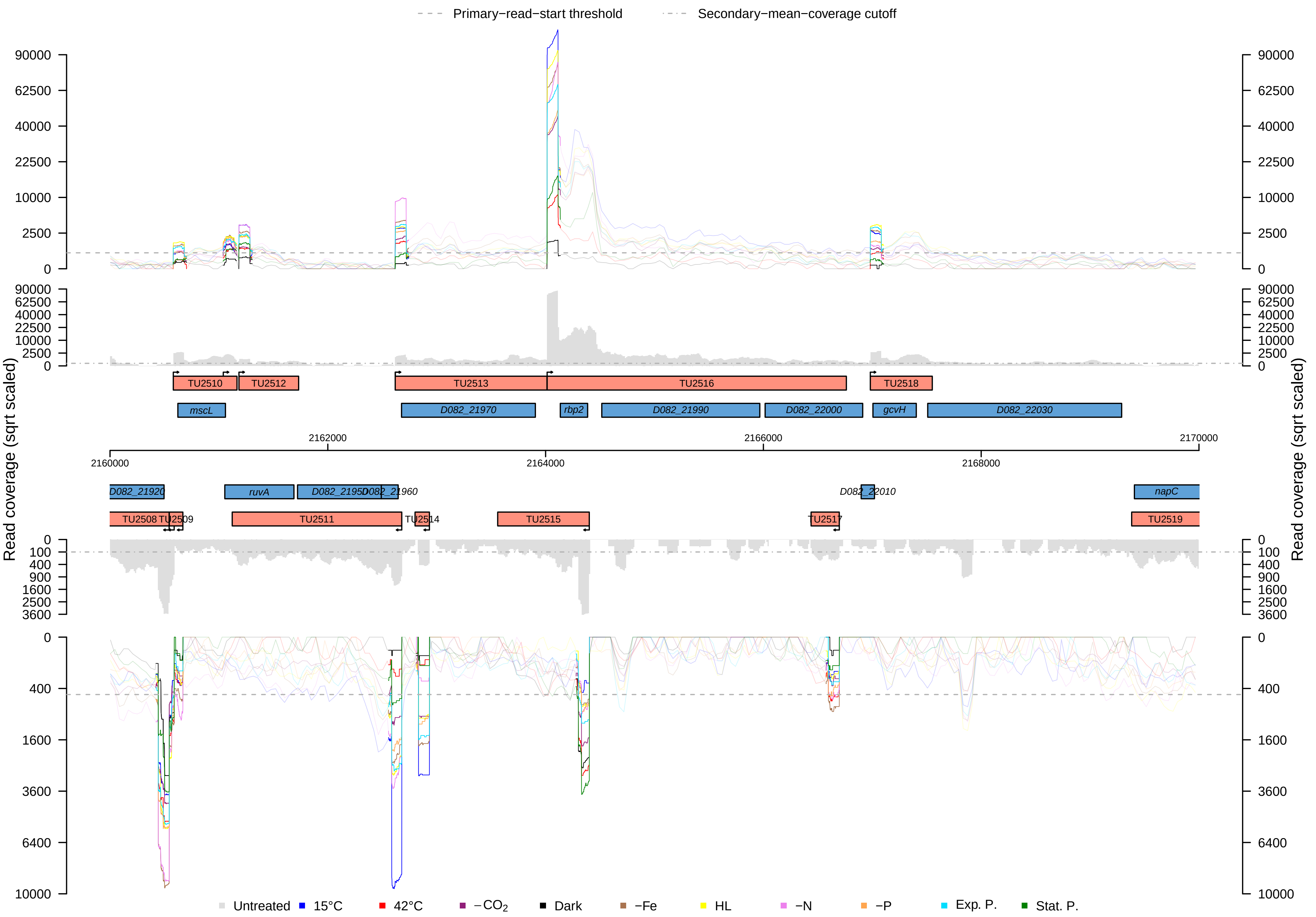

Primary−read−start threshold
Secondary−mean−coverage cutoff
Read coverage (sqrt scaled)
90000
62500
40000
22500
10000
2500
0
TU2510
TU2512
TU2513
TU2516
TU2518
mscL
D082_21970
rbp2
D082_21990
D082_22000
gcvH
D082_22030
2160000
2162000
2164000
2166000
2168000
2170000
D082_21920
ruvA
D082_21950
D082_21960
D082_22010
napC
TU2508
TU2509
TU2511
TU2514
TU2515
TU2517
TU2519
100
400
900
1600
2500
3600
6400
10000
Untreated
15°C
42°C
−CO2
Dark
−Fe
HL
−N
−P
Exp. P.
Stat. P.

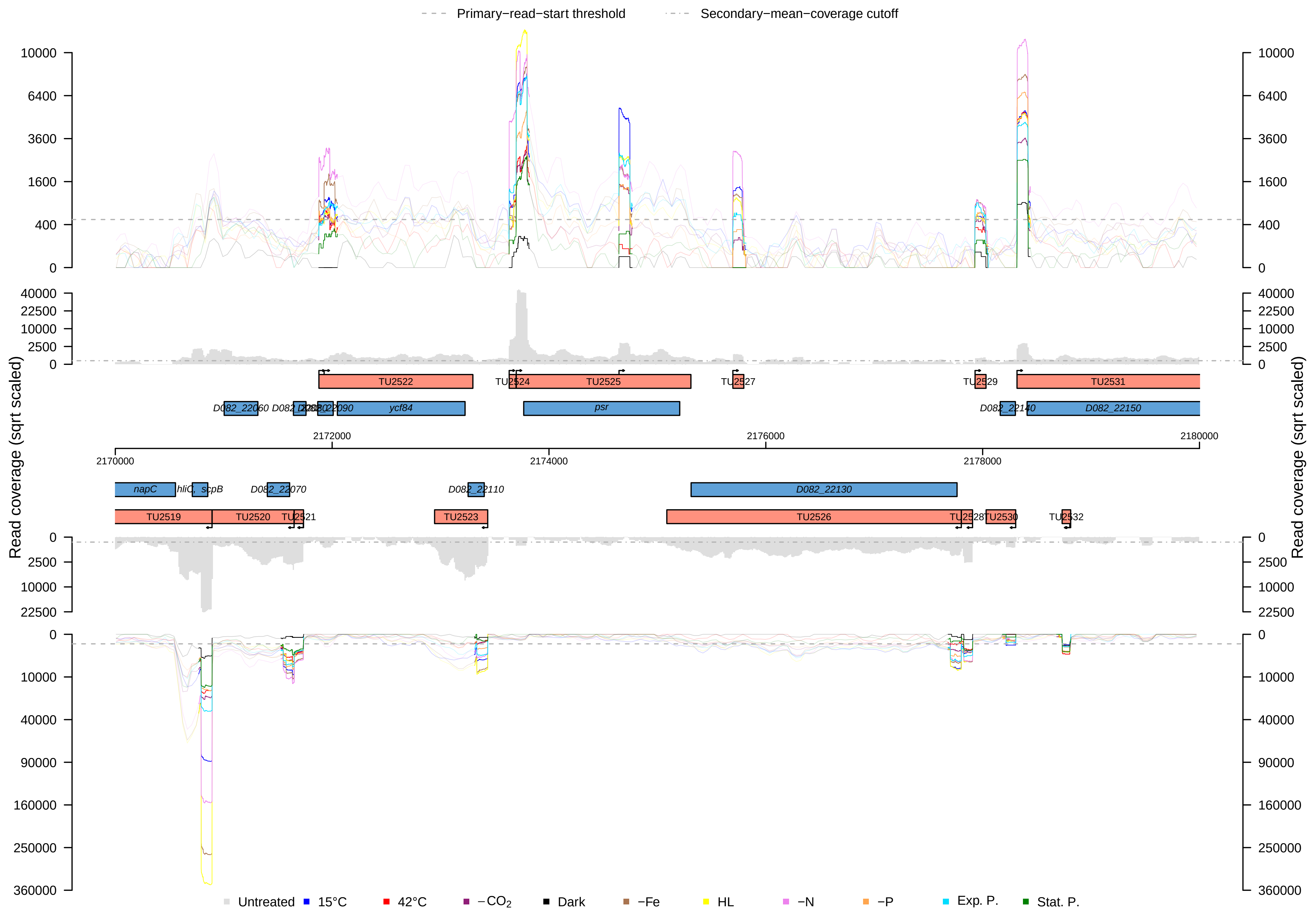

Primary−read−start threshold
Secondary−mean−coverage cutoff
Read coverage (sqrt scaled)
10000
6400
3600
1600
400
0
40000
22500
10000
2500
0
TU2522
TU2524
TU2525
TU2527
TU2529
TU2531
D082_22060
D082_22080
D082_22090
ycf84
psr
D082_22140
D082_22150
2170000
2172000
2174000
2176000
2178000
2180000
napC
hliC
scpB
D082_22070
D082_22110
D082_22130
TU2519
TU2520
TU2521
TU2523
TU2526
TU2528
TU2530
TU2532
90000
160000
250000
360000
Untreated
15°C
42°C
−CO2
Dark
−Fe
HL
−N
−P
Exp. P.
Stat. P.

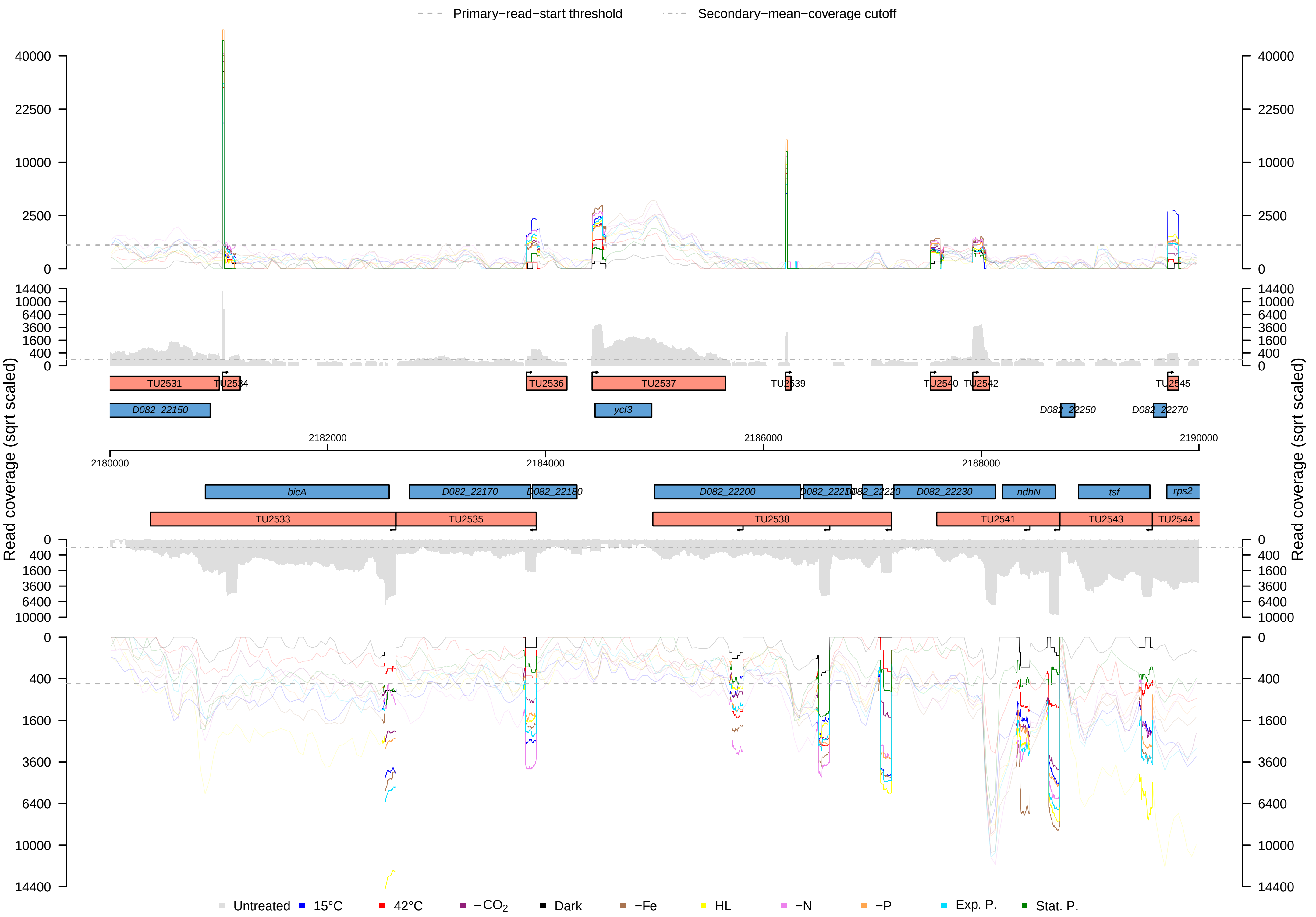

Primary−read−start threshold
Secondary−mean−coverage cutoff
Read coverage (sqrt scaled)
40000
22500
10000
2500
0
14400
10000
6400
3600
1600
400
TU2531
TU2534
TU2536
TU2537
TU2539
TU2540
TU2542
TU2545
D082_22150
ycf3
D082_22250
D082_22270
2180000
2182000
2184000
2186000
2188000
2190000
bicA
D082_22170
D082_22180
D082_22200
D082_22210
D082_22220
D082_22230
ndhN
tsf
rps2
TU2533
TU2535
TU2538
TU2541
TU2543
TU2544
Untreated
15°C
42°C
−CO2
Dark
−Fe
HL
−N
−P
Exp. P.
Stat. P.

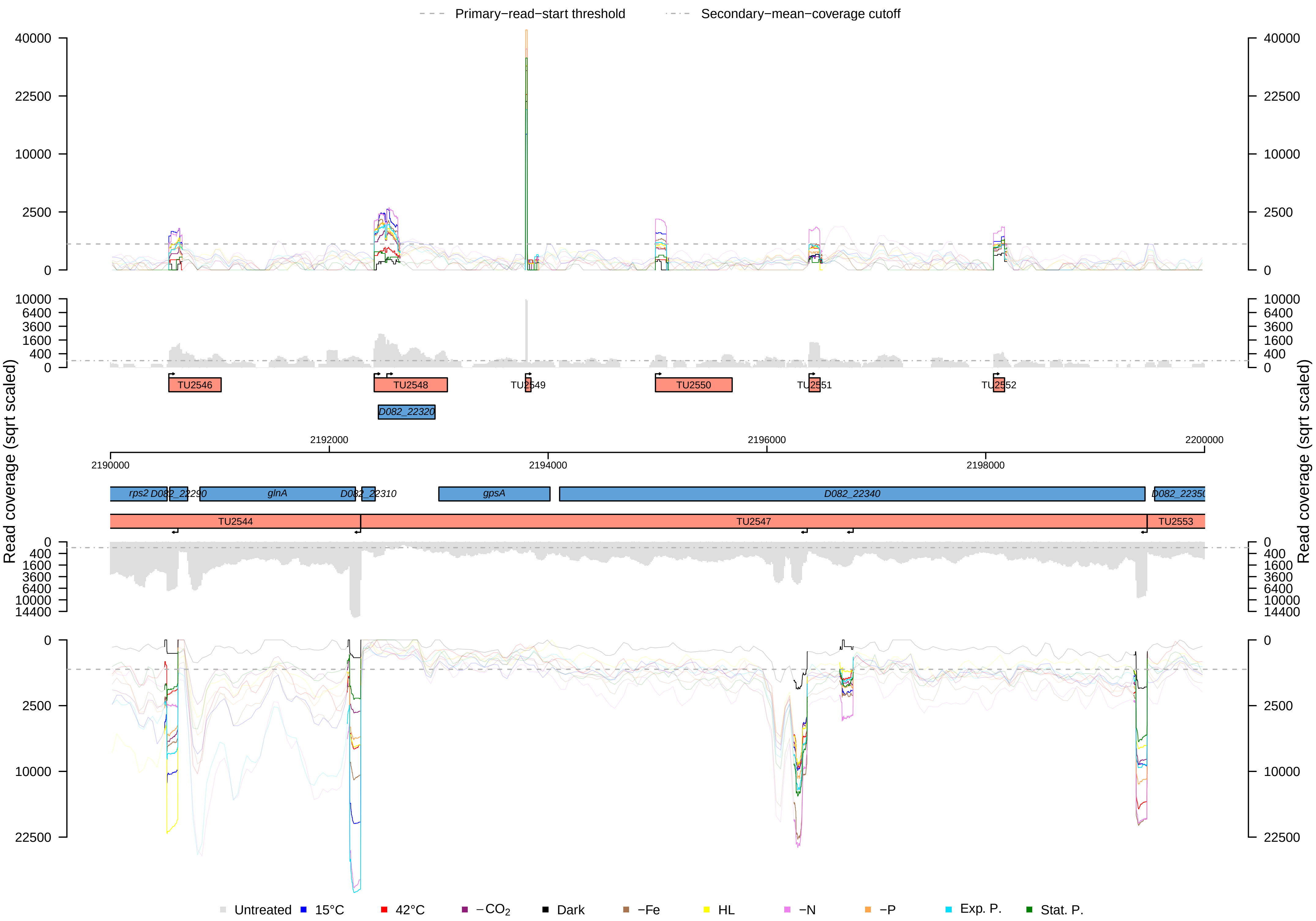

Primary−read−start threshold
Secondary−mean−coverage cutoff
Read coverage (sqrt scaled)
40000
22500
10000
2500
0
10000
6400
3600
1600
400
0
TU2546
TU2548
TU2549
TU2550
TU2551
TU2552
D082_22320
2192000
2196000
2200000
2190000
2194000
2198000
rps2
D082_22290
glnA
D082_22310
gpsA
D082_22340
D082_22350
TU2544
TU2547
TU2553
14400
Untreated
15°C
42°C
−CO2
Dark
−Fe
HL
−N
−P
Exp. P.
Stat. P.

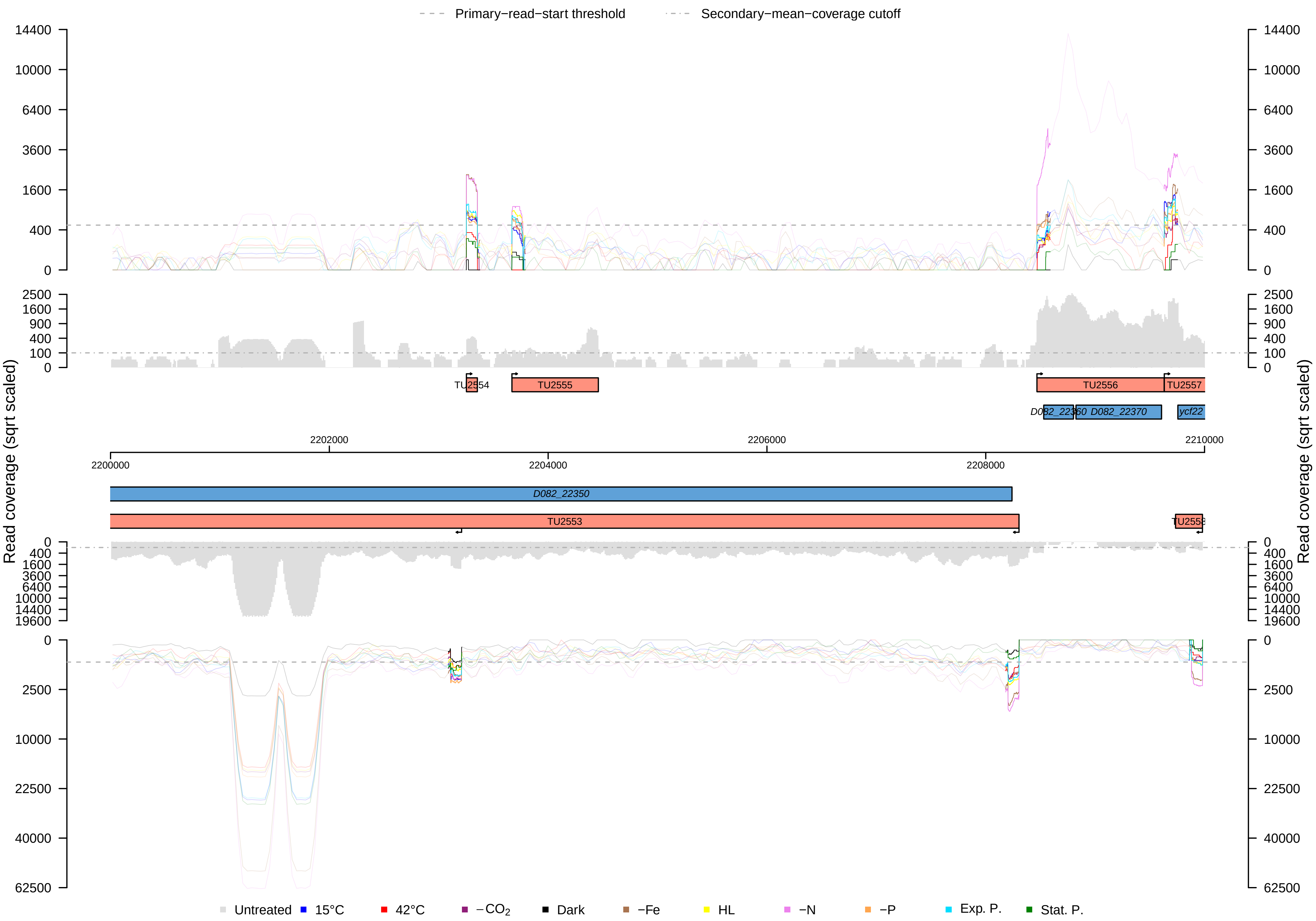

Primary−read−start threshold
Secondary−mean−coverage cutoff
Read coverage (sqrt scaled)
TU2554
TU2555
TU2556
TU2557
D082_22360
D082_22370
ycf22
D082_22350
TU2553
TU2558
2200000
2202000
2204000
2206000
2208000
2210000
Untreated
15°C
42°C
−CO2
Dark
−Fe
HL
−N
−P
Exp. P.
Stat. P.

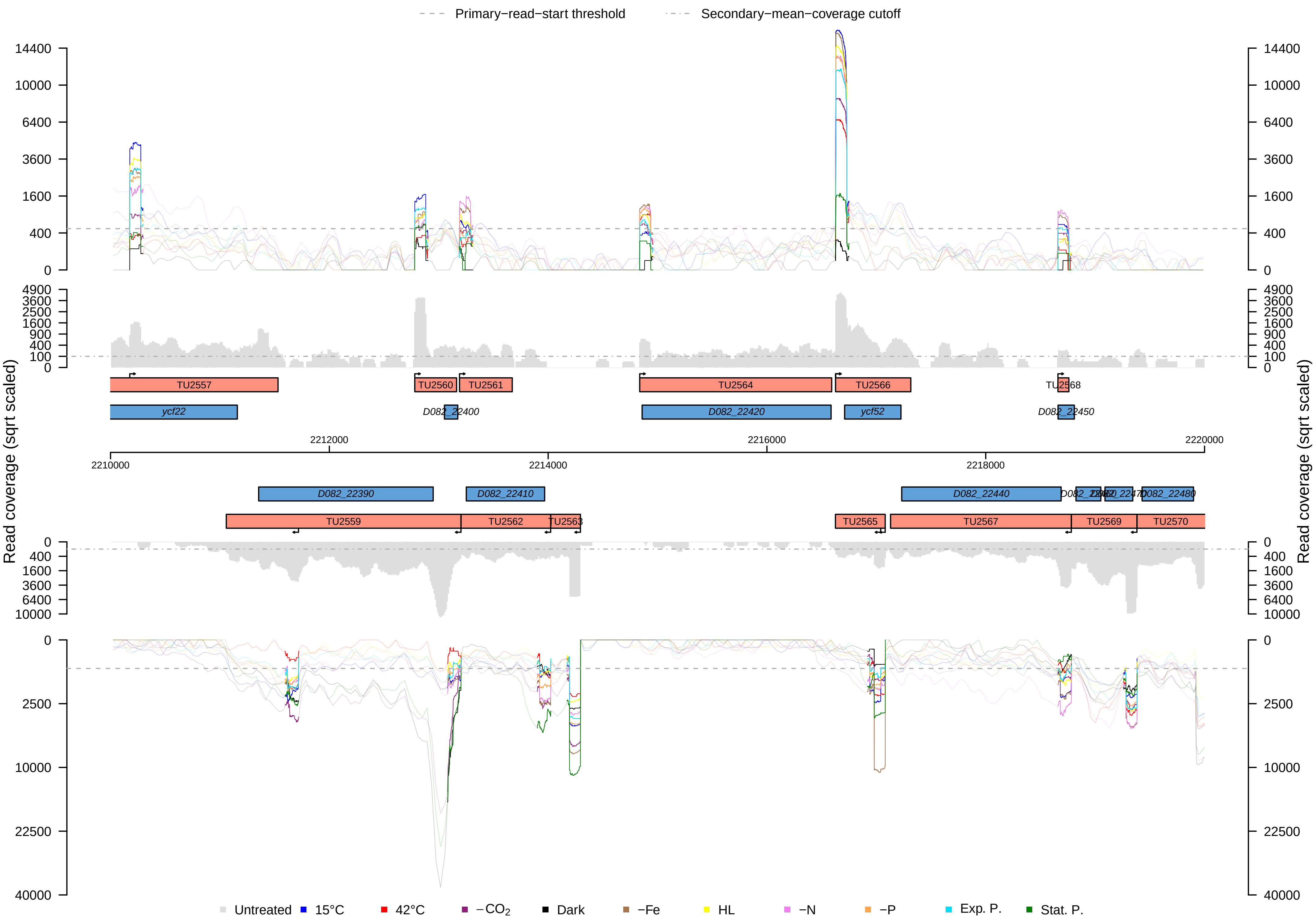

Primary−read−start threshold
Secondary−mean−coverage cutoff
Read coverage (sqrt scaled)
TU2557
TU2560
TU2561
TU2564
TU2566
TU2568
ycf22
D082_22400
D082_22420
ycf52
D082_22450
2210000
2212000
2214000
2216000
2218000
2220000
D082_22390
D082_22410
D082_22440
D082_22480
TU2559
TU2562
TU2563
TU2565
TU2567
TU2569
TU2570
Untreated
15°C
42°C
−CO_2
Dark
−Fe
HL
−N
−P
Exp. P.
Stat. P.

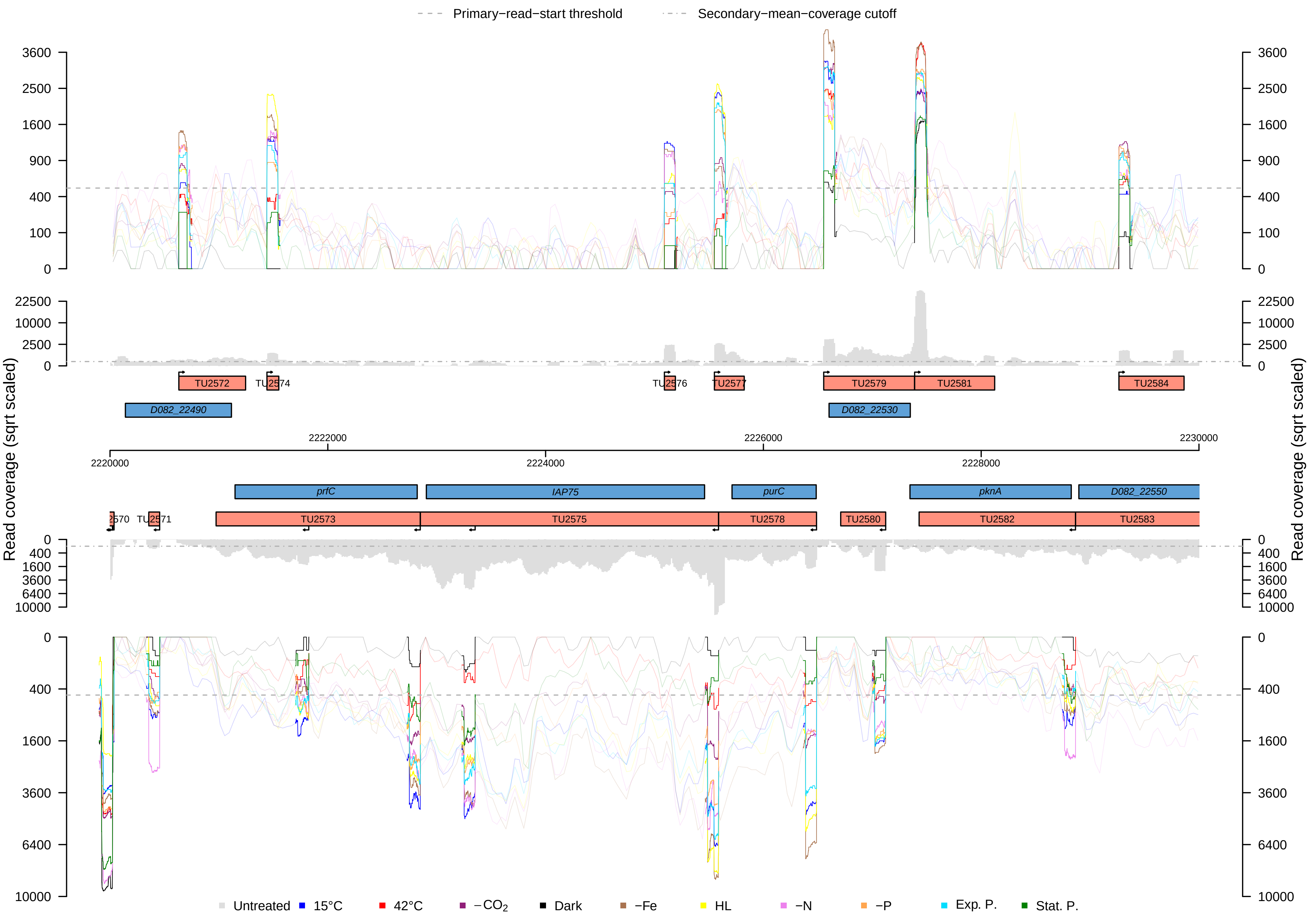

Primary−read−start threshold
Secondary−mean−coverage cutoff
Read coverage (sqrt scaled)
3600
2500
1600
900
400
100
0
22500
10000
2500
0
TU2572
TU2574
TU2576
TU2577
TU2579
TU2581
TU2584
D082_22490
D082_22530
2220000
2222000
2224000
2226000
2228000
2230000
prfC
IAP75
purC
pknA
D082_22550
2570
TU2571
TU2573
TU2575
TU2578
TU2580
TU2582
TU2583
400
1600
3600
6400
10000
Untreated
15°C
42°C
−CO_2
Dark
−Fe
HL
−N
−P
Exp. P.
Stat. P.

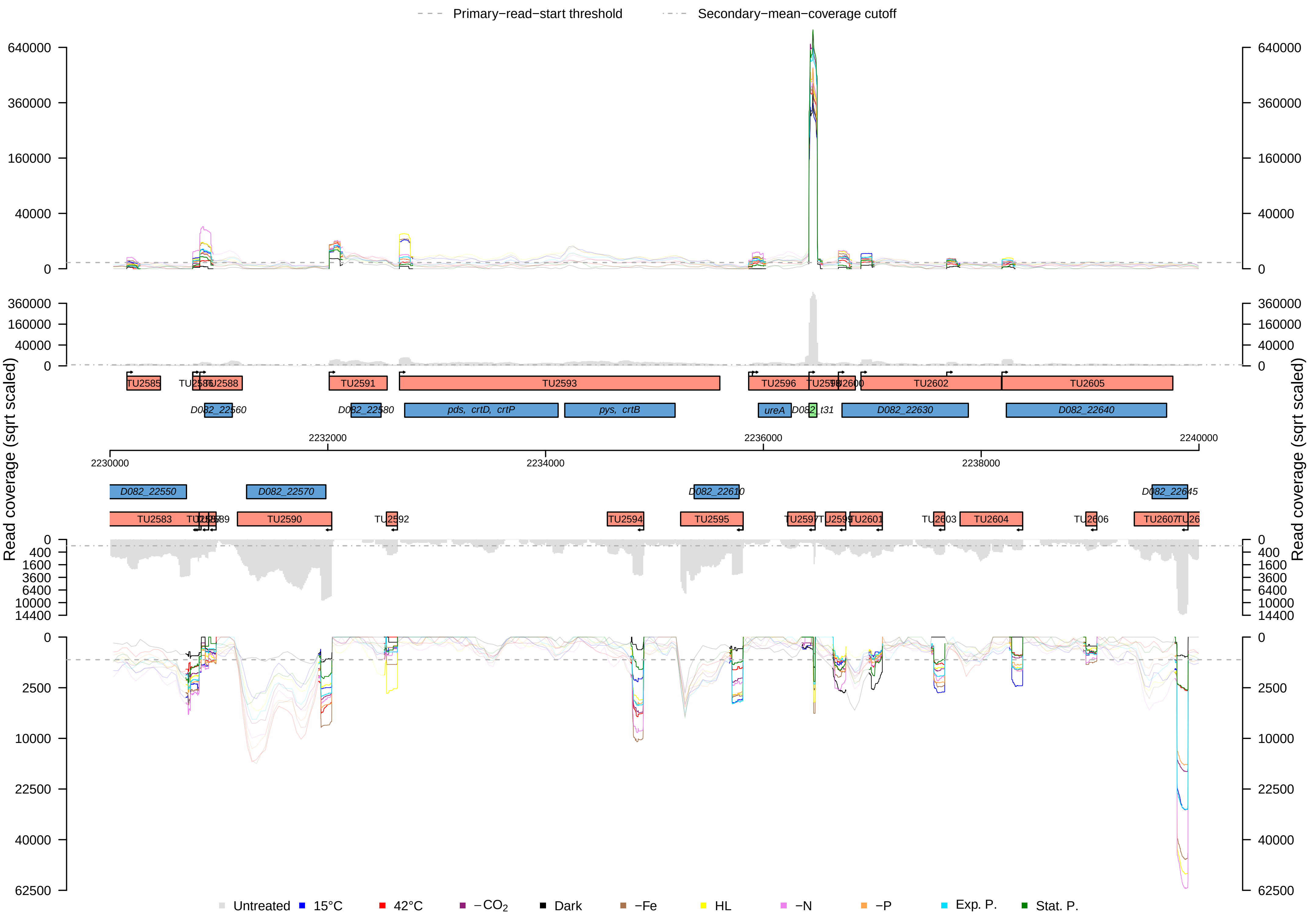

Primary−read−start threshold
Secondary−mean−coverage cutoff
Read coverage (sqrt scaled)
640000
360000
160000
40000
0
TU2585
TU2588
TU2591
TU2593
TU2596
TU2602
TU2605
D082_22560
D082_22580
pds, crtD, crtP
pys, crtB
ureA
D082_22630
D082_22640
2230000
2232000
2234000
2236000
2238000
2240000
D082_22550
D082_22570
D082_22610
D082_22645
TU2583
TU2590
TU2594
TU2595
TU2601
TU2604
2500
10000
22500
40000
62500
Untreated
15°C
42°C
−CO2
Dark
−Fe
HL
−N
−P
Exp. P.
Stat. P.

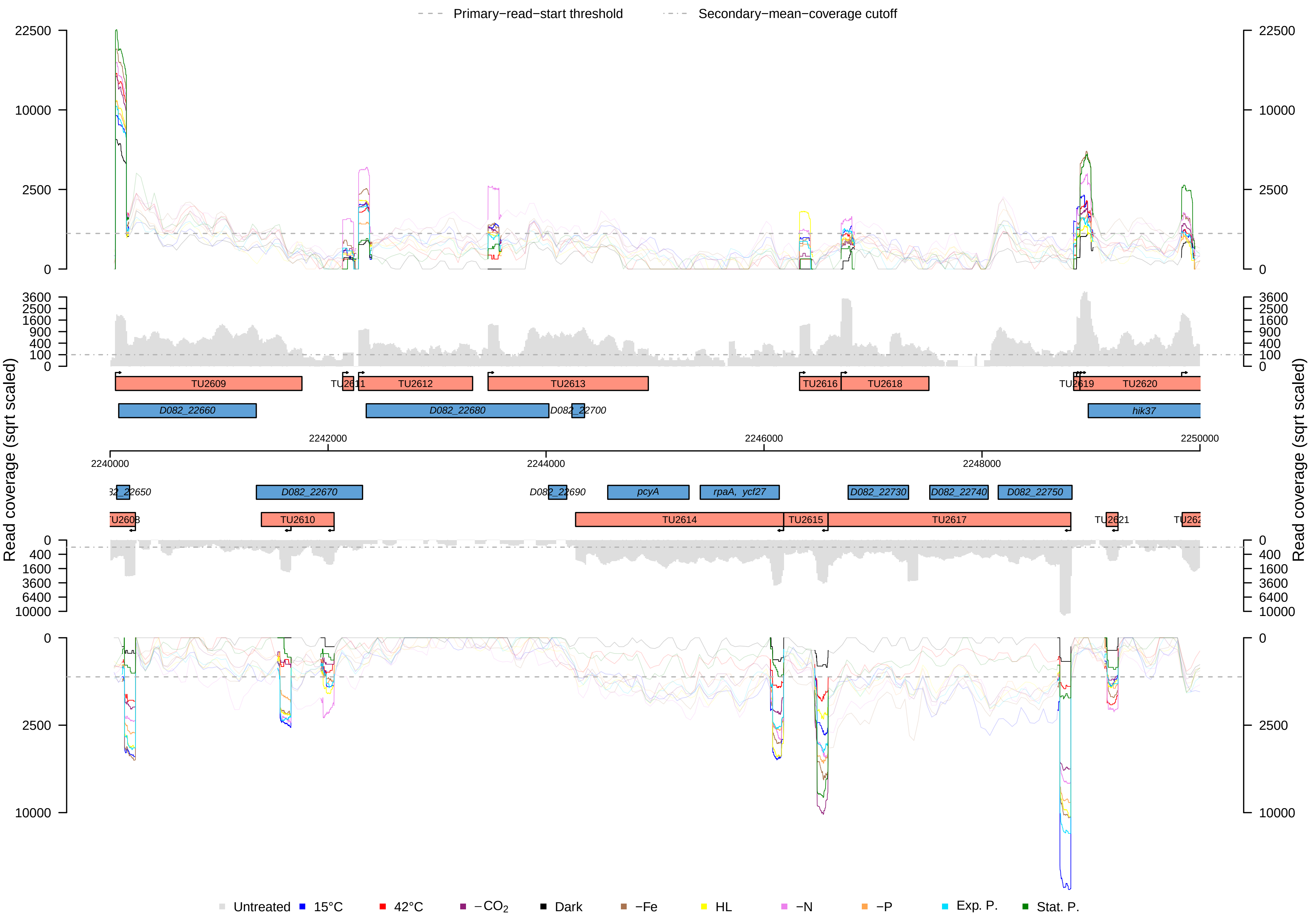

Primary−read−start threshold
Secondary−mean−coverage cutoff
Read coverage (sqrt scaled)
22500
10000
2500
0
3600
2500
1600
900
400
100
TU2609
TU2611
TU2612
TU2613
TU2616
TU2618
TU2619
TU2620
D082_22660
D082_22680
D082_22700
hik37
2240000
2242000
2244000
2246000
2248000
2250000
D082_22650
D082_22670
D082_22690
pcyA
rpaA, ycf27
D082_22730
D082_22740
D082_22750
TU2608
TU2610
TU2614
TU2615
TU2617
TU2621
6400
Untreated
15°C
42°C
−CO2
Dark
−Fe
HL
−N
−P
Exp. P.
Stat. P.

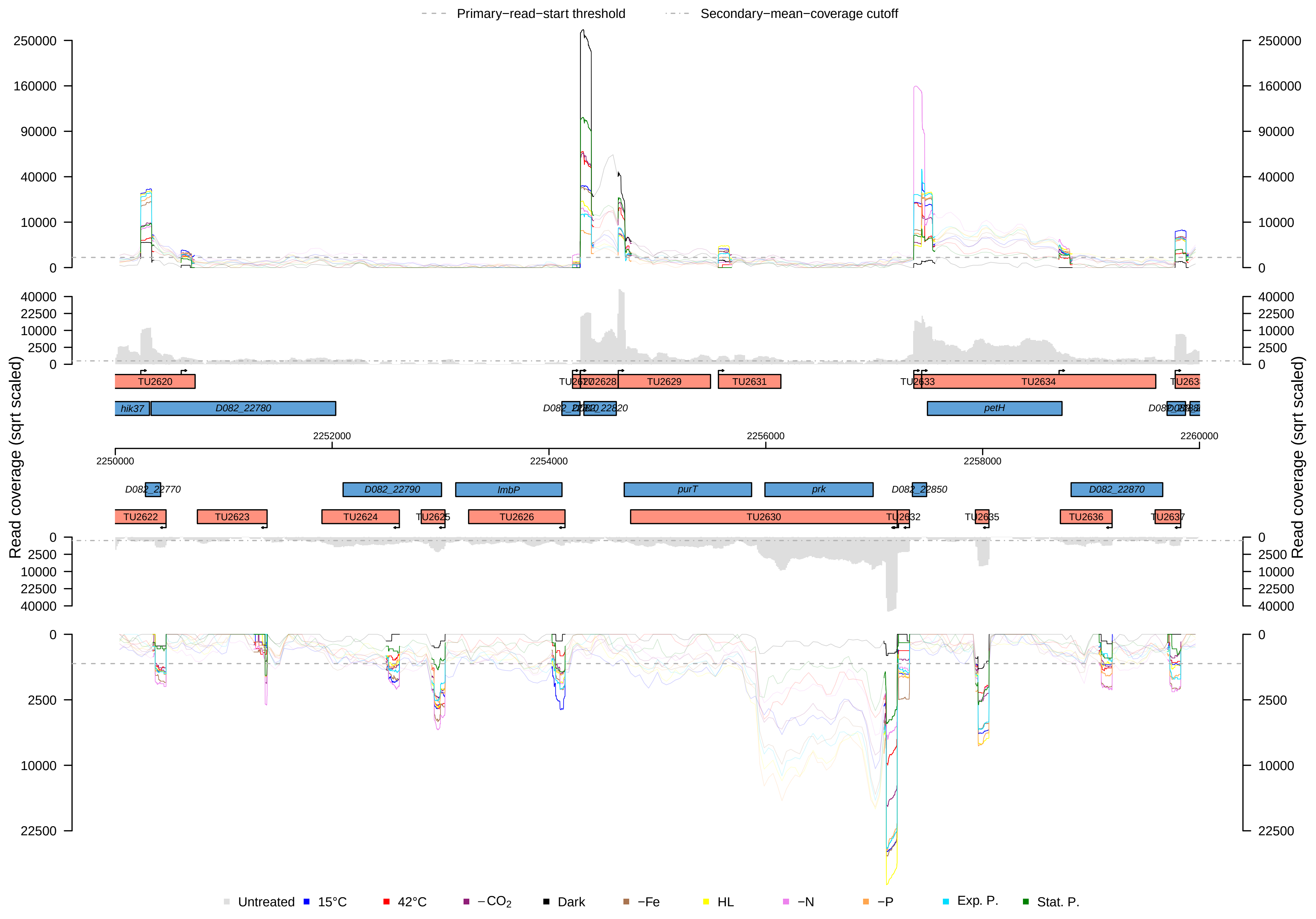

Primary−read−start threshold
Secondary−mean−coverage cutoff
Read coverage (sqrt scaled)
250000
160000
90000
40000
10000
0
40000
22500
10000
2500
0
TU2620
TU2629
TU2631
TU2634
hik37
D082_22780
petH
2252000
2256000
2260000
2250000
2254000
2258000
D082_22770
D082_22790
lmbP
purT
prk
D082_22850
D082_22870
TU2622
TU2623
TU2624
TU2626
TU2630
TU2636
Untreated
15°C
42°C
−CO2
Dark
−Fe
HL
−N
−P
Exp. P.
Stat. P.

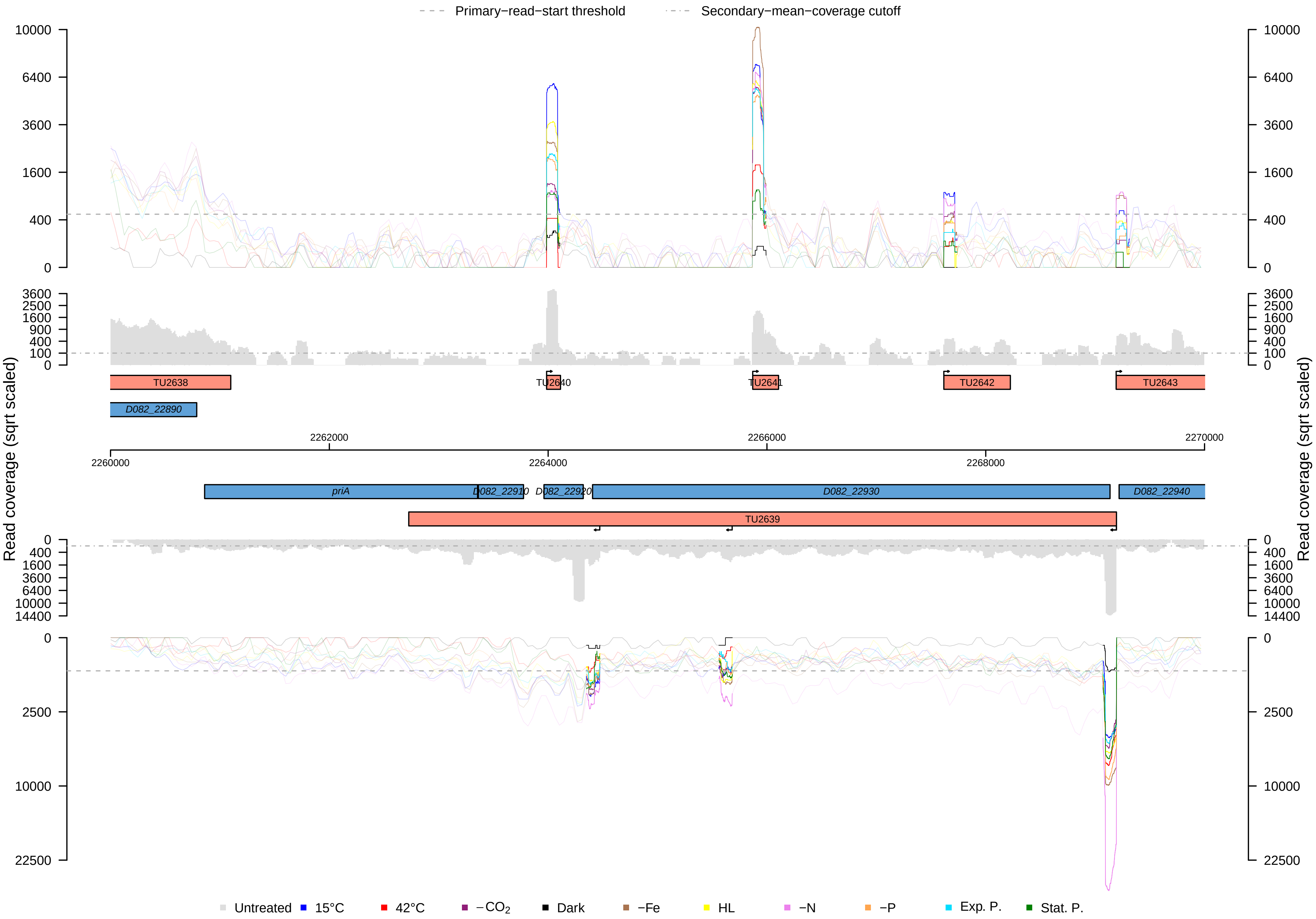

Primary−read−start threshold
Secondary−mean−coverage cutoff
Read coverage (sqrt scaled)
10000
6400
3600
1600
400
0
2500
900
100
14400
22500
TU2638
TU2640
TU2641
TU2642
TU2643
TU2639
D082_22890
priA
D082_22910
D082_22920
D082_22930
D082_22940
2260000
2262000
2264000
2266000
2268000
2270000
Untreated
15°C
42°C
−CO2
Dark
−Fe
HL
−N
−P
Exp. P.
Stat. P.

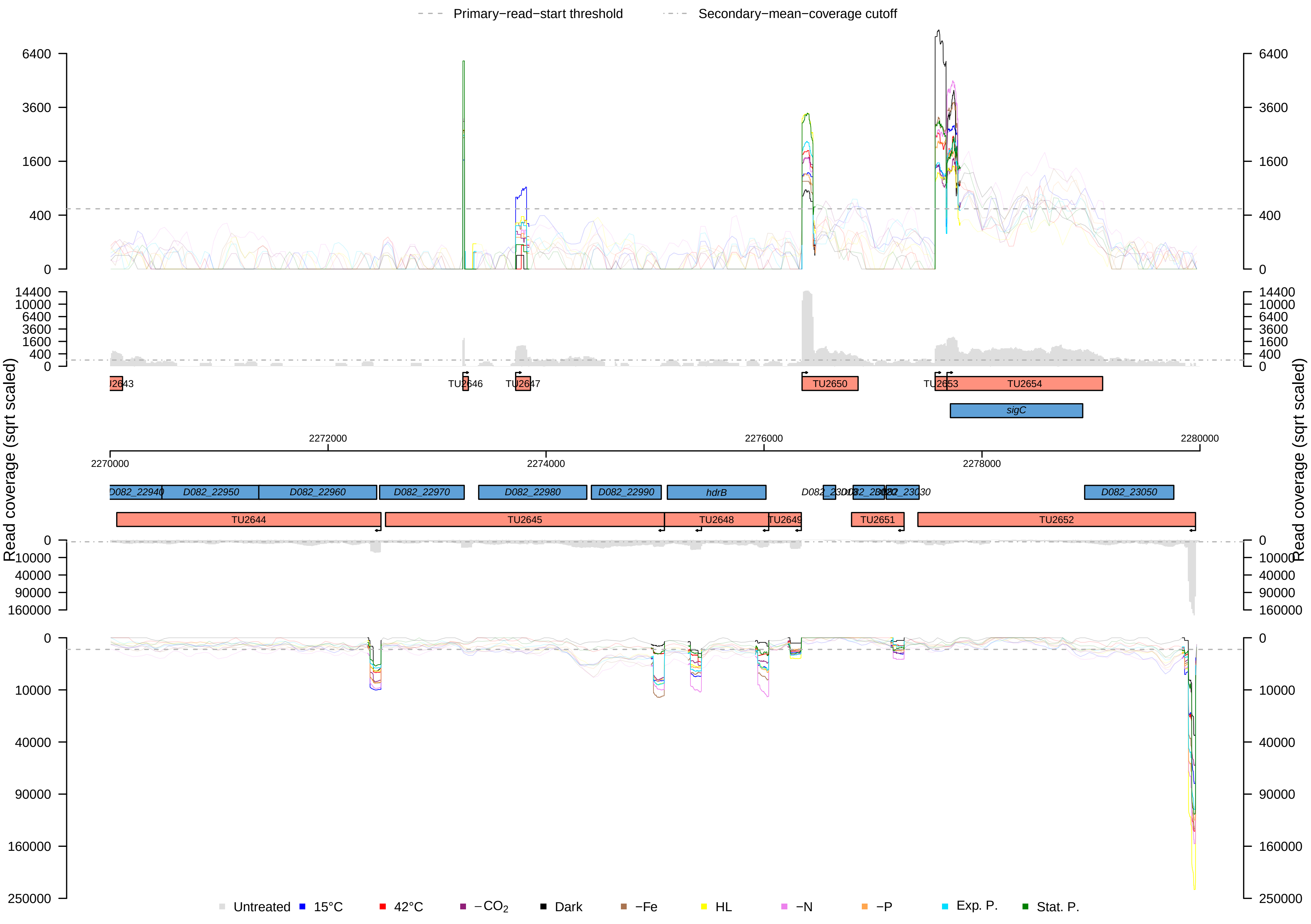

Primary−read−start threshold
Secondary−mean−coverage cutoff
Read coverage (sqrt scaled)
6400
3600
1600
400
0
14400
10000
6400
3600
1600
400
0
TU2646
TU2647
TU2650
TU2653
TU2654
sigC
2270000
2272000
2274000
2276000
2278000
2280000
D082_22950
D082_22960
D082_22970
D082_22980
D082_22990
hdrB
D082_23050
TU2644
TU2645
TU2648
TU2651
TU2652
10000
40000
90000
160000
250000
Untreated
15°C
42°C
−CO2
Dark
−Fe
HL
−N
−P
Exp. P.
Stat. P.

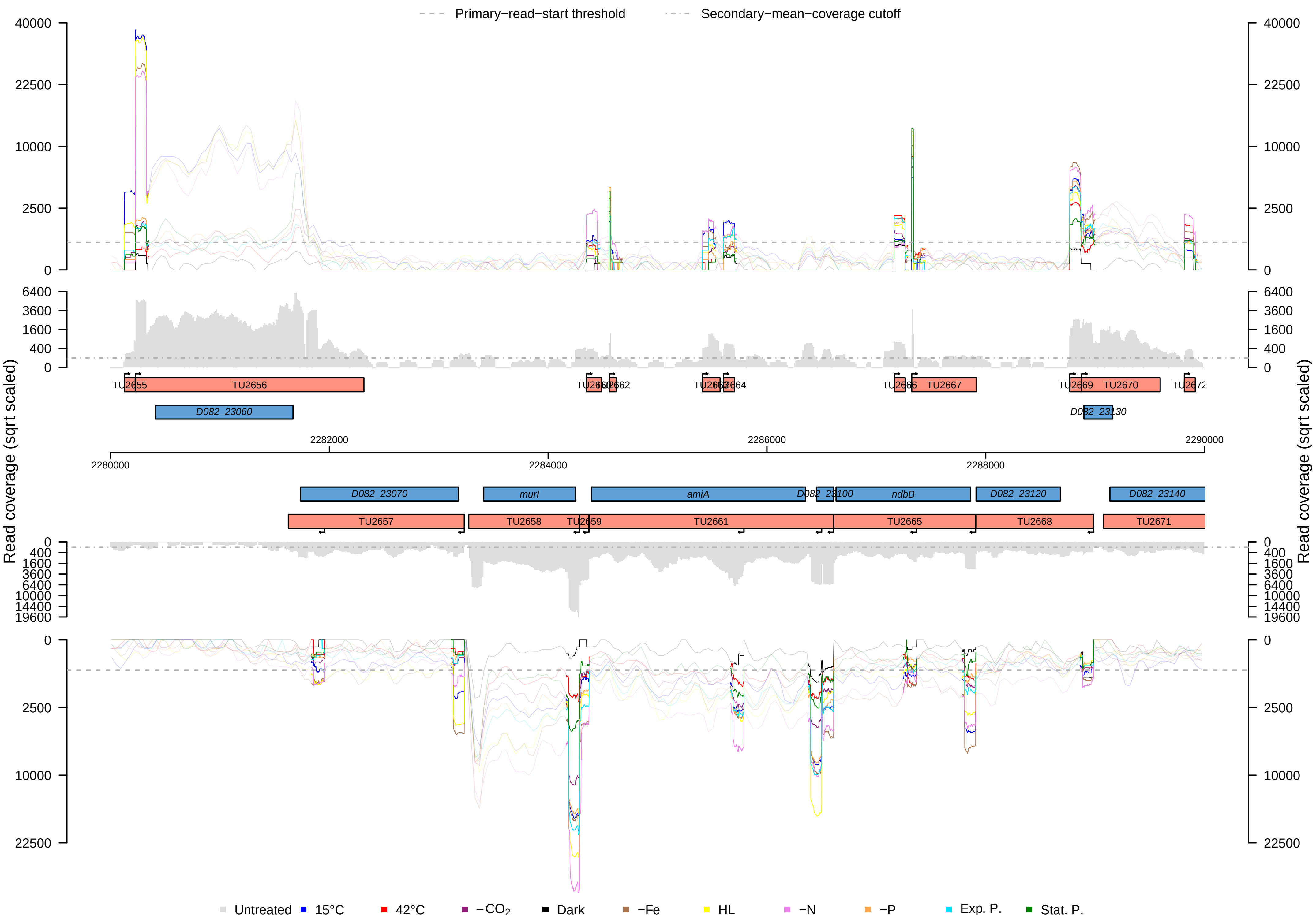

Primary−read−start threshold
Secondary−mean−coverage cutoff
Read coverage (sqrt scaled)
40000
22500
10000
2500
0
6400
3600
1600
400
TU2655
TU2656
TU2662
TU2664
TU2666
TU2667
TU2669
TU2670
D082_23060
D082_23130
2280000
2282000
2284000
2286000
2288000
2290000
D082_23070
murI
amiA
ndbB
D082_23120
D082_23140
TU2657
TU2658
TU2659
TU2661
TU2665
TU2668
TU2671
14400
19600
Untreated
15°C
42°C
−CO2
Dark
−Fe
HL
−N
−P
Exp. P.
Stat. P.

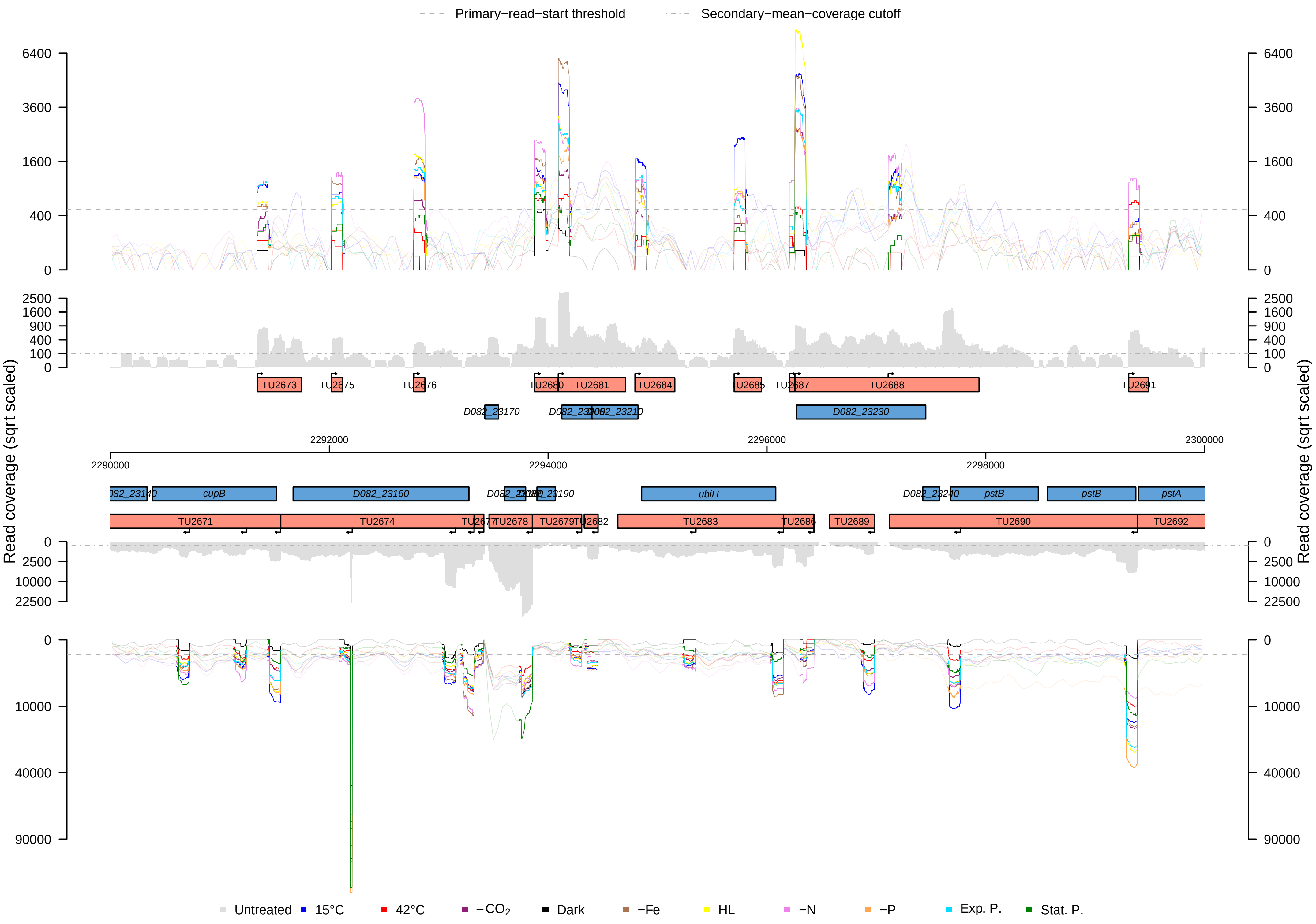

Primary−read−start threshold
Secondary−mean−coverage cutoff
Read coverage (sqrt scaled)
6400
3600
1600
400
0
2500
1600
900
400
100
0
TU2673
TU2675
TU2676
TU2680
TU2681
TU2684
TU2685
TU2687
TU2688
TU2691
D082_23170
D082_23210
D082_23230
2290000
2292000
2294000
2296000
2298000
2300000
cupB
D082_23160
D082_23190
ubiH
pstB
pstB
pstA
TU2671
TU2674
TU2678
TU2679
TU2683
TU2686
TU2689
TU2690
TU2692
2500
10000
22500
40000
90000
Untreated
15°C
42°C
−CO2
Dark
−Fe
HL
−N
−P
Exp. P.
Stat. P.

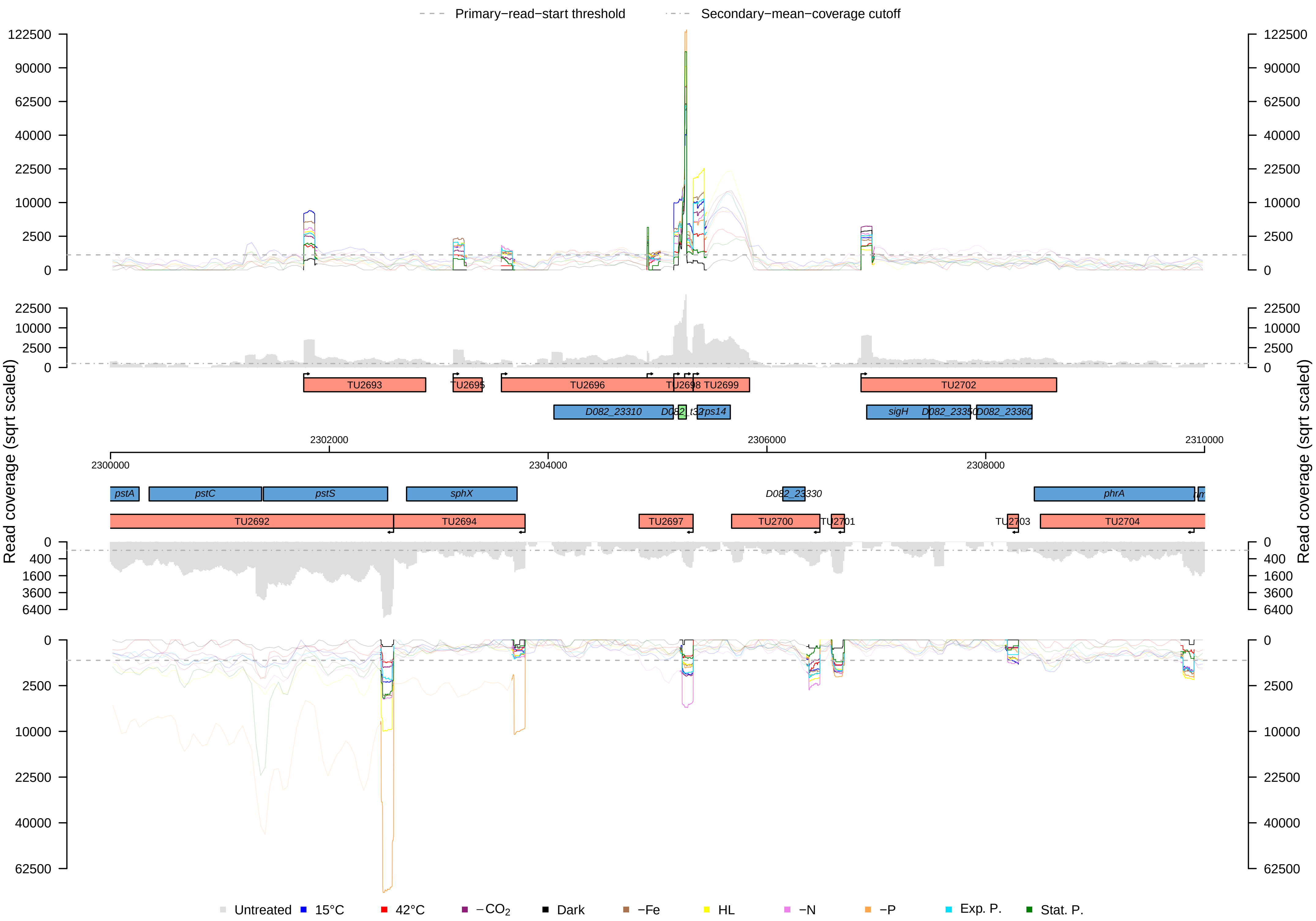

Primary−read−start threshold
Secondary−mean−coverage cutoff
Read coverage (sqrt scaled)
122500
90000
62500
40000
22500
10000
2500
0
400
1600
3600
6400
TU2693
TU2695
TU2696
TU2698
TU2699
TU2702
D082_23310
rps14
sigH
D082_23350
D082_23360
2300000
2302000
2304000
2306000
2308000
2310000
pstA
pstC
pstS
sphX
D082_23330
phrA
TU2692
TU2694
TU2697
TU2700
TU2701
TU2703
TU2704
Untreated
15°C
42°C
−CO2
Dark
−Fe
HL
−N
−P
Exp. P.
Stat. P.

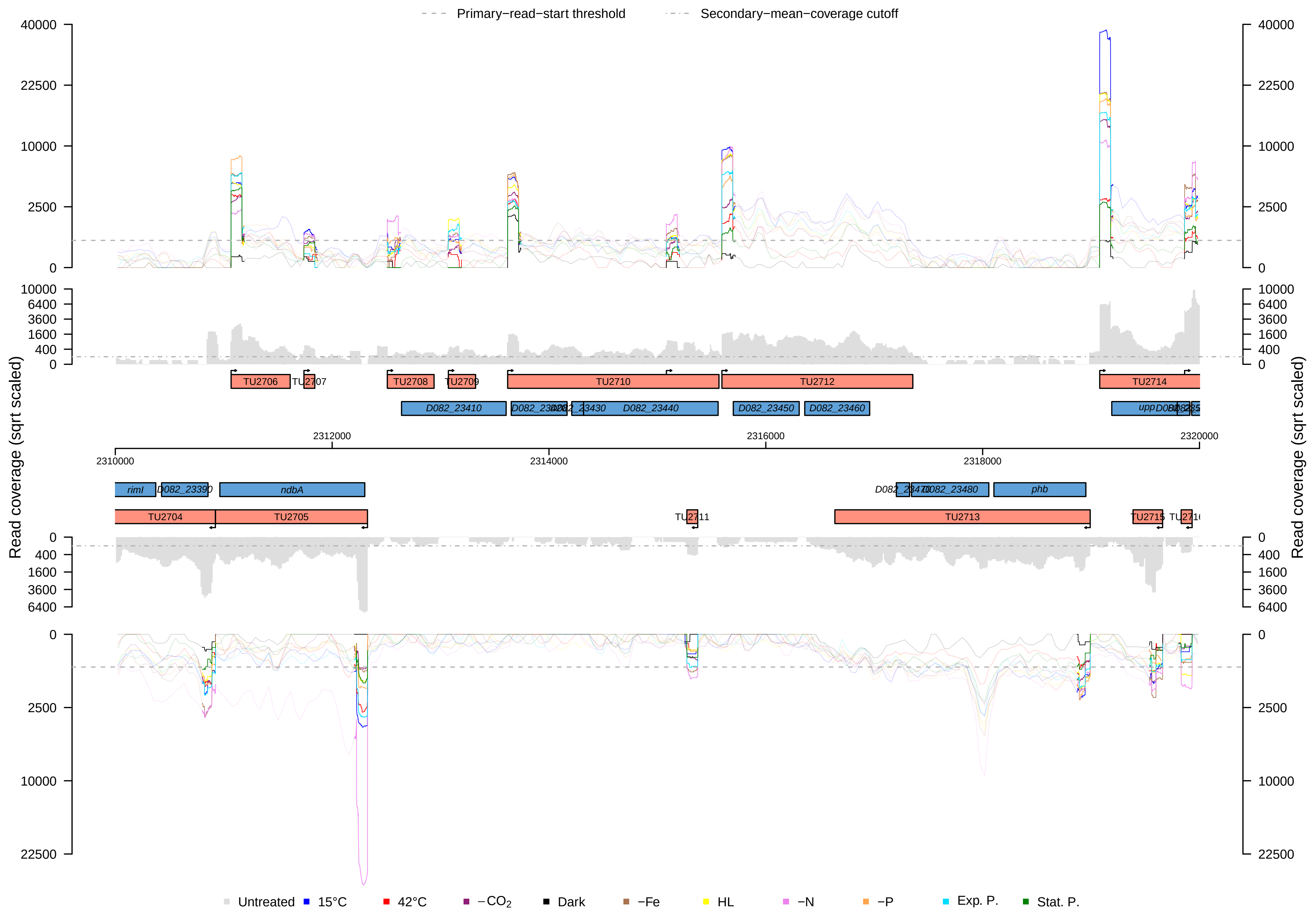

Primary−read−start threshold
Secondary−mean−coverage cutoff
Read coverage (sqrt scaled)
40000
22500
10000
2500
0
10000
6400
3600
1600
400
TU2706
TU2707
TU2708
TU2709
TU2710
TU2712
TU2714
D082_23410
D082_23430
D082_23440
D082_23450
D082_23460
upp
2310000
2312000
2314000
2316000
2318000
2320000
rimI
D082_23390
ndbA
D082_23480
phb
TU2704
TU2705
TU2711
TU2713
TU2715
Untreated
15°C
42°C
−CO2
Dark
−Fe
HL
−N
−P
Exp. P.
Stat. P.

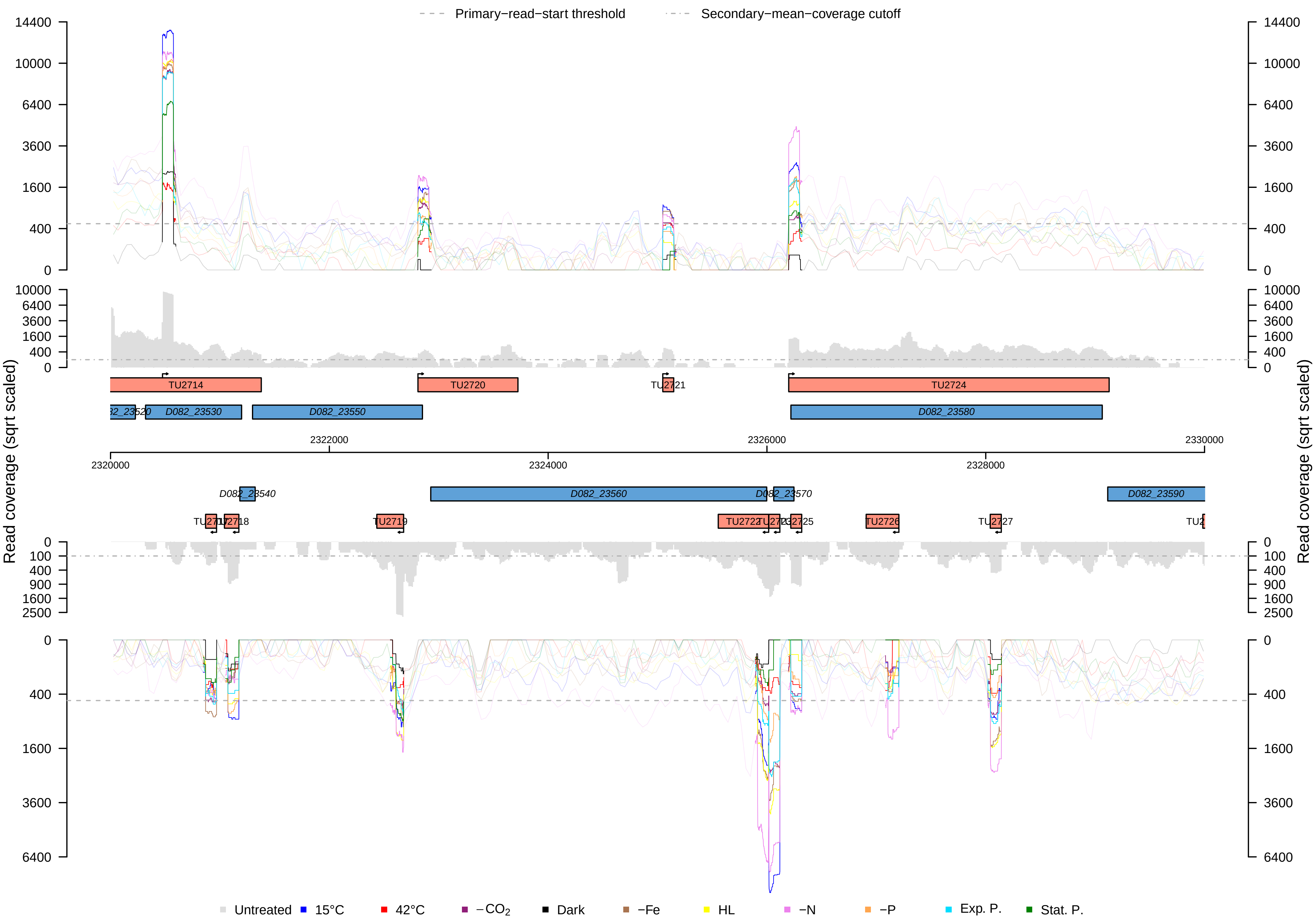

Primary−read−start threshold
Secondary−mean−coverage cutoff
Read coverage (sqrt scaled)
Read coverage (sqrt scaled)
14400
10000
6400
3600
1600
400
0
TU2714
TU2720
TU2721
TU2724
D082_23530
D082_23550
D082_23580
2320000
2322000
2324000
2326000
2328000
2330000
D082_23540
D082_23560
D082_23570
D082_23590
TU2718
TU2719
TU2726
TU2727
Untreated
15°C
42°C
−CO2
Dark
−Fe
HL
−N
−P
Exp. P.
Stat. P.

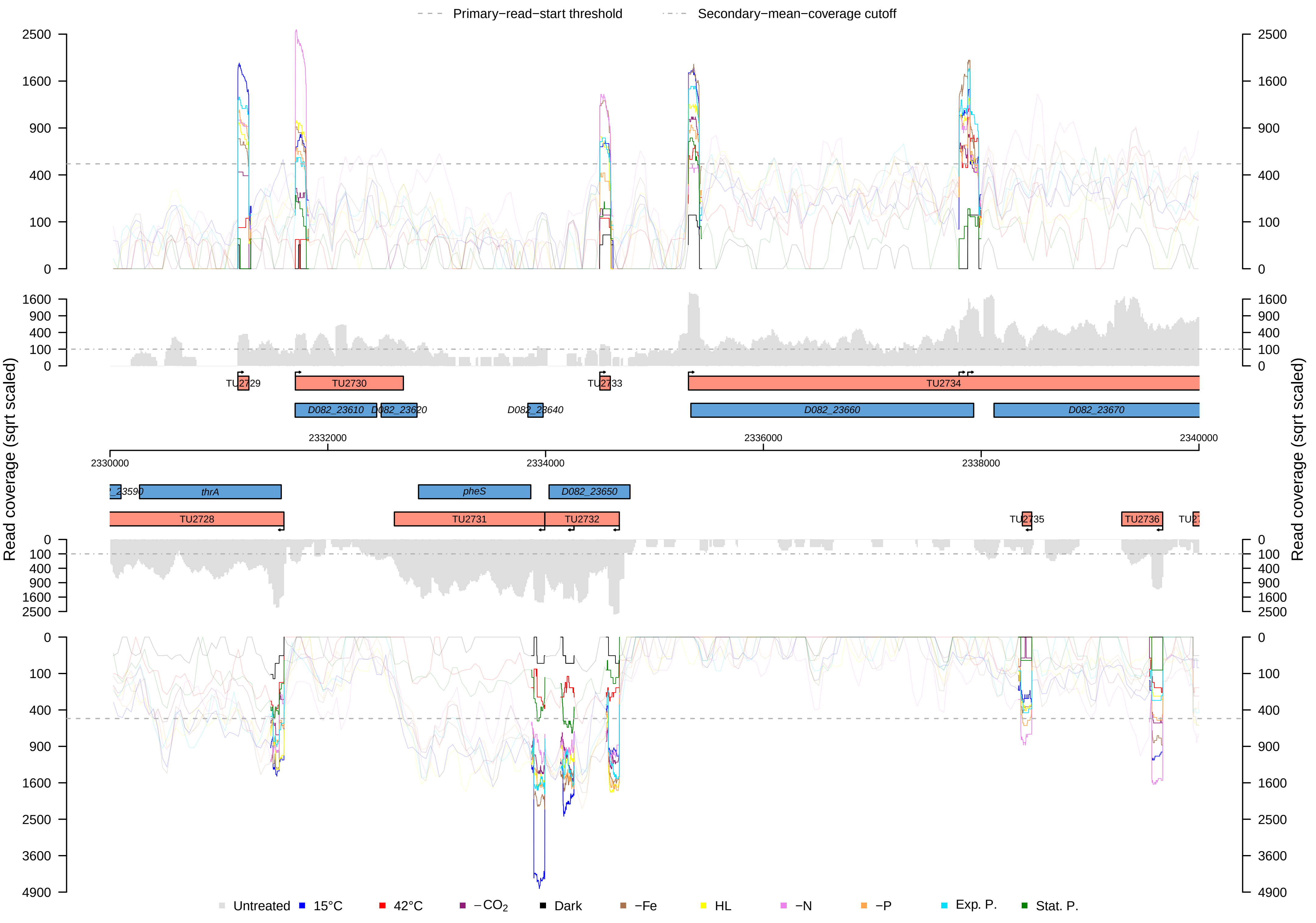

Primary−read−start threshold
Secondary−mean−coverage cutoff
Read coverage (sqrt scaled)
TU2729
TU2730
TU2733
TU2734
D082_23610
D082_23620
D082_23640
D082_23660
D082_23670
2330000
2332000
2334000
2336000
2338000
2340000
thrA
pheS
D082_23650
TU2728
TU2731
TU2732
TU2735
TU2736
Untreated
15°C
42°C
−CO2
Dark
−Fe
HL
−N
−P
Exp. P.
Stat. P.

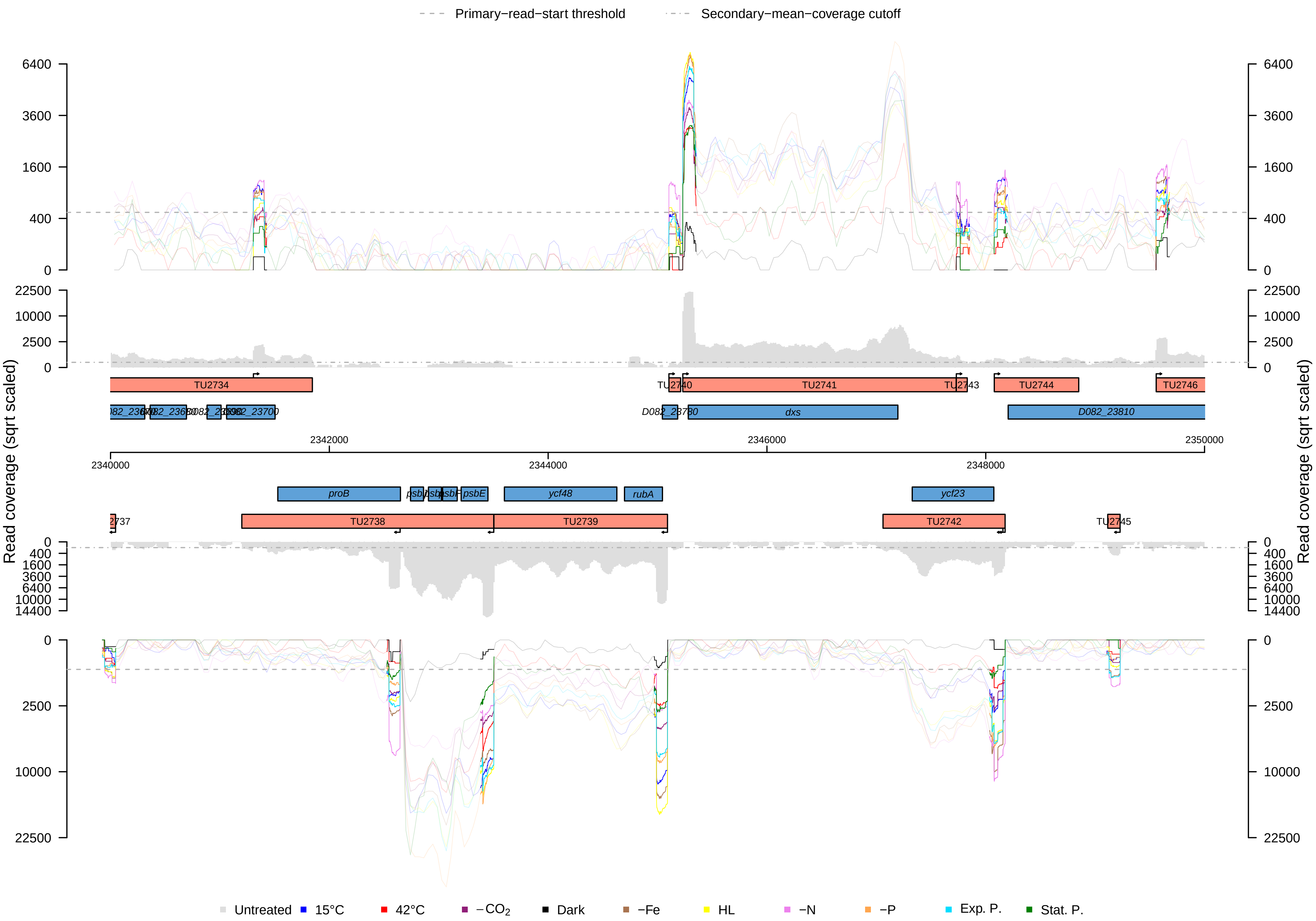

Primary−read−start threshold
Secondary−mean−coverage cutoff
Read coverage (sqrt scaled)
TU2734
TU2740
TU2741
TU2743
TU2744
TU2746
dxs
D082_23810
2340000
2342000
2344000
2346000
2348000
2350000
proB
psbE
ycf48
rubA
ycf23
TU2738
TU2739
TU2742
TU2745
Untreated
15°C
42°C
−CO2
Dark
−Fe
HL
−N
−P
Exp. P.
Stat. P.

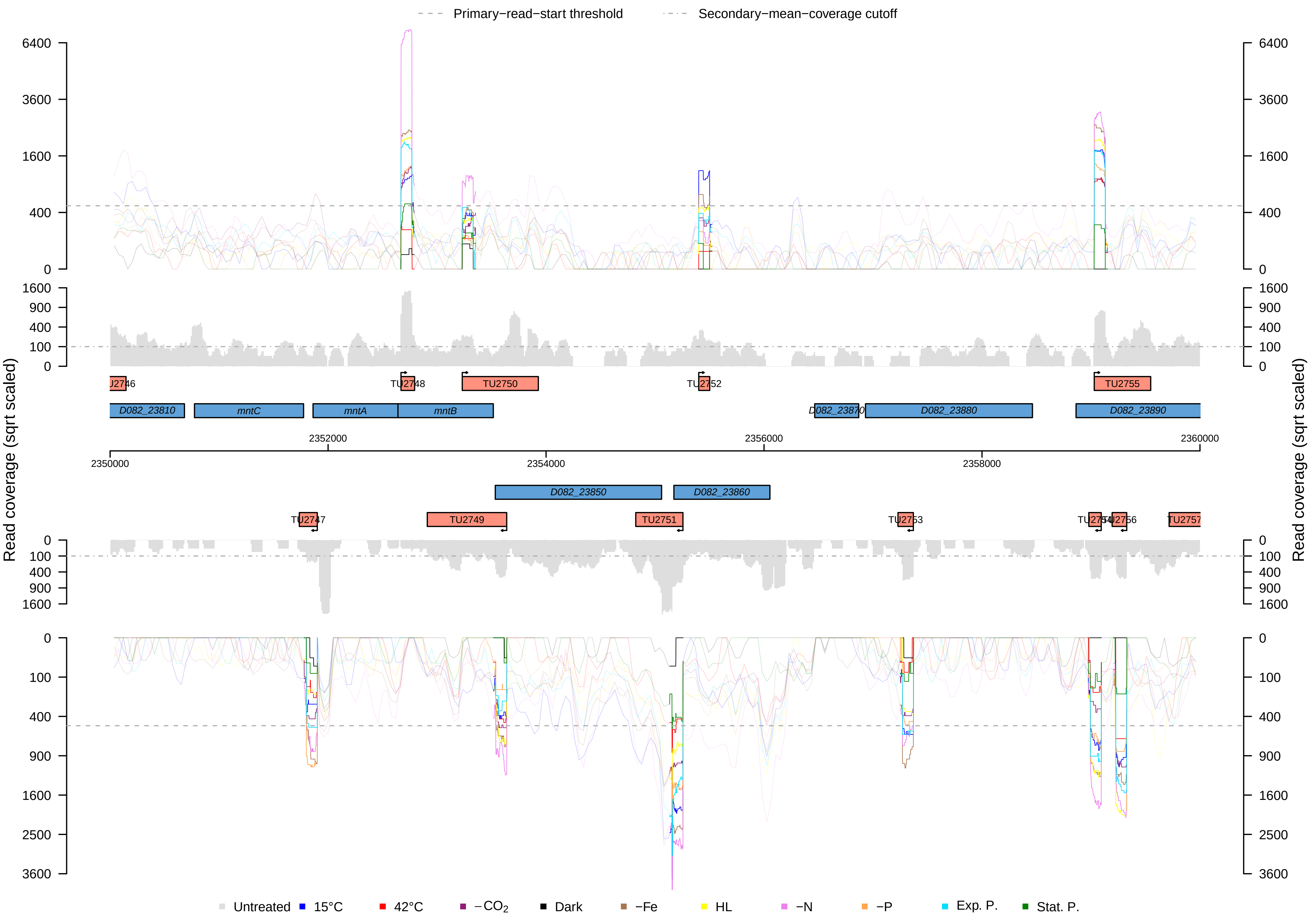
Primary−read−start threshold
Secondary−mean−coverage cutoff
Read coverage (sqrt scaled)
6400
3600
1600
400
0
900
100
2500
TU2748
TU2750
TU2752
TU2755
D082_23810
mntC
mntA
mntB
D082_23870
D082_23880
D082_23890
2350000
2352000
2354000
2356000
2358000
2360000
D082_23850
D082_23860
TU2747
TU2749
TU2751
TU2753
TU2756
TU2757
Untreated
15°C
42°C
−CO2
Dark
−Fe
HL
−N
−P
Exp. P.
Stat. P.

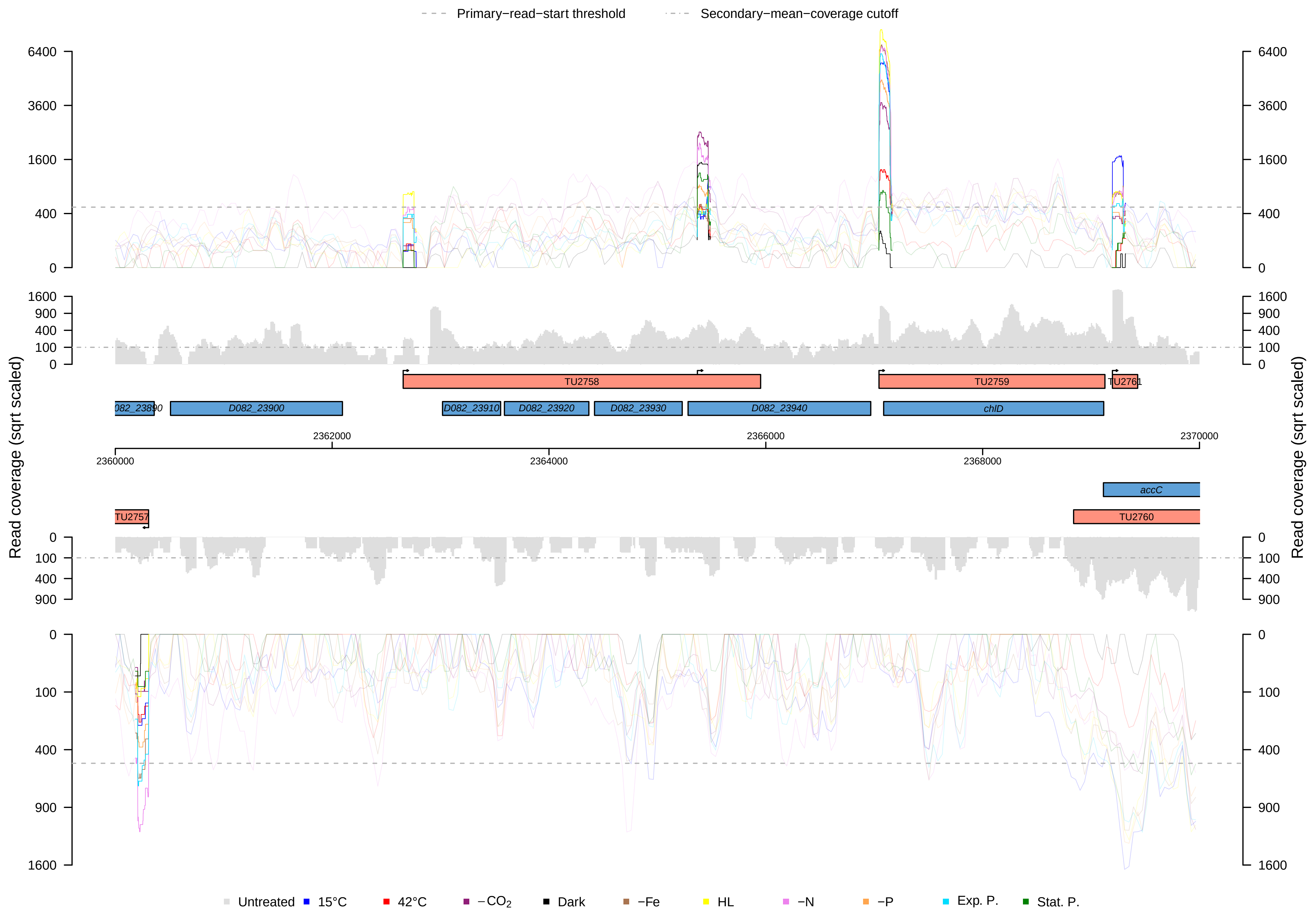

Primary−read−start threshold
Secondary−mean−coverage cutoff
Read coverage (sqrt scaled)
6400
3600
1600
400
0
1600
900
400
100
0
TU2758
TU2759
TU2761
082_23890
D082_23900
D082_23910
D082_23920
D082_23930
D082_23940
chlD
2360000
2362000
2364000
2366000
2368000
2370000
accC
TU2757
TU2760
Untreated
15°C
42°C
−CO2
Dark
−Fe
HL
−N
−P
Exp. P.
Stat. P.

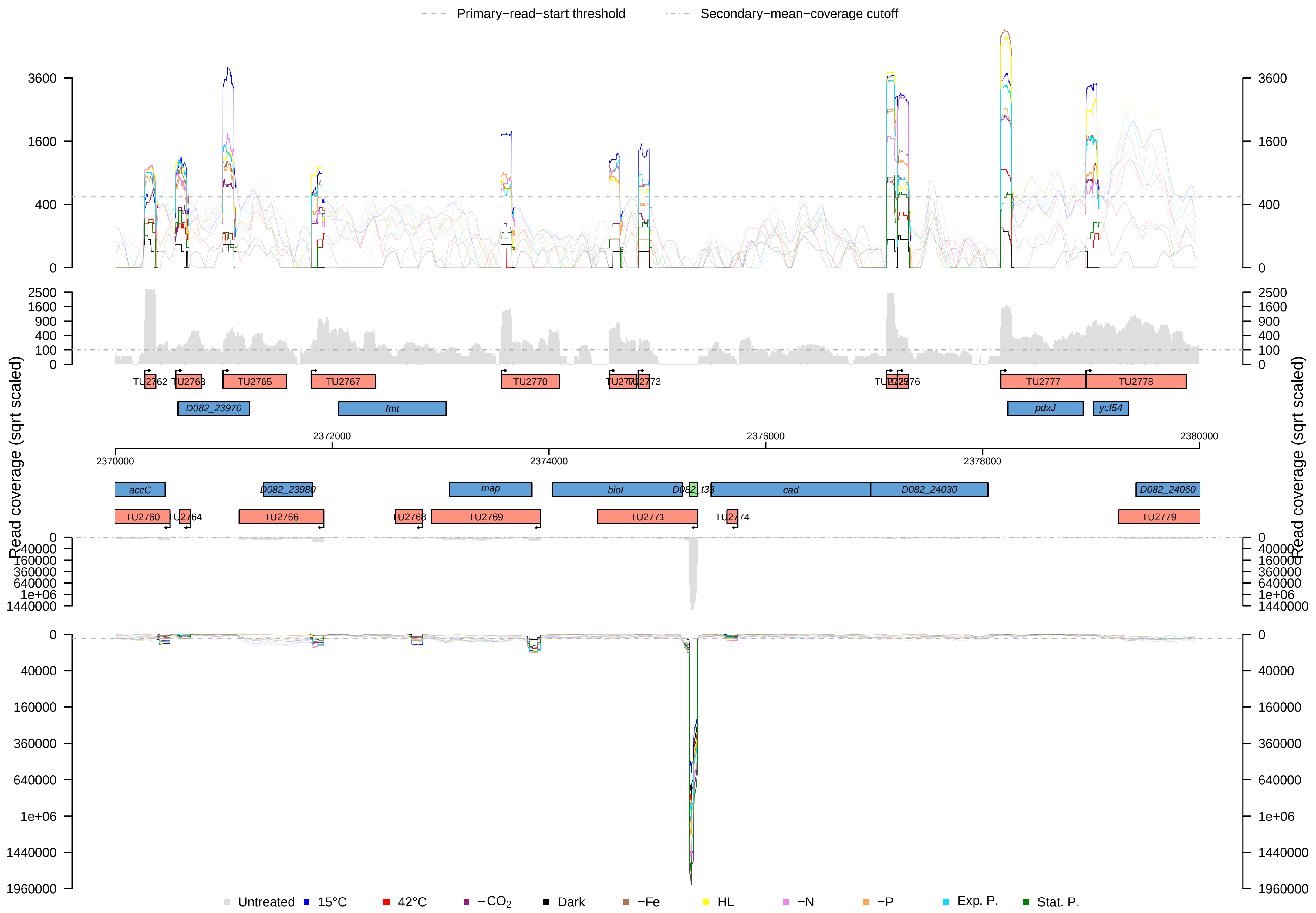

Primary−read−start threshold
Secondary−mean−coverage cutoff
Read coverage (sqrt scaled)
TU2762
TU2763
TU2765
TU2767
TU2770
TU2772
TU2773
TU2775
TU2776
TU2777
TU2778
D082_23970
fmt
pdxJ
ycf54
2370000
2372000
2374000
2376000
2378000
2380000
accC
D082_23980
map
bioF
D082
t33
cad
D082_24030
D082_24060
TU2760
TU2764
TU2766
TU2768
TU2769
TU2771
TU2774
TU2779
Untreated
15°C
42°C
−CO2
Dark
−Fe
HL
−N
−P
Exp. P.
Stat. P.

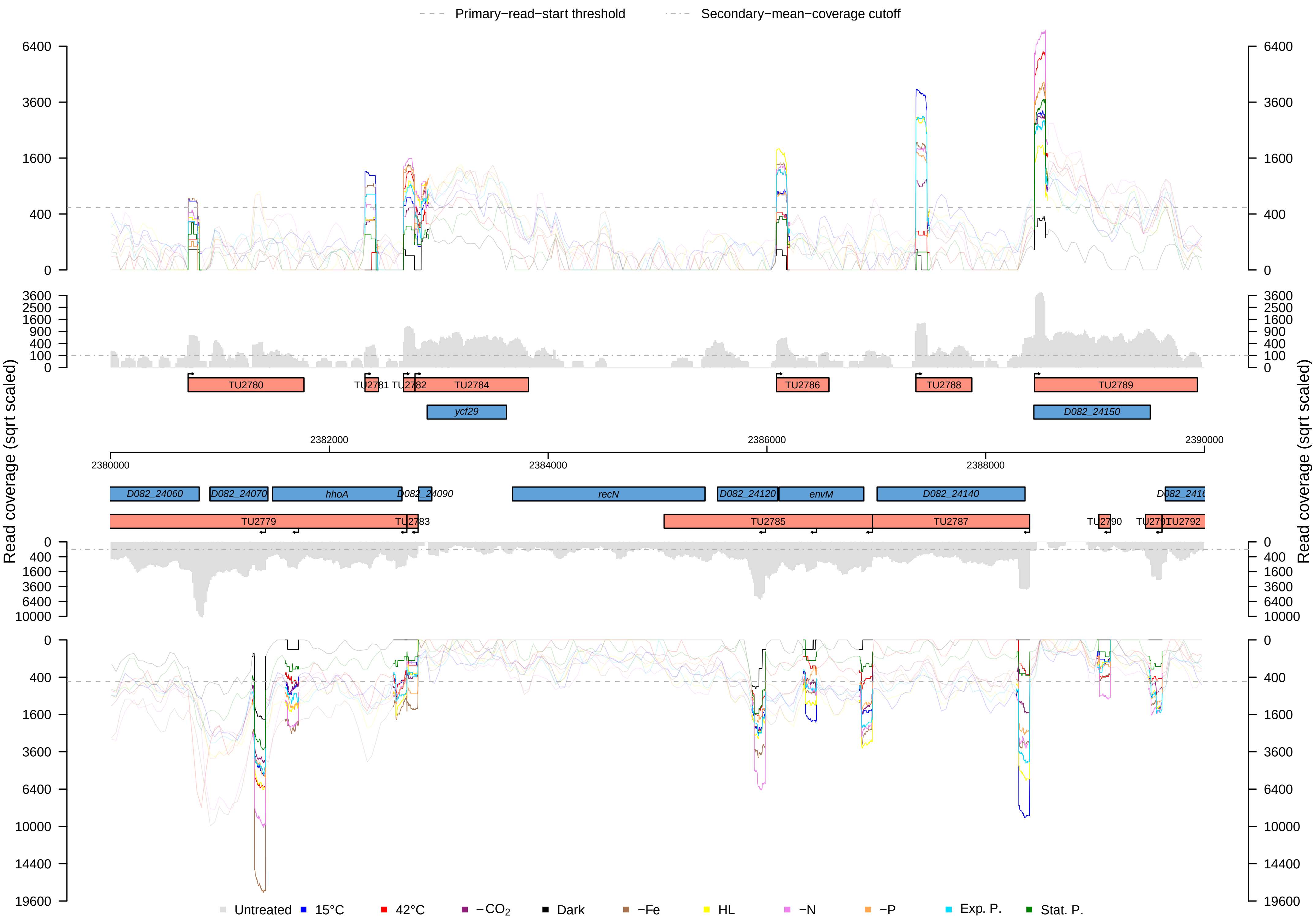

Primary−read−start threshold
Secondary−mean−coverage cutoff
Read coverage (sqrt scaled)
TU2780
TU2781
TU2782
TU2784
TU2786
TU2788
TU2789
ycf29
D082_24150
2380000
2382000
2384000
2386000
2388000
2390000
D082_24060
D082_24070
hhoA
D082_24090
recN
D082_24120
envM
D082_24140
TU2779
TU2783
TU2785
TU2787
TU2790
TU2791
TU2792
Untreated
15°C
42°C
−CO2
Dark
−Fe
HL
−N
−P
Exp. P.
Stat. P.

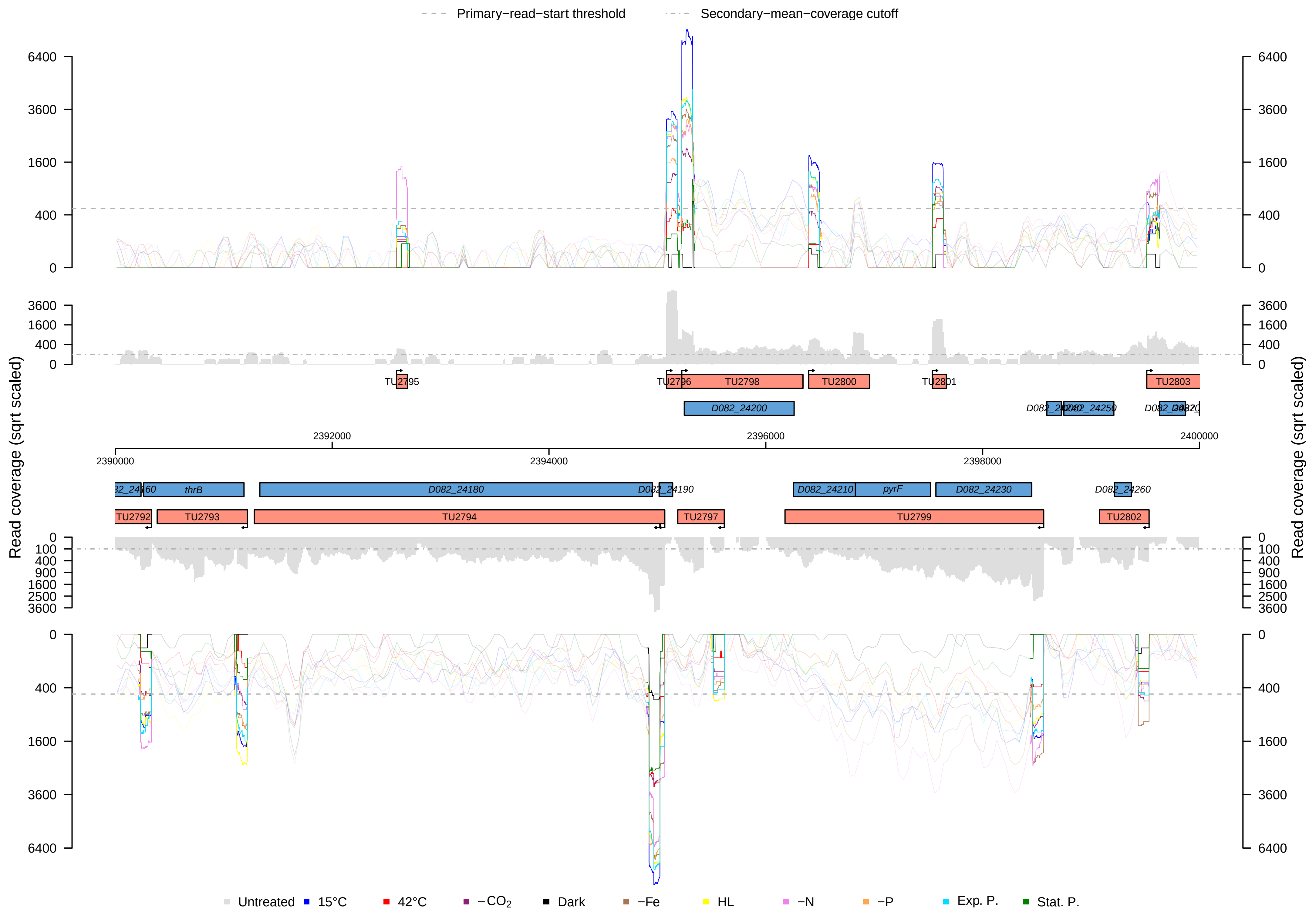

Primary−read−start threshold
Secondary−mean−coverage cutoff
Read coverage (sqrt scaled)
TU2795
TU2796
TU2798
TU2800
TU2801
TU2803
D082_24200
D082_24250
thrB
D082_24180
D082_24190
D082_24210
pyrF
D082_24230
D082_24260
TU2792
TU2793
TU2794
TU2797
TU2799
TU2802
2390000
2392000
2394000
2396000
2398000
2400000
Untreated
15°C
42°C
−CO2
Dark
−Fe
HL
−N
−P
Exp. P.
Stat. P.

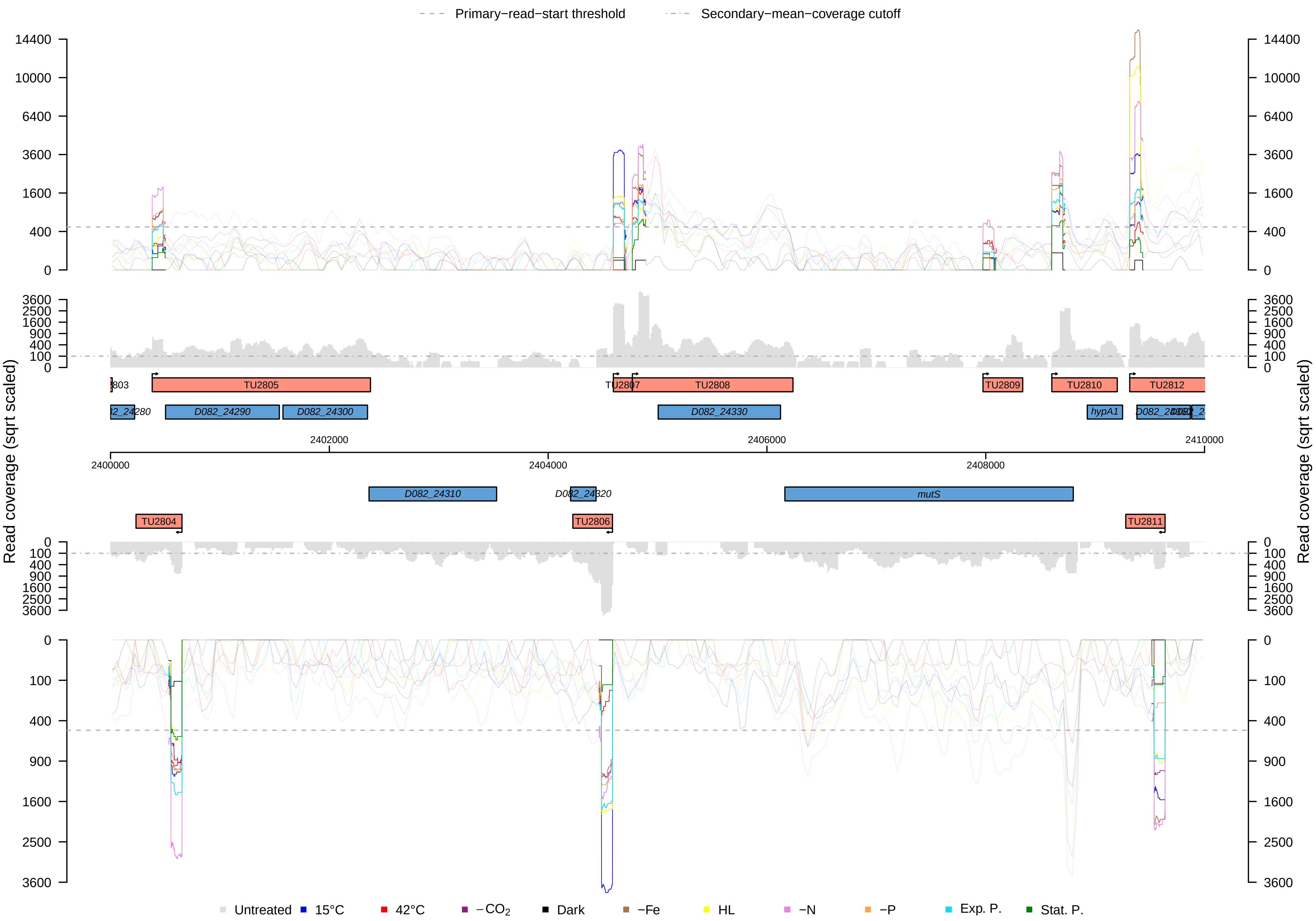

Primary−read−start threshold
Secondary−mean−coverage cutoff
Read coverage (sqrt scaled)
TU2805
TU2807
TU2808
TU2809
TU2810
TU2812
D082_24290
D082_24300
D082_24330
hypA1
2400000
2402000
2404000
2406000
2408000
2410000
D082_24310
mutS
TU2804
TU2806
TU2811
Untreated
15°C
42°C
−CO2
Dark
−Fe
HL
−N
−P
Exp. P.
Stat. P.

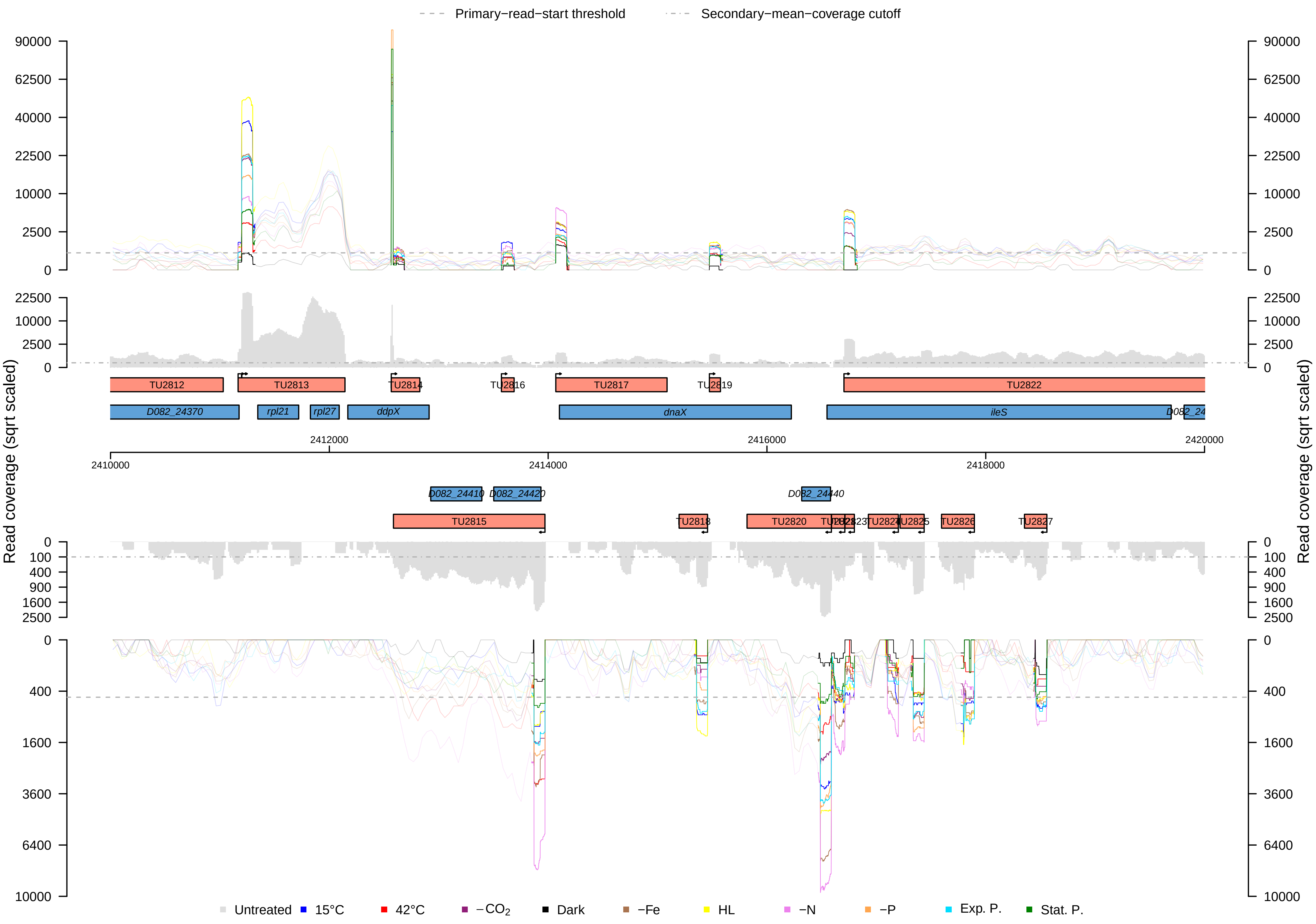

Primary−read−start threshold
Secondary−mean−coverage cutoff
Read coverage (sqrt scaled)
TU2812
TU2813
TU2814
TU2816
TU2817
TU2819
TU2822
D082_24370
rpl21
rpl27
ddpX
dnaX
ileS
2410000
2412000
2414000
2416000
2418000
2420000
D082_24410
D082_24420
D082_24440
TU2815
TU2818
TU2820
TU2824
TU2825
TU2826
TU2827
Untreated
15°C
42°C
−CO2
Dark
−Fe
HL
−N
−P
Exp. P.
Stat. P.

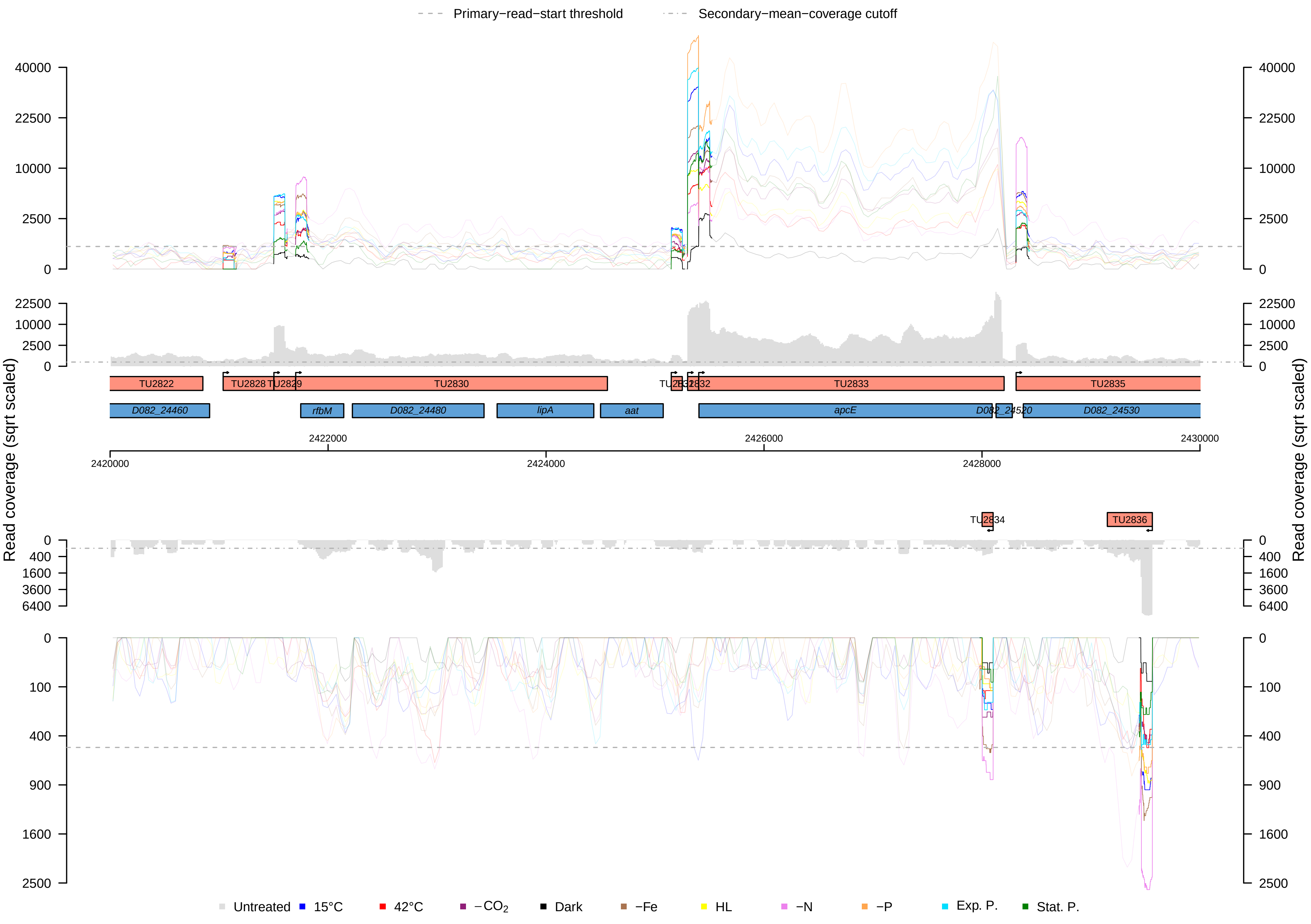

Primary−read−start threshold
Secondary−mean−coverage cutoff
40000
22500
10000
2500
0
TU2822
TU2828
TU2829
TU2830
TU2832
TU2833
TU2835
D082_24460
rfbM
D082_24480
lipA
aat
apcE
D082_24520
D082_24530
2420000
2422000
2424000
2426000
2428000
2430000
Read coverage (sqrt scaled)
TU2834
TU2836
400
1600
3600
6400
100
900
Untreated
15°C
42°C
−CO_2
Dark
−Fe
HL
−N
−P
Exp. P.
Stat. P.

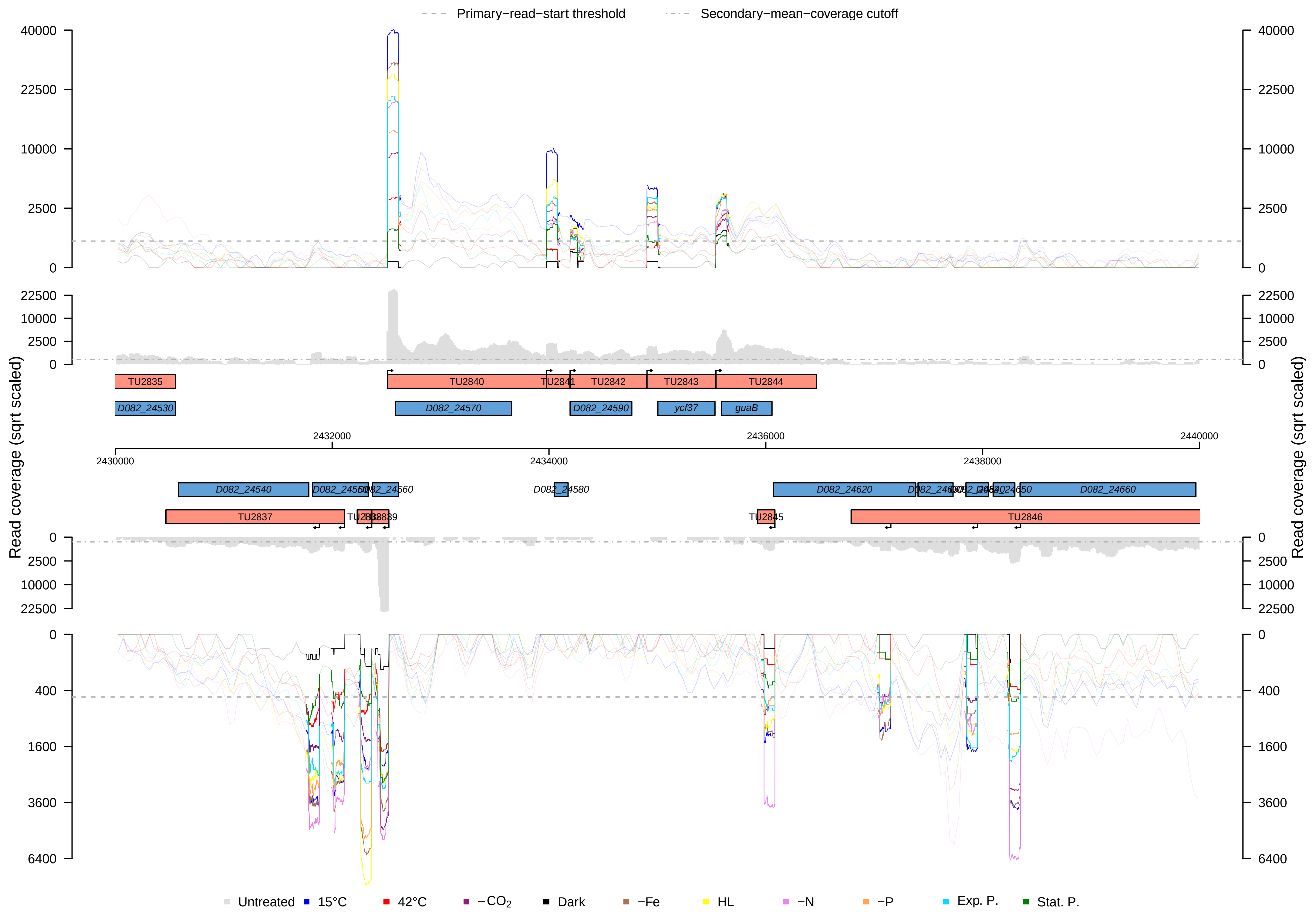

Primary−read−start threshold
Secondary−mean−coverage cutoff
40000
22500
10000
2500
0
Read coverage (sqrt scaled)
TU2835
TU2840
TU2841
TU2842
TU2843
TU2844
D082_24530
D082_24570
D082_24590
ycf37
guaB
2430000
2432000
2434000
2436000
2438000
2440000
D082_24540
D082_24620
D082_24660
D082_24580
TU2837
TU2845
TU2846
400
1600
3600
6400
Untreated
15°C
42°C
−CO2
Dark
−Fe
HL
−N
−P
Exp. P.
Stat. P.

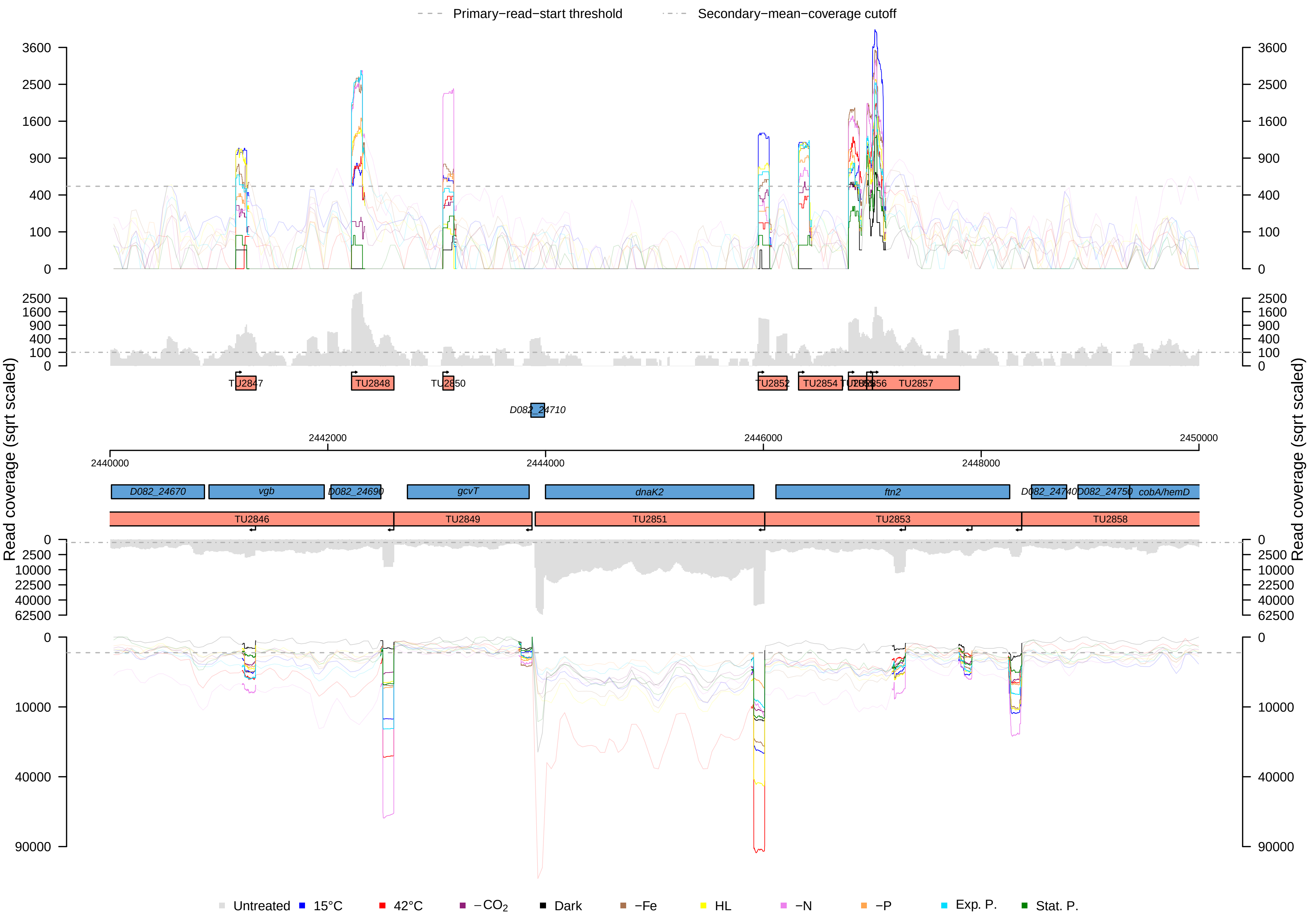

Primary−read−start threshold
Secondary−mean−coverage cutoff
Read coverage (sqrt scaled)
TU2847
TU2848
TU2850
TU2852
TU2854
TU2857
D082_24710
2440000
2442000
2444000
2446000
2448000
2450000
D082_24670
vgb
D082_24690
gcvT
dnaK2
ftn2
D082_24750
cobA/hemD
TU2846
TU2849
TU2851
TU2853
TU2858
Untreated
15°C
42°C
−CO2
Dark
−Fe
HL
−N
−P
Exp. P.
Stat. P.

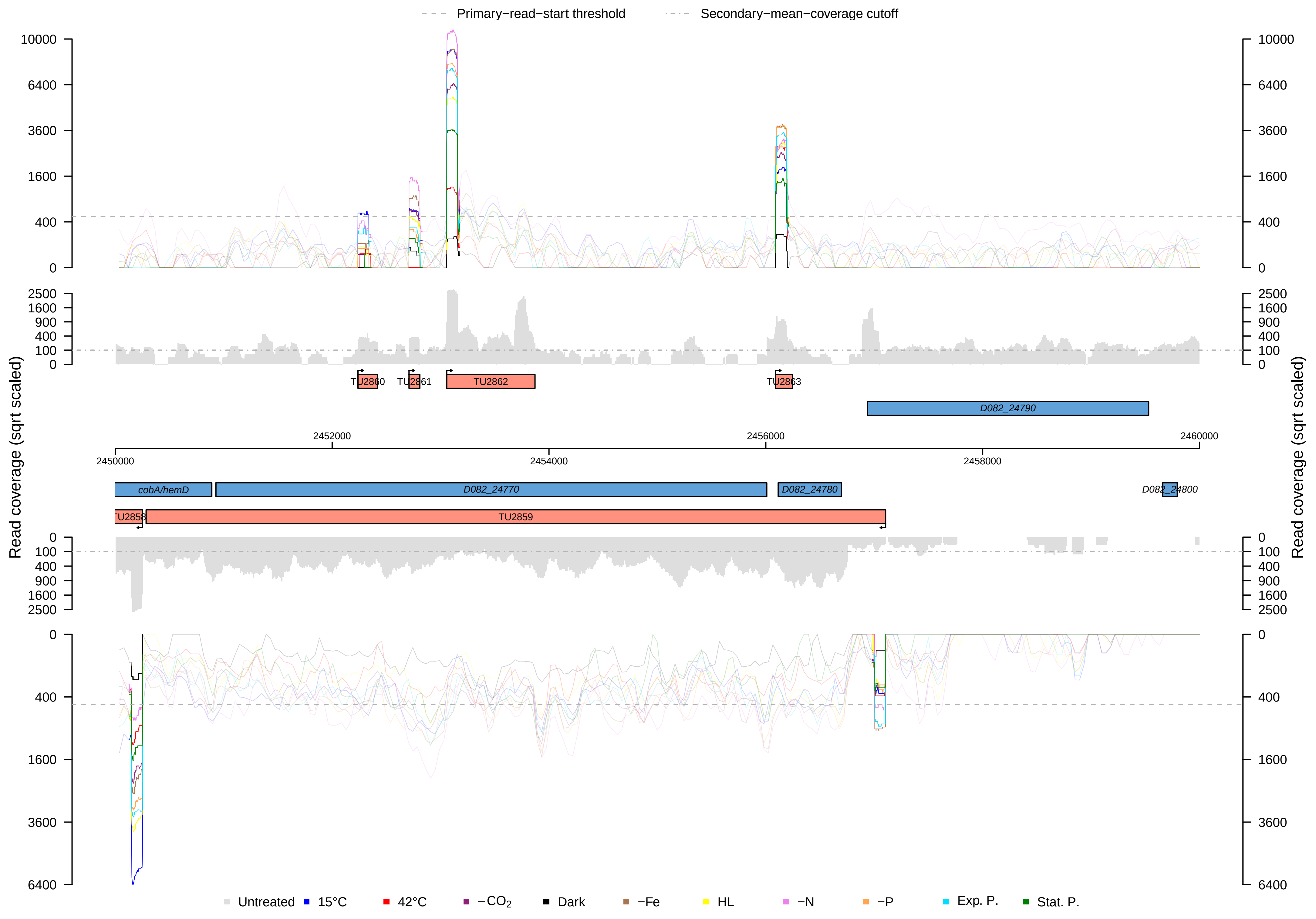

Primary−read−start threshold
Secondary−mean−coverage cutoff
Read coverage (sqrt scaled)
10000
6400
3600
1600
400
0
2500
1600
900
400
100
TU2860
TU2861
TU2862
TU2863
D082_24790
2450000
2452000
2454000
2456000
2458000
2460000
cobA/hemD
D082_24770
D082_24780
D082_24800
TU2858
TU2859
Untreated
15°C
42°C
−CO2
Dark
−Fe
HL
−N
−P
Exp. P.
Stat. P.

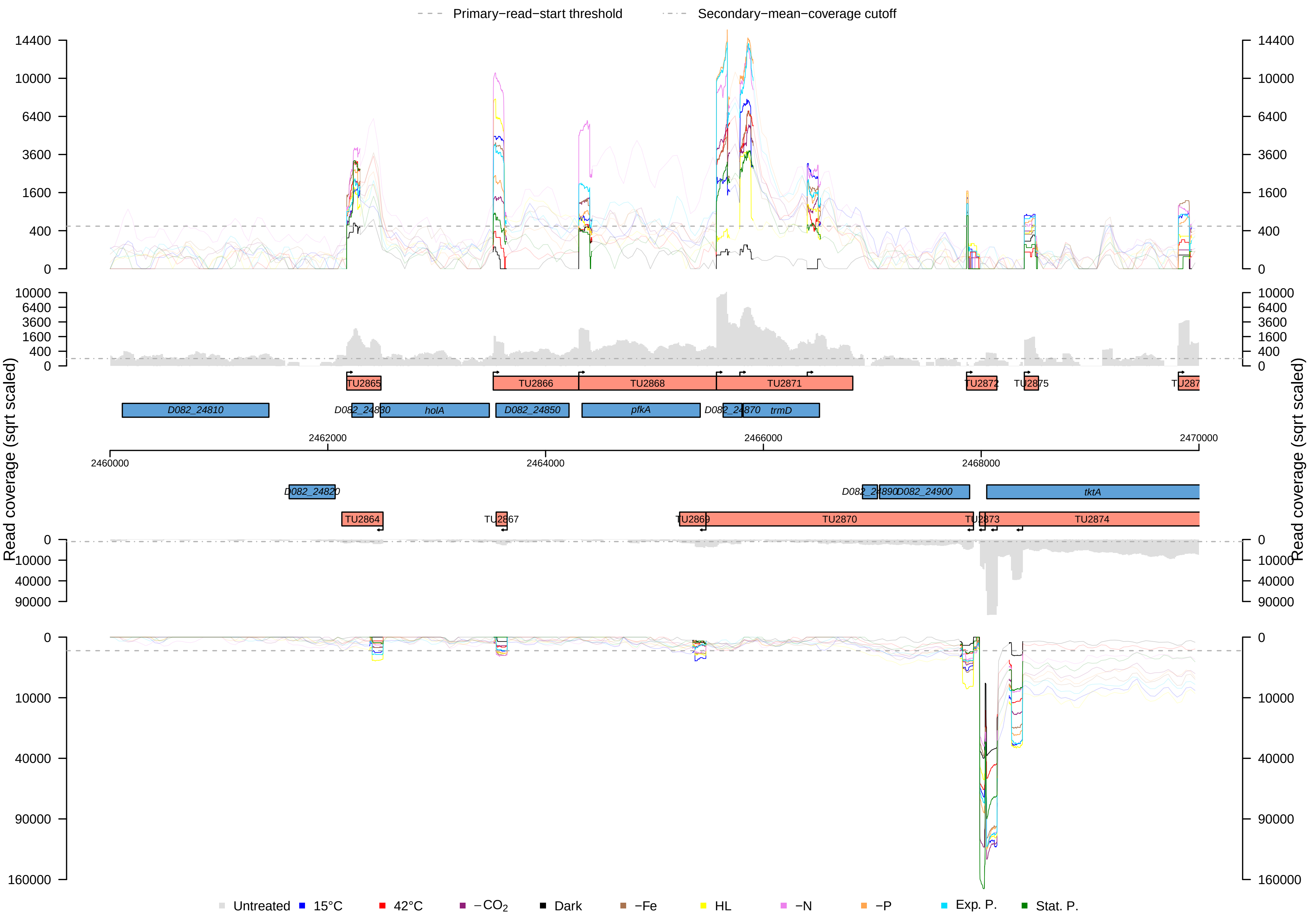

Primary−read−start threshold
Secondary−mean−coverage cutoff
Read coverage (sqrt scaled)
14400
10000
6400
3600
1600
400
0
TU2865
TU2866
TU2868
TU2871
TU2872
TU2875
TU287
D082_24810
D082_24830
holA
D082_24850
pfkA
D082_24870
trmD
2460000
2462000
2464000
2466000
2468000
2470000
D082_24820
D082_24890
D082_24900
tktA
TU2864
TU2867
TU2869
TU2870
TU2873
TU2874
40000
90000
160000
Untreated
15°C
42°C
−CO2
Dark
−Fe
HL
−N
−P
Exp. P.
Stat. P.

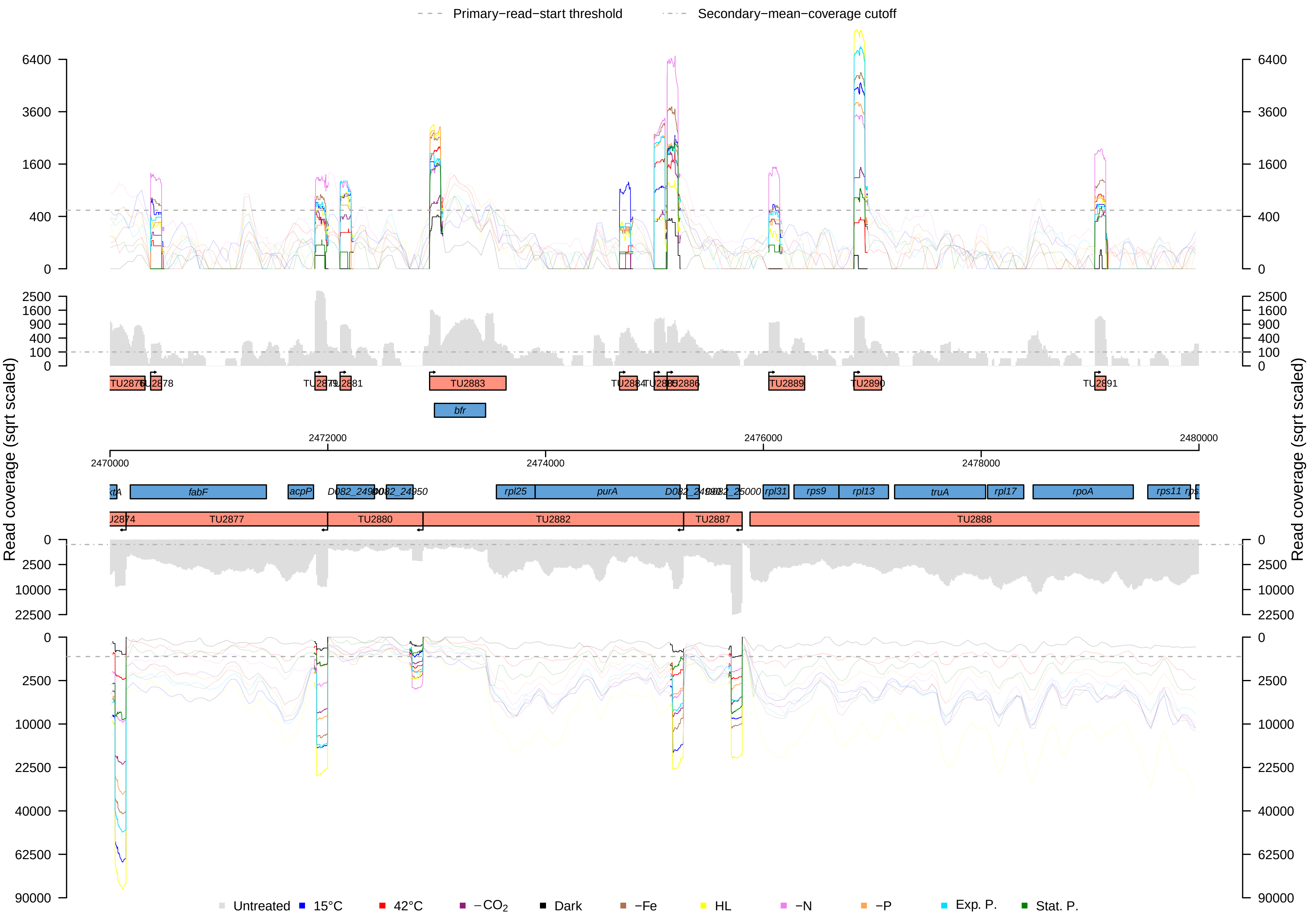

Primary−read−start threshold
Secondary−mean−coverage cutoff
Read coverage (sqrt scaled)
6400
3600
1600
400
0
2500
1600
900
400
100
0
TU2876
TU2878
TU2879
TU2881
TU2883
bfr
TU2884
TU2886
TU2889
TU2890
TU2891
2470000
2472000
2474000
2476000
2478000
2480000
fabF
acpP
D082_24950
rpl25
purA
D082_25000
rpl31
rps9
rpl13
truA
rpl17
rpoA
rps11
TU2877
TU2880
TU2882
TU2887
TU2888
0
2500
10000
22500
40000
62500
90000
Untreated
15°C
42°C
−CO2
Dark
−Fe
HL
−N
−P
Exp. P.
Stat. P.

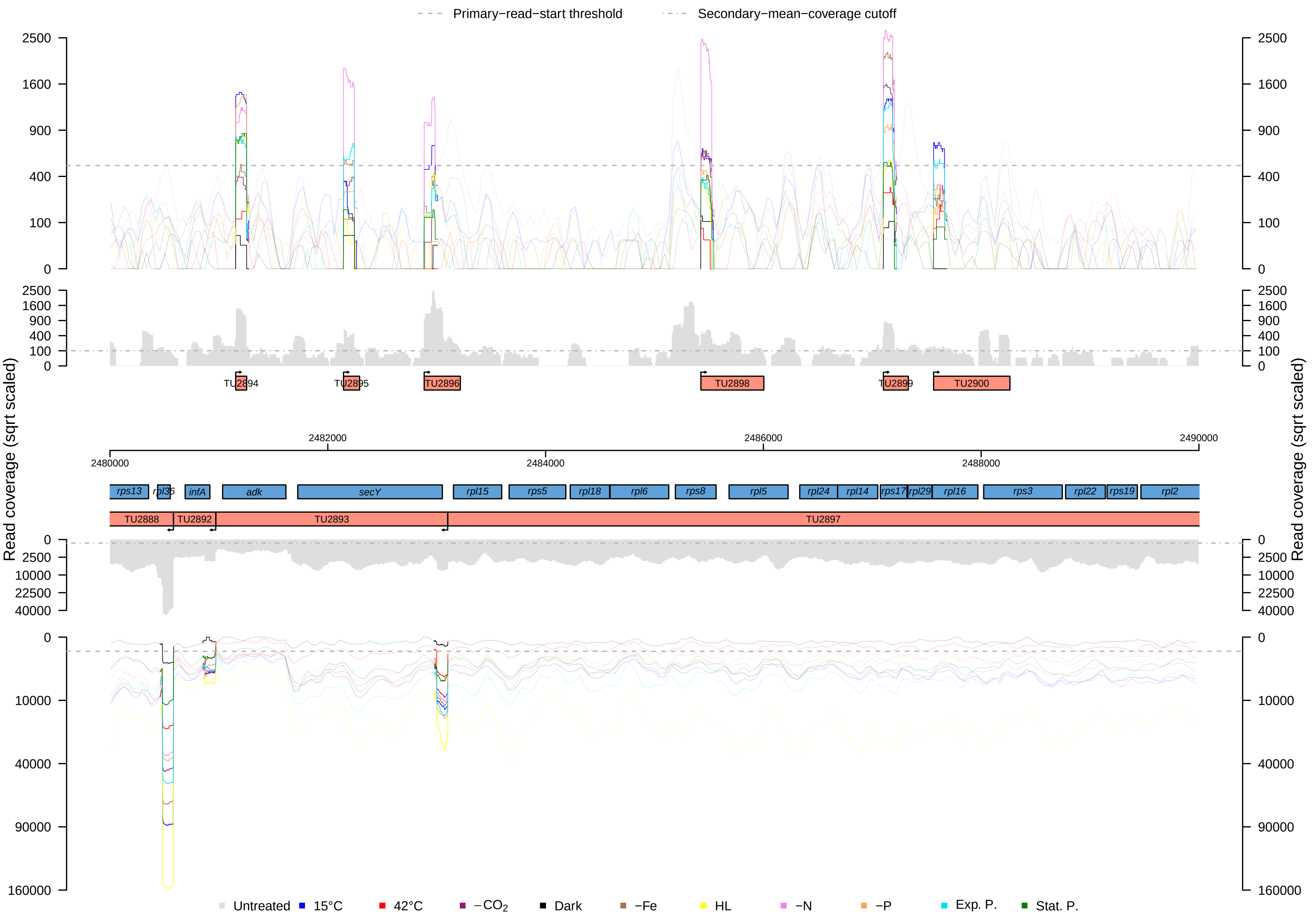

Primary−read−start threshold
Secondary−mean−coverage cutoff
Read coverage (sqrt scaled)
2500
1600
900
400
100
0
TU2894
TU2895
TU2896
TU2898
TU2899
TU2900
2480000
2482000
2484000
2486000
2488000
2490000
rps13
rpl36
infA
adk
secY
rpl15
rps5
rpl18
rpl6
rps8
rpl5
rpl24
rpl14
rps17
rpl29
rpl16
rps3
rpl22
rps19
rpl2
TU2888
TU2892
TU2893
TU2897
10000
22500
40000
90000
160000
Untreated
15°C
42°C
−CO2
Dark
−Fe
HL
−N
−P
Exp. P.
Stat. P.

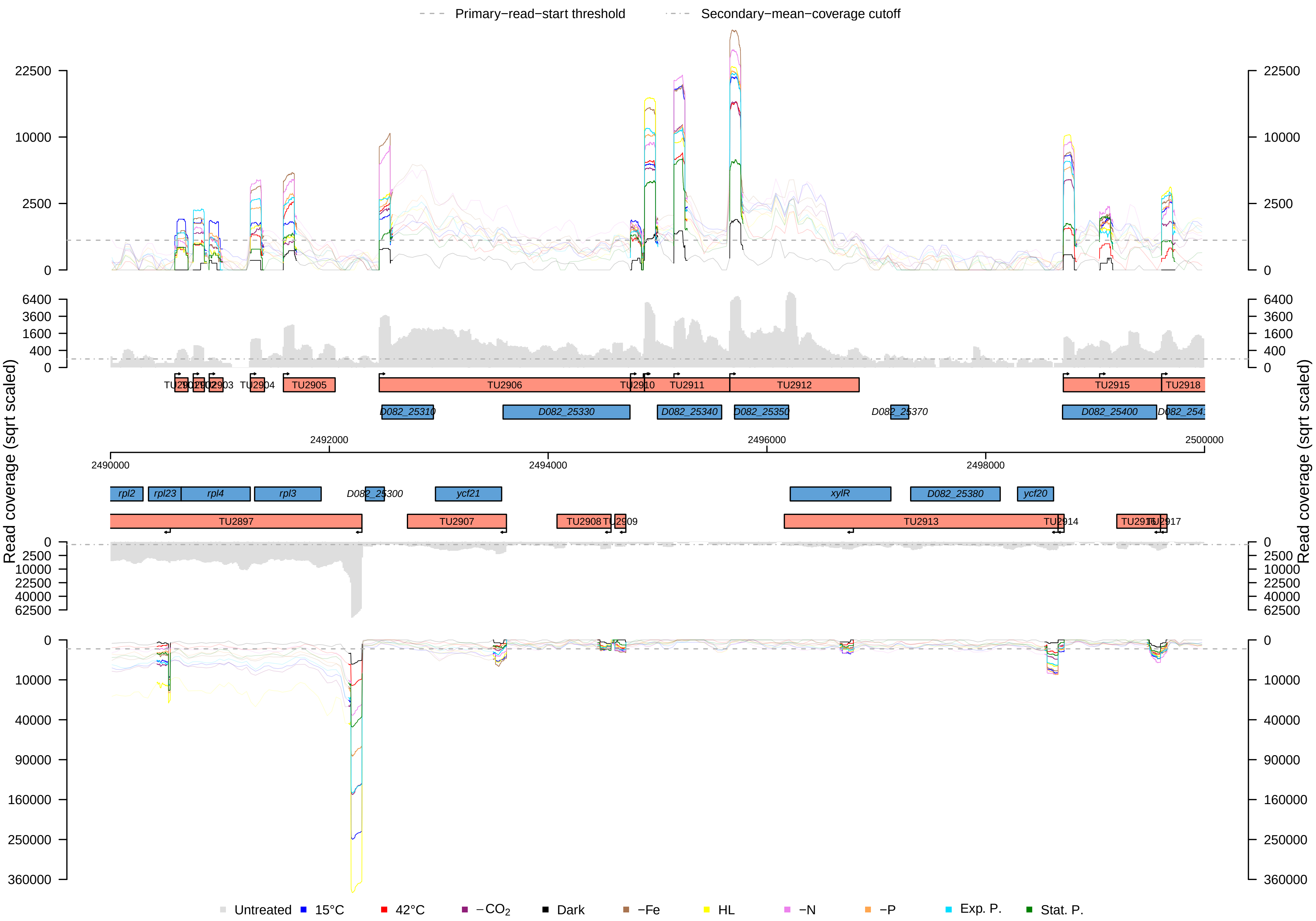

Primary−read−start threshold
Secondary−mean−coverage cutoff
Read coverage (sqrt scaled)
22500
10000
2500
0
6400
3600
1600
400
TU2905
TU2906
TU2911
TU2912
TU2915
TU2918
D082_25310
D082_25330
D082_25340
D082_25350
D082_25370
D082_25400
2490000
2492000
2494000
2496000
2498000
2500000
rpl2
rpl23
rpl4
rpl3
D082_25300
ycf21
xylR
D082_25380
ycf20
TU2897
TU2907
TU2908
TU2913
62500
40000
90000
160000
250000
360000
Untreated
15°C
42°C
−CO_2
Dark
−Fe
HL
−N
−P
Exp. P.
Stat. P.

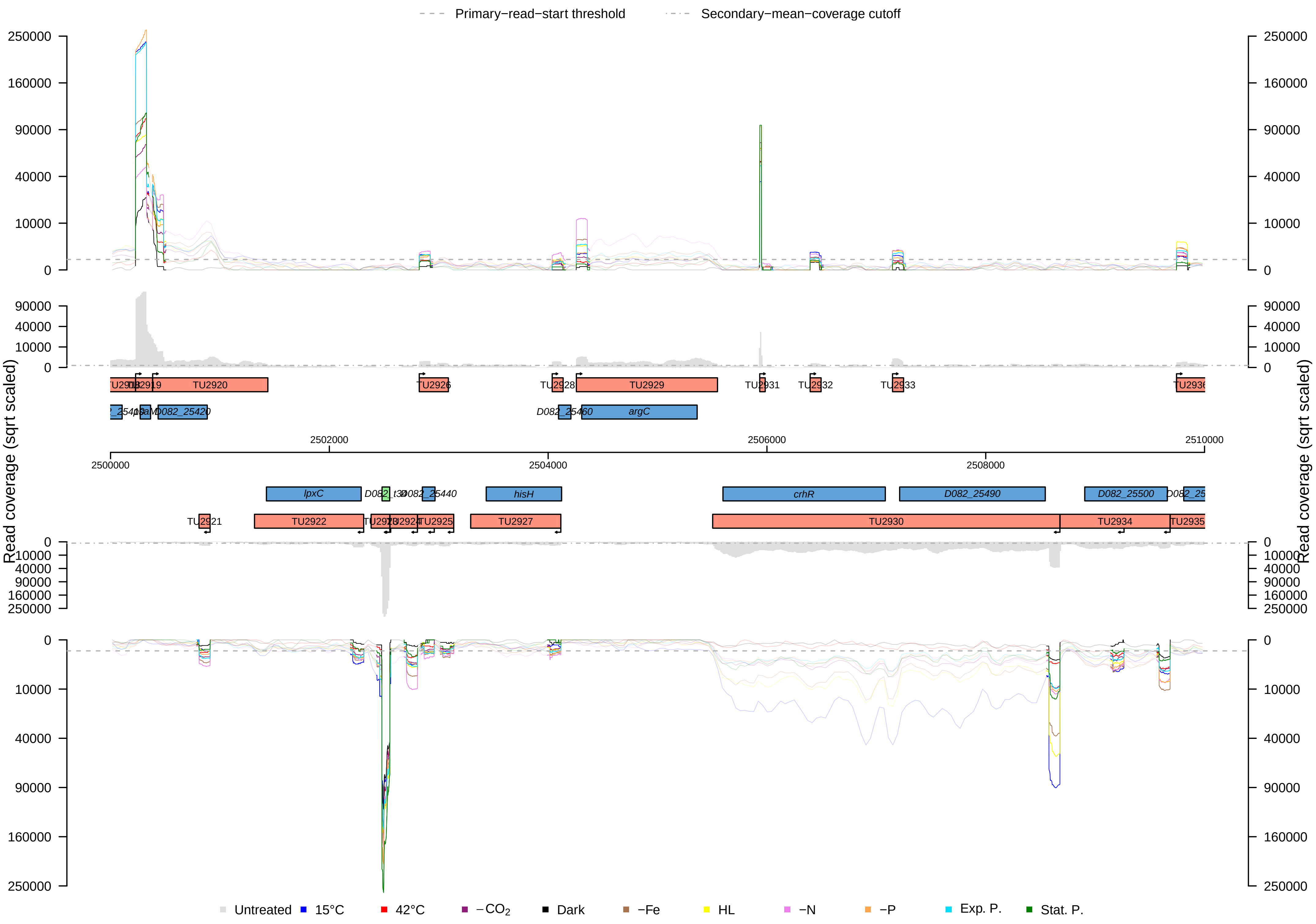

Primary−read−start threshold
Secondary−mean−coverage cutoff
Read coverage (sqrt scaled)
250000
160000
90000
40000
10000
0
TU2918
TU2919
TU2920
TU2926
TU2928
TU2929
TU2931
TU2932
TU2933
psaM
D082_25420
D082_25460
argC
2500000
2502000
2504000
2506000
2508000
2510000
lpxC
D082_25440
hisH
crhR
D082_25490
D082_25500
TU2921
TU2922
TU2924
TU2925
TU2927
TU2930
TU2934
TU2935
Untreated
15°C
42°C
−CO2
Dark
−Fe
HL
−N
−P
Exp. P.
Stat. P.

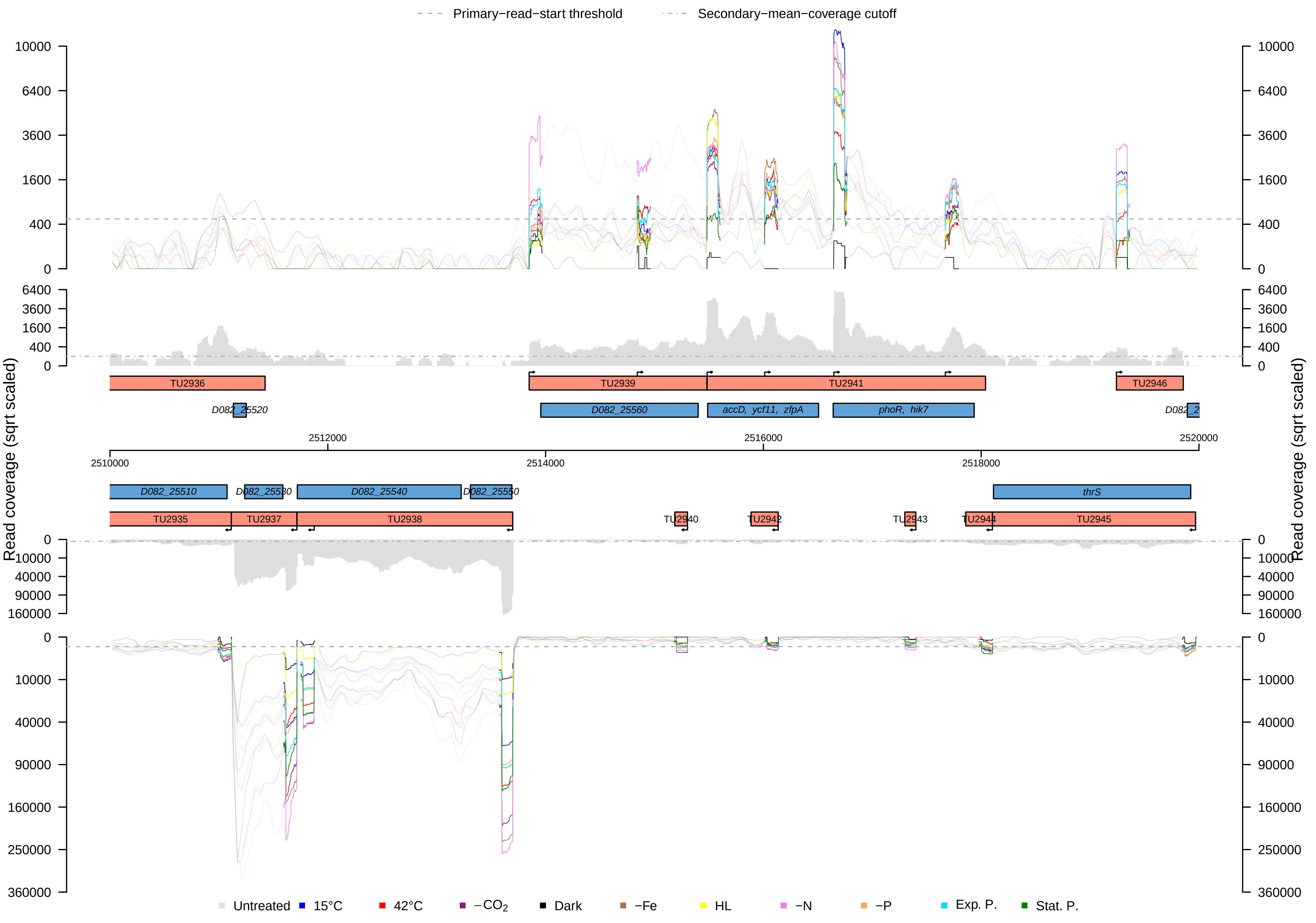

Primary−read−start threshold
Secondary−mean−coverage cutoff
Read coverage (sqrt scaled)
10000
6400
3600
1600
400
0
160000
90000
40000
TU2936
TU2939
TU2941
TU2946
D082_25520
D082_25560
accD, ycf11, zfpA
phoR, hik7
2512000
2516000
2520000
2510000
2514000
2518000
D082_25510
D082_25530
D082_25540
D082_25550
thrS
TU2935
TU2937
TU2938
TU2940
TU2942
TU2943
TU2944
TU2945
250000
360000
Untreated
15°C
42°C
−CO_2
Dark
−Fe
HL
−N
−P
Exp. P.
Stat. P.

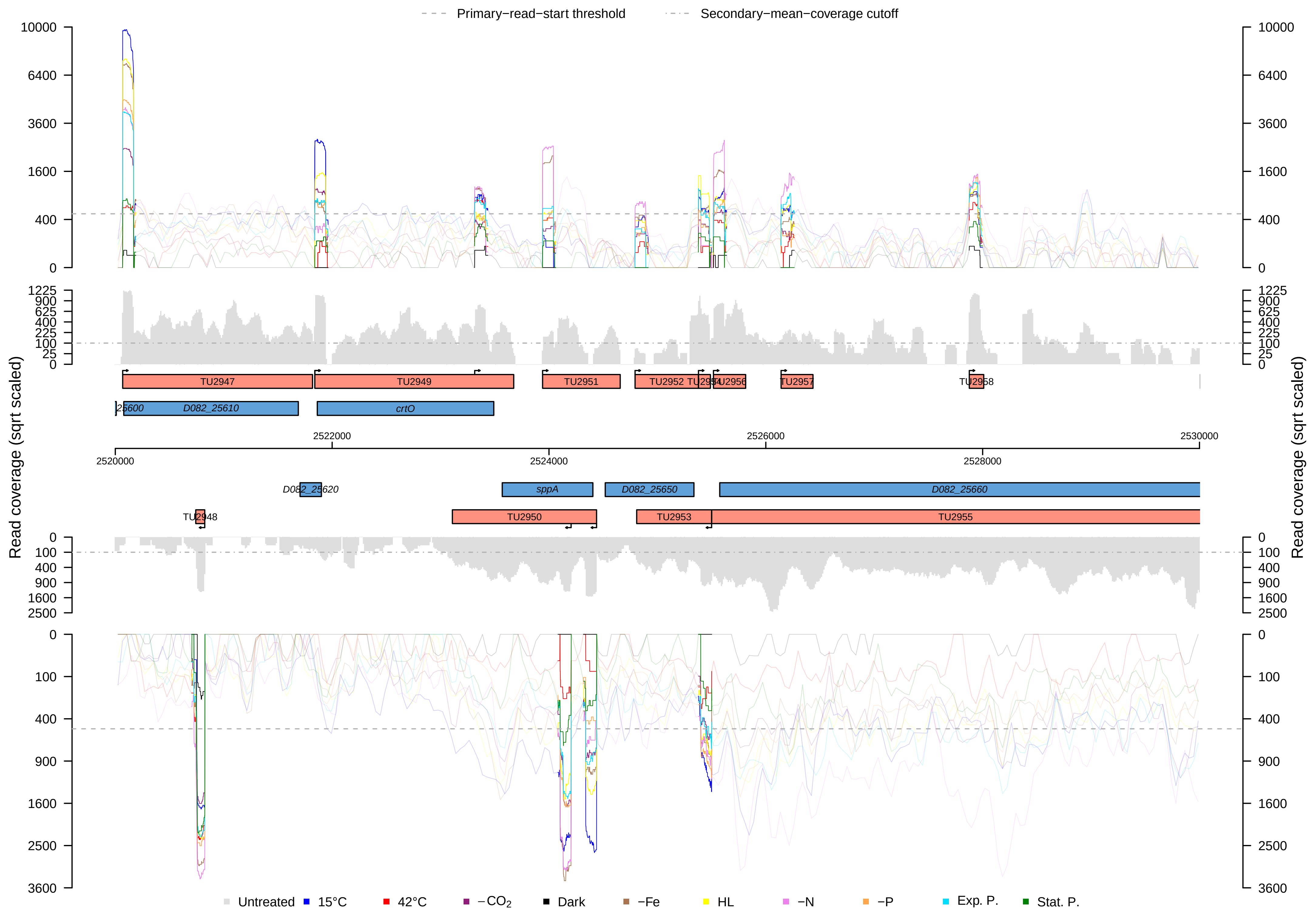

Primary−read−start threshold
Secondary−mean−coverage cutoff
Read coverage (sqrt scaled)
TU2947
TU2949
TU2951
TU2952
TU2954
TU2956
TU2957
TU2958
25600
D082_25610
crtO
D082_25620
sppA
D082_25650
D082_25660
TU2948
TU2950
TU2953
TU2955
2520000
2522000
2524000
2526000
2528000
2530000
Untreated
15°C
42°C
−CO2
Dark
−Fe
HL
−N
−P
Exp. P.
Stat. P.

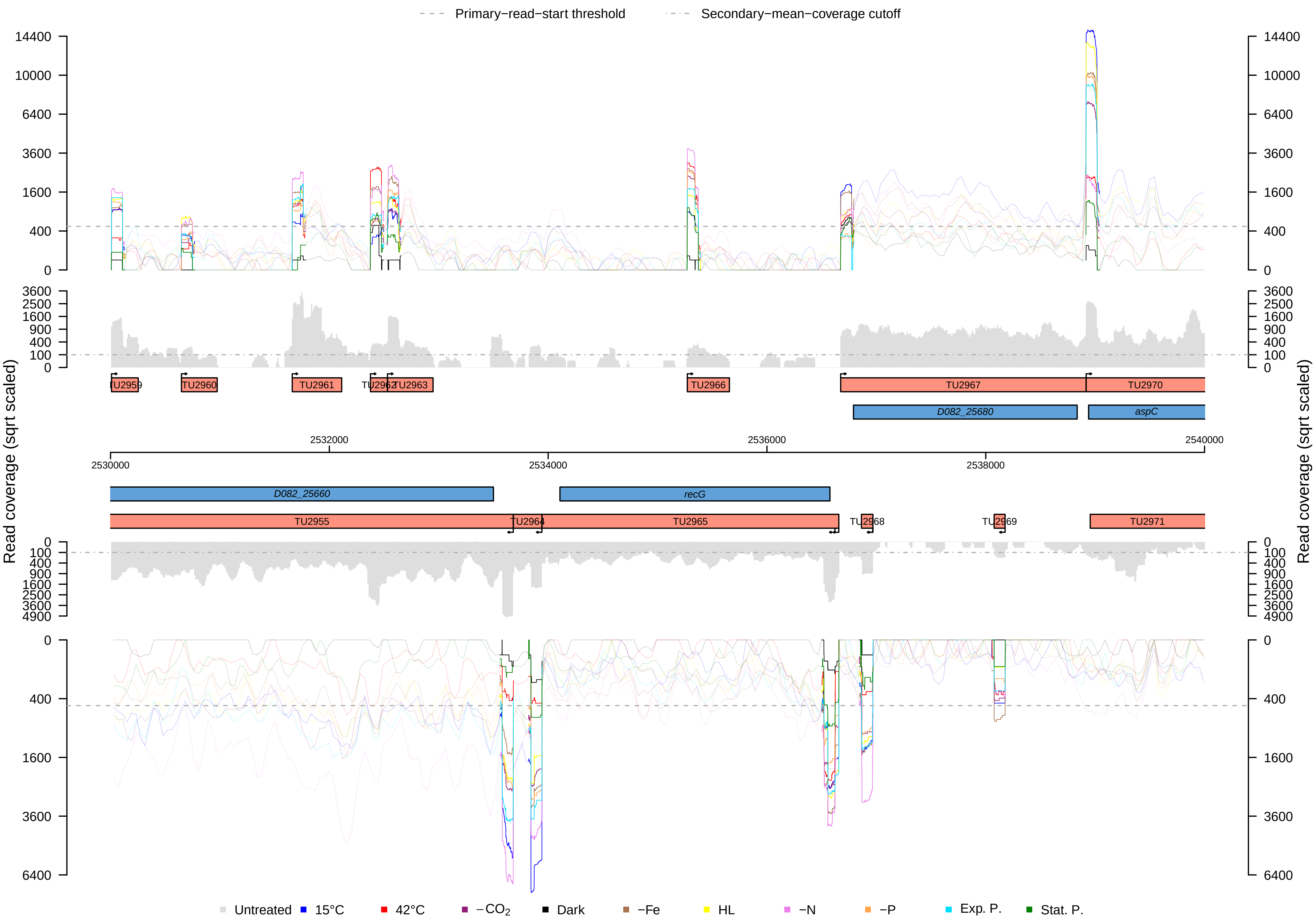

Primary−read−start threshold
Secondary−mean−coverage cutoff
Read coverage (sqrt scaled)
14400
10000
6400
3600
1600
400
0
2500
900
100
4900
TU2959
TU2960
TU2961
TU2962
TU2963
TU2966
TU2967
TU2970
D082_25680
aspC
2530000
2532000
2534000
2536000
2538000
2540000
D082_25660
recG
TU2955
TU2964
TU2965
TU2968
TU2969
TU2971
Untreated
15°C
42°C
−CO_2
Dark
−Fe
HL
−N
−P
Exp. P.
Stat. P.

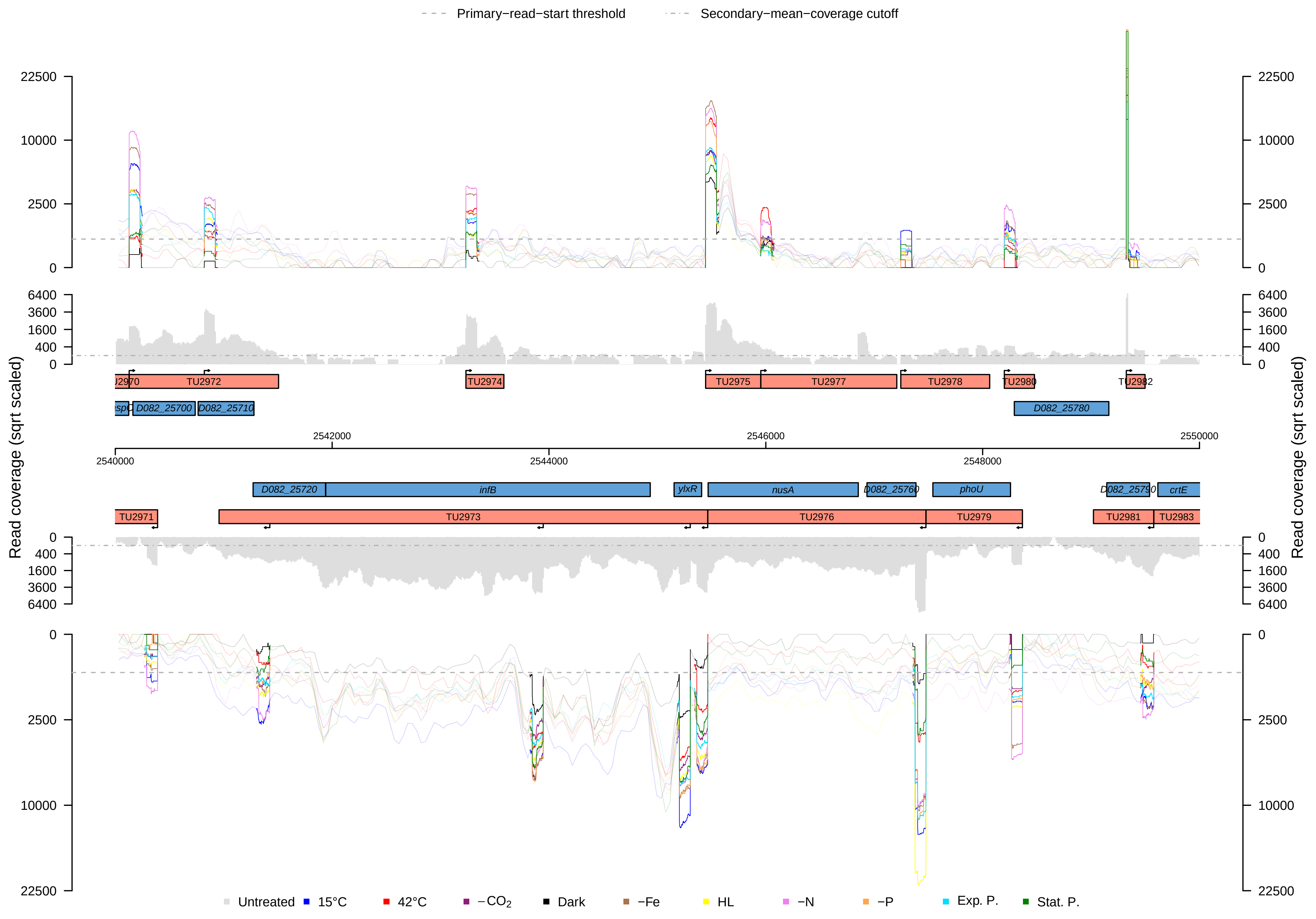

Primary−read−start threshold
Secondary−mean−coverage cutoff
Read coverage (sqrt scaled)
22500
10000
2500
0
6400
3600
1600
400
TU2972
TU2974
TU2975
TU2977
TU2978
TU2980
TU2982
D082_25700
D082_25710
D082_25780
2540000
2542000
2544000
2546000
2548000
2550000
D082_25720
infB
ylxR
nusA
D082_25760
phoU
D082_25790
crtE
TU2971
TU2973
TU2976
TU2979
TU2981
TU2983
Untreated
15°C
42°C
−CO_2
Dark
−Fe
HL
−N
−P
Exp. P.
Stat. P.

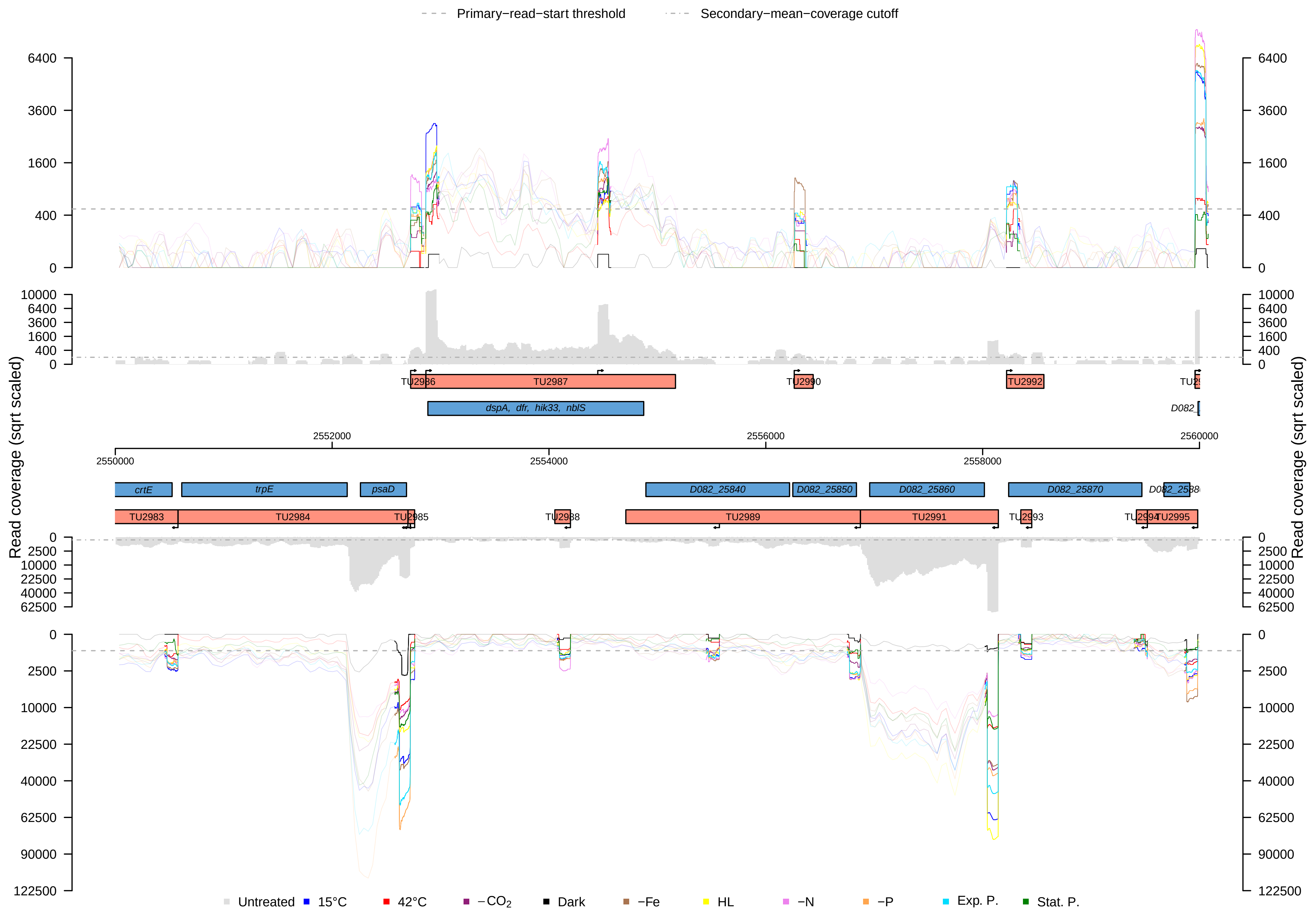

Primary−read−start threshold
Secondary−mean−coverage cutoff
Read coverage (sqrt scaled)
6400
3600
1600
400
0
10000
6400
3600
1600
400
0
TU2986
TU2987
TU2990
TU2992
dspA, dfr, hik33, nblS
2552000
2556000
2560000
2550000
2554000
2558000
crtE
trpE
psaD
D082_25840
D082_25850
D082_25860
D082_25870
TU2983
TU2984
TU2985
TU2988
TU2989
TU2991
TU2993
TU2994
TU2995
0
2500
10000
22500
40000
62500
90000
122500
Untreated
15°C
42°C
−CO2
Dark
−Fe
HL
−N
−P
Exp. P.
Stat. P.

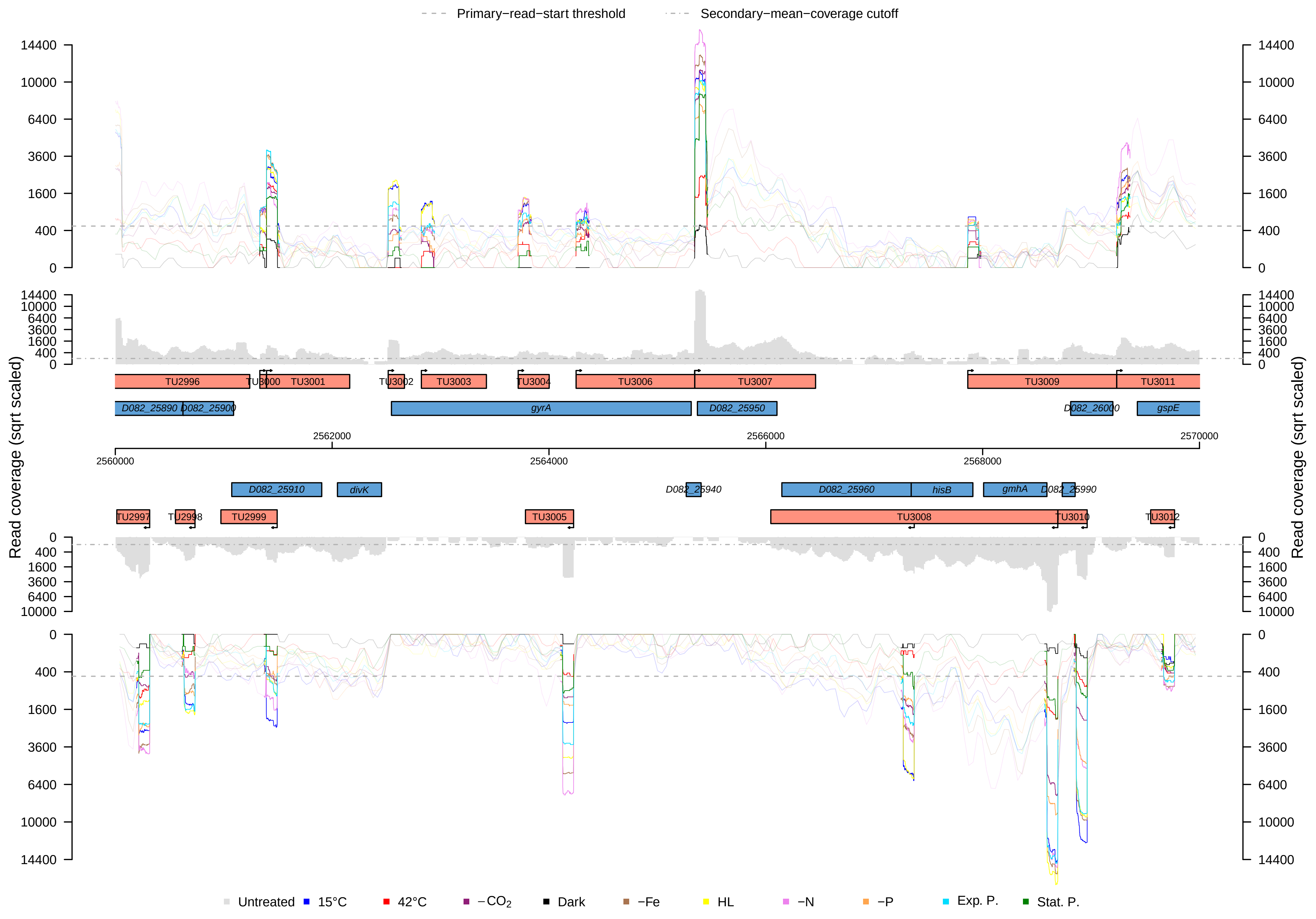

Primary−read−start threshold
Secondary−mean−coverage cutoff
Read coverage (sqrt scaled)
TU2996
TU3000
TU3001
TU3002
TU3003
TU3004
TU3006
TU3007
TU3009
TU3011
D082_25890
D082_25900
gyrA
D082_25950
D082_26000
gspE
2560000
2562000
2564000
2566000
2568000
2570000
D082_25910
divK
D082_25940
D082_25960
hisB
gmhA
D082_25990
TU2997
TU2998
TU2999
TU3005
TU3008
TU3010
TU3012
Untreated
15°C
42°C
−CO2
Dark
−Fe
HL
−N
−P
Exp. P.
Stat. P.

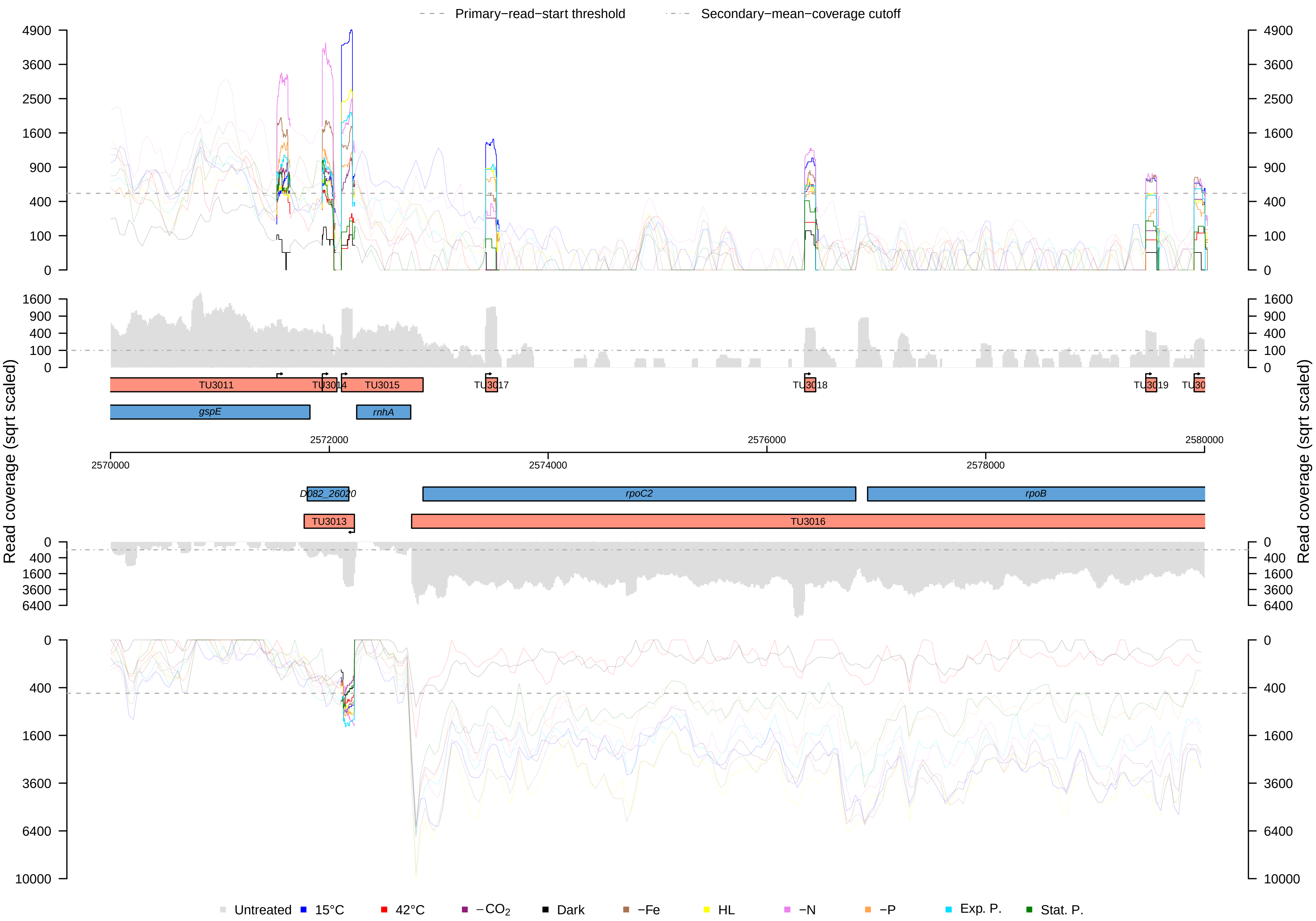

Primary−read−start threshold
Secondary−mean−coverage cutoff
Read coverage (sqrt scaled)
4900
3600
2500
1600
900
400
100
0
TU3011
TU3014
TU3015
TU3017
TU3018
TU3019
gspE
rnhA
2570000
2572000
2574000
2576000
2578000
2580000
D082_26020
rpoC2
rpoB
TU3013
TU3016
6400
10000
Untreated
15°C
42°C
−CO2
Dark
−Fe
HL
−N
−P
Exp. P.
Stat. P.

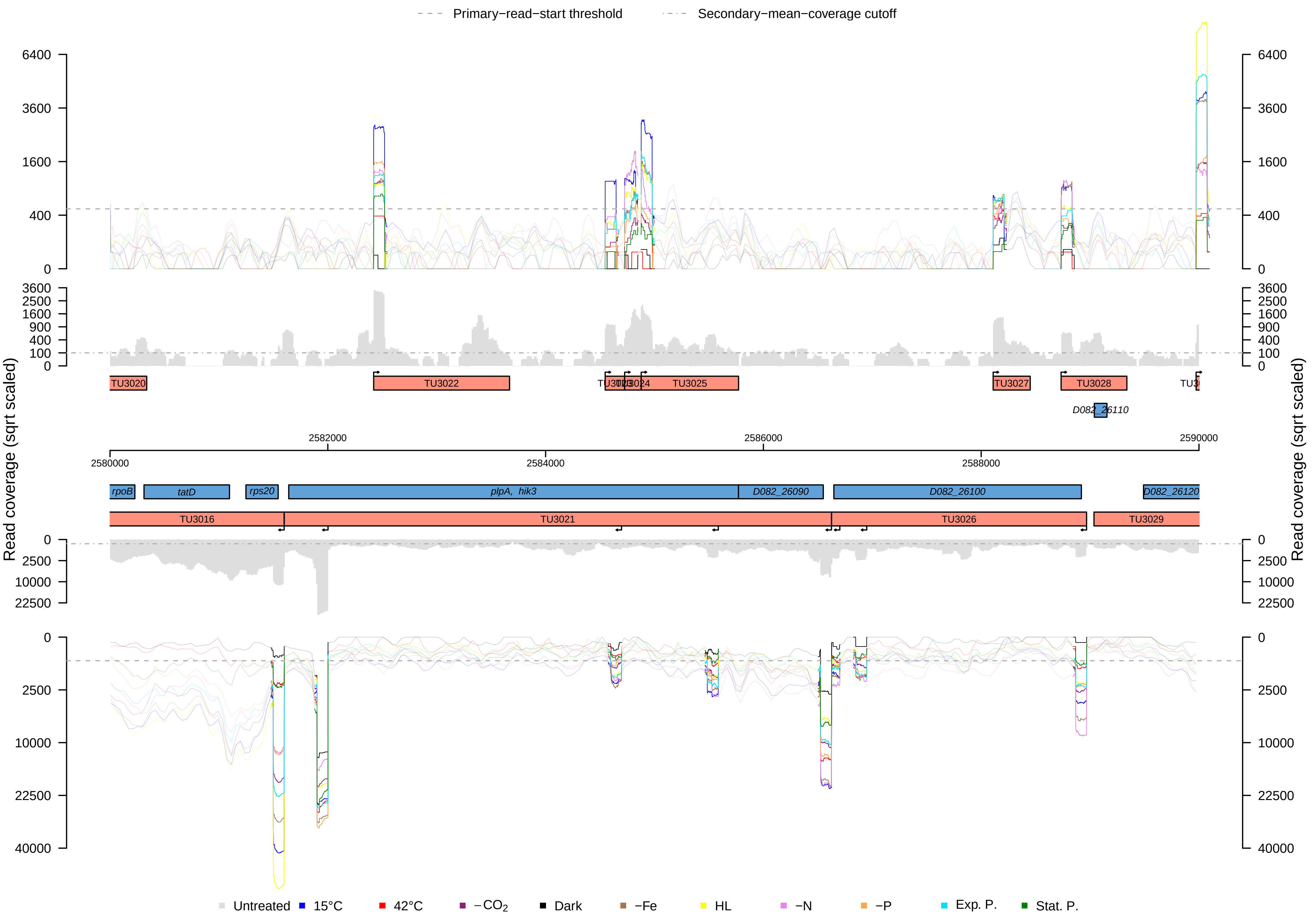

Primary−read−start threshold
Secondary−mean−coverage cutoff
Read coverage (sqrt scaled)
6400
3600
1600
400
0
2500
900
100
10000
22500
40000
TU3020
TU3022
TU3023
TU3024
TU3025
TU3027
TU3028
D082_26110
2580000
2582000
2584000
2586000
2588000
2590000
rpoB
tatD
rps20
plpA, hik3
D082_26090
D082_26100
D082_26120
TU3016
TU3021
TU3026
TU3029
Untreated
15°C
42°C
−CO2
Dark
−Fe
HL
−N
−P
Exp. P.
Stat. P.

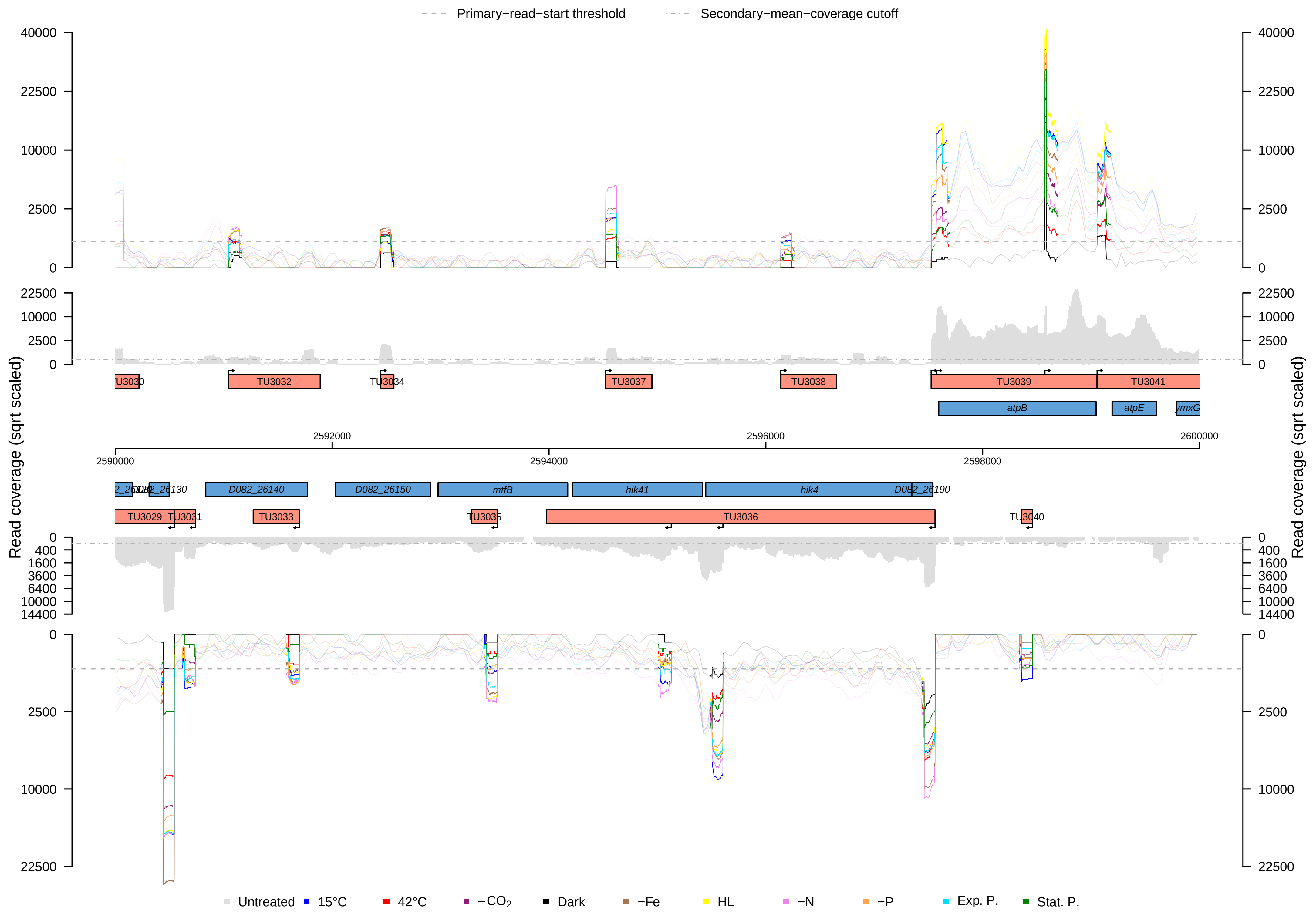

Primary−read−start threshold
Secondary−mean−coverage cutoff
Read coverage (sqrt scaled)
40000
22500
10000
2500
0
TU3030
TU3032
TU3034
TU3037
TU3038
TU3039
TU3041
atpB
atpE
2592000
2596000
2600000
2590000
2594000
2598000
D082_26140
D082_26150
mtfB
hik41
hik4
TU3029
TU3031
TU3033
TU3035
TU3036
TU3040
400
1600
3600
6400
14400
Untreated
15°C
42°C
−CO_2
Dark
−Fe
HL
−N
−P
Exp. P.
Stat. P.

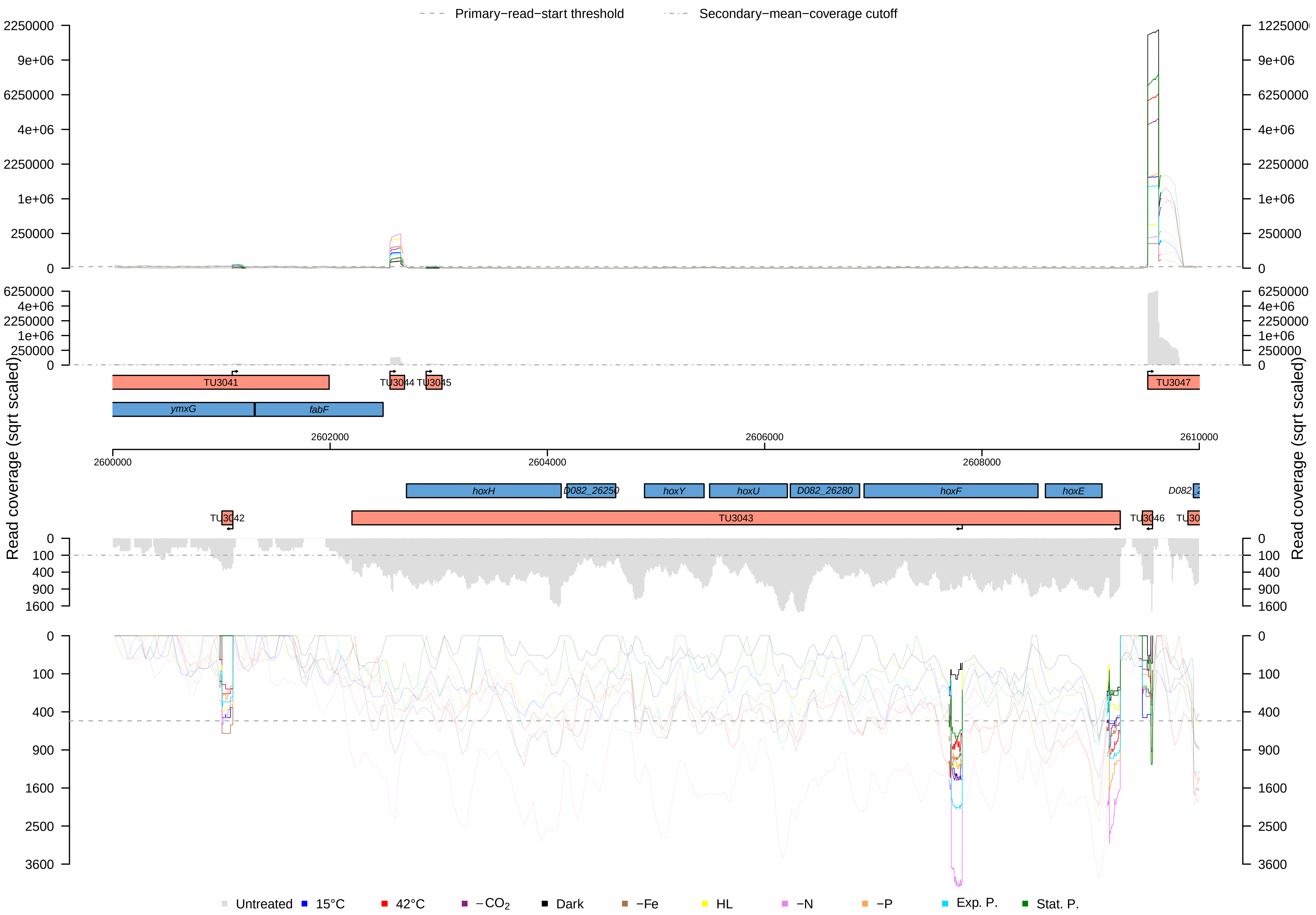

Primary−read−start threshold
Secondary−mean−coverage cutoff
Read coverage (sqrt scaled)
TU3041
TU3044
TU3045
TU3047
ymxG
fabF
2600000
2602000
2604000
2606000
2608000
2610000
hoxH
D082_26250
hoxY
hoxU
D082_26280
hoxF
hoxE
TU3042
TU3043
TU3046
Untreated
15°C
42°C
−CO2
Dark
−Fe
HL
−N
−P
Exp. P.
Stat. P.

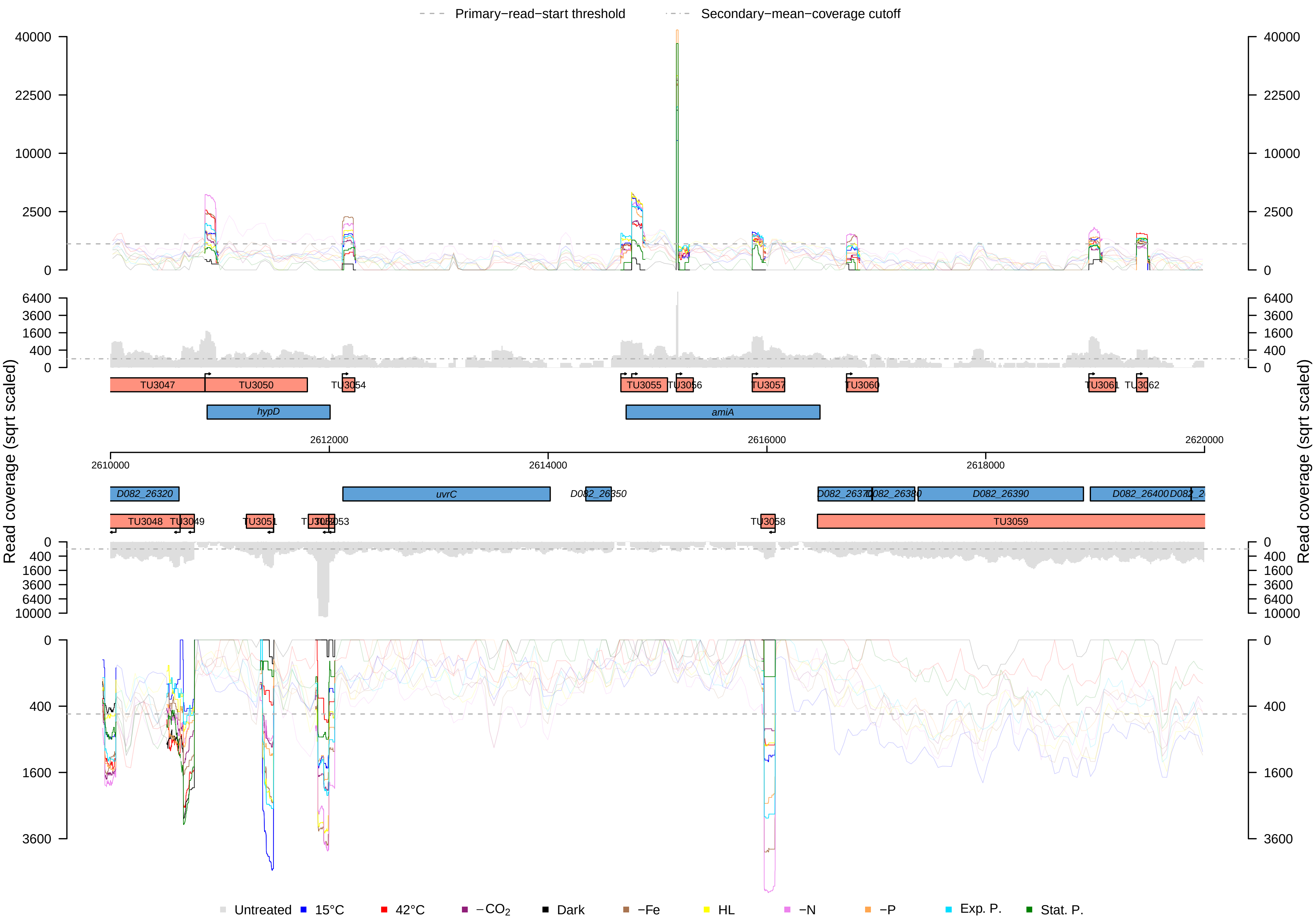

Primary−read−start threshold
Secondary−mean−coverage cutoff
Read coverage (sqrt scaled)
40000
22500
10000
2500
0
6400
3600
1600
400
TU3047
TU3050
TU3054
TU3055
TU3056
TU3057
TU3060
TU3061
TU3062
hypD
amiA
2610000
2612000
2614000
2616000
2618000
2620000
D082_26320
uvrC
D082_26350
D082_26390
TU3048
TU3049
TU3051
TU3053
TU3058
TU3059
10000
Untreated
15°C
42°C
−CO2
Dark
−Fe
HL
−N
−P
Exp. P.
Stat. P.

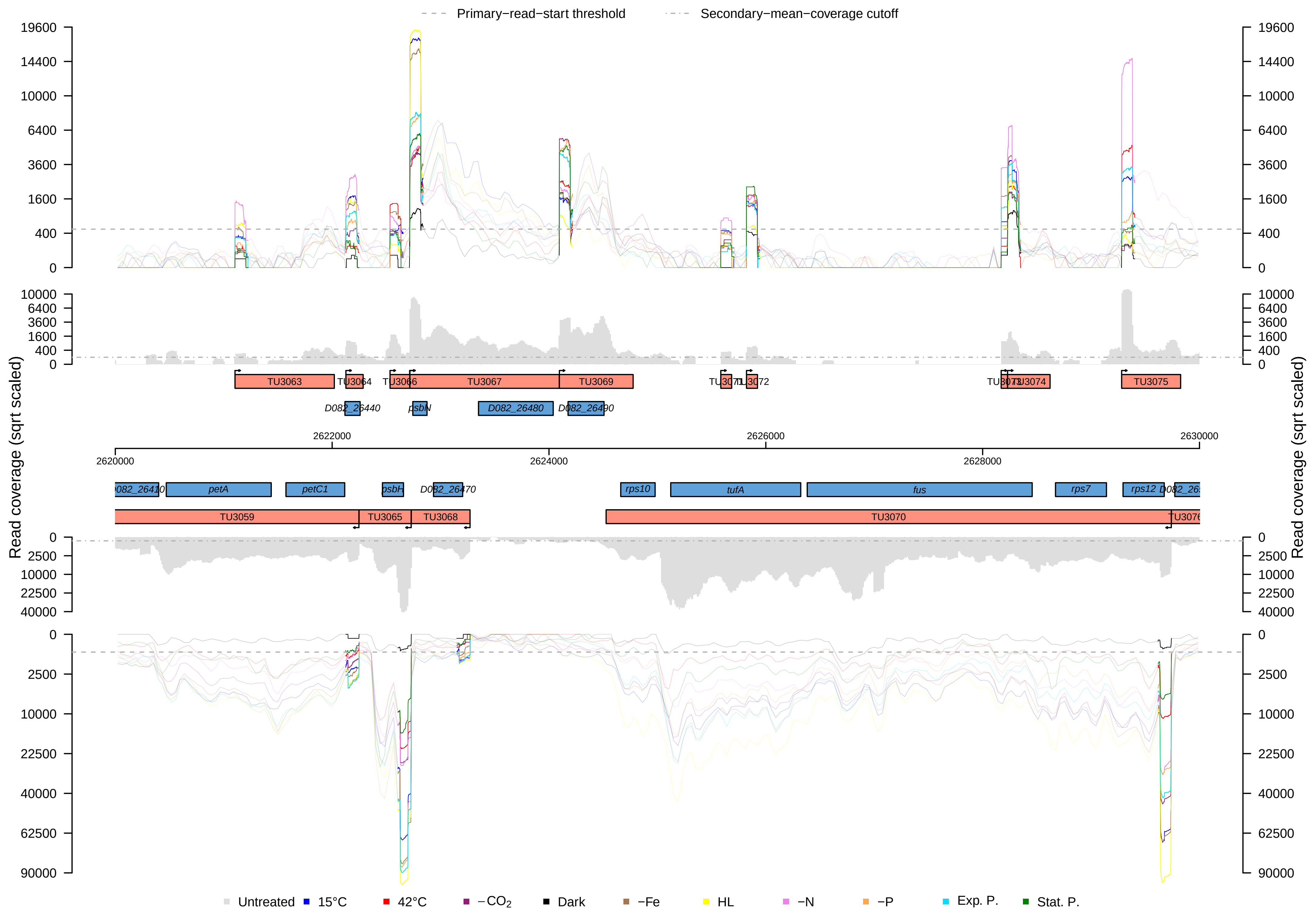

Primary−read−start threshold
Secondary−mean−coverage cutoff
Read coverage (sqrt scaled)
Read coverage (sqrt scaled)
19600
14400
10000
6400
3600
1600
400
0
TU3063
TU3064
TU3066
TU3067
TU3069
TU3071
TU3072
TU3073
TU3074
TU3075
D082_26440
psbN
D082_26480
D082_26490
2620000
2622000
2624000
2626000
2628000
2630000
petA
petC1
psbH
D082_26470
rps10
tufA
fus
rps7
rps12
TU3059
TU3065
TU3068
TU3070
2500
22500
40000
62500
90000
Untreated
15°C
42°C
−CO2
Dark
−Fe
HL
−N
−P
Exp. P.
Stat. P.

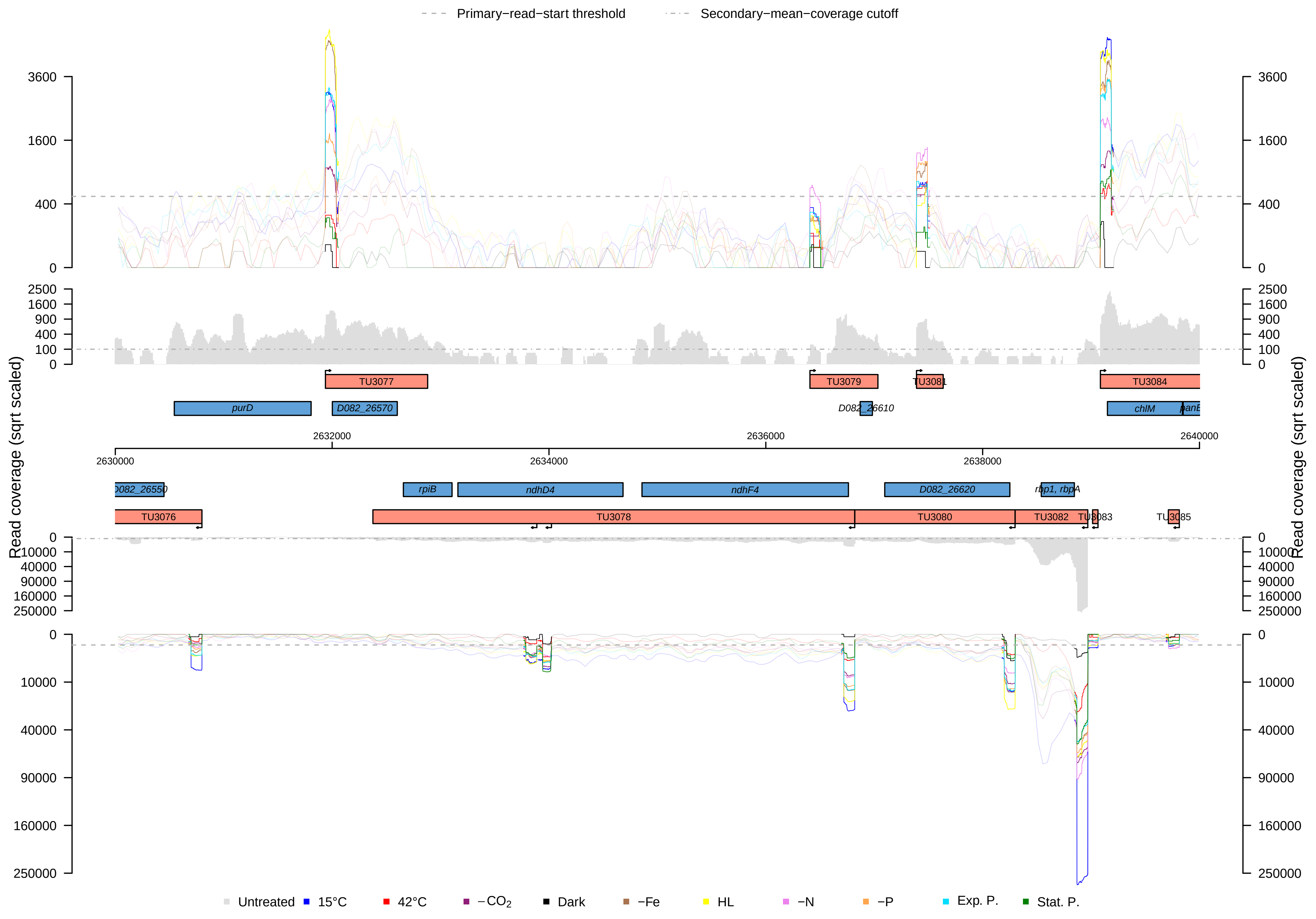

Primary−read−start threshold
Secondary−mean−coverage cutoff
Read coverage (sqrt scaled)
3600
1600
400
0
2500
1600
900
400
100
0
TU3077
TU3079
TU3081
TU3084
purD
D082_26570
D082_26610
chlM
panE
2632000
2636000
2640000
2630000
2634000
2638000
D082_26550
rpiB
ndhD4
ndhF4
D082_26620
rbp1, rbpA
TU3076
TU3078
TU3080
TU3082
TU3083
TU3085
10000
40000
90000
160000
250000
Untreated
15°C
42°C
−CO2
Dark
−Fe
HL
−N
−P
Exp. P.
Stat. P.

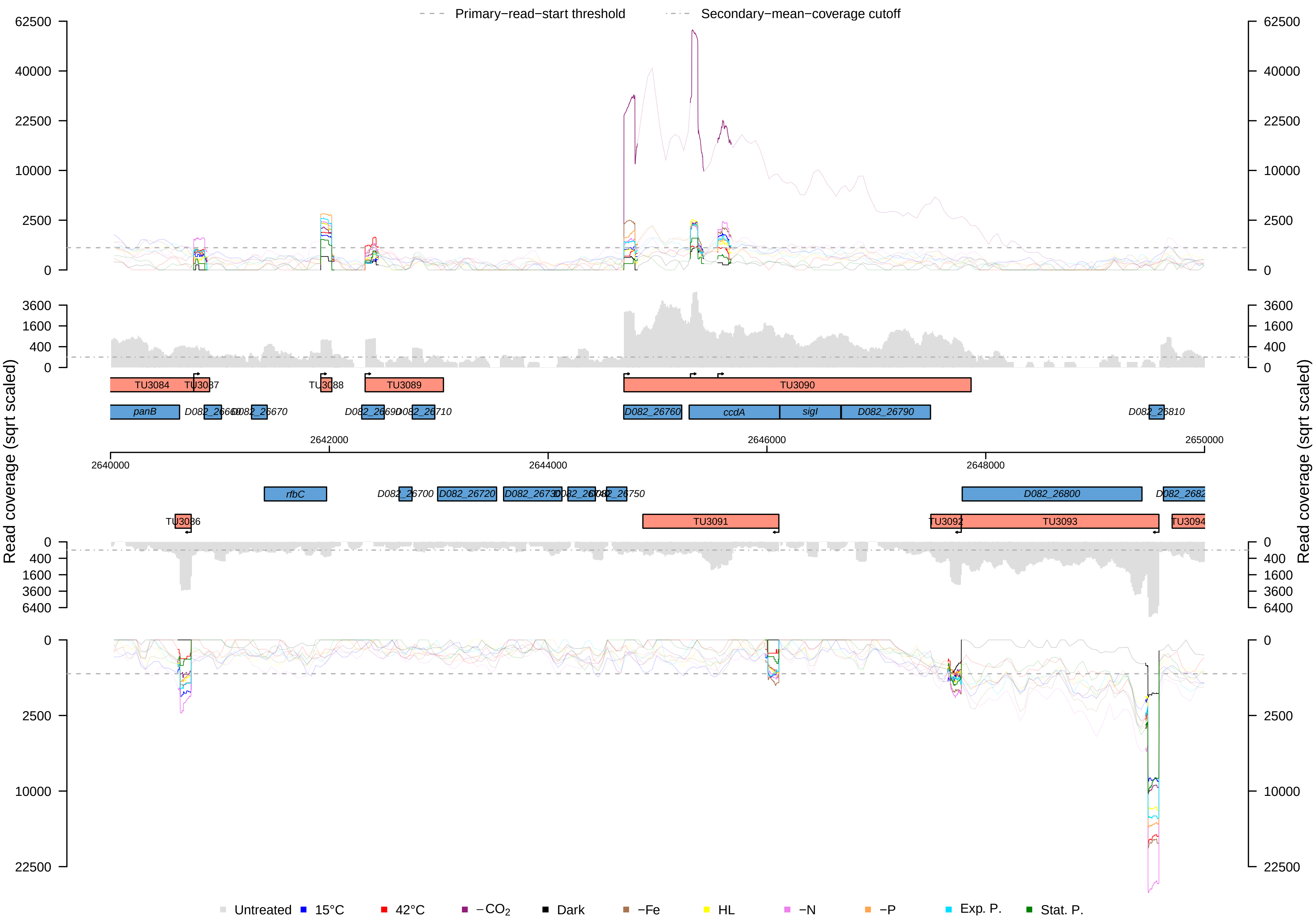

Primary−read−start threshold
Secondary−mean−coverage cutoff
62500
40000
22500
10000
2500
0
3600
1600
400
0
Read coverage (sqrt scaled)
TU3084
TU3087
TU3088
TU3089
TU3090
panB
D082_26660
D082_26670
D082_26690
D082_26710
D082_26760
ccdA
sigI
D082_26790
D082_26810
2640000
2642000
2644000
2646000
2648000
2650000
rfbC
D082_26700
D082_26720
D082_26730
D082_26740
D082_26750
D082_26800
D082_2682
TU3086
TU3091
TU3092
TU3093
TU3094
6400
Untreated
15°C
42°C
−CO_2
Dark
−Fe
HL
−N
−P
Exp. P.
Stat. P.

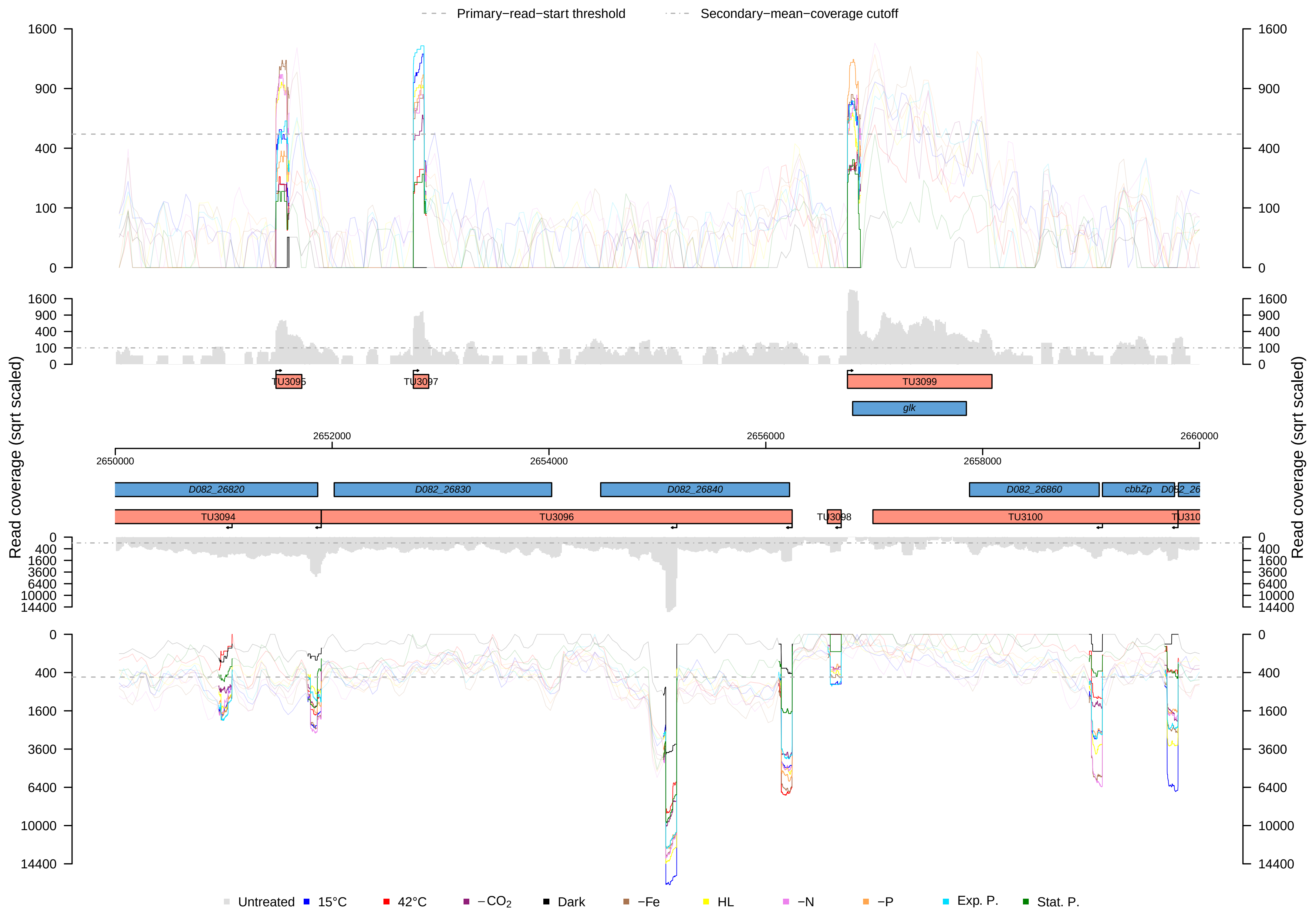

Primary−read−start threshold
Secondary−mean−coverage cutoff
Read coverage (sqrt scaled)
1600
900
400
100
0
TU3095
TU3097
TU3099
glk
2650000
2652000
2654000
2656000
2658000
2660000
D082_26820
D082_26830
D082_26840
D082_26860
cbbZp
TU3094
TU3096
TU3098
TU3100
3600
6400
10000
14400
Untreated
15°C
42°C
−CO2
Dark
−Fe
HL
−N
−P
Exp. P.
Stat. P.

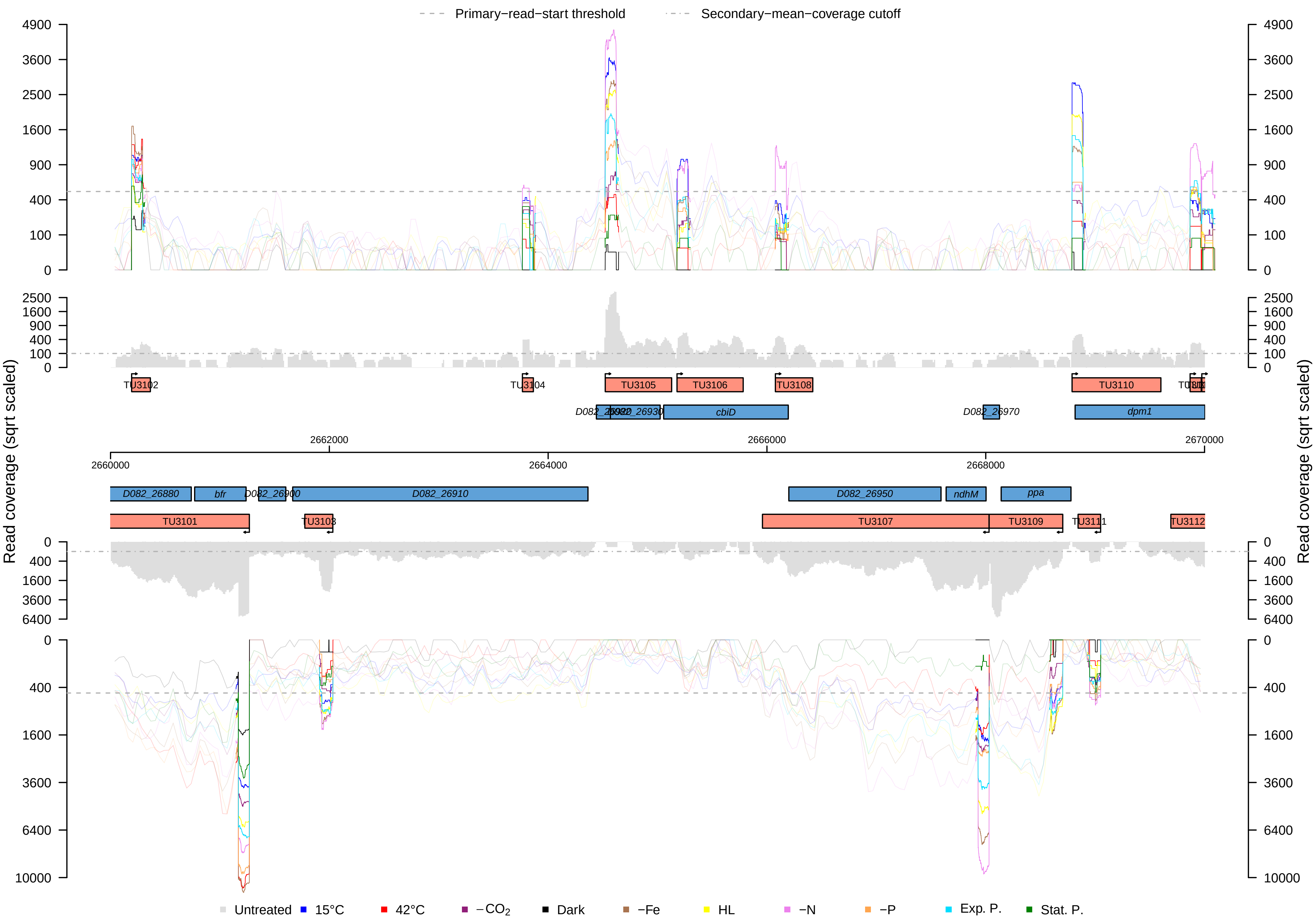

Primary−read−start threshold
Secondary−mean−coverage cutoff
Read coverage (sqrt scaled)
TU3101
TU3102
TU3103
TU3104
TU3105
TU3106
TU3107
TU3108
TU3109
TU3110
TU3111
TU3112
D082_26880
bfr
D082_26900
D082_26910
D082_26930
cbiD
D082_26950
ndhM
D082_26970
ppa
dpm1
2660000
2662000
2664000
2666000
2668000
2670000
Untreated
15°C
42°C
−CO2
Dark
−Fe
HL
−N
−P
Exp. P.
Stat. P.

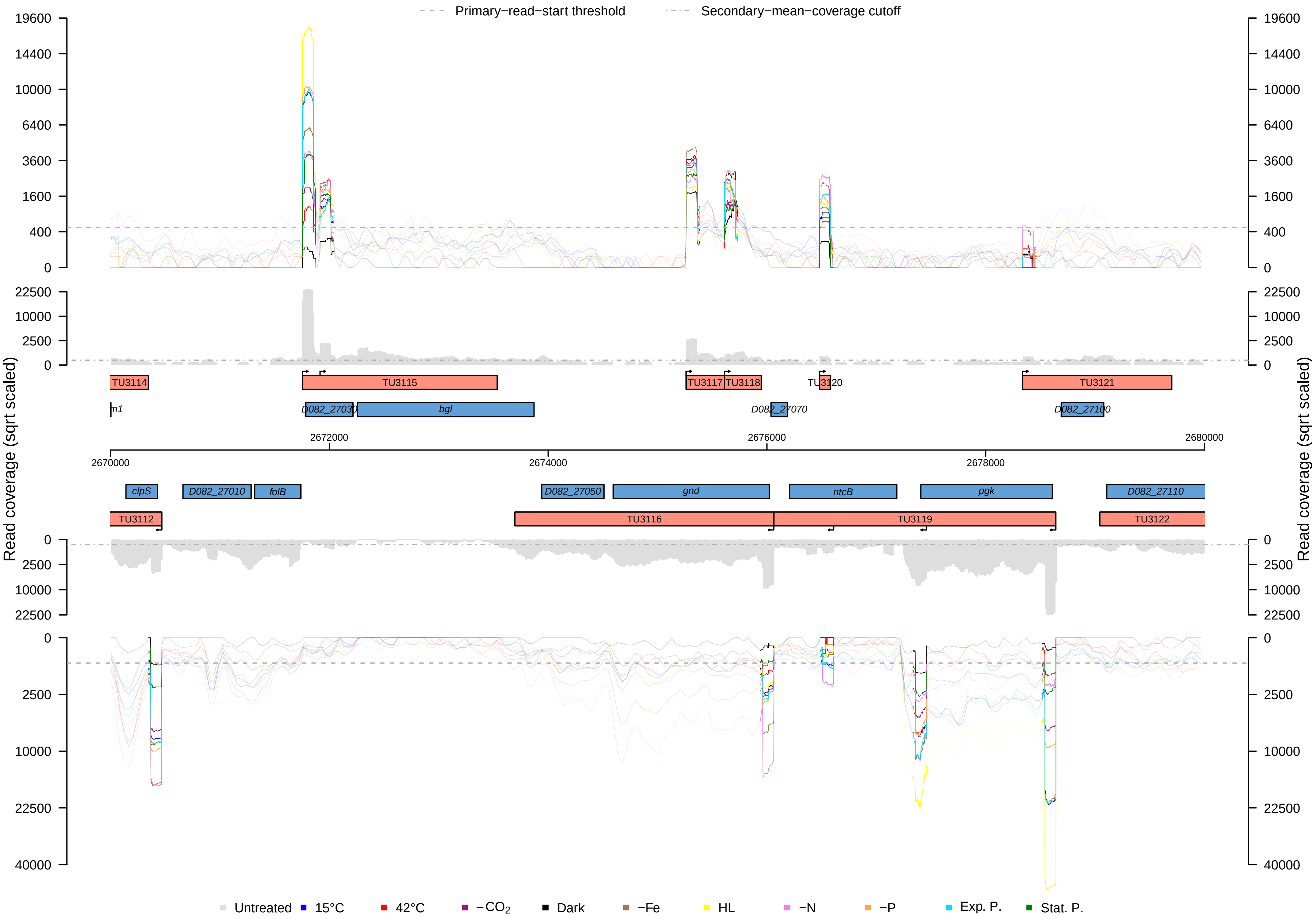

Primary−read−start threshold
Secondary−mean−coverage cutoff
Read coverage (sqrt scaled)
19600
14400
10000
6400
3600
1600
400
0
22500
10000
2500
0
TU3114
TU3115
TU3117
TU3118
TU3120
TU3121
bgl
D082_27070
2672000
2676000
2680000
2670000
2674000
2678000
clpS
D082_27010
folB
D082_27050
gnd
ntcB
pgk
D082_27110
TU3112
TU3116
TU3119
TU3122
40000
Untreated
15°C
42°C
−CO2
Dark
−Fe
HL
−N
−P
Exp. P.
Stat. P.

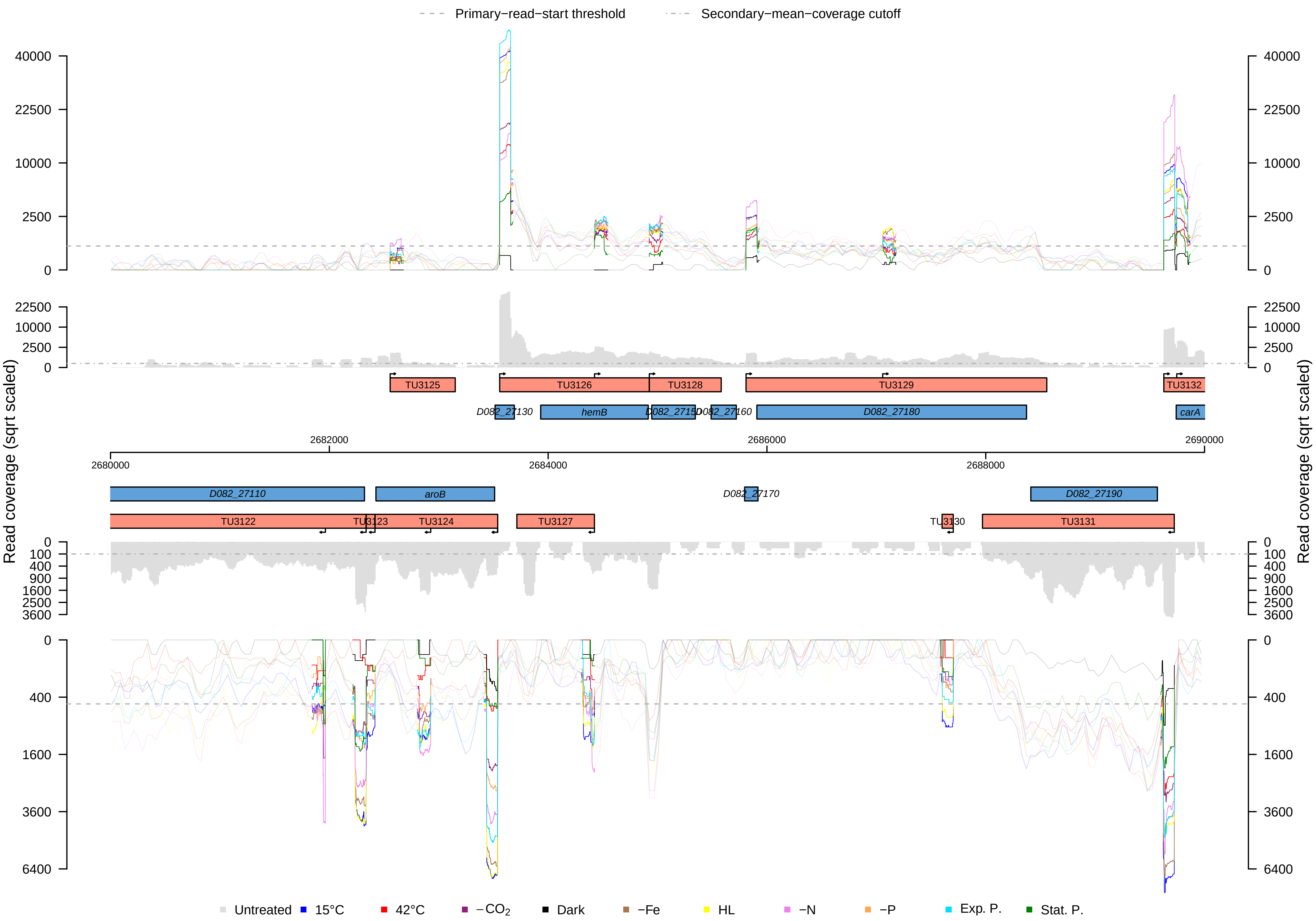

Primary−read−start threshold
Secondary−mean−coverage cutoff
Read coverage (sqrt scaled)
40000
22500
10000
2500
0
100
400
900
1600
2500
3600
6400
TU3125
TU3126
TU3128
TU3129
TU3132
D082_27130
hemB
D082_27150
D082_27160
D082_27180
carA
2680000
2682000
2684000
2686000
2688000
2690000
D082_27110
aroB
D082_27170
D082_27190
TU3122
TU3123
TU3124
TU3127
TU3130
TU3131
Untreated
15°C
42°C
−CO2
Dark
−Fe
HL
−N
−P
Exp. P.
Stat. P.

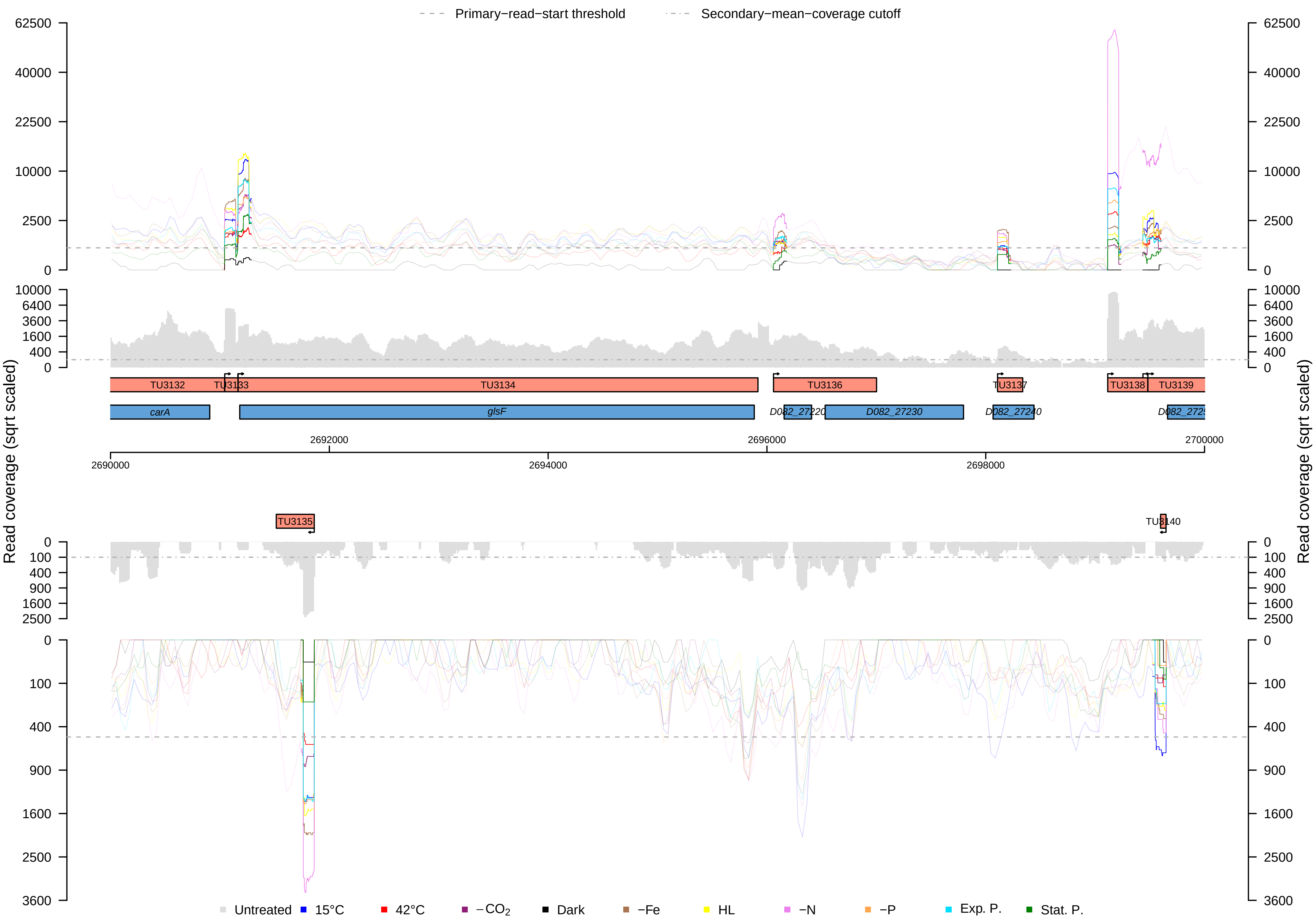

Primary−read−start threshold
Secondary−mean−coverage cutoff
Read coverage (sqrt scaled)
TU3132
TU3133
TU3134
TU3136
TU3137
TU3138
TU3139
carA
glsF
D082_27220
D082_27230
D082_27240
TU3135
TU3140
2690000
2692000
2694000
2696000
2698000
2700000
Untreated
15°C
42°C
−CO2
Dark
−Fe
HL
−N
−P
Exp. P.
Stat. P.

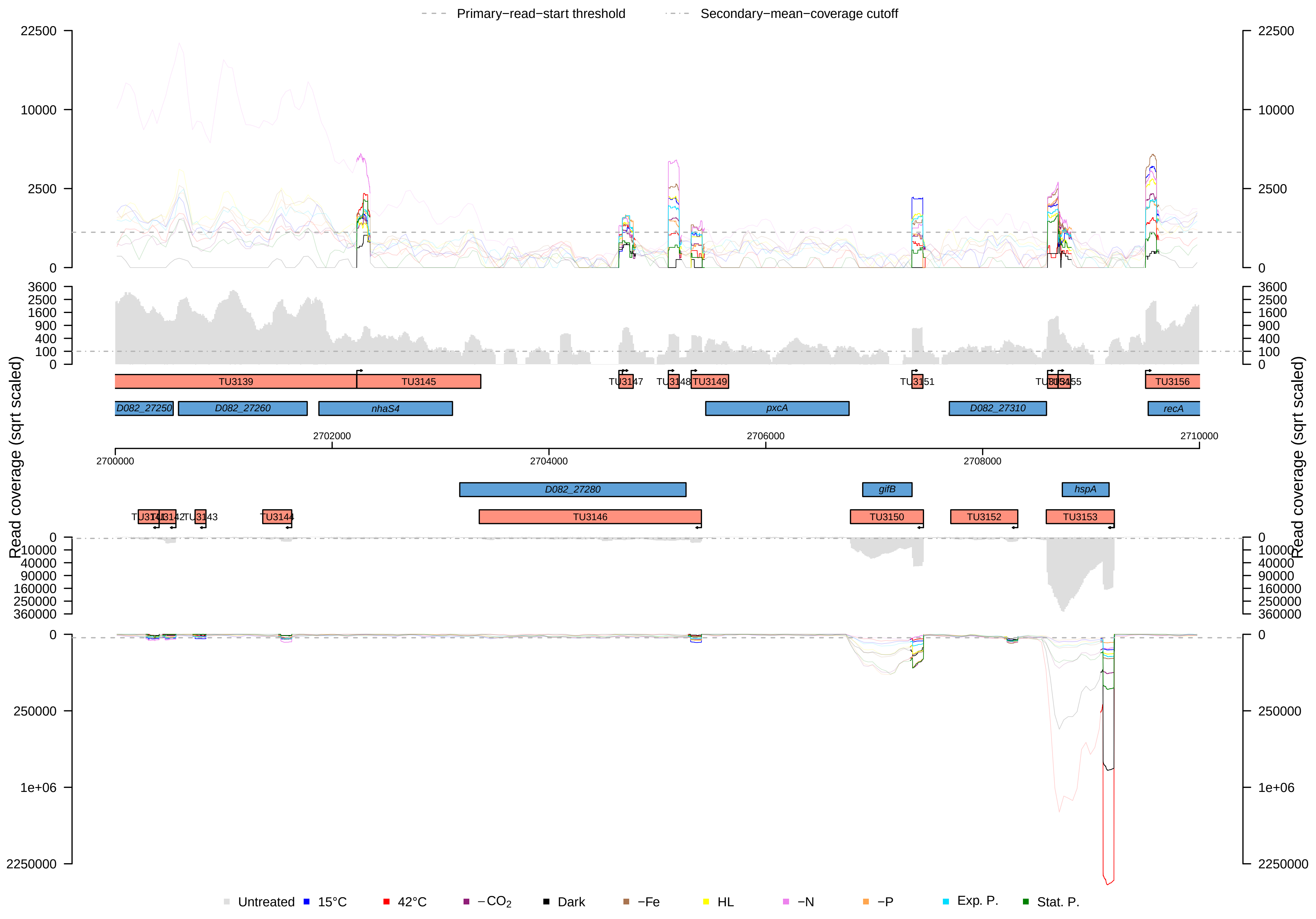

Primary−read−start threshold
Secondary−mean−coverage cutoff
Read coverage (sqrt scaled)
TU3139
TU3145
TU3147
TU3148
TU3149
TU3151
TU3156
D082_27250
D082_27260
nhaS4
pxcA
D082_27310
recA
D082_27280
gifB
hspA
TU3143
TU3144
TU3146
TU3150
TU3152
TU3153
2700000
2702000
2704000
2706000
2708000
2710000
Untreated
15°C
42°C
−CO_2
Dark
−Fe
HL
−N
−P
Exp. P.
Stat. P.

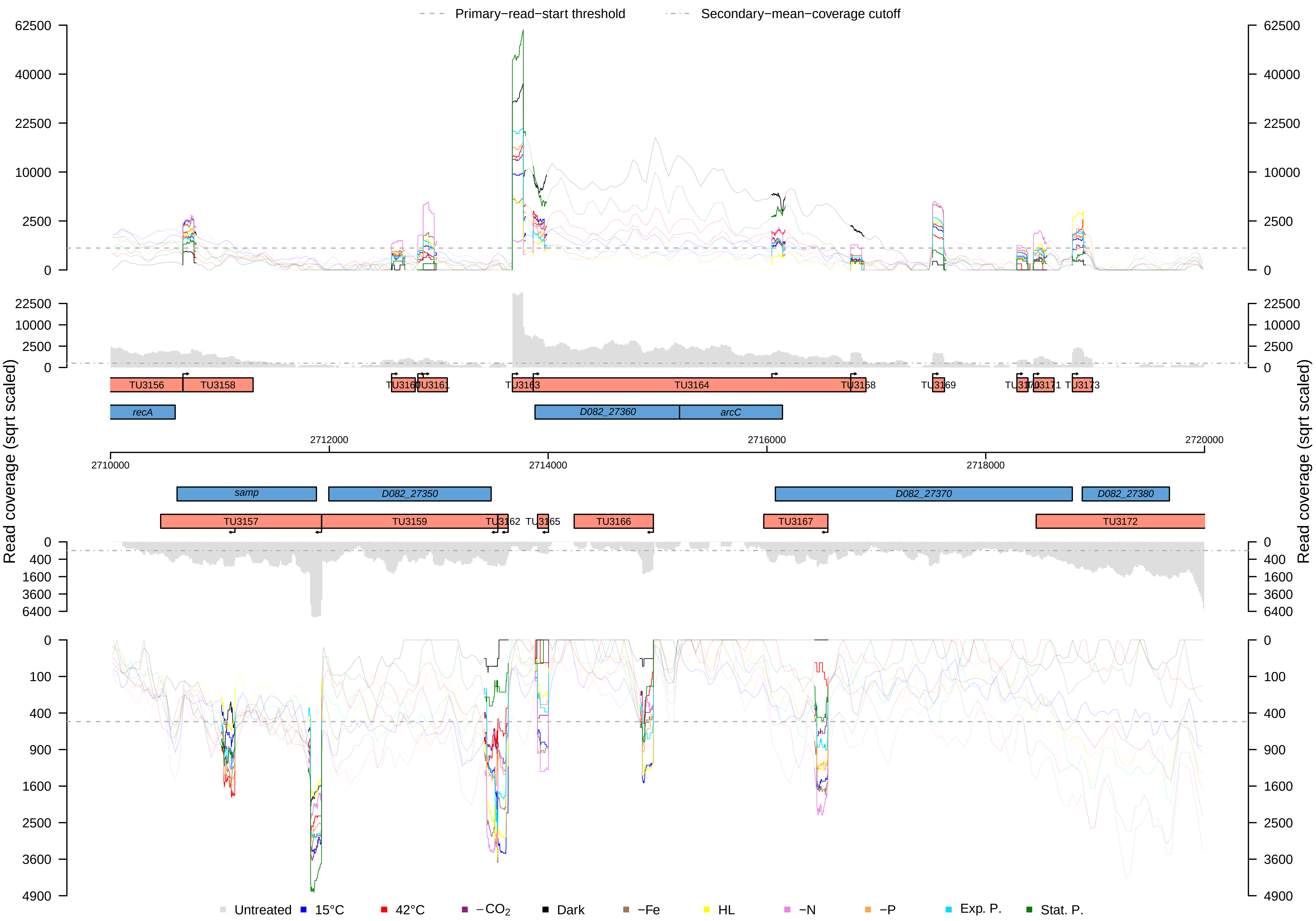

Primary−read−start threshold
Secondary−mean−coverage cutoff
Read coverage (sqrt scaled)
62500
40000
22500
10000
2500
0
TU3156
TU3158
TU3160
TU3161
TU3163
TU3164
TU3168
TU3169
TU3170
TU3171
TU3173
recA
D082_27360
arcC
2710000
2712000
2714000
2716000
2718000
2720000
samp
D082_27350
D082_27370
D082_27380
TU3157
TU3159
TU3162
TU3165
TU3166
TU3167
TU3172
400
1600
3600
6400
100
900
4900
Untreated
15°C
42°C
−CO_2
Dark
−Fe
HL
−N
−P
Exp. P.
Stat. P.

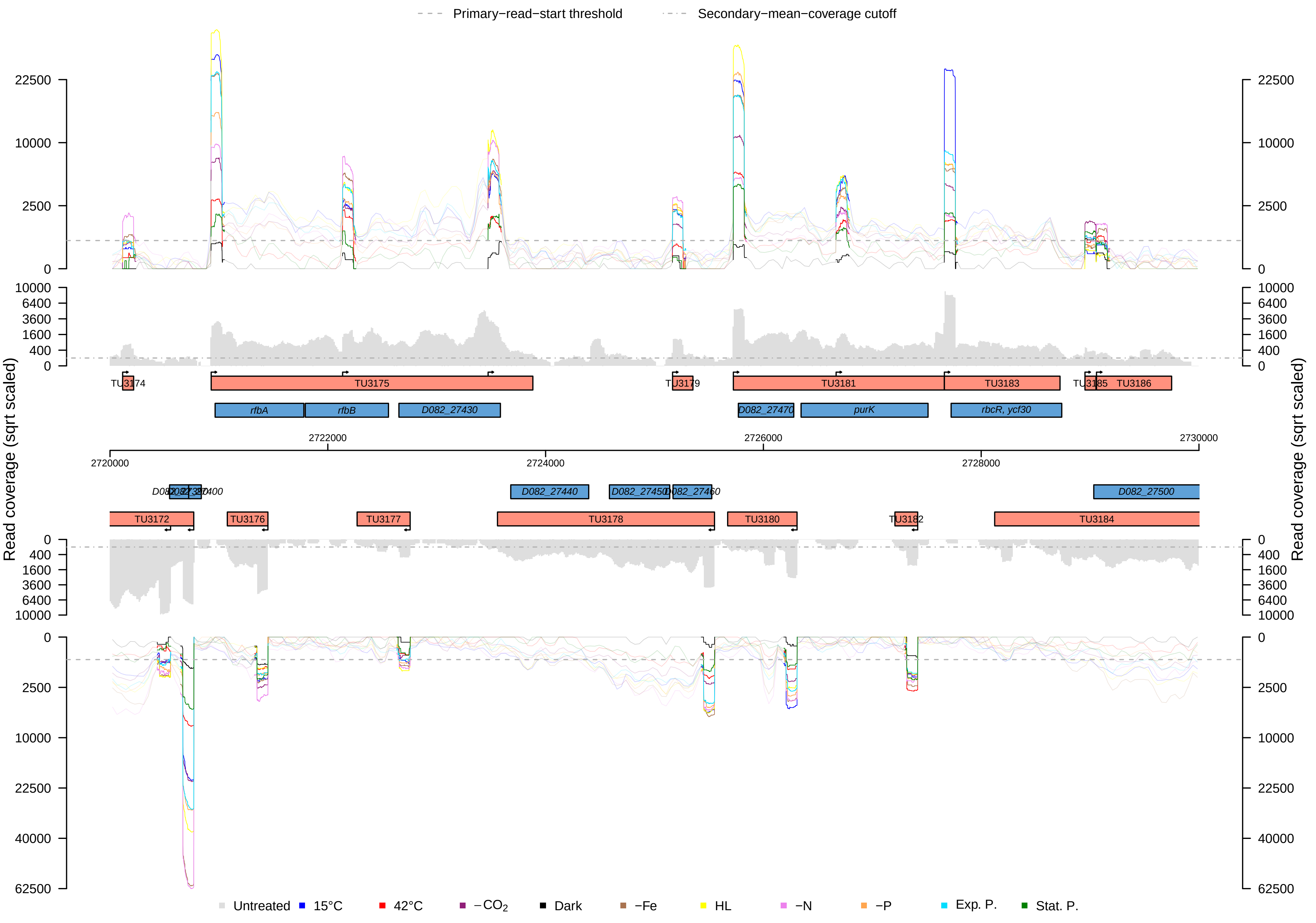

Primary−read−start threshold
Secondary−mean−coverage cutoff
Read coverage (sqrt scaled)
22500
10000
2500
0
10000
6400
3600
1600
400
0
TU3174
TU3175
TU3179
TU3181
TU3183
TU3185
TU3186
rfbA
rfbB
D082_27430
D082_27470
purK
rbcR, ycf30
2720000
2722000
2724000
2726000
2728000
2730000
D082_27440
D082_27450
D082_27460
D082_27500
TU3172
TU3176
TU3177
TU3178
TU3180
TU3182
TU3184
40000
62500
Untreated
15°C
42°C
−CO2
Dark
−Fe
HL
−N
−P
Exp. P.
Stat. P.

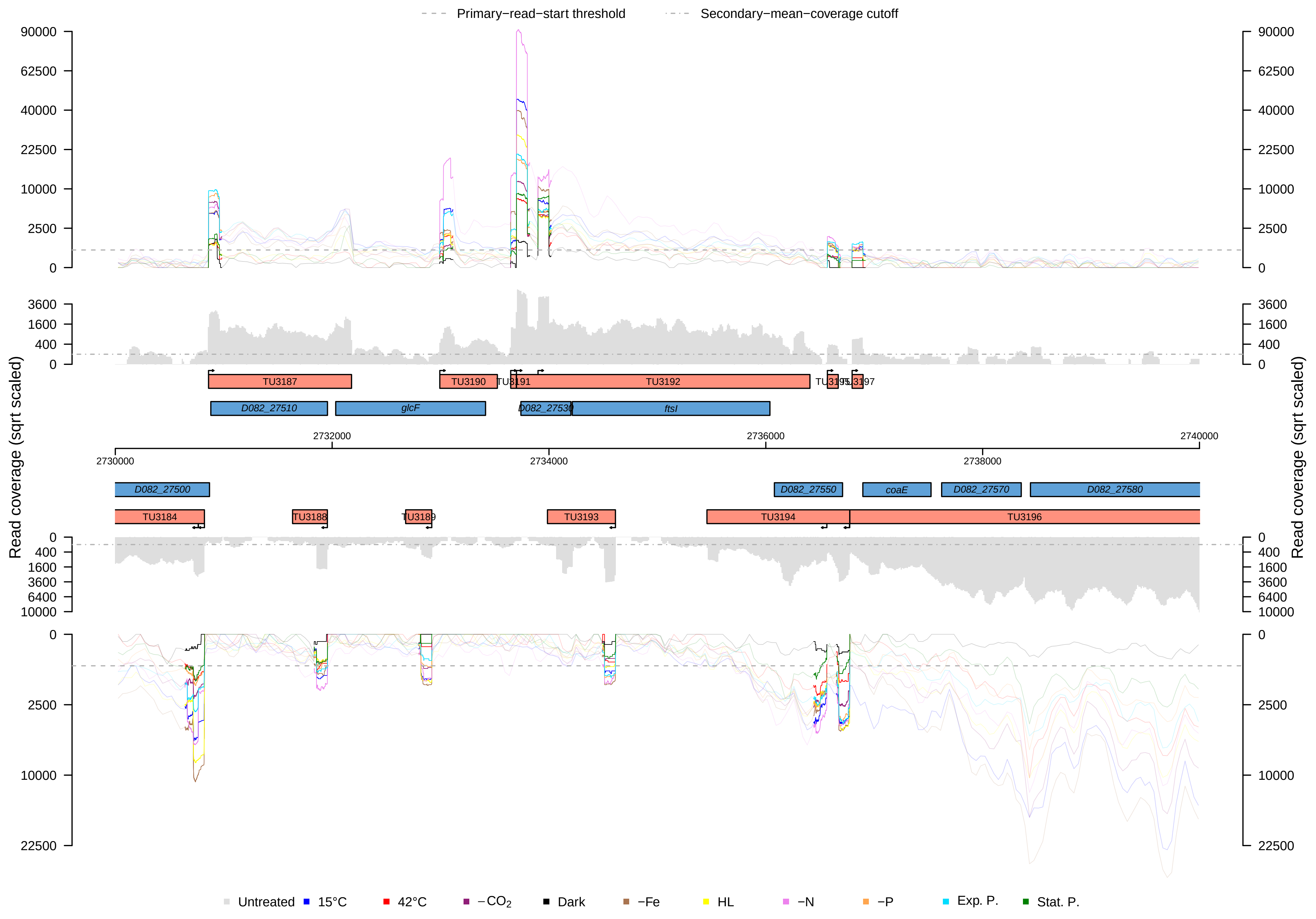

Primary−read−start threshold
Secondary−mean−coverage cutoff
Read coverage (sqrt scaled)
90000
62500
40000
22500
10000
2500
0
3600
1600
400
6400
TU3187
TU3190
TU3191
TU3192
TU3197
D082_27510
glcF
D082_27530
ftsI
2730000
2732000
2734000
2736000
2738000
2740000
D082_27500
D082_27550
coaE
D082_27570
D082_27580
TU3184
TU3188
TU3189
TU3193
TU3194
TU3196
Untreated
15°C
42°C
−CO2
Dark
−Fe
HL
−N
−P
Exp. P.
Stat. P.

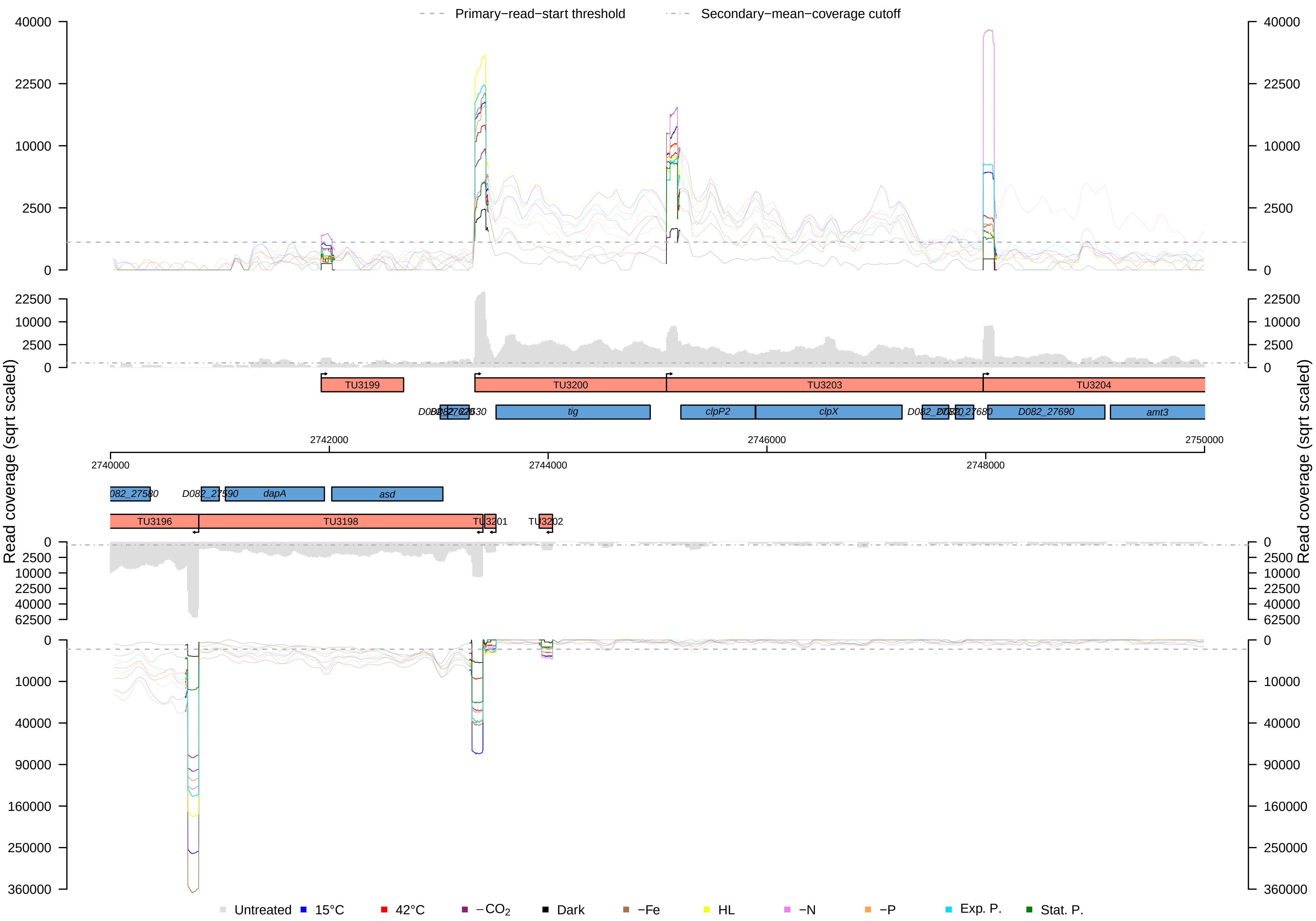

Primary−read−start threshold
Secondary−mean−coverage cutoff
Read coverage (sqrt scaled)
TU3199
TU3200
TU3203
TU3204
tig
clpP2
clpX
D082_27690
amt3
2740000
2742000
2744000
2746000
2748000
2750000
dapA
asd
TU3196
TU3198
TU3201
TU3202
Untreated
15°C
42°C
−CO2
Dark
−Fe
HL
−N
−P
Exp. P.
Stat. P.

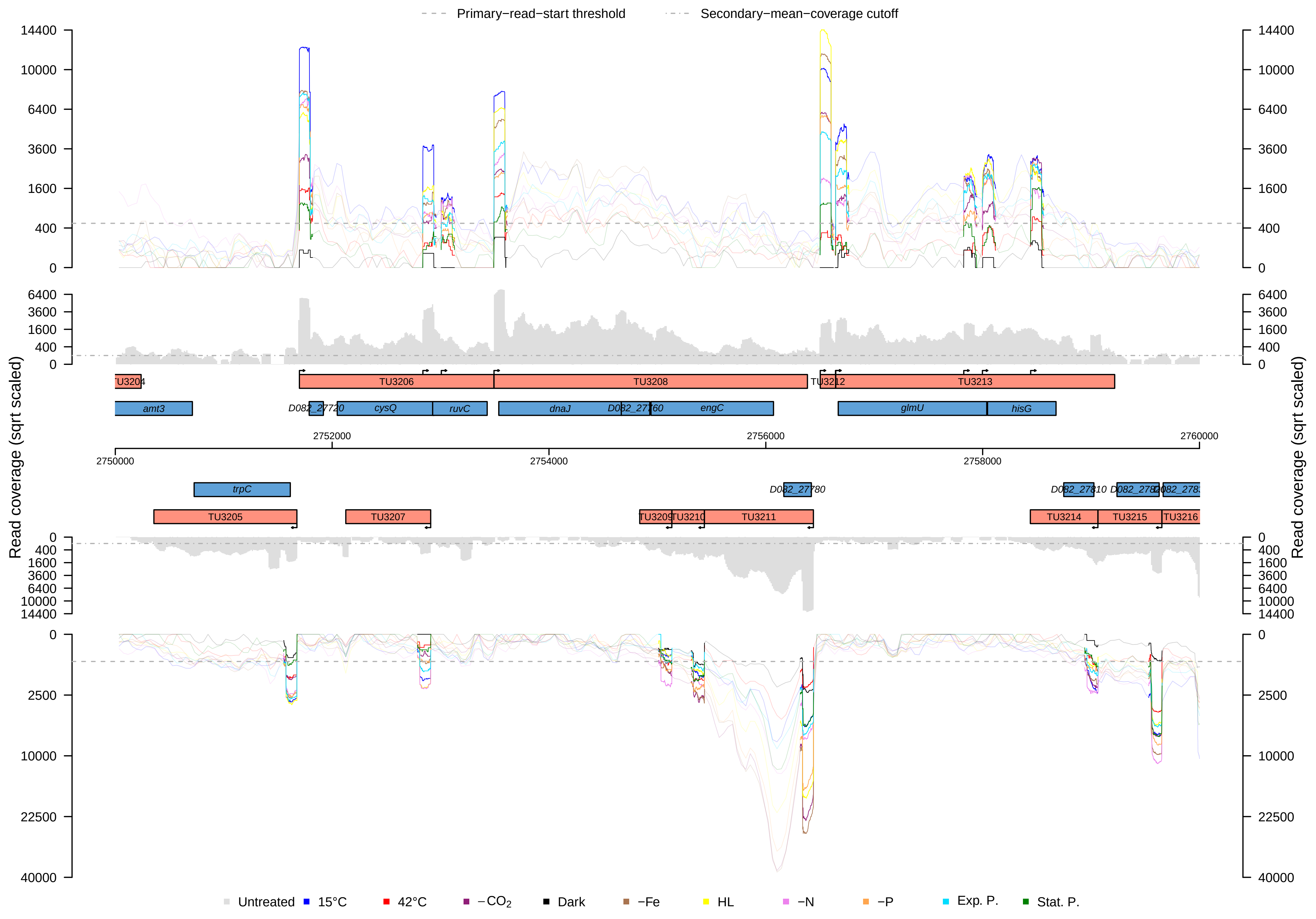

Primary−read−start threshold
Secondary−mean−coverage cutoff
Read coverage (sqrt scaled)
TU3204
TU3206
TU3208
TU3212
TU3213
amt3
D082_27720
cysQ
ruvC
dnaJ
D082_27760
engC
glmU
hisG
2750000
2752000
2754000
2756000
2758000
2760000
trpC
D082_27780
D082_27810
TU3205
TU3207
TU3209
TU3210
TU3211
TU3214
TU3215
TU3216
Untreated
15°C
42°C
−CO_2
Dark
−Fe
HL
−N
−P
Exp. P.
Stat. P.

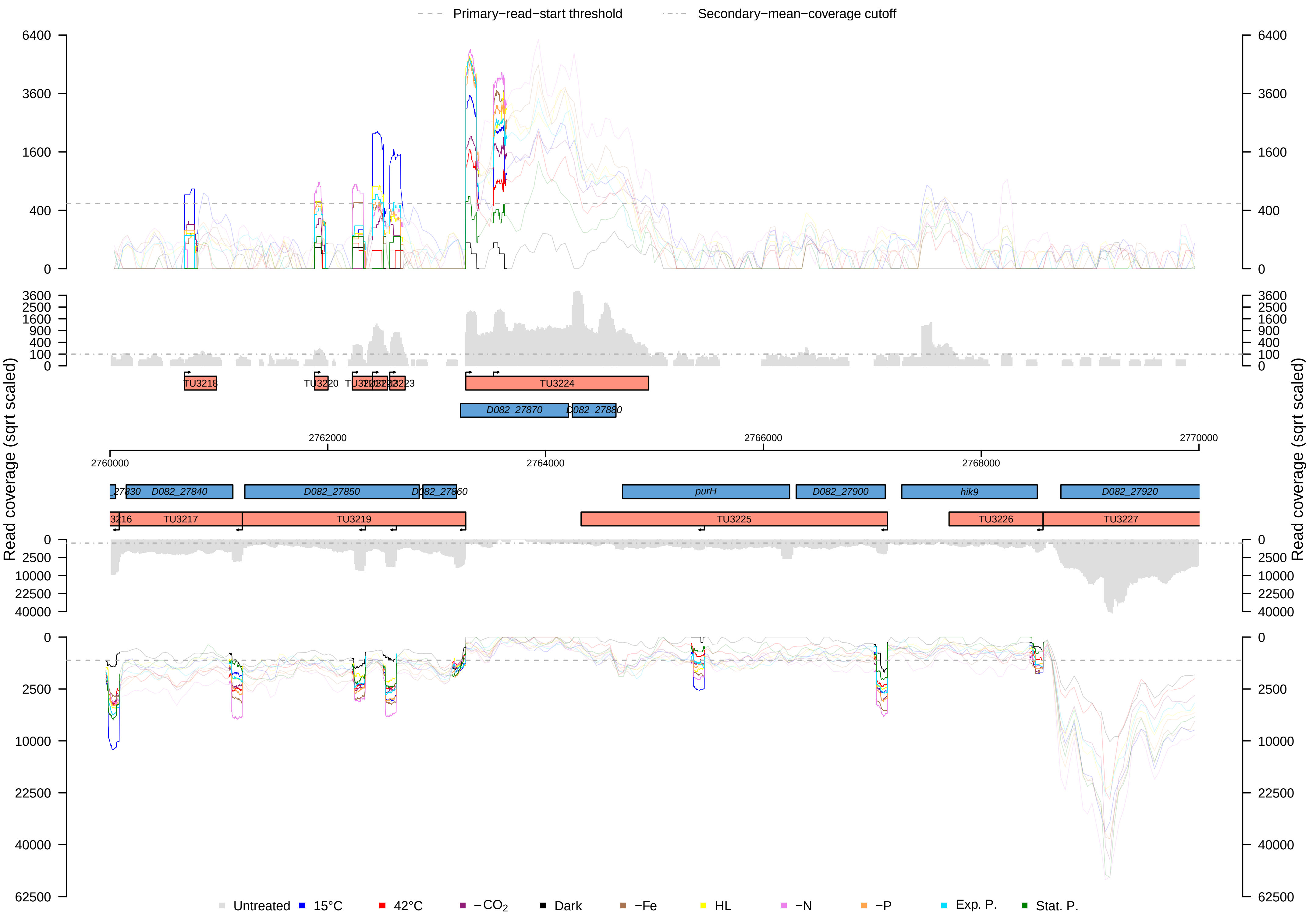

Primary−read−start threshold
Secondary−mean−coverage cutoff
Read coverage (sqrt scaled)
TU3218
TU3220
TU3224
D082_27870
D082_27880
2760000
2762000
2764000
2766000
2768000
2770000
D082_27840
D082_27850
D082_27860
purH
D082_27900
hik9
D082_27920
TU3217
TU3219
TU3225
TU3226
TU3227
Untreated
15°C
42°C
−CO2
Dark
−Fe
HL
−N
−P
Exp. P.
Stat. P.

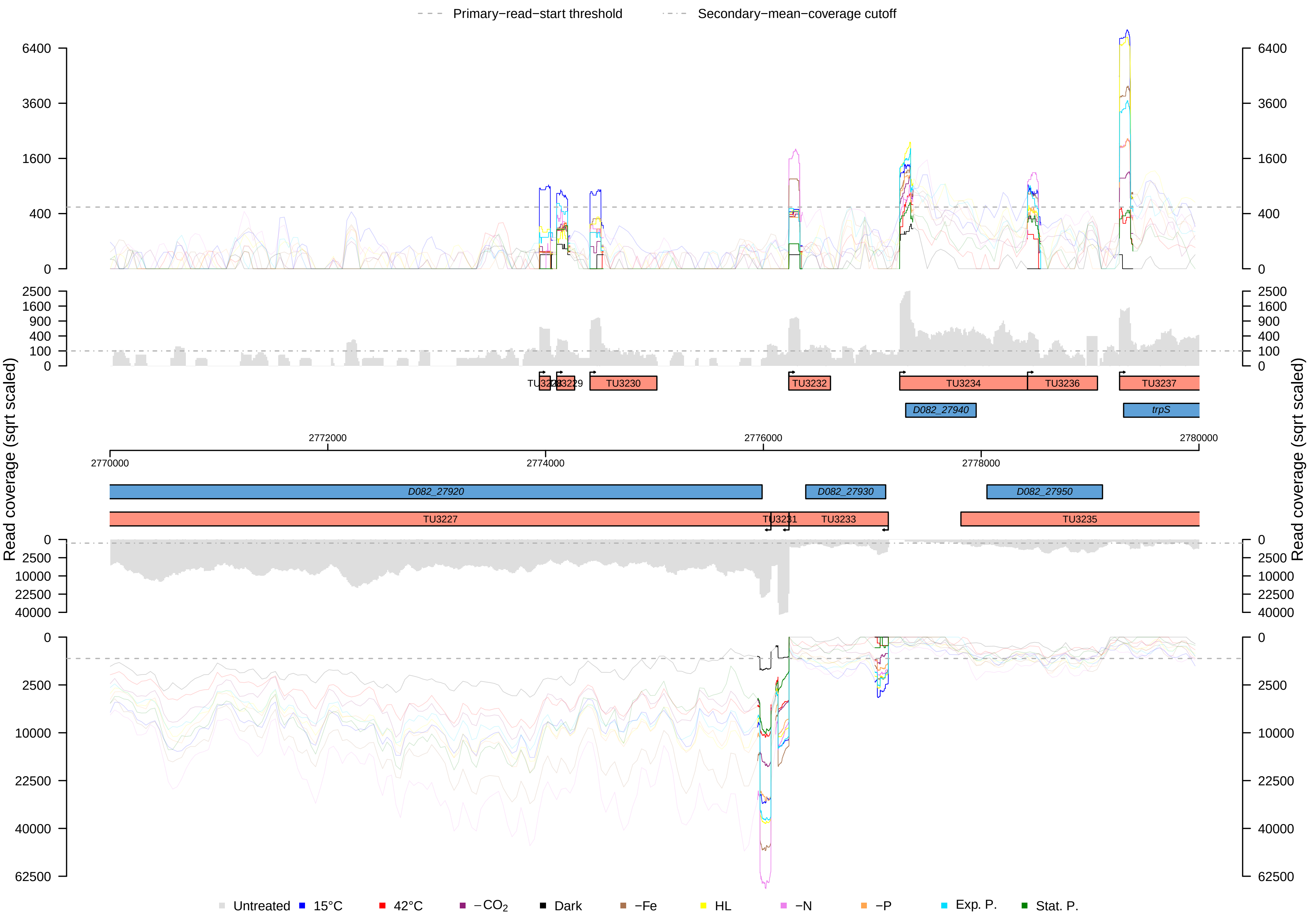

Primary−read−start threshold
Secondary−mean−coverage cutoff
Read coverage (sqrt scaled)
6400
3600
1600
400
0
2500
1600
900
400
100
0
TU3228
TU3229
TU3230
TU3232
TU3234
TU3236
TU3237
D082_27940
trpS
2770000
2772000
2774000
2776000
2778000
2780000
D082_27920
D082_27930
D082_27950
TU3227
TU3231
TU3233
TU3235
0
2500
10000
22500
40000
0
2500
10000
22500
40000
62500
Untreated
15°C
42°C
−CO2
Dark
−Fe
HL
−N
−P
Exp. P.
Stat. P.

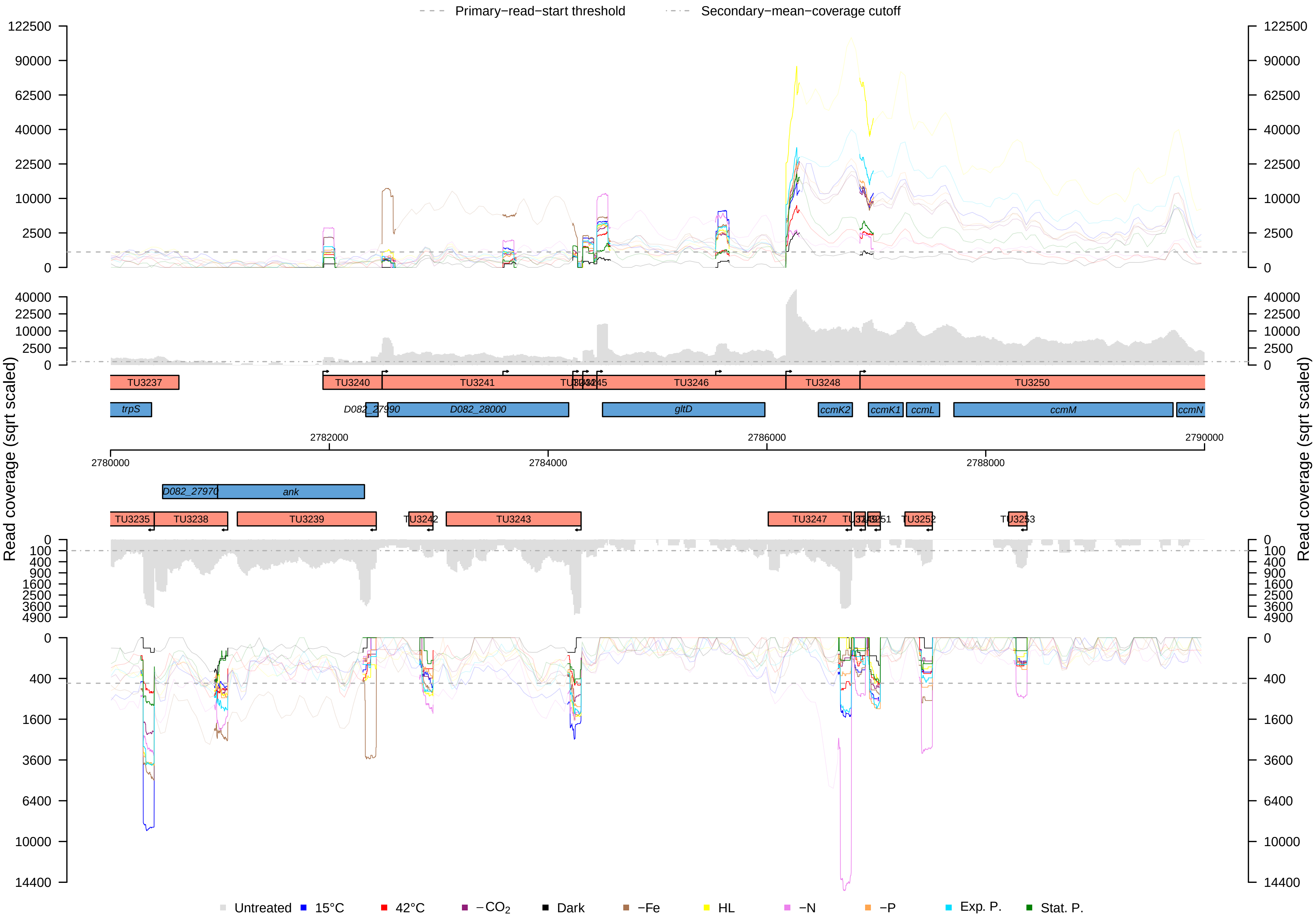

Primary−read−start threshold
Secondary−mean−coverage cutoff
Read coverage (sqrt scaled)
122500
90000
62500
40000
22500
10000
2500
0
TU3237
TU3240
TU3241
TU3246
TU3248
TU3250
trpS
D082_27990
D082_28000
gltD
ccmK2
ccmK1
ccmL
ccmM
ccmN
2780000
2782000
2784000
2786000
2788000
2790000
D082_27970
ank
TU3235
TU3238
TU3239
TU3242
TU3243
TU3247
TU3252
TU3253
Untreated
15°C
42°C
−CO2
Dark
−Fe
HL
−N
−P
Exp. P.
Stat. P.

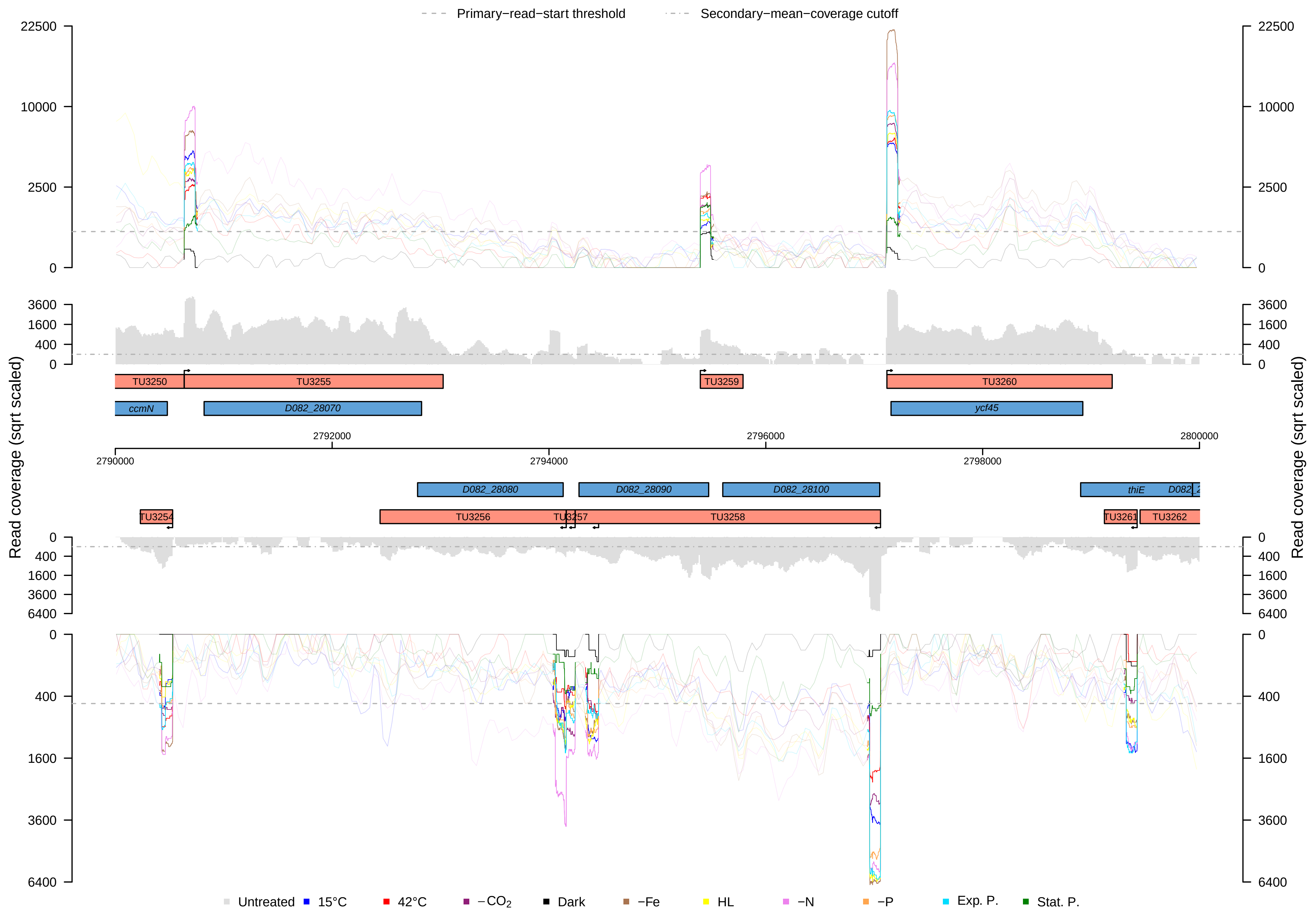

Primary−read−start threshold
Secondary−mean−coverage cutoff
Read coverage (sqrt scaled)
22500
10000
2500
0
3600
1600
400
6400
TU3250
TU3255
TU3259
TU3260
ccmN
D082_28070
ycf45
2790000
2792000
2794000
2796000
2798000
2800000
D082_28080
D082_28090
D082_28100
thiE
TU3254
TU3256
TU3257
TU3258
TU3261
TU3262
Untreated
15°C
42°C
−CO2
Dark
−Fe
HL
−N
−P
Exp. P.
Stat. P.

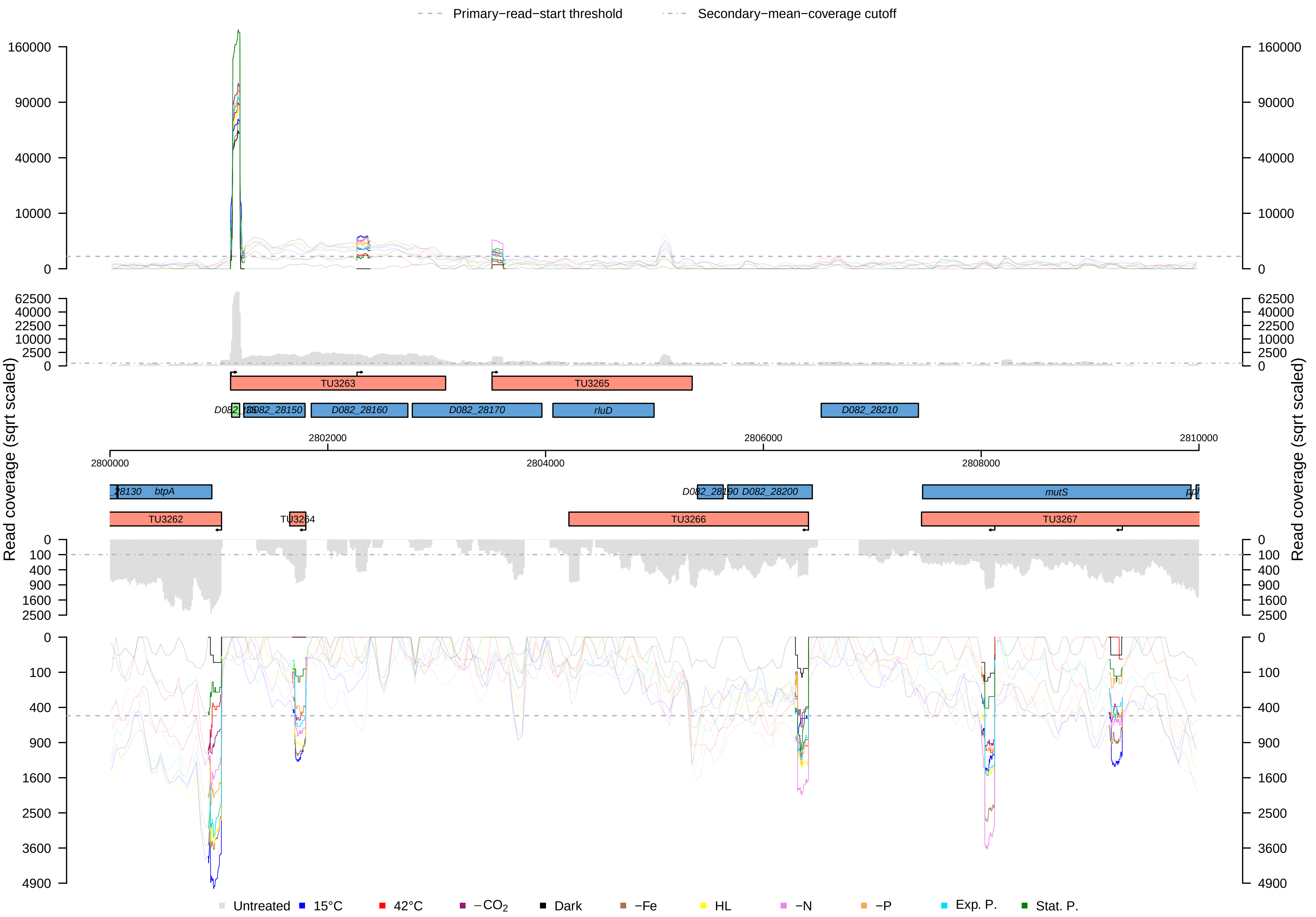

Primary−read−start threshold
Secondary−mean−coverage cutoff
160000
90000
40000
10000
0
62500
40000
22500
10000
2500
0
TU3263
TU3265
D082_28150
D082_28160
D082_28170
rluD
D082_28210
2800000
2802000
2804000
2806000
2808000
2810000
btpA
D082_28200
mutS
TU3262
TU3266
TU3267
100
400
900
1600
2500
3600
4900
Read coverage (sqrt scaled)
Untreated
15°C
42°C
−CO2
Dark
−Fe
HL
−N
−P
Exp. P.
Stat. P.

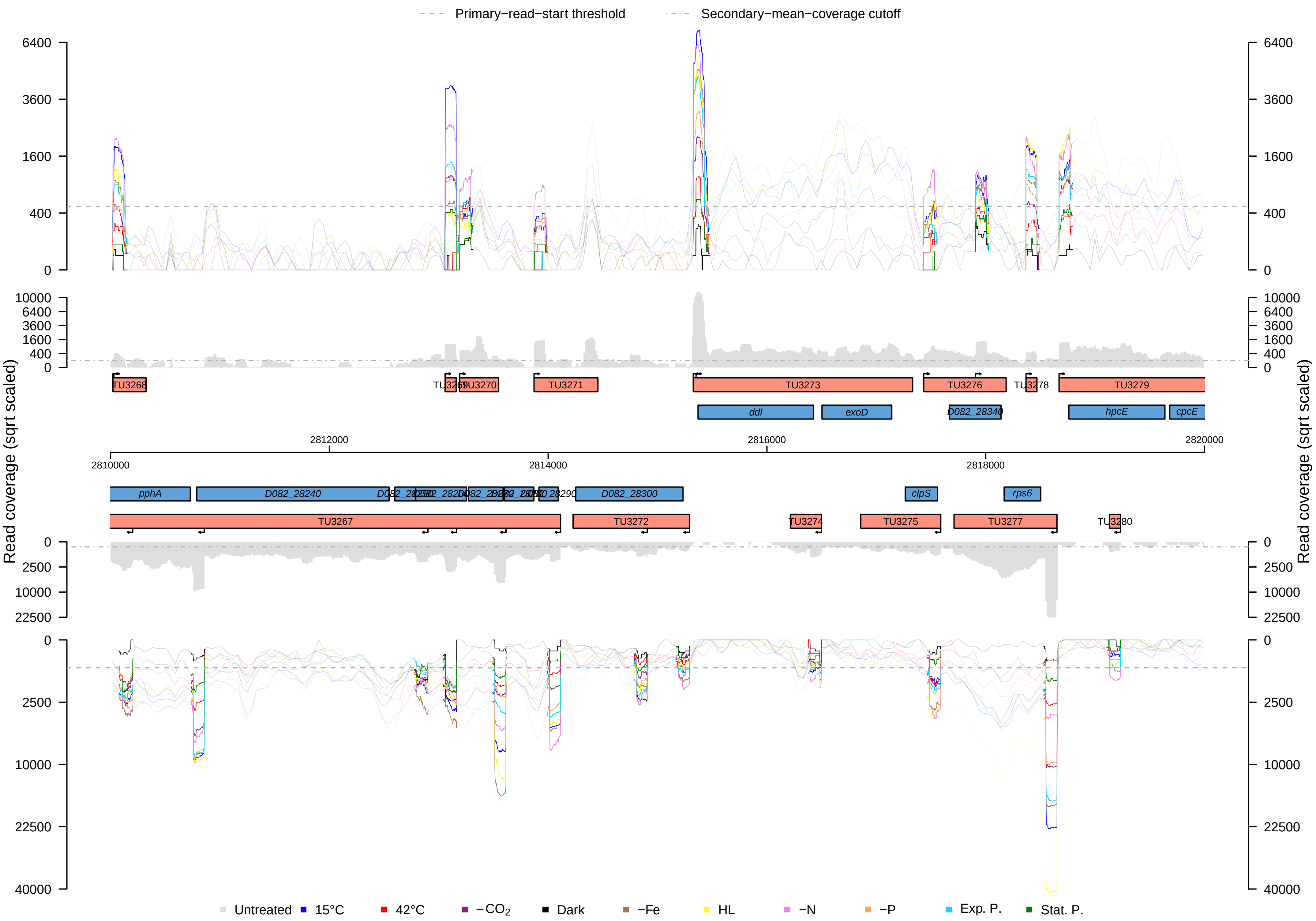

Primary−read−start threshold
Secondary−mean−coverage cutoff
Read coverage (sqrt scaled)
6400
3600
1600
400
0
10000
2500
22500
40000
TU3268
TU3269
TU3270
TU3271
TU3273
TU3276
TU3278
TU3279
ddl
exoD
D082_28340
hpcE
cpcE
2810000
2812000
2814000
2816000
2818000
2820000
pphA
D082_28240
D082_28300
clpS
rps6
TU3267
TU3272
TU3274
TU3275
TU3277
TU3280
Untreated
15°C
42°C
−CO2
Dark
−Fe
HL
−N
−P
Exp. P.
Stat. P.

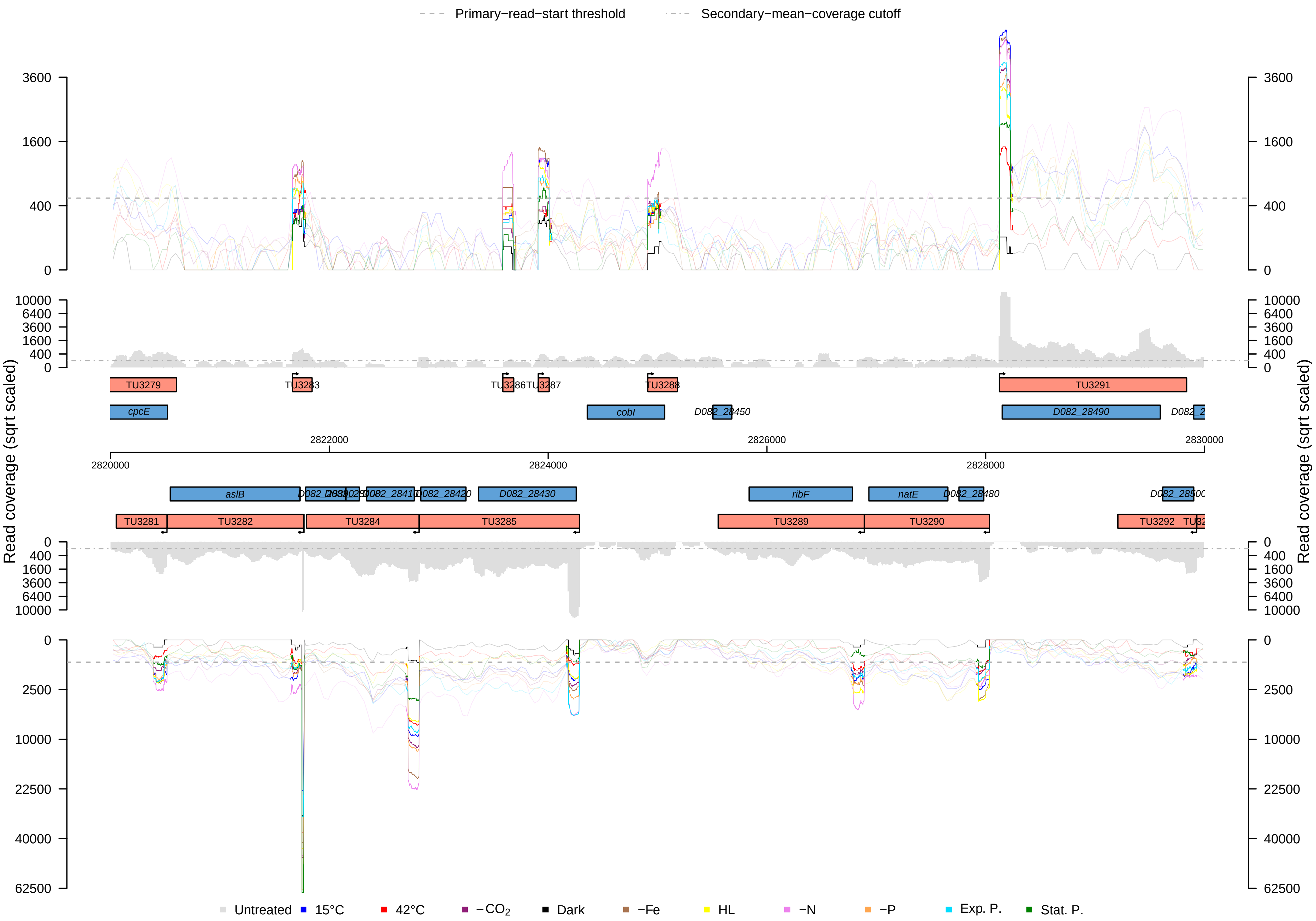

Primary−read−start threshold
Secondary−mean−coverage cutoff
Read coverage (sqrt scaled)
3600
1600
400
0
10000
6400
3600
1600
400
0
TU3279
TU3283
TU3286
TU3287
TU3288
TU3291
cpcE
cobI
D082_28450
D082_28490
2820000
2822000
2824000
2826000
2828000
2830000
aslB
D082_28410
D082_28420
D082_28430
ribF
natE
D082_28480
TU3281
TU3282
TU3284
TU3285
TU3289
TU3290
TU3292
2500
10000
22500
40000
62500
Untreated
15°C
42°C
−CO2
Dark
−Fe
HL
−N
−P
Exp. P.
Stat. P.

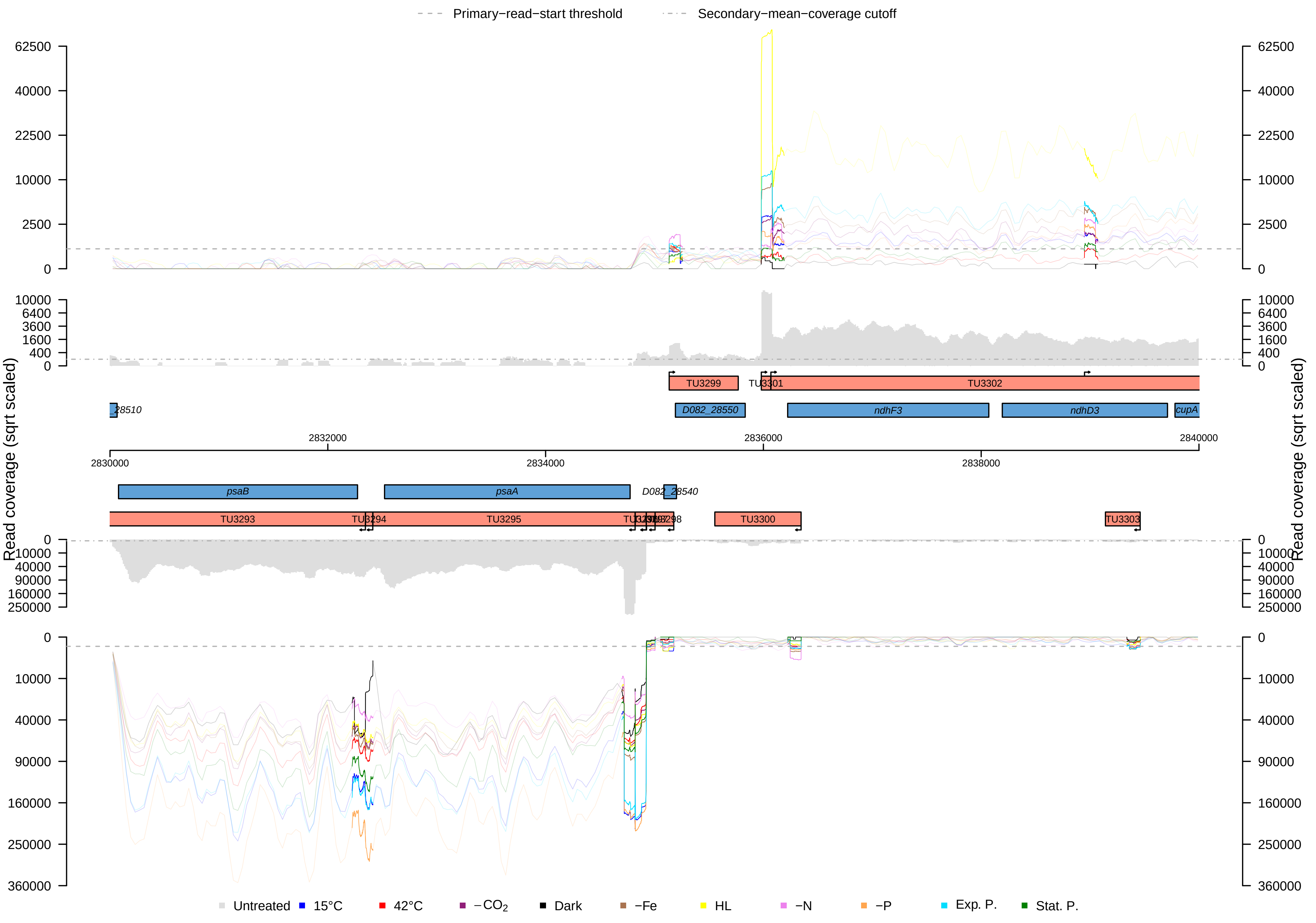

Primary−read−start threshold
Secondary−mean−coverage cutoff
Read coverage (sqrt scaled)
62500
40000
22500
10000
2500
0
6400
3600
1600
400
90000
160000
250000
360000
TU3299
TU3301
TU3302
D082_28550
ndhF3
ndhD3
cupA
_28510
2830000
2832000
2834000
2836000
2838000
2840000
psaB
psaA
D082_28540
TU3293
TU3294
TU3295
TU3300
TU3303
Untreated
15°C
42°C
−CO2
Dark
−Fe
HL
−N
−P
Exp. P.
Stat. P.

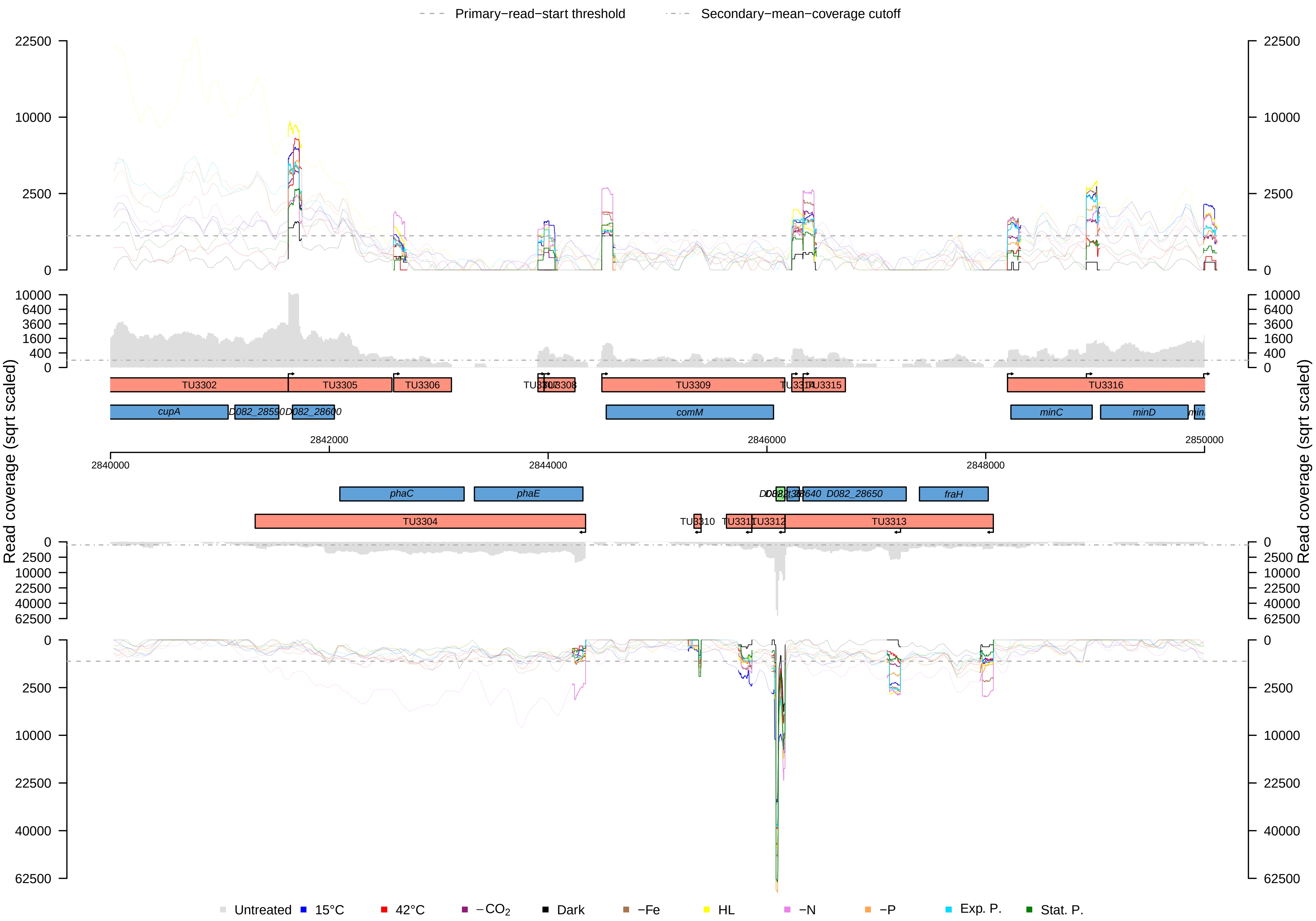

Primary−read−start threshold
Secondary−mean−coverage cutoff
Read coverage (sqrt scaled)
TU3302
TU3305
TU3306
TU3308
TU3309
TU3315
TU3316
cupA
comM
minC
minD
phaC
phaE
D082_28650
fraH
TU3304
TU3312
TU3313
2840000
2842000
2844000
2846000
2848000
2850000
Untreated
15°C
42°C
−CO2
Dark
−Fe
HL
−N
−P
Exp. P.
Stat. P.

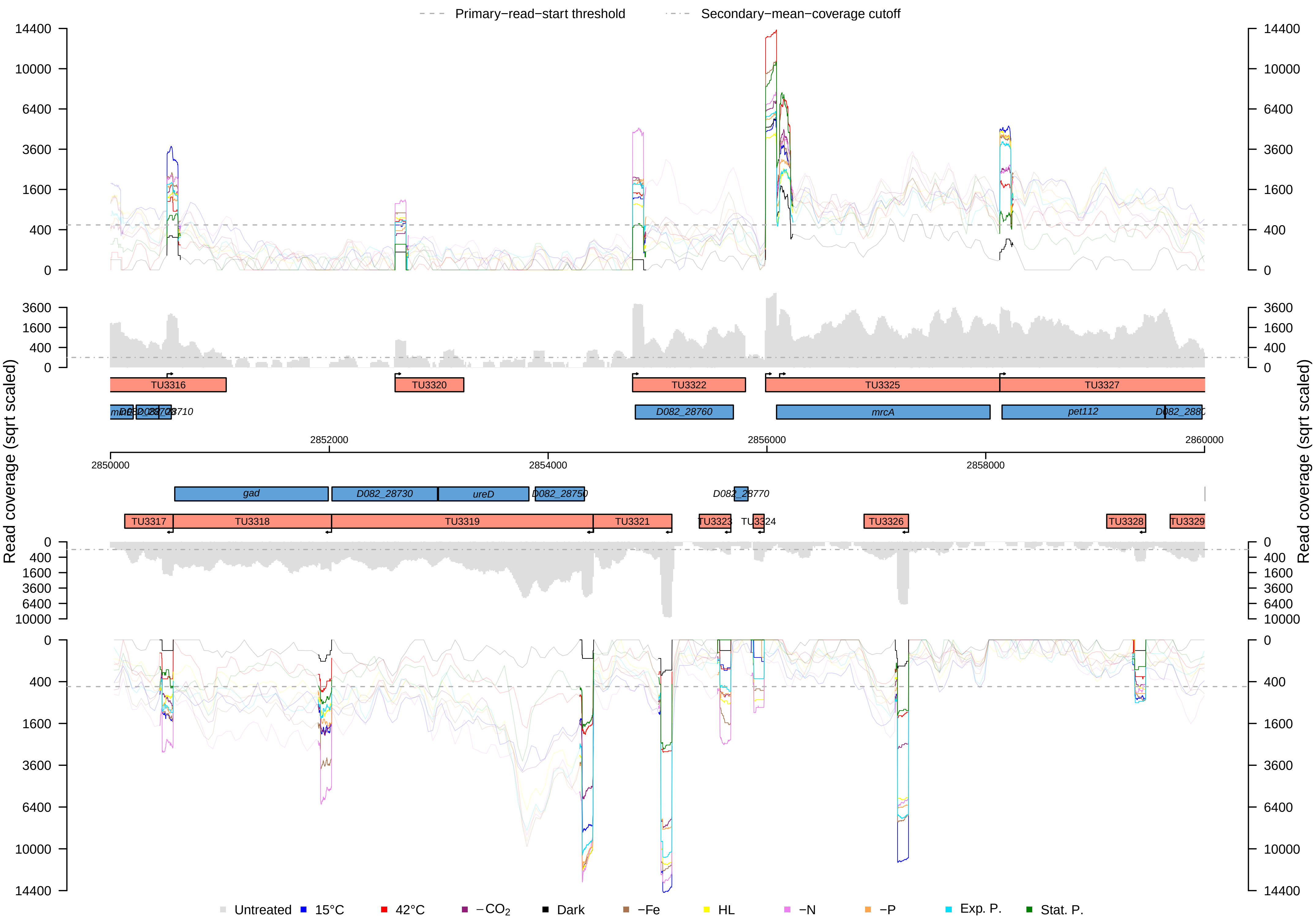

Primary−read−start threshold
Secondary−mean−coverage cutoff
Read coverage (sqrt scaled)
14400
10000
6400
3600
1600
400
0
TU3316
TU3320
TU3322
TU3325
TU3327
D082_28760
mrcA
pet112
2850000
2852000
2854000
2856000
2858000
2860000
gad
D082_28730
ureD
D082_28750
D082_28770
TU3317
TU3318
TU3319
TU3321
TU3323
TU3324
TU3326
TU3328
TU3329
Untreated
15°C
42°C
−CO2
Dark
−Fe
HL
−N
−P
Exp. P.
Stat. P.

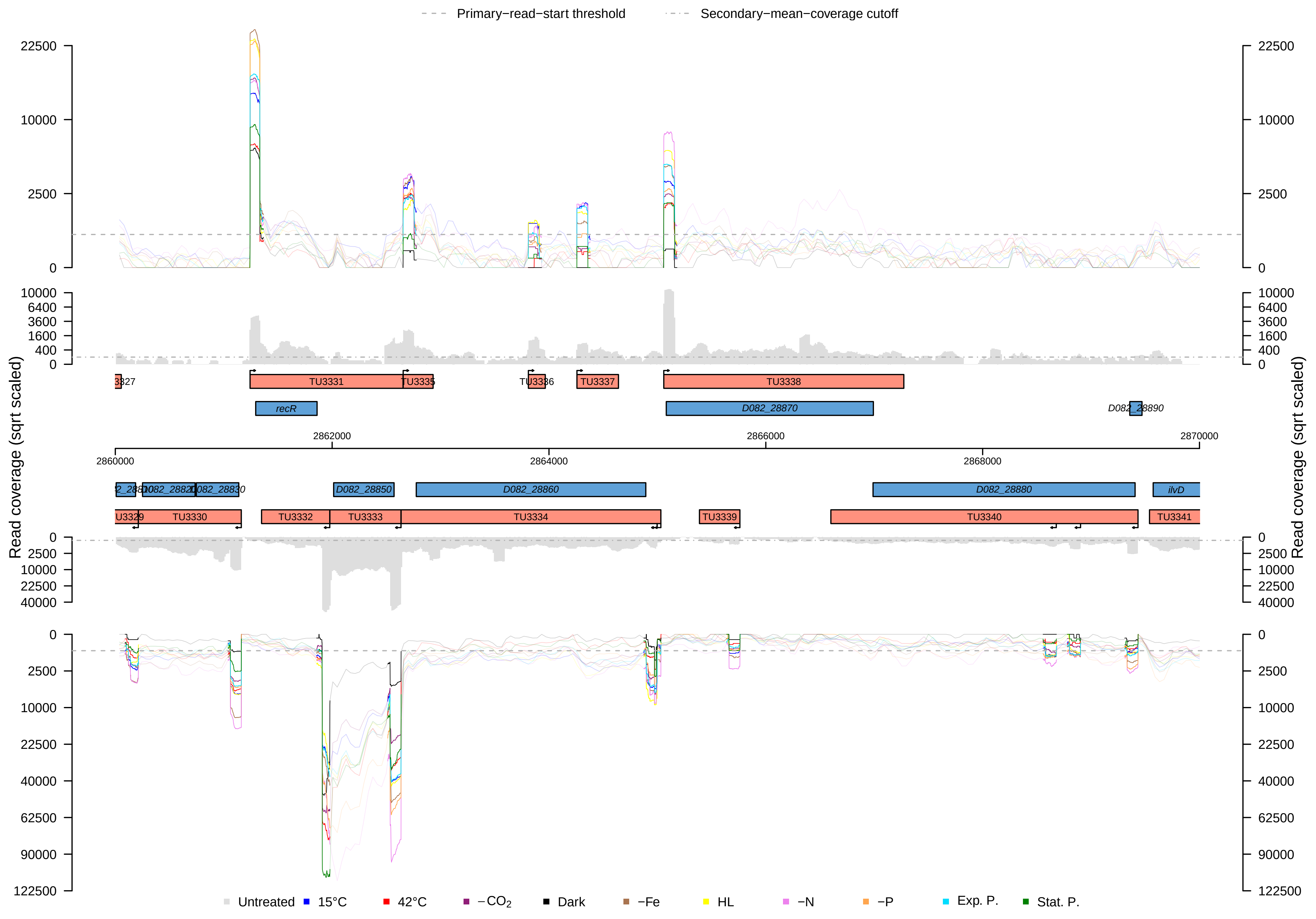

Primary−read−start threshold
Secondary−mean−coverage cutoff
Read coverage (sqrt scaled)
22500
10000
2500
0
10000
6400
3600
1600
400
TU3331
TU3335
TU3336
TU3337
TU3338
recR
D082_28870
D082_28890
2860000
2862000
2864000
2866000
2868000
2870000
D082_28850
D082_28860
D082_28880
ilvD
TU3330
TU3332
TU3333
TU3334
TU3339
TU3340
TU3341
40000
62500
90000
122500
Untreated
15°C
42°C
−CO2
Dark
−Fe
HL
−N
−P
Exp. P.
Stat. P.

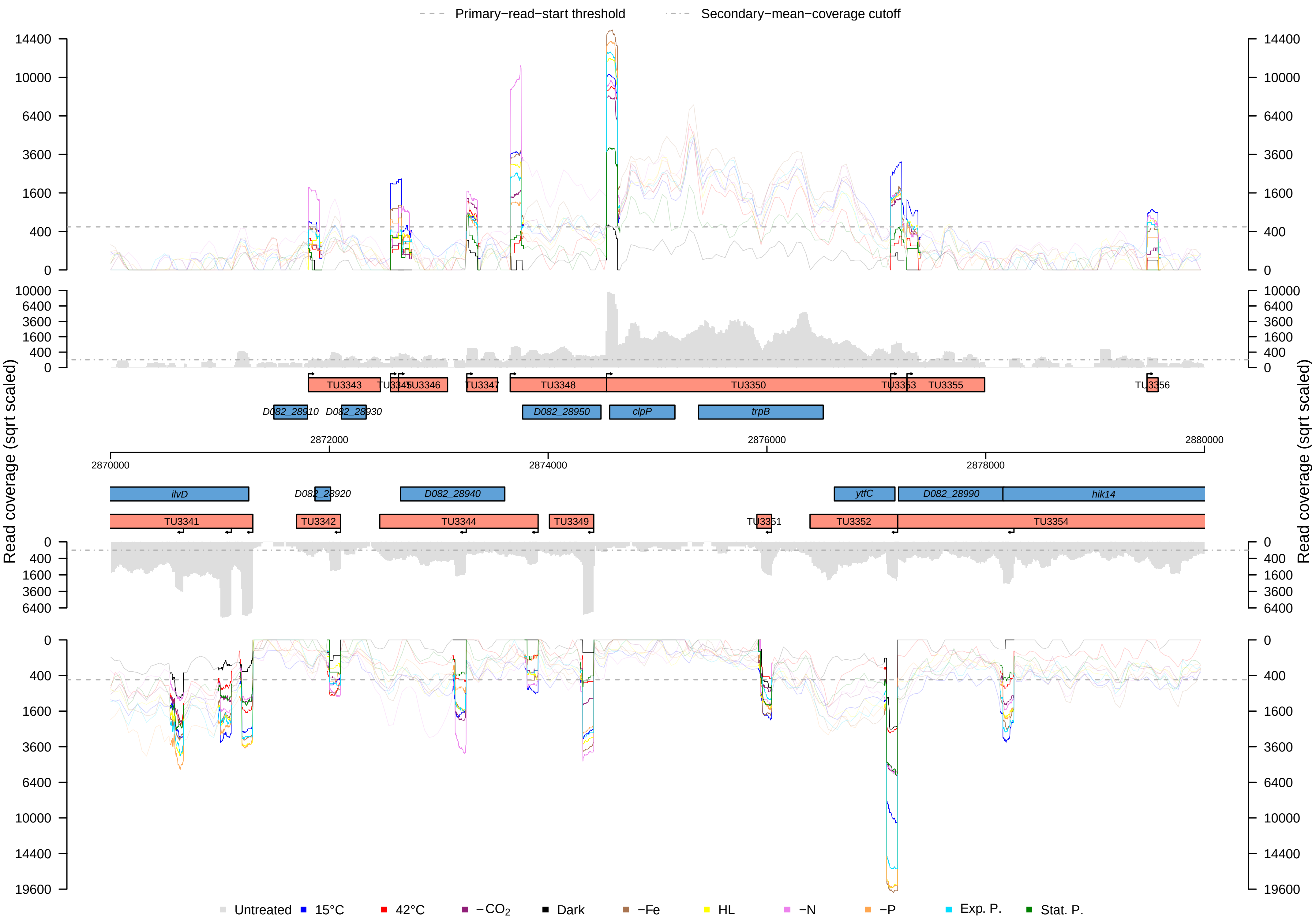

Primary−read−start threshold
Secondary−mean−coverage cutoff
Read coverage (sqrt scaled)
14400
10000
6400
3600
1600
400
0
TU3343
TU3345
TU3346
TU3347
TU3348
TU3350
TU3353
TU3355
TU3356
D082_28910
D082_28930
D082_28950
clpP
trpB
2870000
2872000
2874000
2876000
2878000
2880000
ilvD
D082_28920
D082_28940
ytfC
D082_28990
hik14
TU3341
TU3342
TU3344
TU3349
TU3351
TU3352
TU3354
19600
Untreated
15°C
42°C
−CO2
Dark
−Fe
HL
−N
−P
Exp. P.
Stat. P.

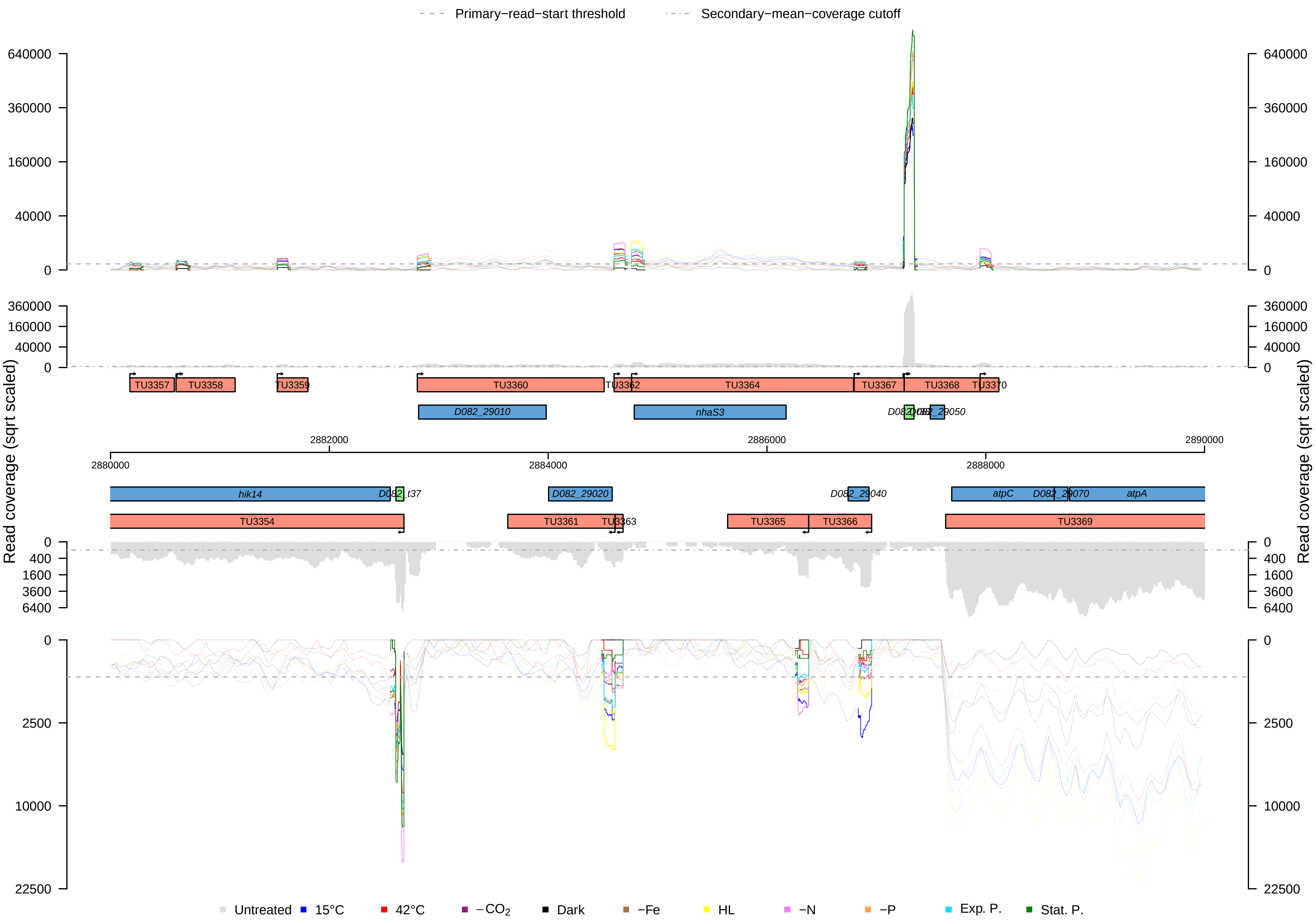

Primary−read−start threshold
Secondary−mean−coverage cutoff
Read coverage (sqrt scaled)
640000
360000
160000
40000
0
TU3357
TU3358
TU3359
TU3360
TU3362
TU3364
TU3367
TU3368
TU3370
D082_29010
nhaS3
D082_29050
2880000
2882000
2884000
2886000
2888000
2890000
hik14
D082_t37
D082_29020
D082_29040
atpC
D082_29070
atpA
TU3354
TU3361
TU3363
TU3365
TU3366
TU3369
400
1600
3600
6400
2500
10000
22500
Untreated
15°C
42°C
−CO2
Dark
−Fe
HL
−N
−P
Exp. P.
Stat. P.

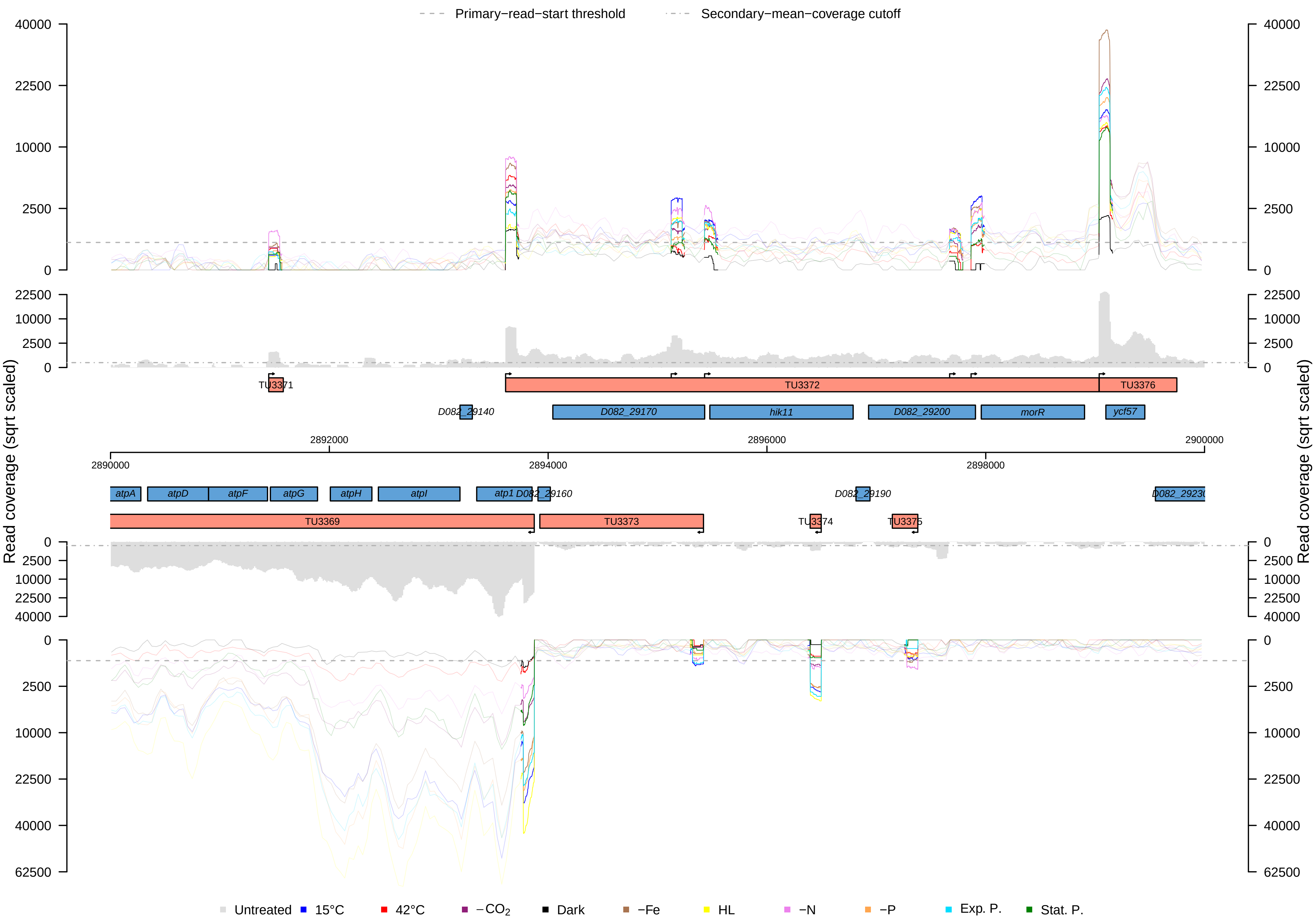

Primary−read−start threshold
Secondary−mean−coverage cutoff
Read coverage (sqrt scaled)
40000
22500
10000
2500
0
62500
TU3371
TU3372
TU3376
D082_29140
D082_29170
hik11
D082_29200
morR
ycf57
2890000
2892000
2894000
2896000
2898000
2900000
atpA
atpD
atpF
atpG
atpH
atpI
atp1
D082_29160
D082_29190
D082_29230
TU3369
TU3373
TU3374
TU3375
Untreated
15°C
42°C
−CO2
Dark
−Fe
HL
−N
−P
Exp. P.
Stat. P.

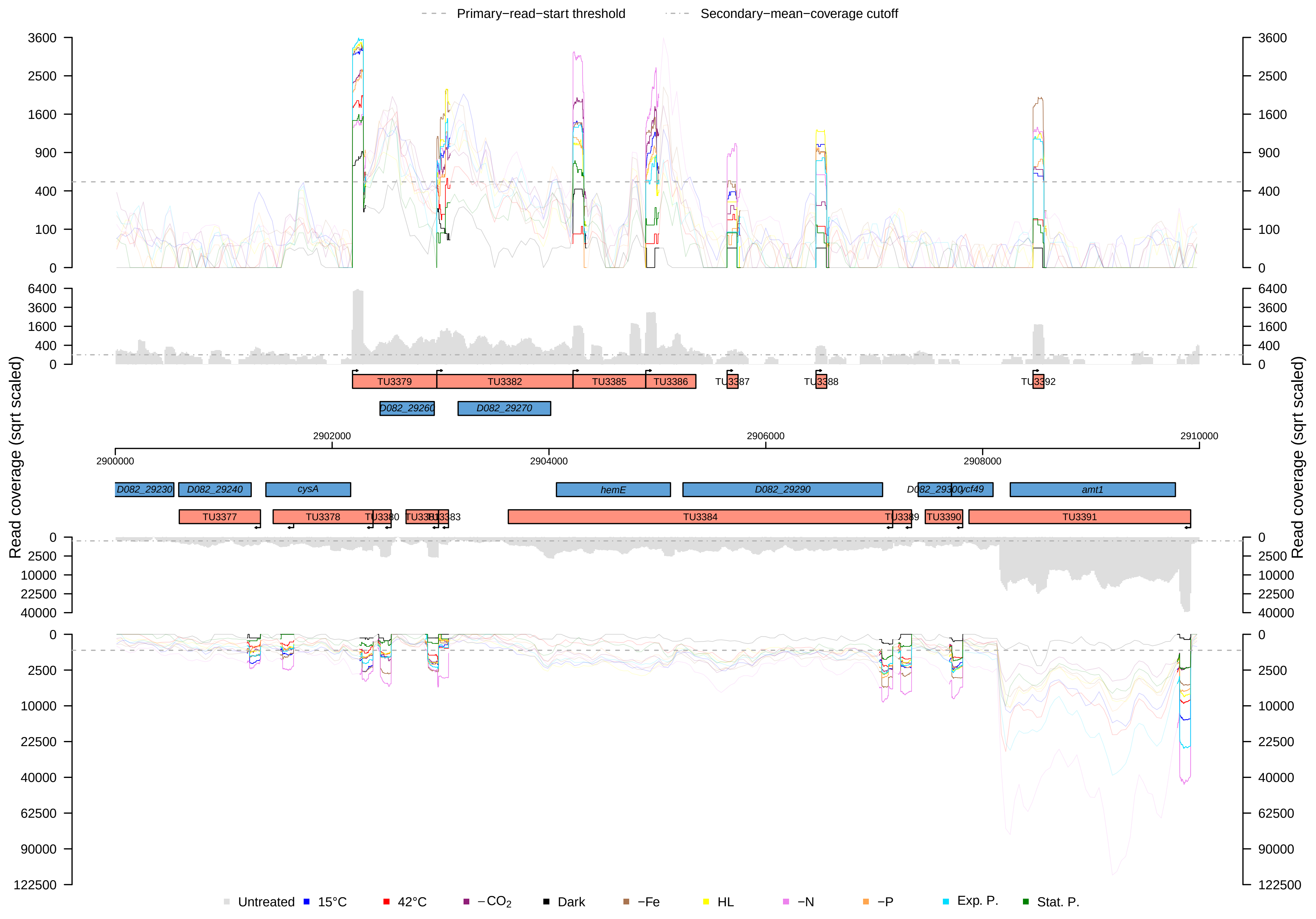

Primary−read−start threshold
Secondary−mean−coverage cutoff
Read coverage (sqrt scaled)
3600
2500
1600
900
400
100
0
6400
TU3379
TU3382
TU3385
TU3386
TU3387
TU3388
TU3392
D082_29260
D082_29270
2900000
2902000
2904000
2906000
2908000
2910000
D082_29230
D082_29240
cysA
hemE
D082_29290
D082_29300
ycf49
amt1
TU3377
TU3378
TU3380
TU3381
TU3383
TU3384
TU3389
TU3390
TU3391
10000
22500
40000
62500
90000
122500
Untreated
15°C
42°C
$-CO_2$
Dark
−Fe
HL
−N
−P
Exp. P.
Stat. P.

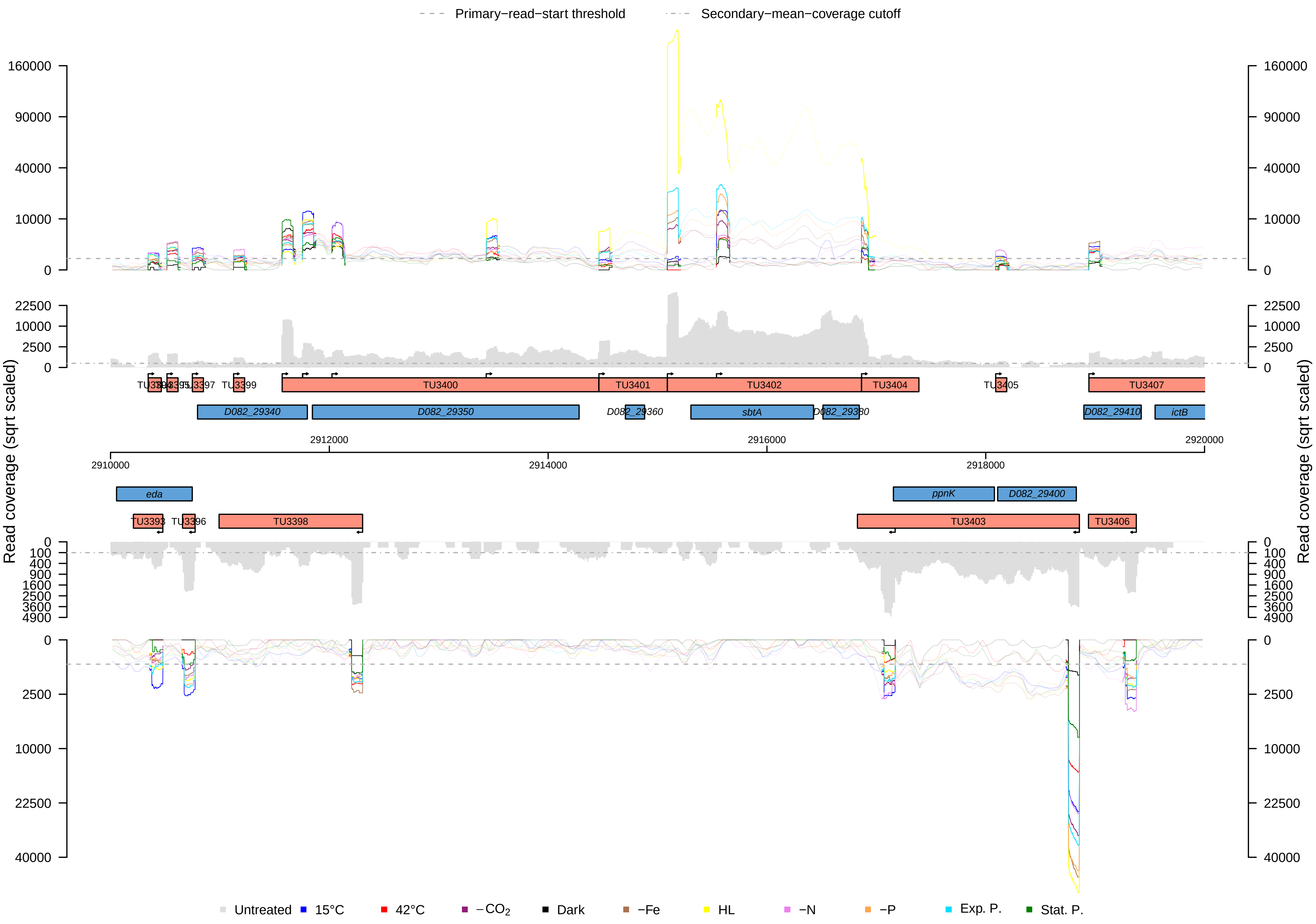

Primary−read−start threshold
Secondary−mean−coverage cutoff
Read coverage (sqrt scaled)
TU3400
TU3401
TU3402
TU3404
TU3407
D082_29340
D082_29350
D082_29360
sbtA
D082_29380
D082_29410
ictB
2910000
2912000
2914000
2916000
2918000
2920000
eda
ppnK
D082_29400
TU3398
TU3403
TU3406
Untreated
15°C
42°C
−CO2
Dark
−Fe
HL
−N
−P
Exp. P.
Stat. P.

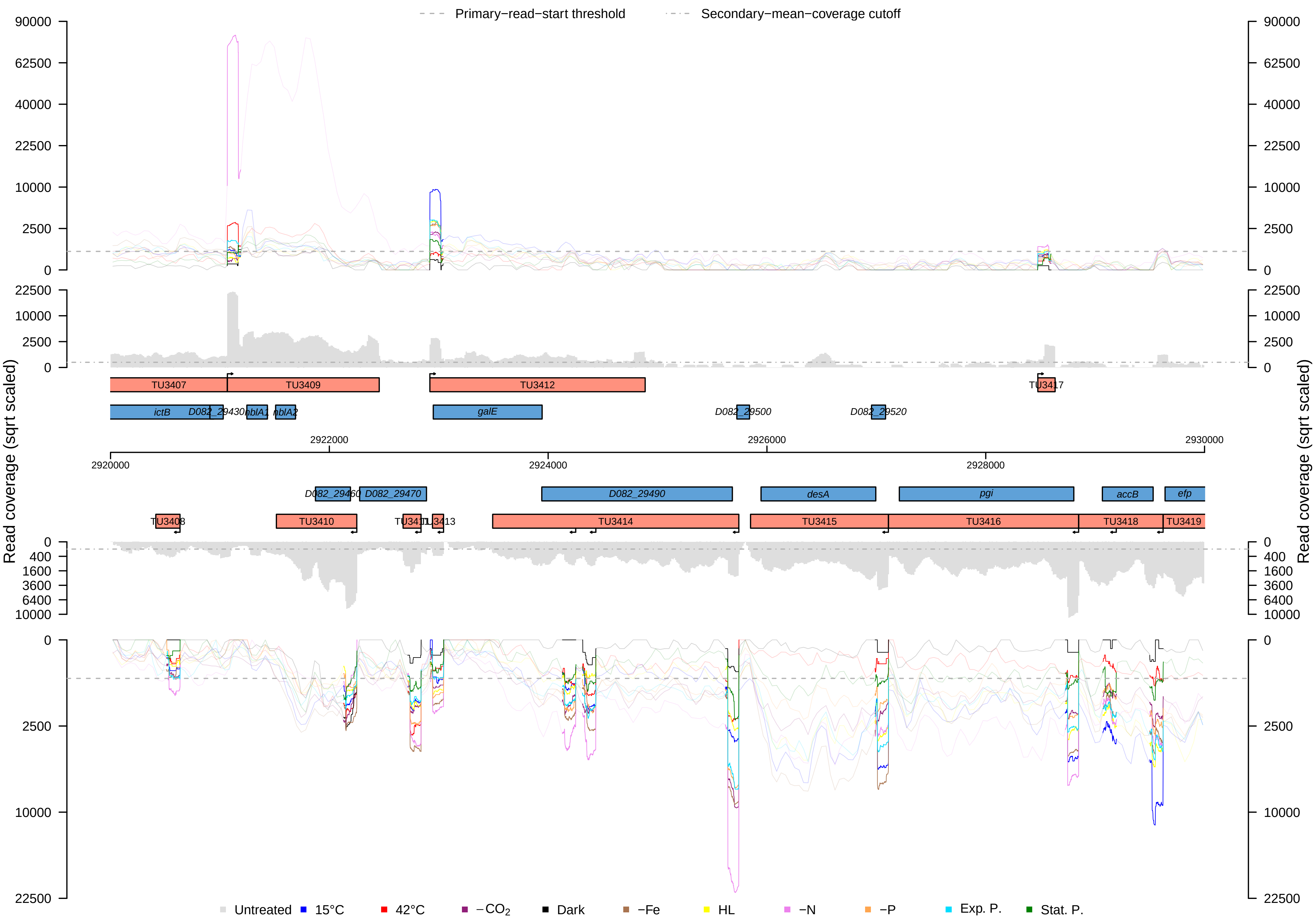

Primary−read−start threshold
Secondary−mean−coverage cutoff
Read coverage (sqrt scaled)
TU3407
TU3409
TU3412
TU3417
ictB
D082_29430
nblA1
nblA2
galE
D082_29500
D082_29520
2920000
2922000
2924000
2926000
2928000
2930000
D082_29460
D082_29470
D082_29490
desA
pgi
accB
efp
TU3408
TU3410
TU3411
TU3413
TU3414
TU3415
TU3416
TU3418
TU3419
Untreated
15°C
42°C
−CO2
Dark
−Fe
HL
−N
−P
Exp. P.
Stat. P.

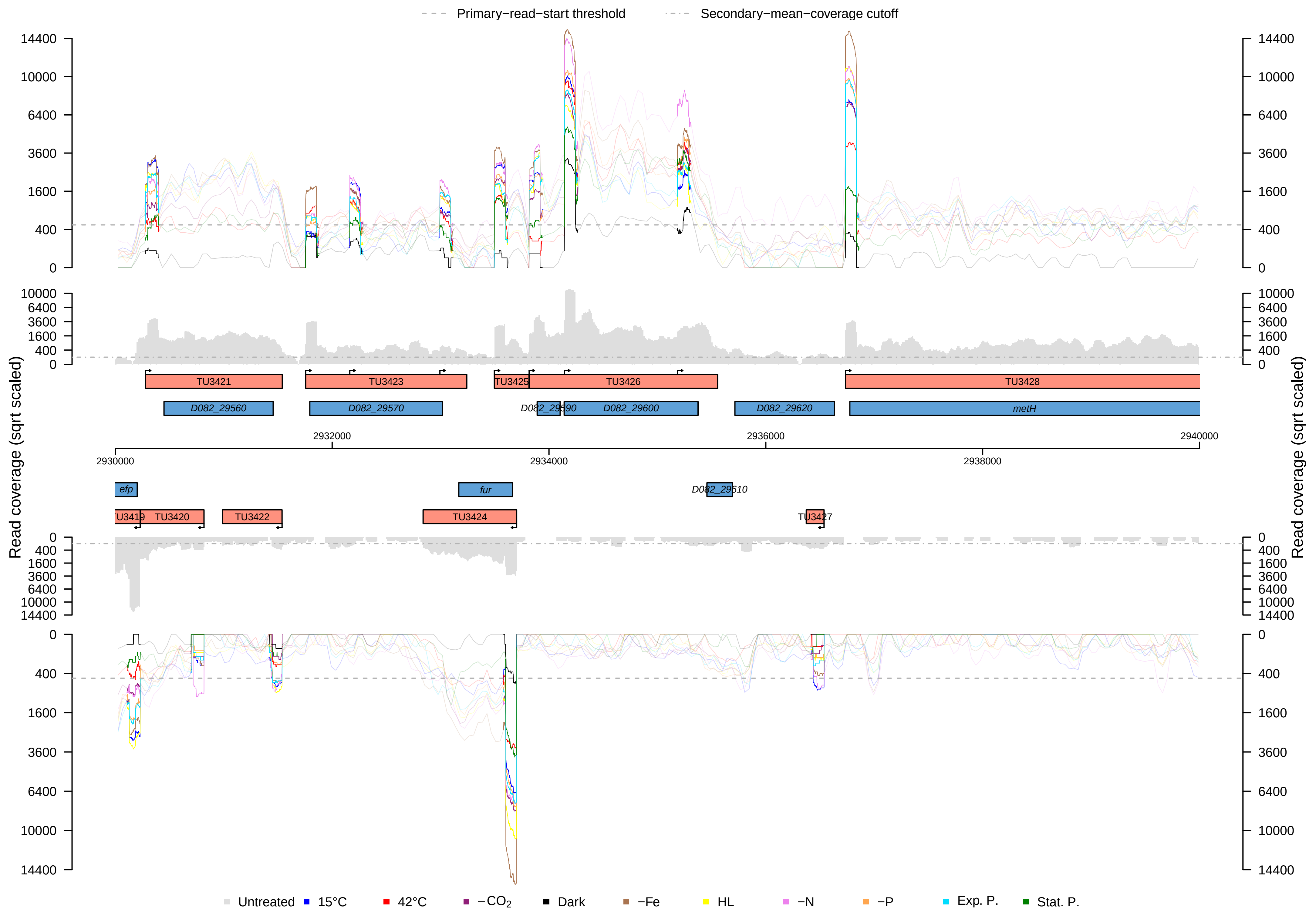

Primary−read−start threshold
Secondary−mean−coverage cutoff
Read coverage (sqrt scaled)
TU3421
TU3423
TU3425
TU3426
TU3428
D082_29560
D082_29570
D082_29590
D082_29600
D082_29620
metH
2930000
2932000
2934000
2936000
2938000
2940000
efp
fur
D082_29610
TU3419
TU3420
TU3422
TU3424
TU3427
Untreated
15°C
42°C
−CO2
Dark
−Fe
HL
−N
−P
Exp. P.
Stat. P.

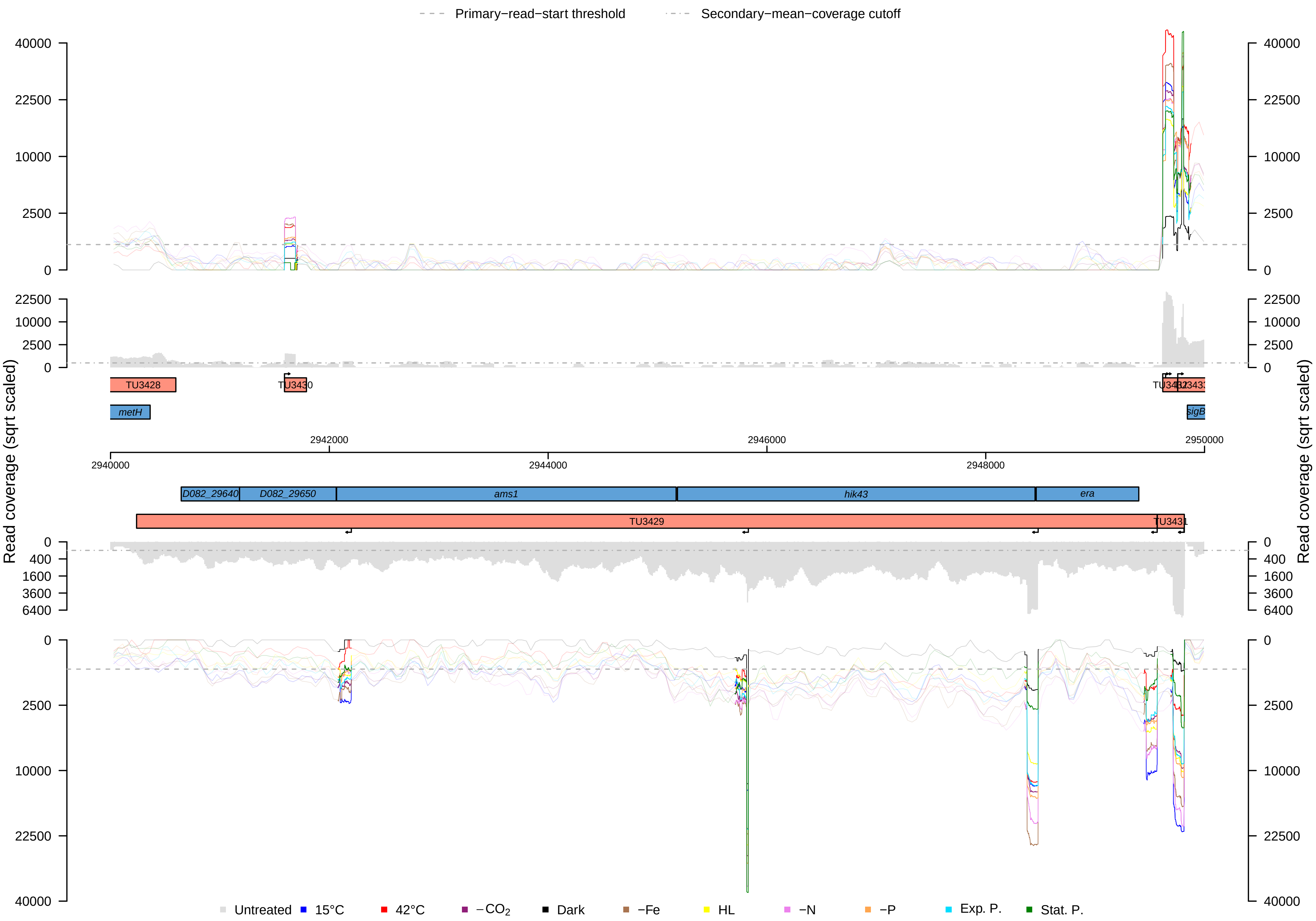

Primary−read−start threshold
Secondary−mean−coverage cutoff
Read coverage (sqrt scaled)
40000
22500
10000
2500
0
400
1600
3600
6400
TU3428
TU3430
metH
sigB
2940000
2942000
2944000
2946000
2948000
2950000
D082_29640
D082_29650
ams1
hik43
era
TU3429
TU3431
Untreated
15°C
42°C
−CO2
Dark
−Fe
HL
−N
−P
Exp. P.
Stat. P.

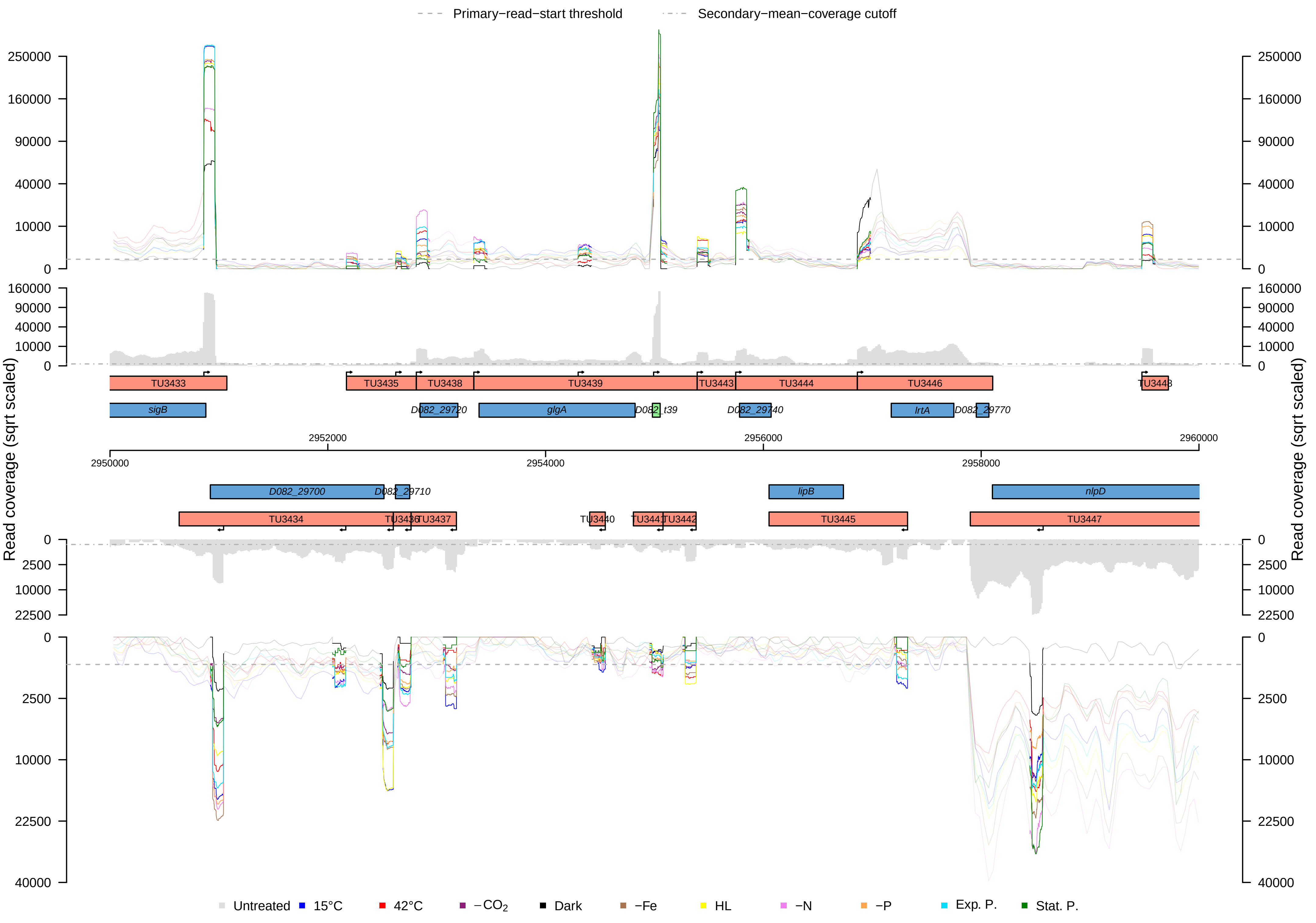

Primary−read−start threshold
Secondary−mean−coverage cutoff
Read coverage (sqrt scaled)
250000
160000
90000
40000
10000
0
22500
2500
TU3433
TU3435
TU3438
TU3439
TU3443
TU3444
TU3446
TU3448
sigB
D082_29720
glgA
D082_t39
D082_29740
lrtA
D082_29770
2950000
2952000
2954000
2956000
2958000
2960000
D082_29700
D082_29710
lipB
nlpD
TU3434
TU3436
TU3437
TU3440
TU3441
TU3442
TU3445
TU3447
Untreated
15°C
42°C
−CO2
Dark
−Fe
HL
−N
−P
Exp. P.
Stat. P.

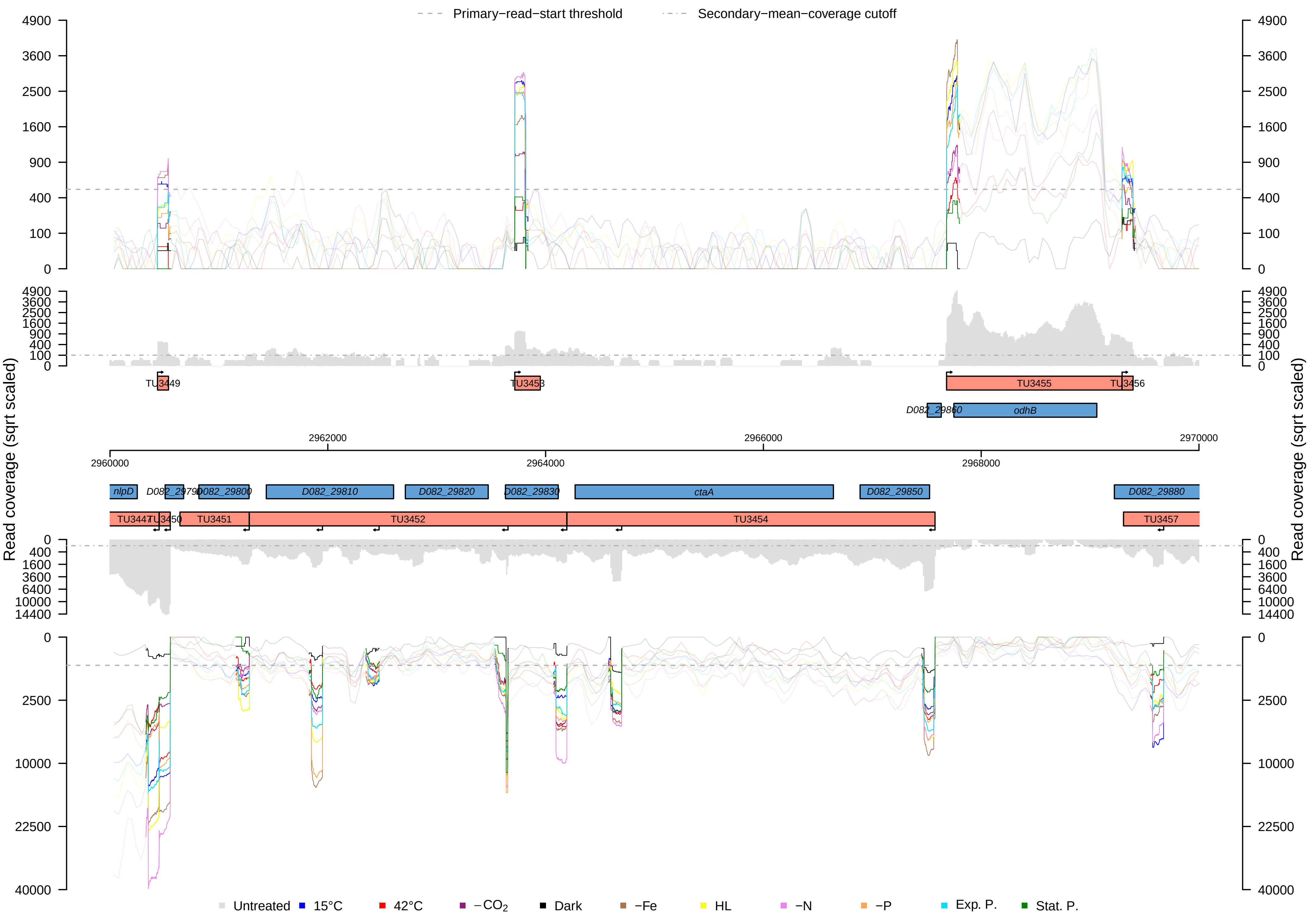

Primary−read−start threshold
Secondary−mean−coverage cutoff
Read coverage (sqrt scaled)
4900
3600
2500
1600
900
400
100
0
TU3449
TU3453
TU3455
TU3456
D082_29860
odhB
2962000
2966000
2970000
2960000
2964000
2968000
nlpD
D082_29790
D082_29800
D082_29810
D082_29820
D082_29830
ctaA
D082_29850
D082_29880
TU3447
TU3450
TU3451
TU3452
TU3454
TU3457
6400
10000
14400
22500
40000
Untreated
15°C
42°C
−CO2
Dark
−Fe
HL
−N
−P
Exp. P.
Stat. P.

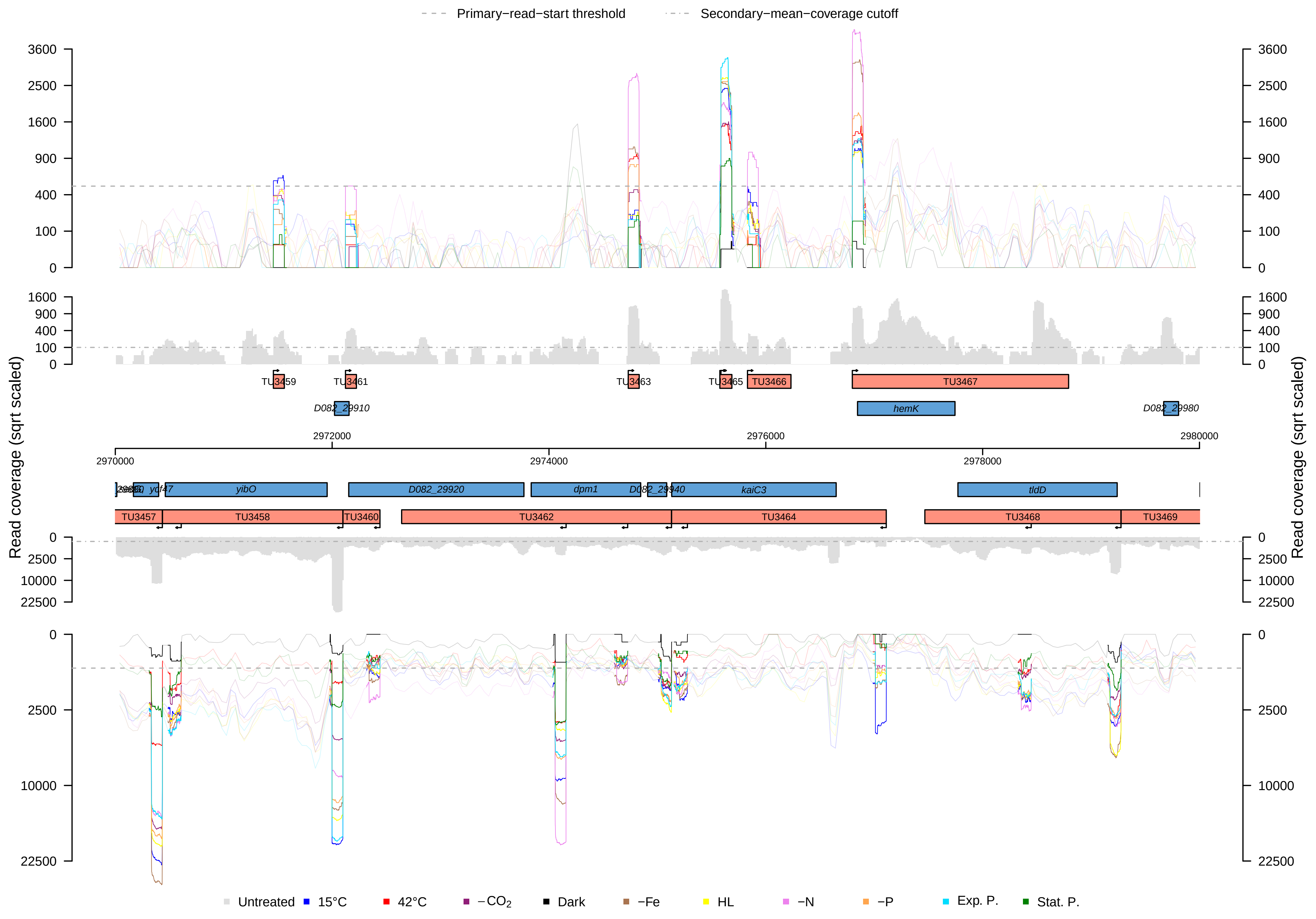

Primary−read−start threshold
Secondary−mean−coverage cutoff
Read coverage (sqrt scaled)
0
100
400
900
1600
2500
3600
2500
10000
22500
TU3457
TU3458
TU3459
TU3460
TU3461
TU3462
TU3463
TU3464
TU3465
TU3466
TU3467
TU3468
TU3469
D082_29910
hemK
D082_29980
ycf47
yibO
D082_29920
dpm1
kaiC3
tldD
2970000
2972000
2974000
2976000
2978000
2980000
Untreated
15°C
42°C
−CO2
Dark
−Fe
HL
−N
−P
Exp. P.
Stat. P.

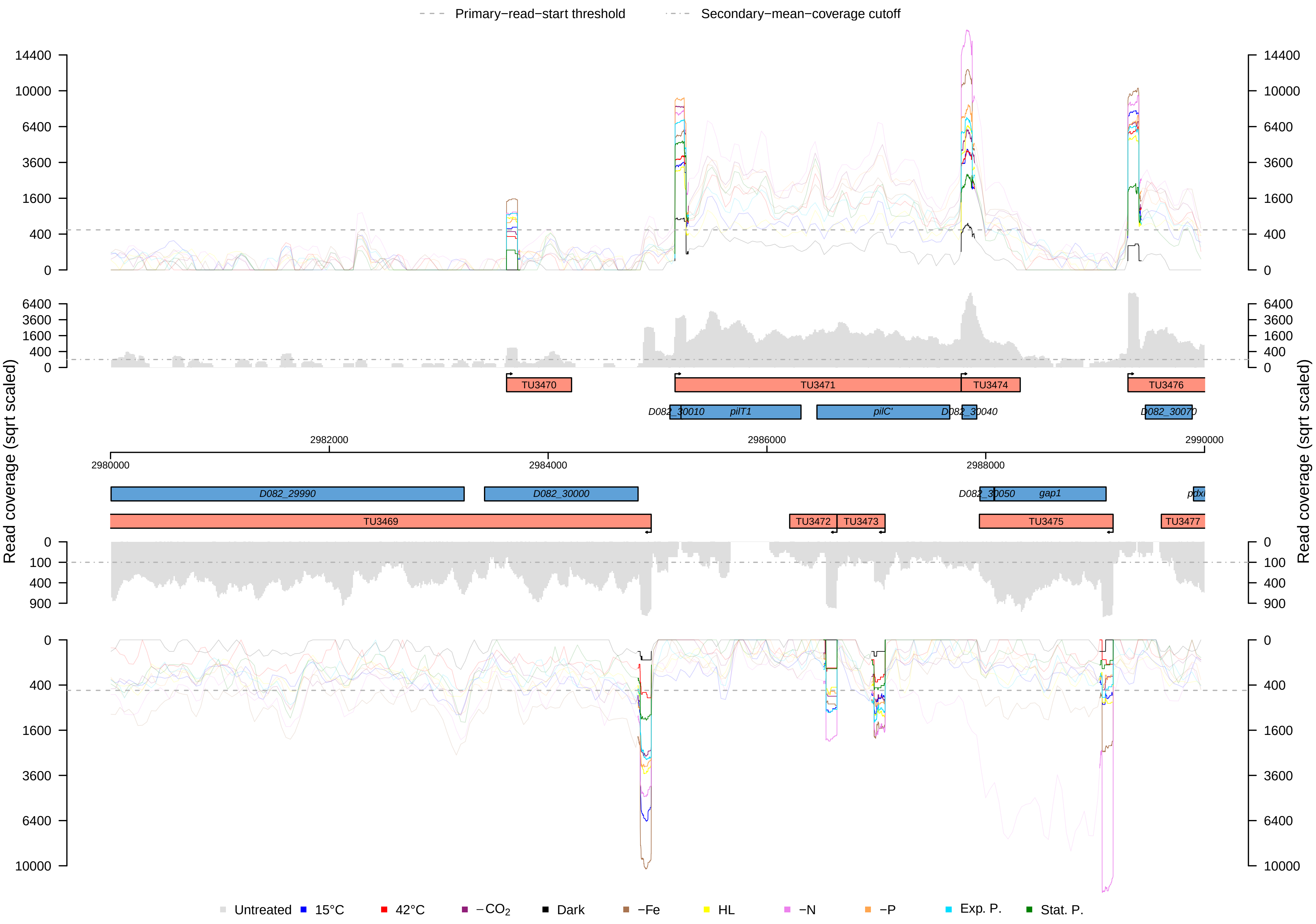

Primary−read−start threshold
Secondary−mean−coverage cutoff
Read coverage (sqrt scaled)
14400
10000
6400
3600
1600
400
0
TU3470
TU3471
TU3474
TU3476
D082_30010
pilT1
pilC'
D082_30040
D082_30070
2980000
2982000
2984000
2986000
2988000
2990000
D082_29990
D082_30000
D082_30050
gap1
pdxI
TU3469
TU3472
TU3473
TU3475
TU3477
100
900
Untreated
15°C
42°C
−CO_2
Dark
−Fe
HL
−N
−P
Exp. P.
Stat. P.

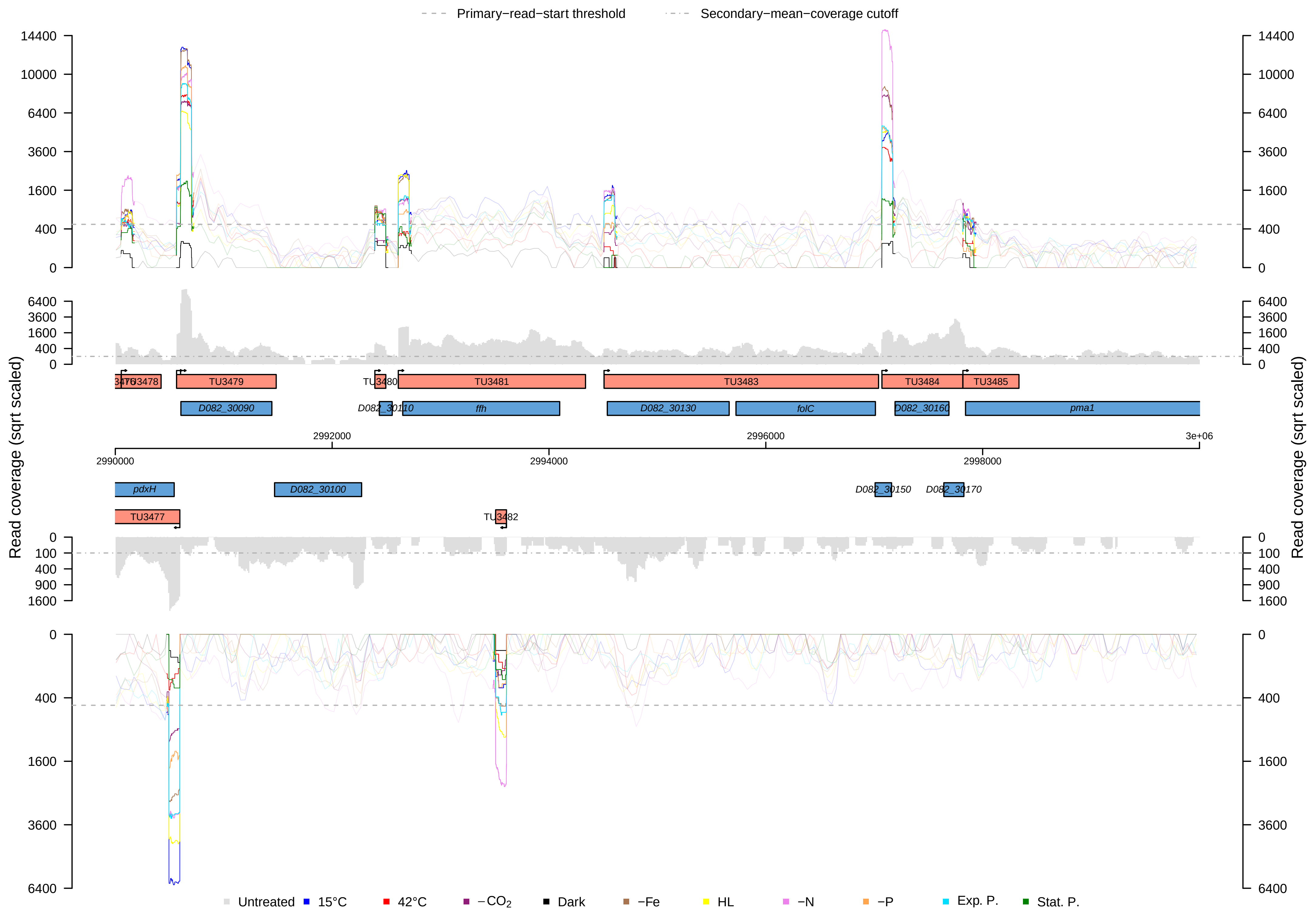

Primary−read−start threshold
Secondary−mean−coverage cutoff
Read coverage (sqrt scaled)
14400
10000
6400
3600
1600
400
0
TU3478
TU3479
TU3480
TU3481
TU3483
TU3484
TU3485
D082_30090
D082_30110
ffh
D082_30130
folC
D082_30160
pma1
2990000
2992000
2994000
2996000
2998000
3e+06
pdxH
D082_30100
D082_30150
D082_30170
TU3477
TU3482
Untreated
15°C
42°C
−CO2
Dark
−Fe
HL
−N
−P
Exp. P.
Stat. P.

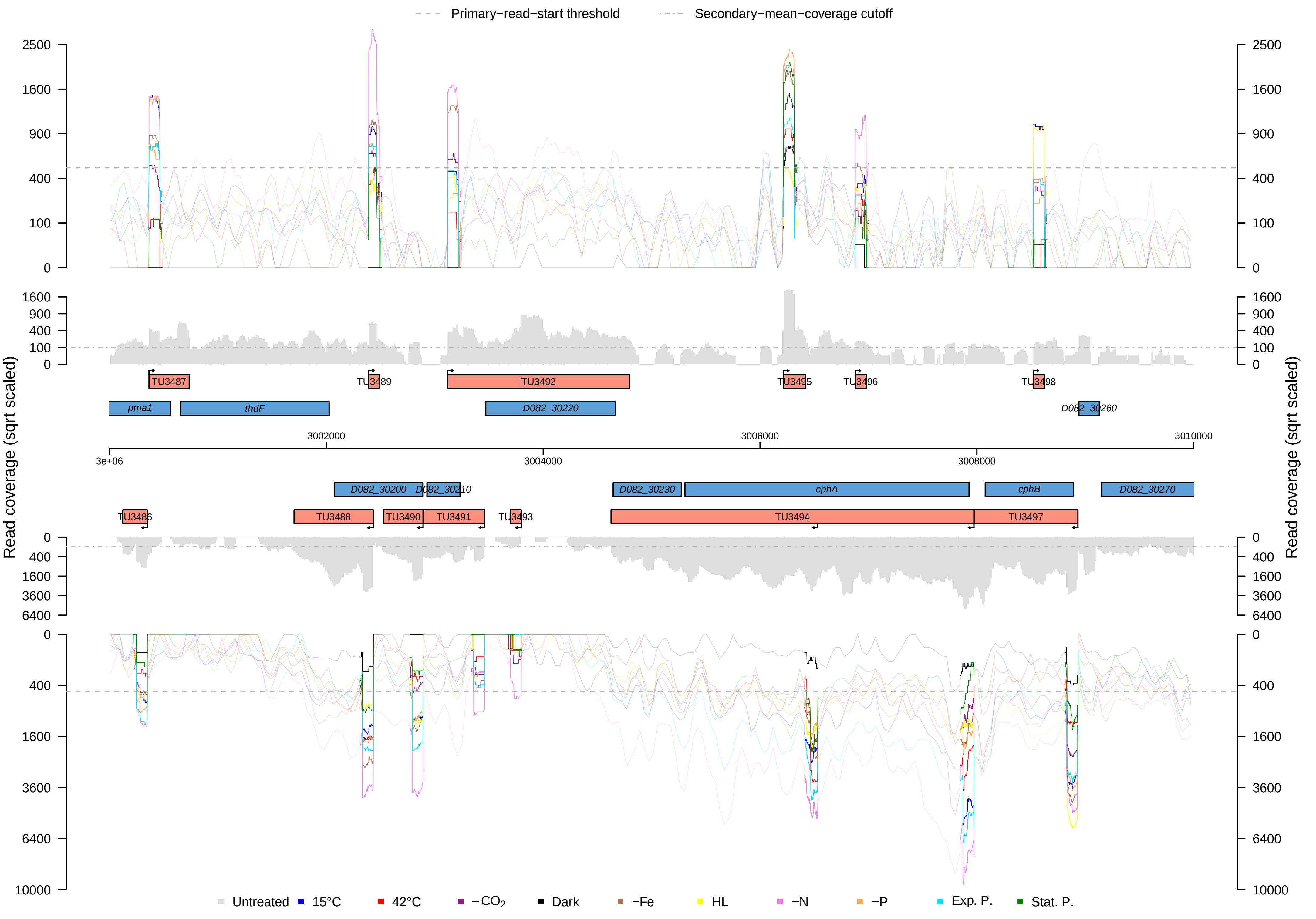

Primary−read−start threshold
Secondary−mean−coverage cutoff
Read coverage (sqrt scaled)
2500
1600
900
400
100
0
TU3487
TU3489
TU3492
TU3495
TU3496
TU3498
pma1
thdF
D082_30220
D082_30260
3e+06
3002000
3004000
3006000
3008000
3010000
D082_30200
D082_30210
D082_30230
cphA
cphB
D082_30270
TU3486
TU3488
TU3490
TU3491
TU3493
TU3494
TU3497
3600
6400
10000
Untreated
15°C
42°C
−CO2
Dark
−Fe
HL
−N
−P
Exp. P.
Stat. P.

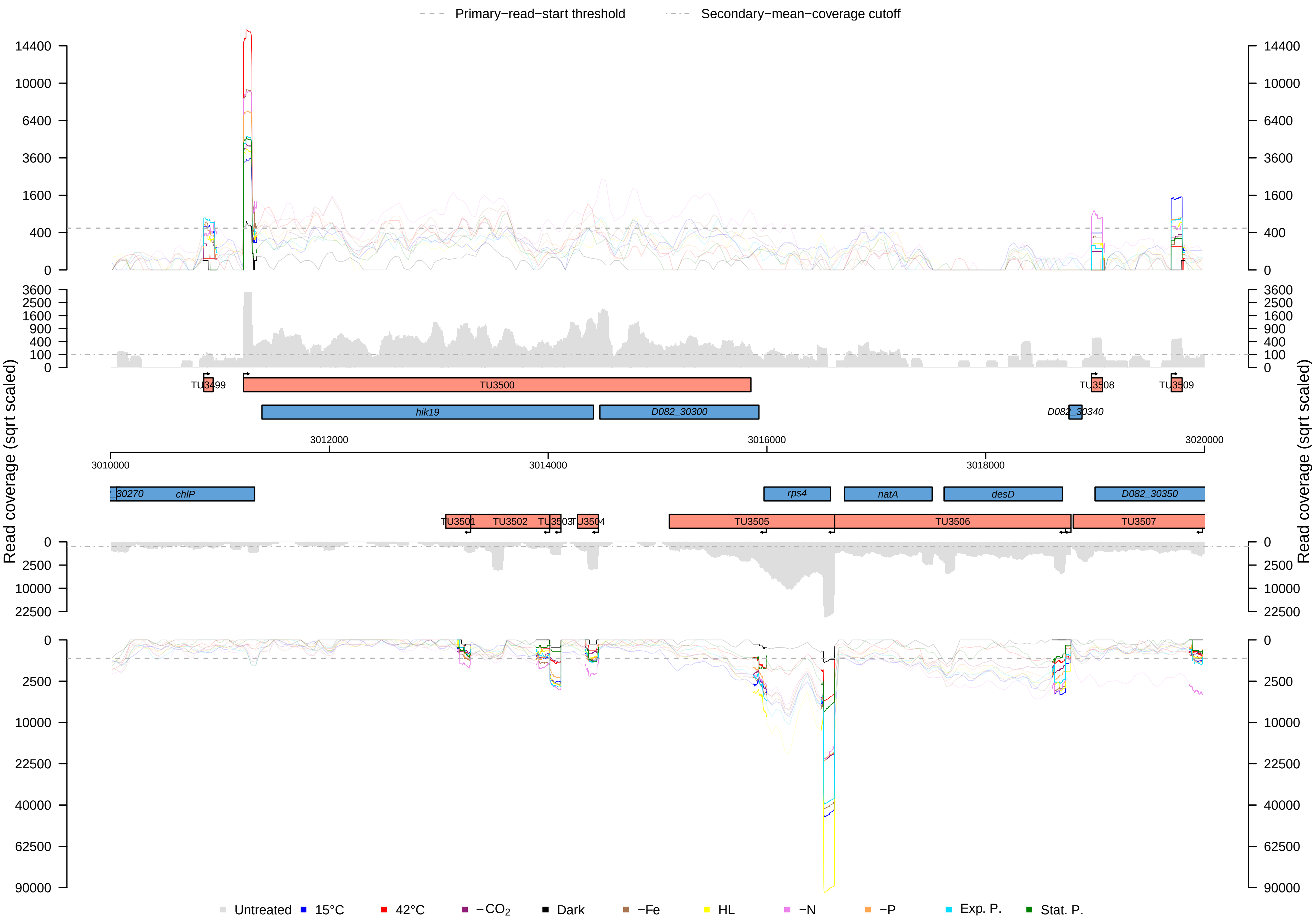

Primary−read−start threshold
Secondary−mean−coverage cutoff
Read coverage (sqrt scaled)
14400
10000
6400
3600
1600
400
0
2500
900
100
TU3499
TU3500
TU3508
TU3509
hik19
D082_30300
D082_30340
3010000
3012000
3014000
3016000
3018000
3020000
30270
chlP
rps4
natA
desD
D082_30350
TU3501
TU3502
TU3503
TU3504
TU3505
TU3506
TU3507
22500
40000
62500
90000
Untreated
15°C
42°C
−CO2
Dark
−Fe
HL
−N
−P
Exp. P.
Stat. P.

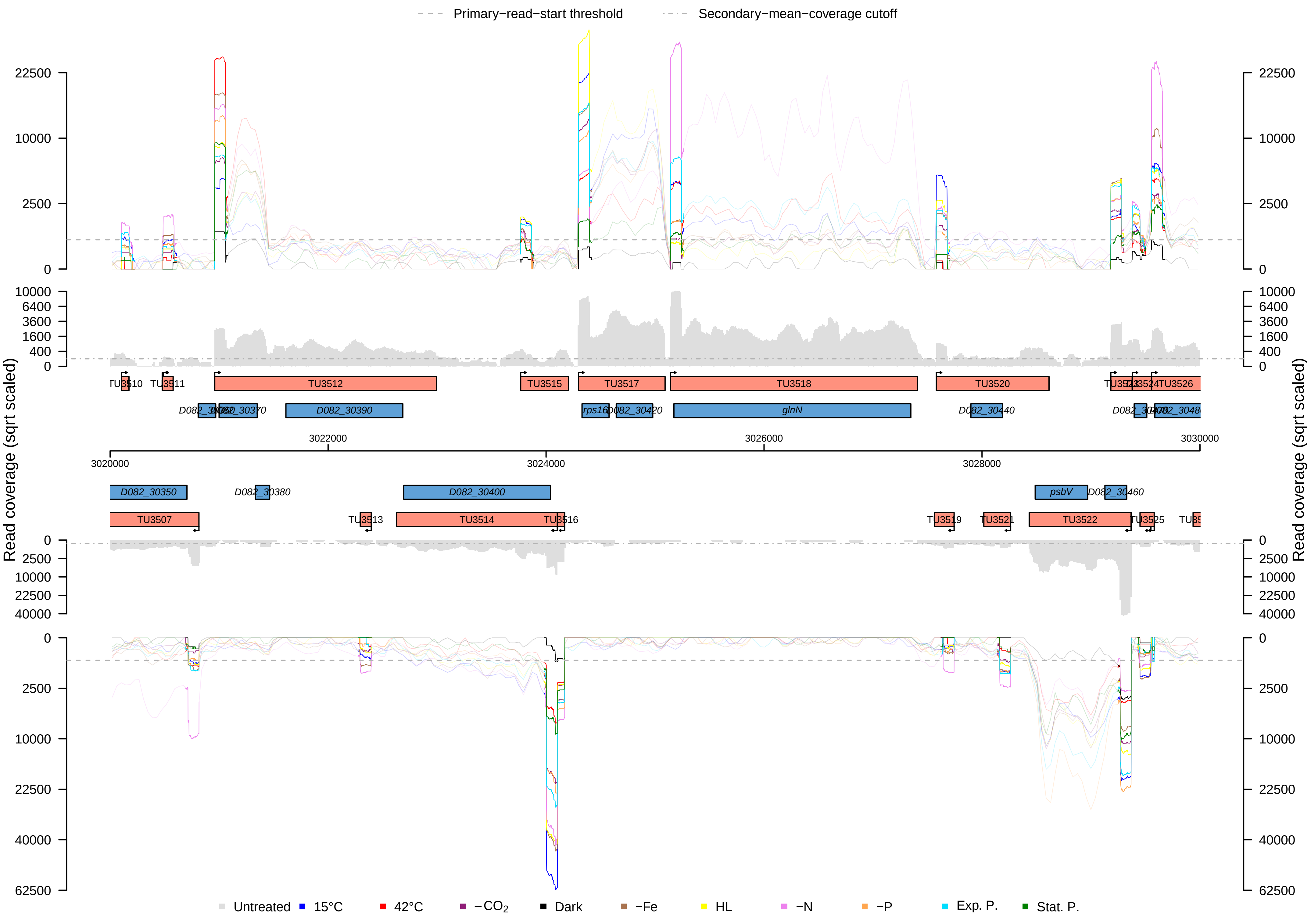

Primary−read−start threshold
Secondary−mean−coverage cutoff
Read coverage (sqrt scaled)
22500
10000
2500
0
10000
6400
3600
1600
400
0
TU3510
TU3511
TU3512
TU3515
TU3517
TU3518
TU3520
TU3526
D082_30390
rps16
D082_30420
glnN
D082_30440
3022000
3026000
3030000
3020000
3024000
3028000
D082_30350
D082_30380
D082_30400
psbV
D082_30460
TU3507
TU3513
TU3514
TU3516
TU3519
TU3522
TU3525
40000
62500
Untreated
15°C
42°C
−CO2
Dark
−Fe
HL
−N
−P
Exp. P.
Stat. P.

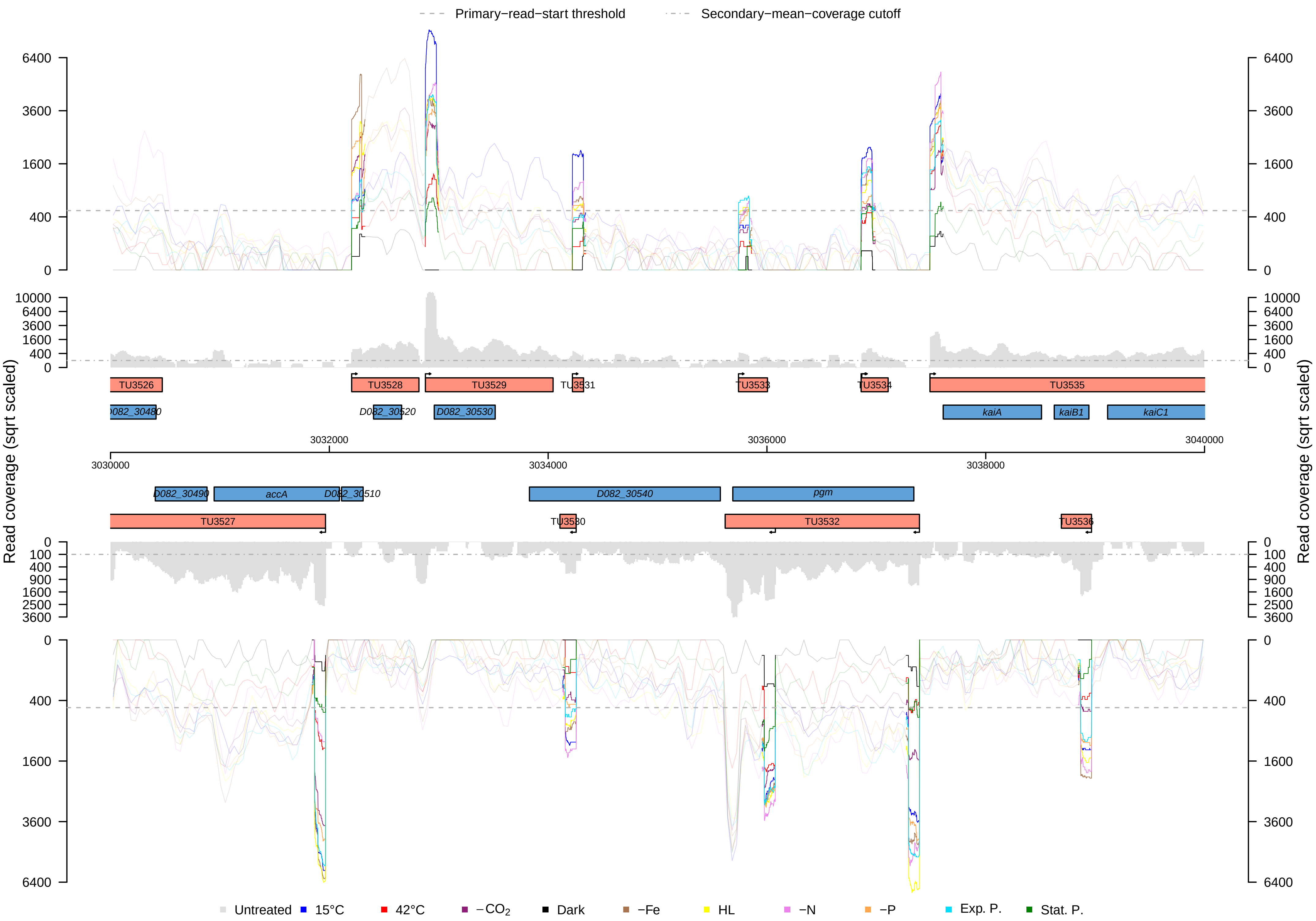

Primary−read−start threshold
Secondary−mean−coverage cutoff
Read coverage (sqrt scaled)
TU3526
TU3528
TU3529
TU3531
TU3533
TU3534
TU3535
D082_30520
D082_30530
kaiA
kaiB1
kaiC1
3030000
3032000
3034000
3036000
3038000
3040000
D082_30490
accA
D082_30510
D082_30540
pgm
TU3527
TU3530
TU3532
TU3536
Untreated
15°C
42°C
−CO2
Dark
−Fe
HL
−N
−P
Exp. P.
Stat. P.

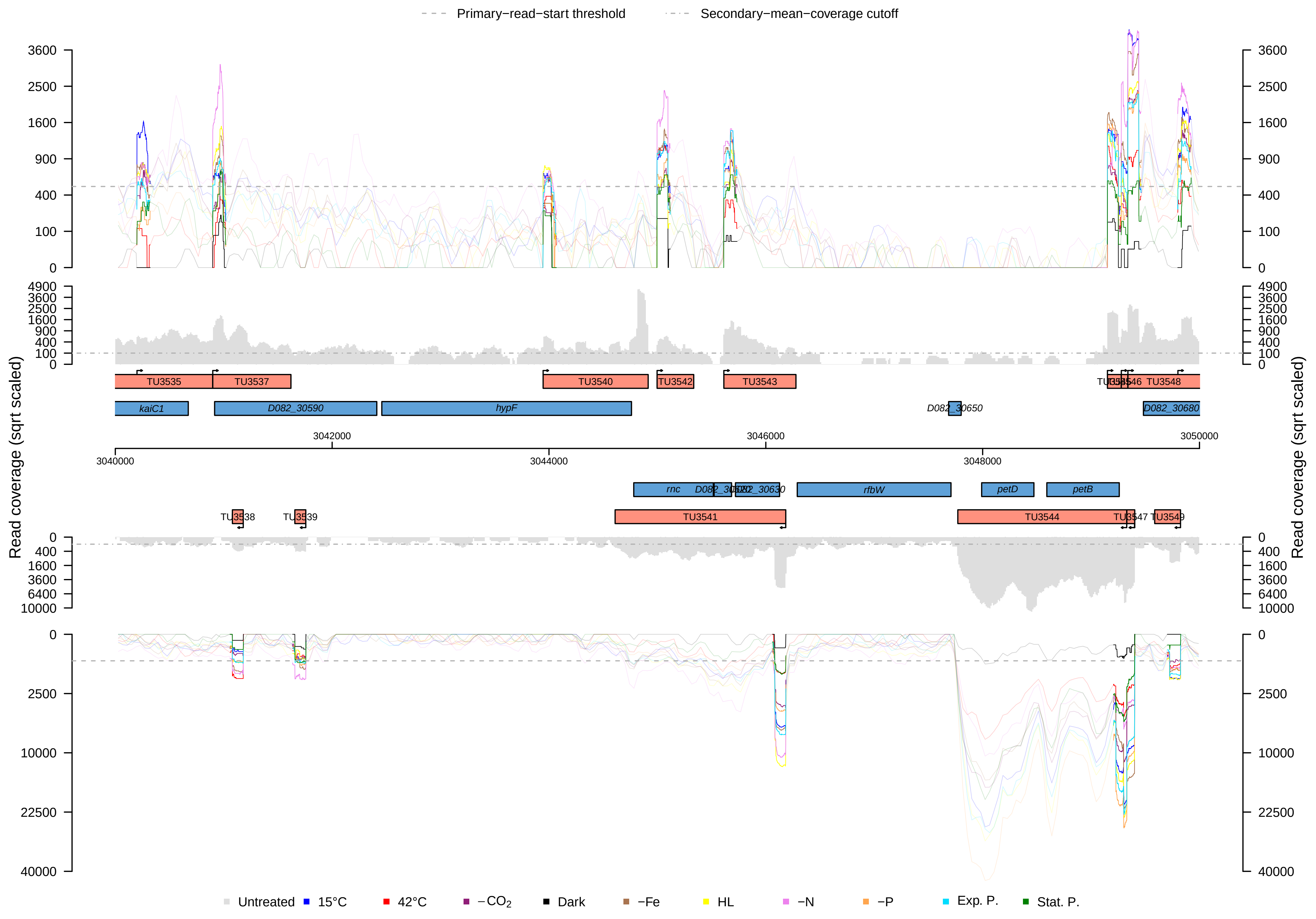

Primary−read−start threshold
Secondary−mean−coverage cutoff
Read coverage (sqrt scaled)
TU3535
TU3537
TU3540
TU3542
TU3543
TU3548
kaiC1
D082_30590
hypF
D082_30650
D082_30680
3040000
3042000
3044000
3046000
3048000
3050000
rnc
rfbW
petD
petB
TU3538
TU3539
TU3541
TU3544
TU3549
Untreated
15°C
42°C
−CO_2
Dark
−Fe
HL
−N
−P
Exp. P.
Stat. P.

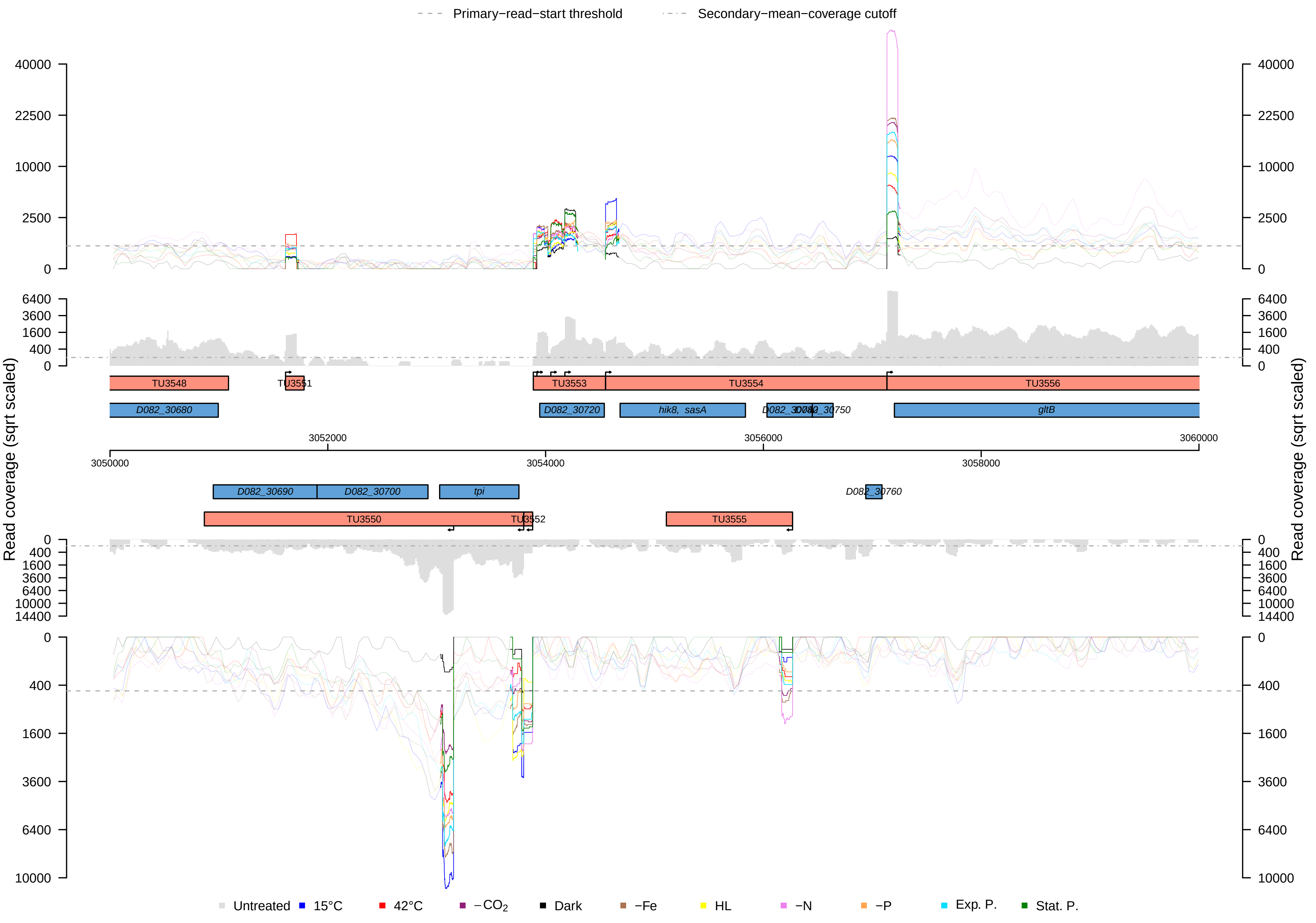

Primary−read−start threshold
Secondary−mean−coverage cutoff
Read coverage (sqrt scaled)
40000
22500
10000
2500
0
6400
3600
1600
400
TU3548
TU3551
TU3553
TU3554
TU3556
D082_30680
D082_30720
hik8, sasA
D082_30740
D082_30750
gltB
3050000
3052000
3054000
3056000
3058000
3060000
D082_30690
D082_30700
tpi
D082_30760
TU3550
TU3552
TU3555
10000
14400
Untreated
15°C
42°C
−CO_2
Dark
−Fe
HL
−N
−P
Exp. P.
Stat. P.

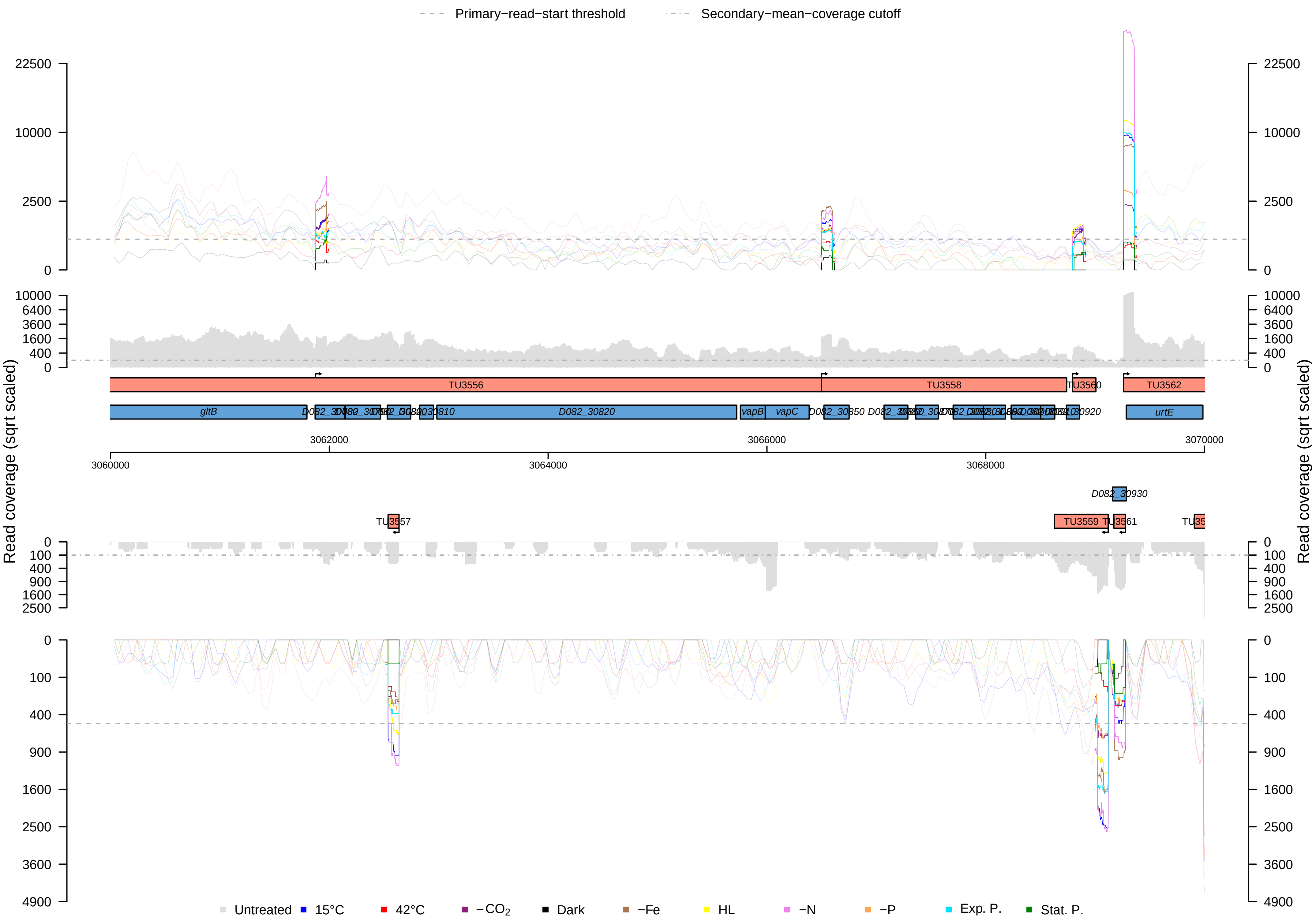

Primary−read−start threshold
Secondary−mean−coverage cutoff
Read coverage (sqrt scaled)
TU3556
TU3558
TU3560
TU3562
gltB
D082_30820
vapB
vapC
urtE
D082_30930
TU3557
TU3559
TU3561
3060000
3062000
3064000
3066000
3068000
3070000
Untreated
15°C
42°C
−CO2
Dark
−Fe
HL
−N
−P
Exp. P.
Stat. P.

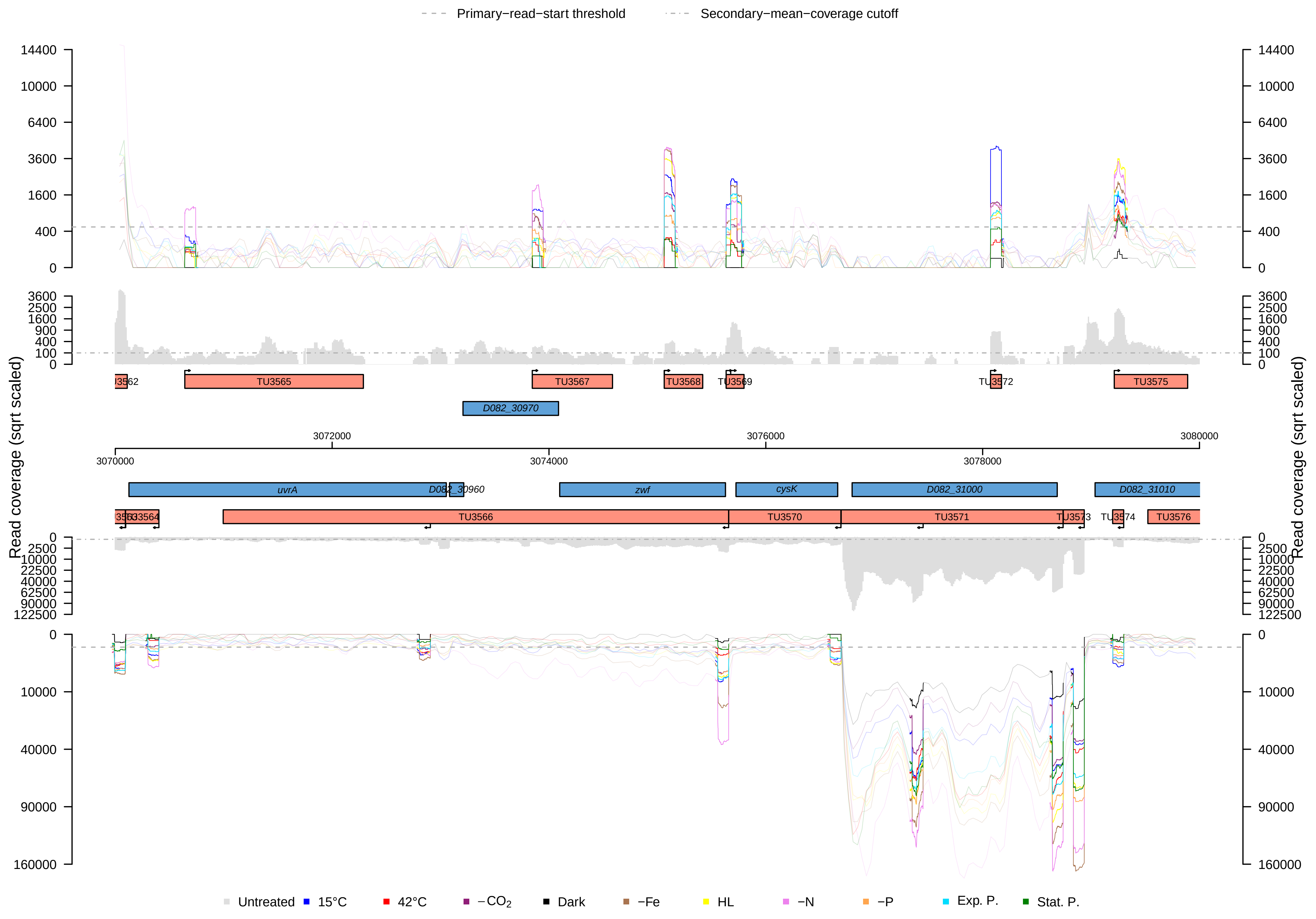

Primary−read−start threshold
Secondary−mean−coverage cutoff
Read coverage (sqrt scaled)
14400
10000
6400
3600
1600
400
0
2500
900
100
TU3565
TU3567
TU3568
TU3569
TU3572
TU3575
D082_30970
3070000
3072000
3074000
3076000
3078000
3080000
uvrA
D082_30960
zwf
cysK
D082_31000
D082_31010
TU3566
TU3570
TU3571
TU3573
TU3574
TU3576
22500
40000
62500
90000
122500
160000
Untreated
15°C
42°C
−CO2
Dark
−Fe
HL
−N
−P
Exp. P.
Stat. P.

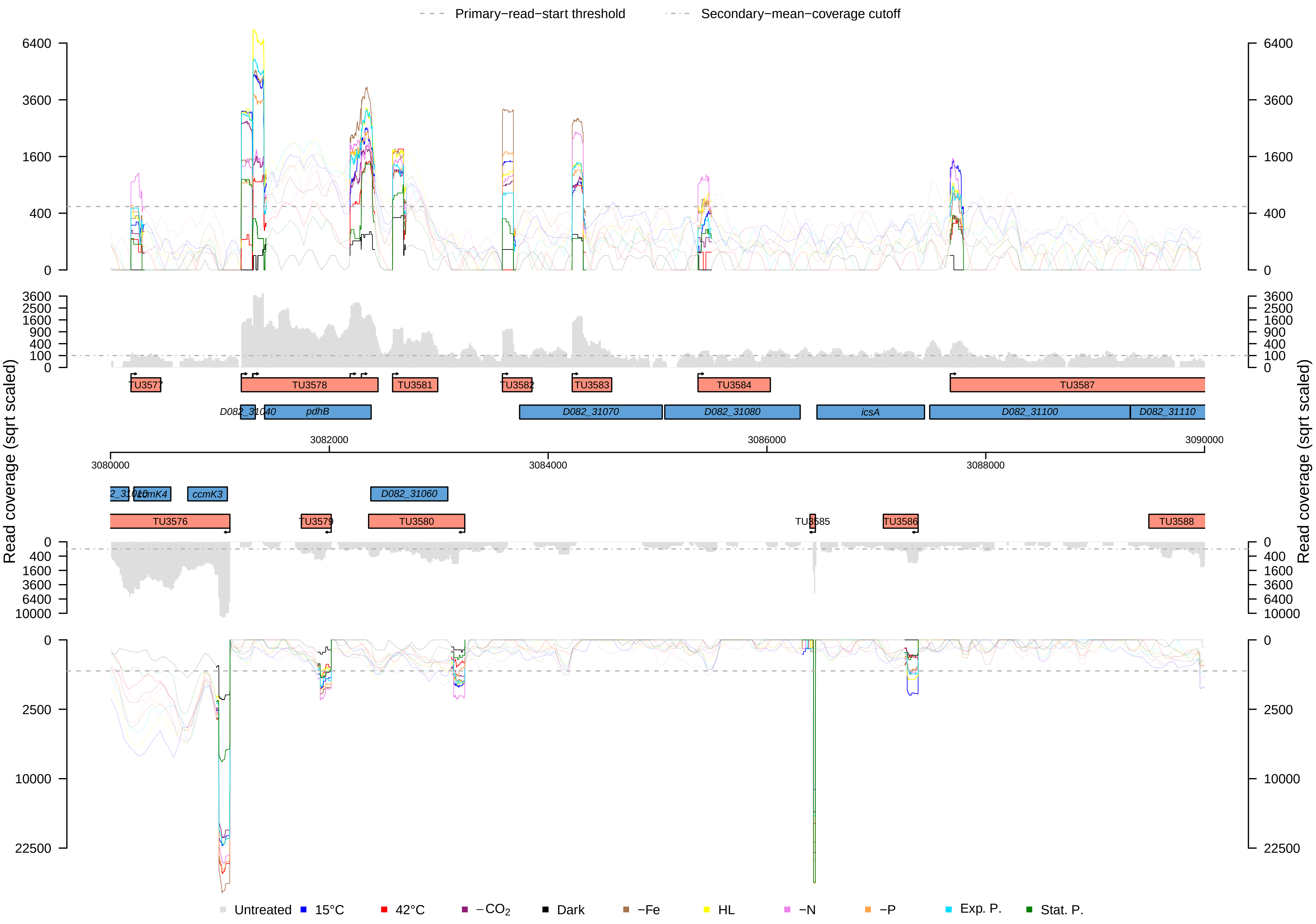

Primary−read−start threshold
Secondary−mean−coverage cutoff
Read coverage (sqrt scaled)
6400
3600
1600
400
0
3600
2500
1600
900
400
100
0
TU3577
TU3578
TU3581
TU3582
TU3583
TU3584
TU3587
D082_31040
pdhB
D082_31070
D082_31080
icsA
D082_31100
D082_31110
3080000
3082000
3084000
3086000
3088000
3090000
ccmK4
ccmK3
D082_31060
TU3576
TU3579
TU3580
TU3585
TU3586
TU3588
10000
2500
22500
Untreated
15°C
42°C
−CO2
Dark
−Fe
HL
−N
−P
Exp. P.
Stat. P.

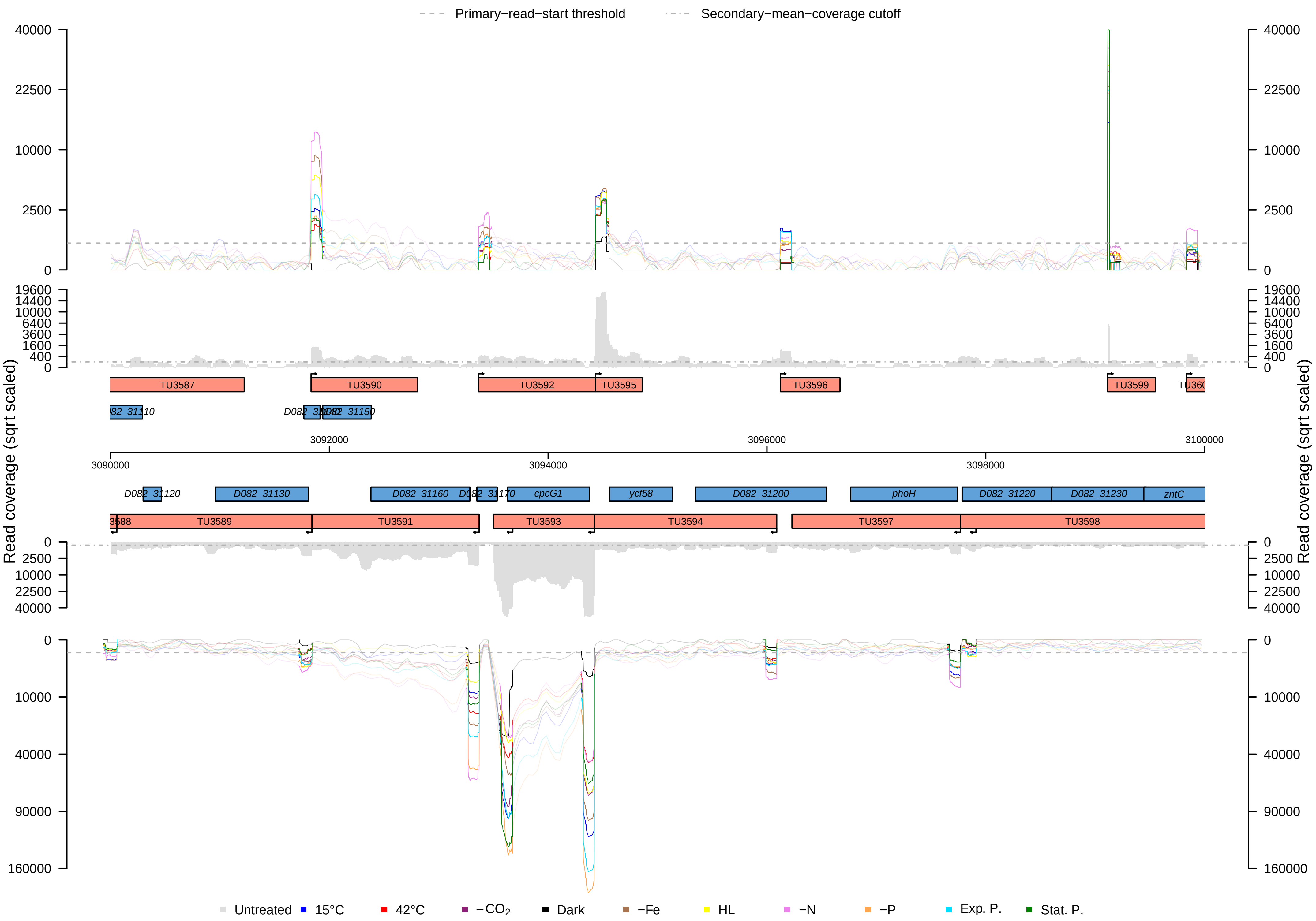

Primary−read−start threshold
Secondary−mean−coverage cutoff
Read coverage (sqrt scaled)
40000
22500
10000
2500
0
19600
14400
6400
3600
1600
400
TU3587
TU3590
TU3592
TU3595
TU3596
TU3599
D082_31150
3090000
3092000
3094000
3096000
3098000
3100000
D082_31130
D082_31160
cpcG1
ycf58
D082_31200
phoH
D082_31220
D082_31230
zntC
TU3589
TU3591
TU3593
TU3594
TU3597
TU3598
90000
160000
Untreated
15°C
42°C
−CO2
Dark
−Fe
HL
−N
−P
Exp. P.
Stat. P.

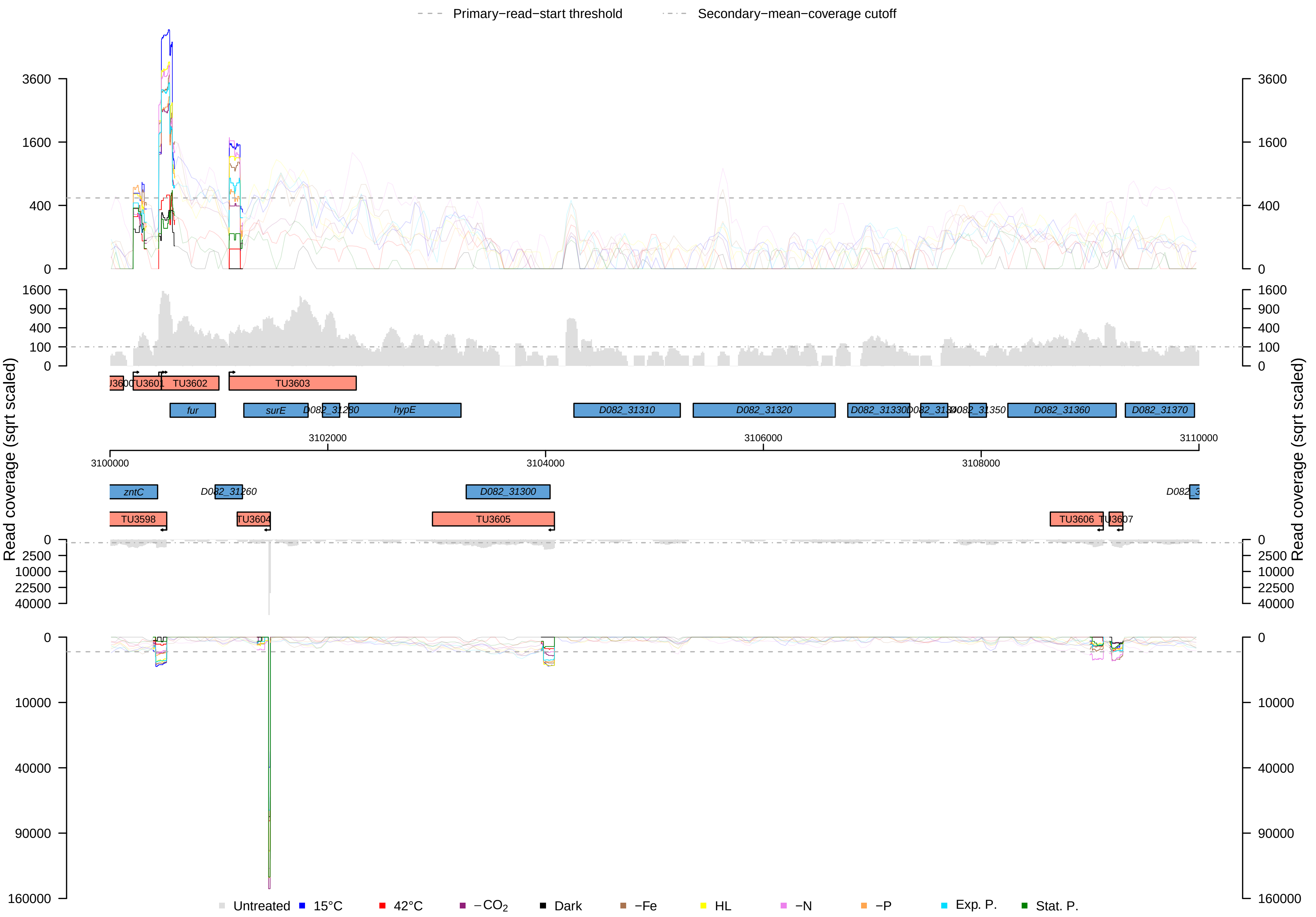

Primary−read−start threshold
Secondary−mean−coverage cutoff
Read coverage (sqrt scaled)
3600
1600
400
0
1600
900
400
100
0
TU3601
TU3602
TU3603
fur
surE
D082_31280
hypE
D082_31310
D082_31320
D082_31330
D082_31350
D082_31360
D082_31370
3100000
3102000
3104000
3106000
3108000
3110000
zntC
D082_31260
D082_31300
TU3598
TU3604
TU3605
TU3606
TU3607
0
2500
10000
22500
40000
0
10000
40000
90000
160000
Untreated
15°C
42°C
−CO2
Dark
−Fe
HL
−N
−P
Exp. P.
Stat. P.

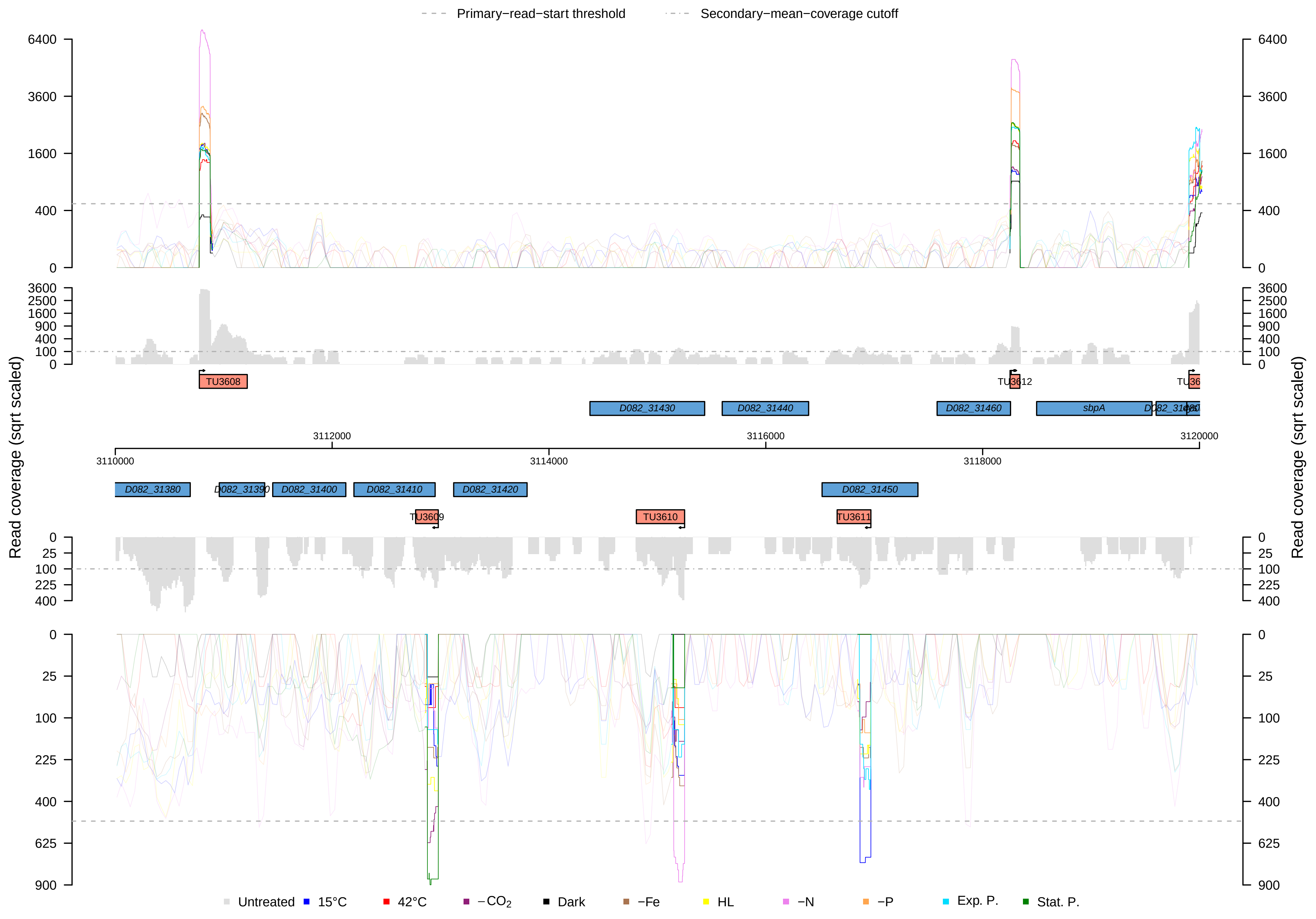

Primary−read−start threshold
Secondary−mean−coverage cutoff
Read coverage (sqrt scaled)
6400
3600
1600
400
0
2500
900
100
25
225
625
TU3608
TU3609
TU3610
TU3611
TU3612
D082_31380
D082_31390
D082_31400
D082_31410
D082_31420
D082_31430
D082_31440
D082_31450
D082_31460
sbpA
3110000
3112000
3114000
3116000
3118000
3120000
Untreated
15°C
42°C
$-CO_2$
Dark
−Fe
HL
−N
−P
Exp. P.
Stat. P.

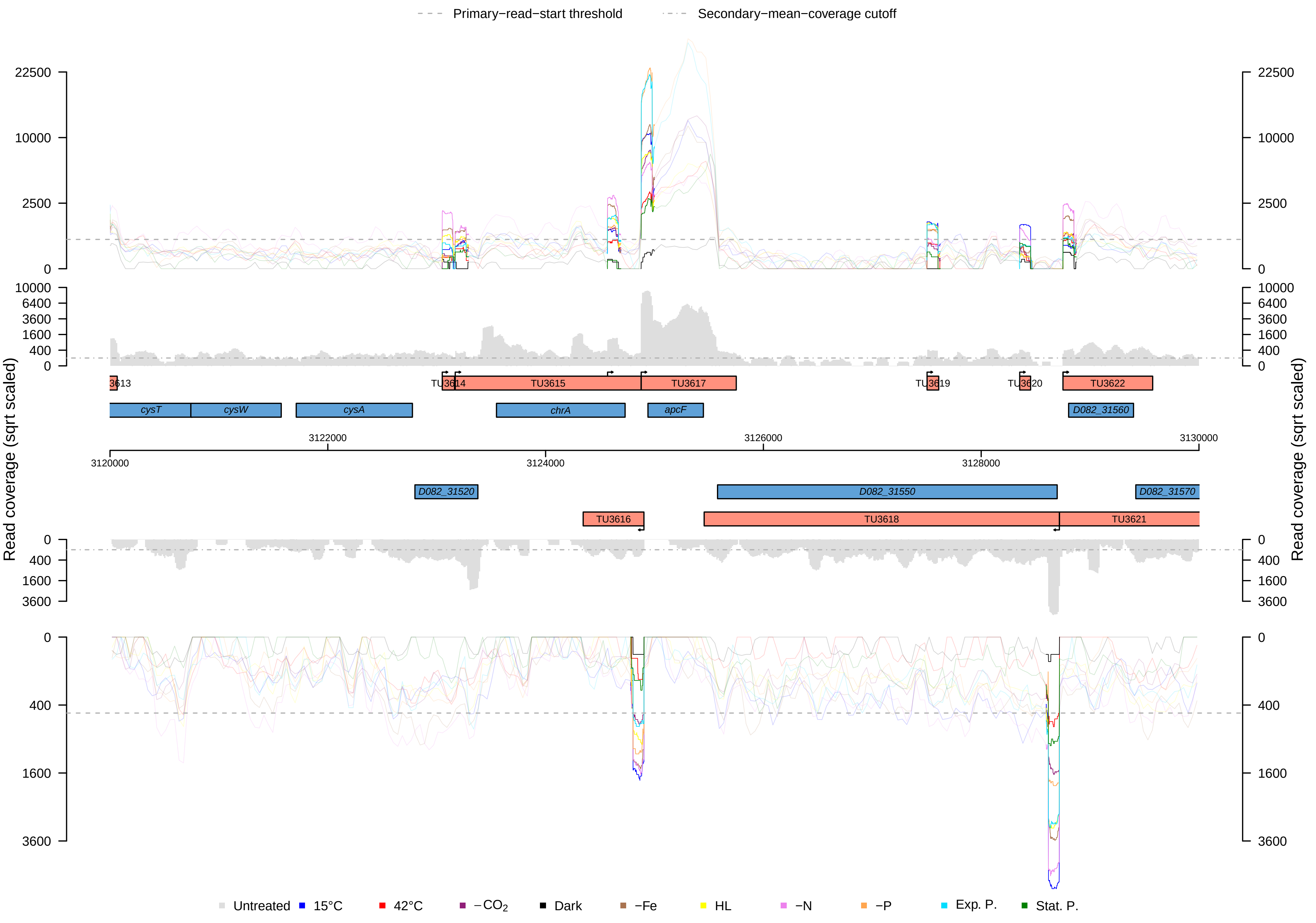

Primary−read−start threshold
Secondary−mean−coverage cutoff
22500
10000
2500
0
10000
6400
3600
1600
400
0
Read coverage (sqrt scaled)
3613
TU3614
TU3615
TU3617
TU3619
TU3620
TU3622
cysT
cysW
cysA
chrA
apcF
D082_31560
3122000
3126000
3130000
3120000
3124000
3128000
D082_31520
D082_31550
D082_31570
TU3616
TU3618
TU3621
3600
1600
400
Untreated
15°C
42°C
−CO2
Dark
−Fe
HL
−N
−P
Exp. P.
Stat. P.

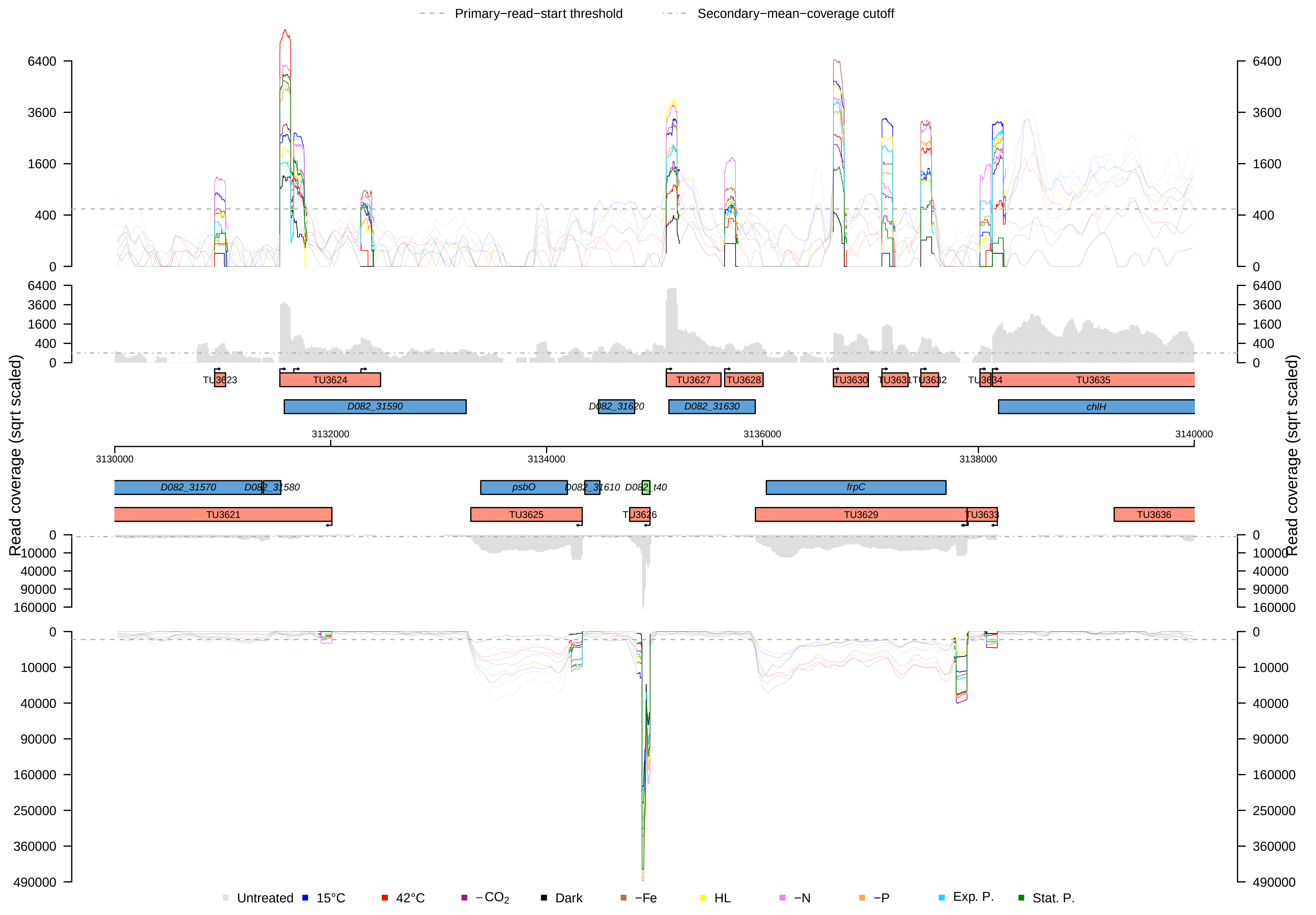

Primary−read−start threshold
Secondary−mean−coverage cutoff
Read coverage (sqrt scaled)
6400
3600
1600
400
0
10000
40000
90000
160000
250000
360000
490000
TU3623
TU3624
TU3627
TU3628
TU3630
TU3631
TU3632
TU3634
TU3635
D082_31590
D082_31620
D082_31630
chlH
3130000
3132000
3134000
3136000
3138000
3140000
D082_31570
D082_31580
psbO
D082_31610
D082_t40
frpC
TU3621
TU3625
TU3626
TU3629
TU3633
TU3636
Untreated
15°C
42°C
−CO2
Dark
−Fe
HL
−N
−P
Exp. P.
Stat. P.

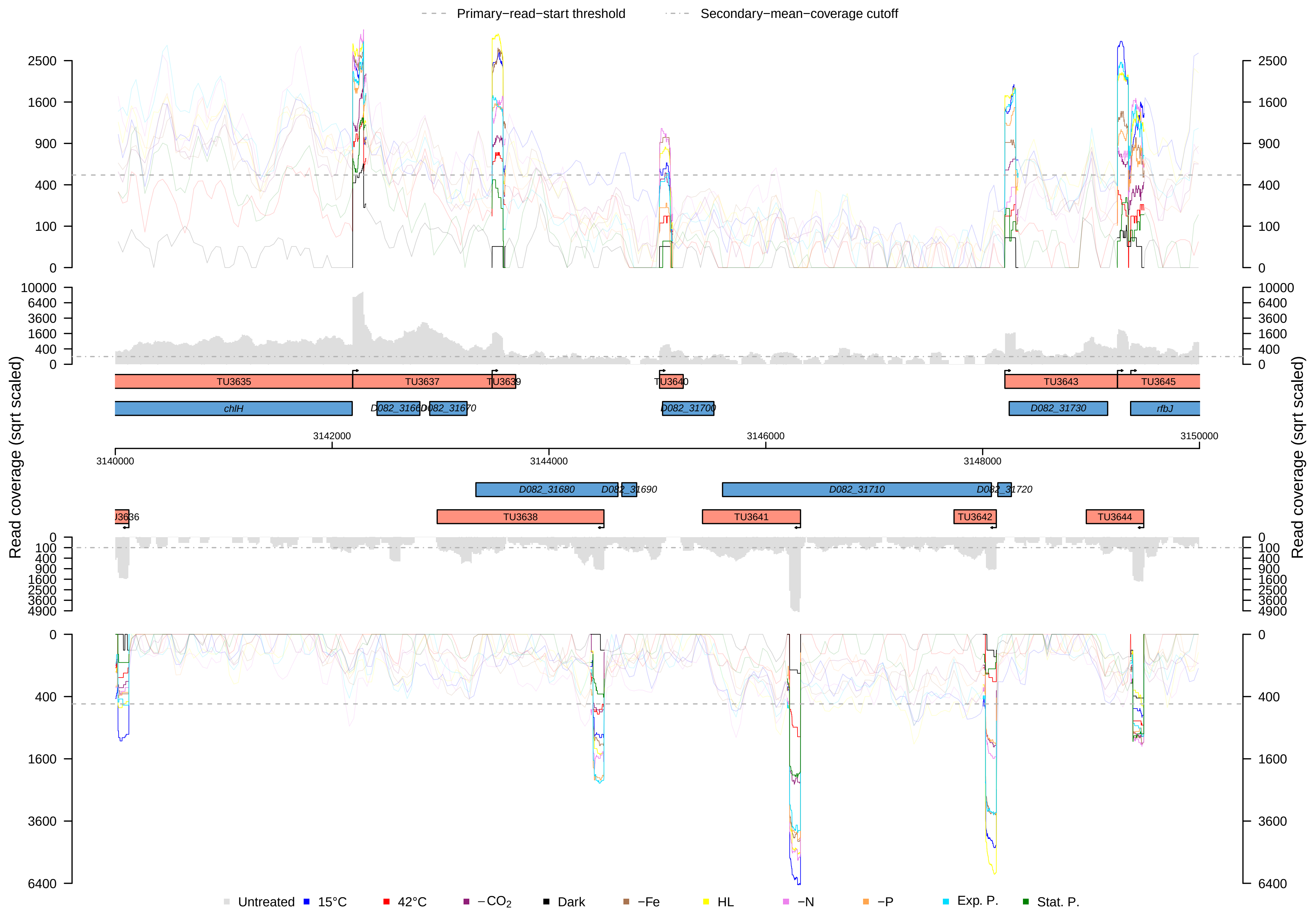

Primary−read−start threshold
Secondary−mean−coverage cutoff
Read coverage (sqrt scaled)
2500
1600
900
400
100
0
10000
6400
3600
1600
400
0
TU3635
TU3637
TU3639
TU3640
TU3643
TU3645
chlH
D082_31660
D082_31670
D082_31700
D082_31730
rfbJ
3140000
3142000
3144000
3146000
3148000
3150000
D082_31680
D082_31690
D082_31710
D082_31720
TU3636
TU3638
TU3641
TU3642
TU3644
100
400
900
1600
2500
3600
4900
6400
Untreated
15°C
42°C
−CO2
Dark
−Fe
HL
−N
−P
Exp. P.
Stat. P.

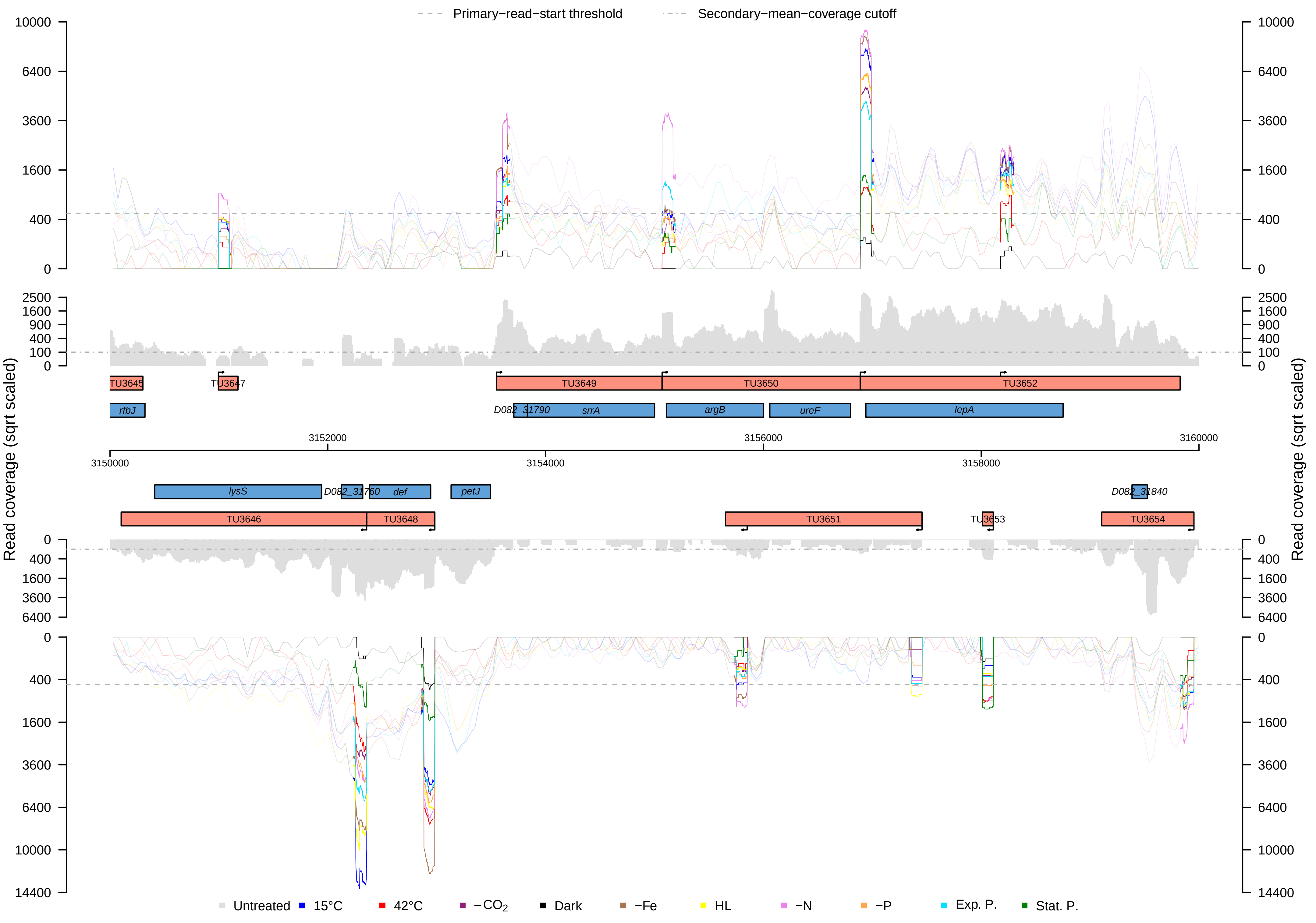
Primary−read−start threshold
Secondary−mean−coverage cutoff
Read coverage (sqrt scaled)
TU3645
TU3647
TU3649
TU3650
TU3652
rfbJ
D082_31790
srrA
argB
ureF
lepA
3150000
3152000
3154000
3156000
3158000
3160000
lysS
D082_31760
def
petJ
D082_31840
TU3646
TU3648
TU3651
TU3653
TU3654
Untreated
15°C
42°C
−CO2
Dark
−Fe
HL
−N
−P
Exp. P.
Stat. P.

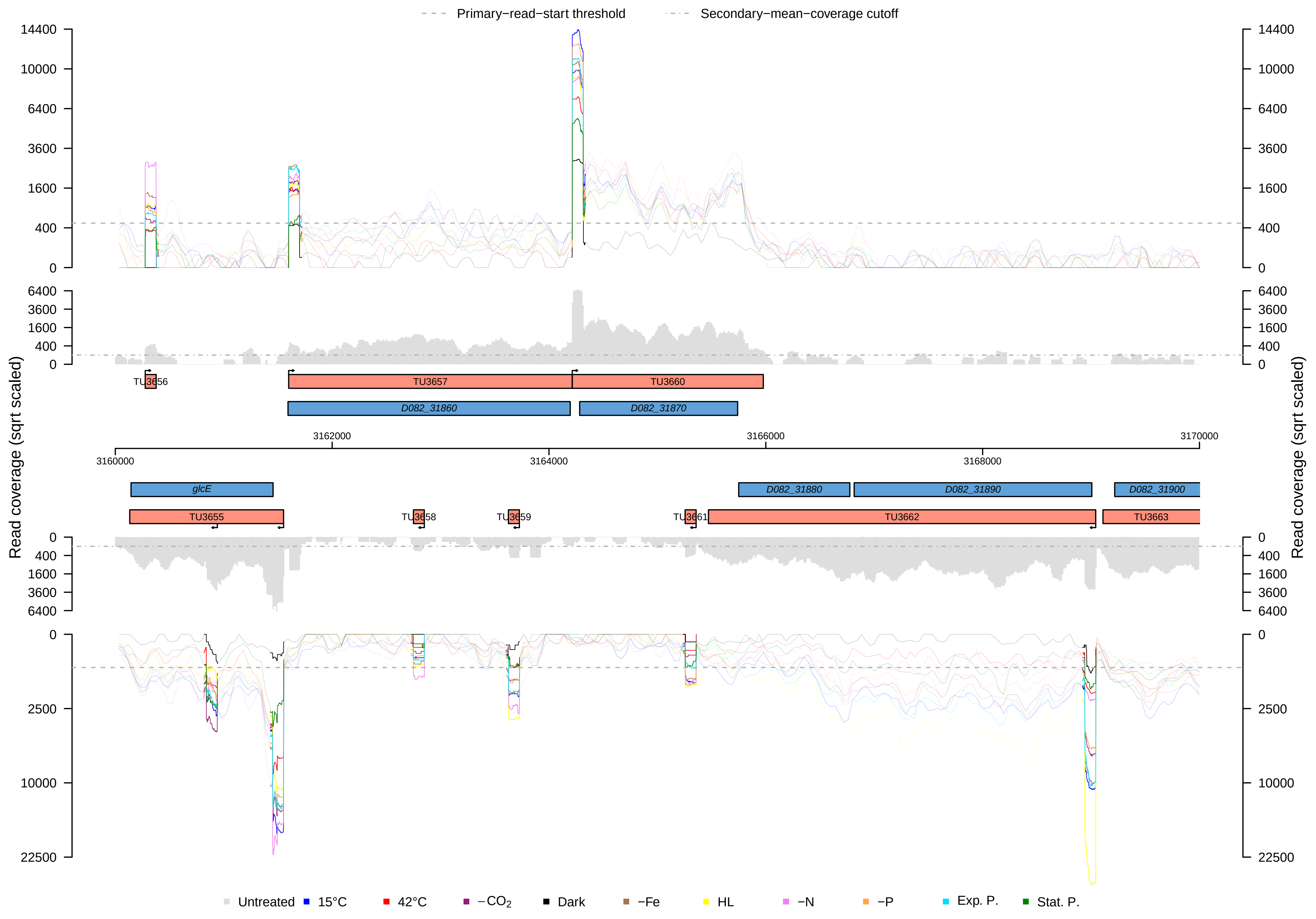

Primary−read−start threshold
Secondary−mean−coverage cutoff
Read coverage (sqrt scaled)
TU3656
TU3657
TU3660
D082_31860
D082_31870
3160000
3162000
3164000
3166000
3168000
3170000
glcE
D082_31880
D082_31890
D082_31900
TU3655
TU3658
TU3659
TU3661
TU3662
TU3663
Untreated
15°C
42°C
−CO_2
Dark
−Fe
HL
−N
−P
Exp. P.
Stat. P.

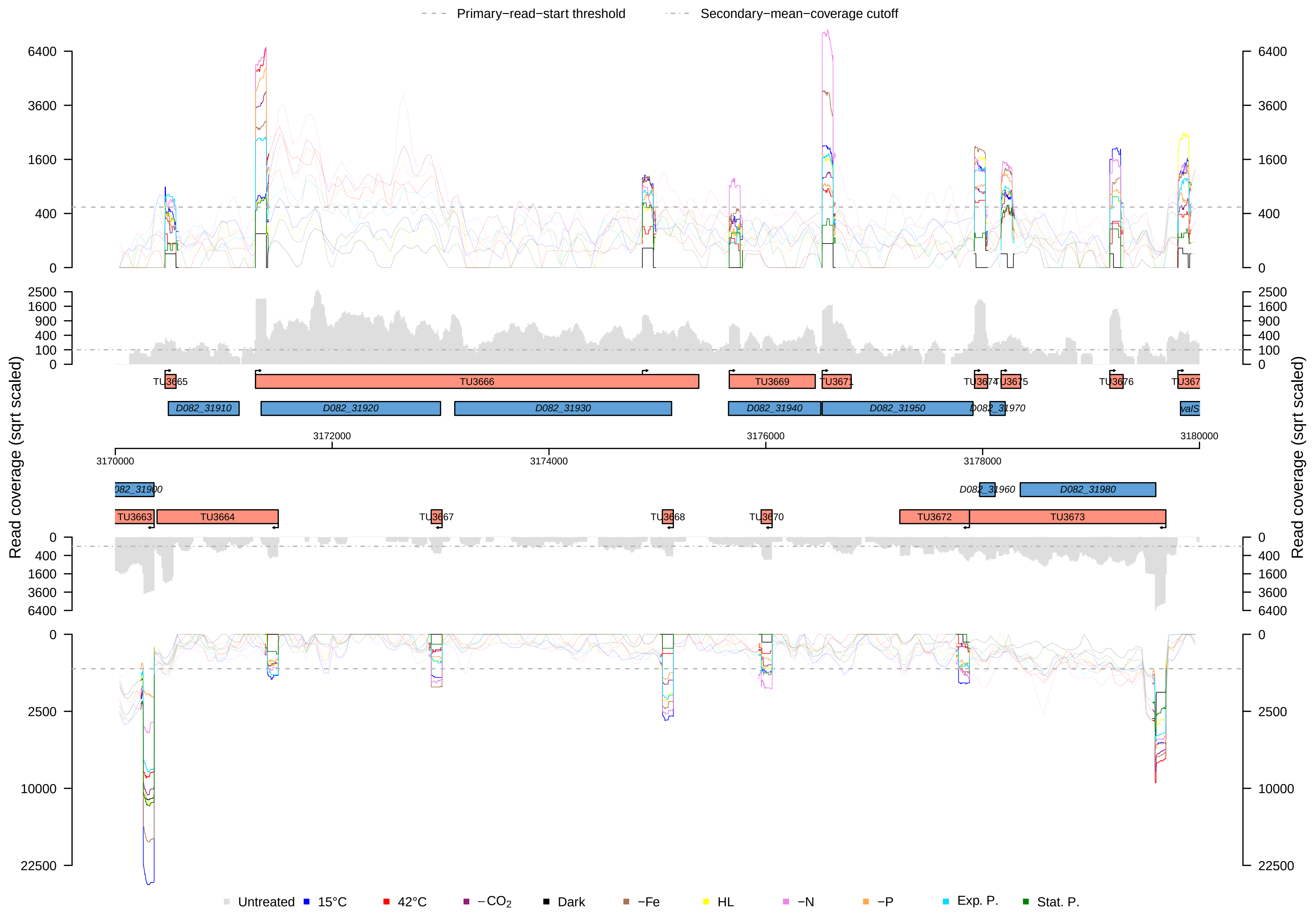
Primary−read−start threshold
Secondary−mean−coverage cutoff
Read coverage (sqrt scaled)
6400
3600
1600
400
0
2500
1600
900
400
100
0
TU3665
TU3666
TU3669
TU3671
TU3674
TU3675
TU3676
TU367
D082_31910
D082_31920
D082_31930
D082_31940
D082_31950
D082_31970
valS
3170000
3172000
3174000
3176000
3178000
3180000
082_31900
D082_31960
D082_31980
TU3663
TU3664
TU3667
TU3668
TU3670
TU3672
TU3673
10000
22500
Untreated
15°C
42°C
−CO2
Dark
−Fe
HL
−N
−P
Exp. P.
Stat. P.

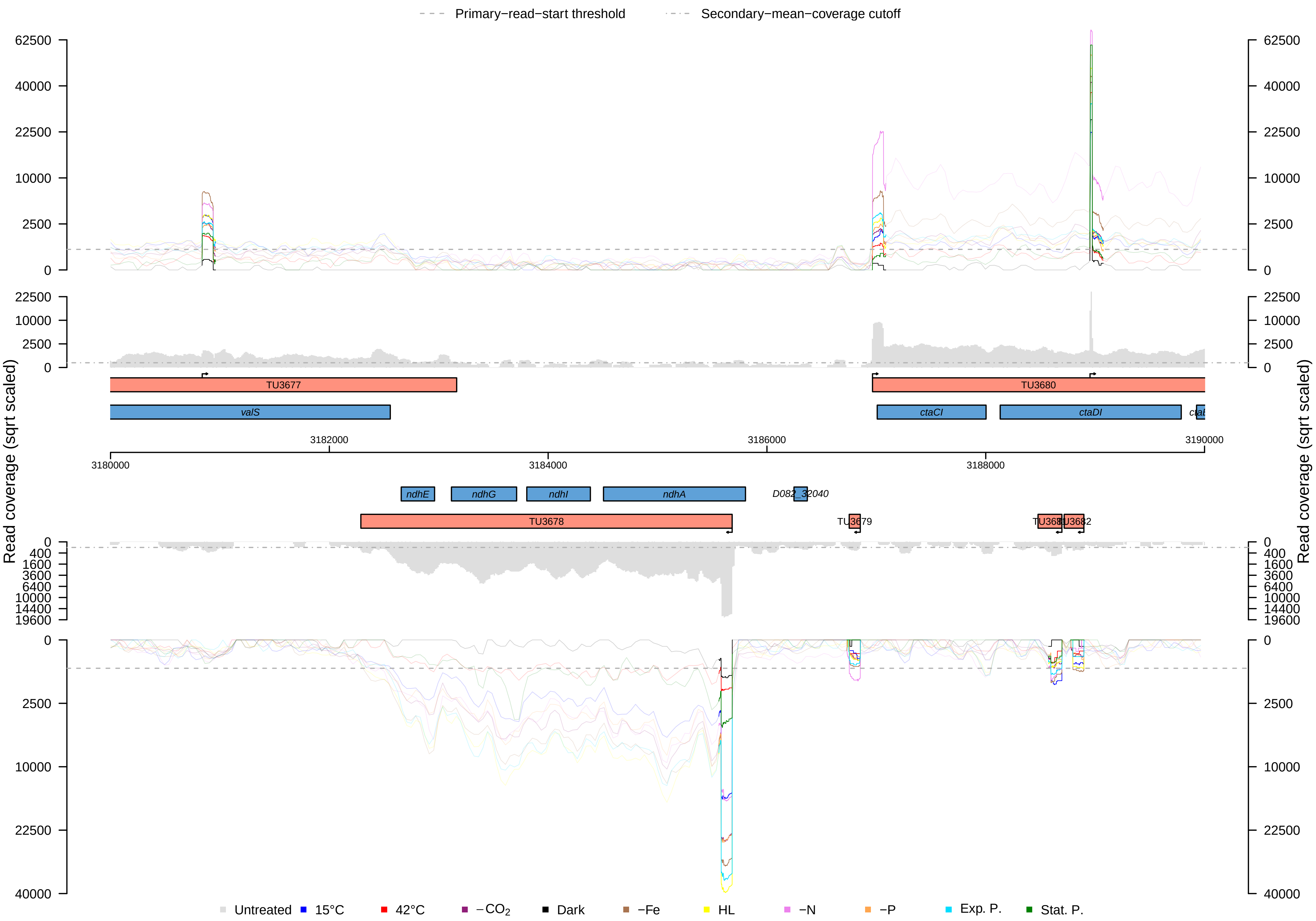

Primary−read−start threshold
Secondary−mean−coverage cutoff
Read coverage (sqrt scaled)
62500
40000
22500
10000
2500
0
TU3677
TU3680
valS
ctaCI
ctaDI
3180000
3182000
3184000
3186000
3188000
3190000
ndhE
ndhG
ndhI
ndhA
D082_32040
TU3678
TU3679
TU3681
TU3682
400
1600
3600
6400
14400
19600
Untreated
15°C
42°C
−CO2
Dark
−Fe
HL
−N
−P
Exp. P.
Stat. P.

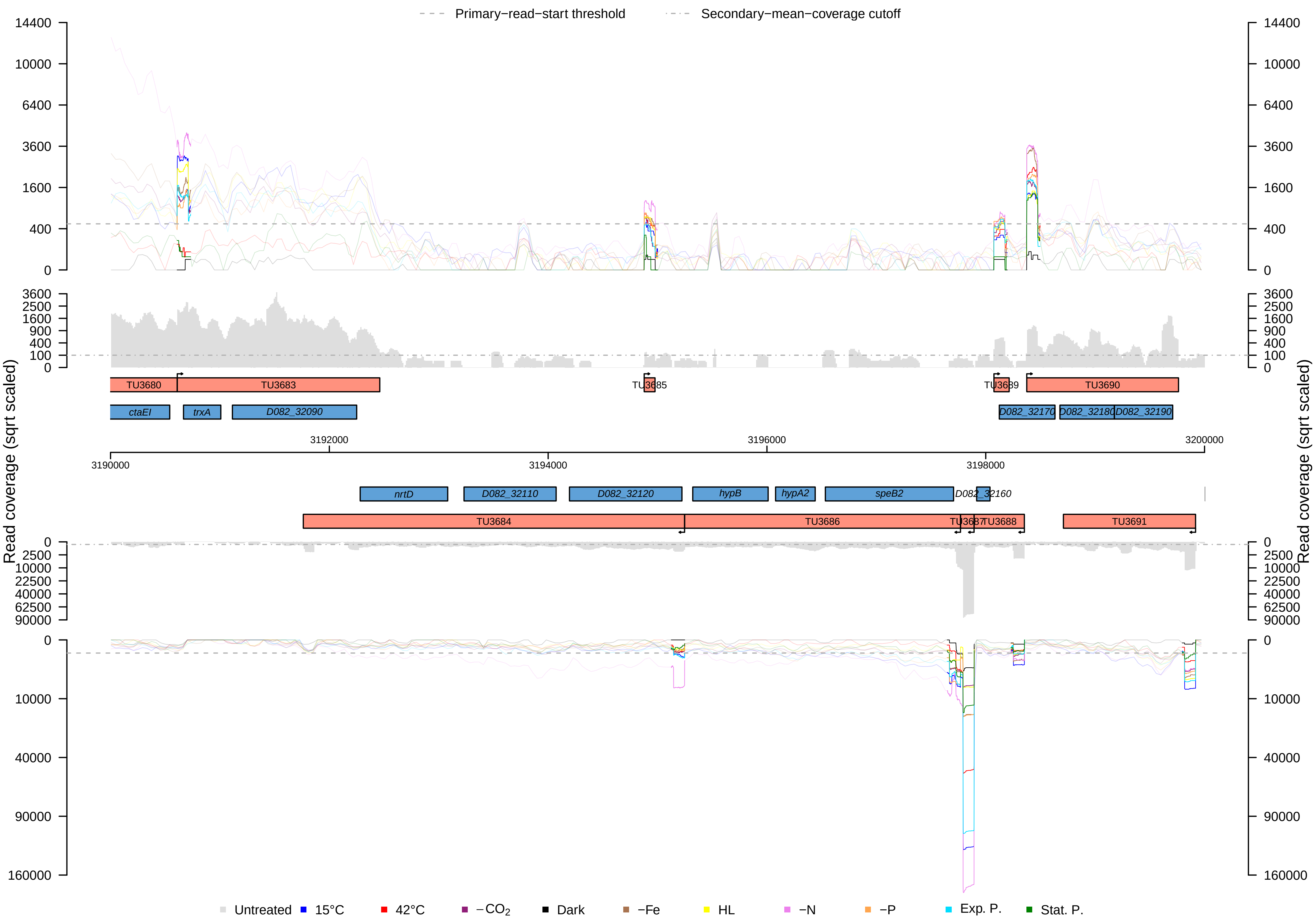

Primary−read−start threshold
Secondary−mean−coverage cutoff
Read coverage (sqrt scaled)
14400
10000
6400
3600
1600
400
0
3600
2500
1600
900
400
100
0
TU3680
TU3683
TU3685
TU3689
TU3690
ctaEI
trxA
D082_32090
D082_32170
D082_32180
D082_32190
3190000
3192000
3194000
3196000
3198000
3200000
nrtD
D082_32110
D082_32120
hypB
hypA2
speB2
D082_32160
TU3684
TU3686
TU3687
TU3688
TU3691
2500
10000
22500
40000
62500
90000
160000
Untreated
15°C
42°C
−CO2
Dark
−Fe
HL
−N
−P
Exp. P.
Stat. P.

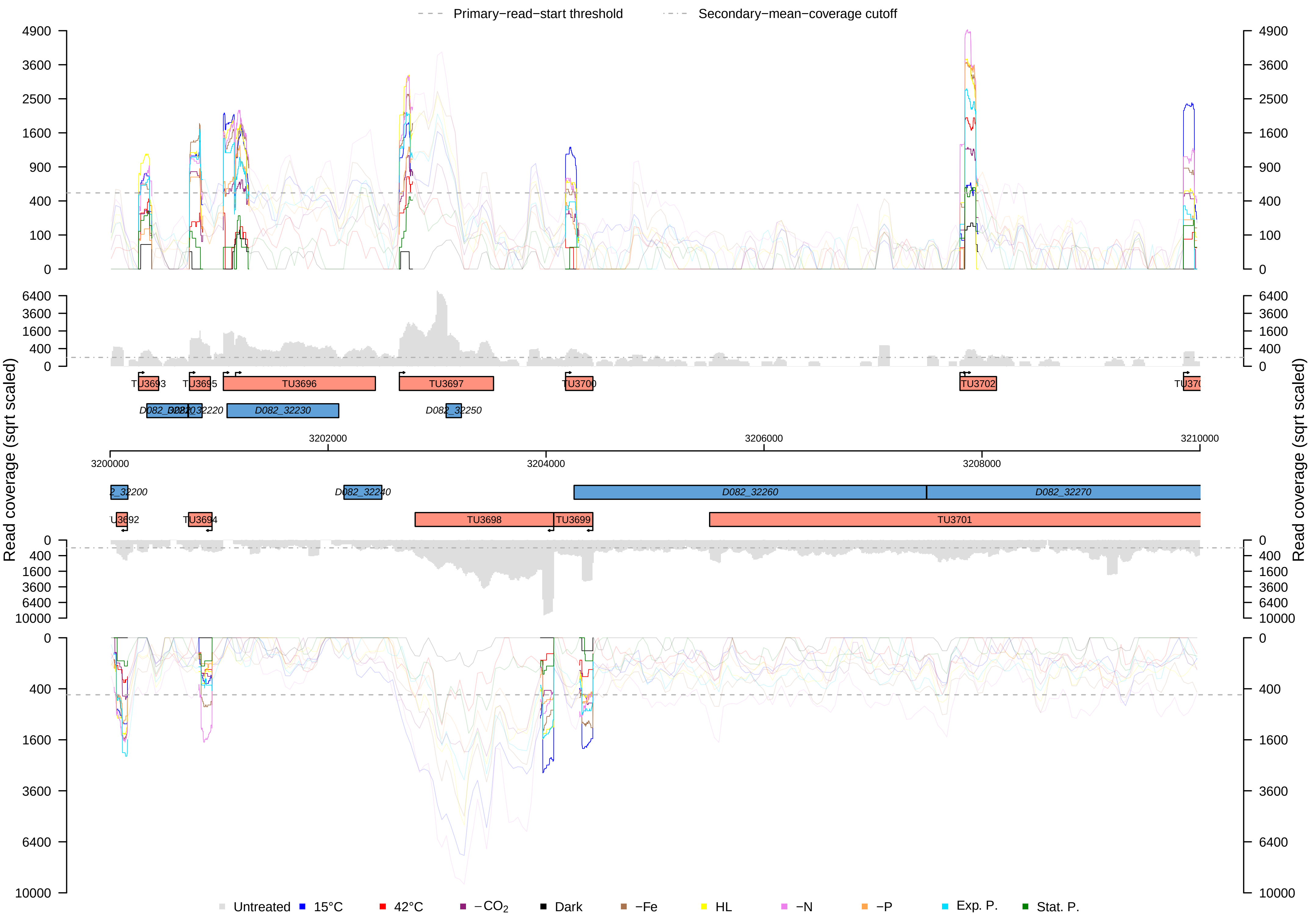
Primary−read−start threshold
Secondary−mean−coverage cutoff
Read coverage (sqrt scaled)
TU3693
TU3695
TU3696
TU3697
TU3700
TU3702
D082_32230
3202000
3206000
3210000
3200000
3204000
3208000
D082_32240
D082_32260
D082_32270
TU3694
TU3698
TU3699
TU3701
Untreated
15°C
42°C
−CO2
Dark
−Fe
HL
−N
−P
Exp. P.
Stat. P.

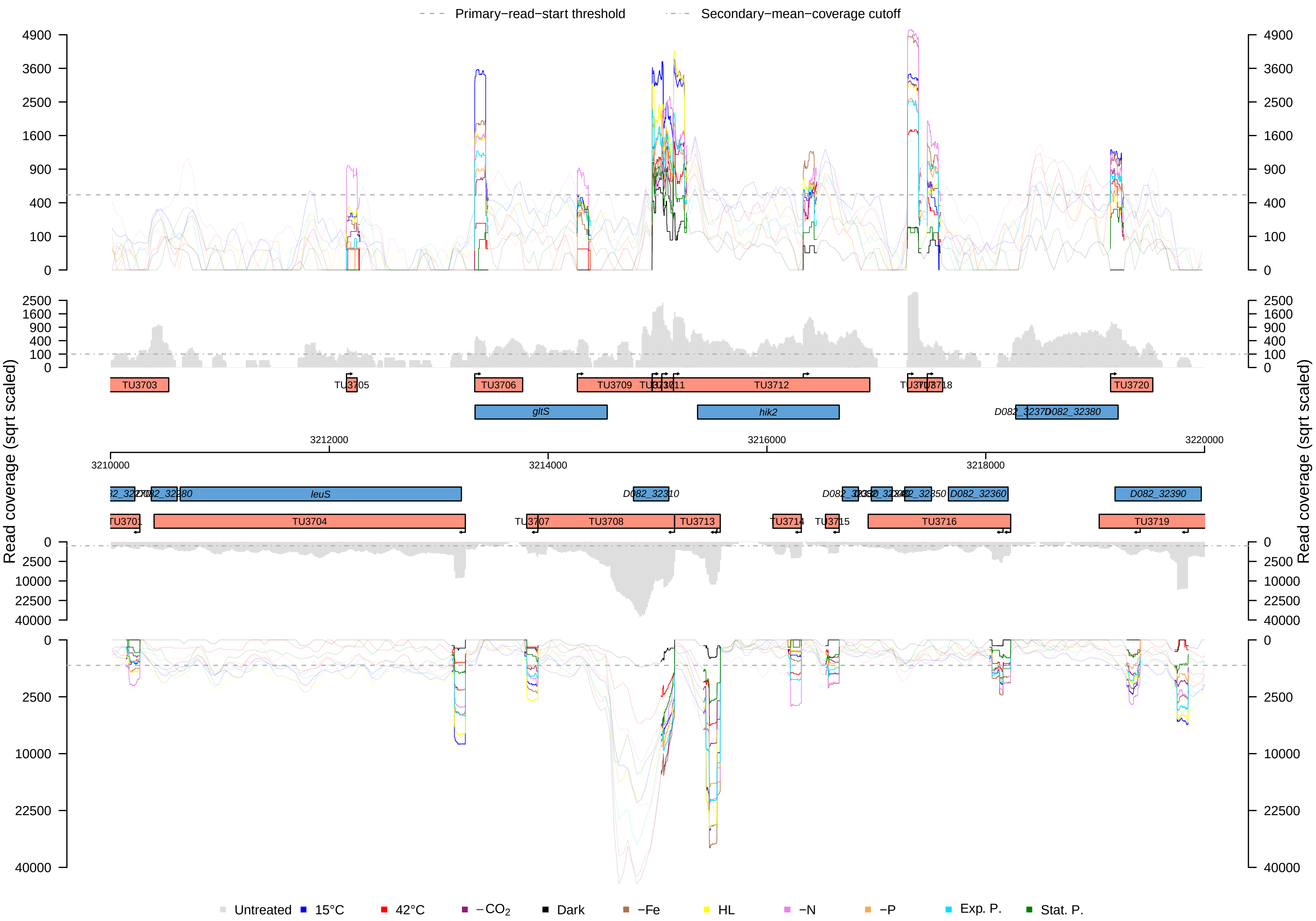

Primary−read−start threshold
Secondary−mean−coverage cutoff
Read coverage (sqrt scaled)
4900
3600
2500
1600
900
400
100
0
TU3703
TU3705
TU3706
TU3709
TU3712
TU3720
gltS
hik2
3210000
3212000
3214000
3216000
3218000
3220000
leuS
TU3701
TU3704
TU3707
TU3708
TU3713
TU3714
TU3715
TU3716
TU3719
10000
22500
40000
Untreated
15°C
42°C
−CO2
Dark
−Fe
HL
−N
−P
Exp. P.
Stat. P.

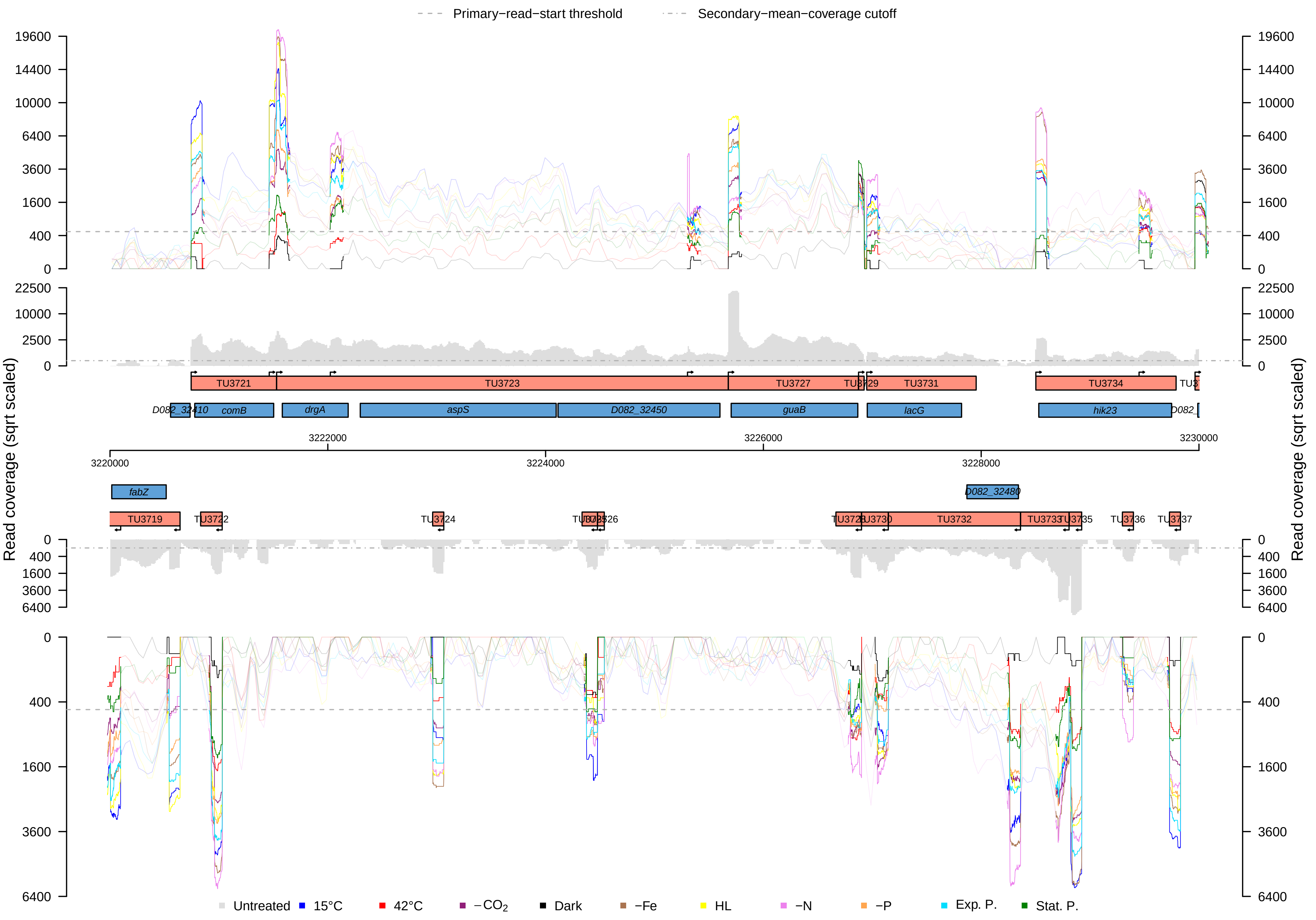

Primary−read−start threshold
Secondary−mean−coverage cutoff
Read coverage (sqrt scaled)
19600
14400
10000
6400
3600
1600
400
0
22500
10000
2500
TU3721
TU3723
TU3727
TU3731
TU3734
D082_32410
comB
drgA
aspS
D082_32450
guaB
lacG
hik23
3220000
3222000
3224000
3226000
3228000
3230000
fabZ
D082_32480
TU3719
TU3722
TU3724
TU3732
TU3736
TU3737
Untreated
15°C
42°C
−CO2
Dark
−Fe
HL
−N
−P
Exp. P.
Stat. P.

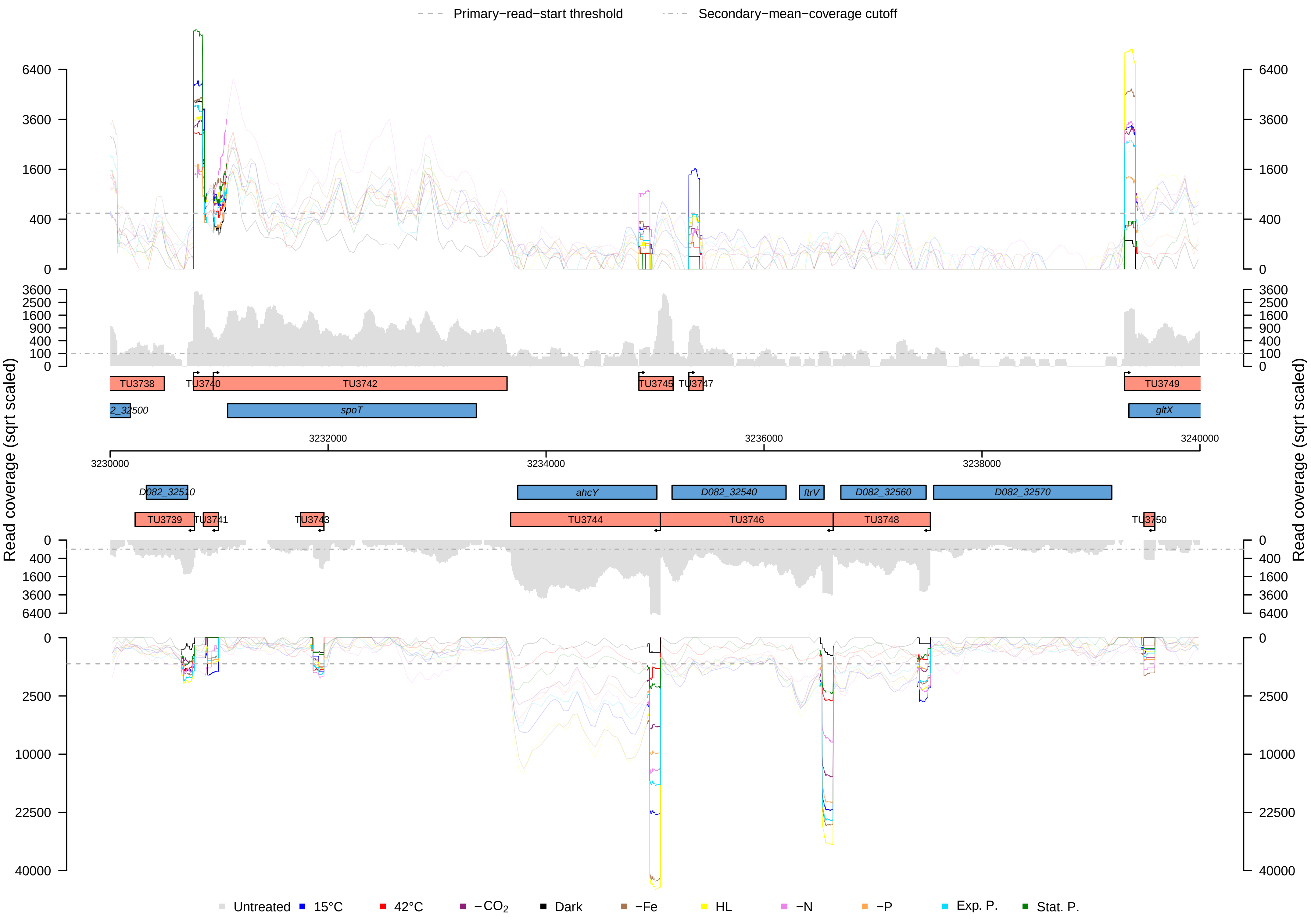

Primary−read−start threshold
Secondary−mean−coverage cutoff
6400
3600
1600
400
0
3600
2500
1600
900
400
100
0
Read coverage (sqrt scaled)
TU3738
TU3740
TU3742
TU3745
TU3747
TU3749
2_32500
spoT
gltX
3232000
3236000
3240000
3230000
3234000
3238000
D082_32510
ahcY
D082_32540
ftrV
D082_32560
D082_32570
TU3739
TU3741
TU3743
TU3744
TU3746
TU3748
TU3750
2500
10000
22500
40000
Untreated
15°C
42°C
−CO2
Dark
−Fe
HL
−N
−P
Exp. P.
Stat. P.

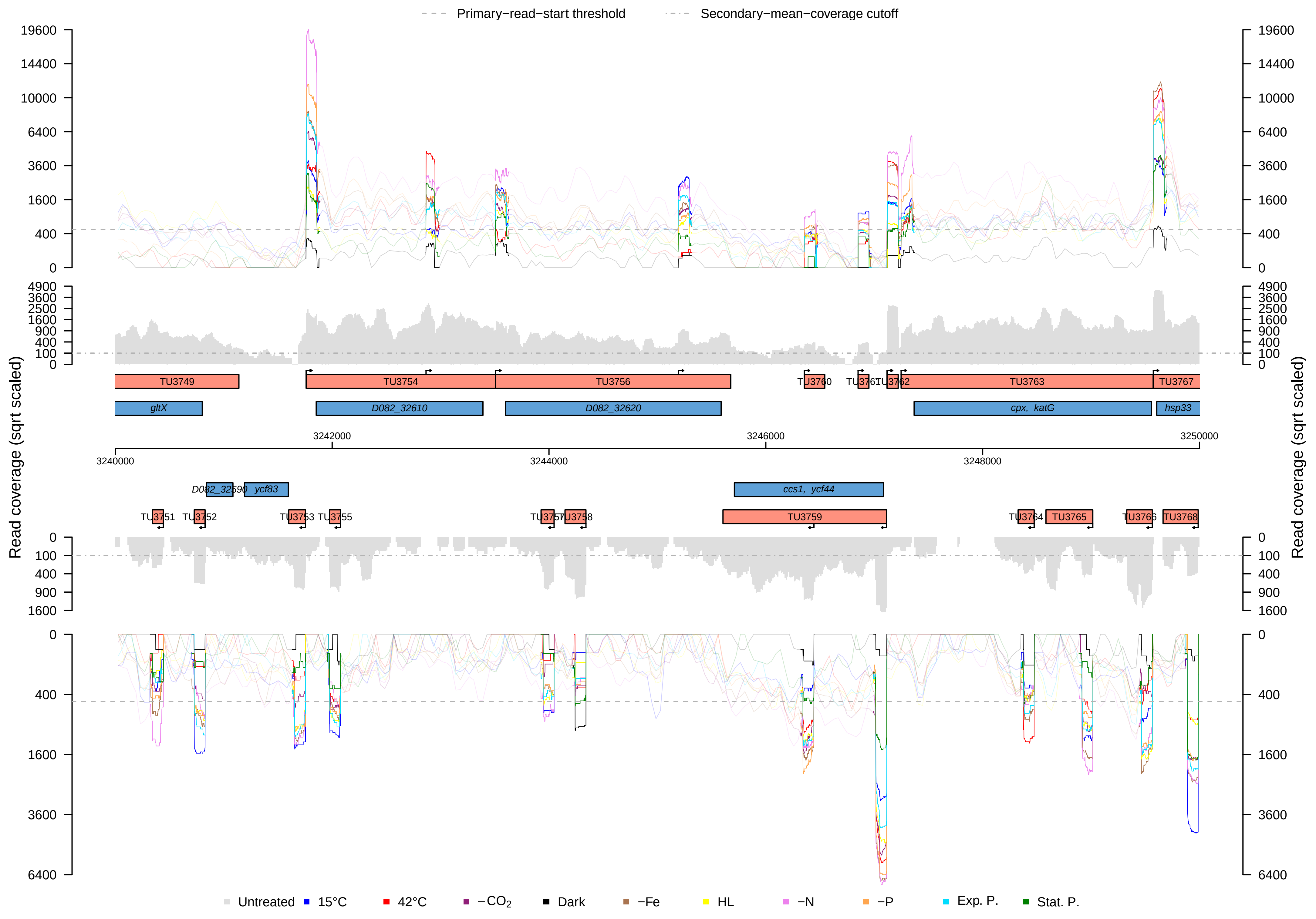

Primary−read−start threshold
Secondary−mean−coverage cutoff
Read coverage (sqrt scaled)
19600
14400
10000
6400
3600
1600
400
0
4900
2500
900
100
TU3749
TU3754
TU3756
TU3760
TU3761
TU3762
TU3763
TU3767
gltX
D082_32610
D082_32620
cpx, katG
hsp33
3240000
3242000
3244000
3246000
3248000
3250000
D082_32590
ycf83
ccs1, ycf44
TU3751
TU3752
TU3753
TU3755
TU3757
TU3758
TU3759
TU3764
TU3765
TU3766
TU3768
Untreated
15°C
42°C
−CO2
Dark
−Fe
HL
−N
−P
Exp. P.
Stat. P.

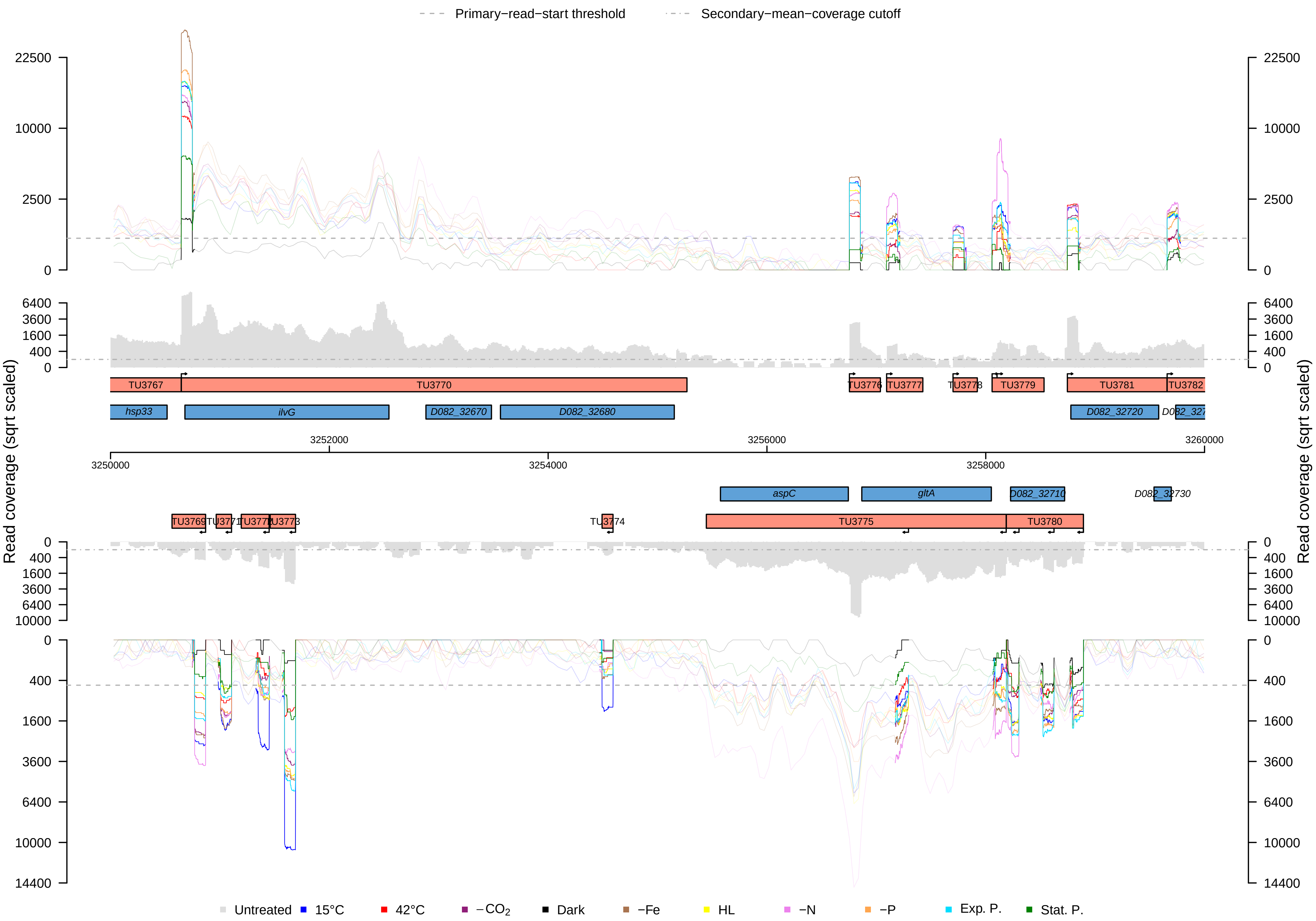

Primary−read−start threshold
Secondary−mean−coverage cutoff
Read coverage (sqrt scaled)
22500
10000
2500
0
6400
3600
1600
400
TU3767
TU3770
TU3776
TU3777
TU3778
TU3779
TU3781
TU3782
hsp33
ilvG
D082_32670
D082_32680
D082_32720
3250000
3252000
3254000
3256000
3258000
3260000
aspC
gltA
D082_32710
D082_32730
TU3769
TU3771
TU3772
TU3773
TU3774
TU3775
TU3780
14400
Untreated
15°C
42°C
−CO_2
Dark
−Fe
HL
−N
−P
Exp. P.
Stat. P.

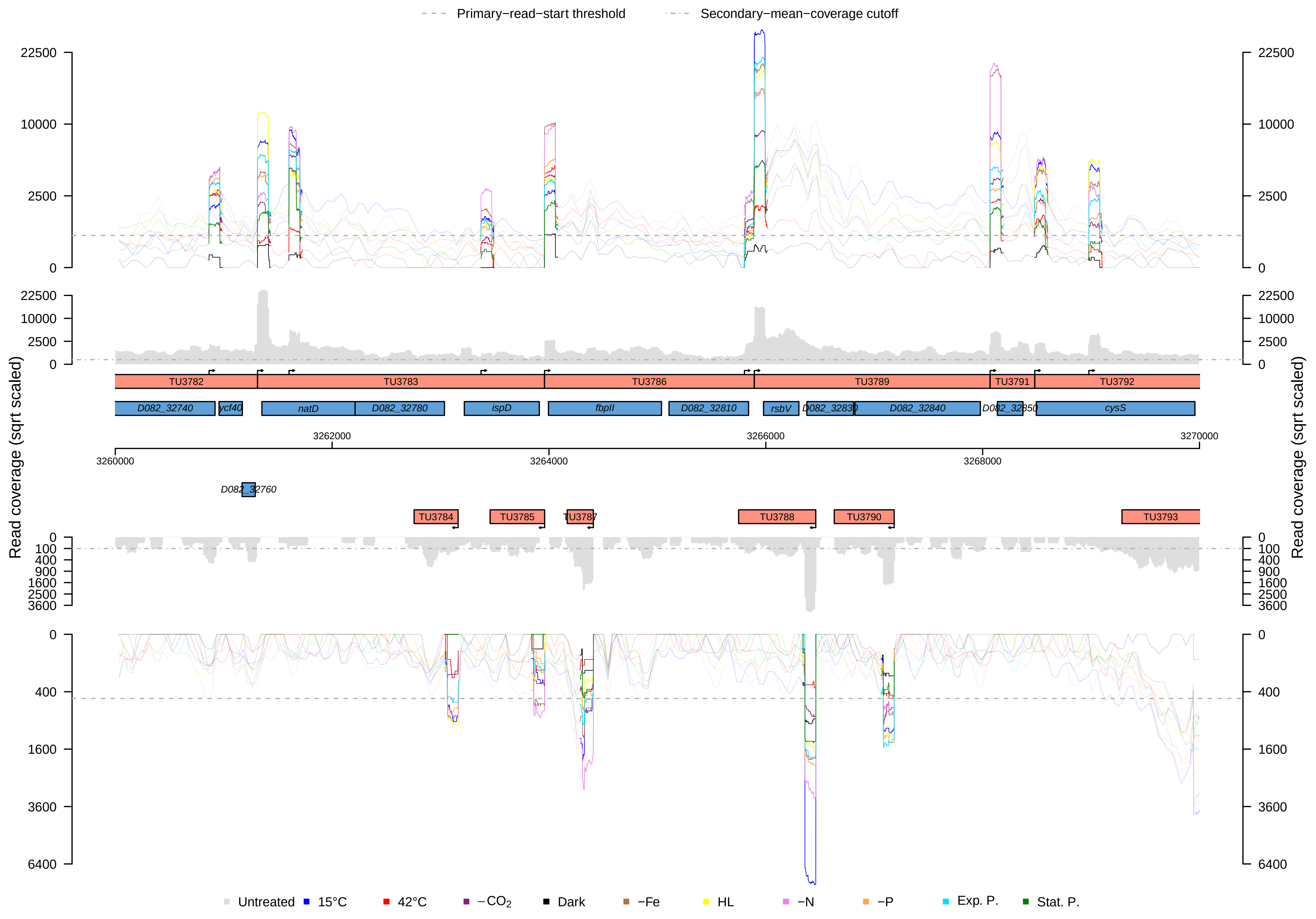

Primary−read−start threshold
Secondary−mean−coverage cutoff
Read coverage (sqrt scaled)
TU3782
TU3783
TU3786
TU3789
TU3791
TU3792
D082_32740
ycf40
natD
D082_32780
ispD
fbpII
D082_32810
rsbV
D082_32830
D082_32840
D082_32850
cysS
D082_32760
TU3784
TU3785
TU3787
TU3788
TU3790
TU3793
3260000
3262000
3264000
3266000
3268000
3270000
Untreated
15°C
42°C
$-CO_2$
Dark
−Fe
HL
−N
−P
Exp. P.
Stat. P.

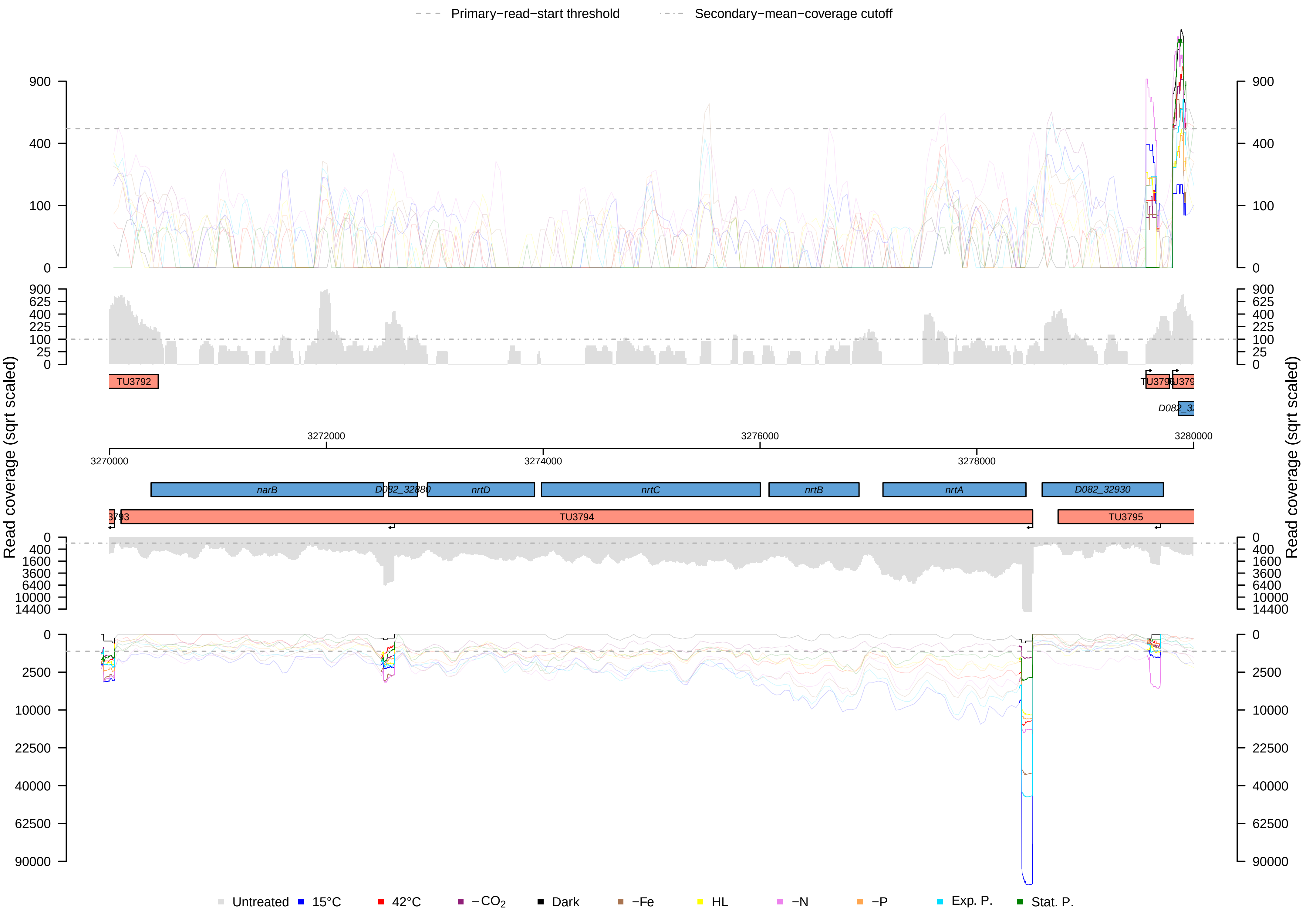

Primary−read−start threshold
Secondary−mean−coverage cutoff
Read coverage (sqrt scaled)
900
400
100
0
625
225
25
TU3792
TU3796
D082_32
3272000
3276000
3280000
3270000
3274000
3278000
narB
D082_32880
nrtD
nrtC
nrtB
nrtA
D082_32930
TU3794
TU3795
1600
3600
6400
10000
14400
2500
22500
40000
62500
90000
Untreated
15°C
42°C
−CO2
Dark
−Fe
HL
−N
−P
Exp. P.
Stat. P.

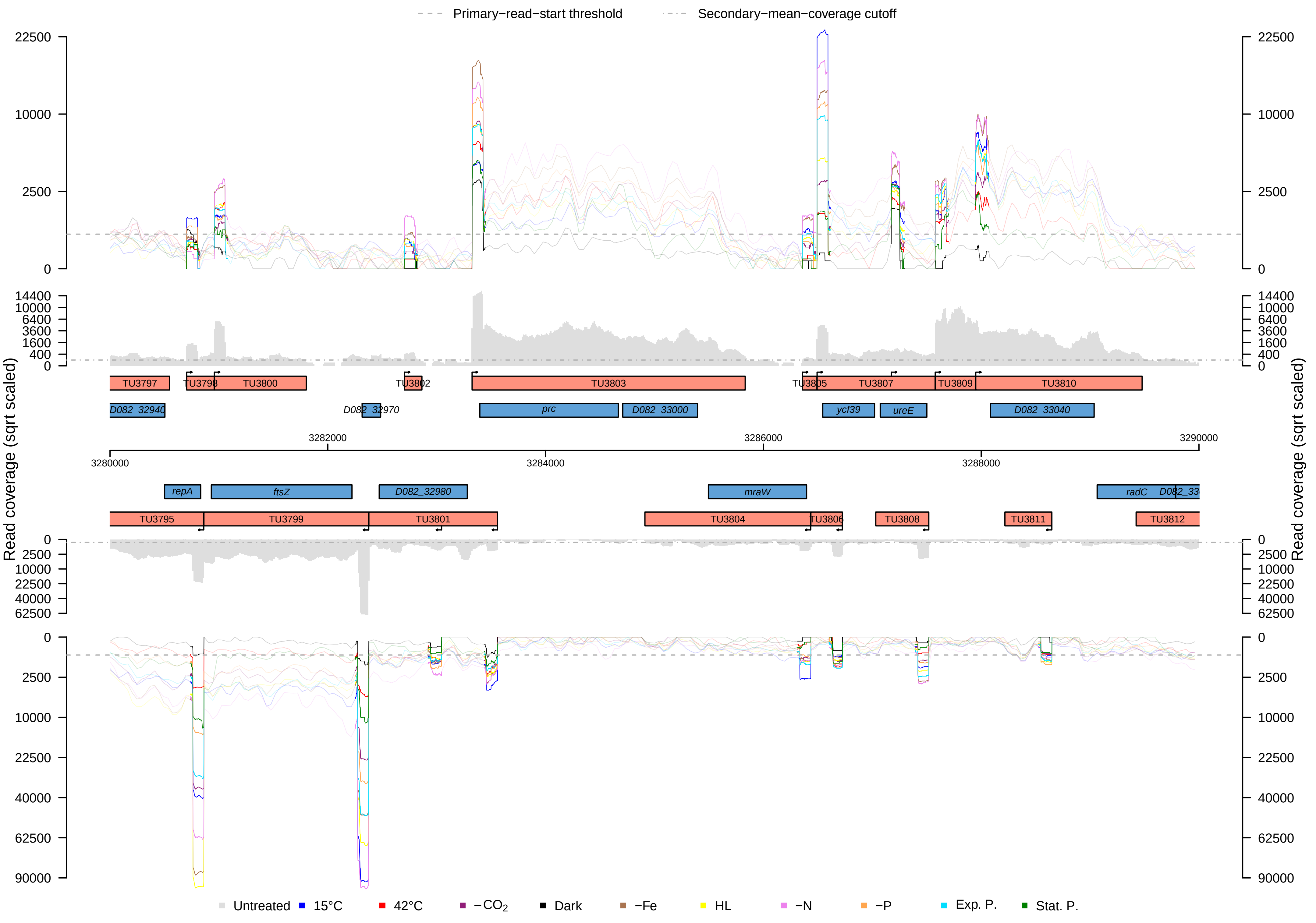

Primary−read−start threshold
Secondary−mean−coverage cutoff
Read coverage (sqrt scaled)
TU3797
TU3798
TU3800
TU3802
TU3803
TU3805
TU3807
TU3809
TU3810
D082_32940
D082_32970
prc
D082_33000
ycf39
ureE
D082_33040
3280000
3282000
3284000
3286000
3288000
3290000
repA
ftsZ
D082_32980
mraW
radC
TU3795
TU3799
TU3801
TU3804
TU3806
TU3808
TU3811
TU3812
Untreated
15°C
42°C
−CO_2
Dark
−Fe
HL
−N
−P
Exp. P.
Stat. P.

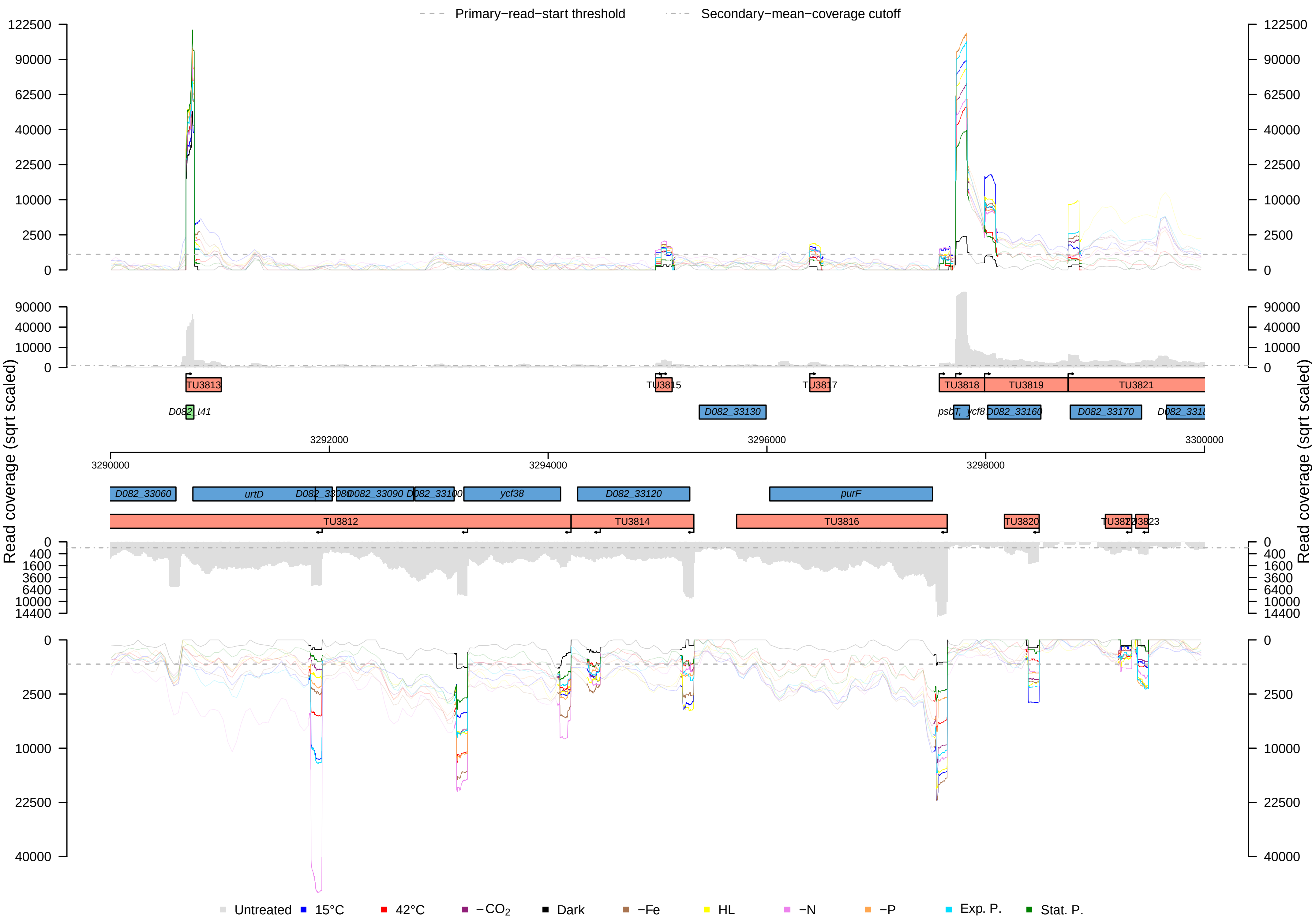

Primary−read−start threshold
Secondary−mean−coverage cutoff
Read coverage (sqrt scaled)
122500
90000
62500
40000
22500
10000
2500
0
40000
10000
400
1600
3600
6400
14400
TU3813
TU3815
TU3817
TU3818
TU3819
TU3821
D082_t41
D082_33130
psbT, ycf8
D082_33160
D082_33170
3290000
3292000
3294000
3296000
3298000
3300000
D082_33060
urtD
D082_33090
ycf38
D082_33120
purF
TU3812
TU3814
TU3816
TU3820
Untreated
15°C
42°C
−CO2
Dark
−Fe
HL
−N
−P
Exp. P.
Stat. P.

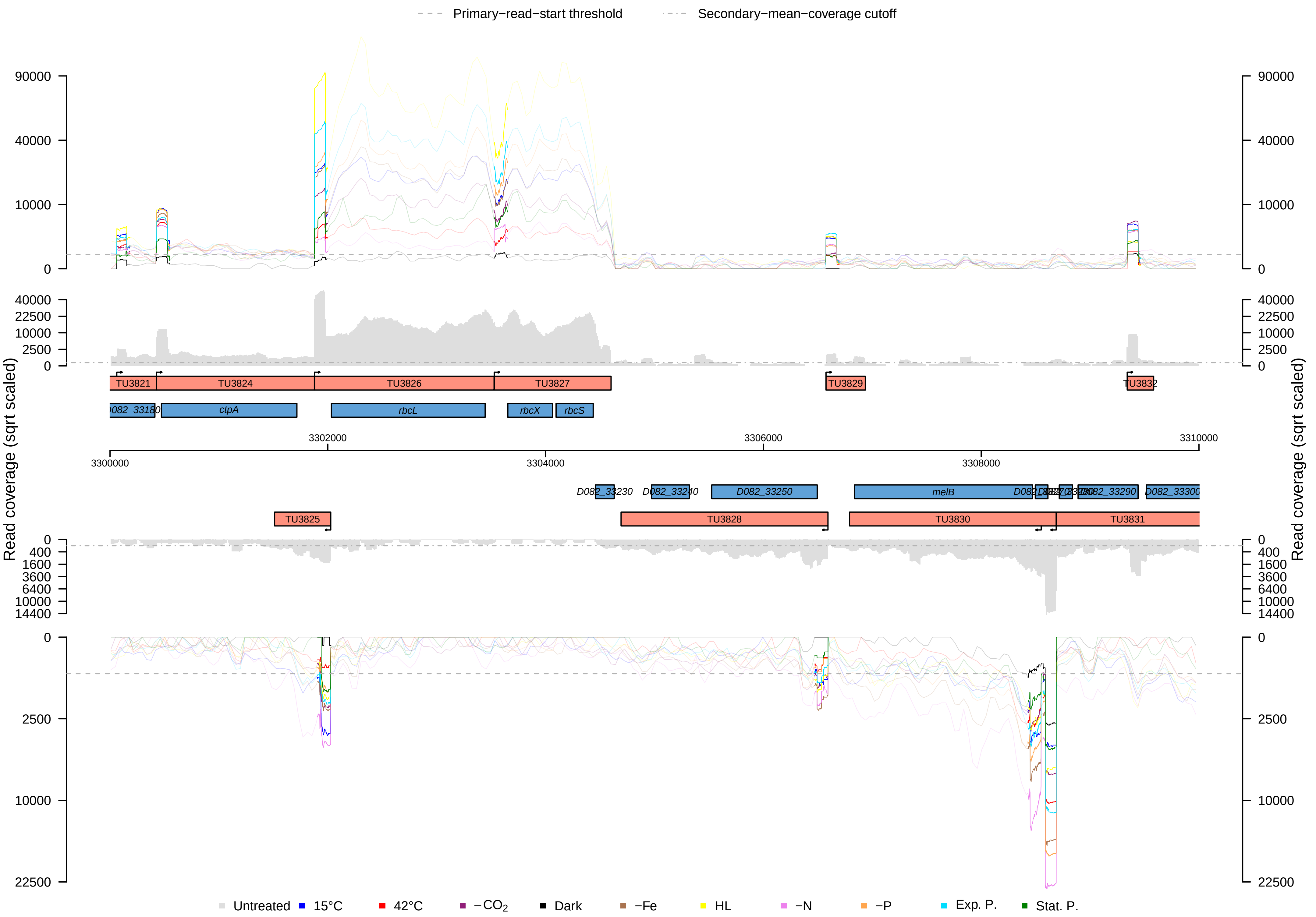

Primary−read−start threshold
Secondary−mean−coverage cutoff
Read coverage (sqrt scaled)
TU3821
TU3824
TU3826
TU3827
TU3829
TU3832
ctpA
rbcL
rbcX
rbcS
3300000
3302000
3304000
3306000
3308000
3310000
D082_33230
D082_33240
D082_33250
melB
D082_33290
TU3825
TU3828
TU3830
TU3831
Untreated
15°C
42°C
−CO2
Dark
−Fe
HL
−N
−P
Exp. P.
Stat. P.

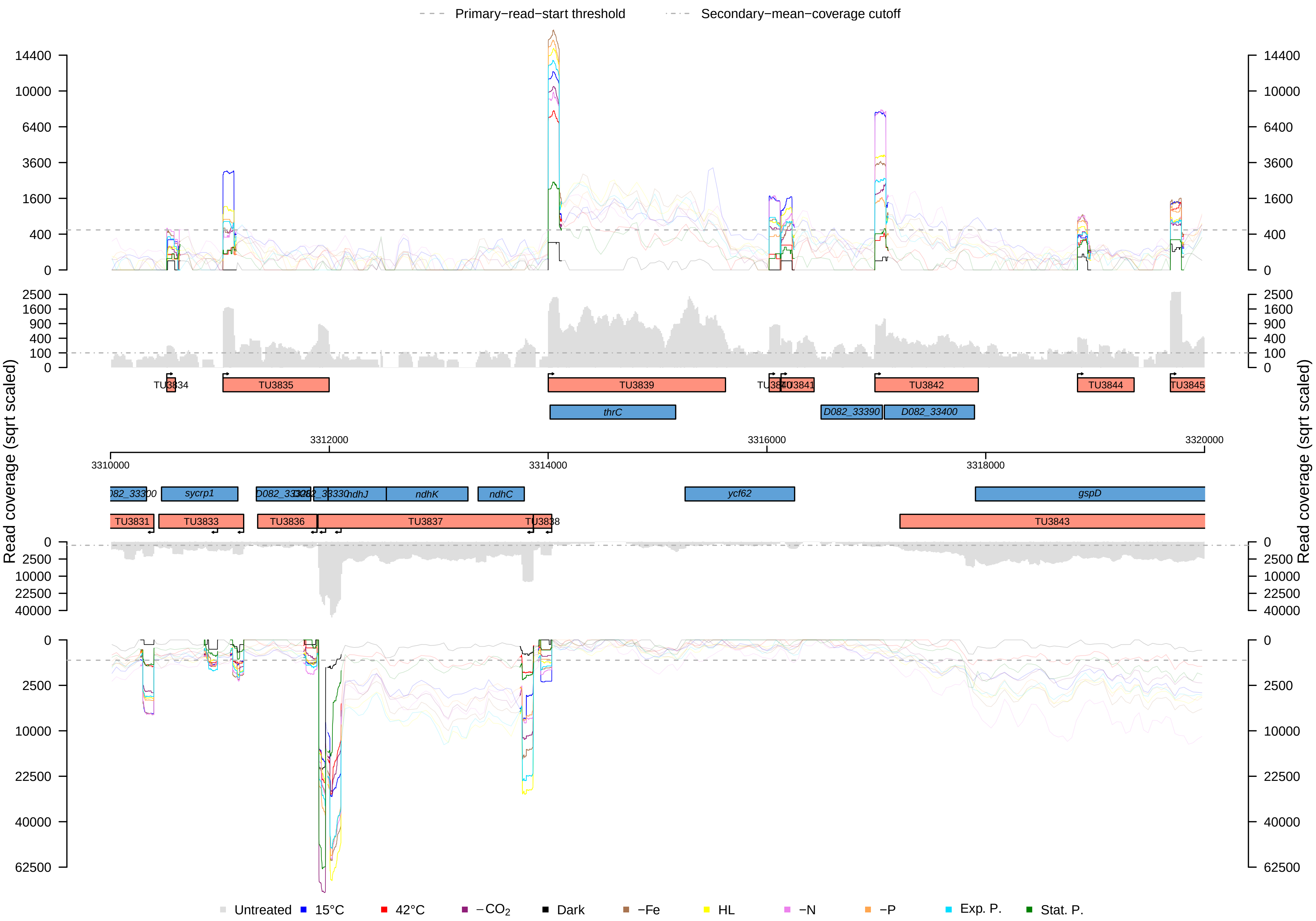

Primary−read−start threshold
Secondary−mean−coverage cutoff
Read coverage (sqrt scaled)
TU3834
TU3835
TU3839
TU3841
TU3842
TU3844
TU3845
thrC
D082_33390
D082_33400
3310000
3312000
3314000
3316000
3318000
3320000
sycrp1
ndhJ
ndhK
ndhC
ycf62
gspD
TU3831
TU3833
TU3836
TU3837
TU3843
Untreated
15°C
42°C
−CO2
Dark
−Fe
HL
−N
−P
Exp. P.
Stat. P.

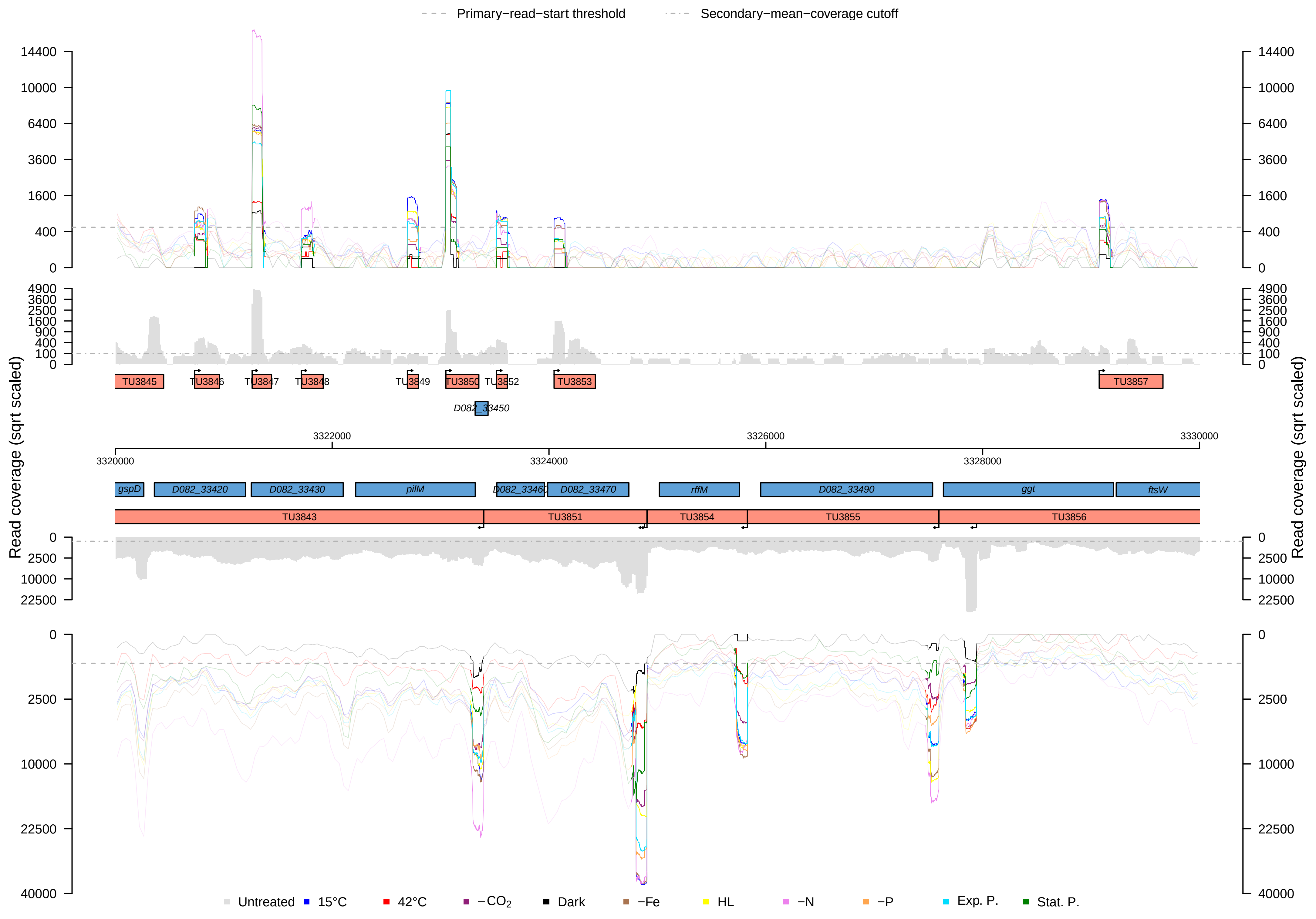

Primary−read−start threshold
Secondary−mean−coverage cutoff
Read coverage (sqrt scaled)
14400
10000
6400
3600
1600
400
0
4900
3600
2500
1600
900
400
100
0
TU3845
TU3846
TU3847
TU3848
TU3849
TU3850
TU3852
TU3853
TU3857
D082_33450
3320000
3322000
3324000
3326000
3328000
3330000
gspD
D082_33420
D082_33430
pilM
D082_33460
D082_33470
rffM
D082_33490
ggt
ftsW
TU3843
TU3851
TU3854
TU3855
TU3856
2500
10000
22500
40000
Untreated
15°C
42°C
−CO2
Dark
−Fe
HL
−N
−P
Exp. P.
Stat. P.

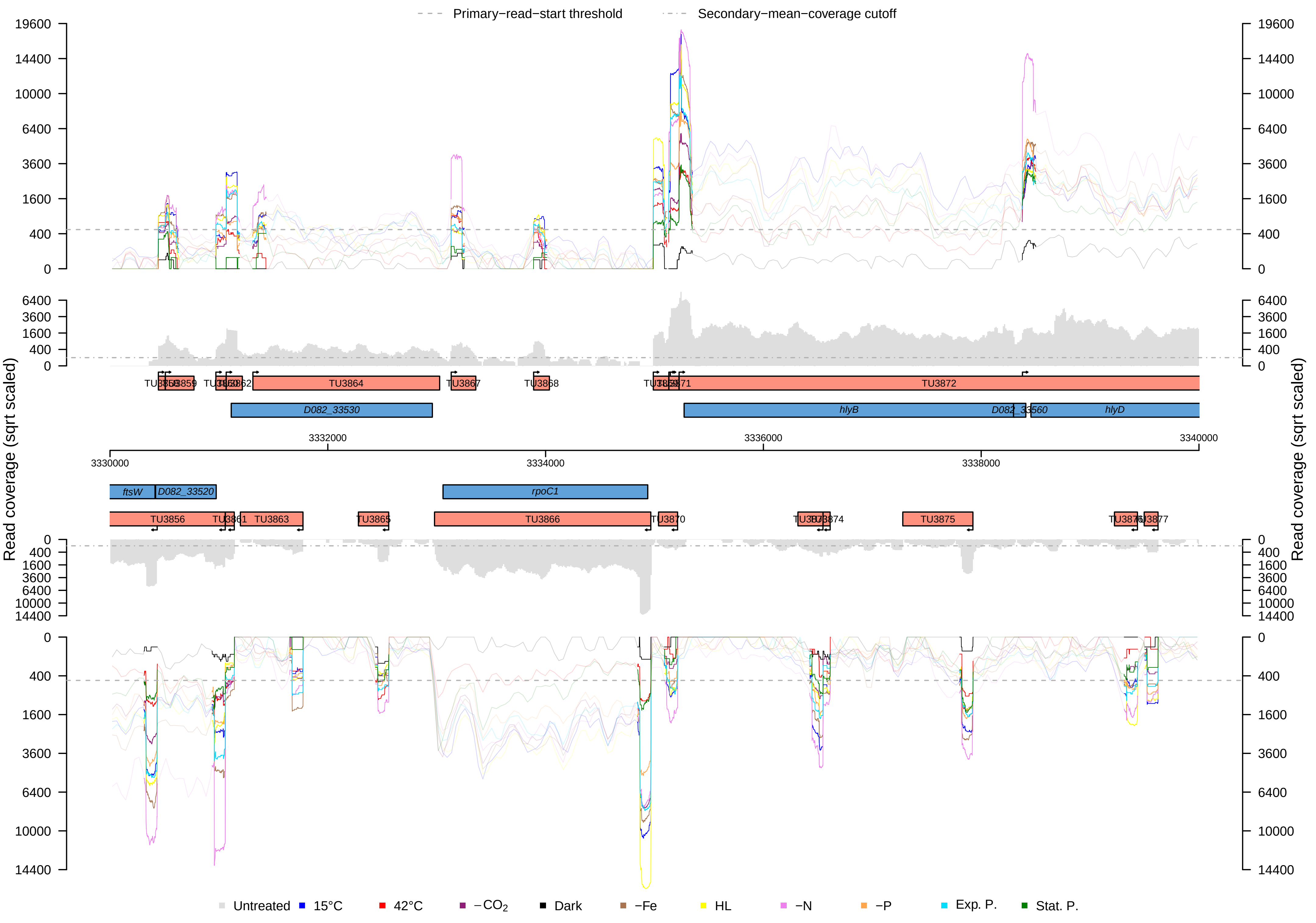

Primary−read−start threshold
Secondary−mean−coverage cutoff
Read coverage (sqrt scaled)
19600
14400
10000
6400
3600
1600
400
0
TU3864
TU3867
TU3872
TU3856
TU3863
TU3865
TU3866
TU3870
TU3875
D082_33530
hlyB
hlyD
ftsW
D082_33520
rpoC1
3330000
3332000
3334000
3336000
3338000
3340000
Untreated
15°C
42°C
−CO2
Dark
−Fe
HL
−N
−P
Exp. P.
Stat. P.

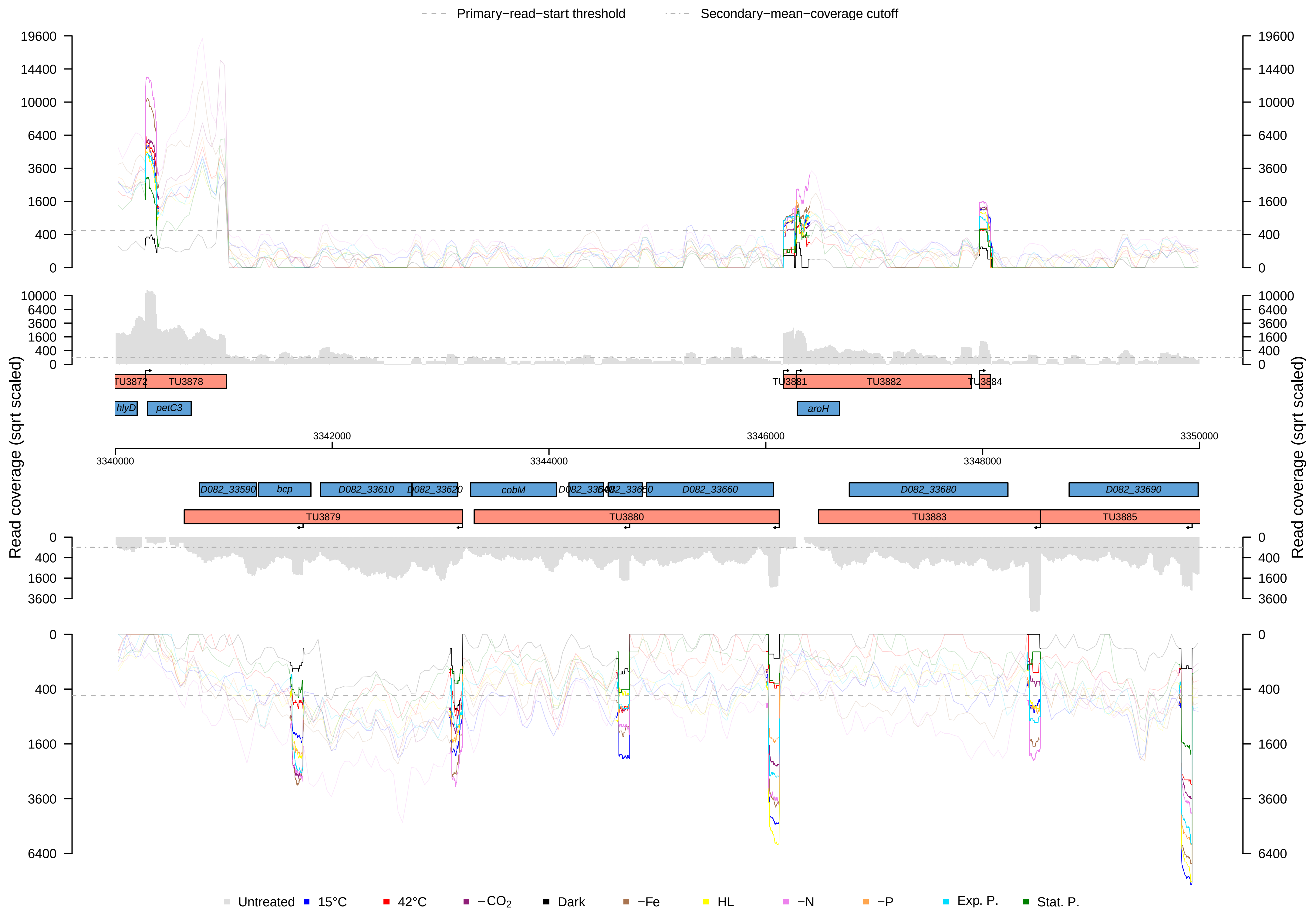

Primary−read−start threshold
Secondary−mean−coverage cutoff
Read coverage (sqrt scaled)
19600
14400
10000
6400
3600
1600
400
0
TU3872
TU3878
TU3881
TU3882
TU3884
hlyD
petC3
aroH
3340000
3342000
3344000
3346000
3348000
3350000
D082_33590
bcp
D082_33610
D082_33620
cobM
D082_33650
D082_33660
D082_33680
D082_33690
TU3879
TU3880
TU3883
TU3885
Untreated
15°C
42°C
−CO2
Dark
−Fe
HL
−N
−P
Exp. P.
Stat. P.

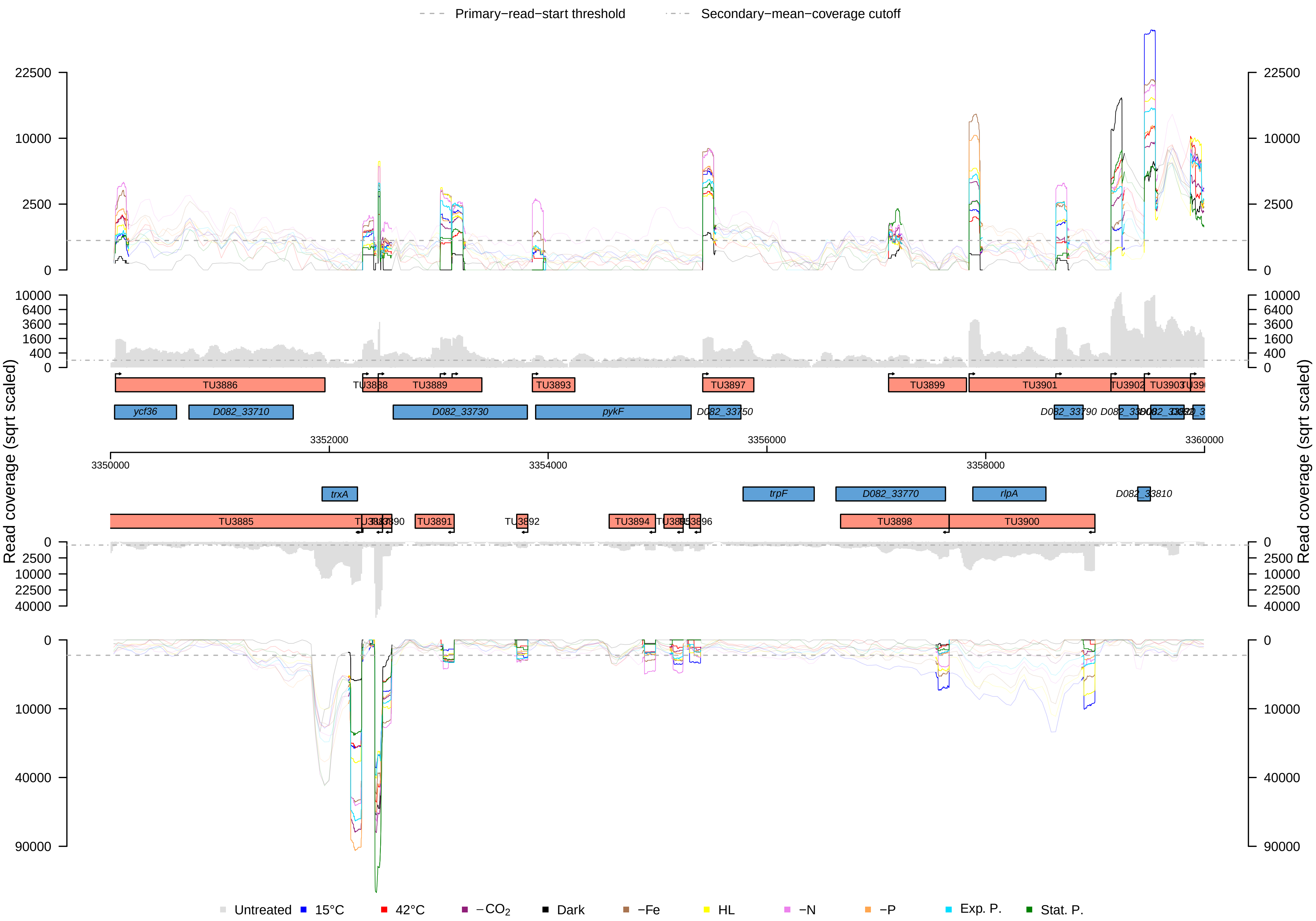

Primary−read−start threshold
Secondary−mean−coverage cutoff
Read coverage (sqrt scaled)
22500
10000
2500
0
6400
3600
1600
400
40000
90000
TU3886
TU3888
TU3889
TU3893
TU3897
TU3899
TU3901
TU3902
TU3903
ycf36
D082_33710
D082_33730
pykF
D082_33750
D082_33790
3350000
3352000
3354000
3356000
3358000
3360000
trxA
trpF
D082_33770
rlpA
D082_33810
TU3885
TU3891
TU3892
TU3894
TU3896
TU3898
TU3900
Untreated
15°C
42°C
−CO2
Dark
−Fe
HL
−N
−P
Exp. P.
Stat. P.

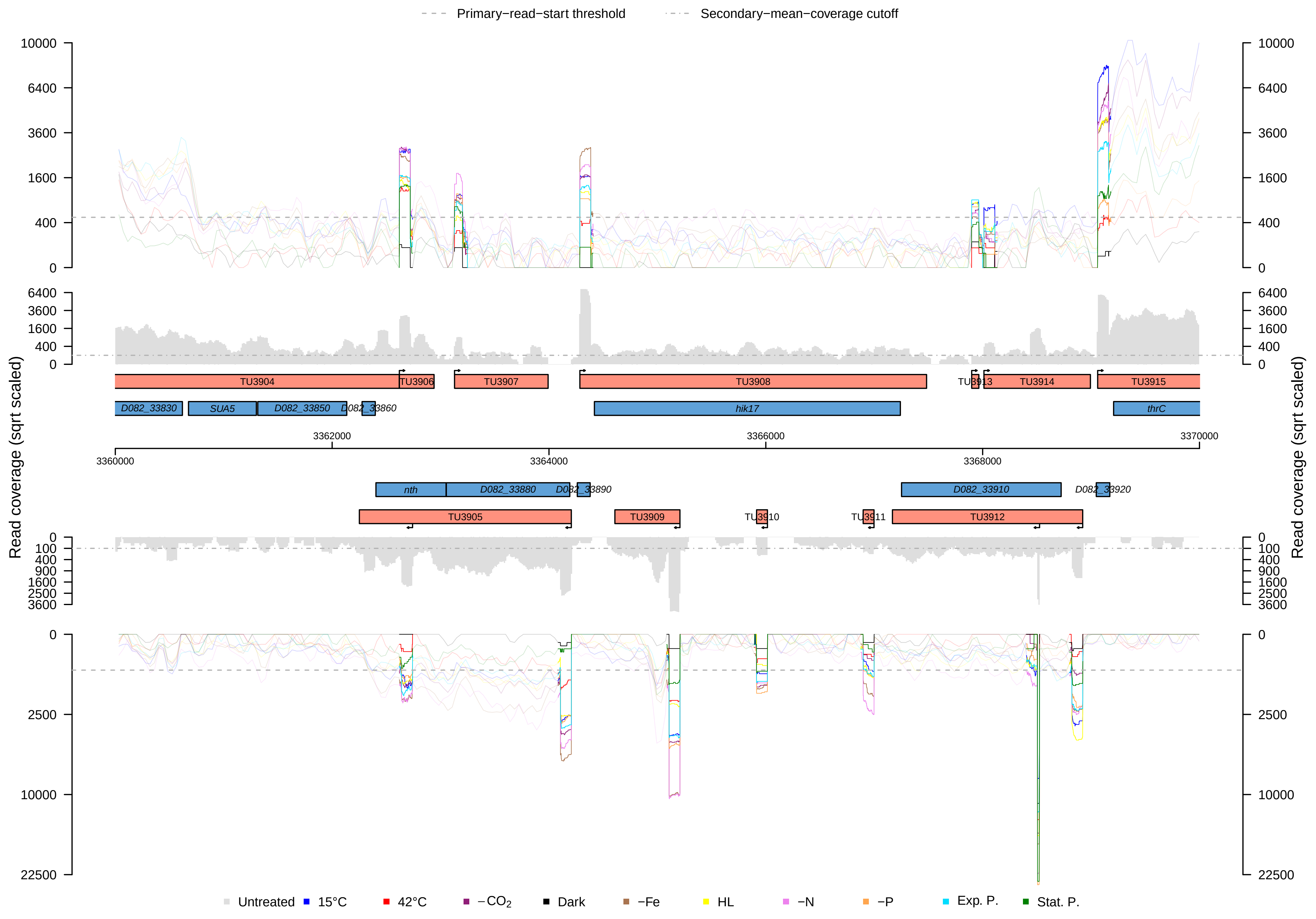

Primary−read−start threshold
Secondary−mean−coverage cutoff
Read coverage (sqrt scaled)
TU3904
TU3906
TU3907
TU3908
TU3913
TU3914
TU3915
D082_33830
SUA5
D082_33850
D082_33860
hik17
thrC
3360000
3362000
3364000
3366000
3368000
3370000
nth
D082_33880
D082_33890
D082_33910
D082_33920
TU3905
TU3909
TU3910
TU3911
TU3912
Untreated
15°C
42°C
−CO2
Dark
−Fe
HL
−N
−P
Exp. P.
Stat. P.

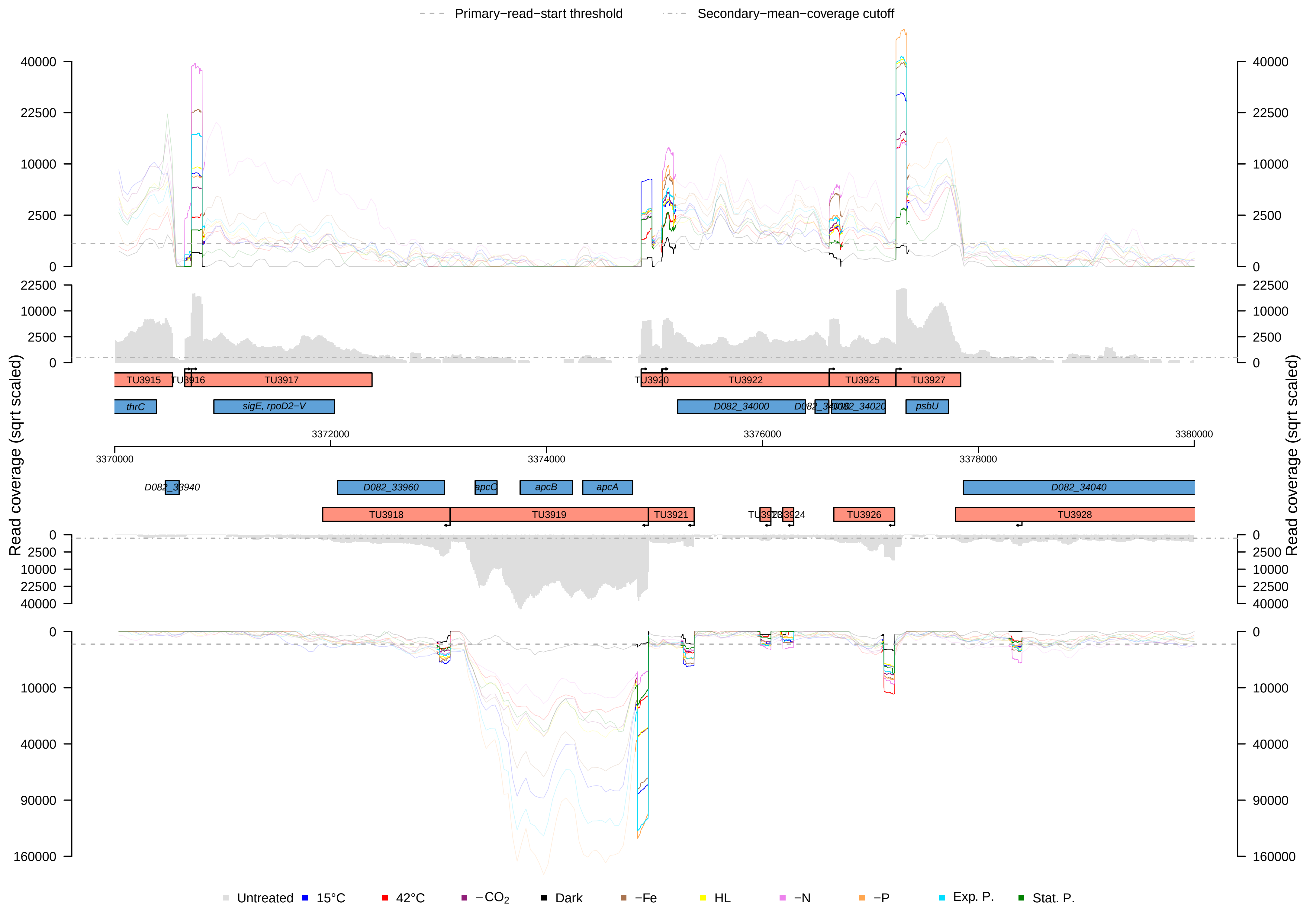

Primary−read−start threshold
Secondary−mean−coverage cutoff
Read coverage (sqrt scaled)
40000
22500
10000
2500
0
TU3915
TU3916
TU3917
TU3920
TU3922
TU3925
TU3927
thrC
sigE, rpoD2−V
D082_34000
D082_34010
D082_34020
psbU
3370000
3372000
3374000
3376000
3378000
3380000
D082_33940
D082_33960
apcC
apcB
apcA
D082_34040
TU3918
TU3919
TU3921
TU3923
TU3924
TU3926
TU3928
90000
160000
Untreated
15°C
42°C
−CO2
Dark
−Fe
HL
−N
−P
Exp. P.
Stat. P.

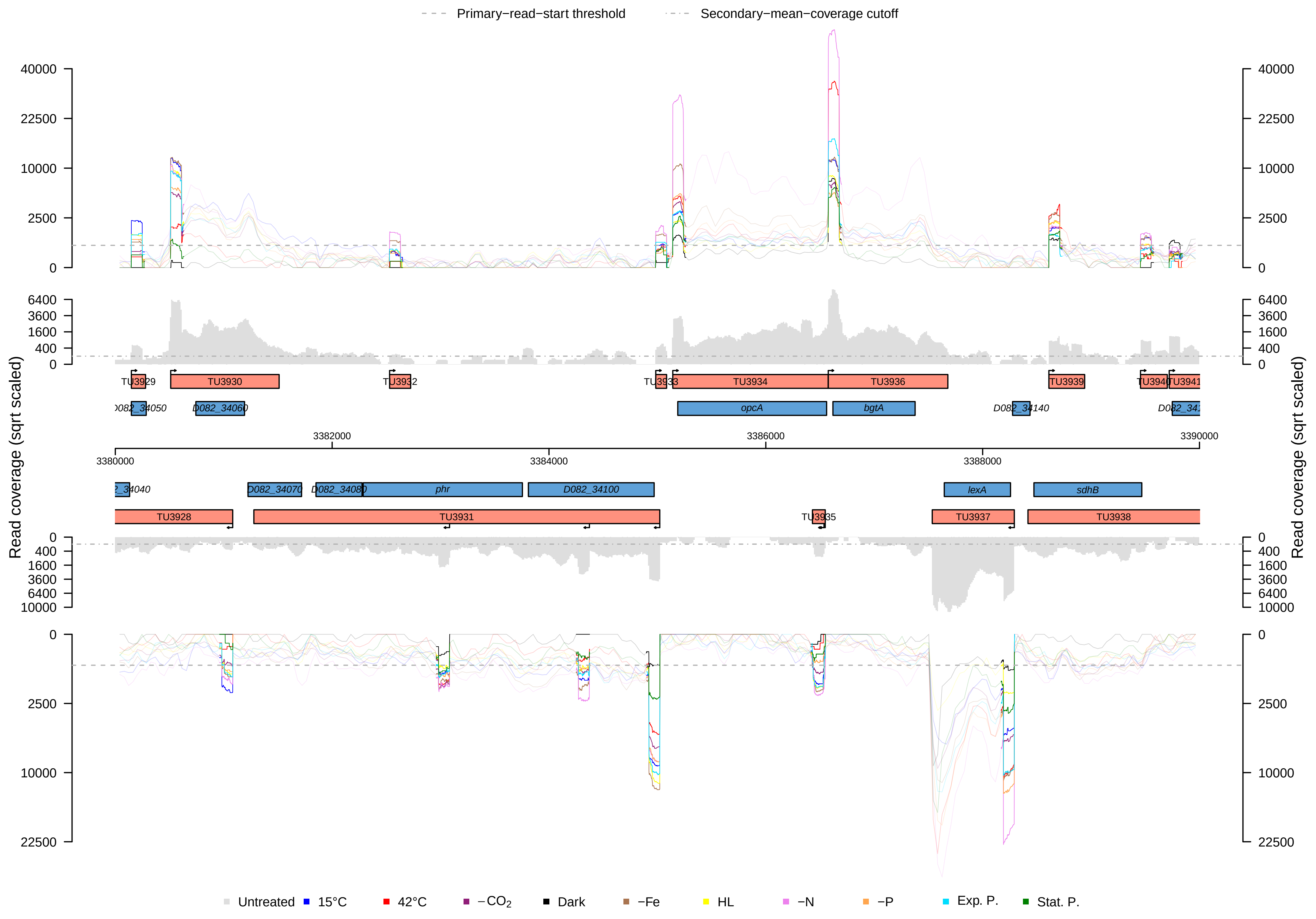

Primary−read−start threshold
Secondary−mean−coverage cutoff
40000
22500
10000
2500
0
6400
3600
1600
400
Read coverage (sqrt scaled)
TU3929
TU3930
TU3932
TU3933
TU3934
TU3936
TU3939
TU3940
TU3941
D082_34050
D082_34060
opcA
bgtA
D082_34140
3382000
3386000
3390000
3380000
3384000
3388000
D082_34070
D082_34080
phr
D082_34100
lexA
sdhB
TU3928
TU3931
TU3935
TU3937
TU3938
Untreated
15°C
42°C
−CO2
Dark
−Fe
HL
−N
−P
Exp. P.
Stat. P.

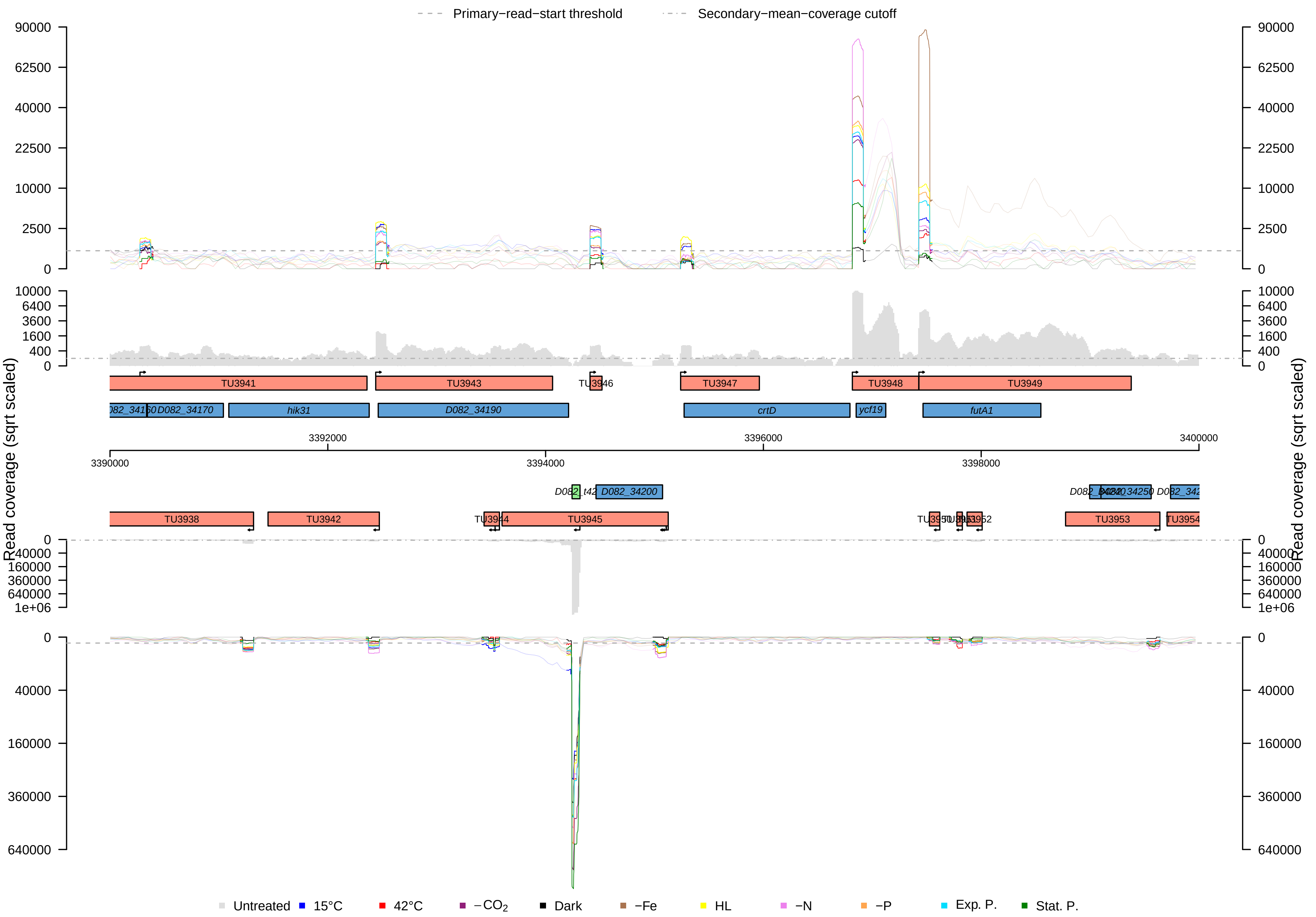

Primary−read−start threshold
Secondary−mean−coverage cutoff
Read coverage (sqrt scaled)
TU3941
TU3943
TU3946
TU3947
TU3948
TU3949
D082_34170
hik31
D082_34190
crtD
ycf19
futA1
3390000
3392000
3394000
3396000
3398000
3400000
D082_34200
TU3938
TU3942
TU3944
TU3945
TU3953
TU3954
Untreated
15°C
42°C
−CO_2
Dark
−Fe
HL
−N
−P
Exp. P.
Stat. P.

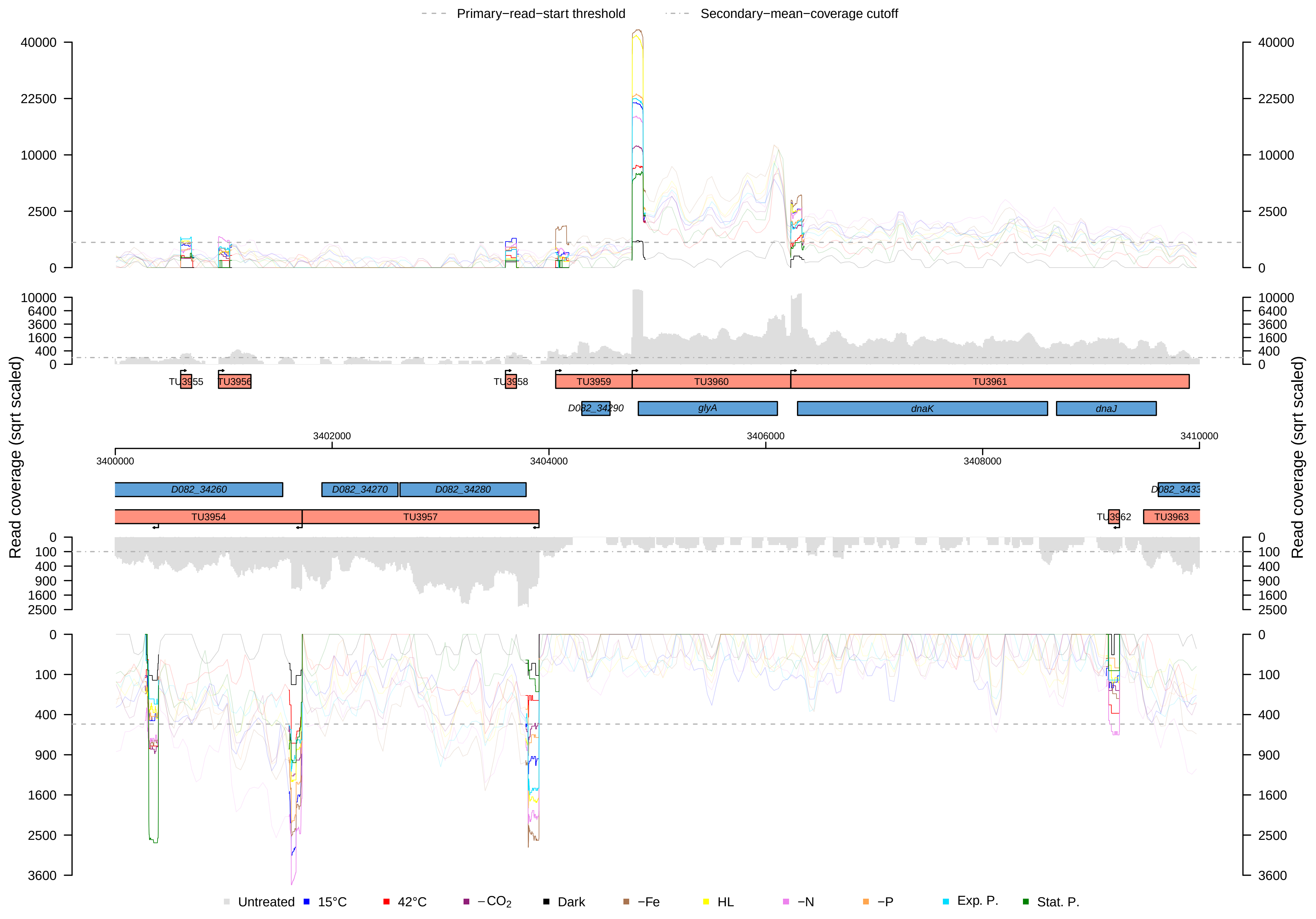

Primary−read−start threshold
Secondary−mean−coverage cutoff
Read coverage (sqrt scaled)
40000
22500
10000
2500
0
10000
6400
3600
1600
400
0
TU3955
TU3956
TU3958
TU3959
TU3960
TU3961
D082_34290
glyA
dnaK
dnaJ
3400000
3402000
3404000
3406000
3408000
3410000
D082_34260
D082_34270
D082_34280
TU3954
TU3957
TU3962
TU3963
100
400
900
1600
2500
3600
Untreated
15°C
42°C
−CO2
Dark
−Fe
HL
−N
−P
Exp. P.
Stat. P.

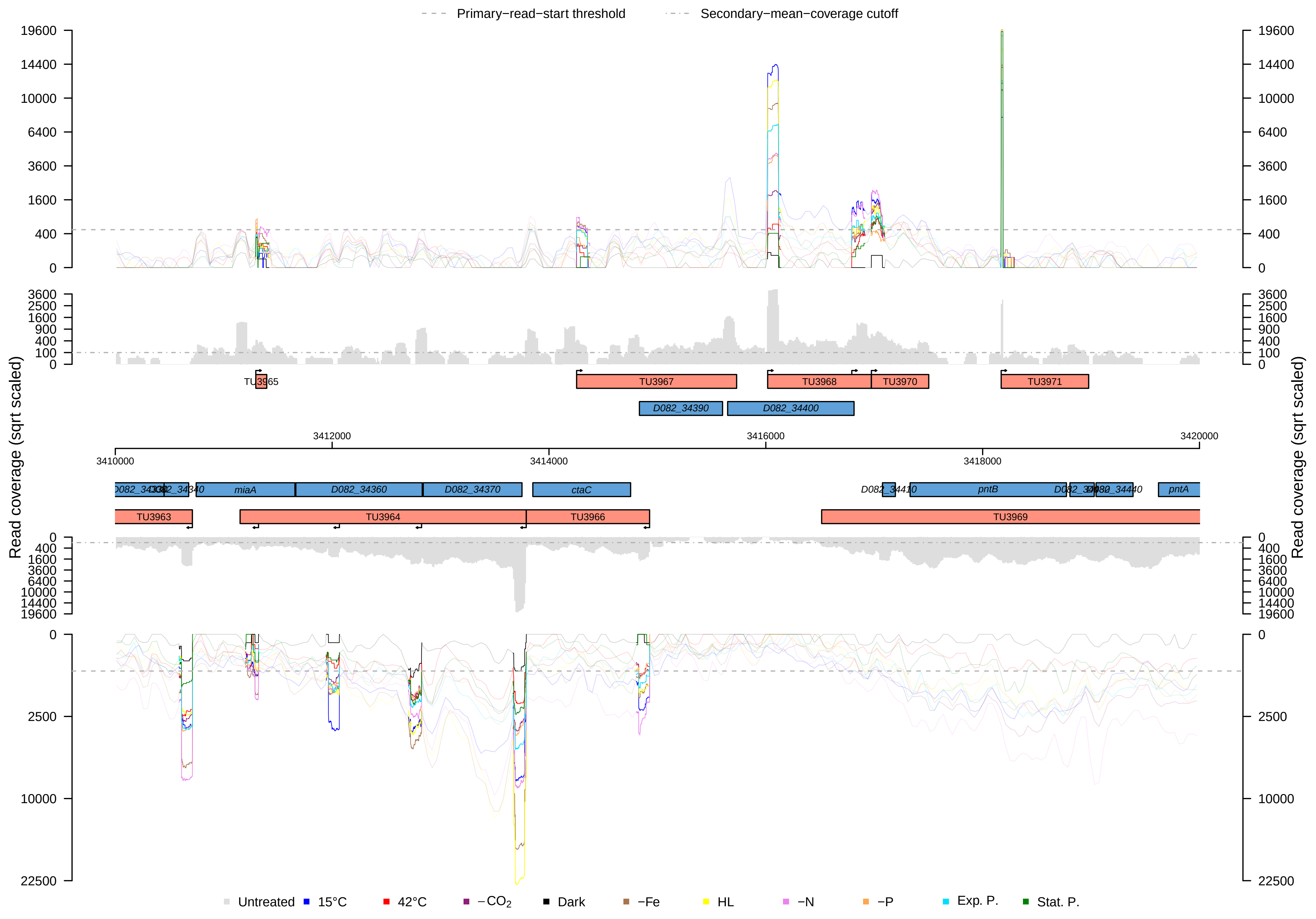

Primary−read−start threshold
Secondary−mean−coverage cutoff
Read coverage (sqrt scaled)
TU3965
TU3967
TU3968
TU3970
TU3971
D082_34390
D082_34400
3410000
3412000
3414000
3416000
3418000
3420000
miaA
D082_34360
D082_34370
ctaC
pntB
pntA
TU3963
TU3964
TU3966
TU3969
Untreated
15°C
42°C
−CO2
Dark
−Fe
HL
−N
−P
Exp. P.
Stat. P.

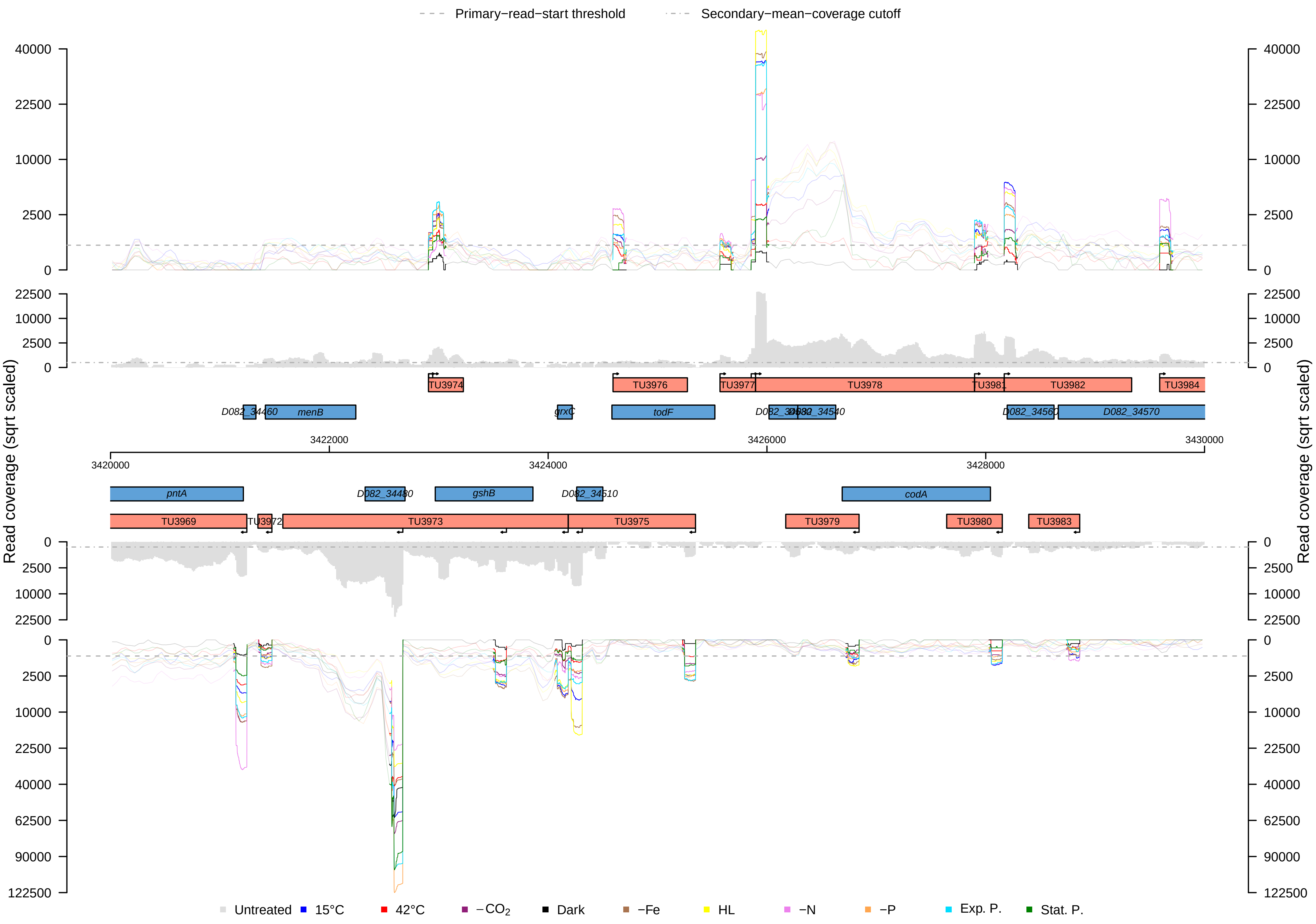

Primary−read−start threshold
Secondary−mean−coverage cutoff
Read coverage (sqrt scaled)
40000
22500
10000
2500
0
TU3974
TU3976
TU3977
TU3978
TU3981
TU3982
TU3984
D082_34460
menB
grxC
todF
D082_34540
D082_34560
D082_34570
3420000
3422000
3424000
3426000
3428000
3430000
pntA
D082_34480
gshB
D082_34510
codA
TU3969
TU3972
TU3973
TU3975
TU3979
TU3980
TU3983
62500
90000
122500
Untreated
15°C
42°C
−CO2
Dark
−Fe
HL
−N
−P
Exp. P.
Stat. P.

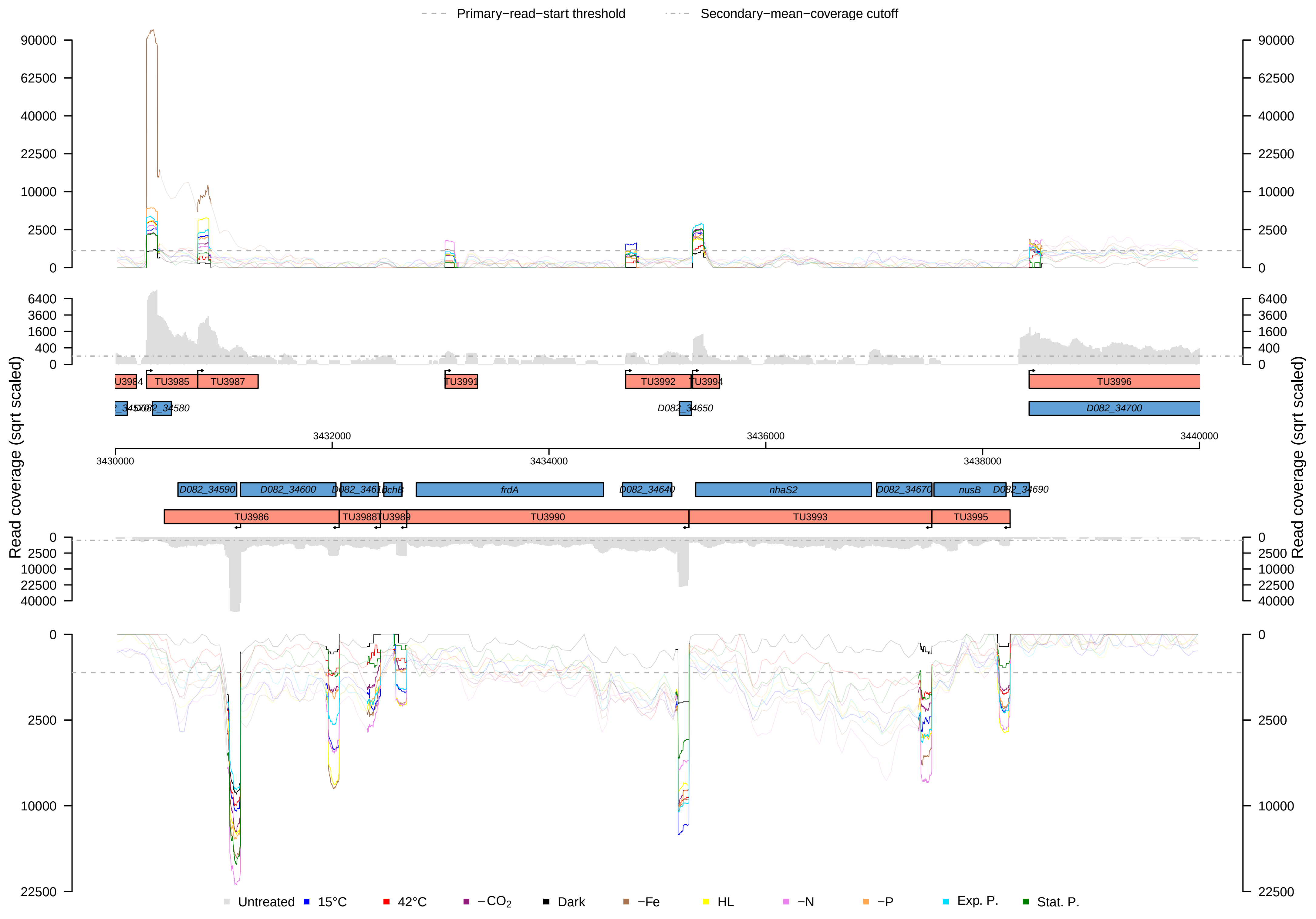

Primary−read−start threshold
Secondary−mean−coverage cutoff
Read coverage (sqrt scaled)
90000
62500
40000
22500
10000
2500
0
6400
3600
1600
400
TU3984
TU3985
TU3987
TU3991
TU3992
TU3994
TU3996
D082_34580
D082_34650
D082_34700
3430000
3432000
3434000
3436000
3438000
3440000
D082_34590
D082_34600
D082_34610
frdA
D082_34640
nhaS2
D082_34670
nusB
D082_34690
TU3986
TU3988
TU3989
TU3990
TU3993
TU3995
Untreated
15°C
42°C
−CO_2
Dark
−Fe
HL
−N
−P
Exp. P.
Stat. P.

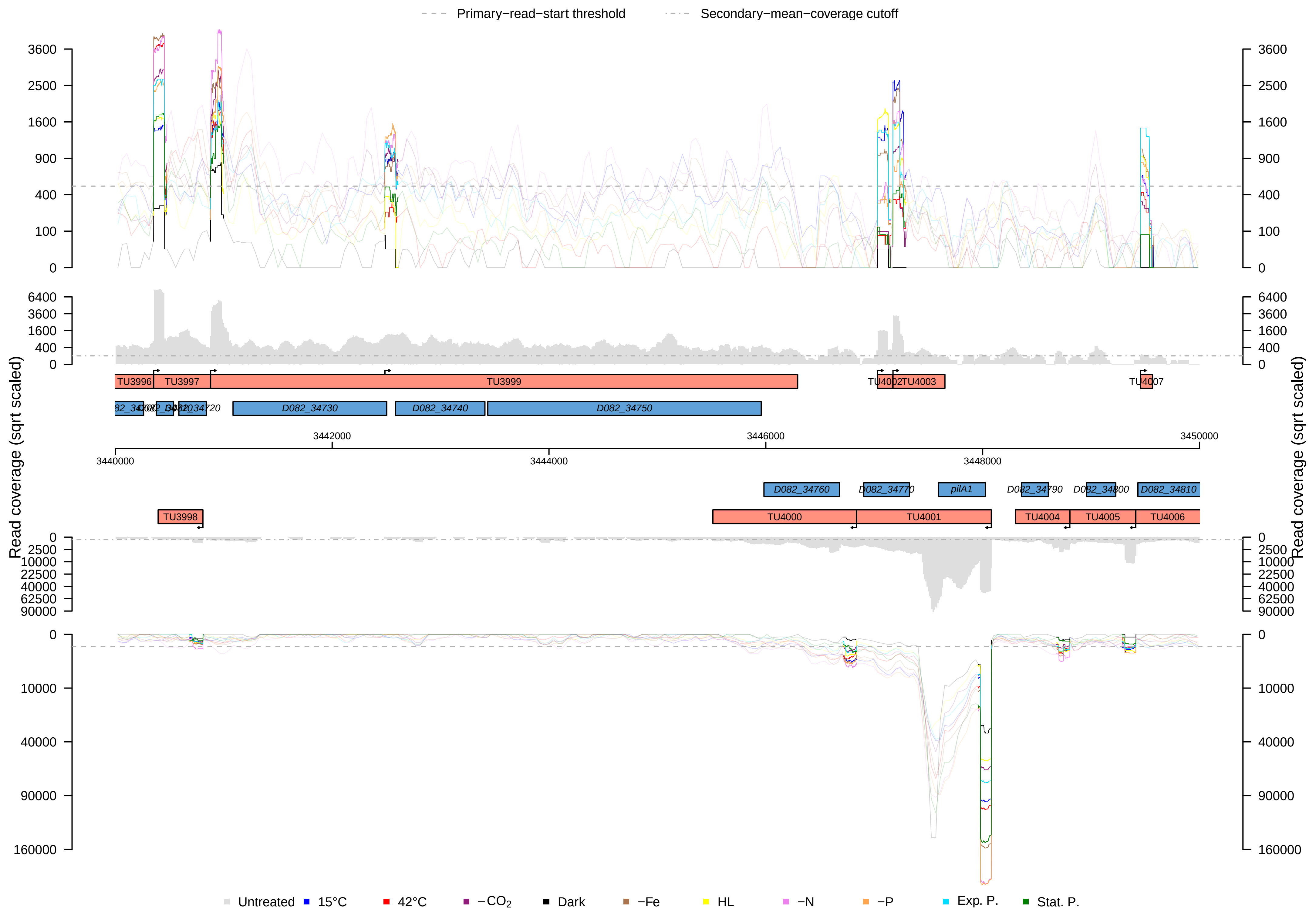

Primary−read−start threshold
Secondary−mean−coverage cutoff
Read coverage (sqrt scaled)
TU3996
TU3997
TU3999
TU4002
TU4003
TU4007
D082_34730
D082_34740
D082_34750
3440000
3442000
3444000
3446000
3448000
3450000
D082_34760
D082_34770
pilA1
D082_34790
D082_34800
D082_34810
TU3998
TU4000
TU4001
TU4004
TU4005
TU4006
Untreated
15°C
42°C
−CO2
Dark
−Fe
HL
−N
−P
Exp. P.
Stat. P.

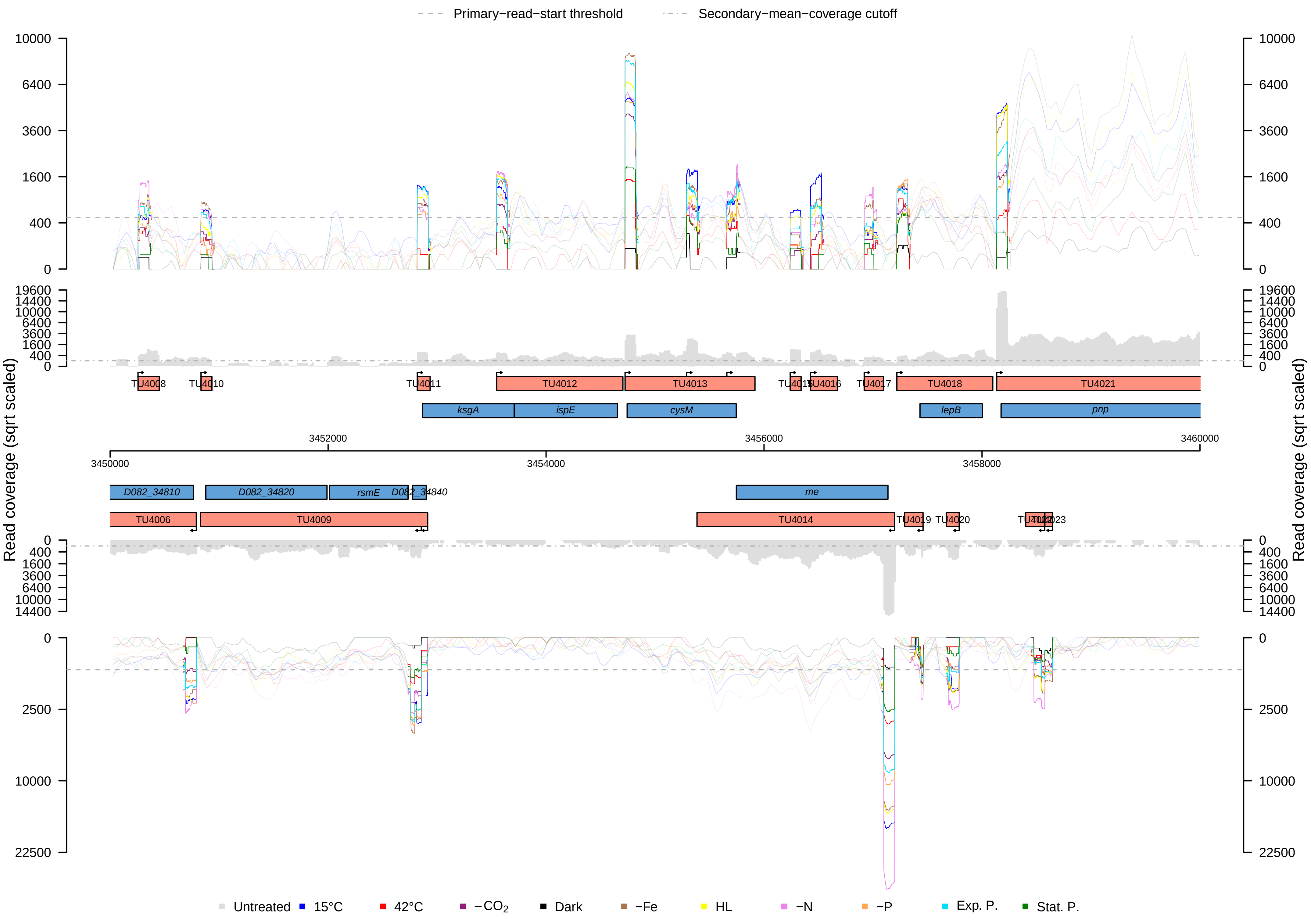

Primary−read−start threshold
Secondary−mean−coverage cutoff
Read coverage (sqrt scaled)
10000
6400
3600
1600
400
0
19600
14400
10000
6400
3600
1600
400
0
TU4008
TU4010
TU4011
TU4012
TU4013
TU4015
TU4016
TU4017
TU4018
TU4021
ksgA
ispE
cysM
lepB
pnp
3450000
3452000
3454000
3456000
3458000
3460000
D082_34810
D082_34820
rsmE
D082_34840
me
TU4006
TU4009
TU4014
TU4019
TU4020
TU4022
TU4023
2500
10000
22500
Untreated
15°C
42°C
−CO2
Dark
−Fe
HL
−N
−P
Exp. P.
Stat. P.

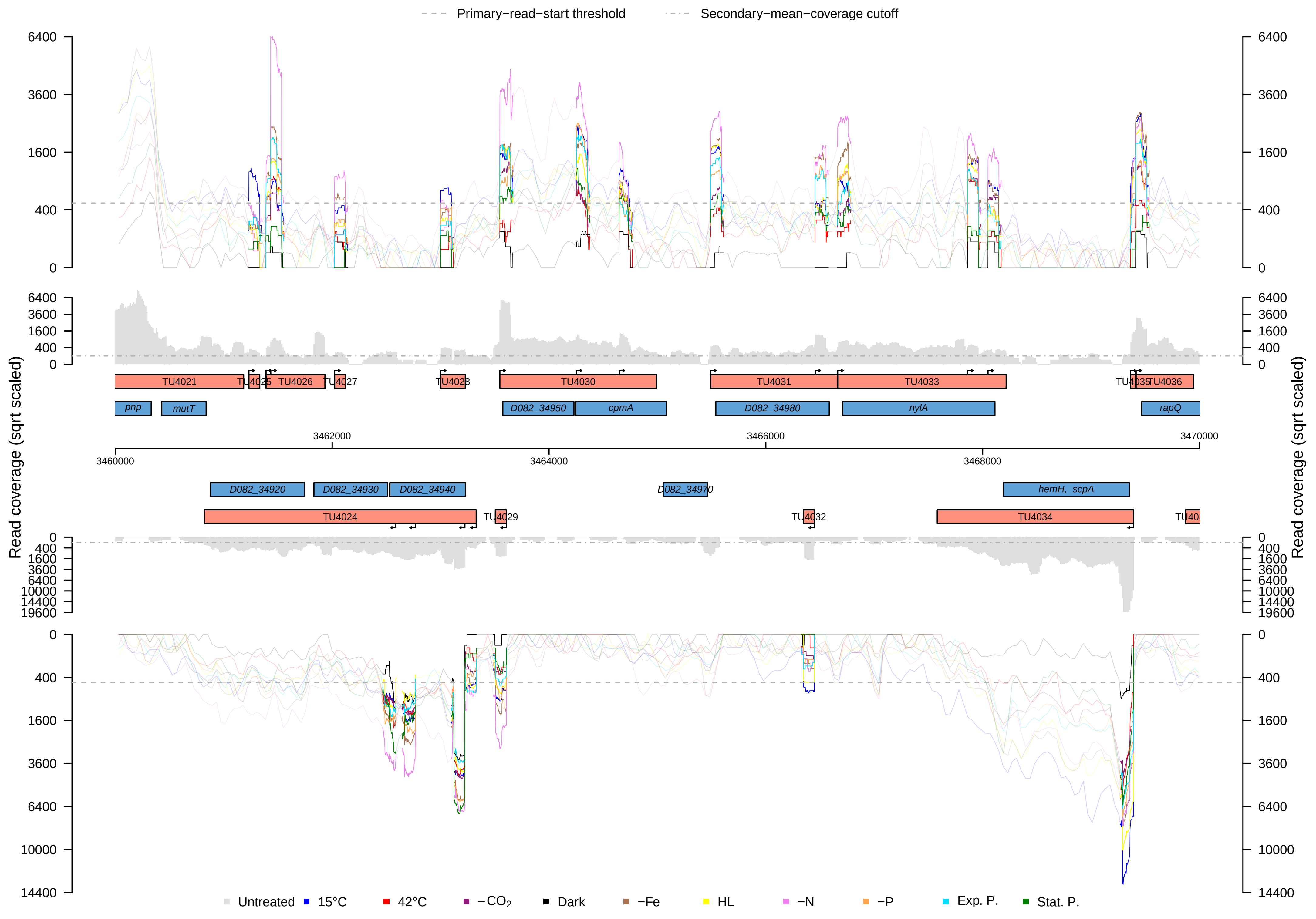

Primary−read−start threshold
Secondary−mean−coverage cutoff
Read coverage (sqrt scaled)
TU4021
TU4025
TU4026
TU4027
TU4028
TU4030
TU4031
TU4033
TU4035
TU4036
pnp
mutT
D082_34950
cpmA
D082_34980
nylA
rapQ
3460000
3462000
3464000
3466000
3468000
3470000
D082_34920
D082_34930
D082_34940
D082_34970
hemH, scpA
TU4024
TU4029
TU4032
TU4034
Untreated
15°C
42°C
−CO2
Dark
−Fe
HL
−N
−P
Exp. P.
Stat. P.

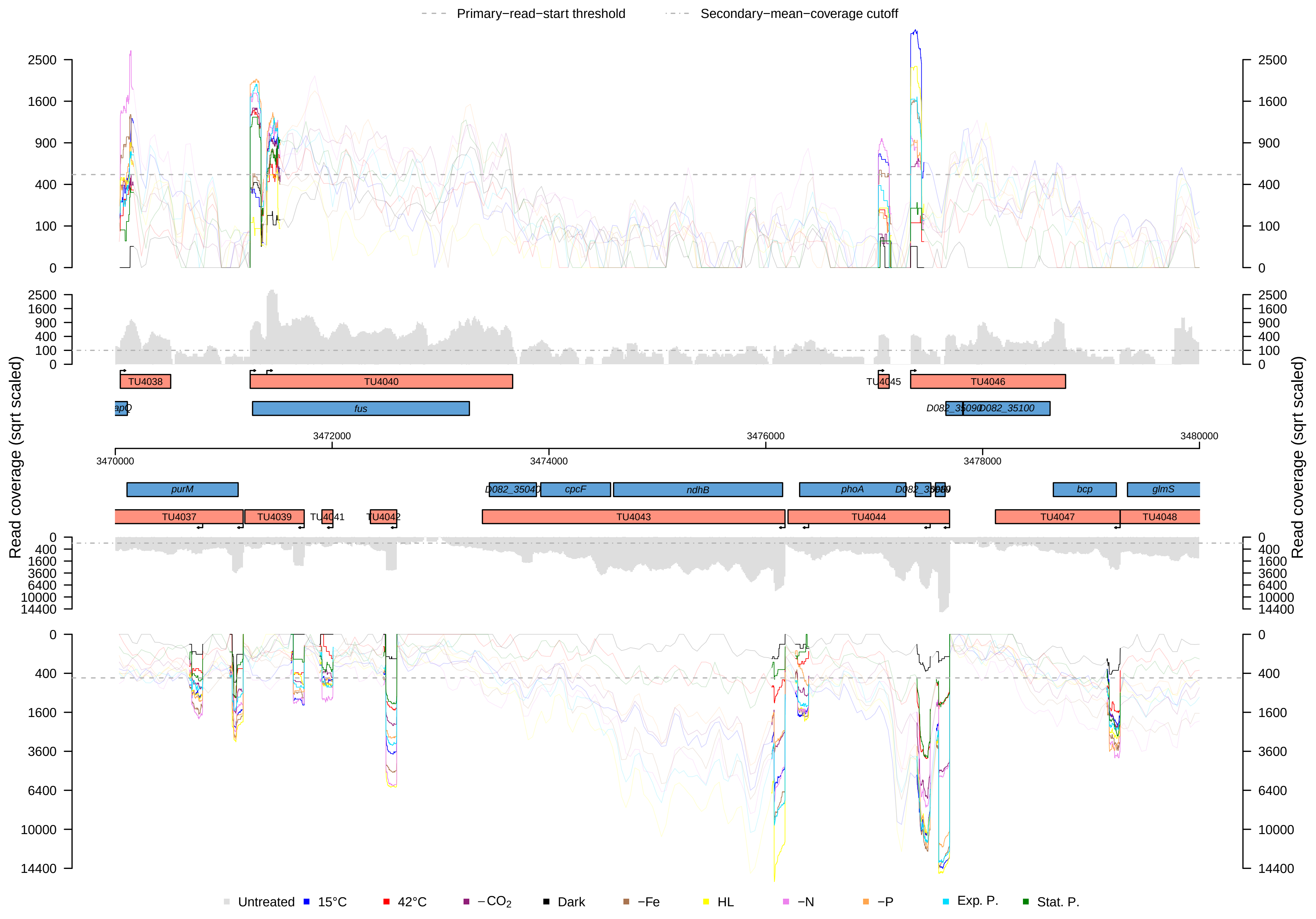

Primary−read−start threshold
Secondary−mean−coverage cutoff
Read coverage (sqrt scaled)
TU4038
TU4040
TU4045
TU4046
fus
D082_35090
D082_35100
3470000
3472000
3474000
3476000
3478000
3480000
purM
D082_35040
cpcF
ndhB
phoA
bcp
glmS
TU4037
TU4039
TU4041
TU4042
TU4043
TU4044
TU4047
TU4048
Untreated
15°C
42°C
−CO2
Dark
−Fe
HL
−N
−P
Exp. P.
Stat. P.

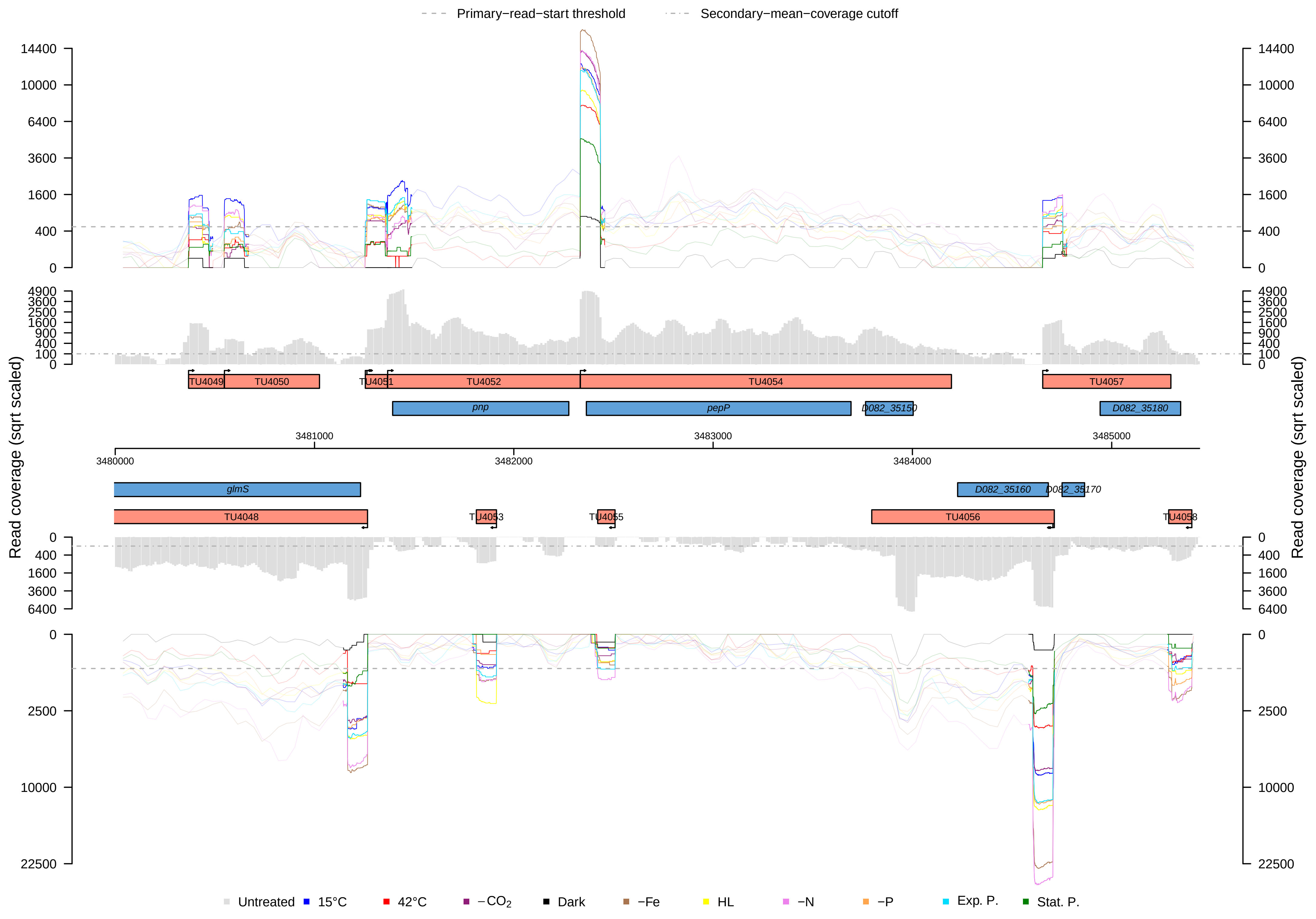

Primary−read−start threshold
Secondary−mean−coverage cutoff
Read coverage (sqrt scaled)
14400
10000
6400
3600
1600
400
0
4900
3600
2500
1600
900
400
100
TU4049
TU4050
TU4051
TU4052
TU4054
TU4057
pnp
pepP
D082_35150
D082_35180
3480000
3481000
3482000
3483000
3484000
3485000
glmS
D082_35160
D082_35170
TU4048
TU4053
TU4055
TU4056
TU4058
2500
10000
22500
Untreated
15°C
42°C
−CO2
Dark
−Fe
HL
−N
−P
Exp. P.
Stat. P.
