# Supplementary material for: Variations in the non-coding transcriptome as a driver of inter-strain divergence and physiological adaptation in bacteria
Source: Sci Rep. 2015 Apr 22;5:9560. doi: 10.1038/srep09560 (PMC5386190; doi:10.1038/srep09560)
Supplement: Supplementary Information — Supplementary Figures [file srep09560-s5.pdf]

Variations in the non-coding transcriptome as a driver of inter-strain divergence and physiological adaptation in bacteria

Matthias Kopf*, Stephan Klähn*, Ingeborg Scholz, Wolfgang R. Hess[§] and Björn Voß

Genetics and Experimental Bioinformatics, Faculty of Biology, University of Freiburg, Schänzlestr. 1, 79104 Freiburg, Germany

[§]Corresponding author

*shared first authors

Supplementary Figures S1- S4

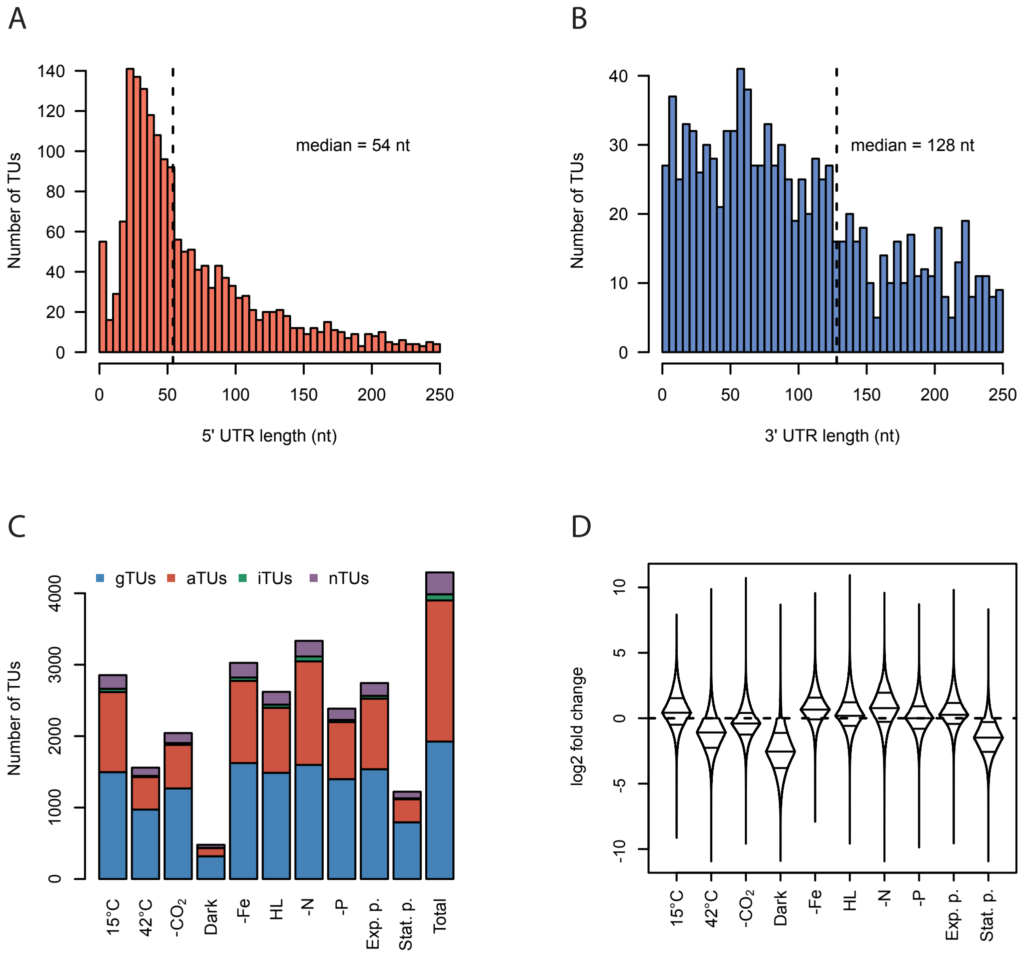

Figure S1 I Analysis of UTR lengths and the different types of TUs for *Synechocystis* 6714.

(A) Histogram of the 5' UTR lengths in *Synechocystis* 6714 (bin size 5 nt). (B) Histogram of the 3' UTR lengths in *Synechocystis* 6714 (bin size 5 nt). (C) The number of TU types for different growth conditions in *Synechocystis* 6714. (D) Box-percentile plot of all pairwise fold changes of all TUs for each condition.

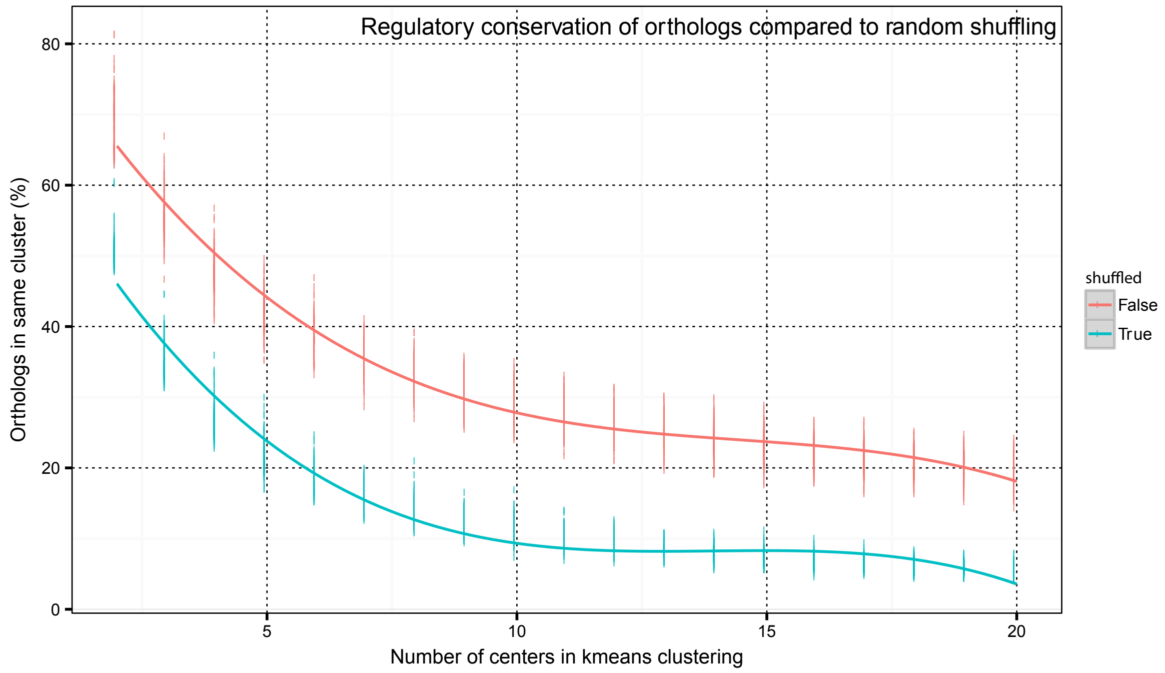

Figure S2 I Recapturing of orthologs in expression-based clustering. Combined k-means clustering of all orthologous TUs (red line) and randomly paired TUs (green line) based on their expression profiles for 2 to 20 centers. The clustering was recalculated for all possible combinations of 5 out of the 10 sequenced conditions.

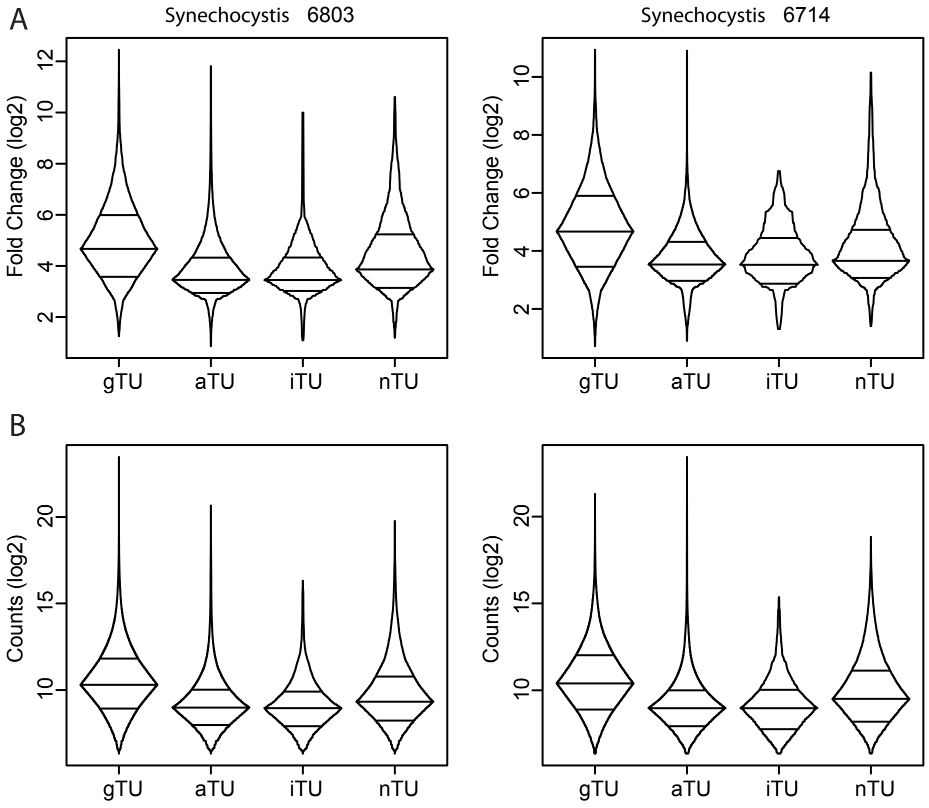

Figure S3 I Box-percentile plots.
(A) Maximum fold change measured between any pair of conditions for each predicted TU in *Synechocystis* 6803 and 6714. (B) Distribution of log2 expression levels for the different TU types.

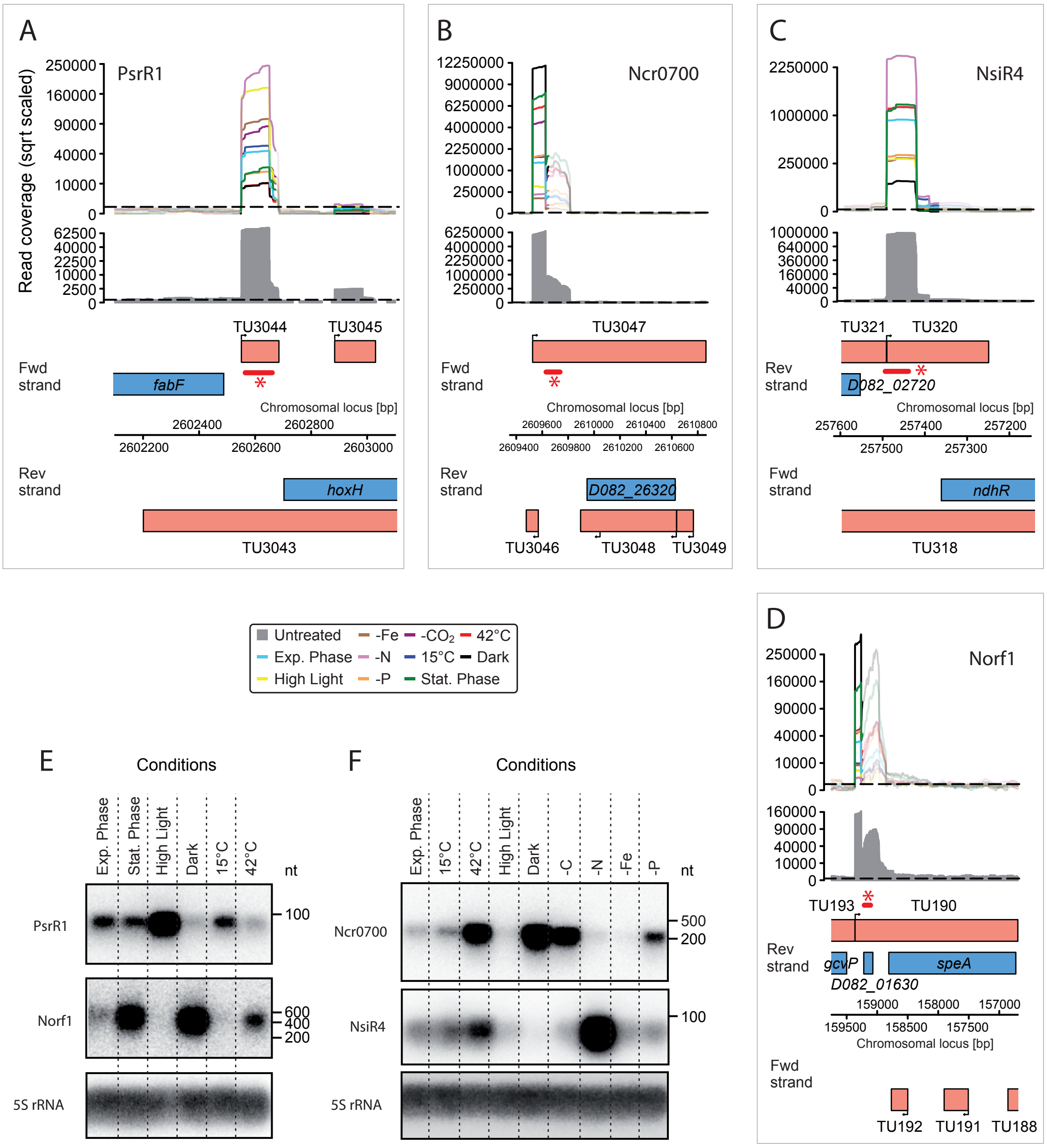

Figure S4 I Verification of selected transcripts in *Synechocystis* 6714. Genome plots (A-D) and Northern blot verifications (E+F) for PsrR1, Norf1, Ncr0700 and NsiR4 which have been previously described for *Synechocystis* 6803[19]. *The bar in each plot marks the location of the ^{32}P-labelled probe. The membranes were verified for equal loading in a separate hybridization for 5S rRNA.

[19]Kopf, M. *et al.* Comparative Analysis of the Primary Transcriptome of Synechocystis sp. PCC 6803. DNA Res. **21**, 527–539 (2014).
