# Supplementary material for: Variations in the non-coding transcriptome as a driver of inter-strain divergence and physiological adaptation in bacteria
Source: Sci Rep. 2015 Apr 22;5:9560. doi: 10.1038/srep09560 (PMC5386190; doi:10.1038/srep09560)
Supplement: Supplementary Information — Supporting File 4 [file srep09560-s4.pdf]

# Variations in the non-coding transcriptome as a driver of inter-strain divergence and physiological adaptation in bacteria

**Matthias Kopf*, Stephan Klähn*, Ingeborg Scholz, Wolfgang R. Hess[§] and Björn Voß**
Genetics and Experimental Bioinformatics, Faculty of Biology, University of Freiburg,
Schänzlestr. 1, 79104 Freiburg, Germany
[§]Corresponding author
*shared first authors

## Supporting file S4 | Visualization of the dRNA-seq data for the plasmid pSYLC of *Synechocystis* sp. PCC 6714.

The colored graphs represent the coverage of reads from the treated libraries for ten sequenced conditions. The corresponding colors are given at the bottom of each page. The TSS positions are indicated by black arrows, e.g. at the beginning of each predicted transcriptional unit (TU, shown in red), and the read coverage graphs are highlighted for the following 100 bp. The coverage from the untreated library is shown grey. The y-axis of the graphs is square-root-scaled but the tick labels show the true normalized coverage values. Protein coding genes are shown in blue, tRNA in green and rRNA in dark grey. The data for the forward strand is shown above and data for the reverse strand below the x-axis. The x-axis shows the nucleotide position of the plasmid pSYLC in bp. This document is searchable by locus tags from *Synechocystis* sp. PCC 6714, gene names (if available), nucleotide position (every 2000 bp) and the *Synechocystis* sp. PCC 6803 locus tags of the 2854 orthologous protein-coding genes.

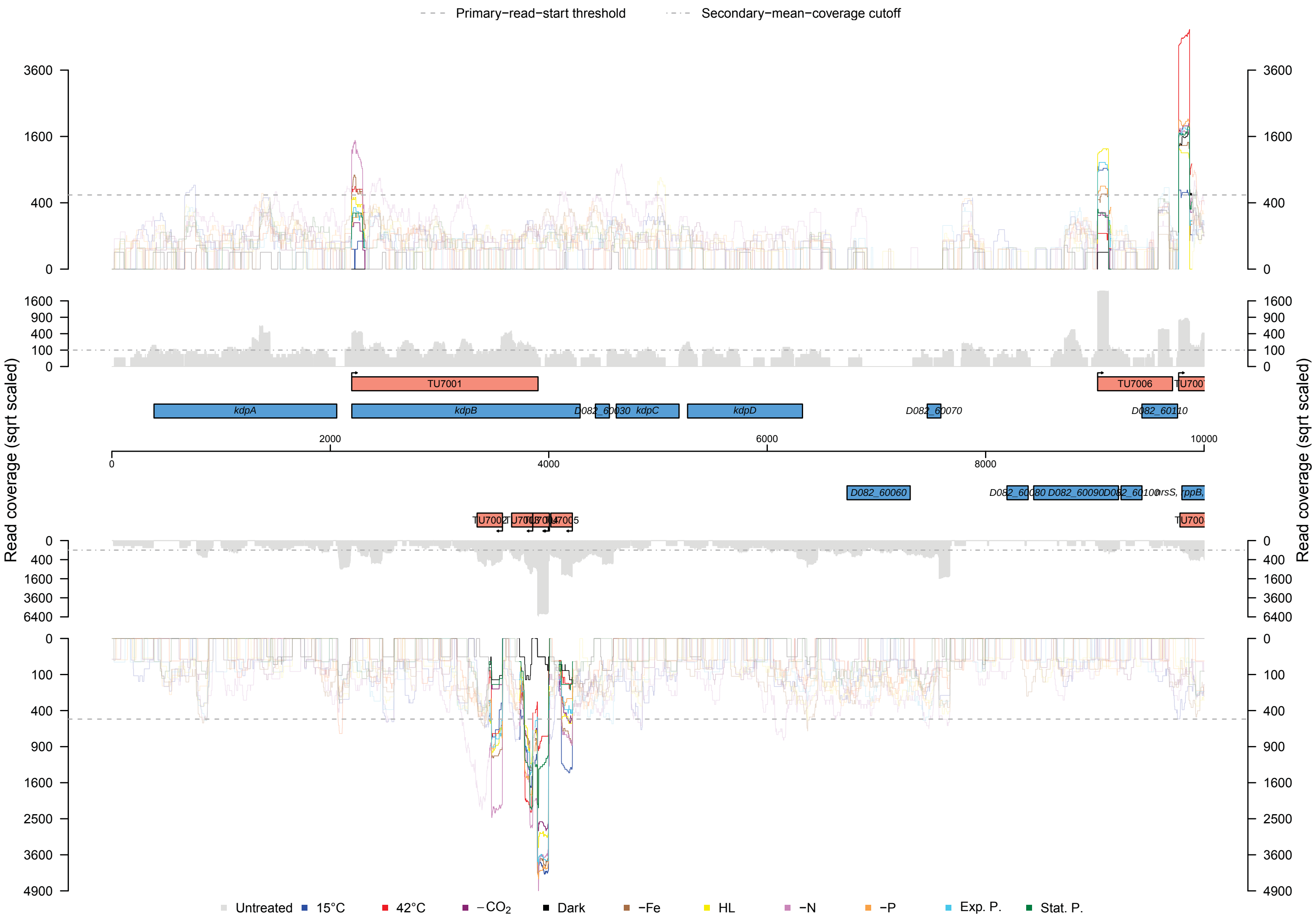

Primary−read−start threshold
Secondary−mean−coverage cutoff
Read coverage (sqrt scaled)
3600
1600
400
0
900
100
TU7001
TU7006
kdpA
kdpB
kdpC
kdpD
D082_60070
D082_60110
0
2000
4000
6000
8000
10000
D082_60060
D082_60090
TU7002
TU7005
6400
2500
4900
Untreated
15°C
42°C
−CO2
Dark
−Fe
HL
−N
−P
Exp. P.
Stat. P.

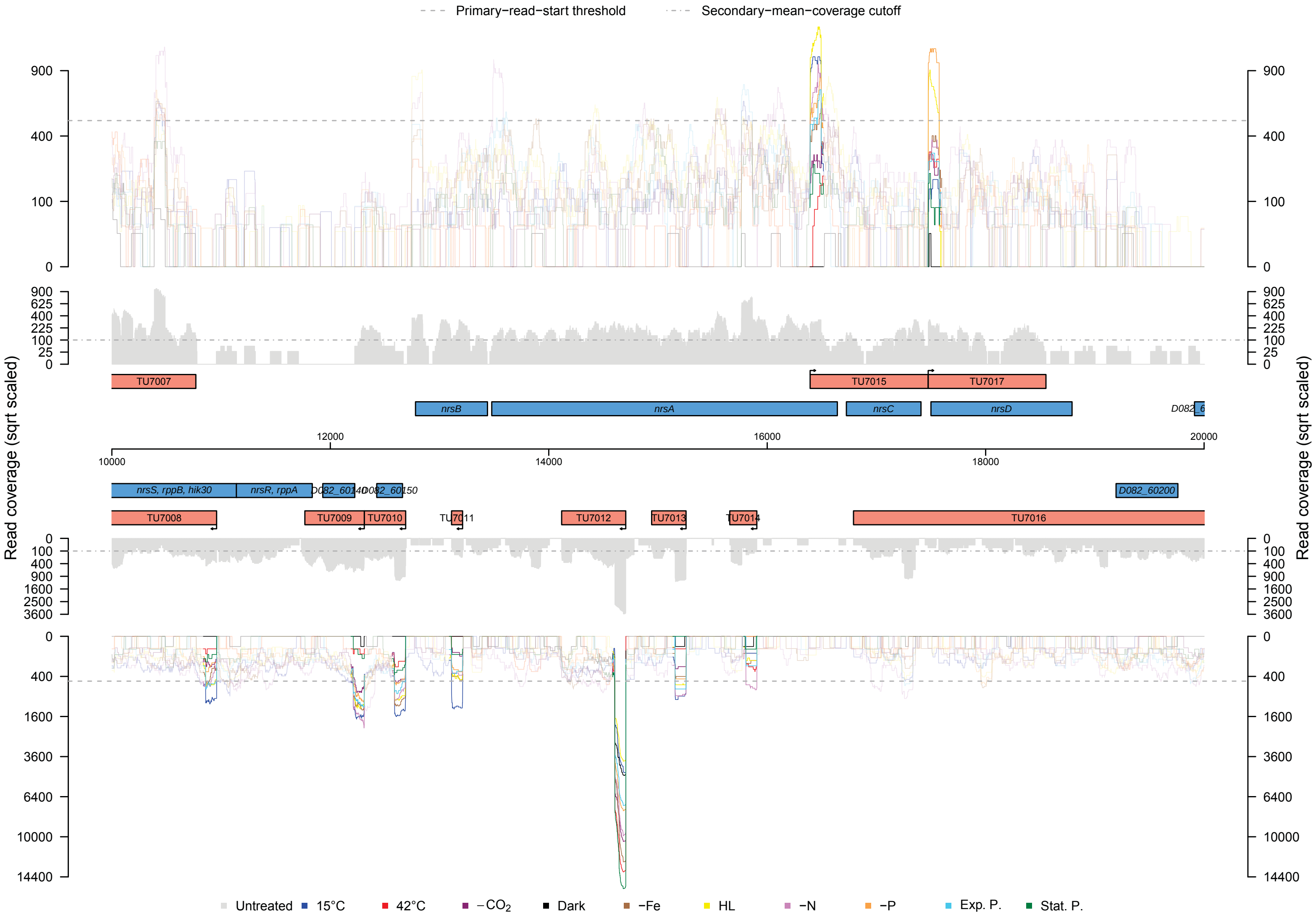

Primary−read−start threshold
Secondary−mean−coverage cutoff
Read coverage (sqrt scaled)
900
400
100
0
625
225
25
TU7007
TU7015
TU7017
nrsB
nrsA
nrsC
nrsD
10000
12000
14000
16000
18000
20000
nrsS, rppB, hik30
nrsR, rppA
D082_60140
D082_60150
D082_60200
TU7008
TU7009
TU7010
TU7011
TU7012
TU7013
TU7014
TU7016
1600
2500
3600
6400
10000
14400
Untreated
15°C
42°C
−CO2
Dark
−Fe
HL
−N
−P
Exp. P.
Stat. P.

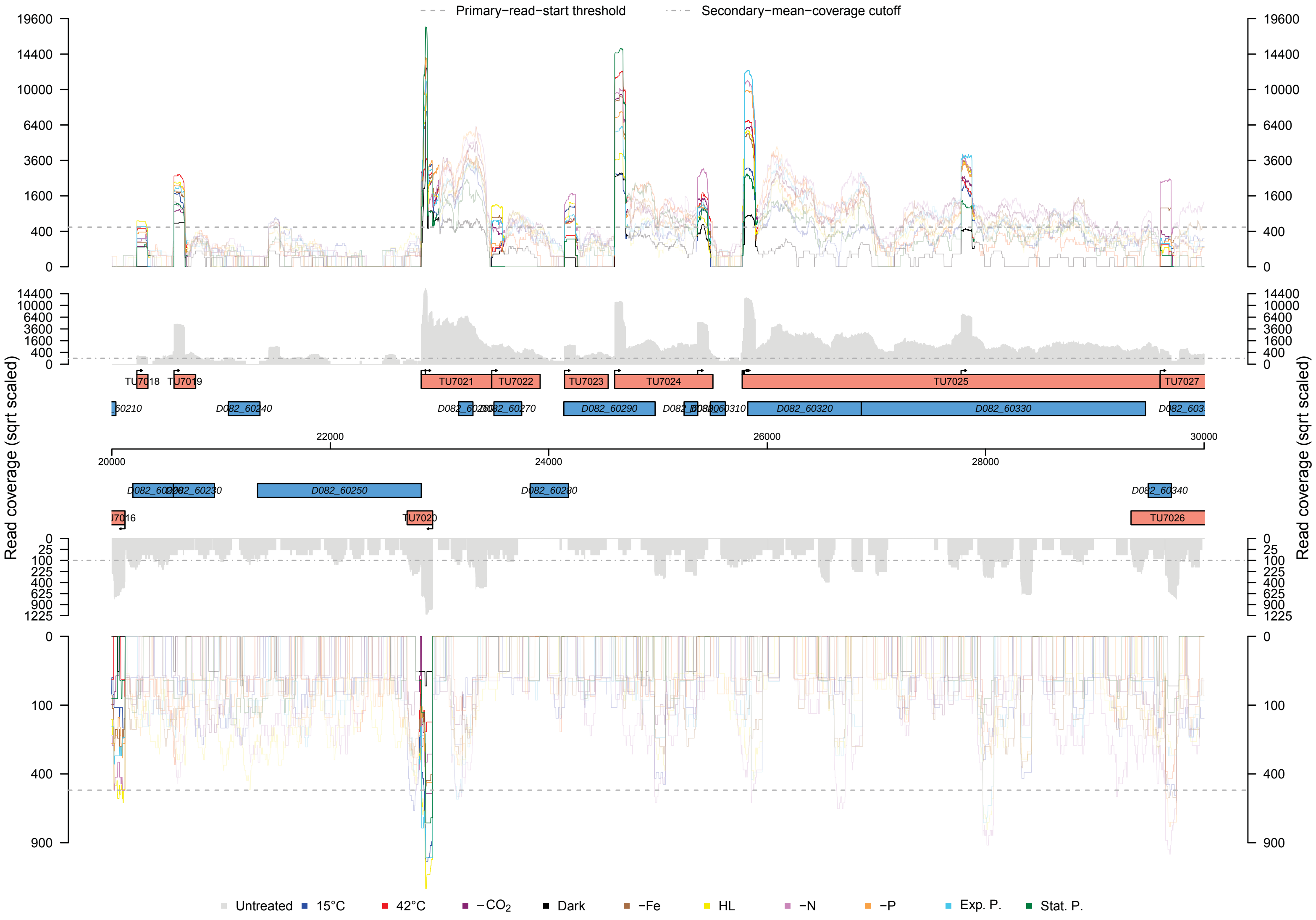

Primary−read−start threshold
Secondary−mean−coverage cutoff
Read coverage (sqrt scaled)
19600
14400
10000
6400
3600
1600
400
0
TU7018
TU7019
TU7021
TU7022
TU7023
TU7024
TU7025
TU7027
D082_60240
D082_60270
D082_60290
D082_60320
D082_60330
20000
22000
24000
26000
28000
30000
D082_60250
D082_60280
D082_60340
TU7020
TU7026
1225
900
625
225
100
25
Untreated
15°C
42°C
−CO2
Dark
−Fe
HL
−N
−P
Exp. P.
Stat. P.

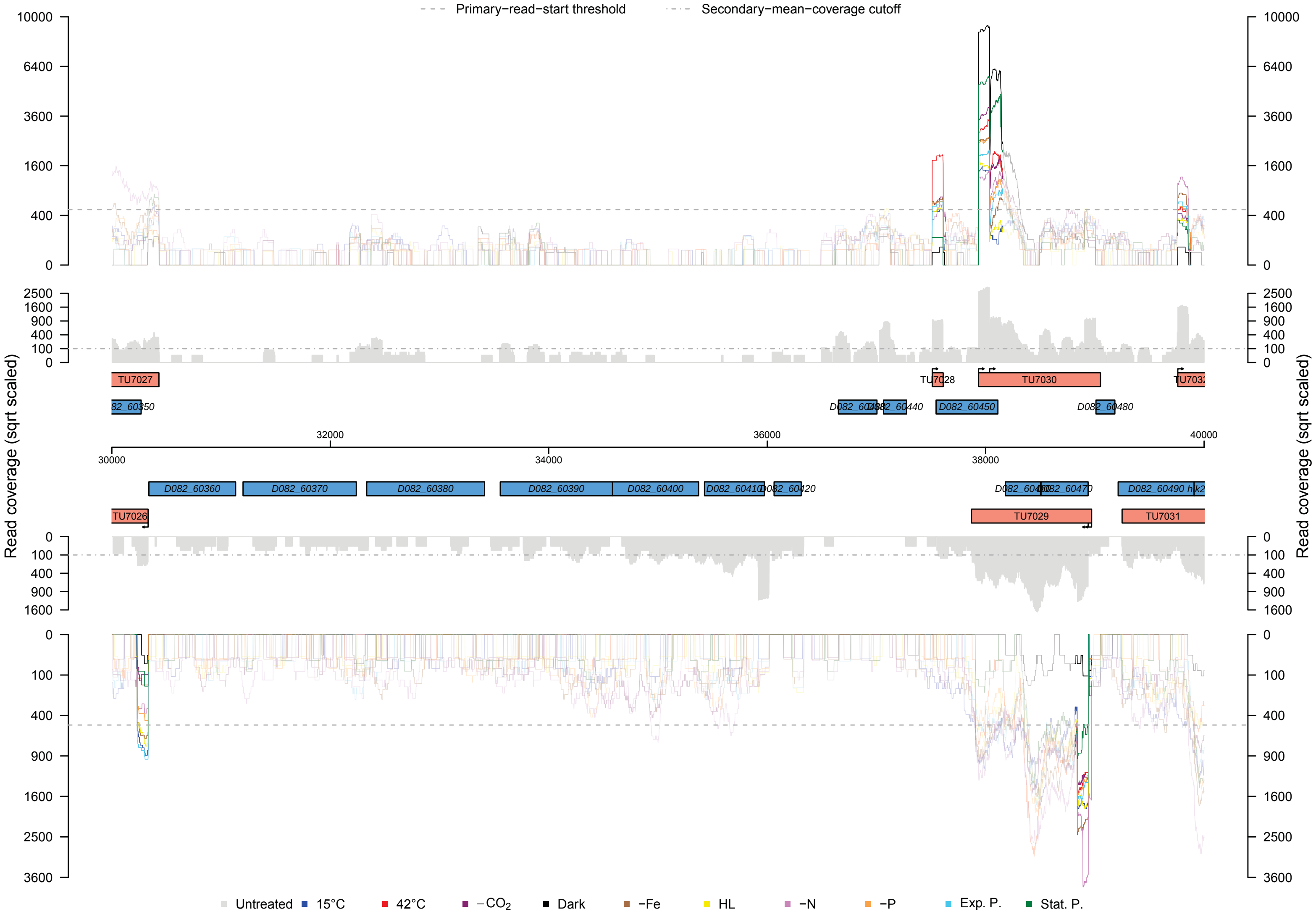

Primary−read−start threshold
Secondary−mean−coverage cutoff
Read coverage (sqrt scaled)
10000
6400
3600
1600
400
0
2500
1600
900
400
100
0
TU7027
TU7028
TU7030
82_60350
D082_60450
D082_60480
32000
36000
40000
30000
34000
38000
D082_60360
D082_60370
D082_60380
D082_60390
D082_60400
D082_60490
TU7026
TU7029
TU7031
Untreated
15°C
42°C
−CO2
Dark
−Fe
HL
−N
−P
Exp. P.
Stat. P.

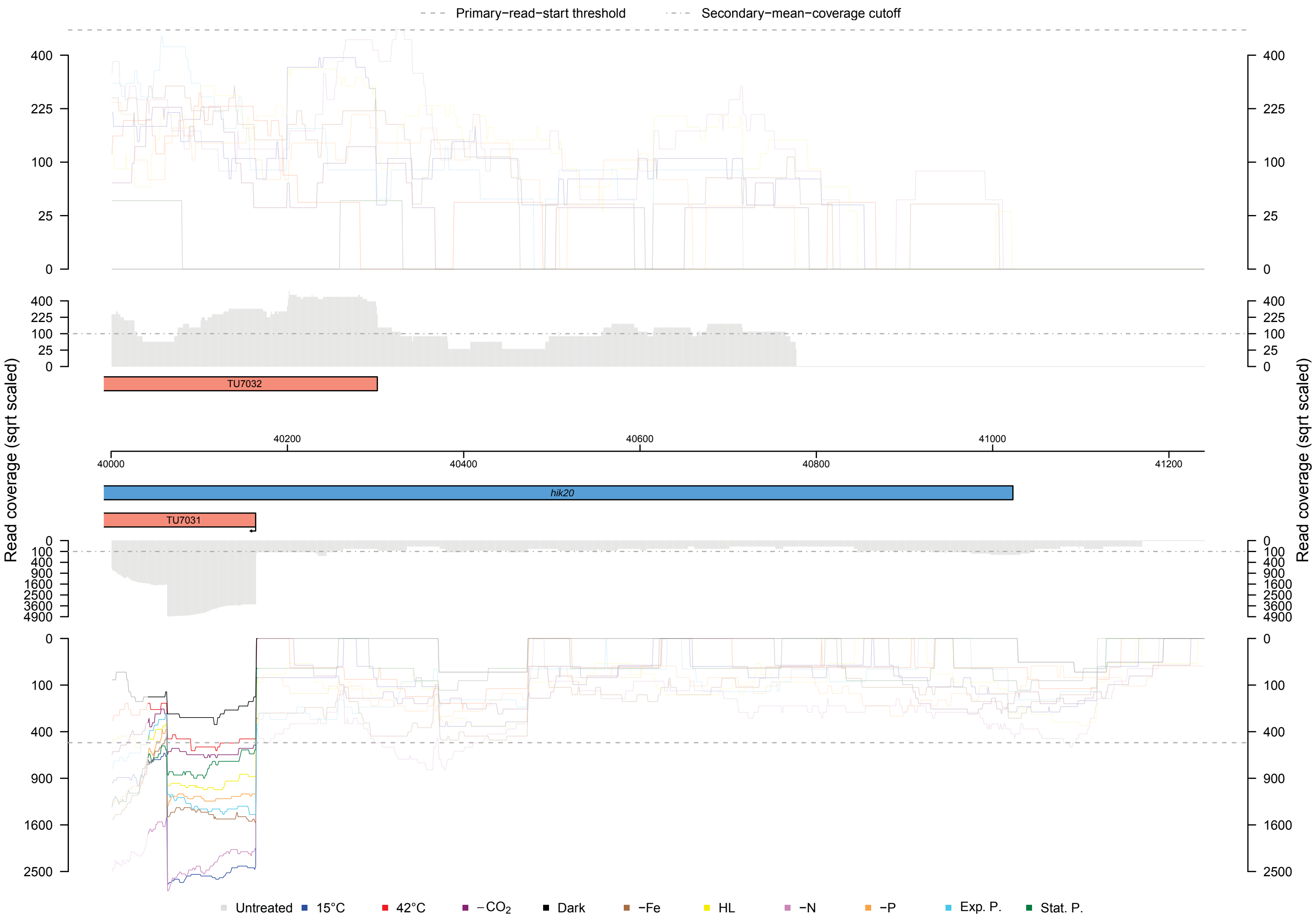

Primary−read−start threshold
Secondary−mean−coverage cutoff
Read coverage (sqrt scaled)
400
225
100
25
0
TU7032
40000
40200
40400
40600
40800
41000
41200
hik20
TU7031
100
400
900
1600
2500
3600
4900
Untreated
15°C
42°C
−CO2
Dark
−Fe
HL
−N
−P
Exp. P.
Stat. P.
